# Effectiveness of clinical decision support in fall prevention among older adults: A systematic review and meta-analysis

**DOI:** 10.1371/journal.pone.0340025

**Published:** 2026-01-12

**Authors:** Rune Solli, Nina Rydland Olsen, Linda Aimée Hartford Kvæl, Stijn Van de Velde, Are Hugo Pripp, Signe Agnes Flottorp, Therese Brovold

**Affiliations:** 1 Department of Rehabilitation Science and Health Technology, Faculty of Health Sciences, OsloMet – Oslo Metropolitan University, Oslo, Norway; 2 Department of Health and Functioning, Western Norway University of Applied Sciences, Faculty of health and social sciences, Bergen, Norway; 3 Norwegian Social Research (NOVA), OsloMet – Oslo Metropolitan University, Oslo, Norway; 4 MAGIC Evidence Ecosystem Foundation; Research Department, Lovisenberg Diaconal Hospital, Oslo, Norway; 5 Division of Health Services, Norwegian Institute of Public Health, Oslo, Norway; University Hospital Cologne: Uniklinik Koln, GERMANY

## Abstract

**Background:**

Systematic use of Clinical Decision Support (CDS), which provides timely information to assist healthcare practitioners in decision-making, is recommended in the implementation of fall prevention among older adults. This systematic review aimed to evaluate the effects of CDS for fall prevention on healthcare practitioners’ adherence to recommended practice, medication outcomes, and patient outcomes.

**Methods:**

We searched Medline, EMBASE, CINAHL, Cochrane Library, Web of Science, AMED, PEDro, and Google Scholar from the earliest available dates through January 2025. We included randomised and non-randomised studies that directly compared interventions consisting of CDS presented on-screen or on paper to healthcare practitioners aiming to prevent falls in persons aged 65 years or older. We analysed healthcare practitioner performance, medication review and prescribing, fall risk, fall rate, and fall injury rate as primary outcomes. Two reviewers independently screened studies and assessed for risk of bias. We synthesised results using meta-analyses and vote-counting based on direction of effect, when possible, otherwise narratively, and we rated the certainty of the evidence using the GRADE approach.

**Results:**

Of 25 included studies, 20 were randomised and five were non-randomised. Most CDS tools supported healthcare practitioners in performing multifactorial fall risk assessments and follow-up interventions based on identified risks (60%) and most were delivered electronically (60%). CDS may improve healthcare practitioners’ adherence to recommended practice (all eight comparisons favouring CDS; 95% confidence interval [CI] 68% to 100%; low certainty) and likely improve medication review and prescribing (all nine comparisons favouring CDS; 95% CI 70% to 100%; moderate certainty), although the effect sizes are uncertain. CDS may reduce fall risk, but the effect may be small (odds ratio 0.93; 95% CI 0.81 to 1.01; low certainty). CDS likely reduces fall rates in hospitals or residential care (rate ratio [RaR] 0.74; 95% CI 0.63 to 0.88; moderate certainty) and in patients aged 80 years or older (RaR 0.72; 95% CI 0.61 to 0.86; moderate certainty). CDS may reduce fall rates in community-dwelling older adults (RaR 0.97; 95% CI 0.93 to 1.00; moderate certainty) and in patients aged between 65 and 80 years (RaR 0.92; 95% CI 0.84 to 1.01; low certainty), though the effects in both of these subgroups may be small. CDS may reduce fall injury rates in older adults aged between 65 and 80 years (RaR 0.80; 95% CI 0.59 to 1.09; low certainty). The evidence on fall injury rates in patients aged 80 years or older was very uncertain.

**Conclusion:**

CDS likely enhances healthcare practitioners’ performance in fall prevention among older adults; however, the effect sizes remain unknown. Although CDS may improve patient outcomes in fall prevention, both the effect sizes and the certainty of evidence vary. Results from this study may inform the planning and implementation of CDS in fall prevention. Future studies should strive for clearer reporting of CDS design factors to allow for an evaluation of which factors may influence the success of CDS interventions in fall prevention.

**Trial registration:**

**Registration:** PROSPERO, CRD42021250500.

## Introduction

Falls are a major cause of morbidity and mortality among community-dwelling older adults aged 65 years or older [1–3]. Annually, one in three older adults experiences a fall, which can lead to significant health loss and increased care needs [1–5]. Globally, fall-related injuries are one of the most expensive conditions in terms of economic expenditures [4–6]. Identified risk factors for falls include a history of falls, higher age, female sex, and fear of falling [7,8]. A range of cost-effective interventions has been shown to prevent falls and reduce the incidence of fall-related injuries in older adults [9–11]. These interventions include muscle strength and balance training, home safety assessments and modifications, and medication adjustments [9,10]. Falls are often underreported, as older adults may not report falls unprompted [12,13]. The World Falls Guidelines 2022 (WFG2022) [14] emphasise the need to identify older adults at increased fall risk. They recommend that all older adults receive advise on fall prevention and physical activity.

Despite scientific support for the implementation of fall-prevention recommendations for older adults, the systematic uptake of evidence-based fall prevention practices has been slow [14–17] Consequently, fall rates and fall-related mortality have not declined [3,18,19]. The WFG2022 recommend the systematic use of Clinical Decision Support (CDS) in fall prevention to identify older adults at increased risk of falling and to facilitate fall risk assessments and interventions. CDS is an implementation strategy found to improve healthcare practitioners’ adherence to clinical guidelines [20]. CDS is defined as computerised or non-computerised tools that combine health-related and medical information with individual patient information to support clinical decision-making [21,22]. CDS has the potential to assist healthcare practitioners in identifying older adults at increased fall risk [23–25] as well as in individualising fall prevention interventions based on identified risk factors [24,26–28].

Several systematic reviews have evaluated the effects of CDS used by healthcare practitioners across a variety of settings, demonstrating small to moderate positive effects [20,29–37]. Generally, the use of CDS may improve the implementation of clinical practice guidelines [20] and the performance of nurses and allied health professionals [29,34,35]. In addition, CDS has been shown to improve hospital care for older patients [30], medication outcomes in older adults [31,32], and medication outcomes in adults more broadly [36,37]. The effect of CDS on patient outcomes, however, appears less certain [35]. While CDS may not significantly affect mortality, it may moderately improve morbidity outcomes [33] and a variety of other patient outcomes [36]. Two systematic reviews found that CDS may help prevent falls in nursing homes [29] and in hospitals [30]. However, these reviews did not include several known studies on the effects of CDS in fall prevention [38,39], and no statistical syntheses were conducted. Overall, findings from systematic reviews indicate that CDS may provide important benefits for healthcare practitioners’ performance and patient outcomes. However, no systematic review to date has specifically investigated the effects of CDS on healthcare practitioners’ performance and patient outcomes in the context of fall prevention.

### Objectives

This systematic review aimed to evaluate the effects of CDS for fall prevention on healthcare practitioners’ adherence to recommended practice, medication outcomes, and on patient outcomes.

## Materials and methods

### Protocol and registration

The review protocol was drafted and finalised in accordance with the Preferred Reporting Items for Systematic Reviews and Meta-Analyses (PRISMA) extension for study protocols (PRISMA-P) [40] and was registered in the PROSPERO international prospective register of systematic reviews (identification number CRD42021250500) prior to commencing the systematic review. We followed the recommendations in the Cochrane Handbook [41] and the guidance from the Cochrane Effective Practice and Organisation of Care (EPOC) [42]. Reporting of this systematic review adhered to the standards of the Preferred Reporting Items for Systematic Reviews and Meta-Analyses (PRISMA) [43]. See S1 Table for the populated PRISMA checklist. See S2 Table for differences between the protocol and the review.

### Eligibility criteria

We included studies evaluating the effects of CDS interventions designed to support HCPs in making decisions to prevent falls in older adults. Studies conducted in any healthcare setting or in the homes of older adults were eligible for inclusion. We included randomised and non-randomised controlled trials, controlled before-after studies, and interrupted time-series studies. Detailed eligibility criteria are presented in Table 1.

**Table 1:** Study eligibility criteria.

	Inclusion criteria	Exclusion criteria
Participants	Healthcare practitioners (nurses, physiotherapists, general practitioners, occupational therapists, nursing assistants, pharmacists, physicians, primary care providers, paramedics)	Studies of CDS used by students.
Intervention	CDS used by healthcare practitioners in fall prevention interventions. We included both computerised and non-computerised CDS interventions.	Studies where CDS was not part of the intervention in at least one study group or arm.
Comparison	Usual care or no treatment.	
Outcomes	We included studies that reported at least one primary or secondary outcome. Primary: • Healthcare practitioner performance: Adherence to recommended practice and adherence to recommended medication prescribing and review. • Patient outcomes: Rate of falls (i.e., number of falls per unit of follow-up time); risk of falling (i.e., the number of older adults who had one or more falls); and fall injuries. Secondary: • Deaths and hospitalisations.	Studies that did not report at least one primary or secondary outcome.
Design	RCTs: IRPGT and CRT; NRCTs; CBA studies; and ITS studies [42].	Uncontrolled before-after, cross-sectional, case-control, and cohort studies; as well as protocols, editorials, opinion papers, and conference abstracts. Studies without a control group, except for ITS studies with multiple data points collected before and after the implementation of the intervention.
Setting	Any healthcare setting or in the homes of older adults.	Studies conducted in a setting other than a healthcare institution or in the homes of older adults.

CDS: Clinical Decision Support; RCT: Randomised controlled trial; IRPGT: Individually-randomised parallel-group trial; CRT: Cluster-randomised trial; NRCT: Non-randomised controlled trial; CBA: Controlled before-after study; ITS: Interrupted time-series.

### Information sources and search strategy

We searched the following databases from the earliest available date through January 2025: MEDLINE (Ovid), EMBASE (Ovid), CINAHL (EBSCOhost), The Cochrane Library, Web of Science, and AMED (Ovid). Supplemental searches were performed in Google Scholar (S3 Table). The literature search strategy was developed in cooperation with two librarians from the Literature Search Resource/Expert Group at Oslo Metropolitan University (OsloMet). The searches were developed to be highly sensitive and included terms synonymous with the fundamental concepts underlying CDS (e.g., decision rules, reminder systems, algorithms), falls (e.g., accidental falls, slip), and fall prevention (e.g., accident prevention, safety management). We did not impose any language or study design restrictions on the literature searches. To maximise the sensitivity of the search and capture a broader range of potentially relevant studies, study design was not restricted in the search strategy. One reviewer (RS) used the advanced search feature in the Physiotherapy Evidence Database (PEDro) to conduct manual searches for relevant clinical trials. Additionally, we searched the reference lists of the included articles and relevant systematic reviews. Cited reference searching for all included articles was carried out in Web of Science. See S3 Table for the complete search strategy.

### Selection process

Search results were imported into EndNote, where duplicates were removed. Next, all unique abstracts and full text articles were uploaded to the Covidence systematic review software [44]. Two reviewers (RS and either TB, NRO, or LAHK) independently screened titles, abstracts, and full texts against the eligibility criteria. Any disagreements were resolved through consensus or, if needed, by the decision of a third reviewer. Consensus between reviewers was required at both the title and abstract screening stage, as well as during the full-text screening stage.

### Data collection process

We used an adapted version of the EPOC data collection form to extract relevant data from the included studies. The data extraction form was piloted on three reports by two reviewers. One reviewer (RS) independently extracted the data, and another reviewer (TB) checked the extracted data against five arbitrarily selected papers. Any disagreements between reviewers were resolved by consensus, with consultation from a third reviewer if necessary. To address missing outcome data, we contacted the corresponding authors of eight included reports via email. If no responses were received, follow-up emails were sent after two weeks, with a maximum of three email attempts per author. Our efforts to obtain missing data were successful in four instances. All data used in the analyses were obtained directly from primary sources or through successful correspondence with authors, with no data imputation performed.

### Data items

#### Outcomes.

For healthcare practitioner performance, we sought data on adherence to recommended practices, including the provision of specific advice, delivery of specific interventions, and adherence to referral guidelines [42]. We also collected data on medication outcomes, including medication review [45] and prescribing. For patient outcomes, we included fall risk, fall rate, and fall injury rate. Fall risk refers to the proportion of individuals who experienced at least one fall over a specific period, representing the likelihood of falling at the individual level. Fall rate and fall injury rate refer to the total number of falls or fall injuries per unit of time, e.g., per 1,000 person-years, accounting for multiple falls per individual. Fall injuries included both minor injuries, such as bruises or abrasions, and serious injuries, such as fractures or those requiring medical attention [46]. We also sought data for deaths and hospitalisations. If multiple results were reported in a study, we prioritised the primary outcome as specified by the authors or the outcome used for sample size calculation. If primary outcomes were not clearly identified, we prioritised results deemed to be most relevant to the research question, those indicating drug overuse rather than misuse or underuse (due to the significant fall risk imposed by polypharmacy [47]), and results based on whole-group analyses rather than subgroup analyses.

#### Study characteristics.

We extracted the following data on study characteristics: authors, publication year, funding, country, design, setting, duration, healthcare practitioners delivering the intervention (type and number), and patients receiving the intervention (number, sex, age).

#### Interventions and CDS.

We extracted the characteristics of experimental and control interventions using the Template for Intervention Description and Replication (TIDieR) checklist [48], and using domains 1 and 3 from the intervention complexity assessment tool for systematic reviews (iCAT_SR) [49]. Interventions were categorised into 1) manual fall risk assessment and interventions based on CDS; 2) medication review and recommendations made to physician; 3) automatically generated fall risk based on prediction models and recommended interventions; 4) guided medication dosing using computerised CDS; and 5) computerised CDS presented to paramedics on hand-held tablets [50,51]. Design factors of CDS tools were extracted using the GUideline Implementation with DEcision Support (GUIDES) framework [51] and elements from the two-stream model [52].

### Study risk of bias assessment

Two review authors independently assessed risk of bias (RoB) for outcomes indicating healthcare practitioner performance, fall rates, fall risk, and fall injuries. We used the Revised Cochrane Risk of Bias tool for randomised trials (RoB 2.0) [53] for included RCTs, and the Risk Of Bias In Non-randomised Studies of Interventions (ROBINS-I) tool [54,55] for included non-randomised studies. Any disagreements between reviewers were resolved by consensus, and if necessary, a third reviewer was consulted.

### Synthesis methods and effect measures

#### Healthcare practitioner performance.

We performed two analyses related to healthcare practitioner performance, both of which used vote-counting based on direction of effect [41,56]. In the analysis of adherence to recommended practice, we included outcomes indicating the use of recommended intervention components or adherence to the intervention protocol or to the referral guideline [42]. In the analysis of medication outcomes, we included outcomes indicating changes in the use of fall-risk-increasing drugs (FRIDs), polypharmacy, drug underuse, or drug-related problems [31]. To decide which results were eligible for the analyses, we tabulated each study result and compared it against the eligibility criteria. Each effect estimate was dichotomised into ‘favouring CDS’ or ‘favouring control’, based on the observed direction of effect alone. We used the sign test for differences in proportions and presented the proportion of results favouring CDS along with 95% confidence intervals (CIs) using the Wilson interval method [57]. The data used for analyses of healthcare practitioner performance outcomes are available in S1 Appendix.

#### Patient outcomes.

Data on fall risk, fall rate, and fall injury rate were pooled into meta-analyses in line with previous work [9,58]. We assumed that underlying study effects followed a normal distribution and used random effects models and restricted maximum likelihood (REML) estimation methods [59]. Stata, version 18.0, StataCorp, College Station, Texas [60], was used for all analyses and pooled results were presented in forest plots. Results were presented as odds ratios (OR) for fall risk, and as rate ratios (RaR) for fall rate and fall injury rate, along with 95% CIs. Variation between study results (heterogeneity) was assessed by means of a visual inspection of forest plots and the I^2^ statistic [59]. If I^2^ values were 50% or higher, we sought potential explanations for the heterogeneity using post hoc subgroup analyses by RoB (low or some concerns for RoB versus high or serious RoB), study setting (community-dwelling versus hospital or residential care), and patient age (mean age of < 80 years versus ≥ 80 years). If point estimates and confidence intervals were missing but other information was available, e.g., number of events and the follow-up time in both comparison groups, we used established formulas to estimate the point estimates and confidence intervals [61]. Only one outcome per study was included in the analyses to ensure independence between studies. The data used for meta-analyses are available in S2 Appendix. Control intervention fall risks, fall rates, and fall injury rates were derived from a US report on the epidemiology of falls among older adults [62], due to the limited reporting of absolute numbers in the studies included in this review. To report the absolute effects on fall risk, odds ratios were first converted to relative risks using the formula provided in appendix 3 of the Core GRADE 2 article [63].

### Publication bias assessment

The possibility of publication bias was assessed through inspection of funnel plots of effect estimates against their standard errors for analyses that contained at least 10 effect estimates [41]. The Egger test for small-study effects and the trim-and-fill analysis were used for quantitative assessment of publication bias [64].

### Assessment of certainty of the evidence

We judged the certainty of the evidence using the Grading of Recommendations Assessment, Development and Evaluation (GRADE) approach [63,65,66], applied to each outcome from vote-counting analyses and meta-analyses. Despite extensive literature searches conducted with the assistance of two librarians at OsloMet, no minimal important difference (MID) values were identified for fall risk, fall rate, or fall injury rate. Certainty was therefore rated based on whether the true effect lies on the observed side of null, with the null effect used as the threshold. Relative and absolute effects were reported alongside plain-language statements, following recommendations from GRADE guidance [67,68].

## Results

### Study selection

**Fig 1:**
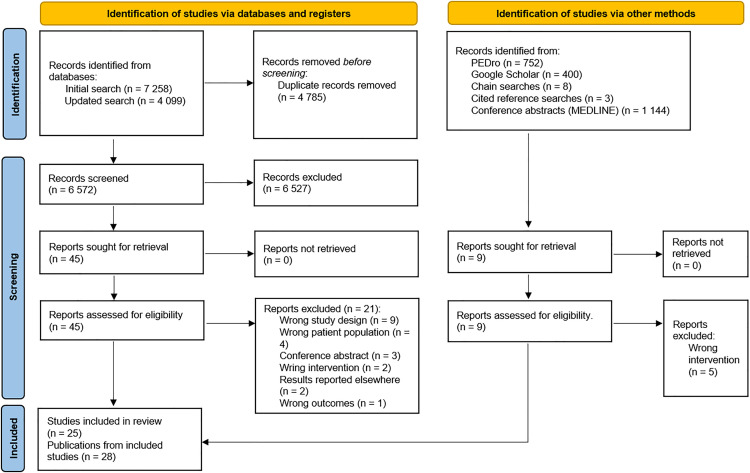
PRISMA flow diagram.

Fig 1 presents an overview of the study selection process. The primary database searches identified 6,572 unique records, of which 6,527 were excluded after title and abstract screening. A total of 28 publications describing 25 unique studies were selected for inclusion [23–28,38,39,69–88]. S4 Table provides a summary of the excluded studies and the reasons for their exclusion.

### Study characteristics

#### Study design, participants, and settings.

Table 2 presents an overview of the characteristics of the included studies. The 25 studies comprised 13 cluster-randomised trials (CRTs), seven individually randomised parallel group trials (IRPGTs), four non-randomised controlled trials (NRCTs), and one controlled before-after (CBA) study. The study duration was median (min–max) 12 (1–60) months. The interventions were primarily delivered by nurses (16 studies; 64%), physicians (15 studies; 60%), pharmacists (6 studies; 24%), and/or physiotherapists (4 studies; 16%) to a median of 1,433 patient participants, with sample sizes ranging from 312 to 46,245. Twelve studies (48%) had eligibility criteria related to an increased risk of falls, such as a history of previous falls or the use of fall-risk-increasing drugs. Most studies were conducted in hospitals (44%) or primary care practices (36%) and took place in North America (52%) or Europe (28%).

**Table 2:** Characteristics of included studies.

Author & year	Country	Design	Setting	Study duration (months)	Healthcare practitioners	Patients	Outcomes
Aizen 2015	Israel	CRT	Rehabilitation geriatric hospital	6	Nurses n = ?	Patients admitted to hospital n = 508 (200 in CDS and 308 in CG), 52.2% female, mean 84.3 years	-Rate of falls
Barker 2016	Australia	CRT	Hospital	12	Nurses n = ?	Patients admitted to hospital n = 35,264 (17,698 in CDS and 17,566 in CG), 49.5% female, median 67.5 years	-Adherence to recommended practice -Rate of falls -Rate of fall injuries
Bhasin 2020	USA	CRT	Primary care practices	44	Nurses n = ?	Community-dwelling at increased risk for fall injuries n = 5,451 (2,802 in CDS and 2,649 in CG), 62% female, mean 79.7 years	-Rate of fall injuries
Blalock 2020	USA	CRT	Community pharmacies	24	Pharmacists n = ?	Adults ≥65 years using either four or more chronic medications or ≥1 medication associated with increased fall risk, n = 3,213 (1,467 in CDS and 1,745 in CG)	-Medication outcomes -Risk of falling
Blum 2021	Switzerland, Netherlands, Belgium, Republic of Ireland	CRT	Hospitals	12	Physicians and pharmacists n = ?	Adults years with multimorbidity (≥3 chronic conditions) and polypharmacy (≥5 drugs used long term) n = 2,008 (963 in CDS and 1,045 in CG), 44.7% female, median 79 years	-Rate of falls -Medication outcomes -Mortality -Hospital admissions
Byrne 2005	USA	CBA	Nursing homes	33	Nurses n = 153	n = ?,? females, Mean age in IG: 82.5 (group 1) and 80.9 (group 2); in CG: 79.2 (group 5) and 82.2 (group 6)	- Rate of falls
Carroll 2012	USA	CRT	Urban hospitals (academic medical centres & community hospitals)	6	Nurses n = ?	Patients admitted during study period n = 364,? female,? age	-Adherence to recommended practice
Dykes 2010						Patients admitted during study period n = 10,264 (5,160 in CDS and 5,104 in CG), 54.5% female, mean 78.8 years (among patients 65 years or older)	-Rate of falls -Rate of fall injuries -Adherence to recommended practice
Clemson 2024	Australia	CRT	Primary care practices	12	Physicians n = 75 Allied health professionals n = 342	Community-dwelling older adults who had had a fall in the past year or were concerned about falling n = 560 (275 in CDS and 285 in CG), 67.9% female, mean 78.6 years	-Rate of falls -Adherence to recommended practice
Dykes 2020	USA	NRCT	Hospital (academic medical centres)	42	Nurses n = ?	Patients admitted during study period n = 37,231 (17,948 in pre-intervention group and 19,283 in post-intervention group), 53.8% female, mean 60.8 years	-Rate of falls -Rate of fall injuries
Elley 2008	New Zealand	IRPGT	Primary care practices	12	Nurses n = 54	Adults who had fallen in the past 12 months n = 312 (155 in CDS and 157 in CG), 68.9% female, mean 80.8 years	-Rate of falls
Ferrer 2014	Spain	IRPGT	Primary care practices	24	Physicians and nurses n = ?	Community-dwelling older adults born in 1924 (85 years of age at study start) n = 328 (164 in CDS and 164 in CG), 61.1% female	-Risk of falling -Time to first, second, and recurrent falls -Hospital admissions
Frankenthal 2014	Israel	IRPGT	Hospital (Chronic care geriatric facility)	12	Pharmacist n = ?	Residents prescribed at least one medication n = 359 (183 in CDS and 176 in CG), 66.6% female, mean 82.7 years	-Rate of falls -Hospital admissions
Gallagher 2011	Ireland	IRPGT	University hospital	6	Physicians n = ?	Patients admitted via the emergency department under care of a GP n = 382 (190 in CDS and 192 in CG), 53.1% female, median 74.5 years (IG) and 77 (CG)	-Medication outcomes -Risk of falling -Hospital admissions
Ganz 2015	USA	NRCT	Primary care practices	24	Physicians, nurse practitioner, physician assistant n = 44	Patients who screened positive for fall risk n = 1,791 (1,187 in CDS and 604 in control), 72% female, mean 82.9 years	-Rate of fall injuries
Wenger 2010				12		Patients who screened positive for falls or fear of falling and UI: n = 1,211 (586 in CDS and 625 in CG), 71.7% female, mean 83 years	-Adherence to recommended practice
Ganz 2022	USA	CRT	Primary care practices	60	Nurses n = ?	Community-living persons at increased risk for serious fall injuries n = 5,451 (2,802 in CDS and 2,649 in CG), 62% female, mean 79.7 years	-Rate of falls -Rate of falls leading to medical attention -Hospital admissions
Groshaus 2012	Canada	NRCT	Acute care hospitals	3	Nurses n = ?	Patients residing on study units n = ?,? female,? years	-Adherence to recommended practice -Risk of falling
Healey 2004	UK	CRT	District general hospital	12	Nurses n = ?	All older adults who received care in the wards during the study period n = 1,654 (905 in CDS and 749 in CG), 60% female, mean 81.3 years	-Rate of falls -Rate of fall injuries
Lightbody 2002	UK	IRPGT	University hospital	6	Nurses n = ?	Older adults discharged from Accident and Emergency Department after a fall n = 348 (171 in CDS and 177 in CG), 74.4% female, median 75 years	-Medication outcomes -Risk of falling -Rate of falls -Hospital admissions
Logan 2021	UK	CRT	Long-term care homes	12	Care home staff n = 3,609	Long-term care home residents in care homes for older adults n = 1,657 (775 in CDS and 882 in CG), 67.9% female, mean 85 years	-Rate of falls -Mortality
Mahoney 2007	USA	IRPGT	Home visits	12	Registered nurse, physical therapist, physician n = ?	Community-dwelling adults with two falls in the past year or one fall in the previous two years with injury or balance problems n = 349 (174 in CDS and 175 in CG), 78.5% female, mean 80 years	-Rate of falls -Hospital admissions
Peterson 2007	USA	IRPGT	Tertiary care hospital	9	Physicians n = 778	Inpatients ≥ receiving care on one of the order entry wards (i.e., emergency room, intensive care units, subacute units) n = 2,981,? female, = years	-Adherence to recommended practice
Phelan 2024	USA	CRT	Primary care practices	18	Physicians	Community-dwelling adults aged ≥ 60 years, prescribed at least 1 medication from any of 5 targeted medication classes (opioids, sedative-hypnotics, skeletal muscle relaxants, tricyclic antidepressants, and first-generation antihistamines for at least 3 consecutive months n = 2,367 (1,106 in CDS and 1,261 in CG), 63% female, mean 70.6	-Time to first medically treated fall (risk of fall injuries) -Medication outcomes
Snooks 2014	UK	CRT	Emergency ambulance services	1	Paramedics n = 42	Community-dwelling older adults living in the catchment area of a participating falls service n = 779 (436 in CDS and 343 in CG), 63.4% female, median 82.5 years	-Adherence to recommended practice -Risk of falling -Mortality -Hospital admissions
Tamblyn 2012	Canada	CRT	Primary care practices	23	GPs n = 81	Patients with a prescription for a psychotropic drug n = 5,628 (2,887 in CDS and 2,741 in CG), 67.1% female, mean 75.2 years	-Medication outcomes
Weber 2008	USA	CRT	Primary care practices	15	Pharmacists and GPs n = ?	Community-dwelling patients at risk for falls n = 620 (413 in CDS and 207 in CG), 79.2% female, mean 76.9 years	-Risk of falling -Medication outcomes
Wenger 2009	USA	NRCT	Primary care practices	32	Physicians n = 40	Community-dwelling patients who had at least one of three geriatric conditions: falls and gait impairment, urinary incontinence, and cognitive impairment n = 644 (357 in CDS and 287 in CG), 66% female, mean 81 years	-Adherence to recommended practice

CBA: Controlled Before-After study; CCDS: Computerised Clinical Decision Support; CDS: Clinical Decision Support group; CG: Control group; CRT: Cluster-Randomised Trial; ED: Emergency Department; GP: General Practitioner; IG: Intervention Group; ITS: Interrupted Time-Series study; IRPGT: Individually-Randomised Parallel-Group Trial; NRCT: Non-Randomised Controlled Trial; UI: Urinary Incontinence.

#### Interventions and CDS tools.

Table 3 presents an overview of the characteristics of the interventions and CDS tools assessed in the included studies. Most interventions (15 studies; 60%) delivered CDS to healthcare practitioners to aid with fall risk assessments, followed by an offer to the patient of specific fall prevention interventions. The majority of interventions (22 studies; 88%) consisted of more than one component delivered as a bundle, i.e., there was a defined order in the delivery of the components, such as conducting a medication review prior to implementing medication changes. Interventions were primarily directed at either one type of healthcare practitioner (12 studies; 48%) or at two or more types of healthcare practitioners within the same healthcare setting (11 studies; 44%). See S5 Table for a detailed description of the interventions. Most CDS tools were designed as algorithms presented as if-then statements or as checklists with risk factors for falls, followed by recommendations for fall prevention interventions that were either generated automatically or chosen manually from a list (68%). Regarding delivery methods, 15 CDS tools (60%) were electronic, three (12%) were delivered both electronically and on paper, two (8%) were paper-based, and for five studies (20%), the delivery method was unclear. Among the electronic CDS tools, nine (36%) included alerts, reminders, or prompts, while the others delivered decision support on demand. Control interventions included usual care (76%), usual care plus a falls information pamphlet (4%), usual care plus an offer of two social visits (4%), home safety visits (4%), paper-based CDS (4%), and no treatment (4%). One study (4%) failed to mention control. See S6 Table for a detailed description of the CDS design factors.

**Table 3:** Characteristics of interventions and Clinical Decision Support.

Study	Type of intervention	Active components	n types of intervention deliverers	Organisational levels	CDS format	CDS features
Aizen 2015	1	High	1: Nurses	Low	Electronic	Risk assessment tool with intervention recommendations based on risk
Barker 2016	1	High	3: Nurse, site clinical leader, champion	Intermediate	–	Checklist with risk factors and recommended interventions; Reminders on the use of intervention components
Bhasin 2020, Ganz 2022	1	High	3: Nurse, primary care physician, pharmacist	Intermediate	Electronic	Algorithm with risk factors and intervention recommendations
Blalock 2020	2	High	2: Pharmacist, primary care physician	Intermediate	Electronic	Adapted STEADI algorithm with risk factors and intervention recommendations
Blum 2021	2	High	3: Pharmacist, hospital physician, general practitioner	High	Electronic	Lists of FRIDs (STOPP/START criteria)
Byrne 2005	3	High	1: Nurse	Low	Electronic	Automatically generated fall risk estimates and intervention recommendations
Carroll 2012, Dykes 2010	1	High	1: Nurse	Low	Electronic and paper-based	Alerts printed on paper to hang over bed
Clemson 2024	1	High	> 7: Primary care physicians, physiotherapists, occupational therapists, nurses, podiatrists, pharmacists, exercise physiologists, and other professions	High	Electronic and paper-based	iSOLVE algorithm; Fall risk assessment checklist; GP fall risk assessment chart; Tailoring interventions to fall risk chart; Risk information automatically sent to GP; Case studies which illustrate the algorithm and tailoring options; Examples of how to talk with patients about falls
Dykes 2020	1	High	1: Nurse	Low	Electronic	Checklist with risk factors and recommended interventions
Elley 2008	1	High	3: Nurse, trained practitioner, physiotherapist	Intermediate	–	Algorithm with risk factors and intervention recommendations
Ferrer 2014	1	High	2: Nurse, primary care physician	Intermediate	–	Algorithm with risk factors and intervention recommendations
Frankenthal 2014	2	High	2: Pharmacist, hospital physician	Intermediate	Electronic	Lists of FRIDs (STOPP/START criteria)
Gallagher 2011	2	High	1: Hospital physician	Low	–	Lists of FRIDs (STOPP/START criteria)
Ganz 2015, Wenger 2010	1	High	3: Primary care physician, nurse, physician assistant	Intermediate	Electronic	Medical record prompts
Groshaus 2012	1	High	1: Nurse	Low	Electronic	Electronic order set
Healey 2004	1	High	1: Nurse	Low	Paper-based	Checklist with risk factors and intervention recommendations
Lightbody 2002	1	High	1: Nurse	Low	–	Checklist with risk factors and intervention recommendations
Logan 2021	1	High	3: Nurse, physiotherapist, occupational therapist	Intermediate	Paper-based	Checklist with risk factors and intervention recommendations
Mahoney 2007	1	High	3: Nurse, physiotherapist, primary care physician	Intermediate	Electronic	Algorithm with automatically generated intervention recommendations
Peterson 2007	4	Low	1: Hospital physician	Low	Electronic	Guided medication dosing; Prompts presented on-screen
Phelan 2024	2	Intermediate	1: Primary care physician	Low	Electronic	Evidence-based pharmaceutical opinions; Deprescribing pearls with conversation starters
Snooks 2014	5	High	1: Paramedic	Low	Electronic	Prompts to start assessment; Algorithm with automatically generated care plan
Tamblyn 2012	4	Low	1: Primary care physician	Low	Electronic	Predictive model to automatically estimate risk of injury; Alerts when patient was prescribed a FRID; Graphics presenting risk estimates; Guided medication dosing
Weber 2008	2	High	3: Pharmacist, geriatrician, primary care physician	Intermediate	Electronic	Guided medication dosing; Alerts with patient’s fall risk
Wenger 2009	1	High	3: Nurse, primary care physician, medical assistant	Intermediate	Electronic and paper-based	Medical record prompts with suggestions for appropriate action

CDS: Clinical Decision Support; FRIDs: Fall-risk-increasing drugs; GP: General practitioner; STEADI: Stopping Elderly Accidents, DEaths, and Injuries; STOPP/START: Screening Tool of Older Person’s Prescriptions and Screening Tool to Alert doctors to Right Treatment.

**Type of intervention:**

1: Fall risk assessment and interventions based on CDS.

2: Medication review and recommendations made to physician.

3: Automatically generated fall risk based on prediction models, followed by recommended interventions.

4: Guided medication dosing using computerised CDS.

5: Computerised CDS presented to paramedics on hand-held tablets.

**Active components (iCAT_SR domain 2):**

High: More than one component and delivered as a bundle (clear order in the delivery of the components) (high level of complexity).

Intermediate: More than one component and delivered as a package (no specific order) (intermediate level of complexity).

Low: One component (low level of complexity).

**Organisational levels and categories targeted by the intervention (iCAT_SR domain 3):**

High: Intervention directed at two or more healthcare settings, e.g., primary care and hospitals (multi-level).

Intermediate: Intervention directed at two or more categories of healthcare practitioners within the same healthcare setting, e.g., nurse and physiotherapist in primary care (multi-category).

Low: Intervention directed at one category of healthcare practitioner, e.g., nurses (single category).

#### Risk of bias in included studies.

A detailed description of RoB assessments is available in S7 Table. We assessed a total of 40 results across five outcomes. Out of 33 results from randomised trials, 19 were judged to have high RoB, 10 had some concerns, and four were rated as having a low RoB. All seven results from non-randomised studies were judged to be at serious RoB, primarily owing to concerns for bias related to confounding.

### Results of individual studies

Results from each individual study are available in S8 Table.

### Healthcare practitioner performance

#### Adherence to recommended practice.

**Fig 2:**
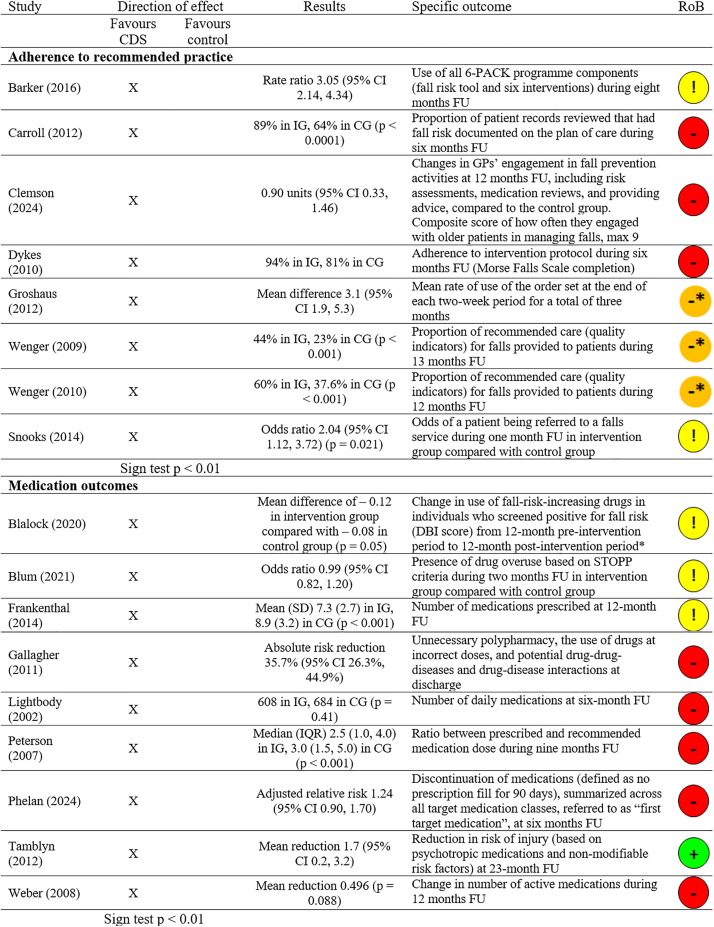
Healthcare practitioner performance results. FU: Follow-up; IQR: Interquartile range; DBI: Drug Burden Index; IG: Intervention group; CG: Control group; RoB: Overall risk of bias judgement. † Based on subgroup analysis and therefore not randomised. * Non-randomised study. Serious risk of bias based on ROBINS-I. Sign test: H0: n results favouring CDS = n results favouring control. Ha: n results favouring CDS ≠ n results favouring control.

Five CRTs [24,25,28,69,86] and three NRCTs [78,80,88] reported results on adherence to recommended practice regarding fall prevention (Fig 2). Outcomes across these studies included fall risk documentation and provision of recommended intervention components. The median follow-up time was 7 months (range: 1–13 months). All eight results favoured the intervention (100%; 95% CI: 68% to 100%; p < 0.01). The certainty of evidence was low due to risk of bias (Table 4). Overall, these findings suggest that CDS may improve healthcare practitioners’ adherence to recommended practice in fall prevention.

**Table 4:** Summary of findings.

**CDS interventions compared with usual care for HCPs in fall prevention among older adults**					
**Population:** HCPs, including nurses, physiotherapists, general practitioners, occupational therapists, nursing assistants, pharmacists, physicians, primary care providers, and paramedics **Settings:** Hospitals, residential care, primary care, and the homes of older adults **Intervention:** CDS targeted at HCPs **Comparison:** Usual care					
**Outcomes** **No of participants (studies)**	**Relative effects (95% CI)**	Anticipated absolute effects* (95% CI)			**Certainty of evidence**
		**In control**	**CDS interventions**	**Difference**	
**Adherence to recommended practice: fall risk assessments and interventions** Follow-up: median 7 (1–13) months ^a^ (8)	^b^	^b^	^b^	^b^	⊕⊕OO Low^c^ Due to very serious risk of bias
**Adherence to recommended medication review and prescribing** Follow-up: median 9 (0–23) months ^a^ (9)	^b^	^b^	^b^	^b^	⊕⊕⊕O Moderate^d^ Due to risk of bias
**Fall risk** Follow-up: median 9 (1–24) months > 13,636^e^ (10)	OR 0.93 (0.85 to 1.01)	287 per 1,000	273 per 1,000 (255–290)	14 fewer fallers per 1,000 (32 fewer to 3 more)	⊕⊕OO Low^f^ Due to risk of bias and imprecision
**Rate of falls**					
In hospitals or residential care Follow-up: median 6 (3–21) months > 50,054^e^ (8)	RaR 0.74 (0.63 to 0.88)	672 per 1,000 person-years	497 per 1,000 person-years (423–591)	175 fewer falls per 1,000 person-years (249 fewer to 81 fewer)	⊕⊕⊕O Moderate^g^ Due to risk of bias
In community-dwelling older adults Follow-up: median 12 (12–24) months 7,000 (5)	RaR 0.97 (0.93 to 1.00)	672 per 1,000 person-years	652 per 1,000 person-years (625–672)	20 fewer falls per 1,000 person-years (47 fewer to 0 fewer)	⊕⊕OO Low^h^ Due to risk of bias and imprecision
In patients with mean age ≥ 80 years Follow-up: median 12 (3–12) months 6,264 (7)	RaR 0.72 (0.61 to 0.86)	672 per 1,000 person-years	484 per 1,000 person-years (410–578)	188 fewer falls per 1,000 person-years (262 fewer to 94 fewer)	⊕⊕⊕O Moderate^i^ Due to risk of bias
In patients with mean age between 65 and 80 years Follow-up: median 12 (6–24) months 84,084 (6)	RaR 0.92 (0.84 to 1.01)	672 per 1,000 person-years	618 per 1,000 person-years (564–679)	54 fewer falls per 1,000 person-years (108 fewer to 7 more)	⊕⊕OO Low^j^ Due to risk of bias and imprecision
**Rate of fall injuries**					
In patients with mean age between 65 and 80 years Follow-up: median 16.5 (6–24) months > 50,979^e^ (4)	RaR 0.80 (0.59 to 1.09)	164 per 1,000 person-years	131 per 1,000 person-years (97–179)	33 fewer fall injuries per 1,000 person-years (67 fewer to 15 more)	⊕⊕OO Low^k^ Due to risk of bias and imprecision
In patients with mean age ≥ 80 years Follow-up: median 9 (6–12) months 3,445 (2)	RaR 1.29 (0.99 to 1.69)	164 per 1,000 person-years	212 per 1,000 person-years (162–277)	48 more fall injuries per 1,000 person-years (2 fewer to 113 more)	⊕OOO Very low^l^ Due to very serious risk of bias and imprecision

*Assuming a control group fall risk of 28.7%, a fall rate of 672 falls per 1,000 person-years, and a fall injury rate of 164 fall injuries per 1,000 person-years, based on data from Bergen et al. [62]. The risk in the intervention group is based on the assumed risk in the comparison group and the relative effect of the intervention (and its 95% CI).

CDS: clinical decision support; CI: confidence interval; HCPs: healthcare practitioners; OR: odds ratio; RaR: rate ratio.

**Explanations:**

^a^The numbers of participating healthcare practitioners were not reported.

^b^Not estimable due to the use of different and incompletely reported effect measures across studies.

^c^Very serious risk of bias due to confounding, deviations from intended interventions, and selection of reported results.

^d^Risk of bias due to deviations from intended interventions.

^e^The number of participants were not reported in all studies.

^f^Risk of bias due to the randomisation process, deviations from intended interventions, and measurement of the outcome. Imprecision due to the upper bound of the 95% confidence interval crossing the null effect.

^g^Risk of bias in the randomisation process and due to deviations from intended interventions.

^h^Risk of bias due to deviations from intended interventions. Imprecision due to the upper bound of the 95% confidence interval including the null effect.

^i^Risk of bias due to deviations from intended interventions and selection of reported results.

^j^Risk of bias due to the randomisation process, confounding, and deviations from intended interventions. Imprecision due to the upper bound of the 95% confidence interval crossing the null effect.

^k^Risk of bias due to confounding and deviations from intended interventions. Imprecision due to the upper bound of the 95% confidence interval crossing the null effect.

^l^Very serious risk of bias due to the randomisation process and confounding. Imprecision due to the lower bound of the 95% confidence interval crossing the null effect.

#### Adherence to recommended medication review and prescribing.

Five CRTs [23,39,70,72,87] and four IRPGTs [75,76,82,85] reported indicators of medication outcomes (Fig 2). The common outcome across these studies was the reviewing and prescribing of drugs that may increase fall risk. The median follow-up time was 9 months (range: 0–23 months). All nine results favoured the intervention (100%; 95% CI: 70% to 100%; p < 0.01). The certainty of evidence was moderate due to risk of bias. These findings suggest that CDS likely improves medication reviewing and prescribing outcomes in the context of fall prevention.

### Patient outcomes

#### Fall risk.

**Fig 3:**
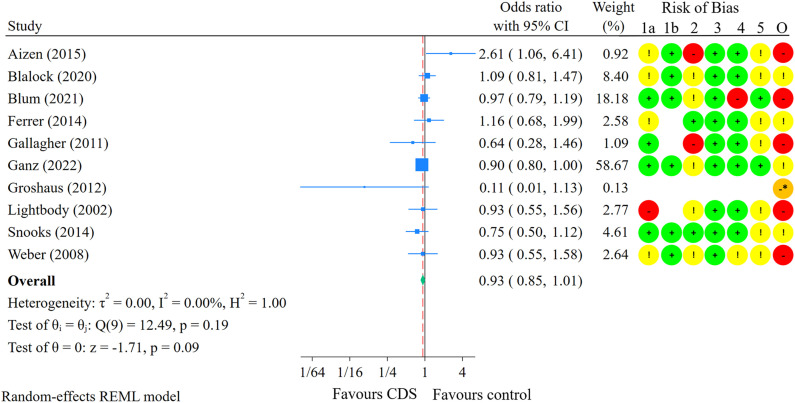
Meta-analysis comparing CDS interventions with control on fall risk. Red dashed line shows the point estimate for the meta-analysis overall. **Risk of Bias legend:** Circles: Green: Low risk of bias (RoB); Yellow: Some concerns for RoB; Red: High RoB; Orange: Serious RoB. 1a: Bias arising from the randomisation process. 1b: Bias arising from the timing of identification or recruitment of individual participants within clusters (for cluster-randomised only). 2: Bias due to deviations from intended interventions. 3: Bias due to missing outcome data. 4: Bias in measurement of the outcome. 5: Bias in selection of the reported result. O: Overall RoB judgement. * Non-randomised study. ROBINS-I was used to assess RoB.

Nineteen of the included studies reported outcome data on fall risk (proportion of participants who fell) and/or fall rate (number of falls per x person-years of follow-up), enabling meta-analyses of these outcomes. Ten studies were included in the meta-analysis on fall risk, with a median follow-up time of 9 months (range: 1–24 months) [23,27,72,74,76,79,80,82,86,87] (Fig 3). Of these studies, six were conducted in patients aged 65–80 years, three in patients aged 80 years or older, and one study did not report the age of patients. Furthermore, five studies were conducted in hospitals or residential care settings, while the other five focused on community-dwelling older adults. The overall estimated effect of CDS on fall risk was an odds ratio of 0.93 (95% CI: 0.85 to 1.01). Assuming a baseline fall risk of 28.7% [62], this 7% reduction in the odds of falling results in 14 fewer fallers per 1,000 older adults (95% CI: 32 fewer to 3 more) receiving the intervention compared with controls, during nine months of follow-up (Table 4). Heterogeneity was unimportant (I^2^ = 0%; p = 0.19). Visual inspection of the funnel plot showed no clear sign of small-study effects (S1 Fig). The Egger test suggested no evidence of small-study effects (p = 0.89). Additionally, the trim-and-fill analysis imputed no studies, and therefore made no difference in the effect estimate (S2 Fig). The certainty of the evidence was low due to risk of bias and imprecision. While CDS aimed at healthcare practitioners may influence fall risk, the evidence does not demonstrate a statistically significant effect, and uncertainty remains regarding its effectiveness.

#### Rate of falls.

**Fig 4:**
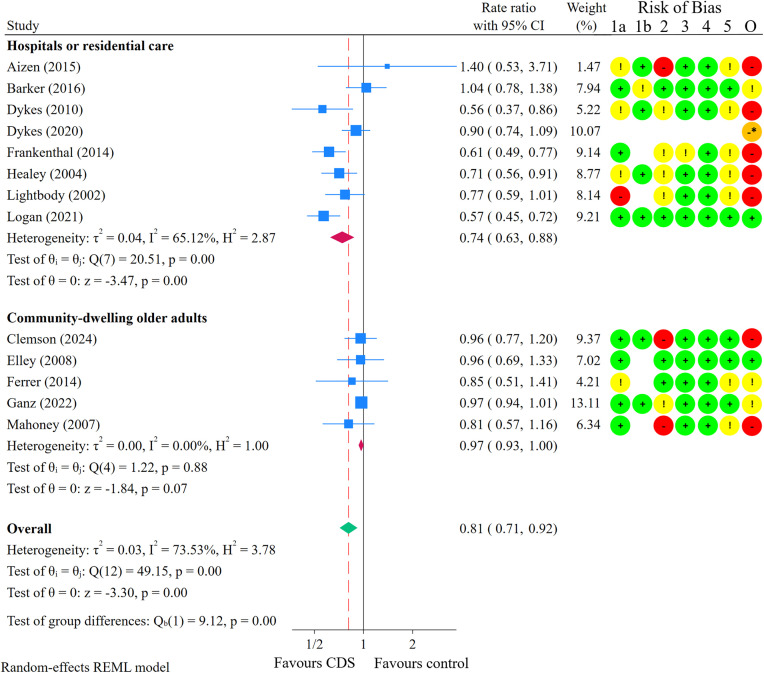
Meta-analysis comparing CDS interventions with control on fall rate, subgroup analysis by study setting. Red dashed line shows the point estimate for the meta-analysis overall. **Risk of Bias legend:** Circles: Green: Low risk of bias (RoB); Yellow: Some concerns for RoB; Red: High RoB; Orange: Serious RoB. 1a: Bias arising from the randomisation process. 1b: Bias arising from the timing of identification or recruitment of individual participants within clusters (for cluster-randomised only). 2: Bias due to deviations from intended interventions. 3: Bias due to missing outcome data. 4: Bias in measurement of the outcome. 5: Bias in selection of the reported result. O: Overall RoB judgement. * Non-randomised study. ROBINS-I was used to assess RoB.

Thirteen studies were included in the meta-analysis on fall rate [24,26–28,38,69,74,75,79,81–84] (Fig 4). Substantial heterogeneity was present in the effect estimate (I^2^ = 74%; p < 0.01). Subgroup analyses revealed statistically significant differences in fall rates when grouped by study setting (p < 0.01) and patients’ age (p < 0.01). The results are therefore presented separately by study setting and patients’ age.

Regarding subgroup analysis by study setting, the median follow-up time was 6 months (range: 3–21 months) for studies conducted in hospitals or residential care, and 12 months (range: 12–24 months) for studies on community-dwelling older adults. Among the eight studies conducted in hospitals or residential care, four included patients aged 80 years or older, and four included patients aged 65–80 years. Similarly, among the five studies focusing on community-dwelling older adults, three included patients aged 80 years or older, while two included patients aged 65–80 years. In hospitals or residential care, the overall estimated effect of CDS on fall rate was a rate ratio of 0.74 (95% CI: 0.63 to 0.88; I^2^ = 65%; p < 0.01). Assuming a baseline fall rate of 672 falls per 1,000 person-years [62], this 26% reduction in fall rate corresponds to 175 fewer falls per 1,000 person-years (95% CI: 249 fewer to 81 fewer) (Table 4). The certainty of the evidence was moderate due to risk of bias. These findings suggest that CDS aimed at healthcare practitioners likely reduces the rate of falls in hospitals and residential care settings.

For community-dwelling older adults, the overall estimated effect on fall rate was a rate ratio of 0.97 (95% CI: 0.93 to 1.00; I^2^ = 0%; p = 0.88). The certainty of the evidence was low due to risk of bias and imprecision. While CDS may influence fall rates in community-dwelling older adults, the effect is not statistically significant, and the certainty of the evidence is low.

Regarding the subgroup analysis by patients’ age, the median follow-up time was 12 months (range: 3–12 months) for studies involving patients with a mean age ≥ 80 years, and 12 months (range: 6–24 months) for studies of patients with a mean age between 65 and 80 years (S3 Fig). Among the six studies including patients aged 65–80 years, four were conducted in hospitals or residential care settings, while two focused on community-dwelling older adults. Similarly, among the seven studies involving patients aged 80 years or older, four were conducted in hospitals or residential care, while three focused on community-dwelling older adults. The overall estimated effect of CDS on fall rate among patients with a mean age of ≥ 80 years was a rate ratio of 0.72 (95% CI: 0.61 to 0.86; I^2^ = 46%; p = 0.09). Assuming a baseline fall rate of 672 falls per 1,000 person-years [62], this 28% reduction in fall rate corresponds to 188 fewer falls per 1,000 person-years (95% CI: 262 fewer to 94 fewer). The certainty of the evidence was moderate due to risk of bias. These findings suggest that CDS likely reduces the rate of falls among patients with a mean age of ≥ 80 years.

For patients with a mean age between 65 and 80 years, the overall estimated effect on fall rate was a rate ratio of 0.92 (95% CI: 0.84 to 1.01; I^2^ = 31%; p = 0.09). The certainty of the evidence was low due to risk of bias and imprecision. While CDS may influence fall rates in patients aged 65–80 years, the effect is not statistically significant, and the certainty of the evidence is low. Visual inspection of the funnel plot showed no clear sign of small-study effects (S4 Fig). The Egger test suggested no evidence of small-study effects (p = 0.99). Additionally, the trim-and-fill analysis imputed no studies and therefore made no difference in the effect estimate (S5 Fig). There was no statistically significant subgroup difference in fall rates when stratified by RoB (p = 0.47) (S6 Fig).

#### Rate of fall injuries.

**Fig 5:**
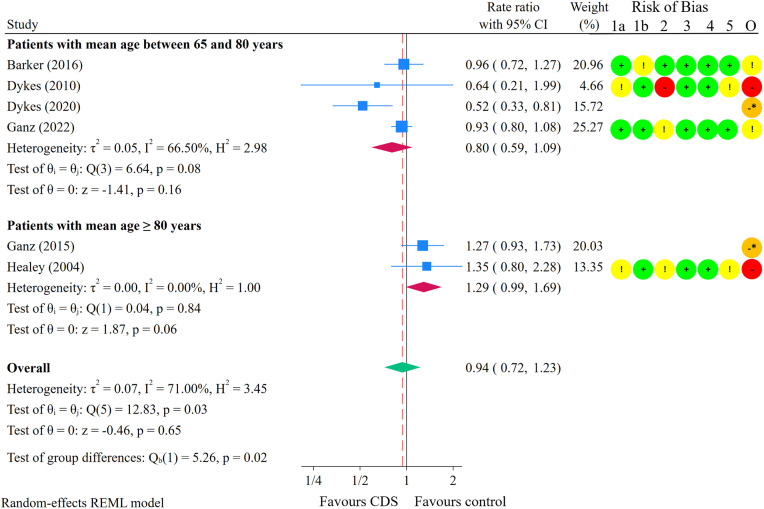
Meta-analysis comparing CDS interventions with control on fall injury rate, subgroup analysis by patients’ age. Red dashed line shows the point estimate for the meta-analysis overall. Circles: Green: Low risk of bias (RoB); Yellow: Some concerns for RoB; Red: High RoB; Orange: Serious RoB. 1a: Bias arising from the randomisation process. 1b: Bias arising from the timing of identification or recruitment of individual participants within clusters (for cluster-randomised only). 2: Bias due to deviations from intended interventions. 3: Bias due to missing outcome data. 4: Bias in measurement of the outcome. 5: Bias in selection of the reported result. O: Overall RoB judgement. * Non-randomised study. ROBINS-I was used to assess RoB.

Six studies were included in the meta-analysis on fall injury rate [24,26,28,77,79,81] (Fig 5). Substantial heterogeneity was present in the effect estimate (I^2^ = 71%, p = 0.03). Subgroup analyses revealed a statistically significant difference in fall injury rate between patients with a mean age of 65–80 years and patients with a mean age of 80 years or older (p = 0.02). The results are therefore presented separately by patients’ age. The median follow-up time was 16.5 months (range: 6–24 months) for studies including patients with a mean age of 65–80 years and 9 months (range: 6–12 months) for studies involving patients with a mean age of 80 years or older. The overall estimated effect of CDS in preventing fall injuries among patients aged 65–80 years was a rate ratio of 0.80 (95% CI: 0.59 to 1.09; I^2^ = 67%; p = 0.08), indicating a statistically non-significant result as the confidence interval crosses the null effect. Assuming a baseline fall injury rate of 164 fall injuries per 1,000 person-years [62], this 20% reduction in fall injury rate corresponds to 33 fewer fall injuries per 1,000 person-years (95% CI: 67 fewer to 15 more) in older adults receiving the intervention compared to controls (Table 4). The certainty of the evidence was low due to risk of bias and imprecision. While CDS may influence fall injury rates in patients aged 65–80 years, the effect is not statistically significant.

For patients with a mean age of 80 years or older, the overall estimated effect on fall injury rate was a rate ratio of 1.29 (95% CI: 0.99 to 1.69; I^2^ = 0%; p = 0.84). The certainty of the evidence was very low due to risk of bias and imprecision. We are very uncertain about the effect of CDS on fall injury rates in patients aged 80 years or older. There was no statistically significant subgroup difference in fall injury rates when stratified by RoB (p = 0.93) or study setting (p = 0.41). See S7 and S8 Figs for subgroup analyses on fall injury rate.

### Mortality and hospitalisations

Five CRTs [70–72,84,86] reported on mortality, while five IRPGTs [74–76,82,83] and three CRTs [71,72,86] reported on hospital admissions. The sample sizes were small, the outcomes were rare, and no consistent patterns were observed in the direction of effect for either mortality or hospital admissions. For further details on individual study results, see S8 Table.

### Certainty of the evidence

The certainty of the evidence was assessed for nine outcomes, with the results summarised in the GRADE evidence profile in S9 Table and Table 4.

## Discussion

This systematic review summarised 25 studies (28 publications) investigating healthcare practitioners’ use of CDS in fall prevention and its effects on healthcare practitioner performance and patient outcomes. The interventions were delivered by nurses, physicians, physiotherapists, pharmacists, occupational therapists, and paramedics. Regarding healthcare practitioner performance, CDS may improve fall risk assessments and the provision of recommended interventions and likely improves medication review and prescribing. Regarding patient outcomes, CDS likely decreases the rate of falls in hospitals and residential care settings. CDS also likely reduces falls in patients aged 80 years or older. Furthermore, CDS may reduce fall rates in community-dwelling older adults. It also appears to reduce fall rates in patients aged 65–80 years; however, the effects in these subgroups may be small. CDS interventions likely reduce fall risk slightly and may reduce the rate of fall injuries in patients aged 65–80 years. Finally, while CDS may reduce fall injuries in adults aged 65–80 years, the effect on fall injuries in adults aged 80 years or older remains very uncertain.

### Interventions

A common feature of all experimental interventions included in this review is that they delivered decision support to healthcare practitioners aiming to prevent falls in older adults. The control interventions were generally described only as consisting of usual care, with limited detail about the specific interventions, which varied depending on the setting and type of healthcare practitioner. Most experimental interventions utilised CDS to assist healthcare practitioners in conducting risk assessments and implementing preventive measures across multiple domains, such as gait and balance problems, environmental factors, and medications, rather than focusing on a single domain. Multifactorial fall prevention interventions have been shown effective in reducing falls [58], and the use of CDS to deliver such complex interventions may offer healthcare practitioners structure and guidance [89]. However, it is worth noting that, while most interventions provided CDS electronically, only 36% of the CDS tools automatically delivered recommendations to the healthcare practitioners. A systematic review previously found that automatically providing CDS recommendations, as opposed to requiring practitioners to access them on demand, may lead to large improvements in adherence to clinical guidelines [21].

### Healthcare practitioner performance

Our findings align with those of previous systematic reviews, which have reported improvements in adherence to recommended practice [30,32] and medication outcomes [31]. Mebrahtu et al. [29] also found favourable effects on healthcare practitioner performance, including improvements in nurses’ adherence to hand disinfection guidance, insulin dosing, timely blood sampling, and documentation of care. Similarly, Kwan et al. [32] reported that CDS increased the proportion of patients receiving recommended care. Additionally, Yourman et al. [31] found that CDS improved medication outcomes in most cases. Although improvements were mostly moderate, these results demonstrate the diversity of clinical areas in which CDS may be beneficial.

### Patient outcomes

Our meta-analyses suggested a possible reduction in fall risk with CDS, corresponding to an estimated range of 32 fewer to three more fallers per 1,000 older adults. They also indicated that CDS may reduce fall rates, with a reduction ranging from 20 to 188 fewer falls per 1,000 person-years. The studies included in the meta-analysis on fall risk represent a range of age groups and care settings, suggesting that the findings are broadly applicable to older adults at risk of falls. Several factors may explain the observed differences in the effects of CDS on fall risk versus fall rate. Dautzenberg et al. [11] proposed that the fall outcome may be more accurately measured using fall rate rather than fall risk. The ‘fall risk’ outcome counts the number of individuals who experience at least one fall, regardless of whether they fall multiple times, with each person contributing only one event. In contrast, the ‘rate of falls’ outcome captures each individual fall as a separate event. For example, a person who falls five times during the follow-up period would contribute five events to the ‘rate of falls’ outcome but only one event to the ‘fall risk’ outcome. If an intervention successfully prevents two of these five falls, it would lead to a reduced fall rate but not a reduced fall risk, as the individual would still be classified as having fallen. Consequently, the ‘fall risk’ outcome does not capture changes in the frequency of falls among older adults. It is possible that the interventions studied were more effective at reducing the frequency of falls among individuals with the highest fall risk, i.e., recurrent fallers, than among those who experienced only a single fall. A reduction in the frequency of falls among recurrent fallers would result in a greater reduction in fall rate compared to fall risk. In agreement with our findings, earlier systematic reviews [11,90] reported that multifactorial interventions were associated with a reduction in fall rate but had a smaller impact on fall risk.

The studies included in the meta-analyses on fall rate encompass a range of age groups and care settings, suggesting that the findings are broadly applicable to the entire study population. Several factors may explain the differences in fall rate reduction between settings, i.e., a 26% reduction in hospitals and residential care versus a 3% reduction in community-dwelling older adults. Older adults in hospitals and care homes are at high risk of falls [14], and interventions may be more effective in high-risk populations than in those at lower risk [91,92]. Even if the relative effects were similar, interventions directed at high-risk populations tend to show larger absolute effects than those directed at low- or moderate-risk populations. However, it is important to consider whether the relative effect itself varies across populations with different baseline risk levels, as this could further influence intervention outcomes. Furthermore, CDS tools may be more readily implemented in hospital and residential care settings than in primary care due to the greater complexity of these settings [93]. This may increase healthcare practitioners’ fidelity to intervention protocols, thereby increasing intervention effectiveness. Another contributing factor could be the differences in follow-up time across the included studies. The median follow-up time was six months for studies conducted in hospitals and residential care, compared to 12 months for studies involving community-dwelling older adults. The effectiveness of fall prevention interventions may dwindle as time passes [94], potentially because of patients discontinuing their engagement in fall prevention programmes, such as exercise regimens, after the study period [14].To reduce the risk of type I errors and avoid false positive results, we chose not to conduct a subgroup analysis by follow-up time. Moreover, while our results indicate a 26% reduction in fall rate in hospitals and residential care, the substantial heterogeneity (I^2^ = 65%) suggests that the observed effects likely vary across studies. This variability may be attributed to differences in study populations, settings, or intervention delivery. Despite this heterogeneity, CDS interventions targeting healthcare practitioners likely reduce fall rates in hospitals and residential care settings, although the effect size may depend on the context.

The certainty of the effects on fall injuries is low for patients aged 65–80 years and very low for patients aged 80 years or older, due to risk of bias and imprecision. The wide CIs for fall injury rates may be explained by the fact that fall injuries are relatively rare compared to falls [62]. Importantly, while the estimated effect of CDS on fall injuries among patients aged 80 years or older was a rate ratio of 1.29, the certainty of the evidence is very low. Notably, no studies outside the subgroup analysis on fall injury rate in this age group reported any negative effects attributed to the CDS interventions. Our findings are consistent with those of previous systematic reviews [29,31]. For instance, Yourman et al. found that while CDS may occasionally result in medication prescribing errors, it generally helps to reduce side-effects and improve patient safety [31].

When implementing innovations into clinical practice, both high-income countries (HIC) and low- and middle-income countries (LMIC) face barriers related to political, social, and cultural factors, as well as resource limitations and healthcare practitioner-related factors [95,96]. While the studies included in this review were conducted in HIC, LMIC face additional barriers, such as physical challenges like unreliable power supplies and poor internet connectivity, limited access to electronic health records and computers, and human resources constraints, including overburdened staff and insufficient formal training for healthcare workers [96]. These limitations in digital infrastructure could hinder the integration of CDS tools, which often rely on access to electronic health records and other digital systems [89]. To address these challenges, CDS systems need to be adaptable to LMIC contexts by incorporating offline functionality or offering paper-based versions. Additionally, CDS tools should be user-friendly to minimise the workload on already overburdened staff.

A notable aspect of this study is the absence of established MID values for fall risk, fall rate, and fall injury rate. We assessed certainty based on whether the true effect lies on the observed side of the null, using the null effect as the threshold. While some of the findings in this review suggest that the effects of CDS may be small, we did not determine what constitutes a minimal clinically important effect, as this often depends on the context and setting.

In HIC with robust healthcare infrastructure, even small reductions in fall risk or fall injuries may justify the use of CDS interventions. Conversely, in LMIC contexts, where resources such as computers, internet access, and trained personnel are limited, the threshold for what constitutes an important difference may be higher. In these settings, interventions with larger impacts may be prioritised to promote the best use of limited resources. Establishing context-specific thresholds for clinical importance could enable a more nuanced evaluation and better inform decisions on whether the implementation of CDS interventions is justified.

### Implications

Given that many of the included studies targeted participants with an increased fall risk, such as previous fallers or those with specific risk factors, the findings of this review are likely most applicable to populations at higher risk of falls. Our estimates indicate that, on average, CDS interventions directed at healthcare practitioners may prevent 14 older adults per 1,000 from falling, assuming a baseline fall risk of 28.7%. Additionally, CDS interventions may prevent 175 falls per 1,000 person-years in hospitals and residential care, as well as 188 falls per 1,000 person-years in adults aged 80 years or older, assuming a baseline fall rate of 672 falls per 1,000 person-years. However, further research is needed to better understand the significant differences in fall rate reduction between hospitals or residential care settings and community-dwelling older adults. Future studies should aim to identify the factors that contribute to the greater effectiveness of interventions in hospitals and residential care and explore ways to adapt these strategies for community-dwelling older adults.

The findings of this review have important implications for the clinical implementation of CDS in different care settings. In hospital and residential care settings, where older adults often have a higher risk of falls and healthcare practitioners operate in more structured environments, CDS may be more readily implemented. The fall rate reductions observed in these settings suggest that CDS can support healthcare practitioners in conducting comprehensive fall risk assessments and delivering multifactorial interventions. In contrast, the implementation of CDS in community-based settings may face additional challenges, as these contexts often involve delivering services within older adults’ homes, which typically have less structure and access to digital infrastructure. These differences highlight the need to tailor CDS tools to the specific demands of each setting. For instance, in community-based settings, CDS tools that fit into existing workflows and provide automated decision-support could encourage greater adoption and adherence. Future research should investigate setting-specific determinants for the successful implementation of CDS, with a view to ensuring that tools are adaptable across diverse clinical environments.

This systematic review provides valuable insight to guide the implementation of CDS tools in fall prevention efforts among older adults. Directing interventions at high-risk populations may offer greater benefits than interventions aimed at low- or moderate-risk individuals. Additionally, CDS tools should be designed to automatically deliver on-screen decision support rather than relying on paper-based or on-demand systems [21]. Notably, only 36% of the CDS interventions included in this review met this criterion, highlighting an opportunity to further improve patient outcomes, adherence to recommended practice, and medication outcomes.

### Strengths and limitations

To our knowledge, this is the first systematic review to provide a comprehensive overview of the effects of CDS used by healthcare practitioners in fall prevention among older adults. A major strength of this review is the use of a thorough and sensitive literature search strategy. Potential non-reporting biases, poor indexing, and other factors make it impossible to know whether all relevant studies were in fact identified [41]. We therefore searched the reference lists of included articles and relevant systematic reviews. Additionally, the funnel plots, along with Egger tests and trim-and-fill analyses, showed no evidence of publication bias for fall risk or fall rate outcomes. This indicates that we have likely included most relevant studies. Furthermore, outcomes related to healthcare practitioner performance were reported so varyingly among the studies that meta-analyses were not possible. We consider it a strength that we conducted vote-counting based on the direction of effect, rather than relying solely on textual descriptions of results [41]. Limitations of vote-counting include that the method provides no information on the magnitude of effects and does not account for differences in the relative sizes of the studies. However, vote-counting enables a statistical analysis of whether there is evidence of an intervention effect. Also, this method may be preferable to a narrative description, in which some results are privileged above others without appropriate justification [41]. Moreover, we performed meta-analyses on fall outcomes and applied the GRADE approach, making explicit judgements about the certainty of the evidence [97].

A limitation of this review is that verification of the collected data by a second reviewer was performed for only five of 25 included studies, raising concerns about potential errors during data collection. However, the selection of each result from each study was discussed and verified with the project statistician to ensure accuracy and consistency. Another limitation is the uncertainty surrounding the specific effects of different CDS tools. For example, while some tools delivered decision support to healthcare practitioners conducting multifactorial risk assessments and interventions [38,84], others focused exclusively on guided medication dosing [39,85]. This variation in tool types and use cases makes it difficult to evaluate the specific effects of different tools or to determine whether certain tools have any measurable impact at all. Furthermore, a limitation of the studies included in this review is that most results were judged as having a high or serious risk of bias. All results from the included non-randomised studies were judged to be at risk of bias due to confounding. The lack of randomisation increases the risk of unequal distribution of confounding factors between intervention groups, as group assignment may be influenced by knowledge of prognostic factors. Moreover, none of the included non-randomised studies employed analysis methods that adequately controlled for all important confounders, such as age, history of falls, sex, or gait and balance impairments. The direction and magnitude of this confounding remain unknown, making it difficult to determine whether the reported effect estimates are greater or lower than the true effect. For the randomised studies, the main concerns for risk of bias were related to deviations from intended interventions, such as crossover effects observed in Peterson et al. [85] and intervention contamination reported in Clemson et al. [69].

## Conclusions

CDS likely improves the performance of healthcare practitioners in fall prevention for certain groups of older adults and in specific care settings. While CDS probably reduces falls and may lower fall injury rates, its effects appear to vary across different subgroups of older adults and care settings. This systematic review provides valuable insights into the role of CDS in supporting healthcare practitioners in fall prevention efforts among older adults. Prioritising resources and targeting interventions toward high-risk patients may yield the greatest impact in reducing falls. To maximise effectiveness, interventions should be sustained over time, and CDS tools should be designed to support improved adherence to recommended practice. Future research on fall injuries should aim to improve precision by increasing the number of participants and extending follow-up periods. In addition, meta-analyses of healthcare practitioner performance outcomes would become possible with standardised and detailed reporting of results. Finally, while the design of CDS tools may positively or negatively affect healthcare practitioners’ adherence to recommended practice, further research is needed to identify the specific design elements that contribute to successful outcomes in fall prevention.

## Supporting information

S1 Table PRISMA checklist. (DOCX)

S2 Table Differences between protocol and review. (DOCX)

S3 Table Electronic searches. (DOCX)

S4 Table Excluded studies. (XLSX)

S5 Table Description of interventions and funding sources. (DOCX)

S6 Table Design factors of CDS. (DOCX)

S7 Table Risk of bias. (PDF)

S8 Table Individual study results. (DOCX)

S9 Table GRADE evidence profile. (DOCX)

S1 Appendix Data used for analyses of healthcare practitioner performance outcomes. (XLSX)

S2 Appendix Data used for meta-analyses. (XLSX)

S3 Appendix Extracted data. (CSV)

S1 Fig Funnell plot of comparison: CDS interventions vs control on fall risk. (PNG)

S2 Fig Trim-and-fill analysis of comparison: CDS interventions vs control on fall risk. (PNG)

S3 Fig Meta-analysis comparing CDS interventions with control on fall rate, subgroup analysis by patients’ age. (PNG)

S4 Fig Funnel plot of comparison: CDS interventions vs control on fall rate. (PNG)

S5 Fig Trim-and-fill analysis of comparison: CDS interventions vs control on fall rate. (PNG)

S6 Fig Meta-analysis comparing CDS interventions with control on fall rate, subgroup analysis by risk of bias. (PNG)

S7 Fig Meta-analysis comparing CDS interventions with control on fall injury rate, subgroup analysis by risk of bias. (PNG)

S8 Fig Meta-analysis comparing CDS interventions with control on fall injury rate, subgroup analysis by study setting. (PNG)

## References

[1] Elias Filho J, Borel WP, Diz JBM, Barbosa AWC, Britto RR, Felício DC. Prevalence of falls and associated factors in community-dwelling older Brazilians: a systematic review and meta-analysis. Cad Saude Publica. 2019;35(8):e00115718. doi: 10.1590/0102-311X00115718 31483046

[2] James SL, Lucchesi LR, Bisignano C, Castle CD, Dingels ZV, Fox JT, et al. The global burden of falls: global, regional and national estimates of morbidity and mortality from the Global Burden of Disease Study 2017. Inj Prev. 2020;26(Supp 1):i3–11. doi: 10.1136/injuryprev-2019-043286 31941758 PMC7571347

[3] Haagsma JA, Olij BF, Majdan M, van Beeck EF, Vos T, Castle CD, et al. Falls in older aged adults in 22 European countries: incidence, mortality and burden of disease from 1990 to 2017. Inj Prev. 2020;26(Supp 1):i67–74. doi: 10.1136/injuryprev-2019-043347 32111726 PMC7571349

[4] Davis JC, Robertson MC, Ashe MC, Liu-Ambrose T, Khan KM, Marra CA. International comparison of cost of falls in older adults living in the community: a systematic review. Osteoporos Int. 2010;21(8):1295–306. doi: 10.1007/s00198-009-1162-0 20195846

[5] Florence CS, Bergen G, Atherly A, Burns E, Stevens J, Drake C. Medical costs of fatal and nonfatal falls in older adults. J Am Geriatr Soc. 2018;66(4):693–8. doi: 10.1111/jgs.15304 29512120 PMC6089380

[6] Heinrich S, Rapp K, Rissmann U, Becker C, König H-H. Cost of falls in old age: a systematic review. Osteoporos Int. 2010;21(6):891–902. doi: 10.1007/s00198-009-1100-1 19924496

[7] Li Y, Hou L, Zhao H, Xie R, Yi Y, Ding X. Risk factors for falls among community-dwelling older adults: a systematic review and meta-analysis. Front Med (Lausanne). 2023;9:1019094. doi: 10.3389/fmed.2022.1019094 36687461 PMC9853191

[8] Shao L, Shi Y, Xie X-Y, Wang Z, Wang Z-A, Zhang J-E. Incidence and risk factors of falls among older people in nursing homes: systematic review and meta-analysis. J Am Med Dir Assoc. 2023;24(11):1708–17. doi: 10.1016/j.jamda.2023.06.002 37433427

[9] Gillespie LD, Robertson MC, Gillespie WJ, Sherrington C, Gates S, Clemson LM, et al. Interventions for preventing falls in older people living in the community. Cochrane Database Syst Rev. 2012;2012(9):CD007146. doi: 10.1002/14651858.CD007146.pub3 22972103 PMC8095069

[10] Olij BF, Ophuis RH, Polinder S, van Beeck EF, Burdorf A, Panneman MJM, et al. Economic evaluations of falls prevention programs for older adults: a systematic review. J Am Geriatr Soc. 2018;66(11):2197–204. doi: 10.1111/jgs.15578 30325013

[11] Dautzenberg L, Beglinger S, Tsokani S, Zevgiti S, Raijmann RCMA, Rodondi N, et al. Interventions for preventing falls and fall-related fractures in community-dwelling older adults: a systematic review and network meta-analysis. J Am Geriatr Soc. 2021;69(10):2973–84. doi: 10.1111/jgs.17375 34318929 PMC8518387

[12] Meekes WM, Korevaar JC, Leemrijse CJ, van de Goor IA. Practical and validated tool to assess falls risk in the primary care setting: a systematic review. BMJ Open. 2021;11(9):e045431. doi: 10.1136/bmjopen-2020-045431 34588228 PMC8483054

[13] Stevens JA, Ballesteros MF, Mack KA, Rudd RA, DeCaro E, Adler G. Gender differences in seeking care for falls in the aged Medicare population. Am J Prev Med. 2012;43(1):59–62. doi: 10.1016/j.amepre.2012.03.008 22704747

[14] Montero-Odasso M, van der Velde N, Martin FC, Petrovic M, Tan MP, Ryg J, et al. World guidelines for falls prevention and management for older adults: a global initiative. Age Ageing. 2022;51(9):afac205. doi: 10.1093/ageing/afac205 36178003 PMC9523684

[15] Markle-Reid MF, Dykeman CS, Reimer HD, Boratto LJ, Goodall CE, McGugan JL. Engaging community organizations in falls prevention for older adults: moving from research to action. Can J Public Health. 2015;106(4):e189-96. doi: 10.17269/cjph.106.4776 26285189 PMC6972171

[16] Olij BF, Erasmus V, Kuiper JI, van Zoest F, van Beeck EF, Polinder S. Falls prevention activities among community-dwelling elderly in the Netherlands: a Delphi study. Injury. 2017;48(9):2017–21. doi: 10.1016/j.injury.2017.06.022 28684078

[17] Leysens G, Vlaeyen E, Vanaken D, Janssens E, Dejaeger E, Cambier D, et al. Het toepassen van valpreventiemaatregelen bij thuiswonende ouderen: een survey onderzoek in Vlaanderen [The use of fall prevention strategies in home care: a survey in Flanders]. Tijdschr Gerontol Geriatr. 2017;48:121–33. doi: 10.1007/s12439-017-0215-7 28466244

[18] Moreland B, Kakara R, Henry A. Trends in nonfatal falls and fall-related injuries among adults aged ≥65 years - United States, 2012-2018. MMWR Morb Mortal Wkly Rep. 2020;69(27):875–81. doi: 10.15585/mmwr.mm6927a5 32644982 PMC7732363

[19] Shaver AL, Clark CM, Hejna M, Feuerstein S, Wahler RG Jr, Jacobs DM. Trends in fall-related mortality and fall risk increasing drugs among older individuals in the United States,1999-2017. Pharmacoepidemiol Drug Saf. 2021;30(8):1049–56. doi: 10.1002/pds.5201 33534172 PMC8254780

[20] Fretheim A, Flottorp S, Oxman A. Effekt av tiltak for implementering av kliniske retningslinjer. Rapport fra Kunnskapssenteret nr. 10-2015. Oversikt over systematiske oversikter [Effect of interventions for implementing clinical practice guidelines]. Oslo; 2015. 28510383

[21] Van de Velde S, Heselmans A, Delvaux N, Brandt L, Marco-Ruiz L, Spitaels D, et al. A systematic review of trials evaluating success factors of interventions with computerised clinical decision support. Implement Sci. 2018;13(1):114. doi: 10.1186/s13012-018-0790-1 30126421 PMC6102833

[22] Wasylewicz ATM, Scheepers-Hoeks AMJW. Clinical Decision Support Systems. In: Kubben P, Dumontier M, Dekker A, editors. Fundamentals of Clinical Data Science. Cham (CH): Springer. 2018. p. 153–69. doi: 10.1007/978-3-319-99713-1_11 31314237

[23] Blalock SJ, Ferreri SP, Renfro CP, Robinson JM, Farley JF, Ray N, et al. Impact of STEADI-Rx: a community pharmacy-based fall prevention intervention. J Am Geriatr Soc. 2020;68(8):1778–86. doi: 10.1111/jgs.16459 32315461

[24] Dykes PC, Carroll DL, Hurley A, Lipsitz S, Benoit A, Chang F, et al. Fall prevention in acute care hospitals: a randomized trial. JAMA. 2010;304(17):1912–8. doi: 10.1001/jama.2010.1567 21045097 PMC3107709

[25] Carroll DL, Dykes PC, Hurley AC. An electronic fall prevention toolkit: effect on documentation quality. Nurs Res. 2012;61(4):309–13. doi: 10.1097/NNR.0b013e31825569de 22592389

[26] Dykes PC, Burns Z, Adelman J, Benneyan J, Bogaisky M, Carter E, et al. Evaluation of a patient-centered fall-prevention tool kit to reduce falls and injuries: a nonrandomized controlled trial. JAMA Netw Open. 2020;3(11):e2025889. doi: 10.1001/jamanetworkopen.2020.25889 33201236 PMC7672520

[27] Aizen E, Lutsyk G, Wainer L, Carmeli S. Effectiveness of individualized fall prevention program in geriatric rehabilitation hospital setting: a cluster randomized trial. Aging Clin Exp Res. 2015;27(5):681–8. doi: 10.1007/s40520-015-0330-7 25697080

[28] Barker AL, Morello RT, Wolfe R, Brand CA, Haines TP, Hill KD, et al. 6-PACK programme to decrease fall injuries in acute hospitals: cluster randomised controlled trial. BMJ. 2016;352:h6781. doi: 10.1136/bmj.h6781 26813674 PMC4727091

[29] Mebrahtu TF, Skyrme S, Randell R, Keenan A-M, Bloor K, Yang H, et al. Effects of computerised clinical decision support systems (CDSS) on nursing and allied health professional performance and patient outcomes: a systematic review of experimental and observational studies. BMJ Open. 2021;11(12):e053886. doi: 10.1136/bmjopen-2021-053886 34911719 PMC8679061

[30] Damoiseaux-Volman BA, van der Velde N, Ruige SG, Romijn JA, Abu-Hanna A, Medlock S. Effect of interventions with a clinical decision support system for hospitalized older patients: systematic review mapping implementation and design factors. JMIR Med Inform. 2021;9(7):e28023. doi: 10.2196/28023 34269682 PMC8325084

[31] Yourman L, Concato J, Agostini JV. Use of computer decision support interventions to improve medication prescribing in older adults: a systematic review. Am J Geriatr Pharmacother. 2008;6(2):119–29. doi: 10.1016/j.amjopharm.2008.06.001 18675770

[32] Kwan JL, Lo L, Ferguson J, Goldberg H, Diaz-Martinez JP, Tomlinson G, et al. Computerised clinical decision support systems and absolute improvements in care: meta-analysis of controlled clinical trials. BMJ. 2020;370:m3216. doi: 10.1136/bmj.m3216 32943437 PMC7495041

[33] Moja L, Kwag KH, Lytras T, Bertizzolo L, Brandt L, Pecoraro V, et al. Effectiveness of computerized decision support systems linked to electronic health records: a systematic review and meta-analysis. Am J Public Health. 2014;104(12):e12–22. doi: 10.2105/AJPH.2014.302164 25322302 PMC4232126

[34] Garg AX, Adhikari NKJ, McDonald H, Rosas-Arellano MP, Devereaux PJ, Beyene J, et al. Effects of computerized clinical decision support systems on practitioner performance and patient outcomes: a systematic review. JAMA. 2005;293(10):1223–38. doi: 10.1001/jama.293.10.1223 15755945

[35] Jaspers MWM, Smeulers M, Vermeulen H, Peute LW. Effects of clinical decision-support systems on practitioner performance and patient outcomes: a synthesis of high-quality systematic review findings. J Am Med Inform Assoc. 2011;18(3):327–34. doi: 10.1136/amiajnl-2011-000094 21422100 PMC3078663

[36] Taheri Moghadam S, Sadoughi F, Velayati F, Ehsanzadeh SJ, Poursharif S. The effects of clinical decision support system for prescribing medication on patient outcomes and physician practice performance: a systematic review and meta-analysis. BMC Med Inform Decis Mak. 2021;21(1):98. doi: 10.1186/s12911-020-01376-8 33691690 PMC7944637

[37] Shahmoradi L, Safdari R, Ahmadi H, Zahmatkeshan M. Clinical decision support systems-based interventions to improve medication outcomes: a systematic literature review on features and effects. Med J Islam Repub Iran. 2021;35:27. doi: 10.47176/mjiri.35.27 34169039 PMC8214039

[38] Elley CR, Robertson MC, Garrett S, Kerse NM, McKinlay E, Lawton B, et al. Effectiveness of a falls-and-fracture nurse coordinator to reduce falls: a randomized, controlled trial of at-risk older adults. J Am Geriatr Soc. 2008;56(8):1383–9. doi: 10.1111/j.1532-5415.2008.01802.x 18808597

[39] Tamblyn R, Eguale T, Buckeridge DL, Huang A, Hanley J, Reidel K, et al. The effectiveness of a new generation of computerized drug alerts in reducing the risk of injury from drug side effects: a cluster randomized trial. J Am Med Inform Assoc. 2012;19(4):635–43. doi: 10.1136/amiajnl-2011-000609 22246963 PMC3384117

[40] Shamseer L, Moher D, Clarke M, Ghersi D, Liberati A, Petticrew M, et al. Preferred reporting items for systematic review and meta-analysis protocols (PRISMA-P) 2015: elaboration and explanation. BMJ. 2015;350:g7647. doi: 10.1136/bmj.g7647 25555855

[41] Higgins J, Thomas J, Chandler J, Cumpston M, Li T, Page M. Cochrane Handbook for Systematic Reviews of Interventions version 6.4 (updated August 2023). Cochrane; 2023.

[42] EPOC resources for review authors. [cited 22 Nov 2020]. Available from: https://epoc.cochrane.org/resources/epoc-resources-review-authors

[43] Page MJ, McKenzie JE, Bossuyt PM, Boutron I, Hoffmann TC, Mulrow CD, et al. The PRISMA 2020 statement: an updated guideline for reporting systematic reviews. BMJ. 2021;372:n71. doi: 10.1136/bmj.n71 33782057 PMC8005924

[44] Covidence Systematic Review Tool. In: Covidence [Internet]. [cited 17 May 2025]. Available from: https://www.covidence.org/

[45] Griese-Mammen N, Hersberger KE, Messerli M, Leikola S, Horvat N, van Mil JWF, et al. PCNE definition of medication review: reaching agreement. Int J Clin Pharm. 2018;40(5):1199–208. doi: 10.1007/s11096-018-0696-7 30073611

[46] El-Khoury F, Cassou B, Charles M-A, Dargent-Molina P. The effect of fall prevention exercise programmes on fall induced injuries in community dwelling older adults. Br J Sports Med. 2015;49(20):1348–1348. doi: 10.1136/bmj.f6234 26429908

[47] Seppala LJ, van de Glind EMM, Daams JG, Ploegmakers KJ, de Vries M, Wermelink AMAT, et al. Fall-risk-increasing drugs: a systematic review and meta-analysis: III. others. J Am Med Dir Assoc. 2018;19(4):372.e1–372.e8. doi: 10.1016/j.jamda.2017.12.099 29402646

[48] Hoffmann TC, Glasziou PP, Boutron I, Milne R, Perera R, Moher D, et al. Better reporting of interventions: template for intervention description and replication (TIDieR) checklist and guide. BMJ. 2014;348:g1687. doi: 10.1136/bmj.g1687 24609605

[49] Lewin S, Hendry M, Chandler J, Oxman AD, Michie S, Shepperd S, et al. Assessing the complexity of interventions within systematic reviews: development, content and use of a new tool (iCAT_SR). BMC Med Res Methodol. 2017;17(1):76. doi: 10.1186/s12874-017-0349-x 28446138 PMC5406941

[50] Lamb SE, Becker C, Gillespie LD, Smith JL, Finnegan S, Potter R, et al. Reporting of complex interventions in clinical trials: development of a taxonomy to classify and describe fall-prevention interventions. Trials. 2011;12:125. doi: 10.1186/1745-6215-12-125 21586143 PMC3127768

[51] Van de Velde S, Kunnamo I, Roshanov P, Kortteisto T, Aertgeerts B, Vandvik PO, et al. The GUIDES checklist: development of a tool to improve the successful use of guideline-based computerised clinical decision support. Implement Sci. 2018;13(1):86. doi: 10.1186/s13012-018-0772-3 29941007 PMC6019508

[52] Medlock S, Wyatt JC, Patel VL, Shortliffe EH, Abu-Hanna A. Modeling information flows in clinical decision support: key insights for enhancing system effectiveness. J Am Med Inform Assoc. 2016;23(5):1001–6. doi: 10.1093/jamia/ocv177 26911809 PMC11741008

[53] Sterne JAC, Savović J, Page MJ, Elbers RG, Blencowe NS, Boutron I, et al. RoB 2: a revised tool for assessing risk of bias in randomised trials. BMJ. 2019;366:l4898. doi: 10.1136/bmj.l4898 31462531

[54] Sterne JA, Hernán MA, Reeves BC, Savović J, Berkman ND, Viswanathan M, et al. ROBINS-I: a tool for assessing risk of bias in non-randomised studies of interventions. BMJ. 2016;355:i4919. doi: 10.1136/bmj.i4919 27733354 PMC5062054

[55] Sterne J, Hernán M, McAleenan A, Reeves B, Higgins J. Chapter 25: Assessing risk of bias in a non-randomized study. In: Higgins JPT, Thomas J, Chandler J, Cumpston M, Li T, Page MJ, et al., editors. Cochrane Handbook for Systematic Reviews of Interventions version 6.4 (updated August 2023). Cochrane; 2023. http://www.training.cochrane.org/handbook

[56] Campbell M, McKenzie JE, Sowden A, Katikireddi SV, Brennan SE, Ellis S, et al. Synthesis without meta-analysis (SWiM) in systematic reviews: reporting guideline. BMJ. 2020;368:l6890. doi: 10.1136/bmj.l6890 31948937 PMC7190266

[57] McKenzie J, Brennan S. Chapter 12: Synthesizing and presenting findings using other methods. In: Higgins JPT, Thomas J, Chandler J, Cumpston M, Li T, Page MJ, et al., editors. Cochrane Handbook for Systematic Reviews of Interventions version 6.4 (updated August 2023). Cochrane; 2023. http://www.training.cochrane.org/handbook

[58] Tricco AC, Thomas SM, Veroniki AA, Hamid JS, Cogo E, Strifler L, et al. Comparisons of interventions for preventing falls in older adults: a systematic review and meta-analysis. JAMA. 2017;318(17):1687–99. doi: 10.1001/jama.2017.15006 29114830 PMC5818787

[59] Deeks JJ, Higgins JP, Altman DG. Chapter 10: Analysing data and undertaking meta-analyses. In: Higgins JPT, Thomas J, Chandler J, Cumpston M, Li T, Page MJ, et al., editors. Cochrane Handbook for Systematic Reviews of Interventions version 6.4 (updated August 2023). Cochrane; 2023. www.training.cochrane.org/handbook

[60] StataCorp. Stata Statistical Software: Release 18. College Station, TX: StataCorp; 2023.

[61] Higgins J, Li T, Deeks J. Chapter 6: Choosing effect measures and computing estimates of effect. In: Higgins JPT, Thomas J, Chandler J, Cumpston M, Li T, Page MJ, et al., editors. Cochrane Handbook for Systematic Reviews of Interventions version 6.4 (updated August 2023). Cochrane; 2023. http://www.training.cochrane.org/handbook

[62] Bergen G, Stevens MR, Burns ER. Falls and fall injuries among adults aged ≥65 years - United States, 2014. MMWR Morb Mortal Wkly Rep. 2016;65(37):993–8. doi: 10.15585/mmwr.mm6537a2 27656914

[63] Guyatt G, Zeng L, Brignardello-Petersen R, Prasad M, De Beer H, Murad MH, et al. Core GRADE 2: choosing the target of certainty rating and assessing imprecision. BMJ. 2025;389:e081904. doi: 10.1136/bmj-2024-081904 40300802

[64] Egger M, Davey Smith G, Schneider M, Minder C. Bias in meta-analysis detected by a simple, graphical test. BMJ. 1997;315(7109):629–34. doi: 10.1136/bmj.315.7109.629 9310563 PMC2127453

[65] Hultcrantz M, Rind D, Akl EA, Treweek S, Mustafa RA, Iorio A, et al. The GRADE Working Group clarifies the construct of certainty of evidence. J Clin Epidemiol. 2017;87:4–13. doi: 10.1016/j.jclinepi.2017.05.006 28529184 PMC6542664

[66] Zeng L, Brignardello-Petersen R, Hultcrantz M, Mustafa RA, Murad MH, Iorio A, et al. GRADE Guidance 34: update on rating imprecision using a minimally contextualized approach. J Clin Epidemiol. 2022;150:216–24. doi: 10.1016/j.jclinepi.2022.07.014 35934265

[67] Santesso N, Glenton C, Dahm P, Garner P, Akl EA, Alper B, et al. GRADE guidelines 26: informative statements to communicate the findings of systematic reviews of interventions. J Clin Epidemiol. 2020;119:126–35. doi: 10.1016/j.jclinepi.2019.10.014 31711912

[68] Guyatt G, Yao L, Murad MH, Hultcrantz M, Agoritsas T, De Beer H, et al. Core GRADE 6: presenting the evidence in summary of findings tables. BMJ. 2025;389:e083866. doi: 10.1136/bmj-2024-083866 40425239

[69] Clemson L, Mackenzie L, Lovarini M, Roberts C, Poulos R, Sherrington C, et al. Integrated solutions for sustainable fall prevention in primary care: a pragmatic hybrid-type 2 mixed methods implementation and effectiveness study. Front Public Health. 2024;12:1446525. doi: 10.3389/fpubh.2024.1446525 39703488 PMC11656318

[70] Phelan EA, Williamson BD, Balderson BH, Cook AJ, Piccorelli AV, Fujii MM, et al. Reducing central nervous system-active medications to prevent falls and injuries among older adults: a cluster randomized clinical trial. JAMA Netw Open. 2024;7(7):e2424234. doi: 10.1001/jamanetworkopen.2024.24234 39052289 PMC11273227

[71] Bhasin S, Gill TM, Reuben DB, Latham NK, Ganz DA, Greene EJ, et al. A randomized trial of a multifactorial strategy to prevent serious fall injuries. N Engl J Med. 2020;383(2):129–40. doi: 10.1056/NEJMoa2002183 32640131 PMC7421468

[72] Blum MR, Sallevelt BTGM, Spinewine A, O’Mahony D, Moutzouri E, Feller M, et al. Optimizing Therapy to Prevent Avoidable Hospital Admissions in Multimorbid Older Adults (OPERAM): cluster randomised controlled trial. BMJ. 2021;374:n1585. doi: 10.1136/bmj.n1585 34257088 PMC8276068

[73] Byrne CM. Impact of prospective computerized clinical decision support information and targeted assistance on nursing home resident outcomes. University of Albany, State University of New York. 2005. Available from: https://www.proquest.com/openview/59a276fe7e8913563fcd2bb9b487d19d/1?pq-origsite=gscholar&cbl=18750&diss=y

[74] Ferrer A, Formiga F, Sanz H, de Vries OJ, Badia T, Pujol R, et al. Multifactorial assessment and targeted intervention to reduce falls among the oldest-old: a randomized controlled trial. Clin Interv Aging. 2014;9:383–93. doi: 10.2147/CIA.S57580 24596458 PMC3940644

[75] Frankenthal D, Lerman Y, Kalendaryev E, Lerman Y. Intervention with the screening tool of older persons potentially inappropriate prescriptions/screening tool to alert doctors to right treatment criteria in elderly residents of a chronic geriatric facility: a randomized clinical trial. J Am Geriatr Soc. 2014;62(9):1658–65. doi: 10.1111/jgs.12993 25243680

[76] Gallagher PF, O’Connor MN, O’Mahony D. Prevention of potentially inappropriate prescribing for elderly patients: a randomized controlled trial using STOPP/START criteria. Clin Pharmacol Ther. 2011;89(6):845–54. doi: 10.1038/clpt.2011.44 21508941

[77] Ganz DA, Kim S-B, Zingmond DS, Ramirez KD, Roth CP, Jennings LA, et al. Effect of a falls quality improvement program on serious fall-related injuries. J Am Geriatr Soc. 2015;63(1):63–70. doi: 10.1111/jgs.13154 25597558 PMC4299937

[78] Wenger NS, Roth CP, Hall WJ, Ganz DA, Snow V, Byrkit J, et al. Practice redesign to improve care for falls and urinary incontinence: primary care intervention for older patients. Arch Intern Med. 2010;170(19):1765–72. doi: 10.1001/archinternmed.2010.387 20975026

[79] Ganz DA, Yuan AH, Greene EJ, Latham NK, Araujo K, Siu AL, et al. Effect of the STRIDE fall injury prevention intervention on falls, fall injuries, and health-related quality of life. J Am Geriatr Soc. 2022;70(11):3221–9. doi: 10.1111/jgs.17964 35932279 PMC9669115

[80] Groshaus H, Boscan A, Khandwala F, Holroyd-Leduc J. Use of clinical decision support to improve the quality of care provided to older hospitalized patients. Appl Clin Inform. 2012;3(1):94–102. doi: 10.4338/ACI-2011-08-RA-0047 23616902 PMC3613010

[81] Healey F, Monro A, Cockram A, Adams V, Heseltine D. Using targeted risk factor reduction to prevent falls in older in-patients: a randomised controlled trial. Age Ageing. 2004;33(4):390–5. doi: 10.1093/ageing/afh130 15151914

[82] Lightbody E, Watkins C, Leathley M, Sharma A, Lye M. Evaluation of a nurse-led falls prevention programme versus usual care: a randomized controlled trial. Age Ageing. 2002;31(3):203–10. doi: 10.1093/ageing/31.3.203 12006310

[83] Mahoney JE, Shea TA, Przybelski R, Jaros L, Gangnon R, Cech S, et al. Kenosha County falls prevention study: a randomized, controlled trial of an intermediate-intensity, community-based multifactorial falls intervention. J Am Geriatr Soc. 2007;55(4):489–98. doi: 10.1111/j.1532-5415.2007.01144.x 17397425

[84] Logan PA, Horne JC, Gladman JRF, Gordon AL, Sach T, Clark A, et al. Multifactorial falls prevention programme compared with usual care in UK care homes for older people: multicentre cluster randomised controlled trial with economic evaluation. BMJ. 2021;375:e066991. doi: 10.1136/bmj-2021-066991 34876412 PMC8649897

[85] Peterson JF, Rosenbaum BP, Waitman LR, Habermann R, Powers J, Harrell D, et al. Physicians’ response to guided geriatric dosing: initial results from a randomized trial. Stud Health Technol Inform. 2007;129(Pt 2):1037–40. 17911873

[86] Snooks HA, Carter B, Dale J, Foster T, Humphreys I, Logan PA, et al. Support and Assessment for Fall Emergency Referrals (SAFER 1): cluster randomised trial of computerised clinical decision support for paramedics. PLoS One. 2014;9(9):e106436. doi: 10.1371/journal.pone.0106436 25216281 PMC4162545

[87] Weber V, White A, McIlvried R. An electronic medical record (EMR)-based intervention to reduce polypharmacy and falls in an ambulatory rural elderly population. J Gen Intern Med. 2008;23(4):399–404. doi: 10.1007/s11606-007-0482-z 18373136 PMC2359523

[88] Wenger NS, Roth CP, Shekelle PG, Young RT, Solomon DH, Kamberg CJ, et al. A practice-based intervention to improve primary care for falls, urinary incontinence, and dementia. J Am Geriatr Soc. 2009;57(3):547–55. doi: 10.1111/j.1532-5415.2008.02128.x 19175441

[89] Sutton RT, Pincock D, Baumgart DC, Sadowski DC, Fedorak RN, Kroeker KI. An overview of clinical decision support systems: benefits, risks, and strategies for success. NPJ Digit Med. 2020;3:17. doi: 10.1038/s41746-020-0221-y 32047862 PMC7005290

[90] Hopewell S, Adedire O, Copsey BJ, Boniface GJ, Sherrington C, Clemson L, et al. Multifactorial and multiple component interventions for preventing falls in older people living in the community. Cochrane Database Syst Rev. 2018;7(7):CD012221. doi: 10.1002/14651858.CD012221.pub2 30035305 PMC6513234

[91] Wang H, Boissel J-P, Nony P. Revisiting the relationship between baseline risk and risk under treatment. Emerg Themes Epidemiol. 2009;6:1. doi: 10.1186/1742-7622-6-1 19222846 PMC2646709

[92] Ho KM. Importance of the baseline risk in determining sample size and power of a randomized controlled trial. J Emerg Crit Care Med. 2018;2:70–70. doi: 10.21037/jeccm.2018.08.06

[93] Goodwin V, Jones-Hughes T, Thompson-Coon J, Boddy K, Stein K. Implementing the evidence for preventing falls among community-dwelling older people: a systematic review. J Safety Res. 2011;42(6):443–51. doi: 10.1016/j.jsr.2011.07.008 22152262

[94] Finnegan S, Seers K, Bruce J. Long-term follow-up of exercise interventions aimed at preventing falls in older people living in the community: a systematic review and meta-analysis. Physiotherapy. 2019;105(2):187–99. doi: 10.1016/j.physio.2018.09.002 30846193

[95] Wang T, Tan J-YB, Liu X-L, Zhao I. Barriers and enablers to implementing clinical practice guidelines in primary care: an overview of systematic reviews. BMJ Open. 2023;13(1):e062158. doi: 10.1136/bmjopen-2022-062158 36609329 PMC9827241

[96] Leonard E, de Kock I, Bam W. Barriers and facilitators to implementing evidence-based health innovations in low- and middle-income countries: a systematic literature review. Eval Program Plann. 2020;82:101832. doi: 10.1016/j.evalprogplan.2020.101832 32585317

[97] Schünemann H, Brożek J, Guyatt G, Oxman A. GRADE Handbook: Introduction to GRADE Handbook. 2013. https://gdt.gradepro.org/app/handbook/handbook.html

