# Supplementary material for: Effectiveness of clinical decision support in fall prevention among older adults: A systematic review and meta-analysis
Source: PLoS One. 2026 Jan 12;21(1):e0340025. doi: 10.1371/journal.pone.0340025 (PMC12795367; doi:10.1371/journal.pone.0340025)
Supplement: S2 Table — (DOCX) [file pone.0340025.s002.docx]

**S2 Table. Differences between protocol and review**

| Details of difference | Comments |
| --- | --- |
| Research question two (what are the factors of effective CDS in fall prevention among community-dwelling older adults?) was removed. | After extracting data on the CDS tools in the included studies, we saw that the reporting of aspects regarding the CDS content, and especially the CDS system, was so limited that assessing the determinants of effective CDS was not possible |
| Study eligibility criteria were changed | After screening titles, abstracts, and full texts for eligibility, but before synthesising results, we changed study eligibility criteria to include studies that (1) included both computerised and non-computerised CDS tools, and (2) were conducted in any healthcare setting or in the homes of older adults. |
| Specification of subgroup analyses | After screening titles, abstracts, and full texsts for eligibility, but before synthesizing results, we created a detailed plan for subgroup analyses. The three factors of risk of bias, study setting, and patient age were chosen as there were at least two studies in each subgroup for these factors. |
| Specification of syntheses for which publication bias was assessed | We did not select syntheses to assess for risk of publication bias when writing the protocol. |
