# Supplementary material for: Effectiveness of clinical decision support in fall prevention among older adults: A systematic review and meta-analysis
Source: PLoS One. 2026 Jan 12;21(1):e0340025. doi: 10.1371/journal.pone.0340025 (PMC12795367; doi:10.1371/journal.pone.0340025)
Supplement: S3 Table — (DOCX) [file pone.0340025.s003.docx]

**S3 Table: Electronic searches**

The search was performed publications from the earliest available date through April 29^th^, 2021. An updated search was performed in January, 2025. An updated search was performed in January 2025.

**Original literature searches through April 29^th^ 2021**

The original search was for publications from the earliest available date through April 29^th^, 2021.

**Database:** MEDLINE(R) and Epub Ahead of Print, In-Process, In-Data-Review & Other Non-Indexed Citations and Daily <1946 to April 21, 2021> (OVID)
**Date:** 22.04.2021
**Results:** 1656

| 1 | Accidental Falls/ | 25414 |
| --- | --- | --- |
| 2 | ((fall or falls or faller* or fallen or falling or slip or slips or slipping) adj2 (reduce* or reduction* or reducing* or manag* or risk* or avoid* or decreas* or predict*)).tw,kw,kf. | 14622 |
| 3 | ((fall or falls or faller* or fallen or falling or slip or slips or slipping) adj4 prevent*).tw,kw,kf. | 8525 |
| 4 | (fall or falls or faller* or fallen or falling or slip or slips or slipping).ti,kw. | 26460 |
| 5 | or/1-4 | 47338 |
| 6 | accident prevention/ | 9207 |
| 7 | primary prevention/ or secondary prevention/ or tertiary prevention/ | 38937 |
| 8 | safety management/ or Risk Management/ | 38251 |
| 9 | Harm Reduction/ | 3318 |
| 10 | ((fall or falls or faller* or fallen or falling or slip or slips or slipping) adj2 (reduce* or reduction* or reducing* or manag* or risk* or avoid* or decreas* or predict*)).tw,kw,kf. | 14622 |
| 11 | ((fall or falls or faller* or fallen or falling or slip or slips or slipping) adj4 prevent*).tw,kw,kf. | 8525 |
| 12 | (number* adj2 fall*).tw,kw,kf. | 1791 |
| 13 | Prevention & Control.fs. | 1332190 |
| 14 | or/6-13 | 1390885 |
| 15 | 5 and 14 | 24756 |
| 16 | decision support systems, clinical/ or medical records systems, computerized/ or decision support techniques/ or clinical decision rules/ or Medical Informatics Applications/ | 49630 |
| 17 | electronic health records/ or health information exchange/ | 22313 |
| 18 | Reminder Systems/ | 3594 |
| 19 | Drug Therapy, Computer-Assisted/ or Therapy, Computer-Assisted/ | 8460 |
| 20 | Medical Order Entry Systems/ | 2317 |
| 21 | hospital information systems/ or ambulatory care information systems/ or point-of-care systems/ or Information Systems/ | 44317 |
| 22 | algorithms/ | 265487 |
| 23 | (Decision* adj3 (Support* or tool*)).tw,kw,kf. | 31468 |
| 24 | (("Stop* Elderly Accident* Death*" adj2 Injur*) or STEADI).tw,kw,kf. | 60 |
| 25 | (cds or cdss or ccdss or dcss or ddss or algorithm* or alert* or reminder* or order entry or toolkit or tool kit or FPTK* or health information* technolog* or information exchange or electronic health record*).tw,kw,kf. | 379847 |
| 26 | (HIT adj2 (application* or software or system*)).tw,kw,kf. | 292 |
| 27 | (fall* adj2 screen*).tw,kw,kf. | 458 |
| 28 | (screening or screened).ti,kw. | 173828 |
| 29 | ((information or delivery or medical* or point of care) adj system*).tw,kw,kf. | 110767 |
| 30 | ((point of care or workflow) adj2 tool*).tw,kw,kf. | 637 |
| 31 | or/16-30 | 906753 |
| 32 | 15 and 31 | 1656 |

**Database:** EMBASE <1974 to 2021 April 21> (OVID)
**Date:** 22.04.2021
**Results:** 3007 (av disse er 906 conference abstracts som leveres i separat EN-fil)

| 1 | falling/ | 41448 |
| --- | --- | --- |
| 2 | fall risk assessment/ or fall risk/ | 6123 |
| 3 | ((fall or falls or faller* or fallen or falling or slip or slips or slipping) adj2 (reduce* or reduction* or reducing* or manag* or risk* or avoid* or decreas* or predict*)).tw,kw. | 21820 |
| 4 | ((fall or falls or faller* or fallen or falling or slip or slips or slipping) adj4 prevent*).tw,kw. | 11541 |
| 5 | (fall or falls or faller* or fallen or falling or slip or slips or slipping).ti,kw. | 34927 |
| 6 | or/1-5 | 74704 |
| 7 | prevention/ | 296977 |
| 8 | "prevention and control"/ | 28273 |
| 9 | accident prevention/ | 15957 |
| 10 | primary prevention/ | 42275 |
| 11 | secondary prevention/ | 30157 |
| 12 | tertiary prevention/ | 474 |
| 13 | harm reduction/ | 7204 |
| 14 | risk management/ | 44008 |
| 15 | fall risk assessment/ or fall risk/ | 6123 |
| 16 | prevention.fx. | 1144408 |
| 17 | ((fall or falls or faller* or fallen or falling or slip or slips or slipping) adj2 (reduce* or reduction* or reducing* or manag* or risk* or avoid* or decreas* or predict*)).tw,kw. | 21820 |
| 18 | ((fall or falls or faller* or fallen or falling or slip or slips or slipping) adj4 prevent*).tw,kw. | 11541 |
| 19 | (number* adj2 fall*).tw,kw. | 2654 |
| 20 | or/7-19 | 1564764 |
| 21 | 6 and 20 | 35471 |
| 22 | clinical decision support system/ or decision support system/ or medical decision making/ or clinical decision rule/ | 114927 |
| 23 | electronic health record/ | 21951 |
| 24 | electronic medical record/ | 60419 |
| 25 | drug surveillance program/ | 26356 |
| 26 | medical information system/ or information system/ | 60504 |
| 27 | algorithm/ or risk algorithm/ or detection algorithm/ or dose calculation algorithm/ | 294252 |
| 28 | computer assisted therapy/ or computer assisted drug therapy/ | 5629 |
| 29 | physician order entry system/ | 275 |
| 30 | hospital information system/ or "point of care system"/ | 22672 |
| 31 | reminder system/ or information system/ | 41848 |
| 32 | computerized provider order entry/ | 2049 |
| 33 | screening/ | 179391 |
| 34 | (Decision* adj3 (Support* or tool*)).tw,kw. | 43740 |
| 35 | (("Stop* Elderly Accident* Death*" adj2 Injur*) or STEADI).tw,kw. | 85 |
| 36 | (cds or cdss or ccdss or dcss or ddss or algorithm* or alert* or reminder* or order entry or toolkit or tool kit or FPTK* or health information* technolog* or information exchange or electronic health record*).tw,kw. | 504901 |
| 37 | (HIT adj2 (application* or software or system*)).tw,kw. | 379 |
| 38 | (fall* adj2 screen*).tw,kw. | 692 |
| 39 | (screening or screened).ti,kw. | 267355 |
| 40 | ((information or delivery or medical* or point of care) adj system*).tw,kw. | 152407 |
| 41 | ((point of care or workflow) adj2 tool*).tw,kw. | 894 |
| 42 | or/22-41 | 1374421 |
| 43 | 21 and 42 | 3007 |
| 44 | limit 43 to conference abstracts | 906 |
| 45 | 43 not 44 | 2101 |

**Database:** Cochrane Library
**Date:** 22.04.2021
**Results**: 375 (4 Cochrane reviews, 367 trials, 4 editorials)

| ID | Search | Hits |
| --- | --- | --- |
| #1 | MeSH descriptor: [Accidental Falls] this term only | 1531 |
| #2 | ((fall or falls or faller* or fallen or falling or slip or slips or slipping) NEAR/2 (reduce* or reduction* or reducing* or manag* or risk* or avoid* or decreas* or predict*)):ti,ab,kw | 3487 |
| #3 | ((fall or falls or faller* or fallen or falling or slip or slips or slipping) NEAR/4 prevent*):ti,ab,kw | 2751 |
| #4 | (fall or falls or faller* or fallen or falling or slip or slips or slipping):ti,kw | 4573 |
| #5 | {OR #1-#4} | 6426 |
| #6 | MeSH descriptor: [Accident Prevention] this term only | 134 |
| #7 | MeSH descriptor: [Primary Prevention] this term only | 880 |
| #8 | MeSH descriptor: [Secondary Prevention] this term only | 3215 |
| #9 | MeSH descriptor: [Tertiary Prevention] this term only | 4 |
| #10 | MeSH descriptor: [Safety Management] this term only | 173 |
| #11 | MeSH descriptor: [Risk Management] this term only | 125 |
| #12 | MeSH descriptor: [Harm Reduction] this term only | 129 |
| #13 | ((fall or falls or faller* or fallen or falling or slip or slips or slipping) NEAR/2 (reduce* or reduction* or reducing* or manag* or risk* or avoid* or decreas* or predict*)):ti,ab,kw | 3487 |
| #14 | ((fall or falls or faller* or fallen or falling or slip or slips or slipping) NEAR/4 prevent*):ti,ab,kw | 2751 |
| #15 | (number* NEAR/2 fall*):ti,ab,kw | 640 |
| #16 | (prevent* or reduce* or reduction* or reducing* or manag* or safety or avoid* or decreas* or predict*):ti,kw | 441800 |
| #17 | MeSH descriptor: [] explode all trees and with qualifier(s): [prevention & control - PC] | 94391 |
| #18 | {OR #6-#17} | 444073 |
| #19 | #5 AND #18 | 5554 |
| #20 | MeSH descriptor: [Decision Support Systems, Clinical] this term only | 392 |
| #21 | MeSH descriptor: [Medical Records Systems, Computerized] this term only | 197 |
| #22 | MeSH descriptor: [Decision Support Techniques] this term only | 823 |
| #23 | MeSH descriptor: [Clinical Decision Rules] this term only | 12 |
| #24 | MeSH descriptor: [Medical Informatics Applications] this term only | 23 |
| #25 | MeSH descriptor: [Electronic Health Records] this term only | 373 |
| #26 | MeSH descriptor: [Health Information Exchange] this term only | 7 |
| #27 | MeSH descriptor: [Reminder Systems] this term only | 967 |
| #28 | MeSH descriptor: [Drug Therapy, Computer-Assisted] this term only | 150 |
| #29 | MeSH descriptor: [Therapy, Computer-Assisted] this term only | 1340 |
| #30 | MeSH descriptor: [Medical Order Entry Systems] this term only | 68 |
| #31 | MeSH descriptor: [Hospital Information Systems] this term only | 47 |
| #32 | MeSH descriptor: [Ambulatory Care Information Systems] this term only | 25 |
| #33 | MeSH descriptor: [Point-of-Care Systems] this term only | 433 |
| #34 | MeSH descriptor: [Information Systems] this term only | 59 |
| #35 | MeSH descriptor: [Algorithms] this term only | 3280 |
| #36 | (Decision* NEAR/3 (Support* or tool*)):ti,ab,kw | 4204 |
| #37 | (("Stop* Elderly Accident* Death*" NEAR/2 Injur*) or STEADI):ti,ab,kw | 12 |
| #38 | (cds or cdss or ccdss or dcss or ddss or algorithm* or alert* or reminder* or order entry or toolkit or tool kit or FPTK* or health information* technolog* or information exchange or electronic health record*):ti,ab,kw | 33250 |
| #39 | (HIT NEAR/2 (application* or software or system*)):ti,ab,kw | 18 |
| #40 | (fall* NEAR/2 screen*):ti,ab,kw | 51 |
| #41 | (screening or screened):ti,kw | 19295 |
| #42 | ((information or delivery or medical* or point of care) NEXT system*):ti,ab,kw | 21249 |
| #43 | ((point of care or workflow) NEAR/2 tool*):ti,ab,kw | 3210 |
| #44 | {OR #20-#43} | 76681 |
| #45 | #19 AND #44 | 375 |

**Database:** CINAHL with Full Text (EBSCO)
**Date:** 23.04.2021
**Results:** 950

| **Search ID#** | **Search Terms** | **Results** |
| --- | --- | --- |
| S1 | (MH "Accidental Falls") | 23,807 |
| S2 | (MH "Fall Risk Assessment Tool") OR (MH "Hendrich Fall Risk Model") | 193 |
| S3 | TI ( (fall or falls or faller* or fallen or falling or slip or slips or slipping) N1 (reduce* or reduction* or reducing* or manag* or risk* or avoid* or decreas* or predict*) ) OR AB ( (fall or falls or faller* or fallen or falling or slip or slips or slipping) N1 (reduce* or reduction* or reducing* or manag* or risk* or avoid* or decreas* or predict*) ) | 9,444 |
| S4 | TI ( ((fall or falls or faller* or fallen or falling or slip or slips or slipping) N3 prevent*) ) OR AB ( ((fall or falls or faller* or fallen or falling or slip or slips or slipping) N3 prevent*) ) | 5,985 |
| S5 | TI fall or falls or faller* or fallen or falling or slip or slips or slipping | 62,354 |
| S6 | S1 OR S2 OR S3 OR S4 OR S5 | 62,354 |
| S7 | (MH "Preventive Health Care") | 21,163 |
| S8 | (MH "Home Safety") OR (MH "Safety") OR (MH "Patient Safety") | 93,610 |
| S9 | (MH "Harm Reduction") | 4,281 |
| S10 | (MH "Fall Risk Assessment Tool") OR (MH "Hendrich Fall Risk Model") | 193 |
| S11 | TI ( ((fall or falls or faller* or fallen or falling or slip or slips or slipping) N1 ((reduce* or reduction* or reducing* or manag* or risk* or avoid* or decreas* or predict*)) ) OR AB ( ((fall or falls or faller* or fallen or falling or slip or slips or slipping) N1 ((reduce* or reduction* or reducing* or manag* or risk* or avoid* or decreas* or predict*)) ) | 9,444 |
| S12 | TI ( ((fall or falls or faller* or fallen or falling or slip or slips or slipping) N3 prevent*) ) OR AB ( ((fall or falls or faller* or fallen or falling or slip or slips or slipping) N3 prevent*) ) | 5,985 |
| S13 | TI (number* N1 fall*) OR AB (number* N1 fall*) | 976 |
| S14 | S7 OR S8 OR S9 OR S10 OR S11 OR S12 OR S13 | 130,585 |
| S15 | S6 AND S14 | 15,666 |
| S16 | (MH "Decision Support Systems, Clinical") OR (MH "Decision Support Systems, Management") OR (MH "Decision Support Techniques") | 12,873 |
| S17 | (MH "Patient Record Systems") OR (MH "Electronic Health Records") OR (MH "Patient Portals") OR (MH "Clinical Information Systems") OR (MH "Information Systems") OR (MH "Health Information Systems") OR (MH "Ambulatory Care Information Systems") OR (MH "Clinical Pharmacy Information Systems") OR (MH "Home Health Care Information Systems") OR (MH "Nursing Information Systems") OR (MH "Nursing Care Plans, Computerized") | 49,989 |
| S18 | (MH "Clinical Prediction Rules") | 10 |
| S19 | (MH "Therapy, Computer Assisted") OR (MH "Decision Making, Computer Assisted") OR (MH "Drug Therapy, Computer Assisted") | 7,181 |
| S20 | (MH "Electronic Order Entry") | 3,370 |
| S21 | (MH "Reminder Systems") | 2,969 |
| S22 | (MH "Algorithms") | 39,282 |
| S23 | TI ( (Decision* N2 (support* or tool*) ) OR AB ( (Decision* N2 (support* or tool*) ) | 12,874 |
| S24 | TI ( (("Stop* Elderly Accident* Death*" N1 Injur*) or STEADI) ) OR AB ( (("Stop* Elderly Accident* Death*" N1 Injur*) or STEADI) ) | 45 |
| S25 | TI ( (cds or cdss or ccdss or dcss or ddss or algorithm* OR alert* OR reminder* OR "order entry" OR toolkit OR "tool kit" OR FPTK or "health information* technolog*") ) OR AB ( (cds or cdss or ccdss or dcss or ddss or algorithm* OR alert* OR reminder* OR "order entry" OR toolkit OR "tool kit" OR FPTK or "health information* technolog*") ) | 69,546 |
| S26 | TI ( (HIT N1 (application* or software or system*)) ) OR AB ( (HIT N1 (application* or software or system*)) ) | 128 |
| S27 | TI (fall* N1 screen*) OR AB (fall* N1 screen*) | 344 |
| S28 | TI (screening or screened) | 58,253 |
| S29 | TI ( ((information or delivery or medical* or "point of care") N0 system*) ) OR AB ( ((information or delivery or medical* or "point of care") N0 system*) ) | 24,917 |
| S30 | TI ( (("point of care" or workflow) N1 tool*) ) OR AB ( (("point of care" or workflow) N1 tool*) ) | 180 |
| S31 | S16 OR S17 OR S18 OR S19 OR S20 OR S21 OR S22 OR S23 OR S24 OR S25 OR S26 OR S27 OR S28 OR S29 OR S30 | 238,668 |
| S32 | S15 AND S31 | 950 |

**Database:** AMED (Allied and Complementary Medicine) <1985 to April 2021> (OVID)
**Date:** 23.04.2021
**Results:** 92

| **#** | **Query** | **Results** |
| --- | --- | --- |
| 1 | accidental falls/ | 2,460 |
| 2 | ((fall or falls or faller* or fallen or falling or slip or slips or slipping) adj2 (reduce* or reduction* or reducing* or manag* or risk* or avoid* or decreas* or predict*)).ti,ab. | 1,528 |
| 3 | ((fall or falls or faller* or fallen or falling or slip or slips or slipping) adj4 prevent*).ti,ab. | 763 |
| 4 | (fall or falls or faller* or fallen or falling or slip or slips or slipping).ti. | 2,000 |
| 5 | or/1-4 | 3,243 |
| 6 | accident prevention/ | 509 |
| 7 | prevention/ | 16,807 |
| 8 | ((fall or falls or faller* or fallen or falling or slip or slips or slipping) adj2 (reduce* or reduction* or reducing* or manag* or risk* or avoid* or decreas* or predict*)).ti,ab. | 1,528 |
| 9 | ((fall or falls or faller* or fallen or falling or slip or slips or slipping) adj4 prevent*).ti,ab. | 763 |
| 10 | (number* adj2 fall*).ti,ab. | 162 |
| 11 | or/6-10 | 18,034 |
| 12 | 5 and 11 | 2,538 |
| 13 | information systems/ | 138 |
| 14 | algorithms/ | 271 |
| 15 | (Decision* adj3 (Support* or tool*)).ti,ab. | 359 |
| 16 | (("Stop* Elderly Accident* Death*" adj2 Injur*) or STEADI).ti,ab. | 5 |
| 17 | (cds or cdss or ccdss or dcss or ddss or algorithm* or alert* or reminder* or order entry or toolkit or tool kit or FPTK* or health information* technolog* or information exchange or electronic health record*).ti,ab. | 1,756 |
| 18 | (HIT adj2 (application* or software or system*)).ti,ab. | 3 |
| 19 | (fall* adj2 screen*).ti,ab. | 54 |
| 20 | (screening or screened).ti. | 1,310 |
| 21 | ((information or delivery or medical* or point of care) adj system*).ti,ab. | 644 |
| 22 | ((point of care or workflow) adj2 tool*).ti,ab. | 0 |
| 23 | or/13-22 | 4,224 |
| 24 | 12 and 23 | 92 |

**Database:** Web of Science (Clarivate Analytics) Indexes=SCI-EXPANDED, SSCI, A&HCI, ESCI Timespan=1987-2021
**Date:** 23.04.2021
**Results:** 1784

| #1 | 18, 979 | TS=((fall or falls or faller* or fallen or falling or slip or slips or slipping) NEAR/1 (reduce* or reduction* or reducing* or manag* or risk* or avoid* or decreas* or predict*) ) |
| --- | --- | --- |
| #2 | 9,110 | TS=((fall or falls or faller* or fallen or falling or slip or slips or slipping) NEAR/3 prevent*) |
| #3 | 71,215 | TI=(fall or falls or faller* or fallen or falling or slip or slips or slipping) |
| #4 | 34,720 | AK=(fall or falls or faller* or fallen or falling or slip or slips or slipping) |
| #5 | 99,167 | #4 OR #3 OR #2 OR #1 |
| #6 | 18,979 | TS=((fall or falls or faller* or fallen or falling or slip or slips or slipping) NEAR/1 (reduce* or reduction* or reducing* or manag* or risk* or avoid* or decreas* or predict*) ) |
| #7 | 9,110 | TS=((fall or falls or faller* or fallen or falling or slip or slips or slipping) NEAR/3 prevent*) |
| #8 | 2,418 | TS=(number* NEAR/1 fall*) |
| #9 | 26,451 | #8 OR #7 OR #6 |
| #10 | 25,228 | #9 AND #5 |
| #11 | 74,197 | TS=(Decision* NEAR/2 (Support* or tool*) ) |
| #12 | 84 | TS=(("Stop* Elderly Accident* Death*" NEAR/1 Injur*) or STEADI) |
| #13 | 1,444,455 | TS=(cds or cdss or ccdss or dcss or ddss or algorithm* or alert* or reminder* or "order entry" or toolkit or "tool kit" or FPTK* or "health information* technolog*" or "information exchange" or "electronic health record*") |
| #14 | 483 | TS=(HIT NEAR/1 (application* or software or system*) ) |
| #15 | 467 | TS=(fall* NEAR/1 screen*) |
| #16 | 242,685 | TI=(screening or screened) |
| #17 | 105,726 | AK=(screening or screened) |
| #18 | 190,764 | TS=((information or delivery or medical* or "point of care") NEAR/0 system*) |
| #19 | 750 | TS=(("point of care" or workflow) NEAR/1 tool*) |
| #20 | 1,966,360 | #19 OR #18 OR #17 OR #16 OR #15 OR #14 OR #13 OR #12 OR #11 |
| #21 | 1,784 | #20 AND #10  Indexes=SCI-EXPANDED, SSCI, A&HCI, ESCI Timespan=All years |

**Database:** Google scholar via Harzing’s Publish or Perish (Windows GUI Edition) 7.15.2643.7260.
**Date:** 23.04.2021
**Results:** 300 (sortert på relevans, 300 første i listen valgt)

fall|falls|fallen|falling prevent|prevents|prevention|preventing "decision support"|cds|STEADI|toolkit|"tool kit"|FPTK|algorithm|"screen*fall"|reminders

**Updated Google scholar search**

**Database:** Google scholar via Publish or Perish.
**Date:** 29.01.2025
**Results:** 100 (sortert på relevans, 300 første i listen valgt)

Updated search string:

fall|falls|fallen|falling prevent|prevents|prevention|preventing "decision support"|cds|STEADI|toolkit|"tool kit"|FPTK|algorithm

**Database:** PEDro. All searches were made using advanced search with a filter by Method – clinical trial and Published since
**Date:** 23.05.2023

| Search ID | Query | Results |
| --- | --- | --- |
| #1 | fall* prevent* | 634 |
| #2 | “decision support” | 22 |
| #3 | decision tool | 18 |
| #4 | medical record system | 3 |
| #5 | algorithm fall* | 7 |
| #6 | software fall* | 12 |
| #7 | screen fall* | 9 |
| #8 | workflow fall* | 1 |
| Total |  | 706 |

**Updated PEDro search**

**Database:** PEDro. All searches were made using advanced search with a filter by Method “clinical trial” and Published since “2023”.
**Date:** 22.01.2025

| Search ID | Query | Results |
| --- | --- | --- |
| #1 | fall* prevent* | 43 |
| #2 | “decision support” | 1 |
| #3 | decision tool | 0 |
| #4 | medical record system | 1 |
| #5 | algorithm fall* | 0 |
| #6 | software fall* | 1 |
| #7 | screen fall* | 0 |
| #8 | workflow fall* | 0 |
| Total |  | 46 |
