# Supplementary material for: Effectiveness of clinical decision support in fall prevention among older adults: A systematic review and meta-analysis
Source: PLoS One. 2026 Jan 12;21(1):e0340025. doi: 10.1371/journal.pone.0340025 (PMC12795367; doi:10.1371/journal.pone.0340025)
Supplement: S5 Table — (DOCX) [file pone.0340025.s005.docx]

**S5 Table. Description of interventions and funding sources**

Seventeen (74%) studies were funded by the public sector, one (4%) from industry, one (4%) from a trust, one (4%) unfunded, and three (13%) studies failed to declare funding.

Aizen et al. (2015)

| Funding source | - |
| --- | --- |
| Type of intervention | Fall risk assessment and interventions based on CDS. |
| iCAT_SR domain 2: Active components included in the intervention, in relation to the comparison | **Judgement:**  More than one component and delivered as a bundle.  **Support for judgement:**  Fall risk assessment preceded the individualised fall prevention program; therefore bundle as opposed to package. |
| Notes |  |
| **TIDieR experimental intervention description** | |
| Where located | Journal article |
| Brief name | Targeted multiple intervention falls prevention program based on patient's fall risk |
| Why | - |
| What: Materials | Risk assessment tool. |
| What: Procedures | The intervention package was implemented as part of the usual routine work of the ward in participating wards. The intervention consisted of two parts: 1. Researchers assessed the risk of every patient admitted for rehabilitation once a week using the risk assessment tool. Patients were classified as having a high, moderate, mild, or minimal risk according to the tool. 2. Each week, and a day after each admission, after obtaining the risk score, patients classified as having mild, moderate or high risk were included in an individualized fall prevention program. Patients classified as having mild risk were assessed and managed individually according to the program. The assessment included medical interventions, environmental modifications, toilets and shower-room adjustment, mobility care, bed and wheelchair adjustment, behavioral and cognitive treatment and patient and family guidance. Moderate risk patients were assessed similarly, and in addition received regular orientation guidance, and was assessed for the need of a safety mobility resriction. The safety mobility restriction included assurance that mobility (transfers, walking, toilets usage, etc.) was done only under supervision and/or assistance of a professional staff member. High risk patients received the same treatment package as moderate risk patients, and were additionally placed in a visible location in the ward. Permanent personal supervision and hip protectors were considered and an urgent multidisciplinary discussion regarding the patient's individual approach took place. |
| Who provided | The ward staff delivered the intervention.  T**ypes of healthcare practitioners delivering the intervention:**  One type of healthcare practitioners: Nurses. |
| How | - |
| Where | The intervention was delivered in general geriatric rehabilitation wards at the Fliman Rehabilitation Geriatric Hospital, a 150-bed public geriatric facility affiliated with the Technion-University Medical School and located in Haifa, Israel. Two to three wards were included in each cluster, and there were |
| When and how much | - |
| Tailoring | - |
| Modificaction | - |
| How well: Planned | - |
| How well: Actual | - |
| **TIDieR control intervention description** | |
| Brief name | Usual care |
| Why | - |
| What: Materials | - |
| What: Procedures | Control wards had no trial interventions. "Participants in the control group continued to receive usual care which consists of any activities undertaken by the participants recommended or administered by their treating team and did not receive any of the interventions from the falls prevention program" (cited in journal article). |
| Who provided | Ward staff. |
| How | - |
| Where | General geriatric rehabilitation wards at the Fliman Rehabilitation Geriatric Hospital, a 150-bed public geriatric facility affiliated with the Technion-University Medical School and located in Haifa, Israel. |
| When and how much | - |
| Tailoring | - |
| Modification | - |
| How well: Planned | - |
| How well: Actual | - |

Barker et al. (2016)

| Funding source | Public sector: This project was funded by the National Health and Medical Research Council (NHMRC), Australia (APP1007627). ALB’s salary was funded by a career development fellowship from the NHMRC (APP1067236). RTM’s salary was supported by a postgraduate scholarship from the NHMRC (APP1055604). TPH’s salary was funded by a career development fellowship from the NHMRC (APP1069758). CS’s salary was funded by a career development fellowship from the NHMRC (APP632929). |
| --- | --- |
| Type of intervention | Fall risk assessment and interventions based on CDS. |
| iCAT_SR domain 2: Active components included in the intervention, in relation to the comparison | **Judgement:**  More than one component and delivered as a bundle.  **Support for judgement:**  Fall risk assessment preceded delivery of the 6-PACK intervention components; therefore bundle as opposed to package. |
| Notes |  |
| **TIDieR experimental intervention description** | |
| Where loacted | Journal article and Appendix 1: Intervention description (as per CONSORT extension Template for Intervention Description and Replication guidelines) |
| Brief name | The nurse-led 6-PACK program for reducing falls and fall related injuries in acute hospitals. |
| Why | There is limited high-level evidence to support the effectiveness of multifactorial falls prevention programs in the acute hospital setting. This RCT aimed to provide robust information on the effectiveness of a targeted, multifactorial, nurse-led falls prevention program. Best practice guidelines recommend the use of targeted multifactorial programs. Nurse-led program as nurses are the primary care providers for hospital patients so are optimally positioned to implement falls prevention activities. Having a single discipline responsible for the primary delivery of a falls prevention program may improve accountability for implementation. The program components were added to the existing patient care plan to ensure the program activities were integrated into nurse workflow. |
| What: Materials | The 6-PACK program includes a nine-item fall-risk tool (TNH-STRATIFY) and options for six interventions: 'falls alert' sign; supervision of patients in the bathroom; ensuring patient's walking aids are within reach; establishment of a toileting regime; use of a low-low bed and use of bed/chair alarm. Hospitals provided intervention wards with 'falls alert' signs, low-low beds (a minimum of 1 low-low to 3 standard beds on medical wards and 1 low-low to 10 standard beds on surgical wards), and bed/chair alarms (three on medical wards and one on surgical wards). A low-low bed was defined as a standard hospital bed that could be lowered to 250mm from the floor or lower. Risk scores and interventions applied to patients were recorded on a 6-PACK patient care plan and reviewed and updated each shift. |
| What: Procedures | The 6-PACK program was implemented by nurses on the intervention wards. A hospital-nominated site clinical leader and ward-nominated champions oversaw the implementation of the 6-PACK program on intervention wards. Champions and site clinical leaders met regularly to discuss the assimilation of the 6-PACK on their ward, undertake 'ward walk-rounds' that included practice audits and reminders, and review fall and implementation data provided by the research team. Site clinical leaders were supported by a change management and program facilitator employed by the project via telephone, email and site visits on a needs basis. Monthly meetings with the site clinical leaders from each hospital, change management and program facilitators and the research lead also occured. |
| Who provided | Site clinical leaders were registered nurses with varying levels of experience in education and falls prevention. They participated in a one day training session delivered by the change management and program facilitator, and the research lead. Site clinical leaders trained the champions and intervention ward nurses using a standardised 6-PACK implementation guide. Ward nurses included registered nurses (bachelor degree qualified) and assistants in nursing (technical college training). The change management facilitator was a health professional experienced in health service evaluation, project management and communication. The program facilitator was a registered nurse who designed, implemented and maintained the 6-PACK program at The Northern Hospital over a 10 year period prior to commencement of this study.  T**ypes of healthcare practitioners delivering the intervention:** Three or more types of healthcare practitioners delivered the intervention. Ward nurses, site clinical leaders, champions. |
| How | The 6-PACK program was integrated into daily care activities and delivered to individual patients by their treating nurse without provision of additional staffing resources. |
| Where | The 6-PACK program was delivered on the 12 participating intervention acute wards, in six public hospitals. Hospital 1 was a regional teaching hospital with 221 acute beds, hospital 2 was a metropolitan University teaching hospital with 420 beds, hospitals 3, 5 and 6 were metropolitan teaching hospitals of moderate size (200-500 beds) and hospital 4 was a metropolitan University teaching and major referral centre (>500 beds). |
| When and how much | The 6-PACK program was delivered over the 12-month trial period. The patients' treating nurse completed/updated the fall-risk tool for each patient each shift. For patients classified as high risk, nurses were required to select, record and apply a 'falls alert' sign and a minimum of one additional 6-PACK intervention as they deemed appropriate during the patient ward admission. |
| Tailoring | The 6-PACK interventions applied to each patient were personalized based on the fall-risk tool findings and clinical judgement of their treating nurse. |
| Modificaction | Due to local hospital policies and equipment purchasing procedures different models of low-low beds and bed/chair alarms were used across intervention wards. |
| How well: Planned | Intervention adherence was assessed via daily medical record audit and structured bedside observation by project-employed data collectors for all patients admitted to the intervention wards during the study period. A report of these data, including implementation league tables that ranked wards' level of adherence to the 6-PACK program components, were provided to the site clinical leaders each month during the RCT. The site clinical leaders were expected to communicate this information to the champions, ward nurses and ward Nurse Unit Manager. |
| How well: Actual | "No major protocol deviations or unexpected adverse events occured during the study period." "Use of 6-PACK programme components increased rapidly on the intervention wards after introduction of the programme at the start of the randomised trial period. No change was observed on the control wards, suggesting that contamination was unlikely." |
| **TIDieR control intervention description** | |
| Brief name | Usual fall prevention practices as part of existing hospital policy |
| Why | - |
| What: Materials | Fall prevention interventions provided to patients in control wards may have included some components of the 6-PACK programme and other interventions such as non-slip socks, constant patient observers, and falls alert wrist bands. |
| What: Procedures | "Control wards were asked to continue with usual care throughout the trial period. usual care involved falls prevention practices provided by wards as part of existing hospital policy, which may have included some components of the 6-PACK programme and other interventions such as non-slip socks, constant patient observers, and falls alert wrist bands" (cited in Barker et al. 2016). |
| Who provided | - |
| How | - |
| Where | - |
| When and how much | - |
| Tailoring | - |
| Modification | - |
| How well: Planned | - |
| How well: Actual | - |

Bhasin et al. (2020) and Ganz et al. (2022)

| Funding source | Bhasin et al. (2020): Public sector: Supported by the Patient-Centered Outcomes Research Institute and the National Institute on Aging of the National Institutes of Health (NIH) through a cooperative agreement (5U01AG048270) between the National Institute on Aging and Brigham and Women’s Hospital.  Ganz et al. (2022): Public sector: Supported by the Patient-Centered Outcomes Research Institute and the National Institute on Aging of the National Institutes of Health (NIH) through a cooperative agreement (5U01AG048270) between the National Institute on Aging and Brigham and Women's Hospital. Erich J. Greene, Denise Esserman, James Dziura, and Peter Peduzzi were also supported by a CTSA grant (UL1TR000142) from the National Center for AdvancingTranslational Sciences (NCATS), a component of the NIH. Siobhan K. McMahon was also supported by grants (KL2TR000113 and UL1TR000114) from the University of Minnesota Clinical and Translational Science Institute, funded by NCATS. Shalender Bhasin was supported in part by the Boston Claude D. Pepper Older Americans Independence Center (P30AG031679). Thomas M. Gill and Katy Araujo were also supported by the Yale Claude D. Pepper Older Americans Independence Center (P30AG021342). Neil B. Alexander was also supported in part by the Michigan Claude D. Pepper Older Americans Independence Center (P30 AG024824). Susan L. Greenspan and Neil M. Resnick were also supported in part by the Pittsburgh Claude D. Pepper Older Americans Independence Center (P30AG024827). Elena Volpi was supported in part by the UTMB Claude D. Pepper Older Americans Independence Center (P30AG024832). |
| --- | --- |
| Type of intervention | Fall risk assessment and interventions based on CDS. |
| iCAT_SR domain 2: Active components included in the intervention, in relation to the comparison | **Judgement:**  More than one component and delivered as a bundle.  **Support for judgement:**  Fall risk assessment preceded the individualised fall prevention program; therefore bundle as opposed to package. |
| Notes |  |
| **TIDieR experimental intervention description** | |
| Where located: | Primary paper and Reuben et al. (2017). The Strategies to Reduce Injuries and Develop Confidence in Elders Intervention꞉ Falls Risk Factor Assessment and Management, Patient Engagement, and Nurse Co-management. |
| Brief name | The Strategies to Reduce Injuries and Develop Confidence in Elders (STRIDE) intervention |
| Why | "The STRIDE intervention consists of specific care processes designed to reduce patients' fall risks, as well as two methods of ensuring that these processes are followed for each at-risk patient. The care processes include an assessment of the patient's specific risks of falling, followed by evidence-based interventions steps that are tailored to the patient's individual risk factors and preferences" (cited in Reuben et al. 2017). |
| What: Materials | Standardized and structured assessment procedures and algorithms. Used to conduct risk assessment and generation of recommendations. Falls care management software. Was created and used to facilitate risk calculation, note generation, tracking of care, and implementation of recommendations. |
| What: Procedures | STRIDE consists of five major components: 1. Risk assessment and generation of recommendations This is done by the falls care manager using standardised, structured assessment procedures and algorithms. "Before contacting each high-risk patient, the FCM reviews the patient's electronic medical record for information related to falls risk factors, including prior bone mineral density testing, cognitive function and medications related to falling and osteoporosis" (cited in Reuben et al. 2017). "The FCM reviews the circumstances of any falls and data that will be needed to calculate the patient's risk of future fractures (FRAX score)" (cited in Reuben et al. 2017). "The FCM's goal for the patient's initial visit is to complete a comprehensive falls risk assessment and to draft with the patient (and caregiver, when appropriate) a Falls Care Plan. The history clarifies or expands on PVQ topics and current medications, and the physical exam includes postural vital signs, a Snellen vision screen (when indicated), a modified short physical performance battery (mSPPBS) that was modified for this study to accommodate the time constraints of clinical practice, cognitive screening using the Mini-Cog, and inspection of the patient's feet and footwear" (cited in Reuben et al. 2017). Eight risk factors for falling are evaluated: medicaitons; strength, gait and balance impairment; postural hypotension; feet and footwear; home safety; osteoporosis; vitamin D; and visual impairment. 2. Explanation of identified risks by the FCM The FCM explains to the patient (and caregiver, when appropriate) and suggests interventions using motivational interviewing to elicit patient preferences and readiness to participate in treatments. 3. Co-creation (FCM and patient) of an individualised Falls Care Plan The individualised falls care plan considers risk-specific treatment algorithms, personal preferences, and available resources. "After the initial visit, the FCM completes a comprehensive note and communicates the initial Falls Care Plan electronically to the PCP and other health care team members including the pharmacist, if fall risk increasing drugs (FRIDs) are identified. STRIDE recommended interventions are communicated using standardized SBAR templates through the EHR. Within several days, the PCP sends edits to and/or approval of the Falls Care Plan to the FCM - and then FCM telephones the patient to discuss these recommendations and plan appropriate follow-up" (cited in Reuben et al. 2017). 4. Implementation of the Falls Care Plan The patient, FCM, PCP and other providers work to implement the falls care plan. Interventions within a registered nurse's scope of practice such as recommendations for safe footwear and instructions on simple home exercises are managed by FCMs and patients. Interventions outside a registered nurse's scope of practice, such as treatment of osteoporosis, are communicated to relevant providers using the Situation, Background, Assessment and Recommendation (SBAR) template. Interventions that require specialised skills or programs are referred, e.g. to outpatient physical therapy or community-based exercise programs. 5. Longitudinal follow-up Longitudinal follow-up includes in-person visits at least annually and phone calls at least once during the first year. The follow-up is done to reassess the patient's falls risk factors and to evaluate the implementation and the effects of the Falls Care Plan. The calls and visits determine whether the patient had difficulty implementing the specific prioritised actions in the Care Plan and, if so, what assistance could be provided or modifications could be made. The FCMs also inquired about whether the patient's goals and expectations were met, as outlined in the Care Plan (e.g. better balance, improved function). The FCMs did not collect outcome data, which were collected by the Data Coordination Center. "The six-month follow-up visit focuses on Care Plan evaluation during a scheduled telephone visit unless patients have had challenges implementing their Care Plan; had falls; or had changes in their medical, functional, or mobility status. Annual visits also include re-assessments of all fall risks, as well as Care Plan evaluations and revisions, as needed" (cited in Reuben et al. 2017). |
| Who provided | Falls Care Managers (FCMs) FCMs are Bachelor of Science in Nursing (BSN)-prepared Registered Nurses (RNs) who completed a comprehensive STRIDE training course. The training course includes 26 online modules with narrated presentations, formative assessment, and written resources. The RNs participated in a face-to-face group session designed to facilitate simulation and practice delivering all intervention components. Continuing education is offered throughout the study via annual face-to-face group sessions and monthly conference calls. FCMs practice within a co-management model with PCPs and their health care teams. FCMs are supervised by a site clinical director (SCD), which is a physician, nurse practitioner, or pharmacist with expertise in falls and knowledge regarding the local healthcare system. To ensure cross-site standardisation, FCMs participated in weekly conference calls with a nurse scientist who has expertise in advanced practice nursing in gerontology.  T**ypes of healthcare practitioners delivering the intervention:** Three or more types of healthcare practitioners. Nurses, primary care physician, pharmacist. |
| How | The intervention was delivered face-to-face in the primary care clinics during patient visits. The intervention was provided individually, except for group exercise which some participants may have received. |
| Where | The 10 clinical sites, representing 10 health care systems, at which the trial was conducted included rural and urban locations and involved 15 individual reimbursement plans. 86 out of 162 practices within the participating health care systems were selected based on prespecified criteria that included the size of the practice, the ability to implement the intervention, the geographic proximity of the practice to other practices, the accessibility of electronic health records, and access to community-based exercise programs. |
| When and how much | Follow-up in conducted according to the patients' individualized care plans, but at a minimum includes formal visits six months following the initial assessment and then annually. |
| Tailoring | The nurses implemented the intervention in partnership with the participants and their primary care providers. |
| Modificaction | - |
| How well: Planned | The intervention development and implementation were guided by a Patient and Stakeholder Council that reviewed and revised procedures, algorithms and patient education materials. The Council is composed of 21 members including 4 patients and 1 caregiver as well as community stakeholder representatives, and national fall prevention experts. "Two methods used to ensure implementation are: 1) "practice redesign," based on the ACOVE-2 approach and 2 risk factor assessment and interventions to engage patients and caregivers, based on the Connecticut Collaboration for Fall Prevention" (cited in Reuben et al. 2017). |
| How well: Actual | - |
| **TIDieR control intervention description** | |
| Brief name | Enhanced usual care (usual care plus a falls information pamphlet / booklet |
| Why | - |
| What: Materials | A webinar about preventing falls was made available to primary care providers in both study groups. Participants in the control group received an informational pamphlet about falls that was created by the Centers for Disease Control and Prevention and were encouraged to discuss fall prevention with their primary care provider. |
| What: Procedures | Participants in the control intervention received enhanced usual care which included a falls informational booklet which is part of the Stopping Elderly Accidents, DEaths and Injuries (STEADI) toolkit, titled "Stay Independent". Patients were encouraged to discuss fall prevention with their PCP at their next clinic visit. Participants' physicians received the results of the screening questions and were referred to a training webinar about fall prevention adapted from the STEADI toolkit. |
| Who provided | Registered nurses, primary care physicians. |
| How | - |
| Where | A total of 86 primary practices in 10 health systems across the United States of America. |
| When and how much | - |
| Tailoring | - |
| Modification | - |
| How well: Planned | - |
| How well: Actual | - |

Blalock et al. (2020)

| Funding source | Public sector: This work was supported by Cooperative Agreement Number 1 U01 CE002769-01 from the Centers for Disease Control and Prevention and grant number 1C1CMS331338 from the Department of Health and Human Services, Centers for Medicare & Medicaid Services. |
| --- | --- |
| Type of intervention | Medication review and recommendations made to physician. |
| iCAT_SR domain 2: Active components included in the intervention, in relation to the comparison | **Judgement:**  More than one component and delivered as a bundle.  **Support for judgement:**  Initial fall risk assessment preceded medication review, which preceded faxing the medication recommendations to the patient’s healthcare provider. There was a clear order; therefore bundle as opposed to a package. |
| Notes |  |
| **TIDieR experimental intervention description** | |
| Where located: | Primary paper. |
| Brief name | STEADI-Rx |
| Why | Many older adult falls could be prevented if those at greatest risk could be identified and evidence-based prevention measures were implemeted to remediate modifiable risk factors. Current guidelines recommend that healthcare providers ask all older adults about falls and gait/balance difficulties annually. The United States Centers for Disease Control and Prevention (CDC) developed the Stopping Elderly Accidents, Deaths and Injuries (STEADI) screening algorithm and toolkit to help integrate fall prevention guidelines in practice. The STEADI algorithm includes standardised screening questions to ask patients to assess fall risk and recommendations for follow-up tailored to patients' responses. However, reducing the use of high-risk medications has proven challenging, and there is a need for innovative approaches to reduce older adult exposure to medications that increase fall risk. To address this need, the team developed STEADI-Rx by adapting the STEADI algorithm and toolkit for use in the community pharmacy setting. Community pharmacies are an indeal setting in which to screen patients for medication-related fall risk, and pharamcists are uniquely qualified to address medication management issues. Pharmacists also have access to patients' prescription records that they can use to identify medications patients are taking that may increase their risk of falling. |
| What: Materials | The intervention included the following materials: - Spreadsheet listing the names of patients served by the pharmacy who met study inclusion criteria. - Evidence-based algorithms developed to identify medications associated with an increased risk of falling and provide recommendations to reduce risk. - Brochures containing information about fall prevention handed out to patients. - Forms developed for the purpose of sending medication recommendations to the patient's healthcare provider(s) as well as including a recommendation that the patient receive a gait, balance, and strength evaluation. |
| What: Procedures | Each intervention pharmacy received a spreadsheet listing the names of patients served by the pharmacy who met study inclusion criteria. Pharmacy staff screened patients by asking the following key STEADI questions: 1) Have you fallen in the past year? 2) Do you feel unsteady when standing or walking? 3) Do you worry about falling? Patients who reported hvaing fallen in the past year were also asked how many times they had fallen and whether any of the falls had resulted in injury. During the screening procedures participants also received patient education brochures about fall prevention developed by the CDC as part of STEADI. Patients who answered "yes" to any of the key STEADI questions were classified as having screened positive for increased fall risk and were eligible to receive a medication review by a pharmacist associated with the pharmacy that the participant obtained their medications. As part of the medication review, the pharmacist evaluated the patient's medication regimen using evidence-based algorithms developed to identify medications associated with an increased risk of falling and provide recommendations to reduce risk. After conducting a medication review, the pharmacist faxed recommendations to the patient's healthcare provider(s) using forms developed for this purpose. In addition to the patient-specific medication recommendations, the forms included a recommendation that the patient receive a gait, balance and strength evaluation, per the STEADI algorithm. Intervention pharmacies were not compensated for screening participants, but they received $80 for each medication review completed with documentation of provider follow-up. |
| Who provided | Pharmacists Pharmacists associated with the pharmacy where the patient obtained their medications performed the fall risk screening and the medication review, and used the form to send medication recommendations to the patient's healthcare provider(s). Patients’ primary care physicians.  T**ypes of healthcare practitioners delivering the intervention:** Two types of healthcare practitioners. Pharmacist and primary care physician. |
| How | The fall risk screening and medication review was conducted at the local community pharmacy. |
| Where | A total of 100 community pharmacies in North Carolina (NC) were invited to participate in the study based on their adherence to intervention protocols in previous initiatives. Of these, 65 (30 independently owned pharmacies, 33 pharmacies within eight small chains, and 2 pharmacies in a large grocery chain) agreed to participate); 34 were randomized to the no-treatment control group and 31 to the intervention group. The slight imbalance between the two group occured because three pharmacists performed medication reviews at two to three participating pharmacies. In these cases, pharmacies served by the same pharmacist were randomised together. Community pharmacies North Carolina (NC). The trial was nested within a larger effort sponsored by the Centers for Medicare and Medicaid Innovation to organise and encourage community pharmacies to provide enhanced services (e.g. medication management) to Medicaid and Medicare recipients in North Carolina (NC). |
| When and how much | The STEADI-Rx intervention was provided to individuals who met the following inclusion criteria: 1) age 65 years or older; 2) filled at least 80% of their prescriptions at a participating pharmacy; and 3) used either four or more chronic medications or one or more medications associated with an increased risk of falling. |
| Tailoring | - |
| Modificaction | - |
| How well: Planned | - |
| How well: Actual | - |
| **TIDieR control intervention description** | |
| Brief name | No-treatment control |
| Why | - |
| What: Materials | - |
| What: Procedures | Pharmacies randomised to the no-treatment control group did not receive any STEADI materials. |
| Who provided | - |
| How | - |
| Where | - |
| When and how much | - |
| Tailoring | - |
| Modification | - |
| How well: Planned | - |
| How well: Actual | - |

Blum et al. (2021)

| Funding source | Public sector: This work is part of the project OPERAM: OPtimising thERapy to prevent Avoidable hospital admissions in the Multimorbid elderly supported by the European Union’s Horizon 2020 research and innovation programme under grant agreement No 634238, and by the Swiss State Secretariat for Education, Research, and Innovation (SERI) under contract number 15.0137. |
| --- | --- |
| Type of intervention | Medication review and recommendations made to physician. |
|  |  |
| iCAT_SR domain 2: Active components included in the intervention, in relation to the comparison | **Judgement:**  More than one component and delivered as a bundle.  **Support for judgement:**  First, newly admitted patients were screened using the SHiM questionnaire. Next, medication review was performed jointly by a research physician and pharmacist. Third, the report was discussed with the attending hospital physician. Lastly, a final report was sent to the patient’s GP. There was a clear order; therefore bundle as opposed to a package. |
| Notes |  |
| **TIDieR experimental intervention description** | |
| Where located: | Primary paper and supplement to primary paper. |
| Brief name | Structured pharmacotherapy optimisation intervention supported by a software-based clinical decision support tool. |
| Why | As much as 30% of admission to hospitals in older adults are related to drugs. Of these, half may potentially be prevented. Various intervention have been designed to optimise pharmacotherapy in people with polypharmacy, with the aim of lowering the risk of adverse drug reactions. Most of these interventions consist of multifaceted strategies delivered by pharmacists. More recently, software systems have been developed to support drug appropriateness. The systematic tool to reduce inappropriate prescribing (STRIP) facilitated by the web based Systematic Tool to Reduce Inappropriate Prescribing (STRIP) Assistant (STRIPA) combines the screening tool of older person's prescriptions and screening tool to alert to the right treatment (STOPP/START) criteria with a more global evaluation of drug appropriateness and shared decision making with the patient. A structured pharmacotherapy optimisation intervention supported by a software-based clinical decision support tool is thought to improve clinical outcomes such as drug-related hospital admissions, falls, and other clinical outcomes, in older adults with multimorbidity and polypharamacy, compared with usual care.  The intervention was designed to identify the most relevant drug-related problems and optimize treatment during the hospitalisation. |
| What: Materials | Pharmacotherapeutic assessment is based on the START/STOPP criteria version 2, with 114 criteria. |
| What: Procedures | Newly admitted patients were screened, usually on the day of admission to the inpatient ward. First, pre-admission medication was assessed using the SHiM questionnaire, with the patients or their proxies. In addition to the screening, at least one other source of information was used (pharmacy, general practitioner) to improve the accuracy of the medication list. Second, a research physician and pharmacist jointly performed the medication review using the STRIP method. The pharmaceutical analysis was performed using the web-based STRIP Assistant (STRIPA), a decision support system. A first report with prescribing recommendations was generated via the software based on recommendations from STRIP and physician and pharmacist expertise. Third, the report was discussed with the attending hospital physician to reach agreement about the recommendations. Patients or their proxies were engaged in the decision-making process. Final medication changes were agreed upon by the researchers, treating hospital physicians, and the patient. Lastly, a final report was sent to the patient's GP to inform about in-hospital medication changes and all recommendations, including those that could not be implemented during the index hospitalization. Consideration of in-hospital clinical information (e.g. new diagnoses, adverse drug reactions) were included in the final report. Evidence-based reasons for change were provided with all recommendations. |
| Who provided | A research physician and pharmacist.  T**ypes of healthcare practitioners delivering the intervention:** Three types of healthcare practitioners. Pharmacist, hospital physician, and general practitioner. |
| How | The structured parmacotherapy optimisation intervention was performed at the individual level jointly by a doctor and a pharmacist, with the support of a clinical decision software system deploying the screening tool of older person's prescriptions and screening tool to alert to the right treatment (STOPP/START) criteria to identify potentially inappropriate prescribing. |
| Where | 110 cluters of inpatient wards in university-based hospitals in Switzerland, Netherlands, Belgium, and Republic of Ireland. |
| When and how much | The multi-component intervention used in the OPERAM trial consisted of several steps and started with reviewing medications of newly admitted patients. The intervention was delivered once per admitted patient. For more, see 'What: Procedures'. |
| Tailoring | See 'What: Procedures'. |
| Modificaction | - |
| How well: Planned | The research physician and pharmacist were trained in performing the medication review using the STRIP method. The research team was trained to each step of the intervention and standard operating procedures supported the process. |
| How well: Actual | - |
| **TIDieR control intervention description** | |
| Brief name | Usual care including unstructured medication review unsupported by STOPP/START criteria or the systematic tool to reduce inappropriate prescribing (STRIP) tool. |
| Why | - |
| What: Materials | - |
| What: Procedures | Usual care could include unstructured drug review by the attending hospital doctors, which was not specifically encouraged or discussed. Usual care was performed according to site specific standards of care that did not include application of STOPP/START criteria or STRIP. A sham intervention was administered to all participants through completion of the Morisky medication adherence measure questionnaire (MMAS-8). The purpose of the sham intervention was to mimic the intervention for purposes of blinding of the participants and team members. |
| Who provided | Hospital doctors. |
| How | - |
| Where | 110 cluters of inpatient wards in university-based hospitals in Switzerland, Netherlands, Belgium, and Republic of Ireland. |
| When and how much | - |
| Tailoring | - |
| Modification | - |
| How well: Planned | - |
| How well: Actual | - |

Byrne (2005)

| Funding source | Public sector: The Foundation for Long Term CAre (FLTC) received funding from the DHHS Agency for Healthcare Research and Quality (AHRQ) patient safety initiative to conduct this research. |
| --- | --- |
| Type of intervention | Automatically generated fall risk based on prediction models followed by recommended interventions. |
| iCAT_SR domain 2: Active components included in the intervention, in relation to the comparison | **Judgement:**  More than one component and delivered as a bundle.  **Support for judgement:**  First, clinical risk reports were developed. This was preceded by integrating the information from the risk reports into practice. There was a clear order; therefore bundle as opposed to a package. |
| Notes |  |
| **TIDieR experimental intervention description** | |
| Where located: | Thesis paper. |
| Brief name | Application and integration of computerized resident risk information decision tools into care processes. |
| Why | The administration of fall assessment tools is more often done manually, and “time consuming and often inconvenient” to nursing home staff (Committee on Quality of Health Care in America, 2001; Eagle et al., 1999). However, since much of the risk data is now already being collected electronically via the MDS, the identification of high-risk individuals could be a simpler process, especially with the aid of electronic technology. In today’s nursing home environment of shrinking finances, staffing shortages (New York State Association of Homes and Services for the Aging, 2001), high turnover, increasing workloads and growing acuity levels (B.J & et al., 2001; Shaefer & Moos, 1996), nursing staff cannot gather and analyze all of the resident data needed to accurately assess risk for adverse outcomes without the aid of computerized decision support tools. This project translates research to practice through prospective Internet-based risk reports that synthesize knowledge derived from current research, up-to-date standards of care, successful protocols and best practices; and 1) apply it to existing assessment data collected by nursing homes on all of their residents, called the Minimum Data Set (MDS); and 2) display key information in useful and readily accessible formats that can be acted on to avoid adverse outcomes and improve systems of care. The intervention which is the focus of this evaluation study was based on the assumption that nursing home staff would: 1) access the resident risk information; 2) understand the information and how it could be used to improve care processes; 3) share the information with key staff who could design effective interventions/approaches; and 4) implement the intervention(s) as planned. |
| What: Materials | The Minimum Data Set (MDS): - Used as a risk assessment tool to develop the risk prediction models independent of this dissertation research project. - The MDS has over 500 items and is the source of Quality Indicators (QIs) used by facilities and surveyors in evaluating care in nursing homes. QIs are clinical markers that indicate either the presence or absence of potentially poor nursing home practices. - The MDS data is based on MDS assessments submitted for each resident and each record consists of an assessment, which could be for an admission, a quarterly assessment, an annual assessment, a significant change, a correction, a re-entry, or a discharge. - The new risk assessment and care planning information and reports provided to nursing home staff through this study is based on the standardized Minimum Data Set (MDS) assessment data. The MDS is a uniform set of elements extracted from the Resident Assessment Instrument, a standardized tool for assessing functional status of nursing home residents. Automated transmission of MDS data began in 1998, and all facilities must complete and transmit MDS data every quarter for all residents to their state health agency (clearinghouses) as a condition of Medicare and Medicaid. MDS submission software conforms to careful edits to ensure that each assessment is correctly completed. The data are then transmitted by the state “clearinghouses” to CMS, where they are used for a variety of purposes, such as setting reimbursement levels and rates, and generating quality of care measures for each nursing home. The MDS is regularly audited and reviewed, and is considered to be a high-quality data system and is frequently used for LTC research. Risk reports generated from the MDS: - Three primary types of risk reports were devloped and made available for immediate display and printing at the users site via a secure Internet connection: 1. At Risk Reports: list residents, by level of risk for a fall or pressure ulcer. 2. Risk Profile Reports: display and rank resident-specific risk factors for at risk residents allowing nursing staff to make individualised care decisions based on data-driven, knowledgebased and resident-focused information. 3. Feedback Reports Prediction models: - MDS data was used to develop risk models based on logistic regressions to predict a future fall and pressure ulcer. - Five risk levels were developed based on the distribution of the probability estimates: Very high; high; Moderate; Low; and lowest. The MDS is also the basis for 24 Quality Indicators (Qls), selected by CMS in 1999 for use by facilities and surveyors in evaluating nursing home care. The Qls are clinical markers for potentially poor nursing home practices. They include quality of care areas such as falls, pressure ulcers, behavior problems, loss of basic functional activities, and incontinence. Fall and pressure ulcer risk assessment tools: Web-application that captures the number of 'visits' to the risk reports since the project began to help monitor use of the reports and differentiate level of use and application of the risk reports: - All participating nursing homes were given access to the web-based application. The risk reports were made available through existing nuring home quality improvement software called EQuIP for Quality (EQuIP). EQuIP is based on the same MDS data that nursing homes are required to submit to state clearinghouses for processing. Nursing homes collect the resident assessment data within 2 weeks of admission and on at least a quarterly basis after admission. The MDS information is typically manually entered into MDS ssoftware that may be integrated with other clinical software in the nursing home. Nursing homes then use the software to submit the files electronically to the Clearinghouses. They then typically submit the same files to their Quality Improvement software vendors such as EQuIP so that they can obtain more timely access to their Quality Indicators, study the residents who flagged on these QIs, and obtain more useful information including benchmarks and charts on their resident population. The MDS data is, among other purposes, used to generate QI reports that state and federal surveyors can access, as well as the individual nursing homes. The EQuIP reports, including the risk reports, are availbale as soon as the nursing home staff upload their data via a user-friendly web application. Nursing home data from the New York State Health Facility Master File |
| What: Procedures | In summary, the intervention procedure was as follows: - MDS-based risk models were developed and validated (prior to this thesis) - Clinical risk reports were generated based on MDS-based models: The information was generated automatically using risk prediction models based on the MDS. - Nursing staff ran the risk reports Use of the risk reports was entirely voluntary on the part of the nursing home staff. - Nursing staff used, shared, and integrated the risk information into care planning processes - Nursing staff developed and carried out interventions to reduce outcomes based on reports. |
| Who provided | Nurses Two to three nursing staff from each participating facility were required to attend training seminars as a condition of participation in the study. The nurses usually were the director/assistant director of nursing, MDS coordinator, and/or the director of quality improvement, and sometimes the nursing home administrator. Participant Training - The initial training sessions presented information required to understand and use the risk reports for quality improvement. The sessions covered: - How to access resident assessment information; - Basic statistical terminology and concepts; - Explanation of the risk models and risk factors displayed in reports; - How outcome rates are derived; - Interpreting risk and outcome data and using benchmarks; - Applying information to select the areas of care that are a problem; - Examples of how the risk reports could be used to target remediation and interventions; - How to develop and evaluate changes in care processes based on the information; and Introduction to planned new feedback reports and how to implement them. All facilities were offered a training video after a year of the project for any new staff that may have joined the project and were provided with documents that presented the project background. All of the 11 added replacement nursing homes were provided with these resources as well as telephone consultation to get up to speed on the project, as only a small number of staff took advantage of these resources.  **Types of healthcare practitioners delivering the intervention:** One type of healthcare practitioners: Nurses. |
| How | - |
| Where | Nursing homes in New York. Nursing home group 1 & 2 (34 homes; 36.5% of participants) were identified as using and integrating the risk reports. Group 1 nursing homes (n = 18; 20% ot total nursing homes) were characterized by high access and high integration with care planning. These homes regularly ran and used all of the risk reports (averaging 9 report page "visits" per month), used them prospectively (proactive care planning) rather than solely retrospectively (e.g. to help investigate causes of a fall), and rated them as very useful. The matched control group consisted of a matched sample of nursing homes that volunteered to participate in the project but were not selected (Group 5) or that did not volunteer (Group 6) since Group 5 did not yield high enough matches to use that group only. |
| When and how much | - |
| Tailoring | - |
| Modificaction | - |
| How well: Planned | "Each nursing home that had access to the risk reports was able to use the information in the ways that they felt would work best - there was no required set of interventions for reducing falls and pressure ulcers. It is not possible to evaluate how each facility implemented the risk reports for quality improvement programs... " During this study, three update seminars and regular conference calls were conducted to reinforce the participants’ use and application of the resident information for resident care planning, and to share lessons learned and knowledge gained (e.g. best practices). These sessions also provided an opportunity for the participants to provide feedback on the usefulness of the reports and any additional supporting materials. Nurse consultants were always available to provide assistance on use and interpretation and answer any questions about the reports. Nursing home staff were also given conslutations, and there was ongoing communication that aimed to education nursing staff on how to use the reports (see thesis page 64-65). Since many of the nursing homes took several months to get staff familiar with the reports, share them at meetings, promote their use for various purposes, it was decided to wait a full 15 months after project implementation before beginning the post-intervention evaluation. |
| How well: Actual | In the 34 "higher use" facilities (group 1 and 2), the risk reports were reportedly used in a wide variety of ways; most commonly to: - Serve as the primary risk assessment tool and for many replaced manual tool; - identify and notify other direct care staff which residents were at "high-risk"; - guide deveopment of care plans and interventions aimed at prevention; - ensure a comprehensive assessment of all potential resident risk factors; - educate and inform interdisciplinary team staff; nurse managers and certified nurse assistants; - support quality improvement; quality assurance; fall and skin care committee activities; - support responses to the survey team (e.g. help demonstrate an adverse event was "unavoidable") |
| **TIDieR control intervention description** | |
| Brief name | Usual care |
| Why | - |
| What: Materials | - |
| What: Procedures | The matched control group consisted of a matched sample of nursing homes that volunteered to participate in the project but were not selected (Group 5) or that did not volunteer (Group 6) since Group 5 did not yield high enough matches to use that group only. |
| Who provided | - |
| How | - |
| Where | The matched control group consisted of a matched sample of nursing homes that volunteered to participate in the project but were not selected (Group 5) or that did not volunteer (Group 6) since Group 5 did not yield high enough matches to use that group only. |
| When and how much | - |
| Tailoring | - |
| Modification | - |
| How well: Planned | - |
| How well: Actual | - |

Carroll, Dykes & Hurley (2012) and Dykes et al. (2010)

| Funding source | Research trust: This work was supported by a grant from the Robert Wood Johnson Foundation Interdisciplinary Nursing Quality Research Initiative (Translating Fall Risk Status Into Interventions to Prevent Patient Falls). |
| --- | --- |
| Type of intervention | Fall risk assessment and interventions based on CDS. |
| iCAT_SR domain 2: Active components included in the intervention, in relation to the comparison | **Judgement:**  More than one component and delivered as a bundle.  **Support for judgement:**  Fall risk assessment preceded tailoring of the fall prevention interventions by the FPTK software, which preceded delivery of the fall prevention interventions. There was a clear order; therefore bundle as opposed to a package. |
| Notes |  |
| **TIDieR experimental intervention description** | |
| Where located: | Primary paper |
| Brief name | Electronic Fall Prevention Tool Kit (FPTK) software using health information technology (HIT) |
| Why | - |
| What: Materials | Fall risk assessment. The Morse Fall Scale (MFS) was completed, see "How well: Planned" for more info on the MFS. The FPTK consisted of the following components, which all communicated patient-specific alerts to key stakeholders: Bed posters. Bed posters were composed of brief text with an accompanying icon. Patient education. Patients were handed Plans of care. Plans of care were documented in the EHR. |
| What: Procedures | Fall risk assessment. The MFS was completed using the FPTK. The FPTK software tailored evidence-based and feasible fall prevention interventions based on the results from the fall risk assessment with the MFS. Bed posters. Tailored bed posters automatically printed and were placed above the bed for all patients at risk of falling. The bed posters were updated with a change in fall risk status. Patient education. The patient and/or family members were educated using tailored handouts (automatically printed and updated with change in status). Plans of care. A tailored plan of care was automatically generated by the FPTK from the fall risk assessment. |
| Who provided | Hospital nurses assessed patients for their risk of falls and offered fall prevention interventions.  T**ypes of healthcare practitioners delivering the intervention:** One type of healthcare practitioners: Nurses. |
| How | The intervention was delivered face-to-face individually and sometimes with family members present. |
| Where | The intervention occured at four hospitals in the Partners HealthCare System in the Boston, Massachusetts, area. |
| When and how much | - |
| Tailoring | - |
| Modificaction | - |
| How well: Planned | The research team conducted 3 phases of study to develop and test components o fthe FPTK. In phase 1, they used qualitative inquiry to identify barriers and facilitators to fall risk communication and interventions. Participants reported that alerts related to patient-specific fall risk status and interventions to prevent falls were unavailable yet clearly needed at the bedside. "In phase 2, the study team developed the prototype FPTK by using the MFS risk factors as the foundation for the initial template. Decision rules and interventions were based on evidence from the literature and findings from phase 1 interviews. The MFS (scores range from 0-125) consists of 6 risk foci: (1) recent history of falling (25 points); (2) presence of secondary diagnosis (e.g., >1 medical diagnosis listed in patient record) (15 points); (3) need for ambulatory aid (0-30 points); (4) receiving intravenous therapy (20 points); (5) gait characteristics (0-20 points); and (6) impaired mental status (15 points). An illustrator was added to the team to develop icons to address the need for bedside alerts while simplifying and standardizing communication across stakeholders, including those with low literacy levels" (cited in journal article). In phase 3, we used an iterative process involving domain experts, end users, and an illustrator to identify valid icons for the FPTK. The 11 icons that received the highest ratings for congruence with the text of the fall risk and prevention plan were retained to populate the FPTK (eFigure 1; available at http://www.jama.com). The final FPTK included the MFS (Figure 1) that, when completed, automatically presented corresponding interventions tailored to patient-specific areas of risk. Once the recommended interventions were reviewed, tailored if needed, and approved, the FPTK printed a bed poster, a patient education handout, and a plan of care (eFigure 2)" (cited in journal article). To overcome the lack of effectiveness in previous randomised trials the research team used the Institute for Healthcare Improvement's Framework for Spread to promote unit-level buy-in. The FPTK included an adherence dashboard to facilitate monitoring. |
| How well: Actual | - |
| **TIDieR control intervention description** | |
| Brief name | Usual care |
| Why | - |
| What: Materials | - |
| What: Procedures | Hospital units that were part of the control group continued to provide usual care related to fall prevention. An educational program on fall risk assessment and prevention was used in the control units, which has been found to be effective in the short term but not over time. Fall risk assessment. The Morse Falls Scale (MFS) was completed using existing paper or electronic forms. Bed posters. Generic "high risk for falls" sign was placed above patient's bed for patients scoring >45 on the MFS. Patient education. The patient and/or the patient's family members were educated, and were provided booklets or other handouts as needed. Plans of care. The plan of care was documented manually in paper or electronic record. |
| Who provided | Hospital nurses. |
| How | The intervention was delivered face-to-face. |
| Where | The intervention occured at four hospitals in the Partners HealthCare System in the Boston, Massachusetts, area. |
| When and how much | - |
| Tailoring | - |
| Modification | - |
| How well: Planned | - |
| How well: Actual | - |

Clemson et al. (2024)

| Funding source | National Health and Medical Research Council (NHMRC) Partnership grant 1072790. |
| --- | --- |
| Type of intervention | Fall risk assessment and interventions based on CDS. |
| iCAT_SR domain 2: Active components included in the intervention, in relation to the comparison | **Judgement:**  More than one component and delivered as a bundle.  **Support for judgement:**  Intervention consisted of the stay independent questionnaire answered by the patient, followed by either fall prevention information and a community exercise referral, or multifactorial fall risk assessments and interventions. There was a clear order in the delivery. |
| Notes |  |
| **TIDieR experimental intervention description** | |
| Where located: | Primary paper and clinical trial regsitry. |
| Brief name | The Integrated Solutions for Sustainable Fall Prevention (iSOLVE) intervention. |
| Why | "It is estimated approximately a third of people over 60 years will have at least one fall per year, with more falls experienced by people over 75 years and some falling multiple times a year. [...] Falls are serious events with consequences of injury affecting mobility and independence, as well as psychological consequences such as loss of autonomy, loss of self-efficacy, and fear of falling, all of which would benefit from interventions through primary care. [...] Despite strong evidence to guide effective fall prevention interventions in community-residing older people, there are few models and no clear model in Australia for engaging GPs in fall prevention. Additionally, routine use of allied health professionals (AHPs) in fall prevention has been slow, limiting widespread dissemination. To address these gaps, we developed the Integrated Solutions for Sustainable Fall Prevention (iSOLVE) implementation project to establish and evaluate processes and pathways to identify at-risk older people and engage a whole primary care approach to fall prevention." (cited in primary paper; Clemson et al. 2024). Through the iSOLVE project, the researchers sought to engage GPs and AHPs in fall prevention, increase awareness of falls and fall prevention, and enable ongoing learning and sustainable actions aimed at preventing falls.  The resources developed for the intervention aimed to provide a simple workflow system for identifying people at risk of falls and to initiate fall prevention in GP practice. |
| What: Materials and procedures | During development of the intervention materials, the project group consulted widely with service coordinators and management at the primary care network (NSML and the PHN) and their advisory group (GP, consumer, physiotherapist, exercise physiologist, occupational therapist, pharmacist, podiatrist, nurse, and fall prevention champions). The project group further consulted with local GPs, geriatricians, a geriatrician-clinical pharmacologist, and an ambulatory care specialist. The developers of the STEADI primary care resources in the US shared their resources and their experience with the project group.  The following resources were adapted or developed for the iSOLVE project:  Decision support tool  The decision support tool was based on the STEADI algorithm and provided a workflow system for GP practice, with the GP first asking the questions, 'Have you had a fall?' and 'Are you worried about falling?' The tool included the Stay Independent 12-item self-reported checklist and the GP Fall Risk Assessment. The project group added another element to the decision tool, called "Tailoring Interventions to Fall Risk Factors", to map risk factors to appropriate interventions. The decision support tool further included referral lists to local fall prevention service providers.  A GP manual, which included summaries of fall prevention evidence, a series of case studies providing examples of using the Decision Tool and tailoring management options, Australian Government Medicare Benefits Schedule funding options, and examples of 'how to talk to you patients about falls'.  GP software. Both paper and electronic versions of the decision tool were offered to the GPs. The decision tool was developed within a commercial GP software (PenCS third-party software Topbar) that was used by some GPs in the area. A tablet was set up with the Stay Indepent checklist and was used by practice nurses or reception staff. Patients filled in answers using the tablet, and preliminary risk information was automatically sent to the GP software.  GP face-to-face educational detailing sessions.  The procedure for the decision support was as follows: The patient completed the stay independent checklist, which was then reviewed by the GP or practice nurse. If the patient answered no to all questions, then the patient should be offered fall prevention information and a referral to community exercise. If the patient answered yes or one or more risk factor, then the GP should conduct a fall risk assessment. The fall risk assessment consists of fall history and circumstances. Further, depending on what risk profile emerged, the assessment may include balance, strength, and gait; medication review; vision impairment/cataract; postural hypotension/dizziness/light-headedness; foot pain; urge incontinence; recent hospitalisation; and cognitive impairment. Next, the GP should provide fall prevention information and refer as needed to one or more of the following interventions: balance and lower limb strength training; home safety review; medication review; cataract surgery; fall prevention program; and a falls clinic. If present, other risk factors should be addressed and managed. Further, the decision support encourages follow-up of the patient by reviewing patient education, assessing and encouraging follow through with recommended interventions, discussing and addressing barriers or resistance to interventions, and an annual review of fall risk.  ---  Description of the control intervention from the clinical trial registry:  "1) An algorithm/decision tool is used to facilitate GPs in identifying, assessing and managing patients at risk of falling who are presenting to GP practices. To facilitate integration of the iSOLVE algorithm, face-to-face, one-hour academic detailing will be offered to GPs involved. Further, an electronic clinical decision support tool will be offered to GP practices involved. This electronic tool is developed as part of the iSOLVE project to integrate the processes into general practice systems and software to facilitate workflow.  The decision making tool is adapted from STEADi, a primary care resource for fall prevention developed by the Centres for Disease Control in the United States, using the American Geriatrics Society’s Clinical Practice Guidelines, and has been updated using the Cochrane Database on community fall prevention.  2) Identifying older people: People aged 65 years and over who have had a fall will be identified through auditing GP practice database for patients’ over 65 years and presentation to GP practice for an appointment.  3) Fall risk identification: The patients will be invited to complete the ‘Stay Independent’ brochure in the GP practice waiting room prior to their GP appointment. This will provide an initial screening of risk factors to alert the GP.  4) Fall risk assessment: Patients who have identified a risk factor as per the ‘Stay Independent’ brochure will have the risk factor assessed by a GP.  5) Fall risk management: The GP will tailor a management plan based on the patient’s risk factor by referring to the algorithm, clinical decision tool and referral pathway developed as part of the iSOLVE project. Depending on the algorithm this may trigger further assessment by the GP such as a medication review or hypotension or dizziness. The GP will initiate an appropriate referral, based on the algorithm, to local fall prevention services for example exercise professional for prescription of strength and balance exercise, occupational therapist for home safety intervention, pharmacy medication reviews, Stepping On fall prevention programs or cataract surgery. GPs are encouraged to follow up on subsequent usual visits or addition visits if needed at the discretion of the GP. The GPs will tailor the duration of each consultation as appropriate for the GP and the patient.  At the end of 12 months, the patients will be asked what fall prevention activities (e.g. exercise, home safety adaptations, medication review, cataract extraction) have been recommended by their GP and have been undertaken by them.  6) As part of the wider project, the iSOLVE team will engage with fall prevention service providers within the Northern Sydney Medicare Local (NSML) catchment area (study area) to facilitate referral pathways with GP practices. Education workshops will also be offered to fall prevention service providers in the study area." |
| Who provided | General practitioners (n =75) and allied health professionals (n = 342), of which 135 (40%) were physiotherapists and 82 (24%) were occupational therapists.  T**ypes of healthcare practitioners delivering the intervention:** More than two types of healthcare practitioner: Primary care physicians and allied health professionals (physiotherapists, occupational therapists, practice nurses, podiatrists, pharmacists, exercise physiologists, and other professions). |
| How | The intervention was delivered face-to-face in the primary care clinics. |
| Where | The study took place in 27 primary care practices part of the Primary Health Network (PHN) in Sydney, Australia, and included allied health professionals, most of whom were self-employed and worked in clinics and pharmacies in the local communities. |
| When and how much | - |
| Tailoring | The intervention was tailored to each individual participant in multiple ways. Depending on the patient's answers to the Stay Independent checklist, the patient was either offered fall prevention information and a referral to community exercise, or multifactorial fall risk assessment and interventions. The risk factors that were assessed depended on the emerging risk profile from the Stay Independent. The tailored interventions that were offered to the patient depended on the patient's risk factors. |
| Modificaction | - |
| How well: Planned and actual | Researchers conducted in-depth interviews with GPs in the experimental group (n = 24, 75%). The following six themes were identified: (i) making it easy to ask the iSOLVE questions, (ii) internalising the process, (iii) integrating the iSOLVE into routine practice, (iv) addressing assumptions about patients and fall prevention, (v) the degree of change in practice, and (vi) contextual issues influencing uptake.  In the final 2019 annual survey, a free-text question asked how GPs changed practice and the challenges they encountered. All 204 respondents commented on challenges to providing fall prevention, with the following themes:  "Time issues  Time challenges being a part time GP, health problems as a priority, time for fall assessments, logistics of longer appointments, follow-up,  and nurse appointments.  GP beliefs about patients  Patients viewed falls as being a normal part of ageing, and, along with observed sedentariness, impacted compliance and adherence.  Needing to convince patients about the benefits of fall prevention. Perceived their patients did not easily accept their fall risk, tended not to  report falls, and were fearful of embarrassment.  Service issues  Access to services. Physical access to face-to-face activities, private service costs, lack of publicly funded services, inadequacies of the MBS  chronic disease management funding schemes to subsidise AHP costs, waiting lists, and availability of AHP services for a referral. Transport  was an issue for patients, and GPs commented on the lack of continuity of care and available services.  Other issues  Need for integrated software support, falls not being classed as a medical diagnosis, and the need to focus on high-risk patients and the  challenges of patients with cognitive problems." (cited in the journal article, Clemson et al. 2024). |
| **TIDieR control intervention description** | |
| Brief name | Usual general practice care. |
| Why |  |
| What: Materials and procedures | "The control group will not be provided with education or information about falls prevention or with any of the iSOLVE decision tools." (cited in Clinical Trial registry).  "Control patients were provided with resources after 12 months (and after their GP received educational details) and asked to consult with their GP." (cited in journal article, Clemson et al. 2024). |
| Who provided | General practitioners and allied health professionals. |
| How |  |
| Where | The study took place in 27 primary care practices part of the Primary Health Network (PHN) in Sydney, Australia. |
| When and how much | - |
| Tailoring | - |
| Modification | - |
| How well: Planned and actual | - |

Dykes et al. (2020)

| Funding source | Public sector: This study was funded by grant #P30HS023535 from the Agency for Healthcare Research and Quality. |
| --- | --- |
| Type of intervention | Fall risk assessment and interventions based on CDS. |
| iCAT_SR domain 2: Active components included in the intervention, in relation to the comparison | **Judgement:**  More than one component and delivered as a bundle.  **Support for judgement:**  Fall risk assessment preceded linking of risk factors to appropriate interventions,m which preceded delivery of the interventions. There was a clear order; therefore bundle as opposed to a package. |
| Notes |  |
| **TIDieR experimental intervention description** | |
| Where located: | Primary paper and Dykes et al. (2020). Supplement 1. Trial Protocol. |
| Brief name | Fall TIPS (Tailoring Interventions for Patient Safety) Toolkit (FTTK) that actively engages patients and family in the three-step fall prevention process |
| Why | "In our previous work we have learned that preventing falls is a three step process: 1) identifying risk factors; 2) developing a tailored or personalised plan to decrease risk; and 3) consistently carrying out the plan. Our team designed a fall prevention toolkit that made it easy for professional and paraprofessional providers to consistently complete the three step fall prevention process. We found that our fall prevention toolkit reduced aptient falls by 22%. However, the literature suggests that 78% of falls are preventable. We hypothesize that to further reduce falls, we need to partner with patients and their family members so that the entire team can routinely participate in the prevention process" (cited in trial protocol). The toolkit is based on previous work from the authors and on a simple paper tool prototype that is based on a program they observed during a visit to Cathay Hospital in Taiwan where family caregivers are routinely involved in completing a fall risk assessment and in helping the patient to execute the plan during a hospitalisation. The fall rate in Cathay Hospital is <0.5 falls per 1000 patient days on medical units in contrast to US hospitals where fall rates are typically 3 - 4 falls per 1000 patient days on medical units. The intervention was developed with 1) problem analysing using workflow observations, individual and group interviews; 2) design using knowledge gained in phase 1 to design a patient-centered fall prevention toolkit; and 3) development using participatory design, rapid prototying, and computer modeling and simulation methods to construct the patient-centered fall prevention toolking. |
| What: Materials | The toolkit was integrated with the Epic Medical Record (EMR) system used in the hospital medical units. |
| What: Procedures | "An 11-by-17-inch laminated Fall TIPS poster was displayed at the bedside and used color-coded clinical decision support to link the Morse Fall Scale risk factors to evidence-based interventions. Nurses completed the poster with a dry-erase marker at admission and during each shift with the patient and family (if available) and posted it at the bedside. Using the Fall TIPS EHR-integrated tool-kit, nurses identified patient-specific risk factors using the Morse Fall Scale, and clinical decision support automatically linked each risk factor with the appropriate preventive interventions. Nurses could further tailor prevention plans based on their knowledge of the patient. Once completed, poster (8.5 x 11 in) detailing the risk factors and fall-prevention plan were generated and printed from the EHR system, hung at the bedside (sites 2 and 3), or automatically displayed on the bedside computer screensaver (e-bedside display, site 1) and reviewed with the patient and family at admission and during each shift" (cited in Dykes et al. 2020). |
| Who provided | Hospital nurses Study staff visited study units to provide training during the go-live week.  T**ypes of healthcare practitioners delivering the intervention:** One type of healthcare practitioners: Nurses. |
| How | The intervention was delivered face-to-face between the hospital nurse and the patient. If family members were present, these were also included in the intervention procedures. |
| Where | The intervention was delivered in 14 adult medical units in three academic medical centers (cite 1 in Boston, Massachusetts; cite 2 in Bronx, New York; and site 3 in New York, New York). |
| When and how much | Risk assessment and planning is performed upon admission, weekly, and whenever there is a change in the patient's status. The personalised fall prevention plan was updated with changes in the patient's status. |
| Tailoring | - |
| Modificaction | - |
| How well: Planned | "Study staff engaged leadership at institutional and care-unit levels through presentations on the evidence supporting Fall TIPS. We used a peer-champion model of existing unit-based nursing staff for education and training. Nurse champions who completed competency training were involved in continuous engagement of staff nurses, monitoring of fidelity, and reinforcement, with the intention of successful integration of the intervention into practice" (cited in Dykes et al. 2020 Supplement 1). "Unit-based nurse champions measured adherence to the protocol witih patient engagement audits consisting of 3 questions: (1) Is the Fall TIPS poster updated with the correct patient information? (2) Can the patient/family express their fall risk factors? (3) Can the patient/family express their fall-prevention plan? Based on continuous feedback from unit champions, barriers to adoption and spread were addressed. After the go-live date, nurse champions completed 5 random audits per month and provided peer feedback to the nurses caring for the audited patients" (cited in Dykes et al. 2020 Supplement 1). |
| How well: Actual | "One limitation is that support from hospital leadership and unit champions, communication channels, timing of implementation, and nurse and patient adherence to the protocol were variables that could not be fully controlled" (cited in Dykes et al. 2020). |
| **TIDieR control intervention description** | |
| Brief name | Usual care as it relates to fall prevention |
| Why | - |
| What: Materials | - |
| What: Procedures | The protocol for the control intervention is as follows: 1. Fall Risk Assessment and Planning Risk assessment and planning is performed upon admission, weekly, and whenever there is a change in the patient's status. Nurses complete the Morse Fall Scale (MFS) using existing paper or electronic forms. Thereafter nurses follow the unit or facility protocol for care planning. 2. Bedside Alert to all Stakeholders Nurses hang a generic 'High risk for falls' sign above the paitent's bed for patients scoring >45 points on MFS. 3. Patient Education The nurse educates the patient and family members, and provides booklets or other generic fall prevention handouts as needed. 4. Documentation of the Fall Prevention Plan The fall prevention plan should be documented in the medical record. |
| Who provided | Hospital nurses |
| How | The intervention was delivered face-to-face between the hospital nurse and the patient. If family members were present, these were also included in the intervention procedures. |
| Where | The intervention was delivered in 14 adult medical units in three academic medical centers (cite 1 in Boston, Massachusetts; cite 2 in Bronx, New York; and site 3 in New York, New York). |
| When and how much | - |
| Tailoring | - |
| Modification | - |
| How well: Planned | - |
| How well: Actual | - |

Elley et al. (2008)

| Funding source | Public sector: Funded by the New Zealand ACC, the New Zealand Lotteries Commission, the Wellington Medical Research Foundation, the University of Otago, and the Hutt Valley District Health Board. |
| --- | --- |
| Type of intervention | Fall risk assessment and interventions based on CDS. |
| iCAT_SR domain 2: Active components included in the intervention, in relation to the comparison | **Judgement:**  More than one component and delivered as a bundle.  **Support for judgement:**  The community-based health assessment of falls and fracture risk factors preceded delivery of the interventions and referrals. There was a clear order; therefore bundle as opposed to a package. |
| Notes |  |
| **TIDieR experimental intervention description** | |
| Where located: | Primary paper and Trial protocol. |
| Brief name | Falls-and-fracture nurse (FFN) coordinator and multifactorial intervention |
| Why | The intervention incorporates aspects from the successful PROFET trial, Tinetti's multifactorial intervention trial, and the individually tailored Otago Exercise Programme. |
| What: Materials | - |
| What: Procedures | The FFN instigated a referral to the regional occupational therapy service if a need for modification was detected using the standard home assessment. The intervention contained the following components: - A community-based health assessment of falls and fracture risk factors - Home hazards assessment - Bone health assessment - Referral to appropriate community fall prevention interventions - A strength and balance exercise programme Intervention participants receive a falls risk assessment by a community-based FFN coordinator in their own home usually within one month of enrolment. The health assessment includes history of circumstances of the fall, medications, previous cardiovascular or neurological illness, continence, vision, postural blood pressure, balance and gait, cardiovascular screen (syncope, arrhythmia). The home hazards assessment includes an audit for environmental safety. The bone health assessment includes a brief osteoporosis risk screen, recommendations for vitamin D and calcium supplementation, and DEXA scan and bisphosphonates where indicated. The exercise programme given was the Otago Exercise Programme. Exclusion criteria for the Otago exercise programme were a Timed Up and Go Test score longer than 30 seconds or marked neurological impairment. The FFN coordinator could refer those excluded to a community physical therapist who tailored an alternative exercise program. "The nurse provides appropriate advice, education and coordinated medical referral to the family physician, geriatrician, optometrist, physiotherapist, occupational therapist or other professional if indicated, according to the assessment algorithm. Where indicated, the family physician undertakes a more comprehensive medical assessment, medication review and further referral or intervention where appropriate" (cited in trial protocol). |
| Who provided | The FFN coordinator has gerontological expertise, and was trained in fall prevention. A trained practitioner or physical therapist delivered the otago exercise programme.  T**ypes of healthcare practitioners delivering the intervention:** Three types of healthcare practitioners: Nurse, trained practitioner, and physical therapist. |
| How | - |
| Where | All components of the intervention were delivered in the participants' home. Follow-up contact was maintained by phone. |
| When and how much | The assessment part of the intervention was usually undertaken at one visit. The nurse telephoned 2 to 4 weeks later to ensure that referral consultations had taken place. The otago exercise programme was delivered for 1 year during home visits at weeks 1, 2, 4, and 8 and after 6 months. |
| Tailoring | - |
| Modificaction | - |
| How well: Planned | After completion of the assessment, the nurse made the referrals and followed up to ensure that contact was made with the Otago Exercise Programme exercise instructor". Participants were given monthly calendars to fill in an dreturn to researchers recording daily adherence to exercises and a walking plan. |
| How well: Actual | - |
| **TIDieR control intervention description** | |
| Brief name | Usual care plus an offer of two social visits |
| Why | The offer of at least two social visits was made to control for the effect of social contact by the falls and fracture nurse coordinator and exercise initiator in the intervention group. |
| What: Materials | The pamphlet was produce by the New Zealand Accident Compensation Corporation. Participants received printed information on falls prevention. |
| What: Procedures | All family physicians in the area were invited to an evening educational session about falls prevention, osteoporosis, and fracture prevention as part of regular regional continuing education. This ensured that the physicians had basic background when referrals were made to them for identified falls risk factors. In addition to usual care, participants in the control group were offered at least two social visits from an accredited visitor such as a nursing student, within one month of enrolment. Control participants also received a pamphlet produced by the New Zealand Accident Compensation Corporation about prevention of falls in older adults. |
| Who provided | - |
| How | - |
| Where | - |
| When and how much | - |
| Tailoring | - |
| Modification | - |
| How well: Planned | - |
| How well: Actual | - |

Ferrer et al. (2014)

| Funding source | Public sector: This work was supported by the Fondo de Investigación Sanitaria – Instituto de Salud Carlos III, Spain (grant PS09/00552). |
| --- | --- |
| Type of intervention | Fall risk assessment and interventions based on CDS. |
| iCAT_SR domain 2: Active components included in the intervention, in relation to the comparison | **Judgement:**  More than one component and delivered as a bundle.  **Support for judgement:**  Fall risk assessment preceded the visit to the participants, delivery of fall prevention interventions, and referrals. There was a clear order; therefore bundle as opposed to a package. |
| Notes |  |
| **TIDieR experimental intervention description** | |
| Where located: | Primary paper. |
| Brief name | Multifactorial fall risk assessment and treatment recommendations made to patient and family physician based on algorithm |
| Why | - |
| What: Materials | The intervention used an algorithm that identified nine areas of potentially modifiable risk factors for falls, including psychotropic and cardiovascular drug use, auditory acuity, visual acuity, balance and gait disorders, risk of malnutrition, disability, cognitive impairment, social risk, and home safety. The algorithm evaluated long-term prescriptions, with special emphasis on significant polypharmacy (five or more prescriptions), progressive discontinuation of benzodiazepines, and nutritional or vitamin supplementation. "During the second year, two specifc interventions were also offered as another set of recommendations, ie, rehabilitation and nutritional assessment" (cited in journal article). |
| What: Procedures | Subjects in the intervention group were assessed for their risk of falling. A treatment plan was devised based on their existing medical care and service networks in the community. After the baseline interview, a healthcare professional (doctor or nurse from the health center) visited participants in the intervention group to give recommendations according to the algorithm. For cognitively impaired participants, caregivers were required to be an integral part of the program and ensure that the intervention was implemented. Participants were advised to contact their primary physician to review the results, recommendations, and referrals. Each participant's family physician was mailed after the examination to discuss referrals to medical specialists, changes in medication, and follow-up. "Subjects were referred to an ophthalmologist if their worst corrected monocular near vision was less than 0.5/1 decimals on the Jaeger chart. If there was visual field impairment, the patient was advised to alter their lighting at home to improve visibility (high ambient light level, convential wall-plug night light). Participants with gait disorders were referred to physical therapists for assessment and balance and strength training. There was a focus on progressive balance exercises over 3 months" (cited in journal article). "Information given was reinforced with printed sheets of standard exercises adapted to this age group. The algorithm also generated recommendations for treatment of auditory impairment when the participant was unable to hear a whispered voice at approximately 0.6 m, for risk of malnutrition, and for functional or cognitive decline when deemed necessary" (cited in journal article). "Rehabilitation assessment included subjects with one or more falls and no or minor cognitive impairment (Mini-Mental State Examination .19/35). These subjects received four 90-minute sessions with a physiotherapist over the course of 6 months coordinated by a specialist in rehabilitation at the referral hospital. Subjects at nutritional risk (Mini-Nutritional Assessment score #23.5/30) had three individual one-hour sessions with a dietician from the referral hospital, who developed plans for individualized nutrition. The nutritionist monitored nutritional intervention at the health care center at 3, 6, and 12 months. At the end of each session, the participants received printed information for use at home" (cited in journal article). |
| Who provided | Doctor and nurse received specialized training in geriatrics.  T**ypes of healthcare practitioners delivering the intervention:** Two types of healthcare practitioners: Physician and nurse. |
| How | Intervention components were provided individually and face-to-face. |
| Where | The different component of the intervention were delivered at different locations in seven primary health care centers in Baix Llobregat, Barcelona. |
| When and how much | There was a focus on progressive balance exercises over a period of 3 months. During the second year, two specifc interventions were also offered as another set of recommendations, ie, rehabilitation and nutritional assessment Subjects with one or more falls and no or minor cognitive impairment received four 90-minute sessions with a physiotherapist over the course of 6 months. The nutritionist monitored nutritional intervention at the health care center at 3, 6, and 12 months. |
| Tailoring | - |
| Modificaction | - |
| How well: Planned | - |
| How well: Actual | "Although it is diffcult to identify the effect of a particular component in this type of multifactorial intervention, one explanation for the lack of effect could be a paradoxical rather than undesirable effect of increased physical activity. The higher physical therapy adherence during the second year (90%) versus the frst year (74%) resulted in an increased time at risk and therefore a higher risk of falls and injuries (fractures). Another reason for this effect could be low adherence with the home safety assessment (54% adherence), ie, a systematic approach to identify home hazards as an important component of the intervention, including stairs in the home and lighting factors.35–37 This might be supported by the proportion of home hazards at baseline, which was found to be higher in the intervention group than in the control group. Moreover, there was poor adherence with hospital-based physical therapy and nutritional interventions in the current study" (cited in journal article). |
| **TIDieR control intervention description** | |
| Brief name | Usual health care |
| Why | - |
| What: Materials | - |
| What: Procedures | "Participants in the control group received usual health care" (cited in journal article). |
| Who provided | - |
| How | - |
| Where | The different component of the intervention were delivered at different locations in seven primary health care centers in Baix Llobregat, Barcelona. |
| When and how much | - |
| Tailoring | - |
| Modification | - |
| How well: Planned | - |
| How well: Actual | - |

Frankenthal et al. (2014)

| Funding source | Public sector: This work was supported partly by a research grant from Keshet Association for the Elderly in Tel-Aviv-Yaffo. |
| --- | --- |
| Type of intervention | Medication review and recommendations made to physician. |
| iCAT_SR domain 2: Active components included in the intervention, in relation to the comparison | **Judgement:**  More than one component and delivered as a bundle.  **Support for judgement:**  Medication review preceded discussion with chief physician; therefore bundle as opposed to a package. |
| Notes |  |
| **TIDieR experimental intervention description** | |
| Where located: | Primary paper. |
| Brief name | Screening medications with STOPP/START criteria followed up with recommendations to the chief physician |
| Why | Little is known about the effect of an intervention involving the application of STOPP/START criteria on clinical and economic outcomes. One randomised controlled trial is cited and found that screening medications using the STOPP/START criteria significantly improved prescribing appropriateness. Potentially inappropriate prescriptions (PIPs) are highly prevalent in older adults and are associated with adverse drug events, hospitalisations, morbidity, and inefficient healthcare use. The STOPP criteria focus on avoiding the use of drugs that are potentially inappropriate for older people. The START criteria aim to identify undertreatment or prescribing omissions in older adults. |
| What: Materials | Screening Tool of Older Persons potentially inappropriate Prescriptions/Screening Tool to Alert doctors to Right Treatment (STOPP/START) medication intervention on clinical and economic outcomes |
| What: Procedures | The intervention consisted of a medication review by the study pharmacist for all residents at baseline and 6 and 12 months later. The STOPP/START criteria were applied to identify potentially inappropriate prescriptions (PIPs) and potential prescription omission (PPOs). Recommendations that the pharmacist made for residents in the intervention group but not in the control group were discussed with the chief physician at baseline and at 6 months. The chief physician decided whether to accept these recommendations and implement prescribing changes. |
| Who provided | The study pharmacist and physician.  T**ypes of healthcare practitioners delivering the intervention:** Two types of healthcare practitioners: Pharmacist and physician. |
| How | The medication review was conducted face-to-face. |
| Where | The intervention was implemented at a chronic care geriatric facility in central Israel. The facility has 384 beds and is staffed by five physicians and one chief physician who is the medical director of the facility. It is divided into 12 wards: five nursing departments for residents dependent in their activities of daily living (ADLs) with and without cognitive impairment (ADL-dependent group), four departments for elderly adults independent in their ADLs but dependent in instrumental ADLs (e.g., use of telephone, shopping, food preparation, travel, hosekeeping, handling finances (ADL-independent group), and three departments for residents who are primarily cognitively impaired but are able to walk independently and therefore need special care to prevent them from getting lost (primarily cognitively impaired group). |
| When and how much | At study opening and at six and 12 months later a medication review was conducted. |
| Tailoring | The recommendations made by the pharmacist based on the STOPP/START criteria aim to optimise the prescriptions made to each individual resident. |
| Modificaction |  |
| How well: Planned | The study did not assess adherence to the intervention or fidelity. However, acceptance rate was assessed, i.e., the proportion of recommendations made by the pharmacist that the physician accepted and altered. |
| How well: Actual | The physician accepted 82.4% of STOPP recomendations (n=202) and 92.6% of START recommendations (n=76), altering the prescriptions accordingly. |
| **TIDieR control intervention description** | |
| Brief name | Usual pharmaceutical care |
| Why | - |
| What: Materials | - |
| What: Procedures | The study pharmacist did not make interventional recommendations regarding prescription changes to residents. |
| Who provided | The study pharmacist (that also provided the experimental intervention). |
| How | - |
| Where | Chronic care geriatric facility |
| When and how much | - |
| Tailoring | - |
| Modification | - |
| How well: Planned | - |
| How well: Actual | - |

Gallagher, O’Connor, & O’Mahony (2011)

| Funding source | Public sector: The study was funded by the Health Research Board of Ireland, Clinical Research Training Fellowship number CRT/2006/029. |
| --- | --- |
| Type of intervention | Medication review and recommendations made to physician. |
| iCAT_SR domain 2: Active components included in the intervention, in relation to the comparison | **Judgement:**  More than one component and delivered as a bundle.  **Support for judgement:**  Intervention consisted of several components which would have been delivered in sequence, therefore, bundle as opposed to a package. |
| Notes |  |
| **TIDieR experimental intervention description** | |
| Where located: | Primary paper. |
| Brief name | Screening with STOPP/START criteria followed up with recommendations to patients’ attending physicians |
| Why |  |
| What: Materials | STOPP/START criteria. |
| What: Procedures | The primary research physician applied STOPP/START criteria to the baseline data of patients to identify potentially inappropriate prescriptions and prescribing omissions. These were immediately discussed with the attending medical team and followed up with a written communication within 24 hours. Interventional recommendations consisted of simple statements highlighting potentially inappropriate prescriptions according to relevant STOPP/START criteria, e.g., "digoxin at a daily dose of 250 μg in this patient with renal impairment is potentially inappropriate because of increased risk of digoxin toxicity”; “long-term use of neuroleptic medication in this patient with Parkinsonism is potentially inappropriate owing to risk of worsening extrapyramidal symptoms”; “glibenclamide in this patient with diabetes is potentially inappropriate because of the risk of prolonged hypoglycemia”; “warfarin should be considered for cardioembolic prophylaxis in this patient with chronic atrial fibrillation (no contraindication)”. The receiving physician judged whether to accept the recommendations and implement prescribing changes. Medication changes were included in the discharge summary to the patient’s general practitioner. |
| Who provided | Primary research physician: The primary research physician was clinically trained to obtain informed consent and in applying the STOPP/START criteria. Attending physician  T**ypes of healthcare practitioners delivering the intervention:** One type of healthcare practitioner: Hospital physician. |
| How | - |
| Where | Cork University Hospital, an 800-bed, state-funded, tertiary medical center in southern Ireland serving an urban and rural population. |
| When and how much | - |
| Tailoring | - |
| Modificaction | - |
| How well: Planned | - |
| How well: Actual | "The interrater reliability of the interventional recommendations was tested through a study in which nine physicians practicing independently in six European countries applied STOPP/START criteria to 20 cases comprising 181 medications. The methodology and results have been previously described. Interrater reliability was good, with κ-coefficients of 0.93 for STOPP recommendations and 0.85 for START recommendations." (journal article) |
| **TIDieR control intervention description** | |
| Brief name | Usual pharmaceutical care |
| Why | - |
| What: Materials | - |
| What: Procedures | - |
| Who provided | - |
| How | - |
| Where | Cork University Hospital, an 800-bed, state-funded, tertiary medical center in southern Ireland serving an urban and rural population. |
| When and how much | - |
| Tailoring | - |
| Modification | - |
| How well: Planned | - |
| How well: Actual | - |

Ganz et al. (2015) and Wenger et al. (2010)

| Funding source | Public sector: Ganz et al. (2015): This project was supported by Grant R01AG036776 from the National Institute on Aging (NIA). Dr. Jennings was supported by the UCLA Claude Pepper Older Americans Independence Center funded by the NIA (5P30AG028748) and National Institutes of Health. National Center for Advancing Translational Science UCLA Clinical and Translational Science Institute Grant UL1TR000124.  Public sector: Wenger et al. (2010): This project was supported by grant 11719 from The Atlantic Philanthropies to the American College of Physicians. |
| --- | --- |
| Type of intervention | Fall risk assessment and interventions based on CDS. |
| iCAT_SR domain 2: Active components included in the intervention, in relation to the comparison | **Judgement:**  More than one component and delivered as a bundle.  **Support for judgement:**  Several of the five key components of the ACOVE-prime intervention had to be delivered in order. Therefore bundle as opposed to a package. |
| Notes |  |
| **TIDieR experimental intervention description** | |
| Where located: | Primary paper and Wenger et al. (2010). |
| Brief name | Assessing Care of Vulnerable Elders Practice Redesign for Improved Medical Care for Elders (ACOVEprime) |
| Why | The intervention was developed to improve the quality of care for falls, incontinence, and cognitive impairment among community-dwelling adults age 75 and older. The theory behind the intervention was to develop a low-tech, practical restructuring of care delivery in the primary care office that follows the principles of the Chronic Care Model. The intervention is novel in that it "does not require the hiring of additional personnel to improve quality. Rather, practice redesign hanges the basic process of care. This approach has a great appeal because it may cost little and have potential for widespread dissemination. Moreover, this intervention has demonstrated to be extremely effective in improving the quality of care fo falls in several community-based clinical trials" (cited in Reuben et al. ACOVEprime research proposal & study protocol). |
| What: Materials | ACOVEprime has five key components which were customised to fit the practice's patient flow and electronic staff resources: 1. Case finding 2. Efficient collection of condition-specific clinical data 3. Medicard record prompts 4. Patient and family education materials 5. Health care professional decision support and physician and staff education (Wenger et al. 2010). |
| What: Procedures | In both intervention and control practices, the study screened individuals aged 75 and older to identify individuals at high risk of future falls using the questions: - have you fallen two or more times in the past 12 months? - have you fallen and hurt yourself since your last visit to the doctor? - are you afraid that you might fall because of balance or walking problems? In both intervention and control practices the screening results were made available to the treating primary care provider. Intervention practices implemented face-to-face clinician education about falls and incontinence at the start of the intervention period, decision support to prompt primary care providers to take appropriate action in response to a positive screen (using a paper-based structured visit note templates or computerised electronic health record prompts), and patient education handouts referring patients to appropriate community resources (e.g. exercise programs for fall prevention). An audit and feedback component was included in the intervention, where providers abstracted their own charts and received feedback when improvement was needed. From the original ACOVEprime proposal to: 1. Case finding Patients in the project will be identified prior to routine office visits. This screening for any of the targeted syndromes will be conducted by a brief review of medical records (e.g. to determine if a patient has heart failure) or using a few simple questions that can be administered over the telephone or by office staff at the time of the visit. In the new intervention, case-finding will differ from that employed in ACOVE-2. In the prior study, the screening procedure was linked to enrollment in a controlled trial and case-finding was separated from usual patient care. In the new intervention, the practices, with help from redesign consultants (the Practice Redesign and Evaluation Team), will identify a method of screening that fits into each practice's routine. This might include a brief (<30 second) survey completed at the time of appointment check-in carried out by office staff or brief items inserted into the pre-visit reminder telephone call. For practices with an EMR, a periodic (e.g. annual) mailing could go out to at-risk patients prior to appointments. The practice redesign consultants will suggest screening questions and ideas for implementation, but the clinical sites will chose what method fits their particular practice. 2. Efficient collection of condition-specific clinical data, utilizing non-physician staff and limited automatic orders Identification of a target condition triggers a multifaceted intervention that begins at the time of the patient visit, prior to actually seeing the physician. The completed screening tool (Attachment 4 is an example from ACOVE-2), collected prior to a routine office visit, is attached, along with a structured visit note and supporting educational materials, to the ambulatory chart at the time of the scheduled office visit. These materials prompt the physician to address the identified condition, leads her/him through the appropriate data collection and care process, and may serve as the progress note for that visit. Components of the data collection may be delegated by the practice to office staff and patient flow may be altered to better fit the approach to the condition. 3. Medical record prompts The structured visit note (Attachment 5 is an example of the falls structured visit note from ACOVE-2) suggests to the physician the appropriate care process for the condition that will lead to successful diagnosis and therapy, including patient education and community linkages. Within the context of an EMR, these prompts can trigger follow-up reminders. 4. Patient education materials ACOVE-2 uses simple patient education materials made available to physicians on paper (Attachment 6 is an example from ACOVE-2). These materials include condition-specific information for patients and families, tools to guide home care (such as exercise guides) and materials to enhance adherence (such as calendars). Participating practices can suggest additional materials that will be useful. The proposed project also will offer a wide variety of educational materials on the PIER web site (http://pier.acponline.org/), suitable for downloading on the physician’s letterhead. The web base will allow constant updating of patient educational information, ease of bilingual versions, and has the ability to track usage. The site also offers extraordinarily robust linkages to virtually all College publications and educational resources. PIER is also designed for relatively easy linkage with Electronic Medical Record systems and is already widely downloaded for PDA use. 5. Physician decision support and physician education An example of materials developed by ACOVE-2 is attached (Attachment 7 is an example from ACOVE-2). As with patient information, we plan to make this information web-based, allowing extensive point-of-service physician information. Based on the ACOVE-2 experience and other research, simply providing educational materials is not sufficient to change behavior. Accordingly, geriatricians who have expertise in office management of these conditions will personally teach physicians how to modify and incorporate these educational materials into their practices. In addition to the core components of ACOVE-2, i.e. efficient collection of condition-specific clinical data, medical record prompts to encourage performance of essential care processes, patient education materials and activation of the patient's role in follow-up, and physician decision support and physician education), ACOVEprime includes two components that were not in the original ACOVE-2 intervention - identification of patients with target conditions by office staff and a quality improvement component that persists beyond basic training. ---------- From (Wenger et al. 2010): 1. Case finding Before clinic visits, all patients 75 years and older were screened for falls or fear of falling and urinary incontinence, and the results were available at the clinic visit 2. Efficient collection of condition-specific clinical data A structured visit note and supporting educational materials were added to the patient's medical record at the time of the office visit. Participating health care professionals and their staff decided what clinical data collection (e.g. history taking) would be delegated to office staff and any alterations in patient flow to facilitate data collection. For example, staff placing the patient in a clinic room could follow automatic orders to complete simple condition-related procedures (e.g. orthostatic blood pressure). 3. Medicard record prompts The structured visit note prompted health care professionals to address the identified condition and led them through data collection and diagnostic and therapeutic care processes, including patient education and referrals to community resources. 4. Patient and family education materials Patients were empowered by education materials for target conditions (eg, home safety checklist, pelvic floor exercises). Each practice developed local community resource lists for each condition (eg, community-based exercise programs). 5. Health care professional decision support and physician and staff education Health care professionals participated in a 2-hour educational program (led by D.B.R. or W.J.H.) that taught an approach to each target condition that could be completed during an office visit. A nurse (C.P.R.) conducted a separate training session to orient office staff to intervention implementation. |
| Who provided | Physicians (ACP atlantic proposal): Clinicians were given face-to-face education about falls and incontinence at the start of the intervention period.  T**ypes of healthcare practitioners delivering the intervention:** Three types of healthcare practitioners: Primary care physician, nurse, physician assistant. |
| How | The intervention was delivered face-to-face. |
| Where | Five community-based medical practices to participated in the ACOVEprime intervention as a quality improvement project focused on care for older patients. The practices were selected with the intent of including sites representing variation in geographic location, environment, size and patient characteristics. The five sites were located in rural New York and small to medium sized cities in Pennsylvania, Wisconsin, Arizona, and Oregon. Sites needed to have at least three primary care clinicians caring for an adequate number of older patients and to have either a clinic or pod that could act as the control clinic (Reuben et al. ACOVEprime research proposal & study protocol). |
| When and how much | Patients were screened for falls or fear of falling prior to visiting the clinic. |
| Tailoring | "By design, all sites implemented all components of the intervention, but there was flexibility about how decision support was implemented and how patiente education materials were created and used" (cited in Ganz et al. 2015). "Each practice had flexibility in implementing the intervention. As a result, there were fundamental differences in the ways practices implemented intervention components. For example, condition screening was completed by nursing staff at 4 sites and by patient completion of a written previsit screener at the final site. One site planned to have patients return for a follow-up visit to manage the target condition. One site had a fully integrated electronic health record (EHR), 1 site had a medical record with electronic components, and 3 sites used paper records" (cited in Wenger et al. 2010). |
| Modificaction |  |
| How well: Planned |  |
| How well: Actual | "In a sample of 1 037 participants whose medical records were reviewed, intervention participants received 60% of recommended care for falls during the intervention period, compared with 37.6% for controls (p < .001)" (cited in Ganz et al. 2015). |
| **TIDieR control intervention description** | |
| Brief name | Usual care |
| Why | - |
| What: Materials | - |
| What: Procedures | In the intervention and control practices, the study screened individuals aged 75 and older to identify individuals at high risk of future falls using the questions: - Have you fallen two or more times in the past 12 months? - Have you fallen and hurt yourself since your last visit to the doctor? - Are you afraid that you might fall because of balance or walking problems? In the intervention and control practices, screening results were made available to the treating primary care provider. Unline in intervention practices, control practices did not provide clinician education about falls and incontinence, decision support paper-based structured visit note templates or computerised electronic health record prompts, or patient education handouts. Control practices did not include an audit and feedback. |
| Who provided | - |
| How | - |
| Where | - |
| When and how much | - |
| Tailoring | - |
| Modification | - |
| How well: Planned | - |
| How well: Actual | - |

Groshaus et al. (2012)

| Funding source | - |
| --- | --- |
| Type of intervention | Fall risk assessment and interventions based on CDS. |
| iCAT_SR domain 2: Active components included in the intervention, in relation to the comparison | **Judgement:**  More than one component and delivered as a bundle.  **Support for judgement:**  Fall risk screening was initiated prior to falls preventions trategies. There was a clear order; therefore bundle as opposed to a package. |
| Notes |  |
| **TIDieR experimental intervention description** | |
| Where located: | Primary paper. |
| Brief name | Multi-component knowledge translation intervention that incorporated a nurse-initiated clinical decision support tool |
| Why | Clinical decision support provided to physicians has been fount to improve practitioner performance and possibly patient outcomes. The impact of nursing practice is less clear. The intervention s a multi-component knowledge translation (KT) intervention that incorporated a clinical decision support tool to reduce harms in the care of older medical inpatients. The intervention targeted the care nurses provide to older medical patients in acute care hospitals. |
| What: Materials | The intervention involved three components: 1. An electronic nurse-initiated order set (e.g. delirium and fall risk screening; regular reorientation, ambulation and toileting protocols; feeding assistance; bowel routines; falls prevention strategies; non-pharmacological sleep routines; pain monitoring; encouragement of independence in activities of daily living) orderable by nurses from within a menu of common order sets for medical patients, 2. educational in-servicing (e.g. education about relevant age-related changes and evidence-informed strategies to provide optimal care to the older in-patient), and 3. a binder of geriatric resource materials (e.g. cognitive and depression screening; behavioural mapping; non-pharmacologic delirium prevention strategies; falls screening and prevention; normal age-related changes). |
| What: Procedures |  |
| Who provided | Nurses  T**ypes of healthcare practitioners delivering the intervention:** One type of healthcare practitioner: Nurses. |
| How | The intervention was delivered face-to-face between nurses and in-patients at the hospital units. |
| Where | As this study used a stepped-wedge design, six medical units at two hospitals in Calgary, Alberta, Canada received the intervention in a staggered fashion. |
| When and how much |  |
| Tailoring |  |
| Modificaction |  |
| How well: Planned | Two frameworks were used to implement the intervention: the MRC framework and the Knowledge to Action Cycle. "The electronic nurse-initiated order set was developed by a multi-disciplinary team using evidence obtained from published clinical trials, systematic reviews and practice guidelines. The order set incorporates the current best evidence aimed at addressing delirium, falls, continence promotion, and optimal nutrition and hydration. Issues of workflow impact and sustainability were considered during ddevelopment. The order set was made available within the hospitals’electronic medical record, after it was vetted by frontline nurses, nurse educators and managers, local professional practice leaders, nursing council and clinical informaticians. The research team and local geriatric clinical nurse developed standardised educational materials containing information that supported the electronic order set and the overall care of the older medical inpatient. The brief in-servicing (i.e. 15 minutes) was conducted on a new unit at the start of every 2-week study period. It was offered multiple times on each unit in order to help ensure the majority of unit nurses had the opportunity to attend, and it was kept brief in order to allow nurses to attend during a work shift. At the start of the 12-weel rollout, the order set was available to all users of the electronic medical record but the unit nurses were not informed about its presence until the educational in-servicing." (cited in Groshaus et al. 2012). |
| How well: Actual | "Seven unit nurses and six nurse managers/educators were interviewed. Most felt the order set was good basic nursing care and therefore many unit nurses were not ordering it but thought it could be a useful resource for new nurses. The perceived main barrier to the use of the order set was the high workload on these busy medical units and that there was a need for more staff or volunteer support to help in the care of older inpatients. Many of those interviewed reported that the order set was too wordy and contained long paragraphs. Both unit nurses and nurse managers/educators were unfamiliar with the geriatric resource binder and reported that is was not being used. Several nurse managers/educators felt Internet resources would more likely be used. The three interviewed unit nurses who attended the brief educational in-servicing felt the information provided was good basic nursing care. Among the four interviewed nurse managers/educators whom attended the in-servicing, two felt the information could be included in the future unit nurse teaching" (cited in Groshaus et al. 2012). |
| **TIDieR control intervention description** | |
| Brief name | Control |
| Why | - |
| What: Materials | - |
| What: Procedures | - |
| Who provided | Nurses |
| How | - |
| Where | As this study used a stepped-wedge design, six medical units at two hospitals in Calgary, Alberta, Canada received the intervention in a staggered fashion. Until the experimental intervention was rolled out on a unit, it acted as a control unit for purposes of analysis. |
| When and how much | - |
| Tailoring | - |
| Modification | - |
| How well: Planned | - |
| How well: Actual | - |

Healey et al. (2004)

| Funding source | "None." |
| --- | --- |
| Type of intervention | Fall risk assessment and interventions based on CDS. |
| iCAT_SR domain 2: Active components included in the intervention, in relation to the comparison | **Judgement:**  More than one component and delivered as a bundle.  **Support for judgement:**  Health screening preceded targeted fall prevention interventions. There was a clear order; therefore bundle as opposed to a package. |
| Notes |  |
| **TIDieR experimental intervention description** | |
| Where located: | Primary paper. |
| Brief name | Targeted risk factor factor reduction core care plan |
| Why | - |
| What: Materials | Pre-printed care plan: The pre-printed care plan included risk factors for falls that could be properly addressed in the hospital where the study took place. On the reverse side of the plan, a brief summary of evidence was written, such as medication most likely to be implicated in falls, and local advice such as optical testing arrangements. "The falls care plan was interleaved with accident reporting forms already in routine use on the wards throughout the study period, to prompt its use when ‘near misses’ or falls occurred." |
| What: Procedures | "Nursing staff on intervention wards were asked to apply the intervention to patients admitted with a history of falls, those who had fallen or had a ‘near miss’ during their current admission. This would not identify all patients at risk of falling, but would focus the intervention on the patients at highest risk. It was anticipated that nurses’ experience of the falls care plan could affect their practice in relation to other patients, and might result in reduction of risk factors for those patients not formally identified as requiring a falls care plan." The intervention included the following clinical decision support, which consisted of a health screening checklist along with recommended targeted interventions: - Eyesight (able to recognise pen/key/watch from two meters distance: If unable to recognise, optician visit if lost glasses, ophthalmology referral if no known reason for poor eyesight. - Medication - check for sedatives, anti-depressants, diuretics, polypharmacy, etc: Medical review of prescription benefit related to fall risk. - Lying and standing blood pressure: Refer any deficit to medical staff. Advise patient on changing position slowly. - Ward test urine: Send mid-stream urine sample if positive for nitrites, blood or protein. - Difficulty with mobility: Refer to physiotherapist. - Environmental check - Review risk/benefit of bedrails for individual: Documentation of risk/benefit in nursing notes and removal or addition of bedrails as appropriate. - Footwear safety: Advise relatives on replacement. - Bed height: Keep at lowest height. - Position in ward: Nurse patient with history of falls as close to nurses' station as possible (considering other patients' needs). - Simple environmental cause of falls (e.g., loose cable, wet floor): Act to correct it - Nurse call bell: Explained and within reach. |
| Who provided | Hospital nurses: "No specific training was provided – the nurses already used pre-printed care plans for other conditions, and the simple format made this unnecessary."  T**ypes of healthcare practitioners delivering the intervention:** One type of healthcare practitioner: Nurses. |
| How | - |
| Where | The study took place, and the interventions were delivered, in eight care of the elderly wards or units of a district general hospital serving a mixed urban and rural population in the UK. |
| When and how much | - |
| Tailoring | - |
| Modificaction | - |
| How well: Planned | "The study did not attempt to measure or enforce use of the care plan for all appropriate patients." |
| How well: Actual | - |
| **TIDieR control intervention description** | |
| Brief name | No change in practice or environment relevant to fall prevention |
| Why | - |
| What: Materials | - |
| What: Procedures | "Managers on control wards were made aware of the study, and the need not to introduce the care plan in their area. Control wards made no other changes to practice or environment relevant to falls prevention during the study. Whilst nurses instigated the process, remedial interventions were multi-disciplinary, including mobility assessment by physiotherapists and medication review by medical staff." |
| Who provided | - |
| How | - |
| Where | The study took place, and the interventions were delivered, in eight care of the elderly wards or units of a district general hospital serving a mixed urban and rural population in the UK. |
| When and how much | - |
| Tailoring | - |
| Modification | - |
| How well: Planned | - |
| How well: Actual | - |

Lightbody et al. (2002)

| Funding source | Public sector: This study was funded by North West Region NHS Executive and supported by Liverpool and Wirral Research and Development Liaison Group. |
| --- | --- |
| Type of intervention | Fall risk assessment and interventions based on CDS. |
| iCAT_SR domain 2: Active components included in the intervention, in relation to the comparison | **Judgement:**  More than one component and delivered as a bundle.  **Support for judgement:**  Fall risk assessment preceded advice and education, referral to reltives, community therapy services, social services, and/or the primary care team. There was a clear order; therefore bundle as opposed to a package. |
| Notes |  |
| **TIDieR experimental intervention description** | |
| Where located: | Primary paper. |
| Brief name | Nurse-led fall prevention management plan and care pathway for older people |
| Why | "Interventions using existing resources and mechanisms are likely to be implemented and be sustainable. This study assessed a nurse-led intervention for older people discharged from the Accident and Emergency Department, requiring a single visit, where action on falls risk factor modification could be taken through usual channels." "The intervention aimed to use existing resources and referral mechanisms." |
| What: Materials | An adapted version of the falls checklist "S" test A falls diary: At randomisation patients were given a diary to complete daily for up to six months after the index fall. |
| What: Procedures | A falls nurse assessed the patient's risk factors for falls at home two to four weeks following the Index fall (the current fall). Medication, ECG, blood pressure, cognition, visual acuity, hearing, vestibular dysfunction, balance, mobility, feet and footwear were assessed using adapted versions of the falls checklist and "s" test. The environmental assessment identified inadequate lighting, tripping, hazards and unsuitable furniture. Patients were given advice and education about safety in the home, and simple modifications were made with consent (e.g. mat removal). Risk factors requiring further action were referred to relatives, community therapy services, social services and/or the primary care team. Direct referrals were not made to hospital outpatients or day hospital. In the falls diary that the patients were given, patients tracked falls, consequent injury, subsequent place of treatment (i.e., GP, hospital). |
| Who provided | Nurses: "Following some basic training, therapists and clinicians agreed about the nurse's initial assessment and criteria for onward referral, as some areas require specialist assessment, e.g. provision of aids and adaptations."  T**ypes of healthcare practitioners delivering the intervention:** One type of healthcare practitioner: Nurses. |
| How |  |
| Where | The Accident and Emergency Department of University Hospital Aintree, Liverpool, a teaching hospital with a catchment population of 250 000. |
| When and how much | The intervention required only a single visit. |
| Tailoring | The recommended intervention to prevent falls that the nurse identified were based on a nurse assessment of medication, ECG, blood pressure, cognition, visual acuity, hearing, vestibular dysfunction, balance, mobility, feet and footwear. |
| Modificaction |  |
| How well: Planned |  |
| How well: Actual | "Where the falls nurse could take action on interventions directly, e.g. by removing mats or social service referrals, risk factors were modified by 6 months in most cases. However, where interventions had to be taken indirectly, risk factors were less likely to have been addressed. For example, as polypharmacy increases falls liability, it was imperative for the falls nurse to facilitate modification of target medications." |
| **TIDieR control intervention description** | |
| Brief name | Usual care |
| Why | - |
| What: Materials | - |
| What: Procedures | - |
| Who provided | - |
| How | - |
| Where | The Accident and Emergency Department of University Hospital Aintree, Liverpool, a teaching hospital with a catchment population of 250 000. |
| When and how much | - |
| Tailoring | - |
| Modification | - |
| How well: Planned | - |
| How well: Actual | - |

Logan et al. (2021)

| Funding source | Public sector: This study was funded by the National Institute for Health Research (NIHR) HTA programme (ref 13/115/29). PAL, JCH, JRFG, and ALG are funded in part by the NIHR Applied Research CollaborationEast Midlands (ARC-EM). PAL, JRFG, and ALG are funded in part by the NIHR Nottingham Biomedical Research Centre. The views expressed are those of the authors and not necessarily those of the NIHR, NHS, or Department of Health and Social Care. |
| --- | --- |
| Type of intervention | Fall risk assessment and interventions based on CDS. |
| iCAT_SR domain 2: Active components included in the intervention, in relation to the comparison | **Judgement:**  More than one component and delivered as a bundle.  **Support for judgement:**  Training of care home staff preceded fall risk screening, which preceded staff actions. There was a clear order; therefore bundle as opposed to a package. |
| Notes |  |
| **TIDieR experimental intervention description** | |
| Where located: | Supplementary Material S1: Intervention Description |
| Brief name | The Guide to Action Care Homes (GtACH) Falls Prevention Programme. |
| Why | The relatively untrained nature of care home staff, the complex nature of falls risk factors in care residents and the need for multiple interventions to address multiple risk factors requires a systematic home-wide programme including staff education and support, in the use of risk assessment and decision support tools |
| What: Materials | • GtACH training slides – these were used by the NHS Falls Lead to train each intervention home  • The GtACH manual – given to care home staff to support implementation of the GtACH paper screening and assessment tool. It included a master copy of the paper tool, information about the study, a copy of the training session slides, falls information (including definition of a fall, why falls are important and causes of falls), instructions on how to complete the GtACH paper screening and assessment tool, a Falls Incident Analysis template, a Medication and Falls Chart and information on how to obtain further expert advice or support from the local falls’ expert.  • The GtACH screening and assessment tool comprised 33 items related to falls risk factors grouped into four domains: falls history, medical history, movement/environment, and personal needs. The presence of risk factors prompts up to 30 individual staff actions.  • Attendance certificate – given to care home staff at the end of training  • A4 sized poster – given to care homes for display in the home to act as a reminder to implement the GtACH programme in the home |
| What: Procedures | • Care home staff training provided by the local NHS Falls Lead, lasted one hour per session and included: purpose of the study, purpose of the training, prevalence of falls in care homes, GtACH history, and how to complete and where to file completed forms. It emphasised consistent delivery, referenced the materials listed above especially the GtACH manual. Case studies and role play were used. |
| Who provided | • The NHS Falls Lead was a registered nurse, physiotherapist or occupational therapist who was trained and specialised in falls prevention and bone health.  • A care home Falls Champion, with roles for awareness raising and liaison between staff and the NHS Falls Lead, was identified in each care home. No additional training was provided.  • Trained staff were taught to complete the GtACH screening and assessment tool with every resident, within four weeks after training, in private, and discussed with family, friends and other care home staff. Completed GtACH documentation was to be placed in the resident’s care records and expected to contribute to the care plan for each resident. Re-assessment was expected if the resident developed a new health condition, or fall, or every three-six months.  T**ypes of healthcare practitioners delivering the intervention:** Three types of healthcare practitioners: Nurse, physiotherapist, occupational therapist. |
| How | • Training was given face to face. |
| Where | • Training was provided in a room within the care facility which was deemed appropriate for training by the care facility manager/owner. |
| When and how much | • Each session was one hour. As it was not feasible for all care home staff to attend a single training session, repeated training sessions were offered according to care home staff availability. Staff were not remunerated, their roles were not back filled, repeated sessions maximized opportunity for all staff to attend. |
| Tailoring | • Completed GtACH documentation was to be placed in the resident’s care records and expected to contribute to the care plan for each resident. Re-assessment was expected if the resident developed a new health condition, or fall, or every three-six months. |
| Modificaction | • The training period for care homes was extended from two weeks to four weeks (post randomisation) to enable fall leads time to train very large homes (+50 staff members). |
| How well: Planned | • Fidelity of the intervention is reported separately in the process evaluation which was conducted in parallel to the trial. |
| How well: Actual |  |
| **TIDieR control intervention description** | |
| Brief name | Usual care. |
| Why | - |
| What: Materials | - |
| What: Procedures | To aid recruitment, retention, and adherence to the protocol, all control homes were oﬀered the intervention after the 12 month data had been collected and checked. |
| Who provided | - |
| How | - |
| Where | - |
| When and how much | - |
| Tailoring | - |
| Modification | - |
| How well: Planned | - |
| How well: Actual | - |

Mahoney et al. (2007)

| Funding source | Public sector: A Prevention Grant to the Kenosha County Aging and Disability Resource Center from the Wisconsin Department of Health and Family Services funded this study and work for the preparation of this report. Jane Mahoney, Terry Shea, Robert Przybelski, and Ronald Gangnon have received grants from the Centers for Disease Control and Prevention. Jane Mahoney has received additional support from The Alliance Provider Quality Investment Fund. Kenosha County provided additional support for LaVerne Jaros for conduct of this research. |
| --- | --- |
| Type of intervention | Fall risk assessment and interventions based on CDS. |
| iCAT_SR domain 2: Active components included in the intervention, in relation to the comparison | **Judgement:**  More than one component and delivered as a bundle.  **Support for judgement:**  Fall risk assessment preceded recommendations and referrals and mailing of the recommendations to the subjects’ primary physicians. There was a clear order; therefore bundle as opposed to a package. |
| Notes |  |
| **TIDieR experimental intervention description** | |
| Where located: | Primary paper. |
| Brief name | Intermediate-intensity, community-based multifactorial falls risk assessment and recommendations to primary physician |
| Why | "There is a need to evaluate practical, intermediate-intensity, multifactorial models for their efficacy in reducing falls in the community. An intermediate-intensity multifactorial model was defined as one that provides primarily referrals and recommendations without delivering physical therapy or progressive exercise but with a greater number of contacts with the older adult than a low-intensity approach (>4). It was hypothesized that more contacts would lead to greater adherence and efficacy in reducing falls. The purpose of this study was to test, in a randomized, controlled trial, the efficacy of this intermediate-intensity, individual, multifactorial model to reduce falls" (cited in journal article). |
| What: Materials | In-home multifactorial assessment and intervention using an algorithm. "The intervention used an algorithm based on the University of Wisconsin Falls Prevention Clinic, designed to identify predisposing factors for falls; induce reduction changes in medical conditions, medications, behavior, physical status, and home environment through recommendations to participants and their physicians, and make sure these changes are long-lasting through follow-up and linkages to other care networks" (cited in Mahoney et al. 2007). 11 monthly telephone calls. The calls were made to answer questions, facilitate necessary referrals, and encourage adherence to physical therapy, exercise, and other recommendations. |
| What: Procedures | In-home multifactorial assessment and intervention. A physiotherapist or a registered nurse used the algorithm in the patient's home. "The algorithm evaluated medications, distant vision, balance and gait, some neurological deficits, cognition, mood, home functioning, and home safety. Tools used for the evaluations and examples of corresponding interventions are shown in Table 1" (cited in Mahoney et al. 2007). Based on the multifactorial assessment the physiotherapist or nurse provided recommendations and referrals. "Recommendations were mailed to subjects' primary physicians, and the participants were asked to see their primary physician within 1 month to review the recommendations. Recommendations were made to the physicians to evaluate and reduce psychotropic medications and provide other medical care to elucidate and treat medical causes of falls. A geriatrician (including JM, RP) reviewed all recommendations to physicians. The algorithm generated referrals and recommendations to physical therapy and other healthcare providers based on specific criteria. Required triggers for physical therapy referral included moderate impairment on Berg Balance Scale items, abnormal gait on the Performance Oriented Mobility Assessment, inability to stand for 30 seconds with eyes open on hard surface or foam, and history of pain with walking or doing exercise. Optional triggers included loss of balance with sternal nudge,13 positive Romberg test, absent vibratory sensation at the ankle or metatarsal phalangeal joint, inability to stand for 30 seconds with eyes closed on hard surface or foam, total score less than 80 on the Activities Specific Balance Confidence Scale, and any potentially risky mobility-related activity of daily living (ADL) or instrumental activity of daily living (IADL), per assessor’s judgment. The co-investigator physical therapist (TS), who agreed 100% of the time when the algorithm did not recommend physical therapy, reviewed all assessments. If the algorithm did not recommend physical therapy or if the participant refused physical therapy, then the study therapist provided a set of balance and leg strengthening exercises. The assessment generated referrals for further medical evaluation and treatment (e.g., occupational therapy, ophthalmology, podiatry). The participant was directly referred to ophthalmology or podiatry; referrals for physical or occupational therapy required the primary physician’s signature. The therapist or nurse assisted with setting up referrals and facilitated acquisition of home equipment (e.g., walker or other ambulation aid, bathroom equipment, rails). Costs were covered by private pay or through the study" (cited in Mahoney et al. 2007). Recommendations for long-term exercise. "All subjects in the intervention group were given recommendations for long-term exercise. A recommended long-term exercise program was walking at least 4 to 5 days per week and performance of standing balance exercises 2 to 3 days per week, preferably in a group setting. Standing balance exercise was defined as any exercise that included weight shifts and head turn. It included activities such as dancing, bowling, and tai chi. If the subject received outside physical therapy, the assessor asked the therapist to prescribe a home exercise program at the end of therapy, to encourage participation in a group exercise program, or both" (cited in Mahoney et al. 2007). 11 monthly telephone calls. |
| Who provided | Physiotherapist and registered nurse. The physiotherapist and registered nurse received 3 days of additional training in a standardized fashion from a geriatrician and physiotherapist on the multidisciplinary components used in the algorithm. Physician.  T**ypes of healthcare practitioners delivering the intervention:** Three types of healthcare practitioners: Physiotherapist, nurse, physician. |
| How |  |
| Where | The multifactorial assessment and intervention was provided in the participants' home. |
| When and how much | The physiotherapist or nurse visited intervention participants in their homes twice within the first 3 weeks after enrollment to perform the assessment and give recommendations and referrals. Intervention group participants were also given 11 monthly telephone calls. |
| Tailoring |  |
| Modificaction |  |
| How well: Planned | An exercise plan, a monthly exercise calendar, and 11 monthly telephone calls from the assessor facilitated adherence to exercise. "Adherence to recommendations was monitored using monthly telephone calls from the study assessor. Adherence was categorized as visits to the primary physician, visits to other referral providers if recommended, visits to physical therapy if recommended, changes in medications if recommended, use of a current assistive device or acquisition of a new assistive device, and exercise. A recommendation was adhered to if it was at least partially completed at any time in the 12-month period. Days of balance exercise and days and minutes of walking were monitored using a monthly calendar or a telephone call if a calendar was not returned. The physical therapist to whom the participant was referred corroborated information regarding number of physical therapy visits" (cited in Mahoney et al. 2007). |
| How well: Actual | Some limitations regarding tracking adherence are that adherence to recommendations was only tracked at 4 months and 1 year, and adherence to exercise relied on self-report, which may be subjects to bias. Contamination of the control group was possible, which may have biased the effect estimate toward the null. |
| **TIDieR control intervention description** | |
| Brief name | Home safety visits |
| Why | - |
| What: Materials | - |
| What: Procedures | Participants in the control group received an in-home assessment from an occupational therapist that was limited to home safety recommendations and advice to see their doctor about falls. |
| Who provided | An occupational therapist. |
| How | - |
| Where | - |
| When and how much | - |
| Tailoring | - |
| Modification | - |
| How well: Planned | - |
| How well: Actual | - |

Peterson et al. (2007)

| Funding source | - |
| --- | --- |
| Type of intervention | Guided medication dosing using computerised CDS. |
| iCAT_SR domain 2: Active components included in the intervention, in relation to the comparison | **Judgement:**  One component.  **Support for judgement:**  Intervention consisted of guided medication dosing within a computerized provider order entry; a single component. |
| Notes |  |
| **TIDieR experimental intervention description** | |
| Where located: | Primary paper. |
| Brief name | Guided dosing within a computerized provider order entry (CPOE) presented to physicians |
| Why | "Guided dosing within a computerized provider order entry (CPOE) system is an effective method of individualizing therapy for patients. Physicians’ responses to guided dosing decision support have not been extensively studied. As part of a randomized trial evaluating efficacy of dosing advice on reducing falls in the elderly, CPOE prompts to physicians for 88 drugs included tailored messages and guided dose lists with recommended initial doses and frequencies" (cited in Peterson et al. 2007). |
| What: Materials | "Figure 1 shows the editor screen that enables customization of dose lists, setting minimum and maximum single doses, and designating default doses for each study medication" (cited in Peterson et al. 2007). |
| What: Procedures | "During care of elderly patients, the guided dosing system delivered advice to physicians about appropriate initial dosing for sedatives, neuroleptics, anti-emetics and skeletal muscle relaxants for the most common indications. The geriatric dosing advisor also discouraged prescription of contraindicated drugs as defined by Fick and Beers (i.e. the "Beers Criteria"). Because appropriate sedative and neuroleptic dosing ranges are broad for high-acuity (e.g., intensive care unit) patients, no barriers prevented selecting higher doses than recommended. The system utilized the same computational infrastructure that project members had previously implemented for specialized age- and weight-based pediatric dosing" (cited in Peterson et al. 2007). |
| Who provided | Physicians  T**ypes of healthcare practitioners delivering the intervention:** One type of healthcare practitioner: Hospital physicians. |
| How | The guided geriatric dosing was presented on-screen to physicians. Physicians delivered the intervention to hospital in-patients face-to-face. |
| Where | The study took place in a tertiary care academic health center with 10 years experience with a self-developed CPOE system. |
| When and how much | - |
| Tailoring | - |
| Modificaction | - |
| How well: Planned | "Authors derived study-related dosing information for FDA-approved indications from “package insert” monographs, and for “off-label” indications from the medical literature and published textbooks. The CPOE-based text messages displayed along with study dosing information communicated titration strategies, possible adverse effects, and key monitoring parameters (Figure 2). An advisory group of 2 geriatricians [JP, RH], a geriatrics pharmacist [DH], and an internist [JFP] reviewed the doses and associated messages for accuracy" (cited in Peterson et al. 2007). |
| How well: Actual | "In order to assess the effect of physician crossover, where physicians cared for both control and intervention patients, we compared the prescribed dosing from physicians who only cared for control patients (n=117) to physicians who only cared for intervention patients (n=103). Intervention only physicians prescribed a significantly lower dose than control-only physicians (median 2.0 [1.0,4.0] vs median 4.0 [2.0,6.0], p<0001). The potential for crossover was also assessed by comparing a pre-trial period of 2 months (2315 medication orders) to the trial period. Pre-trial dosing was significantly higher with median 3.0 (2.0, 6.0) vs. median 3.0 (1.0, 5.0) for the trial period, p<0.001" (cited in Peterson et al. 2007). |
| **TIDieR control intervention description** | |
| Brief name | Usual physician practice with no guided dosing presented to physicians |
| Why | - |
| What: Materials | - |
| What: Procedures | - |
| Who provided | Most likely the same physicians who provided the experimental intervention. |
| How | Physicians delivered the intervention to hospital in-patients face-to-face. |
| Where | The study took place in a tertiary care academic health center with 10 years experience with a self-developed CPOE system. |
| When and how much | - |
| Tailoring | - |
| Modification | - |
| How well: Planned | - |
| How well: Actual | - |

Phelan et al. (2024)

| Funding source | Centers for Disease Control (GRANT NUMBER U01CE002967). |
| --- | --- |
| Type of intervention | Medication review and recommendations made to physician. |
| iCAT_SR domain 2: Active components included in the intervention, in relation to the comparison | **Judgement:**  More than one component and delivered as a package.  **Support for judgement:**  Intervention consisted of patient education handouts and clinician decision support used to reduce use of the target medications. Materials to patients were delivered via postal mail, and there was no specific order in the delivery of the components. |
| Notes |  |
| **TIDieR experimental intervention description** | |
| Where located: | Primary paper and study protocol. |
| Brief name | The STOPP-FALLS intervention: Patient education and clinician decision support. |
| Why | Falls are the most frequent cause of fatal and non-fatal injuries among people aged 65 years and older. Meducation use, particularly use of medications that affect the central nervous system, has been consistently linked to falls. Practice guidelines recommend that prescribers review all medications with their older patients to minimize polypharmacy and the use of central nervous system-active and other high-risk medications. However, this practice is not routinely followed due to multiple barriers, including lack of healthcare provider and patient awareness that medications can cause falls. The “D-PRESCRIBE trial” in Canada delivered by community pharmacists evaluated an educational intervention directed to patients and decision support to providers. The intervention was highly effective in reducing the use of potentially inappropriate medications by older adults. However, effects on health outcomes, including falls, were not reported. STOP-FALLS adapted the D-PRESCRIBE intervention for use in an integrated healthcare delivery system in the USA and assessed its effectiveness on medically treated falls. |
| What: Materials | Patient education   - Patient educational brochures, self-care handouts, and a public-domain brochure on ways to prevent falls.   “Patient education consists of educational brochures and self-care/symptom management handouts. Educational brochures were adapted from prior deprescribing trials conducted in Canada for three of the medication classes targeted by STOP-FALLS: opioids, sedative-hypnotics (benzodiazepines and Z-drugs), and first-generation antihistamines. We adapted these materials with input from KPWA delivery system members, including clinical and pharmacy leadership and PCPs.” (cited in trial protocol, Balderson et al. 2023).  Provider decision support  “Provider decision support consists of two elements: An evidence-based pharmaceutical opinion (EBPO) and “deprescribing pearls”. The EBPOs, modelled after those of the D-PRESCRIBE trial, describe the risks associated with the targeted medication class, alternative evidence-based treatments that could be tried to help a participant reduce their use of the medication, and hyperlinks to practice supports for deprescribing (e.g., pharmacy consultation, mental health referral, and self-care support tools).” (cited in trial protocol, Balderson et al. 2023).  “In addition to EBPOs, “deprescribing pearls” will be distributed to all intervention clinic PCPs, regardless of whether they have a participant enrolled. Providers in other settings have endorsed the need for guidance on how to initiate deprescribing discussions. The content of the pearls was developed by STOP-FALLS investigators based on the published literature. Each pearl also givs several examples of how to broach discussions of deprescribing with patients, referred to as “conversation starters”. The pearls were modelled after “clinical pearls” used by KPWA to disseminate clinical information updates and thus are anticipated to feel familiar to providers.” |
| What: Procedures | Prior to intervention implementation, each clinic received a 30-min presentation on the study methods and patient and provider materials, with an emphasis that changes to medication prescriptions were up to their clinical discretion.” (cited in trial protocol, Balderson et al. 2023). |
| Who provided | Physicians  T**ypes of healthcare practitioners delivering the intervention:** One type of healthcare practitioner: Primary care physicians. |
| How | Patient education  All materials were delivered via postal mail.  Provider decision support  Evidence-based pharmaceutical opinions were delivered to the clinician synchronous with mailing of the patient education materials. A brief version of the EBPO was sent to clinicians via the electronic health record staff messaging system with a hyperlink to the full-length EBPO on the study website.  Deprescribing pearls were delivered via periodic emails to the clinic’s study champion who disseminated these materials as they saw fit, e.g., via email. Deprescribing pearls were distributed to all intervention clinic PCPs regardless of whether they had a participant enrolled. |
| Where | The study took place in primary care clinics of the Kaiser Permanente Washington (KPWA) integrated group practice. Eighteen clinics participated: 9 per study arm. |
| When and how much | - |
| Tailoring | Patients were able to select which of the education materials they would study. “Although the intervention materials encourage participants to talk with their PCP about their medication regimens, changes to medications are at the discretion of the participant and their PCP.” (cited in trial protocol, Balderson et al. 2023).  Providers selected, at their own discretion, what evidence-based pharmaceutical opinions and deprescribing pearls to focus on, what discussions to initiate with their conversation, and what changes to their patients’ medication prescriptions that were necessary. |
| Modificaction | - |
| How well: Planned | A pilot study was conducted in within a single intervention clinic (n = 142) and matched control clinic (n = 160) to test study procedures and intervention materials.  “The intervention encourages but does not require behavior change on the part of the participant or their PCP. However, participant and PCP communication about the material and medication changes may be considered proxy responses to the intervention. We will closely examine medication prescriptions and instructions for discontinuation and tapering within the medical chart. We will also examine postcards returned by patient participants regarding if they intend to discuss materials with their PCP.” (cited in trial protocol, Balderson et al. 2023).  “All participant and provider materials have been carefully cross-referenced with KPWA clinical practice guidelines and reviewed by leaders in the KPWA delivery system so that all information and recommendations are concordant with KPWA guidance.” (cited in trial protocol, Balderson et al. 2023). |
| How well: Actual | - |
| **TIDieR control intervention description** | |
| Brief name | Usual care. |
| Why | “Usual care is a common comparator for pragmatic trials, as it captures a wide, realistic range of practice scenarios and controls for changes that may occur within the cohort and the healthcare system. Given there is no contact with the usual care group (i.e., no participant survey or measurement), this further helps to create a “real-world” comparison.” (cited in trial protocol, Balderson et al. 2023). |
| What: Materials | - |
| What: Procedures | - |
| Who provided | Physicians in the control clinics.  T**ypes of healthcare practitioners delivering the intervention:** One type of healthcare practitioner: Primary care physicians. |
| How | Physicians delivered the intervention face-to-face to patients visiting the control clinics. |
| Where | The study took place in primary care clinics of the Kaiser Permanente Washington (KPWA) integrated group practice. Eighteen clinics participated: 9 per study arm. |
| When and how much | - |
| Tailoring | - |
| Modification | - |
| How well: Planned | - |
| How well: Actual | - |

Snooks et al. (2014)

| Funding source | Public sector: Funding provided by Department of Health Ref: 0200055 https://www.gov.uk/government/organisations/department-of-health. The funders had no role in study design, data collection and analysis, decision to publish, or preparation of the manuscript. |
| --- | --- |
| Type of intervention | Computerised CDS presented to paramedics on hand-held tablets. |
| iCAT_SR domain 2: Active components included in the intervention, in relation to the comparison | **Judgement:**  More than one component and delivered as a bundle.  **Support for judgement:**  Training preceded use of the decision support, which preceded potential referrals. There was a clear order; therefore bundle as opposed to a package. |
| Notes |  |
| **TIDieR experimental intervention description** | |
| Where located: | Primary paper and Supplemental material: Protocol S1. |
| Brief name | Computerised Clinical Decision Support on hand-held Tablet computers used by paramedics attending older people who fall |
| Why | It was expected that integrating the CCDS system into the handheld tablet computers to be used by paramedics would improve process and outcomes of care that are clinically important to patients or operationally important to service providers. Specifically, the investigators theorised that the intervention would reduce the number of attendances at EDs in favour of referrals to alternative community-based falls services.  Supplement:  The Clinical Decision Software (CDS) was intended to be used on-scene by paramedics who attended older adults for whom a 999 emergency call was made due to a fall. The CDS was used through a hand-held device to support paramedics to make triage decisions for older people who have fallen, in order to decide who should be taken to A&E and who can be left at home with self-care advice and/or onward referral to a community based service. |
| What: Materials | Paramedics in the experimental intervention arm were provided with CCDS on handheld tablet computers. Site one implemented the CCDS simultaneously with a system for electronic patient data capture; while site two, where a different electronic data capture system was already in place, added CCDS software to the existing system.  Supplement:  The intervention consists of four components: 1. Training: Both clinical and technology based 2. Decision support guidance to assess and plan care for older fallers 3. The hardware and software 4. Referral to community-based care providers The technology will allow the traditional ambulance service patient report form to be replaced with a digital record on the tablet PC. |
| What: Procedures | The CCDS was used by paramedics to decide whether to take patients who had fallen to an ED or leave them at home with referral to a community-based falls service. Both the experimental intervention and the control group could refer people who had suffered a fall to community-based falls services.  Supplement:  Paramedics are expected to use the computer to create an electronic record for every 999 call. "When the crew member attends a patient that meets the inclusion criteria for this study, the additional functionality of the decision support for assessing falls will also be used to assess whether the older person who has fallen should be taken to A&E or offered an alternative care plan. The CDS prompts the assessment and examination of any injuries associated with the fall, as well as co-morbidity that may have contributed to the fall (such as breathlessness or chest pain) and the patient's psychosocial needs (such as their metnal state and their ability to undertake activities of daily living). An assessment of environmental risk is also included. Based on these assessments, the CDS suggests an appropriate care plan (such as patient advice, referral to specific community based services etc.)" (cited in Protocol S1). |
| Who provided | Paramedics: "Paramedics were eligible to participate in SAFER 1 if they worked at any of 13 ambulance stations with a falls referral pathway in place; they continued to be eligible if they moved from one of these stations to another" (cited in journal article).  Supplement: Paramedics: "Paramedics allocated to the intervention group will receive additional training (two days) in the use of the CDS. Following initial training there will be a pre-trial period of one month during which trained crews will be expected to practice using the hand-held computer in place of paper Patient Record Forms, and the CDS for falls assessment as and when appropriate. Towards the end of this period, their use of the CDS will be audited to ensure that they have achieved proficiency. Training will be carried out with groups of 4 paramedics, and will last two days, split so that the initial training is followed up a refresher training session/competency test after three weeks of practice. Training consists of systematic demonstration of the mechanics and functionality of the software coupled with practice and supervised structured and unstructured role play. Critical reflection and discussion is undertaken and encouraged throughout the training programme. Knowledge reviews are carried out at certain points of the process to ensure competence and understanding of key aspects of the software functionality" (cted in Protocol S1).  T**ypes of healthcare practitioners delivering the intervention:** One type of healthcare practitioner: Paramedics. |
| How | The computerised clinical decision support was displayed on-screen to participating paramedics. Paramedics attended older adults who had fallen in their homes. |
| Where | The intervention was implemented at two study sites. Site one, an urban centre where the study recruited paramedics from four ambulance stations; and site two, where the study recruited paramedics from nine stations across a mixed urban and rural area.  Supplement:  The intervention will be tested with paramedics at two study sites (S Wales, Great Western Ambulance Service). |
| When and how much | - |
| Tailoring | There were two sites. Site one implemented the CCDS simultaneously with a system for electronic patient data capture; while site two, where a different electronic data capture system was already in place, added CCDS software to the existing system. Neither site fully integrated CCDS with the electronic software; in particular site one experienced many teething problems including loss of network signal and hardware failures. |
| Modificaction | - |
| How well: Planned | - |
| How well: Actual | - |
| **TIDieR control intervention description** | |
| Brief name | Usual care with paper-based protocols to assess patients and make decisions about their care instead of computerised clinical decision support |
| Why | - |
| What: Materials | - |
| What: Procedures | "Control paramedics at both sites provided usual care, with paper-based protocols to assess patients and make decisions about their care, including patients who had fallen. Usual care comprised assessment, treatment on scene as required and default conveyance to the Emergency Department unless the patient refused to travel to hospital" (cited in journal article). Both the experimental intervention and the control group could refer people who had suffered a fall to community-based falls services |
| Who provided | Paramedics who were randomly allocated to the control group. The paramedics allocated to the control group did not receive the additional training that the paramedics allocated to the intervention group received. |
| How | Instead of using on-screen decision support, paramedics in the control group used paper-based protocols to assess patients and make care decisions. |
| Where | The control intervention was implemented at two study sites. Site one, an urban centre where the study recruited paramedics from four ambulance stations; and site two, where the study recruited paramedics from nine stations across a mixed urban and rural area. |
| When and how much | - |
| Tailoring | "Although we know that practice is variable, we did not attempt to standardise care in the control arm as there is little evidence about what is best for patients" (cited in journal article). |
| Modification | - |
| How well: Planned | - |
| How well: Actual | - |

Tamblyn et al. (2012)

| Funding source | Public sector: RT is supported by the Canadian Institutes of Health Research and the Canadian Patient Safety Institute. TE is supported by The CIHR Frederick Banting and Charles Best Canada Graduate Scholarship and CIHR Emerging Team Grant. DB is supported by a Canada Research Chair in Public Health Informatics. |
| --- | --- |
| Type of intervention | Guided medication dosing using computerised CDS. |
| iCAT_SR domain 2: Active components included in the intervention, in relation to the comparison | **Judgement:**  One component.  **Support for judgement:**  Automated computer alerts: The physician received a patient-specific risk of injury alert (single component). |
| Notes |  |
| **TIDieR experimental intervention description** | |
| Where located: | Primary paper. |
| Brief name | Computerized prescribing decision support presenting patient-specific risk of psychotropic drug-related injury to family physicians |
| Why | "Computerized prescribing and decision support are expected to address preventable medication errors, as these digital technologies can guide dosing and provide alerts on drug treatment duplication, contraindications, and drug interaction errors, especially when integrated with information on all dispensed medication. However, the majority of drug alerts are over-ridden, particularly for psychotropic medication in both hospital-based and community-based studies. Even when drug alert systems are customized to present only clinically important interactions, physicians over-ride the majority of alerts, because they are deemed not clinically relevant and/or the benefit is believed to exceed the risk. Yet, the patient-specific risk is rarely known, even though it can be estimated by incorporating into drug alert systems predictive models of adverse events developed through pharmacoepidemiological studies. The advanced computing power available in today’s electronic record systems and the focus on individualized medicine provides an unprecedented opportunity to integrate detailed patient data into complex predictive models for estimating patient risk" (cited in Tamblyn et al. 2012) "In this study, we tested the hypothesis that the incorporation of patient-specific risk estimates into a computerized prescribing decision-support system in primary care would increase physician response to alerts for psychotropic medication and reduce the risk of psychotropic drug-related injury in older adults, particularly for patients with a higher baseline risk" (cited in Tamblyn et al. 2012). |
| What: Materials | Both intervention and control group used the MOXXI community-based clinical information system (CIS). A link to the provincial insurance agency (RAMQ) was used to pre-populate demographic information for the practice population based on a study physician's billings from the previous year. For patients who consented to participate in the research network, all medical services and prescription drugs provided for the past year were loaded into the MOXXI CIS, and thereafter all new records of medical services and prescriptions were refreshed on a daily basis. |
| What: Procedures | The data on medical services and prescription drugs loaded into the MOXXI CIS were used to update the profile of dispensed medications, dates and reasons for emergency department visits and hospitalisations, medical and surgical procedures, and health problems. "A commercial drug alert system (http://www.vigilance.ca) automatically reviewed each new prescription for potential contraindications, including therapy duplication, dosing error, cumulative toxicity, and drug-disease, drug-drug, and drug-allergy interactions. Physicians could set the threshold for the alert system to one of three levels (1, severe alerts only; 2, moderate and severe alerts; 3, all alerts), which restricted alerts generated automatically during the prescribing process. By default, the system was set to level 2. However, all alerts generated for a patient were available for the physician to review in a drug alert summary in the patient’s electronic chart. Physicians randomized to the control and intervention groups had access to these standard features of the MOXXI CIS" (cited in Tamblyn et al. 2012). "Physicians randomised to the intervention group received a patient-specific risk of injury alert when a patient was prescribed a psychotropic medication that increased the risk of injury. The personalised alert used a published predictive model to estimate the risk of injury based on the patient's age, sex, injury history, presence of cognitive impairment, gait, and balance problems, and doses of selected psychotropic medication (selective serotonin / nor-epinephrine reuptake inhibitors antidepressants, antipsychotics, low-, intermediate- and high-potency opiates, intermediate- and long-acting benzodiazepines, anticonvulsants, and first-generation antihistamines). We set a relatively low threshold for showing the alert - an increase in risk of 1 per 1000 - to enable us to assess the likelihood of changes in drug treatment as a function of the magnitude of patient risk" (cited in Tamblyn et al. 2012). "Graphics, in the form of risk thermometers, were created to show physicians the patient’s risk of injury in the next 12 months related to psychotropic medication as well as nonmodifiable characteristics (eg, age, sex) (figure 1A). Physicians would see the risk thermometer, annotated with numeric values generated by the risk calculation described above, when they opened the patient drug profile or when they prescribed a psychotropic drug. Drugs that contributed to the risk calculation were highlighted in the patient’s drug profile. If the physician attempted to reduce the risk of injury by stopping or decreasing the dose of a psychotropic medication, the absolute and relative reduction in risk would be shown as an adjustment in the level of the thermometer and a change in the numeric values (figure 1B). If a new psychotropic drug was started or the dose was increased, the absolute and relative increase in the risk would be shown (figure 1C). If no change in medication was instituted (or the risk was increased by a medication change), physicians had to select a reason for the decision from a standardized pick-list (eg, prescribed by another physician). A reference section was available with publications on the risk of injury related to psychotropic drug use and methods of tapering benzodiazepines" (cited in Tamblyn et al. 2012). |
| Who provided | Physicians: Physicians in the experimental intervention group received a 5-minute training program on the risk of injury alert that outlined how to interpret the risk thermometer information, the expected changes in risk with new or discontinued medication, the completion of reasons for not changing therapy if applicable, and the location of reference information. Physicians were advised that stopping or reducing the dose of highlighted medications in the patient’s drug profile would reduce the patient’s risk of injury.  T**ypes of healthcare practitioners delivering the intervention:** One type of healthcare practitioner: Primary care physicians. |
| How | The new features of the MOXXI CIS used in the experimental intervention were presented on-screen to physicians. Physicians provided care face-to-face with patients. |
| Where | The intervention was implemented in a population of 41 family physicians and 2887 of their older patients. Physicians practiced in urban community settings in Montreal or Quebec City, Canada. |
| When and how much | The intervention was implemented from September 2008 to July 2010. The information about patient-specific risk of injury was computed and presented to the physician at the time of each visit. |
| Tailoring | - |
| Modificaction | - |
| How well: Planned | - |
| How well: Actual | - |
| **TIDieR control intervention description** | |
| Brief name | Access to standard features of the MOXXI clinical information system without patient-specific risk of psychotropic drug-related injury presented to physicians |
| Why | - |
| What: Materials | - |
| What: Procedures | "A commercial drug alert system (http://www.vigilance.ca) automatically reviewed each new prescription for potential contraindications, including therapy duplication, dosing error, cumulative toxicity, and drugedisease, drugedrug, and drugeallergy interactions. Physicians could set the threshold for the alert system to one of three levels (1, severe alerts only; 2, moderate and severe alerts; 3, all alerts), which restricted alerts generated automatically during the prescribing process. By default, the system was set to level 2. However, all alerts generated for a patient were available for the physician to review in a drug alert summary in the patient’s electronic chart. Physicians randomized to the control and intervention groups had access to these standard features of the MOXXI CIS" (cited in Tamblyn et al. 2012). Physicians in the control intervention group had access to the standard features of the MOXXI CIS but did not receive patient-specific risk of injury alerts when a patient was prescribed a psychotropic medication that increased the risk of injury. Physicians in the control intervention group did were not presented with graphics in the form of risk thermometers to show the patient's risk of injury in the next 12 months related to psychotropic medication. |
| Who provided | - |
| How | - |
| Where | The intervention was implemented in a population of 40 family physicians and 2741 of their older patients. Physicians practiced in urban community settings in Montreal or Quebec City, Canada. |
| When and how much | - |
| Tailoring | - |
| Modification | - |
| How well: Planned | - |
| How well: Actual | - |

Weber, White, & McIlvried (2008)

| Funding source | Public sector: Agency for Healthcare Research and Quality |
| --- | --- |
| Type of intervention | Medication review and recommendations made to physician. |
| iCAT_SR domain 2: Active components included in the intervention, in relation to the comparison | **Judgement:**  More than one component and delivered as a bundle.  **Support for judgement:**  Medication review preceded sending a message with medication recommendations to the primary care physician via the EMR that also contained an evidence-based guideline on fall prevention. There was a clear order; therefore bundle as opposed to a package. |
| Notes |  |
| **TIDieR experimental intervention description** | |
| Where located: | Primary paper. |
| Brief name | Standardized medication review with recommendations to primary care physician via the electronic medical record |
| Why | "Falls lead to fractures, functional disability, and even death ... Polypharmacy in the elderly is a common phenomenon, with estimates that more than 20% of elderly persons are taking medications with the potential for serious adverse effects, including an increased risk for falls. The number of prescribed drugs has been found to be significant, with total medications of 4 or greater as an independent risk factor for falls in other studies. Medications with sedating properties, and in particular, benzodiazepines, antidepressants, and phenothiazines, have been found to be correlated with an increased risk for falls ... Physicians in office practice struggle with how to fit fall risk factor evaluation into an office visit packed with competing needs; therefore, strategies that uncouple interventions from office visits are needed. Over the past several years, reports of computerized systems being used to improve chronic disease care and/or prevention have appeared in the literature. Such trials showed enhanced compliance with cancer prevention guidelines and increases in the use of cholesterol-lowering drugs for the secondary prevention of coronary disease. Many of these studies used either computer-prompted “reminders” to physicians occurring during visits or guidelines available to physicians on the EMR. As reminders and prompts are more widely used, it is clear that their contextual relationship can impact physician usage. Prompts must be properly timed, be easy and quick to use, and provide helpful content. The response to “automated” prompts can quickly extinguish as physicians’ attention to them declines; a phenomenon known as “alert fatigue”. Conversely, there is strong evidence that physicians do respond well to professional “opinion leaders” in a specific content area and/or academic detailing. The objectives of this study were to evaluate whether a unique EMR-based intervention combined with the use of opinion leaders could reduce medication use and the number of falls in an ambulatory elderly population at risk for falls" (cinted in Weber, White & McIlvried 2008). |
| What: Materials | Evidence-based guideline for fall prevention The guideline was closely based on the American Geriatrics Society/American Academy of Orthopedic Surgery fall prevention guidelines and was reviewed by the GHS Practice Guidelines Committee. The guideline was uploaded onto the EMR before the intervention period. |
| What: Procedures | The GHS clinical pharmacist or geriatricians reviewed each patient's medication record via the EMR. The focus was on the use of medications that would increase the risk for falls. Psychoactive medications, the presence of polypharmacy, and the presence of medications at inappropriate doses were the focus of the review. After the medication review, the primary care physician was sent a message via the EMR. The message alerted the receiving physician that the patient was at risk for falls and made recommendations tailored to the individual patient, including specific medications and/or dosing. As part of the message, physicians were referred to an evidence-based guideline for fall prevention that the physician could access directly through the EMR. The messages were not linked to an in-office patient visit, i.e. they were received by the physician unlinked to a patient office visit. The messages were sent to the patient's primary physician as identified in the EMR.  A GHS clinical pharmacist or MD fellowship-trained geriatrician reviewed each patient's medication record via the EMR, focusing on the use of medications that would increase the risk for falls. The focus of the review were psychoactive medications, the presence of polypharmacy, and the presence of medications at inappropriate doses. After the medication review the primary care physician was sent a message via the EMR. The intervention date was defined as the date the electronic message was sent to the physician. which were in January or February of 2003. The message alerted the receiving physician that the patient was at risk for falls and made recommendations tailored to the individual patient, including specific medications and/or dosing. As part of the message, physicians were referred to an evidence-based guideline for fall prevention that the physician could access directly through the EMR. The guideline was closely based on the American Geriatric Society/American Academy of Orthopedic Surgery fall prevention guidelines and was reviewed by the GHS Practice Guidelines Committee. It was uploaded onto the EMR before the intervention period. The messages were not linked to an in-office patient visit; i.e., they were received by the physician unlinked to a patient office visit. The messages were sent to the patient's primary physician as identified in the EMR. |
| Who provided | Clinical pharmacist or MD fellowship-trained geriatrician The clinical pharmacist and MD fellowship-trained geriatrician had expertise in geriatric pharmacology performed the review and sent messages (recommendations) via the EMR. Patient's primary care physician The patient's primary care physician received messages (recommendations) via the EMR.  T**ypes of healthcare practitioners delivering the intervention:** Three types of healthcare practitioners: Pharmacist, geriatrician and primary care physician. |
| How | The recommendations were sent to the primary physician via the EMR and presented on-screen. The primary physician had face-to-face communication with patients. |
| Where | Primary care practices that were part of the Geisinger Health System in rural central and northeastern Pennsylvania. |
| When and how much | Messages were sent only once per patient during the study period. |
| Tailoring | - |
| Modificaction | - |
| How well: Planned | - |
| How well: Actual | - |
| **TIDieR control intervention description** | |
| Brief name | Usual care without medication review and recommendations sent to primary care physician |
| Why | - |
| What: Materials | - |
| What: Procedures | - |
| Who provided | - |
| How | - |
| Where | Primary care practices that were part of the Geisinger Health System in rural central and northeastern Pennsylvania. |
| When and how much | - |
| Tailoring | - |
| Modification | - |
| How well: Planned | - |
| How well: Actual | - |

Wenger et al. (2009)

| Funding source | Industry funded: "This project was supported by a contract from Pfizer Inc, to RAND. No au-thor reports any conflict of interest with this paper." (journal article) |
| --- | --- |
| Type of intervention | Fall risk assessment and interventions based on CDS. |
| iCAT_SR domain 2: Active components included in the intervention, in relation to the comparison | **Judgement:**  More than one component and delivered as a bundle.  **Support for judgement:**  Case-finding and physician education preceded delivery of the condition-specific interventions. There was a clear order; therefore bundle as opposed to a package. |
| Notes |  |
| **TIDieR experimental intervention description** | |
| Where located: | Primary paper. |
| Brief name | Assessing Care of Vulnerable Elders (ACOVE-2) intervention |
| Why | "In an effort to improve the quality of care for geriatric conditions provided by primary care physicians, a practice change intervention aimed at confronting common obstacles to physician behavior change (physician knowledge deficits; disbelief that specific care processes will produce better out-comes; reliance on the influences of peers rather than the medical literature; and perceived lack of time, resources, and self-efficacy) was developed. The intervention integrated care improvement for falls and gait impairment, urinary in-continence, and cognitive impairment into daily clinical practice so that these conditions could be treated in concert with older patients’ coexisting conditions." (journal article). |
| What: Materials | Structured visit note: The structured visit note facilitated the multicomponent practice-change effort-part of the intervention. Home safety checklist. Instructions on exercises to strengthen the pelvic floor. Information handouts to patients. |
| What: Procedures | The intervention included case-finding, physician education, and a multicomponent practice-change effort that aimed to guide care for each condition to achieve processes of care identified by the ACOVE quality indicators (QIs). Case-finding: In each practice, office staff called all patients aged 75 and older on the telephone approximately 1 week before office visits in consecutive order according to appointment date to determine eligibility for the study. Patients were assigned to the intervention or control group based on the primary care physician with whom they had an appointment at the time of study entry. In a telephone call approximately one week before a scheduled routine outpatient appointment, patients were asked screening questions about their health since the previous clinic visit: - Have you fallen two or more times? Have you fallen and hurt yourself? - Are you afraid that you might fall because of balance or walking problems? - Do you have a problem with urinary incontinence (or your bladder) that is bothersome enough that you would like to know more about how it could be treated? In addition, patients were asked to recall three items after a hiatus of at least 60 seconds. If patients were unable to answer the questions, a proxy who knew the most about the patient's health was asked the screening questions for the patient. In place of the three-item recall, proxies were asked the following question about functionally significant cognitive impairment: - Have you noticed that [patient] has recently had more trouble than in the past with memory for day-to-day happenings around the house, such as remembering where he/she put things, recalling recent events, forgetting what you told him/her or what he/she told you, remembering plans, appointments, or phone calls? Physician education: See "Who provided". Multicomponent practice-change effort: At the subsequent office visit, a note was placed on all screened patients’ charts in both the control and the intervention group indicating the responses to these questions. For patients in the intervention group who screened positive for any of these conditions, a condition-specific intervention was initiated with a structured visit note placed on the medical record along with the positive screen. This note, which could function as the visit note, stimulated efficient collection of condition-specific clinical data, including automatic orders for the nurse or medical assistant to complete simple procedures (e.g., urinalysis and culture or orthostatic blood pressure). It also prompted the physician to perform essential care processes for the condition. Specifically, the structured visit note guided the physician to consider potentially important historical and examination elements and suggested diagnostic tests and treatments. The note also facilitated development of an impression and plan that was supported by patient education materials and physician decision support resources. If the note was followed completely, all QIs measured in this study would be met. Condition-specific patient information (e.g., home safety checklist, instructions on exercises to strengthen the pelvic floor) was assembled for cognitive impairment, falls, and urinary incontinence. For each condition, the practice identified local community-based resources to facilitate management (e.g., community-based exercise groups, transportation assistance). These information handouts, which matched the structured visit notes and physician education, were available in each clinician’s examination room in hanging file folders on a portable stand or in bins mounted on the office wall. These materials were designed to enhance patient understanding of the condition and to augment adherence to the treatment plan while reducing the effort required by clinicians to educate and instruct the patient. |
| Who provided | Primary care physicians: Physicians in the intervention group participated in a 3-hour educational program (led by DR) that taught an efficient approach to each condition. This approach was consistent with the ACOVE QIs and matched the structured visit notes for each condition. Brief, written decision-support information was provided that described the management of each condition.  T**ypes of healthcare practitioners delivering the intervention:** Three types of healthcare practitioners: Primary care physician, nurse, medical assistant. |
| How | - |
| Where | "Two large medical groups in different areas of the urban southern California region participated in the trial. Medical Group 1 is a primary care group with 30 physicians caring for 20,000 patients (67% managed care) who make approximately 44,000 office visits annually. Medical Group 2 is a larger multispecialty group with 100 physicians. This group cares for 140,000 patients (50% managed care) who make 500,000 visits annually. Each medical group had two geographically distinct primary care sites; one functioned as the intervention site and the other as the control site. All primary care physicians at each practice site participated in the trial" (journal article). |
| When and how much | - |
| Tailoring | - |
| Modificaction | "The structure and content of the intervention were presented to each practice so that clinicians could modify the intervention to match practice preferences. Practices altered elements of the structured visit notes (e.g., whether cerebral imaging should be performed for cognitive impairment), and individual clinicians decided which aspects of care their office personnel would provide (e.g., whether a patient who fell would have an orthostatic blood pressure evaluation at check-in). After piloting, each practice held small-group sessions with physicians to review charts of patients who triggered the intervention and to discuss physicians’ experiences with restructuring care for these conditions. Practices then made additional modifications to the intervention" (journal article). |
| How well: Planned | The handout materials were designed to enhance patient understanding of the condition and to augment adherence to the treatment plan while reducing the effort required by clinicians to educate and instruct the patient. |
| How well: Actual | - |
| **TIDieR control intervention description** | |
| Brief name | Usual care |
| Why | - |
| What: Materials | Structured visit note. |
| What: Procedures | At the subsequent office visit, a note was placed on all screened patients’ charts in both the control and the intervention group indicating the responses to these questions. |
| Who provided | - |
| How | - |
| Where | "Two large medical groups in different areas of the urban southern California region participated in the trial. Medical Group 1 is a primary care group with 30 physicians caring for 20,000 patients (67% managed care) who make approximately 44,000 office visits annually. Medical Group 2 is a larger multispecialty group with 100 physicians. This group cares for 140,000 patients (50% managed care) who make 500,000 visits annually. Each medical group had two geographically distinct primary care sites; one functioned as the intervention site and the other as the control site. All primary care physicians at each practice site participated in the trial" (journal article). |
| When and how much | - |
| Tailoring | - |
| Modification | - |
| How well: Planned | - |
| How well: Actual | - |
