# Supplementary material for: Effectiveness of clinical decision support in fall prevention among older adults: A systematic review and meta-analysis
Source: PLoS One. 2026 Jan 12;21(1):e0340025. doi: 10.1371/journal.pone.0340025 (PMC12795367; doi:10.1371/journal.pone.0340025)
Supplement: S6 Table — (DOCX) [file pone.0340025.s006.docx]

**S6 Table. Design factors of CDS**

Table 1 presents an overview of how the GUIDES checklist and two-stream model were used to describe the design factors and sub-domains. Elements of the two-stream model, e.g. type of patient data the CDS tool used and which decision rules were applied, were extracted to further specify the GUIDES domains.

| **Table 1** GUIDES checklist and two-stream model domains and sub-domains. | | | |
| --- | --- | --- | --- |
| **GUIDES design factor** | **Sub-domain** | **Description of sub-domain (GUIDES checklist item** [6]**)** | **Description of sub-domain (Two-stream model checklist item** [7]**)** |
| **Context** | Setting |  | Institution or physical space in which the CDS is being used. |
|  | Purpose of/rationale for using CDS | 1.1 CDS can achieve the defined quality objective (see TIDier checklist). |  |
|  | Users |  | Types of users of the CDS, such as nurses, physiotherapists, or other health personnel. |
|  |  | 1.3 Stakeholders and users accept CDS. |  |
|  |  | 3.3 The system delivers the decision support to the right target person.   - Is the system reaching the targeted users? - Is the system able to facilitate team processes when these are needed? |  |
|  | Patients |  | Patient population. |
|  | Clinical workflow | 1.4 CDS can be added to the existing workload, workflows and systems. |  |
| **Content** | Clinical knowledge | 1.2 The quality of the patient data is accurate. | Sources and types of data elements required. |
|  |  | 2.1 The content provides trustworthy evidence-based information. |  |
|  |  | 2.2 The decision support is relevant and accurate.   - Does the decision support contain accurate information that is pertinent to the care of the patient? - Does the decision support address the information needs of the users? - Is it clear to the users why the decision support information is provided for a given patient? |  |
|  |  | 2.3 The decision support provides an appropriate call to action.   - Is the clinical importance and urgency of the recommended action sufficiently clear? - Is the recommended action clear enough for the targeted users to act on? - Is the advice applicable in the setting in which it will be implemented? - Is it clear how the recommended action fits with other current guidelines? |  |
|  | Patient data |  | Data sources and types of data elements. |
|  | Decision rules |  | The algorithm or rules applied by the CDS when deciding on further actions, recommendations, alerts, etc. |
| **System** | Format | Electronic or paperbased. |  |
|  | Channel | 3.1 The system is easy to use.   - Is it easy for users to interact with the CDS system? - Does the system facilitate (or, at least, not hinder) the workflow of the healthcare providers? - Can the system be customised to provide better user support? - Is the system always up and running? |  |
|  |  | 3.2 The decision support is well delivered.   - Is the advice delivered in an appropriate mode, format and channel? - Is the display of the decision support eye-catching, intuitive, concise, consistent and unambiguous? - Is it appropriate to use specific functions (e.g. pop-ups, computerised restrictions, indications of (dis)agreement) for prioritised decision support? |  |
|  | Trigger |  | Data or interaction that triggers the system to begin processing. |
|  | Presentation |  | How advice/output is presented to the user, in terms of content, timing, and interaction functions. |
|  |  | 2.4 The amount of decision support is manageable for the target user.   - Is the amount of decision support per patient manageable? - Is the total amount of decision support manageable for the healthcare provider? |  |
|  |  | 3.4 The decision support is available at the right time.   - Does the system provide the decision support at a moment of need? |  |
| **Implementation** | Training of CDS users | 4.1 Information to users about the CDS system and its functions is appropriate.   - Is the communication and documentation about the CDS appropriate? - Are help topics related to the functioning of the CDS system available to users? - If necessary, is user training available? |  |
|  | Implementation strategies | 4.2 Other barriers and facilitators to compliance with the decision support advice are assessed/addressed.   - Is there an assessment of the beliefs, attitudes and skills of the providers and patients that may affect adherence? Are actions planned/taken accordingly? - Is there an assessment of the professional interactions affecting adherence, and are actions planned/taken accordingly? |  |
|  |  | 4.3 Implementation is stepwise and the improvements in the CDS system are continuous.   - Is the implementation of the CDS stepwise? - Is a plan in place to collect user feedback and to monitor system usage, performance and outcomes? - Are malfunctions and other problems with use of the CDS quickly fixed? |  |
|  |  | 4.4 Governance of the CDS implementation is appropriate.   - Are all the key stakeholders involved in the planning and implementation of the system? - Is the CDS initiative governed in an efficient, sustainable and equitable way? |  |
|  | Outcome evaluations | Process evaluations, fidelity, adherence etc. |  |

| **Table 2** Description of design factors | | | | | | |
| --- | --- | --- | --- | --- | --- | --- |
| **Publication** | **Intervention name (see TIDieR checklist)** | **CDS name** | **CDS design factors (GUIDES)** | | | |
|  | | | **Enabling CDS Context** | **Appropriate CDS Content** | **Effective CDS System** | **Effective CDS Implementation** |
| Aizen et al. (2015) | Targeted multiple intervention falls prevention program based on patient’s fall risk | Fall risk assessment tool and individual management plans | Setting  Rehabilitation geriatric hospital  Purpose of/rationale for using CDS  “A cornerstone of the most fall prevention programs is prediction of falls by identification of risk factors. […] Clinical prediction rules are tools designed to predict health outcomes. Risk assessment tools for prediction of falls typically include a number of risk factors from a patient’s history and physical exam that predicts falls. […] Our current fall risk assessment tool was derived from the results of our previous studies and from published papers on risk factors and risk assessment tools for falls in hospitalized patients.” [1].  Users  Nurses  Patients  Patients over 65 years of age admitted to hospital wards  Clinical workflow  CDS added on top of routine fall prevention, which consisted of any activities undertaken by the patients recommended or administered by the treating team. | Clinical knowledge  “Our current fall risk assessment tool was derived from the results of our previous studies and from published papers on risk factors and risk assessment tools for falls in hospitalized patients.” [1].  Patient data  The assessment included medical interventions environmental modifications, toilets and shower-room adjustments, mobility care, bed and wheelchair adjustments, behavioral and cognitive treatment and patient and family guidance.  Decision rules  Patients were classified as having minimal, mild, moderate, or high risk of falling. Patients classified as having mild, moderate or high fall risk were included in an individualized fall prevention program. Mild risk patients were assessed and managed individually including medical interventions, environmental modifications, toilets and hower-room adjustement, mobility care, bed and wheelchair adjustment, behavioral and cognitive treatment and patient and family guidance. Moderate risk patients were assessed similarly and received additionally regular orientation guidance, and was assessed for the need of a safety mobility restriction. The safety mobility restriction was assurance that mobility (transfers, walking, toilets usage, etc.) was done only under supervision and/or assistance of a professional staff member. High risk patients were additionally placed in a visible location in the ward, a permanent personal supervision and hip protectors were considered and an urgent multidisciplinary discussion regarding the patient’s individual approach took place. | Format  -  Channel  -  Trigger   - Nurses were instructed to obtain a fall risk score for all patients admitted for rehabilitation once a week. Once a week and a day after each admission, patients were included in the individualised fall prevention program.   Presentation   - Risk assessment tool with intervention recommendations based on risk | Training of CDS users  A comprehensive and detailed training program was delivered to the ward staff prior to delivering the intervention.  Implementation strategies   - Training of CDS users   Outcome evaluations  - |
| Barker et al. (2016) | Nurse-led 6-PACK program for reducing falls and fall-related injuries in acute hospitals | Nine-item fall-risk tool (TNH-STRATIFY) and options for six interventions | Setting  12 participating intervention acute wards, in six public  hospitals  Purpose of/rationale for using CDS  “There is limited high-level evidence to support the effectiveness of multifactorial falls prevention programs in the acute hospital setting. This RCT aimed to provide robust information on the  effectiveness of a targeted, multifactorial, nurse-led falls prevention program. Best practice guidelines recommend the use of targeted multifactorial programs. Nurse-led program as nurses are the primary care providers for hospital patients so are optimally positioned to implement falls prevention activities. Having a single discipline responsible for the primary delivery of a falls prevention program may  improve accountability for implementation. The program components were added to the existing patient care plan to ensure the program activities were integrated into nurse workflow.” (Appendix 1)  Users  Nurses  Patients  All patients admitted to wards during the trial.  Clinical workflow  The program components were added to the existing patient care plan to ensure the program activities were integrated into nurse workflow. | Clinical knowledge  “The components of the 6-PACK programme were selected on the basis of best available evidence at the time of development, expert  opinion, and best practice guideline recommendations.” (journal article)  Patient data  Unable to locate all factors, but the following risk factors were available from Barker et al. (2010):Increased age, impaired balance, drug- and alcohol-related problems, multiple falls (increased weighting of this item; three instead of one).  Decision rules  **Checklist:** Checklist with options for six suggested interventions based on checklist findings  A score of three or higher indicates high fall risk. Nurses updated the fall risk tool for each of their patients each shift and applied a falls alert sign and one or more of the remaining 6-PACK interventions (“falls alert” sign, supervision of patients in the bathroom, ensuring patients’ walking aids are within reach, establishment of a toileting regimen, use of a low-low bed, and use of a bed/chair alarm) to patients classified as being at high risk. The 6-PACK interventions applied to each patient were personalised based on the fall-risk tool findings and clinical judgement of their treating nurse. Risk scores and interventions applied to patients were recorded on a 6-PACK patient care plan and reviewed and updated each shift. | Format  -  Channel  -  Trigger   - Reminders on the use of programme components   Availability  -  Presentation   - Checklist with risk factors and recommended intervention | Training of CDS users  Nurses participated in a one day training session delivered by the change management and program facilitator, and the research lead.  Implementation strategies  Outcome evaluations  “Intervention adherence was assessed via daily medical record audit and structured bedside observation by project-employed data collectors for all patients admitted to the intervention wards during the study period. A report of these data, including implementation league tables that ranked wards’ level of adherence to the 6-PACK program components, were provided to the site clinical leaders each month  during the RCT. The site clinical leaders were expected to communicate this information to the champions, ward nurses and ward Nurse Unit Manager.” (Appendix 1) |
| Bhasin et al. (2020) | Strategies to Reduce Injuries and Develop Confidence in Elders (STRIDE) intervention | Fall risk assessment and generation of recommendations | Setting  Primary care (primary care practices)  Purpose of/rationale for using CDS  Determining individual risk factors based on findings from Pre-Visit Questionnaire and linking individual risk factors to possible interventions (Study protocol).  Users  Nurses  Patients  Community-dwelling adults 70 years or older at increased risk for fall injuries  Clinical workflow  - | Clinical knowledge  -  Patient data  The standardised fall risk assessment included seven modifiable risk factors: Impairment of strength, gait, or balance; use of certain medications; postural hypotension; problems with feet or footwear; vision impairment; osteoporosis or vitamin D deficiency; and home safety hazards.  Decision rules  Standardised protocol-driven recommendations for management of risk factors that were explained to the participant, caregiver, or both with the use of motivational interviewing. An individualised care plan was developed, initially focused on one to three risk factors, that was approved by primary care providers. The risk factors for fall injuries were reassessed annually, and the care plan was revised, as needed.  The intervention used structured visit notes and algorithms to develop an individualised Falls Care Plan. The algorithm generates recommended interventions based on identified fall risk factors. | Format  Electronic. “An important component of the intervention is specialized software that supports the Fall Care Managers (FCMs). This software has two major components: an electronic data capture module that is designed and maintained by DCC [(Data Coordinating Center)], and a workflow support module that is designed and maintained by an external vendor (High5LA)” (study protocol).  Channel  **-**  Trigger  **-**  Presentation   - Algorithm with risk factors and intervention recommendations | Training of CDS users  Nurses had completed a 26-module online course supplemented with a face-to-face session, as well as training in motivational interviewing and continuing education.  Implementation strategies  “The patients and stakeholders bring their unique personal perspective of how falls and fall injuries affect their lives, the difficulties they face in adhering to interventions, what outcomes are important to them, and what attributes of the interventions render them feasible, scalable, and sustainable. The clinical sites bring participants, clinical environments, and expertise in patient-centered research and implementation. The investigators, patients, and  other stakeholders planned and prepared this proposal jointly; these partners will continue to be collaboratively engaged in all aspects of the trial’s implementation.” (trial protocol).  Outcome evaluations  - |
| Ganz et al. (2022) |  |  |  |  |  |  |
| Blalock et al. (2020) | STEADI-Rx | STEADI-Rx algorithm | Setting  Primary care (pharmacies)  Purpose of/rationale for using CDS  “… were implemented to  remediate modifiable risk factors. Current guidelines recommend that healthcare providers ask all older adults about falls and gait/balance difficulties annually. However,  this recommendation is not consistently implemented in  practice. To help integrate fall prevention guidelines in  practice, the Centers for Disease Control and Prevention  (CDC) developed the Stopping Elderly Accidents, Deaths, and Injuries (STEADI) screening algorithm and toolkit. The STEADI algorithm includes standardized screening questions to ask patients to assess fall risk and recommendations for follow-up tailored to patient responses.” (journal article)  Users  Pharmacists  Patients  Adults ≥65 years using either four or more chronic medications or ≥1 medication associated with increased fall risk  Clinical workflow  The researchers developed the STEADI-Rx by adapting the STEADI algorithm and toolkit for use in the community pharmacy setting. Community pharmacy settings | Clinical knowledge  STEADI-RX is based on the STEADI algorithm, which was created by researches at the Center for Disease Control and Prevention’s Injury Center. The researchers reviewed relevant literature and conducted in-depth interviews with health care providers to determine current knowledge and practices related to older adult fall prevention. Draft resources were developed based on the AGS/BGS guideline.  Patient data  Pharmacy staff screened patients by asking the following STEADI questions: (1) Have you fallen in the past year? (2) Do you feel unsteady when standing or walking? And (3) Do you worry about falling? Patients who reported having fallen in the past year were also asked how many times they had fallen and whether any of the falls had resulted in injury.  During the medication review, the pharmacist evaluated the patient’s medication regimen using evidence-based algorithms developed to identify medications associated with an increased risk of falling and provide recommendations to reduce risk.  Decision rules  **Algorithm** used to identify medications associated with increased risk of falling: Patients who answered “yes” to any of the key STEADI questions were classified as having screened positive for increased fall risk and were eligible to receive a medication review provided by a pharmacist. | Format  Electronic  Channel   - The STEADI toolkit was developed based on in-depth interviews with health care providers to make it user friendly.   Trigger  -  Presentation   - Adapted STEADI algorithm with risk factors and intervention recommendations | Each intervention pharmacy received a spreadsheet listing the names of patients served by the pharmacy who met study inclusion criteria. Pharmacy staff screened patients based on the list.  Training of CDS users  Pharmacists have intensive training focused on pharmacotherapy.  Implementation strategies  -  Outcome evaluations  - |
| Blum et al. (2021) | Structured pharmacotherapy optimisation intervention supported by a software-based clinical decision support tool. | Systematic Tool to Reduce Inappropriate Prescribing Assistant (STRIPA) | Setting  Specialist care (hospitals)  Purpose of/rationale for using CDS  Although polypharmacy might be indicated and beneficial in many people with multimorbidity, the risk of inappropriate prescribing is also increased. Inappropriate prescribing might take the form of drug overuse, drug underuse, or drug misuse. A wide variety of interventions have been designed to optimise pharmacotherapy in people with polypharmacy, with the aim of improving drug appropriateness and lowering risk of adverse drug reactions. Most of these structured interventions consist of multifaceted strategies delivered by pharmacists, but more recently, software systems have been developed to support pharmacotherapy optimisation. Although most computerised decision support systems focus on a single aspect, such as detecting drug-drug or drug-disease interactions, the systematic tool to reduce inappropriate prescribing (STRIP) can perform multiple tasks intrinsic to pharmacotherapy optimisation simultaneously. STRIP combines the STOPP/START criteria with a more global evaluation of drug appropriateness and shared decision making with the patient.  Users  Physician and pharmacist  Patients  Adults ≥70 years with multimorbidity (≥3 chronic conditions) and polypharmacy (≥5 drugs used long term)  Clinical workflow  The STRIP tool was incorporated into the STRIP assistant (STRIPA). It was considered usual care to conduct an unstructured drug review by the attending hospital doctors. | Clinical knowledge  STOPP/START criteria presents a comprehensive list of potentially inappropriate prescriptions for common conditions in older people [2]. STOPP/START criteria are based on compiled lists of well-established potentially inappropriate prescribing criteria and on extensive scientific literature reviews. A Delphi consensus technique was used to establish the content validity of STOPP/START. The criteria reflect the consensus opinion of a panel of experts in geriatric medicine, clinical pharmacology, psychiatry of old age, pharmacy and general practice [2].  Patient data  The intervention was performed at individual patient level and consisted of a structured drug review using STRIP, a process developed to support pharmacotherapy optimisation in older patients. STRIP combines the STOPP/START criteria to detect drug overview and underuse with implicit drug appropriateness assessment methods, such as structured questions on drug history [2]. STOPP consists of 65 indicators that pertain primarily to important drug-drug and drug-disease interactions (potentially leading to side effects such as cognitive decline and falls) and therapeutic duplication. START incorporates 22 evidence-based indicators of common prescribing omissions [2].  Preadmission drug use was assessed with the Structured History taking of Medication (SHiM) questionnaire and entered into STRIPA along with the patient’s current diagnoses and relevant laboratory values.  When generating prescribing recommendations, additional in-hospital information was also taken into account e.g. new diagnoses and history of adverse drug reactions.  Decision rules  Web-based decision support system that takes into account clinically relevant drug-drug interactions, dose adjustment according to renal function, and predictable adverse drug effects.  A trained research doctor and pharmacist generated patient-specific prescribing recommendations based on the STOPP/START criteria, with possible adaptations after discussion with the attending hospital doctor and the patient to take patient preferences into account. | Format  Electronic  Channel  The web-based STRIP Assistant (STRIPA) facilitated the medication review. STRIP can perform multiple tasks intrinsic to pharmacotherapy optimisation simultaneously.  Trigger  -  Presentation   - List of potentially inappropriate prescriptions are presented on-screen | Training of CDS users  All team members conducting the intervention underwent training prior to the beginning of the study.  Implementation strategies  -  Outcome evaluations  - |
| Byrne (2005) | - | - | Setting  Primary care (nursing homes)  Purpose of/rationale for using CDS  “The MDS is a high-quality, validated, and audited data set. The data are collected quarterly or more frequently by all nursing homes and are used for a variety of purposes, including reimbursement, quality measurement, surveys, and generating publicly reported quality measures. MDS data are not typically used internally within the nursing home after submission, and few software applications use the MDS for other clinical purposes, such as analyzing the data longitudinally to predict resident outcomes and generate residentspecific risk profiles. […] The goal of this patient safety research project that served as the basis of this dissertation research were to … provide this risk information and technical support through user-friendly web-based reports that are used by nursing home staff who have traditionally not used much computerized decision support clinical information …” (cited in thesis paper).  Users  Nurses  Patients  Nursing home residents in participating nursing homes in New York State  Clinical workflow  All participating nursing homes were provided with access to the web-based application. The already existing nursing home quality improvement software EQuIP was used to access risk reports. | Clinical knowledge  The CDS includes a medical knowledge base. “Studies have shown that MDS can be used to accurately assess risk for adverse outcomes” (cited in thesis paper). Data used to generate risk scores were based on risk factors for falls and pressure ulcers identified in relevant literature.  Patient data  Patient data were derived from the Minimum Dataset (MDS). These data were used for risk assessment: chronic and infectious diseases, other health conditions, demographics, cognitive functioning, and mobility. “MDS items that are associated with greater as well as reduced risk were all incorporated into the models, unlike most of the logistic regression models found in the literature, where negatively associated factors are usually excluded” (cited in journal paper).  Decision rules  Based on the medical knowledge base and patient data, the CDS had an inference engine to generate case specific advice. Five risk levels were developed: Very High; High; Moderate (these residents have average to High probability of the adverse event); Low; and Lowest. | Format  Electronic  Channel and presentation  The risk reports were available through existing nursing home quality improvement software called EQuIP for Quality (EQuIP). The risk reports should meet the following criteria: decision focused; easy to use by decision makers; user initiated and controlled interactively; emphasize flexibility, adaptability, and quick response; combines the use of models and analytic techniques with traditional data access and retrieval functions.  Trigger  - | Training of CDs users  “Two to three nursing staff from each participating facility were required to attend training seminars as a condition of participation in this project. These usually were the director/assistant director of nursing, MDS coordinator, and/or the director of quality improvement, and sometimes the nursing home administrator.  **Participant Training** – “The initial training sessions presented information required to understand and use the risk reports for quality improvement. The sessions covered:   - How to access resident assessment and concepts; - Basic statistical terminology and concepts; - Explanation of the risk models and risk factors displayed in reports; - How outcome rates are derived; - Interpreting risk and outcome data and using benchmarks; - Applying information to select the areas of care that are a problem; - Examples of how the risk reports could be used to target remediation and interventions; - How to develop and evaluate changes in care processes based on the information; and - Introduction to planned new feedback reports and how to implement them” (cited in thesis paper).   Implementation strategies  All facilities were offered a training video after a year of the project for any new staff that may have joined the project and were provided with documents that presented the project background.  Three update seminars and regular conference calls were conducted to reinforce the participants’ use and application of the resident information for resident care planning.  Outcome evaluations  - |
| Carroll, Dykes & Hurley (2012) | Electronic Fall Prevention Tool Kit (FPTK) software using health information technology (HIT) | Fall prevention tool kit (FPTK) software | Setting  Urban hospitals (academic medical centers & community hospitals)  Purpose of/rationale for using CDS  “Fall risk assessment and health information technology (HIT) have been underused in fall prevention efforts. Fall risk assessment provides a baseline measure of risk status to guide interventions to counteract identified risks. […] The Institute of Medicine reported on the significant number of errors that occur in hospitals and advocates for the use of HIT. HIT improves communication and facilitates information access and decision support. […] We developed a fall prevention tool kit (FPTK) that used a valid fall risk assessment scale as the foundation for a HIT application that provides fall prevention decision support and communication at the bedside.” [3]  Users  Nurses  Patients  All patients admitted to study during study period  Clinical workflow  The FPTK was integrated into existing communication and workflow patterns. | Clinical knowledge  Literature, findings from phase 1 interviews to identify barriers and facilitators to fall risk communication and interventions.  Patient data  The Morse Falls Scale (scores range from 0-125) consists of 6 risk foci: (1) recent history of falling; presence of secondary diagnosis (e.g., >1 medical diagnosis listed in patient record), need for ambulatory aid, receiving intravenous therapy, gait characteristics, and impaired mental status.  Decision rules  Decision rules were based on evidence from the literature and findings from phase 1 interviews. Electronic software that, based on identified fall risk factors, tailored a 3-item output to communicate fall risk alerts and interventions to prevent falls: (1) an over-bed poster; (2) a patient/family education handout; and (3) a plan of care (tailored plan and interventions)  Presentation  Tailored fall prevention interventions using icons to address patients’ fall risk, a tailored poster, patient education handout, and plan of care was printed after approval | Format  Electronic and paper-based  Channel  -  Trigger  -  Availability  -  Presentation   - Alerts printed on paper to hang over bed | Three study phases were conducted by the research team to develop and test components of the FPTK. In phase 1, qualitative inquiry was used to identify barriers and facilitators to fall risk communication and interventions. In phase 2, a prototype FPTK was developed using risk factors of the Morse Falls Scale. In phase 3, an iterative process was used to identify valid icons for the FPTK. The Institute for Healthcare Improvement’s Framework for Spread was used to promote buy-in on the unit level.  Training of CDS users  -  Implementation strategies   - Icons used to address the need for bedside alerts, and to simplify and standardise communication across stakeholders   Outcome evaluations  - |
| Dykes et al. (2010) |  |  |  |  |  |  |
| Clemson et al. (2024) | The Integrated Solutions for Sustainable Fall Prevention (iSOLVE) intervention | iSOLVE decision support tool for GPs | Setting  Primary care practices.  Purpose of/rationale for using CDS  “These decision making  tools enable fall risk assessment and management and are part of a practice resource package for the GP.” (cited in trial protocol, Clemson et al. 2017).  “… an electronic clinical decision support tool will be offered to GP practices involved. This electronic tool is developed as part of the iSOLVE project to integrate the processes into general practice systems and software to facilitate workflow.” (cited in clinical trial registry).  Users  General practitioners.  Patients  Community-dwelling older adults who had had a fall in the past year or were concerned about falling.  Clinical workflow  Stay Independent Questionnaire  The Stay Independent questionnaire was integrated into a tablet intended for practice nurses or reception staff. Once the patient filled in responses to the questionnaire, risk information was automatically sent to the GP software for GPs to view.  Fall risk assessment  “GP managing patient fall risk: The GP uses the patient check list, conducts a risk assessment, and determines a tailored management plan. The management plan is generated automatically if the computer system is used. The GP may review medications and check cataracts or postural hypotension where clinically indicated. The GP also initiates appropriate referrals to local fall services (e.g., allied health and/or community exercise and/or medication review) which specify “fall prevention”.” (cited in trial protocol, Clemson et al. 2017).  Tailoring interventions to fall risk factors  “A new chart, Tailoring Interventions to Fall Risk, was developed which maps risk factors and risk factor profiles to appropriate interventions. This was based on the intervention evidence (e.g., medication review; balance, and strength training) and additionally, where intervention evidence did not exist, was based on modifiable risk factor evidence which strongly supported a guideline for practice (e.g., postural dizziness).” (cited in trial protocol, Clemson et al. 2017).  Patient follow-up  Patient follow-up included revieing patient education, assessing and encouraging adherence to recommended interventions, and discussing and addressing barriers to adherence. GPs were recommended to review their patient’s fall risk annually. | Clinical knowledge  The iSOLVE decision support tool, based on the US STEADI algorithm, is supported by research on risk factors for falling and the effectiveness of multifactorial assessments and interventions to prevent falls.  "Other iSOLVE resources include background information supporting the evidence for interventions; five case studies which each illustrate the algorithm and tailoring options and were validated by a local expert group; a detailed summary of known medications to be a risk of falling; Medicare reimbursement options for GPs; and, examples of “how to talk with patients about falls.”" (cited in trial protocol, Clemson et al. 2017). These resources provide the GPs with knowledge on risk factors for falling and how to address them.  Patient data  Patient data includes fall history the previous year and circumstances of any falls, worry about falling, risk factors for falling (i.e., balance, strength and gait, medication review, vision impairment/cataract, postural hypotension/dizziness/light-headedness, foot pain, urge incontinence, recent hospitalisation, and cognitive impairment).  Decision rules  “Fall or fall risk alert to GP: People who report a fall in the past year or report “yes” to one of the risk questions on the Stay Independent Fall Check list will indicate an alert to the GP who then starts the process of assessment and management. Where practices agree a tablet device will be given to people 65 years and over by the practice nurse in the waiting room and the fall screen completed to assist in determining risk factors. If the tablet is used, this automatically sends the fall risk information to the GP’s software to speed up the process» (cited in trial protocol, Clemson et al. 2017). | Format  Both paper and electronic versions were offered to GPs.  Channel  The offer of both paper and electronic versions of the tool allowed GPs to choose the most appropriate format. The decision support tool was developed in a commercial GP software (PenCS), which come GPs in the area used.  Trigger  If the Stay Independent questionnaire was answered on the tablet, preliminary risk information was automatically sent to the GP. “Once the GP completes the Fall Risk Assessment, the program produces the recommended, individualized, and tailored interventions that match their fall risk.” (cited in trial protocol, Clemson et al. 2017). The computer system automatically generated a management plan.  Presentation  The decision support was presented at the moment of use. | Training of CDS users  “To facilitate integration of the iSOLVE algorithm, face-to-face, one-hour academic detailing will be offered to GPs involved.” (cited in clinical trial registry).  “An individual face-to-face training session is used to educate GPs in the various components of the iSOLVE  intervention including decision support tools, evidence-based interventions, potential referral pathways and fall  prevention strategies. The training is based on academic detailing which is characterized by principles such as  involvement of a “peer” to enable rapport and credibility, concise graphic print materials and applies social  marketing principles to facilitate behavior change.” (cited in trial protocol, Clemson et al. 2017).  Implementation strategies  The decision support tool was developed within a commercial GP software (PenCS third-party software Topbar) that was used by some GPs in the area.  Outcome evaluations  Researchers conducted in-depth interviews with GPs in the experimental group (n = 24, 75%).  In the final 2019 annual survey, a free-text question asked how GPs changed practice and the challenges they encountered. All 204 respondents commented on challenges to providing fall prevention.  It was found that “it was not workable to embed the Decision Tool in the commercial software (Topbar).” (cited in journal article, Clemson et al. 2024). |
| Dykes et al. (2020) | Fall TIPS (Tailoring Interventions for Patient Safety) Toolkit (FTTK) that actively engages patients and family in the three-step fall prevention process | Web-based Fall TIPS Toolkit (FTTK) with | Setting  Hospital (academic medical centres)  Purpose of/rationale for using CDS  “A previous study theorized that fall prevention was a 3-step process: (1) assessing fall risk, (2) developing a personalized prevention plan, and (3) executing the plan consistently. Out team developed the Fall Tailoring Interventions for Patient Safety (TIPS) tool kit, a nurse-led, evidence-based fall-prevention intervention that uses bedside tools to communicate patient-specific risk factors for falls and a tailored prevention plan. The tool kit provides care team members with the information they need to routinely engage in the fall-prevention process. […] A follow-up case-control study suggested that falls within the intervention units were largely attributable to patients’ nonadherence to their fall-prevention plan and that further strategies are needed for engaging patients in the 3-step fall-prevention process during hospitalization” (cited in journal article).  Users  Nurses  Patients  All patients admitted during study period (sensitivity analyses on ≥65 years)  Clinical workflow  Electronic software integrated into the hospital EHR that, based on identified fall risk factors, helps nurse and patient to select personalized, evidence-based interventions, and tailors a bed poster with alerts and a plan of care. | Clinical knowledge  The research team conducted three phases of study to develop and test components of the FPTK: (1) qualitative inquiry to identify barriers and facilitators to fall risk communication and interventions; (2) development of the prototype FPTK by using the Morse Falls Scale (MSF) risk factors; (3) iterative process involving domain experts, end users, and an illustrator to identify valid icons for the FPTK.  Patient data  The MFS consist of 6 risk foci: (1) recent history of falling; (2) presence of secondary diagnosis (e.g., >1 medical diagnosis listed in patient record); (3) need for ambulatory aid; (4) receiving intravenous therapy; (5) gait characteristics; and (6) impaired mental status.  Decision rules  Decision rules of the FPTK were based on evidence from the literature and findings from phase 1 interviews. | Format  Electronic  Channel  -  Trigger  -  Presentation   - Checklist with risk factors and intervention recommendations | The patient-centered fall prevention toolkit was implemented as one component of a larger integrated suite of tools. Predefined RE-AIM metrics were used to verify whether the fall prevention toolkit was working as intended. Potential barriers and changes that could be made were assessed before full implementation.  Training of CDS users  CDS users participated in a six-month pilot testing period where they tested the FPTK on patient care units.  Implementation strategies   - Input from focus groups, interviews, and workflow observations to learn about the needs and preferences of patients and providers and other social-technical factors that relate to fall prevention. - Participatory design, icon validation with patients and families.   Outcome evaluations  In the fifth phase of the project, evaluation of the toolkit incorporating metrics to address each component of the RE-AIM framework was conducted. |
| Elley et al. (2008) | Falls-and-fracture nurse (FFN) coordinator and multifactorial intervention | - | Setting  Primary care (home visits; primary care practices)  Purpose of/rationale for using CDS  “There are reasons why a multifactorial intervention program using a falls-and-fracture nurse coordinator working with family physicians may be highly effective. Family physicians are in an ideal position to identify those at risk and are influential for recruitment and adherence. Most falls result from multiple risk factors, and multifactorial intervention trials in other settings have reduced falls. […] This randomized, controlled trial was designed to determine whether a falls-and-fracture nurse coordinator working with family physicians could reduce falls by assessing and referring at-risk older adults to appropriate interventions using existing health services” (cited in journal article).  Users  Nurses  Patients  Adults ≥75 years who had fallen in the past 12 months  Clinical workflow  - | Clinical knowledge  Evidence-based algorithm that incorporated aspects from the successful PROFET trial, Tinetti’s multifactorial intervention trial, and the individually tailored Otago Exercise Programme.  Patient data  Health assessment: history of circumstances of the fall, medications previous cardiovascular or neurological illness, continence, vision, postural blood pressure, balance and gait, cardiovascular screen (syncope, arrhythmia).  Home hazards assessment: an audit for environmental safety.  Bone health assessment: a brief osteoporosis risk screen, recommendation for family physician assessment to consider vitamin D and calcium supplementation, dual energy X-ray absorptiometry (DEXA) measurement of bone density, and bisphosphonates where indicated.  Decision rules  **Algorithm:** Algorithm that, based on identified fall risk factors, recommends appropriate advice, education, and further referral to another health personnel or other professional.  Criteria for not prescribing the Otago Exercise programme were a Timed Up and Go Test score longer than 30 seconds or a marked neurological impairment. The falls-and-fracture nurse coordinator could refer those excluded to a community physical therapist who tailored an alternative exercise program.  After assessment risk factors, the nurse made referrals and follow-up to relevant health personnel based on findings. The nurse instigated a referral to the regional occupational therapy service if a need for modification was detected using the standard home assessment. | Format  -  Channel  -  Trigger  -  Presentation   - Algorithm with risk factors and intervention recommendations | Training of CDS users  “A falls-and-fracture nurse coordinator with substantial gerontological experience was trained by the clinical investigators and at an established community-based fall-prevention program in Australia (2 days)” (cited in journal article).  Implementation strategies  -  Outcome evaluations  - |
| Ferrer et al. (2014) | Multifactorial fall risk assessment and treatment recommendations made to patient and family physician based on algorithm | - | Setting  Primary care (primary care practices)  Purpose of/rationale for using CDS  “The evidence from randomized trials suggests that there are a number of fall prevention interventions with proven effectiveness. […] Conflicting results on effectiveness of interventions emphasize that the need to know which group of oldest people are the most suitable for assessment. Moreover, various clinical practice guidelines and recent prevention programs recommend multifactorial intervention as a primary treatment strategy. However, none of these assessments contain high-level evidence on all community-dwelling older persons, including those with cognitive impairment” (cited in journal article).  Users  Nurses; physicians  Patients  Community-dwelling older adults born in 1924 (85 years of age at study start)  Clinical workflow | Clinical knowledge  -  Patient data  Modifiable risk factors for falls: Psychotropic and cardiovascular drug use, auditory acuity, visual acuity, balance and gait disorders, risk of malnutrition, disability, cognitive impairment, social risk, and home safety. Nutritional assessment (during the second year).  Decision rules  **Algorithm:** Algorithm that identified nine areas of potentially modifiable fall risk factors and that aided the physician or nurse in giving recommendations, interventions, or referrals according to identified fall risk factors. The algorithm evaluated long-term prescriptions, with special emphasis on significant polypharmacy (five or more prescriptions), progressive discontinuation of benzodiazepines, and nutritional or vitamin supplementation.  “A treatment plan was devised based on the participant’s existing medical care and service networks in the community. For cognitively impaired participants, caregivers were required to be an integral part of the program and ensure that the intervention was implemented. Participants were referred to an ophthalmologist if their worst corrected monocular near vision was less than 0.5/1 decimals on the Jaeger chart. If there was visual field impairment, the patient was advised to alter their lighting at home to improve visibility (high ambient light level, conventional wall-plug night light). Participants with gait disorders were referred to physical therapists for assessment and balance and strength training. […] The algorithm also generated recommendations for treatment of auditory impairment when the participant was unable to hear a whispered voice at approximately 0.6 m, for risk of malnutrition, and for functional or cognitive decline when deemed necessary” (cited in journal article). | Format  -  Channel  -  Trigger  -  Presentation   - Algorithm with risk factors and intervention recommendations | Training of CDS users  The doctor or nurse that visited participants in the intervention group after their baseline assessment to give recommendations according to the algorithm had specialized training in geriatrics.  Implementation strategies  -  Outcome evaluations  - |
| Frankenthal et al. (2014) | Screening medications with STOPP/START criteria followed up with recommendations to the chief physician | Screening Tool of Older Person’s potentially inappropriate Prescriptions and Screening Tool to Alert doctors to Right Treatment (STOPP/START) criteria | Setting  Specialist care (chronic care geriatric facility)  Purpose of/rationale for using CDS  “Little is known about the effect of an intervention involving the application of STOPP/START criteria on clinical and economic outcomes. Only one randomised controlled trial has reported on the clinical effect of the application of STOPP/START on hospitalised older adults” (cited in journal article).  Users  Pharmacist  Patients  Residents ≥65 years prescribed at least one medication  Clinical workflow  - | Clinical knowledge  See description for Blum et al. (2021).  Patient data  STOPP/START criteria to detect potentially inappropriate prescriptions and potential prescription omissions. List of drugs or drug classes and dosages that are known to cause harmful effects (i.e. increased fall risk) and that helps avoiding PIPs and PPOs  Decision rules  “Interventional recommendations that the study pharmacist made for residents in the intervention group but not in the control group were discussed with the chief physician at study opening and after 6 months. The chief physician decided whether to accept these recommendations and implement prescribing changes” (cited in journal article). | Format  Electronic  Channel  -  Trigger  -  Presentation   - Lists of FRIDs (STOPP/START criteria) | Training of CDS users  -  Implementation strategies  -  Outcome evaluations  - |
| Gallagher, O’Connor & O’Mahony (2011) | Screening with STOPP/START criteria followed up with recommendations to patients’ attending physicians | Screening Tool of Older Person’s potentially inappropriate Prescriptions and Screening Tool to Alert doctors to Right Treatment (STOPP/START) criteria | Setting  University hospital  Purpose of/rationale for using CDS  “Easily delivered strategies for ensuring appropriate prescribing in older patients are needed. One such strategy would be the routine clinical application of explicit indicators of potentially inappropriate use of medications, such as the recently validated STOPP […] and START […] criteria” (cited in journal article).  Users  Physicians  Patients  Patients aged ≥65 years admitted via the emergency department under care of a GP  Clinical workflow | Clinical knowledge  See description for Blum et al. (2021).  Patient data  STOPP/START criteria to detect potentially inappropriate prescriptions and potential prescription omissions. List of drugs or drug classes and dosages that are known to cause harmful effects (i.e. increased fall risk) and that helps avoiding PIPs and PPOs. STOPP consists of 65 indicators that pertain primarily to important drug-drug and drug-disease interactions (potentially leading to side effects such as cognitive decline and falls) and therapeutic duplication. START incorporates 22 evidence-based indicators of common prescribing omissions [2].  Decision rules  PIPs and PPOs identified based on the baseline data of patients in the intervention group were immediately discussed with the attending medical team. Recommendations were then made to the attending physicians of the patients. The physicians chose whether or not to accept the recommendations. | Format  -  Channel  -  Trigger  START/STOPP criteria were applied to baseline data of patients after baseline assessment and randomisation.  Presentation  Lists of FRIDs (STOPP/START criteria) | Training of CDS users  -  Implementation strategies  -  Outcome evaluations  “The interrater reliability of the international recommendations was tested through a study in which nine physicians practicing independently in six European countries applied STOPP/START criteria to 20 cases comprising 181 medications” (cited in journal article). |
| Ganz et al. (2015) | Assessing Care of Vulnerable Elders Practice Redesign for Improved Medical Care for Elders (ACOVEprime) | Decision support to prompt primary care providers to take appropriate action in response to a positive screen | Setting  Primary care (primary care practices)  Purpose of/rationale for using CDS  “The theory behind the [ACOVE-2] intervention was to develop a low-tech, practical restructuring of care delivery in the primary care office that follows the principles of the Chronic Care Model” (cited in trial protocol).  Users  Physicians  Clinical knowledge | Clinical knowledge  **-**  Patient data  **-**  Decision rules   - Condition-specific information for patients and families, tools to guide self-management, materials to enhance adherence (e.g. diaries), and community-specific resources. - **Medical record prompts:** to support primary care providers in to take appropriate action for patients at high risk of future falls (using paper-based structured visit note templates or computerized electronic health record prompts) - Physician decision support and physician education | Format  Electronic  Channel  -  Trigger  -  Presentation  Condition-specific structured visit notes designed to lead physicians through the appropriate care processes for the target condition. | Training of CDS users  Geriatricians with expertise in management of the conditions taught physicians how to modify their practices to incorporate the intervention components.  Implementation strategies  The project involved a quality improvement component including physicians abstracting a sample of their own medical records at the beginning of the project so that they could review the care they had provided. Also, a run-in phase was used to permit practices to test their practice redesign model and make changes. Approximately six months after implementing the intervention, physicians performed a second char taudit and subsequently received feedback reports comparing their baseline and second chart audit data.  Practice leaders and physicians were presented with the intervention components and given the opportunity to indicate how they thought the intervention might work at their site.  Outcome evaluations  - |
| Wenger et al. (2010) |  |  |  |  |  |  |
| Groshaus et al. (2012) | Multi-component knowledge translation intervention that incorporated a nurse-initiated clinical decision support tool | Nurse-initiated clinical decision support tool | Setting  Acute care hospitals  Purpose of/rationale for using CDS  “Clinical decision support provided to physicians has been found to improve practitioner performance and possibly patient outcomes. The impact on nursing practice is less clear. […] [The intervention] incorporated a clinical decision support tool to reduce harms in the care of older medical inpatients. The intervention targeted the care nurses provide to older medical patients in acute care hospitals. We used two frameworks to inform this activity” [4].  Users  Nurses  Patients  Patients ≥65 years residing on study units  Clinical workflow  Workflow impact and sustainability were considered | Clinical knowledge  **Electronic order set:** Electronic nurse-initiated geriatric order set containing fall risk screening and recommended fall prevention interventions. The order set was developed by a multi-disciplinary team using evidence obtained from published clinical trials, systematic reviews and practice guidelines addressing delirium, falls, continence, nutrition and hydration.  Patient data  -  Decision rules  -  Presentation  - | Format  Electronic  Channel  The electronic order set was made available within the hospitals’ EMR.  Trigger  -  Availability  Nurses could access the order set whenever there was a need from within the EMR. At the start of the 12-week rollout on each unit, the order set was available to all users but the unit nurses were not informed about its presence until the educational in-servicing. | MRC framework for complex interventions and Knowledge to Action Cycle were used in the implementation of the inervention.  Training of CDS users  -  Implementation strategies   - Several continuing education strategies about care for older patients, binder of geriatric resource materials. Education about relevant age related changes and evidence-informed strategies to provide optimal care to the older in-patient. - Reminders   Outcome evaluations   - Rate of use of the electronic order set - Qualitative interviews with unit nurses to explore issues with sustainability of the intervention, impact on workload, perceived impact on patient care and barriers and facilitators to use of the CDS |
| Healey et al. (2004) | Targeted risk factor reduction core care plan | Targeted risk factor reduction core care plan | Setting  District general hospital  Purpose of/rationale for using CDS  -  Users  Nurses  Patients  All older adults ≥65 years who received care in the wards during the study period  Clinical workflow  This CDS tool was new to study hospitals: “Prior to the study none of the wards carried out specific fall assessments or interventions, and investigators such as lying and standing blood pressure or ophthalmology referral occurred on an ‘ad hoc’ basis. […] The falls care plan was interleaved with accident reporting forms already in routine use on the wards throughout the study period, to prompt its use when ‘near misses’ or falls occurred” (cited in journal article). | Clinical knowledge  The backside of the pre-printed care plan contained a brief summary of evidence, such as medication most likely to be implicated in falls.  Patient data  Eyesight; medication; lying and standing blood pressure; ward test urine, difficulty with mobility, environmental check, review risk/benefit of bedrails for individuals, footwear safety, bed height, position in ward, simple environmental cause of falls (e.g. loose cable, wet floor), nurse call bell  Decision rules  To focus the intervention on patients with higher fall risk, nursing staff on intervention wards were asked to apply the intervention to patients admitted with a history of falls, those who had fallen or had a ‘near miss’ during their current admission.   - Eyesight: if unable to recognise pen/key/watch from two meters distance, optician visit If lost glasses, referral in no known reason for poor eyesight. - Medication review of prescription benefit related to falls risk. - Lying and standing blood pressure: Refer any deficit to medical staff. Advise patient on changing position slowly. - Ward test urine: Send mid-stream urine sample if positive for nitrites, blood or protein. - Refer to physiotherapist if difficulty with mobility. - Document risk/benefit of bedrails in nursing notes and removal or addition of bedrails as appropriate - Advise relatives on replacement of footwear - Keep bed at lowest height - Position in wards: Nurse patient with history of falls as close to nurses station as possible (considering other patients’ needs) - If simple environmental cause of falls, act to correct it. - Explain nurse call bell and put within reach | Format  Paper-based  Channel  -  Trigger  Nurses were prompted to use the falls care plan when ‘near misses’ or falls occurred.  Presentation  - | Training of CDS users  No specific training was provided – the nurses already used pre-printed care plans for other conditions, and the simple format made this unnecessary.  Implementation strategies  -  Outcome evaluations  “The study did not attempt to measure or enforce use of the care plan for all appropriate patients” (cited in journal article). |
| Lightbody et al. (2002) | Nurse-led fall prevention management plan and care pathway for older people | - | Setting  University hospital  Purpose of/rationale for using CDS  -  Users  Nurses  Patients  Older adults ≥65 years discharged from the Accident and Emergency Department after a fall  Clinical workflow  The intervention made use of existing resources and mechanisms to make it implementable and sustainable. | Clinical knowledge  Risk factors for falls were obtained from relevant literature.  Patient data  Modifiable risk factors for falls: Assessments of medication, ECG, blood pressure, cognition, visual acuity, hearing, vestibular dysfunction, balance, mobility, feet and footwear. Environmental assessment identified inadequate lighting, tripping hazards and unsuitable furniture.  Decision rules  All patients were given advice and education about general safety in the home. Risk factors requiring further action were referred to relatives, community therapy services, social services and/or the primary care team. Direct referrals were not made to hospital outpatients or day hospital. | Format  -  Channel  -  Trigger  -  Presentation  Checklist presenting risk factors for falls and recommendations. | Training of CDS users  Nurses were given basic training.  Implementation strategies  -  Outcome evaluations  **-** |
| Logan et al. (2021) | The Guide to Action Care Homes (GtACH) Falls Prevention Programme | GtACH risk assessment and checklist | Setting  Long-term care homes for older people  Rational for using CDS  “Owing to care home staﬀ being relatively untrained, the complex nature of risk factors for falls in care  residents, and the need for several interventions to deal with multiple risk factors, a systematic care home wide programme including staﬀ education and support in the use of risk assessment and decision support tools was required. The GtACH programme is a systematic approach developed using literature, clinical expertise, and the views of care home residents and families, care home staﬀ, and researchers. The theory was that staﬀ are key to reducing fall rate in care facilities and that by numerous incremental actions, such as improved lighting, greater access to appropriate drinks, timely drug reviews, and monitoring the pattern of falls then the eﬀect on an individual will be seen” (cited in journal article).  Users  Care home staff  Patients  Long-term care home residents in care homes for older adults ≥65 years  Clinical workflow | Clinical knowledge  “The GtACH programme is […] developed using literature, clinical expertise, and the views of care home residents and families, care home staff, and researchers.  Patient data  The GtACH screening and assessment tool comprised 33 items related to falls risk factors grouped into four domains: falls history, medical history, movement/environment, and personal needs.  Decision rules  Staff were to use the GtACH risk assessment and checklist for all residents and produced a written action plan for each resident. Actions for dehydration could be introducing smoothies, offering fruit juice more often, adapting crockery to take account of disabilities, producing soups, and making an event of coffee time. The presence of risk factors prompted up to 30 individual staff actions. | Format  Paper-based  Channel  -  Trigger  -  Presentation  Falls screening and assessment tool that consisted of a checklist presenting risk factors for falls and recommendations. | Training of CDS users  One hour of training face-to-face for all care home staff (including gardeners, caretakers, cooks, cleaners, managers) in small groups, delivered by a falls specialist. During training a power point presentation, case studies and role play were used. After training, a manual summarising the GtACH programme and including resources such as a falls incident chart (to detect patterns) and a drug falls risk chart is left in the care home. Repeated training sessions were offered to reach all staff, including managers. The training aimed to increase awareness and knowledge about the management of falls.  Implementation strategies   - The GtACH intervention was co-designed and tested in a feasibility randomised controlled trial. - Falls champion: A member of care home staff was allocated to the role of falls champion, responsible for training new staff and embedding the GtACH programme.   Outcome evaluations |
| Mahoney et al. (2007) | Intermediate-intensity, community-based multifactorial falls intervention | Algorithm based on the University of Wisconsin Falls Prevention Clinic | Setting  Primary care (home visits)  Purpose of/rationale for using CDS  “There is a need to evaluate practical, intermediate-intensity, multifactorial models for their efficacy in reducing falls in the community. An intermediate-intensity multifactorial model was defined as one that provides primarily referrals and recommendations without delivering physical therapy or progressive exercise but with a greater number of contacts with the older adults than a low-intensity approach (>4)” (cited in journal article).  Users  A nurse and a physiotherapist.  Patients  Community-dwelling adults ≥65 years with two falls in the past year or one fall in the previous two years with injury or balance problems  Clinical workflow  **-** | Clinical knowledge  The algorithm is based on the University of Wisconsin Falls Prevention Clinic.  Patient data  Predisposing factors for falls; induce risk reduction changes in medical conditions, medications, behavior, distant vision, balance and gait, some neurological deficits, cognition, mood, home functioning, and home safety.  Decision rules  **Algorithm** used by nurse or physical therapist for assessment and identification of fall risk factors, along with triggers for further action and suggestions for appropriate fall prevention interventions. “The algorithm generated referrals and recommendations to physical therapy and other healthcare providers based on specific criteria. Required triggers for physical therapy referral included moderate impairment on Berg Balance Scale items, abnormal gait on the Performance Oriented Mobility Assessment, inability to stand for 30 seconds with eyes open on hard surface or foam, and history of pain with walking or doing exercise. Optional triggers included loss of balance with sternal nudge, positive Romberg test, absent vibratory sensation at the ankle or metatarsal phalangeal joint, inability to stand for 30 seconds with eyes closed on hard surface or foam, total score less than 80 on the Activities Specific Balance Confidence Scale, and any potentially risky mobiliy-related activity of daily living (ADL) or instrumental activity of daily living (IADL), per assessor’s judgement […] If the algorithm did not recommend physical therapy, then the study therapist provided a set of balance and leg strengthening exercises. The assessment generated referrals for further medical evaluation and treatment” (cited in journal article). Recommendations were mailed to subjects’ primary physicians. | Format  Electronic  Channel  -  Trigger  -  Presentation  - | Training of CDS users  The nurse or physical therapist received three days of additional training in a standardised fashion from a geriatrician and physical therapist on algorithm’s multidisciplinary components.  Implementation strategies  -  Outcome evaluations  - |
| Peterson et al. (2007) | Guided dosing within a computerized provider order entry (CPOE) presented to physicians | Guided dosing within a computerized provider order entry (CPOE) presented to physicians | Setting  Specialist care (hospital)  Purpose of/rationale for using CDS  “Guided dosing within a computerized provider order entry (CPOE) system is an effective method of individualizing therapy for patients. Physicians’ responses to guided dosing decision support have not been extensively studied. As part of a randomized trial evaluating efficacy of dosing advice on reducing falls in the elderly, CPOE prompts to physicians for 88 drugs included tailored messages and guided dose lists with recommended initial doses and frequencies” (cited in journal article).  Users  Physicians  Patients  Inpatients ≥65 years receiving care on one of the order entry wards (i.e., emergency room, intensive care units, subacute units)  Clinical workflow  “The system utilized the same computational infrastructure that project members had previously implemented for specialized age- and weight-based pediatric dosing” (cited in journal article). | Clinical knowledge  Based on the Beers Criteria for potentially inappropriate prescribing of medications in older adults. “Authors derived study-related dosing information for FDA-approved indications from “package insert” monographs, and for “off-label” indications from the medical literature and published textbooks” (cited in journal article).  Patient data  Medications: Sedatives, neuroleptics, anti-emetics, and skeletal muscle relaxants  Decision rules   - Guided medication dosing: Advice was electronically delivered to physicians about appropriate initial dosing for sedatives, neuroleptics, anti-emetics and skeletal muscle relaxants. - No barriers prevented selecting higher doses than recommended. Instead | Format  Electronic  Channel  CPOE-based text messages communicated titration strategies, possible adverse effects, and key monitoring parameters were displayed along with dosing information.  Trigger  CPOE prompts: CPOE prompts to physicians for 88 drugs associated with increased fall risk.  Presentation   - Presented on-screen - Medication lists and doses were displayed on a computer editor screen. | Training of CDS users  Users had 10 years of experience using the CPOE system, which they developed themselves.  Implementation strategies  -  Outcome evaluations  - |
| Phelan et al. (2024) | The STOP-FALLS intervention. | Evidence-based pharmaceutical opinions (EBPOs) and deprescribing pearls. | Setting  Eighteen primary care clinics part of the Kaiser Permanente Washington (KPWA) integrated group practice in Washington, USA.  Purpose of/rationale for using CDS  Healthcare providers and patients may not be aware that medications can cause falls, patients may believe they need medications, and providers may be reluctant to change prescriptions even in the face of patients prompting the discussion.  Users  Physicians in primary care practices.  Patients  Community-dwelling adults aged ≥ 60 years, prescribed at least 1 medication from any of 5 targeted medication classes (opioids, sedative-hypnotics, skeletal muscle relaxants, tricyclic antidepressants, and first-generation antihistamines for at least 3 consecutive months.  n = 2 367 (1 106 in CDS and 1 261 in CG), 63% female, mean 70.6.  Clinical workflow | Clinical knowledge  The evidence-based pharmaceutical opinion is modelled after those of the D-PRESCRIBE trial, and describe the risks associated with the targeted medication class, alternative evidence-based treatments that could be tried to help a participant reduce their use of the medication, and hyperlinks to practice supports for deprescribing (e.g., pharmacy consultation mental health referral, and self-care support tools).  The content of the deprescribing pearls was developed by STOP-FALLS investigators based on the published literature. Each pearl gives several examples of how to broach discussions of deprescribing with patients, referred to as “conversation starters”. The pearls were modelled after “clinical pearls” used by KPWA to disseminate clinical information updates and thus are anticipated to feel familiar to providers.  Patient data  The source of patient data on medication prescriptions is electronic health records.  Decision rules  Changes to medications are at the discretion of the participant and their primary care provier. | Format  Electronic.  Channel  The evidence-based pharmaceutical opinions were delivered to the clinicians via the electronic health records, which are always up and running. The deprescribing pearls were delivered via periodic emails to the clinici’s study champion (clinician volunteer or clinic director), who disseminated these materials as they saw fit (e.g., via email).  Trigger  No trigger.  Presentation  The evidence-based pharmaceutical opinions are summarised in a format akin to an infographic, i.e., as visual representations of information about a particular class of CNS-active or other high-risk medications.  The deprescribing pearls are presented in short text format, one topic per pearl for a total of 13 pearls. The topics include, among others, medicines linked to falls, sedative-hypnotics, opioids, over-the-counter sleep aids, skeletal muscle relaxants, and deprescribing and the patient-provider relationship. The pearls include “conversation starters”, i.e., short phrases to facilitate deprescribing discussions. | Training of CDS users  Prior to intervention implementation, each clinic received a 30-min presentation on the study methods and patient and provider materials, with an emphasis that changes to medication prescriptions were up to their clinical discretion.  Implementation strategies  “The intervention encourages but does not require behavior change on the part of the participant or their PCP. However, participant and PCP communication about  the material and medication changes may be considered proxy responses to the intervention. We will closely  examine medication prescriptions and instructions for discontinuation and tapering within the medical chart. We will also examine postcards returned by patient participants regarding if they intend to discuss materials  with their PCP. See “Statistical analysis” for details.” (cited in trial protocol).  Outcome evaluations  - |
| Snooks et al. (2014) | Computerised Clinical Decision Support on hand-held Tablet computers used by paramedics attending older people who fall | Computerised Clinical Decision Support on hand-held Tablet computers used by paramedics attending older people who fall | Setting  Specialist care (emergency ambulance services)  Purpose of/rationale for using CDS  “Demand for immediate care through emergency ambulance services has been steadily increasing […] However many callers have no clinical need for treatment or investigation at an Emergency Department. Although health policy in the UK encourages emergency ambulance services to offer alternatives to such callers, there is little evidence about the effectiveness, safety or cost-effectiveness of clinical assessment by paramedics and triage to other care pathways. Computerised clinical decision support (CCDS) is effective in changing practice in other fields, but there is little evidence about its costs and benefits in emergency care” (cited in journal article).  Users  Paramedics  Patients  Community-dwelling older adults ≥65 years living in the catchment area of a participating falls service  Clinical workflow  One site implemented the CCDS simultaneously with a system for electronic patient data capture. The other site already had an electronic data capture system in place, so instead added CCDS software to the existing system. Neither site fully integrated CCDS with the electronic software due to technical issues. | Clinical knowledge  The CDS tool was based on a system that has successfully been used in previous research with ambulance and out of hours services, and that was modified through the T&R and Fit to be Left studies.  Patient data  Assessment of clinical and social needs  The CDS prompted the assessment and examination of:   - injuries associated with the fall - co-morbidity (e.g. breathlessness or chest pain) - the patient’s psycho-social needs (e.g. mental state and ability to undertake ADL) - environmental risk   Decision rules   - Algorithm in the form of a software integrated into the electronic data capture system that prompts assessment and examination. Based on the findings, the electronic software suggests an appropriate care plan and whether the patient should be taken to A&E or offered an alternative care plan. - For every ambulance service call, an electronic record was created. | Format  Electronic  Channel  -  Trigger   - **Prompts** for paramedics to start assessment. - On-scene assessment and care of older people who have fallen and have called the emergency ambulance service. - When the crew member attends a patient that meets the inclusion criteria, the additional functionality of the CDS for assessing falls will be used to assess whether the older person should be taken to A&E or offered an alternative care plan.   Presentation   - Presented on-screen on a hand-held tablet computer. - The traditional ambulance service Patient Report Form was replaced with a digital record on the Tablet PC. | Training of CDS users  A participative approach was taken with crews and managers from the outset to ensure that the practical details of the development and implementation of the intervention maximise the chance of uptake and success. Paramedics received two additional days of training in the use of the CDS. Both clinical and technology based training was given. Following initial training there was a pre-trial period of one month during which trained crews were expected to practise using the hand-held computer in place of paper Patient Record forms.  Implementation strategies  Outcome evaluations  Recommendations of the Medical Research Council for evaluating complex interventions to improve health. Operational ‘process’ indicators were evaluated: on-scene times, job cycle times – from 999 call to ‘ambulance free’ time. |
| Tamblyn et al. (2012) | Computerized prescribing decision support presenting patient-specific risk of psychotropic drug-related injury to family physicians | Same as intervention name | Setting  Primary care (primary care practices)  Purpose of/rationale for using CDS  “Effective management of psychotropic medication is challenging. […] Computerized prescribing and decision support are expected to address preventable medication errors, as these digital technologies can guide dosing and provide alerts on drug treatment duplication, contraindications, and drug interaction errors, especially when integrated with information on all dispensed medication. […] In this study, we tested the hypothesis that the incorporation of patient-specific risk estimates into a computerized prescribing decision support system in primary care would increase physician respone to alerts for psychotropic medication and reduce the risk of psychotropic drug-related injury in older adults, particularly for patients with a higher baseline risk” (cited in journal article).  Users  Physicians  Clinical workflow  The provincial insurance agency’s (RAMQ) databases containing patient data were linked to the experimental community-based clinical information system MOXXI, which was already in use in the primary care practices. MOXXI was used to link the data from the databases to the electronic health record system used by physicians to support clinical decision-making. | Clinical knowledge  Beneficiary, medical billing and pharmacy claims data from the provincial insurance agency (RAMQ) have been validated and are often used for health services and epidemiological research. The alert used a published predictive model to estimate the risk of injury based.  Patient data  Patient’s age, sex, injury history, presence of cognitive impairment, gait, and balance problems, and doses of selected psychotropic medication (selective serotonin / nor-epinephrine reuptake inhibitors antidepressants, antipsychotics, low-, intermediate- -and high-potency opiates, intermediate- and long-acting benzodiazepines, anticonvulsants, and first-generation antihistamines).  Decision rules   - A commercial drug alert system automatically reviewed each new prescription for potential contraindications, including therapy duplication, dosing error, cumulative toxicity, and drug-disease, drug-drug, and drug-allergy interactions. - **Alerts:** Physicians received a patient-specific risk of injury alert when a patient was prescribed a psychotropic medication that increased the risk of injury. Physicians could set the threshold for the alert system to (1) sever alerts only, (2) moderate and severe alerts, or (3) all alerts. A threshold of an increase in risk of 1 per 1000 was set. - Graphics: Physicians were presented with ‘risk thermometers’ showing the patient’s risk of injury the next 12 months based on the chosen medication combination, type and dose. - Guided medication dosing: When the physician changed medication dose, the absolute and relative change in injury risk would be shown. | Format  Electronic.  Channel  Drug alerts and recommendations were displayed on-screen, and physicians were able to interact with the system.  Trigger   - Alert presented when a patient was prescribed a psychotrpic medication that increased the risk of injury.   Presentation  On-screen presentation of alerts and recommendations and interaction with system. All alerts generated for a patient were available for the physician to review in a drug alert summary in the patient’s electronic chart. Physicians were presented with the numeric values generated by the risk calculated. “If the physician attempted to reduce the risk of injury by stopping or decreasing the dose of a psychotropic medication, the absolute and relative reduction in risk would be shown as an adjustment in the level of the thermometer and a change in the numeric values. If a new psychotropic drug was started or the dose was increased, the absolute and relative increase in the risk would be shown. […] If no change in medication was instituted (or the risk was increased by a medication change), physicians had to select a reason for the decision from a standardised pick-list … […] A reference section was available with publications on the risk of injury related to psychotropic drug use and methods of tapering benzodiazepines” (cited in journal article). | Training of CDS users  “5-minute training program on the risk of injury alert that outlined how to interpret the risk thermometer information, the expected changes in risk with new or discontinued medication, the completion of reasons for not changing therapy if applicable, and the location of reference information. Physicians were advised that stopping or reducing the dose of highlighted medications in the patient’s drug profile would reduce the patient’s risk of injury” (cited in journal article).  Implementation strategies  -  Outcome evaluations  - |
| Weber, White & McIlvried (2008) | Standardized medication review with recommendations to primary care physician via the electronic medical record | - | Setting  Primary care (primary care practices)  Purpose of/rationale for using CDS  “Over the next 30 years, the United States will age dramatically, with rapid growth in the elderly population. The economic and social consequences of this trend will create an urgent need to develop innovative strategies to manage chronic illness and maximize quality of life in older persons. Many health care systems are implementing electronic medical records (EMRs); it is hoped that such systems could identify patients at risk for certain disease states and assist in providing routine processes  of care for chronic diseases” (cited in journal article).  Users  Clinical pharmacist and physicians  Patients  Community-dwelling patients ≥70 years at risk for falls  Clinical workflow  Geisinger Health System (GHS) is an integrated system that possessed a fully integrated ambulatory EMR. The EMR contains all information regarding patients’ care, including medications, radiologic and laboratory studies, times and dates of appointments with all system providers, documentation of all phone calls between the office and patients, and documentation for each encounter. | Clinical knowledge  -  Patient data  The focus of the medication review was use of medications that would increase the risk for falls: psychoactive medications, the presence of polypharmacy, and the presence of medications at inappropriate doses.  Decision rules   - **Guided medication dosing**: A clinical pharmacist or MD fellowship-trained geriatrician reviewed the patient’s medication record via the EMR with a focus on fall-risk-increasing drugs. - **Alert/message**: After the medication review, a message was sent to the patient’s physician that alerted the physician of the patient’s fall risk, and that contained recommendations for fall prevention, and a referral to an evidence-based guideline for fall prevention. - **Guideline**: Patient’s physician was referred to an evidence-based fall prevention guideline that the physician could access directly through the EMR. | Format  Electronic  Channel  -  Trigger   - Alert   Presentation  Clinical pharmacist reviewed the patient’s medication record via the EMR. Message sent to the primary care physician via the EMR, presented on-screen. The message alerted the physician of the patient’s fall risk and presented recommendations tailored to the patient. | Training of CDS users  The geriatrician was MD fellowship-trained.  Implementation strategies  Opinion leaders  Outcome evaluations  - |
| Wenger et al. (2009) | Assessing Care of Vulnerable Elders 2 (ACOVE-2) |  | Setting  Primary care (primary care practices)  Purpose of/rationale for using CDS  “In an effort to improve the quality of care for geriatric conditions provided by primary care physicians, a practice change intervention aimed at confronting common obstacles to physician behavior change (physician knowledge deficits; disbelief that specific care processes will produce better outcomes; reliance on the influences of peers rather than the medical literature; and perceived lack of time, resources, and self-efficacy) was developed. The intervention integrated care improvement for falls and gait impairment, urinary incontinence, and cognitive impairment into daily clinical practice so that these conditions could be treated in concert with older patients’ coexisting conditions” (cited in journal article).  Users  Physicians  Patients  Community-dwelling patients aged 75 and older who had at least one of three geriatric conditions: falls and gait impairment, urinary incontinence, and cognitive impairment  Clinical workflow  **-** | **Clinical knowledge**  The intervention components are based on the quality indicators covering 22 conditions important for older persons developed in the ACOVE project [5].  Patient data  **Initial screen**: Patients were screened with the following questions:   - Have you fallen two or more times? Have you fallen and hurt yourself? - Are you afraid that you might fall because of balance or walking problems? - Do you have a problem with urinary incontinence (or your bladder) that is bothersome enough that you would like to know more about how it could be treated?   Patients were also asked to recall three items after a hiatus of at least 60 seconds.  **For high risk patients**: Condition-specific clinical data, e.g. urinalysis and culture or orthostatus blood pressure), data needed for essential care processes for the specific condition.  Decision rules   - **Medical record prompts**: For patients who screened positive to any of the conditions, a condition-specific intervention was initiated with a structured visit note placed on the medical record. Prompts supported primary care providers to take appropriate action for patients at high risk of future falls. | Format  Paper-based  Channel  -  Trigger   - **Medical record prompts** - A positive answer to any of the screening questions   Presentation   - Suggestions for appropriate actions - Note on screened patients’ charts: Notes indicated patients’ responses to the screening questions | Training of CDS users  Physicians were educated during a three-hour educational group session led by a geriatrician that demonstrated an efficient approach to each condition. Brief, written decision support information was provided that described the management of each condition.  Implementation strategies   - Multicomponent practice-change effort that aimed to guide care for each condition to achieve processes of care identified by the ACOVE quality indicators - Use of structured visit note to stimulate data collection   Outcome evaluation |

A&E: Accident & Emergency department; CPOE: Computerized Provider Order Entry; EHR: Electronic Health Record; EMR: Electronic Medical Record; PIPs: Potentially Inappropriate Prescriptions; PPOs: Potential Prescription Omissions;

References

1. Aizen E, Lutsyk G, Wainer L, Carmeli S. Effectiveness of individualized fall prevention program in geriatric rehabilitation hospital setting: a cluster randomized trial. Aging Clin Exp Res [Internet]. 2015;27:681–8. Available from: https://doi.org/10.1007/s40520-015-0330-7

2. Gallagher P, Ryan C, Byrne S, Kennedy J, OMahony D. STOPP (Screening Tool of Older Person&#146;s Prescriptions) and START (Screening Tool to Alert doctors to Right Treatment). Consensus validation. Int J Clin Pharmacol Ther [Internet]. 2008;46:72–83. Available from: http://www.dustri.com/article_response_page.html?artId=1674&doi=10.5414/CPP46072&L=0

3. Dykes PC, Carroll DL, Hurley A, Lipsitz S, Benoit A, Chang F, et al. Fall prevention in acute care hospitals: a randomized trial. JAMA [Internet]. 2010;304:1912–8. Available from: http://www.ncbi.nlm.nih.gov/pubmed/21045097

4. Groshaus H, Boscan A, Khandwala F, Holroyd-Leduc J. Use of Clinical Decision Support to Improve the Quality of Care Provided to Older Hospitalized Patients. Appl Clin Inform [Internet]. 2012;03:94–102. Available from: http://www.thieme-connect.de/DOI/DOI?10.4338/ACI-2011-08-RA-0047

5. ACOVE Quality Indicators. Ann Intern Med [Internet]. 2001;135:653. Available from: http://annals.org/article.aspx?doi=10.7326/0003-4819-135-8_Part_2-200110161-00004

6. Van de Velde S, Kunnamo I, Roshanov P, Kortteisto T, Aertgeerts B, Vandvik PO, et al. The GUIDES checklist: development of a tool to improve the successful use of guideline-based computerised clinical decision support. Implement Sci [Internet]. 2018;13:86. Available from: https://implementationscience.biomedcentral.com/articles/10.1186/s13012-018-0772-3

7. Medlock S, Wyatt JC, Patel VL, Shortliffe EH, Abu-Hanna A. Modeling information flows in clinical decision support: key insights for enhancing system effectiveness. J Am Med Informatics Assoc [Internet]. 2016;23:1001–6. Available from: https://academic.oup.com/jamia/article/23/5/1001/2379820
