# Supplementary material for: Effectiveness of clinical decision support in fall prevention among older adults: A systematic review and meta-analysis
Source: PLoS One. 2026 Jan 12;21(1):e0340025. doi: 10.1371/journal.pone.0340025 (PMC12795367; doi:10.1371/journal.pone.0340025)
Supplement: S7 Table — (PDF) [file pone.0340025.s007.pdf]

Risk of bias assessments for cluster-randomised trials

Unique ID	Aizen(2015)_A	Study ID	5474	Assessor	RS
Ref or Label		Aim	assignment to intervention (the 'intention-to-treat' effect)		
Experimental	Targeted multiple intervention falls prevention program based on patient's fall risk	Comparator	Usual care	Source	Journal article(s)
Outcome	Fall rate per 1000 bed-days in intervention group compared with control group (of participants admitted during the first phase of the study, three months of follow-up)	Results	Adjusted hazard ratio 1.36 (95% CI 0.87, 1.77; p = 0.08)	Weight	

Domain	Signalling question	Response	Comments
Bias arising from the randomization process	1a.1 Was the allocation sequence random?	PY	"Randomization was carried on with a partial stepped wedge design in a way that all clusters eventually were included in the investigation to allow clusters to be enrolled gradually over time" (journal article).
	1a.2 Was the allocation sequence concealed until clusters were enrolled and assigned to interventions?	NI	
	1a.3 Did baseline differences between intervention groups suggest a problem with the randomization process?	N	"Wards participated in the study were general geriatric rehabilitation wards with very similar mix of patients as can be seen in Tables 1 and 2. Two to three wards were included in each cluster" (journal article).
	**Risk of bias judgement**	**Some concerns**	
Bias arising from the timing of identification or recruitment of participants	1b.1 Were all the individual participants identified and recruited (if appropriate) before randomization of clusters?	**N**	"Written informed consent was obtained from participants and/or legally authorized representatives only in the active arm before starting the intervention" (journal article).<br><br>Individual participants were identified and recruited after randomisation of clusters. Hospital wards were randomised to the intervention or control group, and patients admitted during the study period were approached and asked to participate.
	1b.2 If N/PN/NI to 1b.1: Is it likely that selection of individual participants was affected by knowledge of the intervention assigned to the cluster?	**PN**	It is considered unlikely that persons responsible for admitting patients to wards were influenced by knowledge of the intervention assigned to the cluster in their decisions.
	1b.3 Were there baseline imbalances that suggest differential identification or recruitment of individual participants between intervention groups?	**It is considered unlikely that persons responsible for admitting patients to wards were influenced by knowledge of the intervention assigned to the cluster in their decisions.**	N
	**Risk of bias judgement**	**Low**	
Bias due to deviations from intended interventions	2.1a Were participants aware that they were in a trial?	Y	Participants in the intervention wards were approached for informed consent.
	2.1b If Y/PY/NI to 2.1a: Were participants aware of their assigned intervention during the trial?	Y	Nursing staff in intervention wards were aware of the wards' assigned intervention.
	2.2 Were carers and people delivering the interventions aware of participants' assigned intervention during the trial?	Y	
	2.3 If Y/PY/NI to 2.1b or 2.2: Were there deviations from the intended intervention that arose because of the trial context?	PN	"Participants in the control groups continued to receive usual care which consists of any activities undertaken by the participants recommended or administered by their treating team and did not receive any of the interventions from the falls prevention program." (journal article)
	2.4 If Y/PY to 2.3: Were these deviations likely to have affected the outcome?	NA	
	2.5 If Y/PY/NI to 2.4: Were these deviations from intended intervention balanced between groups?	NA	
	2.6 Was an appropriate analysis used to estimate the effect of assignment to intervention?	NI	Unable to find information
	2.7 If N/PN/NI to 2.6: Was there potential for a substantial impact (on the result) of the failure to analyse participants in the group to which they were randomized ?	NI	Unable to find information.
	**Risk of bias judgement**	**High**	
Bias due to	3.1a Were data for this outcome available for all clusters that recruited participants?	PY	Table 3 shows that all individuals who included during the study period were included in the analysis. Whether or not data were available from all participnats is not clear.
	3.1b Were data for this outcome available for all, or nearly all, participants within clusters?	PY	

**missing outcome data**	3.2 If N/PN/NI to 3.1a or 3.1b: Is there evidence that the result was not biased by missing data?	NA	
	3.3 If N/PN to 3.2 Could missingness in the outcome depend on its true value?	NA	
	3.4 If Y/PY/NI to 3.3: Is it likely that missingness in the outcome depended on its true value?	NA	
	**Risk of bias judgement**	**Low**	
**Bias in measurement of the outcome**	4.1 Was the method of measuring the outcome inappropriate?	PN	"Information on falls was collected by the researchers from incident reports filed in patients' medical records,from notes in medical records themselves, and by asking a senior nurse each day about any falls on the ward in the past 24 h" (cited in journal article).
	4.2 Could measurement or ascertainment of the outcome have differed between intervention groups?	PN	Even though patients reporting falls, nursing staff recording falls, and researchers collecting data on falls from incident reports were aware of the assigned intervention, fall is an objective outcome.
	4.3a If N/PN/NI to 4.1 and 4.2: Were outcome assessors aware that a trial was taking place?	Y	
	4.3b If Y/PY/NI to 4.3a: Were outcome assessors aware of the intervention received by study participants?	Y	Participants in intervention wards were aware of their assigned intervention but patients in control wards were unaware that they were in a trial.
	4.4 If Y/PY/NI to 4.3b: Could assessment of the outcome have been influenced by knowledge of intervention received?	PN	
	4.5 If Y/PY/NI to 4.4: Is it likely that assessment of the outcome was influenced by knowledge of intervention received?	NA	
	**Risk of bias judgement**	**Low**	
**Bias in selection of the reported result**	5.1 Were the data that produced this result analysed in accordance with a pre-specified analysis plan that was finalized before unblinded outcome data were available for analysis?	NI	"The researchers had no direct contact with patients but were not able to be kept blind to whether patients were in intervention or control wards." (journal article)
	5.2 ... multiple eligible outcome measurements (e.g. scales, definitions, time points) within the outcome domain?	NI	
	5.3 ... multiple eligible analyses of the data?	NI	
	**Risk of bias judgement**	**Some concerns**	
**Overall bias**	**Risk of bias judgement**	**High**	

**Unique ID**	Barker(2016)_A	**Study ID**	8	**Assessor**	RS
**Ref or Label**		**Aim**	assignment to intervention (the 'intention-to-treat' effect)		
**Experimental**	The nurse-led 6-PACK program for reducing falls and fall related injuries in acute hospitals	**Comparator**	Usual care	**Source**	Journal article(s); Non-commercial trial registry record (e.g. ClinicalTrials.gov record)
**Outcome**	Rate of use of all 6-PACK programme components (fall risk tool and six interventions) per 1000 occupied bed days in intervention group compared with control group at 8 months follow-up	**Results**	Rate Ratio 3.05 (95% CI 2.14, 4.34)	**Weight**	1

Domain	Signalling question	Response	Comments
**Bias arising from the randomization process**	1a.1 Was the allocation sequence random?	Y	"We used the RALLOC command in Stata to develop the randomisation schedule, using a random sequence in blocks of two generated by the study statistician. Concealment of allocation was ensured, as the schedule was accessible only by the study statistician, who was not involved in ward recruitment or data collection."
	1a.2 Was the allocation sequence concealed until clusters were enrolled and assigned to interventions?	Y	
	1a.3 Did baseline differences between intervention groups suggest a problem with the randomization process?	N	"Characteristics of admitted patients and length of stay were similar for intervention and control groups and across baseline and randomised controlled trial periods."
	**Risk of bias judgement**	**Low**	
	1b.1 Were all the individual participants identified and recruited (if appropriate) before randomization of clusters?	**N**	Participants were admitted to the participating hospital wards after randomisation. "This cluster randomised controlled trial recruited 24 acute wards from six Australian hospitals. Wards were eligible to participate if they were nominated by participating hospitals as being wards where falls commonly occurred, had an average length of stay of patients of less than 10 days"

**Bias arising from the timing of identification or recruitment of participants**	1b.2 If N/PN/NI to 1b.1: Is it likely that selection of individual participants was affected by knowledge of the intervention assigned to the cluster?	**Unable to find information about whether the person(s) responsible for admitting patients to different wards were aware of the interventions assigned to the wards.**	
	1b.3 Were there baseline imbalances that suggest differential identification or recruitment of individual participants between intervention groups?	**N**	"Characteristics of admitted patients and length of stay were similar for intervention and control groups and across baseline and randomised controlled trial periods"
	**Risk of bias judgement**	**Some concerns**	Some concerns
**Bias due to deviations from intended interventions**	2.1a Were participants aware that they were in a trial?	Y	
	2.1b If Y/PY/NI to 2.1a: Were participants aware of their assigned intervention during the trial?	PY	As the intervention was nurse led, blinding of ward nurses and patients was not possible. Blinding of the assessors collecting the fall and falls prevention practice data was also not possible.
	2.2 Were carers and people delivering the interventions aware of participants' assigned intervention during the trial?	Y	
	2.3 If Y/PY/NI to 2.1b or 2.2: Were there deviations from the intended intervention that arose because of the trial context?	PN	"We investigated potential contamination by examining changes in the use of 6-PACK programme components from the baseline to the randomised controlled trial period in the control wards."<br><br>The authors state that "no major protocol deviations or unexpected adverse events occured during the study period" and "no change [in the use of 6-PACK programme components] was observed on the control wards, suggesting that contamination was unlikely".
	2.4 If Y/PY to 2.3: Were these deviations likely to have affected the outcome?	NA	
	2.5 If Y/PY/NI to 2.4: Were these deviations from intended intervention balanced between groups?	NA	
	2.6 Was an appropriate analysis used to estimate the effect of assignment to intervention?	PY	It it stated that "all analyses used intention to treat principles". Table 2 in the journal article showed that the number of participants analysed in the intervention and control group were the same as the number of participants randomised.
	2.7 If N/PN/NI to 2.6: Was there potential for a substantial impact (on the result) of the failure to analyse participants in the group to which they were randomized ?	NA	
	**Risk of bias judgement**	**Low**	
**Bias due to missing outcome data**	3.1a Were data for this outcome available for all clusters that recruited participants?	Y	"Eighteen of the 24 wards had a trial period of 12 months (fg 1). Three ward pairs had an 11 month trial period owing to ward closures".
	3.1b Were data for this outcome available for all, or nearly all, participants within clusters?	PY	The three ward pairs that han an 11 month trial period still had data available for nearly all participants admitted during the 12-month period.
	3.2 If N/PN/NI to 3.1a or 3.1b: Is there evidence that the result was not biased by missing data?	NA	
	3.3 If N/PN to 3.2 Could missingness in the outcome depend on its true value?	NA	
	3.4 If Y/PY/NI to 3.3: Is it likely that missingness in the outcome depended on its true value?	NA	
	**Risk of bias judgement**	**Low**	
**Bias in measurement of the outcome**	4.1 Was the method of measuring the outcome inappropriate?	PN	"Process data on completion of the risk tool and use of the 6-PACK programme components were recorded by daily medical record audit and structured bedside observation by trained assessors using a standardised tool during the baseline and study period."
	4.2 Could measurement or ascertainment of the outcome have differed between intervention groups?	NI	"Blinding of the assessors collecting the fall and falls prevention practice data was also not possible. Assessors blinded to group allocation did the secondary coding of characteristics of falls and injuries, and the primary assessor completed the coding."
	4.3a If N/PN/NI to 4.1 and 4.2: Were outcome assessors aware that a trial was taking place?	Y	
	4.3b If Y/PY/NI to 4.3a: Were outcome assessors aware of the intervention received by study participants?	Y	
	4.4 If Y/PY/NI to 4.3b: Could assessment of the outcome have been influenced by knowledge of intervention received?	NI	Based on the information available in the

	4.5 If Y/PY/NI to 4.4: Is it likely that assessment of the outcome was influenced by knowledge of intervention received?	PN	Based on the information available in the journal article and the trial registry record it is difficult to judge whether assessment of the outcome was influenced by knowledge of the intervention received. The outcome assessors were aware of the intervention received, and so could have been influenced when recording information from daily medical record audits and structured bedside observations. However, it seems that the process of collecting data on the use of 6-PACK programme components was fairly well standardised.
	**Risk of bias judgement**	**Some concerns**	
**Bias in selection of the reported result**	5.1 Were the data that produced this result analysed in accordance with a pre-specified analysis plan that was finalized before unblinded outcome data were available for analysis?	Y	Analysis intentions were available in ANZCTR 11.01.2012, and it was stated that one of the outcomes will be rate of falls per 1000 occupied bed days at 12 months follow-up, and that the analysis will be conducted according to intention-to-treat principles. Unblinded outcome data were available for analysis March 2013 at the earliest, because that is when the follow-up period stopped.
	5.2 ... multiple eligible outcome measurements (e.g. scales, definitions, time points) within the outcome domain?	PN	Based on the method of data collection I assume that only one eligible outcome measurement was available for assessment.
	5.3 ... multiple eligible analyses of the data?	N	It is clearly stated that all analyses will be conducted according to ITT principles, and results are presented for unadjusted analyses.
	**Risk of bias judgement**		
**Overall bias**	**Risk of bias judgement**	**Some concerns**	An overall RoB judgement of some concerns was given because the reason for judging the two domains at some concerns was lack of information and not the presence of evidence of bias.

**Unique ID**	Barker(2016)_B	**Study ID**	8	**Assessor**	RS
**Ref or Label**		**Aim**	assignment to intervention (the 'intention-to-treat' effect)		
**Experimental**	The nurse-led 6-PACK program for reducing falls and fall related injuries in acute hospitals	**Comparator**	Usual care	**Source**	Journal article(s); Non-commercial trial registry record (e.g. ClinicalTrials.gov record)
**Outcome**	Rate of falls per 1000 occupied bed days during 12 months follow-up in intervention group compared with control group	**Results**	Rate Ratio 1.04 (95% CI 0.78, 1.37)	**Weight**	

Domain	Signalling question	Response	Comments
**Bias arising from the randomization process**	1a.1 Was the allocation sequence random?	Y	"We used the RALLOC command in Stata to develop the randomisation schedule, using a random sequence in blocks of two generated by the study statistician. Concealment of allocation was ensured, as the schedule was accessible only by the study statistician, who was not involved in ward recruitment or data collection."
	1a.2 Was the allocation sequence concealed until clusters were enrolled and assigned to interventions?	Y	
	1a.3 Did baseline differences between intervention groups suggest a problem with the randomization process?	N	"Characteristics of admitted patients and length of stay were similar for intervention and control groups and across baseline and randomised controlled trial periods."
	**Risk of bias judgement**	**Low**	
**Bias arising from the timing of identification or recruitment of participants**	1b.1 Were all the individual participants identified and recruited (if appropriate) before randomization of clusters?	**N**	Participants were admitted to the participating hospital wards after randomisation. "This cluster randomised controlled trial recruited 24 acute wards from six Australian hospitals. Wards were eligible to participate if they were nominated by participating hospitals as being wards where falls commonly occurred, had an average length of stay of patients of less than 10 days"
	1b.2 If N/PN/NI to 1b.1: Is it likely that selection of individual participants was affected by knowledge of the intervention assigned to the cluster?	**Unable to find information about whether the person(s) responsible for admitting patients to different wards were aware of the interventions assigned to the wards.**	
	1b.3 Were there baseline imbalances that suggest differential identification or recruitment of individual participants between intervention groups?	**N**	"Characteristics of admitted patients and length of stay were similar for intervention and control groups and across baseline and randomised controlled trial periods"

	**Risk of bias judgement**	**Some concerns**	Some concerns
**Bias due to deviations from intended interventions**	2.1a Were participants aware that they were in a trial?	Y	
	2.1b If Y/PY/NI to 2.1a: Were participants aware of their assigned intervention during the trial?	PY	As the intervention was nurse led, blinding of ward nurses and patients was not possible. Blinding of the assessors collecting the fall and falls prevention practice data was also not possible.
	2.2 Were carers and people delivering the interventions aware of participants' assigned intervention during the trial?	Y	
	2.3 If Y/PY/NI to 2.1b or 2.2: Were there deviations from the intended intervention that arose because of the trial context?	PN	"We investigated potential contamination by examining changes in the use of 6-PACK programme components from the baseline to the randomised controlled trial period in the control wards."<br><br>The authors state that "no major protocol deviations or unexpected adverse events occured during the study period" and "no change [in the use of 6-PACK programme components] was observed on the control wards, suggesting that contamination was unlikely".
	2.4 If Y/PY to 2.3: Were these deviations likely to have affected the outcome?	NA	
	2.5 If Y/PY/NI to 2.4: Were these deviations from intended intervention balanced between groups?	NA	
	2.6 Was an appropriate analysis used to estimate the effect of assignment to intervention?	PY	It it stated that "all analyses used intention to treat principles". Table 2 in the journal article showed that the number of participants analysed in the intervention and control group were the same as the number of participants randomised.
	2.7 If N/PN/NI to 2.6: Was there potential for a substantial impact (on the result) of the failure to analyse participants in the group to which they were randomized ?	NA	
	**Risk of bias judgement**	**Low**	
**Bias due to missing outcome data**	3.1a Were data for this outcome available for all clusters that recruited participants?	Y	"Eighteen of the 24 wards had a trial period of 12 months (fg 1). Three ward pairs had an 11 month trial period owing to ward closures".
	3.1b Were data for this outcome available for all, or nearly all, participants within clusters?	PY	The three ward pairs that han an 11 month trial period still had data available for nearly all participants admitted during the 12-month period.
	3.2 If N/PN/NI to 3.1a or 3.1b: Is there evidence that the result was not biased by missing data?	NA	
	3.3 If N/PN to 3.2 Could missingness in the outcome depend on its true value?	NA	
	3.4 If Y/PY/NI to 3.3: Is it likely that missingness in the outcome depended on its true value?	NA	
	**Risk of bias judgement**	**Low**	
**Bias in measurement of the outcome**	4.1 Was the method of measuring the outcome inappropriate?	PN	"Falls data were prospectively collected via daily audit of patients' medical recordsand verbal reports from the ward nurse unit manager about falls known to have occurred within the previous 24 hours, as well as monthly audit of hospital incident reporting and administrative databases. We reviewed radiological investigation reports to verify fractures. A second independent assessor reviewed and re-coded all recorded falls (ward, injuries sustained, fall location, time, and activity), and disagreements were resolved by a third."
	4.2 Could measurement or ascertainment of the outcome have differed between intervention groups?	PN	"Blinding of the assessors collecting the fall and falls prevention practice data was also not possible. Assessors blinded to group allocation did the secondary coding of characteristics of falls and injuries, and the primary<br>assessor completed the coding."
	4.3a If N/PN/NI to 4.1 and 4.2: Were outcome assessors aware that a trial was taking place?	Y	
	4.3b If Y/PY/NI to 4.3a: Were outcome assessors aware of the intervention received by study participants?	Y	
	4.4 If Y/PY/NI to 4.3b: Could assessment of the outcome have been influenced by knowledge of intervention received?	PN	The outcome of falls is reasonably objective. As quoted above, assessors blinded to group allocation did the secondary coding of falls, and the primary assessor completed the coding, so it is unlikely that assessment of the outcome was influence by knowledge of the intervention received.
	4.5 If Y/PY/NI to 4.4: Is it likely that assessment of the outcome was influenced by knowledge of intervention received?	NA	
	**Risk of bias judgement**	**Low**	
**Bias in selection of the reported**	5.1 Were the data that produced this result analysed in accordance with a pre-specified analysis plan that was finalized before unblinded outcome data were available for analysis?	Y	Analysis intentions were available in ANZCTR 11.01.2012, and it was stated that one of the outcomes will be rate of falls per 1000 occupied bed days at 12 months follow-up, and that the analysis will be conducted according to intention-to-treat principles. Unblinded outcome data were available for analysis March 2013 at the earliest, because that is when the follow-up period stopped.

of the reported result	5.2 ... multiple eligible outcome measurements (e.g. scales, definitions, time points) within the outcome domain?	PN	Based on the method of data collection I assume that only one eligible outcome measurement was available for assessment.
	5.3 ... multiple eligible analyses of the data?	N	It is clearly stated that all analyses will be conducted according to ITT principles, and results are presented for unadjusted analyses.
	**Risk of bias judgement**		
**Overall bias**	**Risk of bias judgement**	**Some concerns**	

**Unique ID**	Bhasin(2020)_A	**Study ID**	2974	**Assessor**	RS
**Ref or Label**		**Aim**	assignment to intervention (the 'intention-to-treat' effect)		
**Experimental**	Multifactorial intervention that included risk assessment and individualized plans	**Comparator**	Enhanced usual care	**Source**	Journal article(s); Trial protocol; Statistical analysis plan (SAP); Non-commercial trial registry record (e.g. ClinicalTrials.gov record)
**Outcome**	Rate of times to first adjudicated serious fall injury per 100 person-years during 44 months follow-up in intervention group compared with control group	**Results**	Hazard Ratio 0.92 (95% CI 0.80, 1.06)	**Weight**	

Domain	Signalling question	Response	Comments
**Bias arising from the randomization process**	1a.1 Was the allocation sequence random?	Y	"Practices underwent cluster randomization to the intervention (intervention group) or to an enhanced usual care approach (control group) with the use of covariate-constrained randomization, with stratification according to health care system and balancing covariates (i.e., the size of the practice, the location of the practice [urban vs. rural], and the race and ethnic group of the majority of persons in the practice [nonwhite vs. white, and Hispanic vs. non-Hispanic])" (cited in journal article).<br><br>"The central recruitment staff will be kept blinded to randomization status of the practices and will be rigorously trained to reduce potential bias" (cited in trial protocol).<br><br>"Only the trial biostatisticians participated in the generation of the randomization. No one else from the trial was involved in the process. To<br>minimize the risk of selection bias, practice site names were masked during the process. The practice site randomization assignments to treatment groups were released to the clinical sites only after careful vetting of the entire randomization process by the trial biostatisticians" (cited in trial protocol).
	1a.2 Was the allocation sequence concealed until clusters were enrolled and assigned to interventions?	Y	
	1a.3 Did baseline differences between intervention groups suggest a problem with the randomization process?	N	"The practices assigned to the<br>two groups were similar with respect to the size of the practice, the number of practices that were in urban locations as compared with rural locations, the number of practices in which a majority of the participants was white as compared with nonwhite, and the practice-level baseline characteristics of the participants (Table S1)" (cited in journal article).
	**Risk of bias judgement**	**Low**	
**Bias arising from the timing of identification or recruitment of participants**	1b.1 Were all the individual participants identified and recruited (if appropriate) before randomization of clusters?	**N**	"Because randomization of practices will occur prior to participant enrollment, it will be important to minimize any potential bias due to lack of allocation concealment" (cited in trial protocol).
	1b.2 If N/PN/NI to 1b.1: Is it likely that selection of individual participants was affected by knowledge of the intervention assigned to the cluster?	**"The central recruitment staff will be kept blinded to randomization status of the practices and will be rigorously trained to reduce potential bias" (cited in trial protocol). It is unlikely that selection of individual participants was affected by knowledge of the intervention assigned to the cluster.**	

	1b.3 Were there baseline imbalances that suggest differential identification or recruitment of individual participants between intervention groups?	**N**	"The practices assigned to the two groups were similar with respect to the size of the practice, the number of practices that were in urban locations as compared with rural locations, the number of practices in which a majority of the participants was white as compared with nonwhite, and the practice-level baseline characteristics of the participants (Table S1)" (cited in journal article).
	**Risk of bias judgement**	**Low**	Low
**Bias due to deviations from intended interventions**	2.1a Were participants aware that they were in a trial?	NI	Unable to find information about this.
	2.1b If Y/PY/NI to 2.1a: Were participants aware of their assigned intervention during the trial?	NI	Unable to find information about this.
	2.2 Were carers and people delivering the interventions aware of participants' assigned intervention during the trial?	Y	
	2.3 If Y/PY/NI to 2.1b or 2.2: Were there deviations from the intended intervention that arose because of the trial context?	PN	The unit of randomisation was the primary care practice, and as such it is unlikely that there were deviations from the intended intervention that arose because of the trial context. Figure S2 shows that 2404 of the 2802 patients allocated to experimental intervention received the intervention, and that all 2649 patients allocated to control intervention received the "stay independent" pamphlet.
	2.4 If Y/PY to 2.3: Were these deviations likely to have affected the outcome?	NA	
	2.5 If Y/PY/NI to 2.4: Were these deviations from intended intervention balanced between groups?	NA	
	2.6 Was an appropriate analysis used to estimate the effect of assignment to intervention?	PY	"The analysis of the primary and secondary outcomes will be according to the principle of intent-to-treat (i.e., practices/participants will be analyzed according to their original treatment assignment regardless of adherence to protocol)" (cited in trial protocol).
	2.7 If N/PN/NI to 2.6: Was there potential for a substantial impact (on the result) of the failure to analyse participants in the group to which they were randomized ?	NA	
	**Risk of bias judgement**	**Low**	
**Bias due to missing outcome data**	3.1a Were data for this outcome available for all clusters that recruited participants?	Y	Figure S1 shows that all primary care practices completed the follow-up and were included in the statistical analysis.
	3.1b Were data for this outcome available for all, or nearly all, participants within clusters?	Y	Follow-up status was available for all participants (Figure S2).<br><br>Outcome data were available for 86.5% and 88.5% of the total person-years of follow-up in the experimental intervention and control intervention, respectively.
	3.2 If N/PN/NI to 3.1a or 3.1b: Is there evidence that the result was not biased by missing data?	NA	
	3.3 If N/PN to 3.2 Could missingness in the outcome depend on its true value?	NA	
	3.4 If Y/PY/NI to 3.3: Is it likely that missingness in the outcome depended on its true value?	NA	
	**Risk of bias judgement**	**Low**	
**Bias in measurement of the outcome**	4.1 Was the method of measuring the outcome inappropriate?	N	"Data on fall injuries were collected every 4 months by means of telephone interviews, which were conducted by personnel who were unaware of the treatment assignments. During these interviews, participants were also asked about hospital admissions, emergency department visits, and other health care utilization. To facilitate participants' recall, the participants were provided with a monthly calendar in which to record their falls and injuries" (cited in journal article).
	4.2 Could measurement or ascertainment of the outcome have differed between intervention groups?	PN	The personnel who collected the outcome data were unaware of the treatment assignments.
	4.3a If N/PN/NI to 4.1 and 4.2: Were outcome assessors aware that a trial was taking place?	PY	""Data on fall injuries were collected every 4 months by means of telephone interviews, which were conducted by personnel who were unaware of the treatment assignments" (cited in journal article).
	4.3b If Y/PY/NI to 4.3a: Were outcome assessors aware of the intervention received by study participants?	N	See above
	4.4 If Y/PY/NI to 4.3b: Could assessment of the outcome have been influenced by knowledge of intervention received?	NA	
	4.5 If Y/PY/NI to 4.4: Is it likely that assessment of the outcome was influenced by knowledge of intervention received?	NA	
	**Risk of bias judgement**	**Low**	
	5.1 Were the data that produced this result analysed in accordance with a pre-specified analysis plan that was finalized before unblinded outcome data were available for analysis?	Y	"Revisions to the SAP were done before unblinding of the data" (cited in trial protocol".

**Bias in selection of the reported result**	5.2 ... multiple eligible outcome measurements (e.g. scales, definitions, time points) within the outcome domain?	PN	The outcome as specified in the first version of the SAP (April 2015) seems to be in concordance with the outcome as specified in the journal article. On the first of June 2018 the primary outcome was modified to reflect the recommendations of the Ad Hoc Expert Panel established by the NIA and NIA's concurrence with this recommendation. Also, the result is based on an analysis conducted in accordance with ITT principles, as specified in the SAP.
	5.3 ... multiple eligible analyses of the data?	PN	
	**Risk of bias judgement**		
**Overall bias**	**Risk of bias judgement**	**Low**	

**Unique ID**	Blalock(2020)_A	**Study ID**	1801	**Assessor**	RS
**Ref or Label**		**Aim**	assignment to intervention (the 'intention-to-treat' effect)		
**Experimental**	STEADI-Rx	**Comparator**	No-intervention comparison group	**Source**	Journal article(s)
**Outcome**	Change in use of fall risk-increasing drugs (Drug Burden Index score) from 12-month pre-intervention period to 12-month post-intervention period (among participants who screened positive for fall risk) in intervention group compared with control group	**Results**	ean difference – 0.12 (SE 0.02) in intervention group compared with – 0.08 (SE – 0.01) in control group (p = 0.05)	**Weight**	1

**Domain**	**Signalling question**	**Response**	**Comments**
**Bias arising from the randomization process**	1a.1 Was the allocation sequence random?	NI	Unable to find a statement on how the allocation sequence was generated other than statements such as "... we report finding from a randomized control trial ...", " ... 34 were randomized to the no-treatment control and 31 to the intervention group".<br><br>Unable to find information on whether the allocation sequence was concealed until clusters were enrolled and assigned to interventoins.
	1a.2 Was the allocation sequence concealed until clusters were enrolled and assigned to interventions?	NI	
	1a.3 Did baseline differences between intervention groups suggest a problem with the randomization process?	PN	See Figure 1 in journal article.<br>"Among participants with continuous Medicare Part D/NC Medicaid coverage (n = 3212), mean DBI scores were similar between the intervention and control groups during the preintervention period".
	**Risk of bias judgement**	**Some concerns**	
**Bias arising from the timing of identification or recruitment of participants**	1b.1 Were all the individual participants identified and recruited (if appropriate) before randomization of clusters?	**N**	It is likely that not all individual participants were identified and recruited prior to randomisation of clusters, as some individual participants may have started using a pharmacy after randomisation of pharmacies to experimental intervention or control intervention groups. For instance, some individuals may have started visiting a certain pharmacy after recently having moved to the area.
	1b.2 If N/PN/NI to 1b.1: Is it likely that selection of individual participants was affected by knowledge of the intervention assigned to the cluster?	**It is considered very unlikely that selection of individual participants was affected by knowledge of the intervention assigned to the cluster, as individuals' decision on which pharmacy to visit was most likely not affected by knowledge of an ongoing study.**	
	1b.3 Were there baseline imbalances that suggest differential identification or recruitment of individual participants between intervention groups?	**PN**	See Figure 1 in journal article.
	**Risk of bias judgement**	**Low**	Low
	2.1a Were participants aware that they were in a trial?	PY	
	2.1b If Y/PY/NI to 2.1a: Were participants aware of their assigned intervention during the trial?	PY	It is possible that screening procedures

Bias due to deviations from intended interventions	2.2 Were carers and people delivering the interventions aware of participants' assigned intervention during the trial?	Y	increased the awareness of the role medications play in fall risk among participnats who screened positive but who did not receive a medication review, "leading them to speak with their healthcare provider about reducing the use of unneded medications or discontinuing these medications on their own" (cited in journal article).<br><br>Pharmacists delivering the intervention were aware of participants' assigned intervention during the trial as they directly interacted with the participant during the fall risk assessment and medication review.
	2.3 If Y/PY/NI to 2.1b or 2.2: Were there deviations from the intended intervention that arose because of the trial context?	NI	"Other factors that could have contributed to our null findings include contamination between the intervention and control group caused by providers who had patients in both groups and contemporaneous public health initiatives such as efforts to address the opioid crisis" (cited in journal article).<br><br>If intervention participants who screened positive for fall risk but who did not receive a medication review contacted their healthcare provider, who subsequently altered medications, this is considered a deviation from intervention that is part of usual practice, which does not cause bias. However, intervention contamination is caused by the trial context and may (if contamination took place) have caused bias in the estimated effect of intervention.
	2.4 If Y/PY to 2.3: Were these deviations likely to have affected the outcome?	NA	
	2.5 If Y/PY/NI to 2.4: Were these deviations from intended intervention balanced between groups?	NA	
	2.6 Was an appropriate analysis used to estimate the effect of assignment to intervention?	NI	
	2.7 If N/PN/NI to 2.6: Was there potential for a substantial impact (on the result) of the failure to analyse participants in the group to which they were randomized ?	PN	If there was contamination between intervention and control group, these deviations from intended interventions would probably not have a substantial impact on the estimated effects of interventions.
	**Risk of bias judgement**	**Some concerns**	
Bias due to missing outcome data	3.1a Were data for this outcome available for all clusters that recruited participants?	PY	"In the full sample, 10 participants had missing data for sex" (cited in journal article).
	3.1b Were data for this outcome available for all, or nearly all, participants within clusters?	PY	
	3.2 If N/PN/NI to 3.1a or 3.1b: Is there evidence that the result was not biased by missing data?	NA	
	3.3 If N/PN to 3.2 Could missingness in the outcome depend on its true value?	NA	
	3.4 If Y/PY/NI to 3.3: Is it likely that missingness in the outcome depended on its true value?	NA	
	**Risk of bias judgement**	**Low**	
Bias in measurement of the outcome	4.1 Was the method of measuring the outcome inappropriate?	PN	"We assessed use of medications associated with an increased risk of falling using the Drug Burden Index (DBI). In past research, the DBI was shown to predict falls and other functional outcomes among older adults. In this study, we used information from Medicare Part D/NC Medicaid claims records (i.e., medication name, strength, dosage form, date dispensed, quantity dispensed, days' supply) to calculate a DBI score for each patient for each month during the 12-month preintervention and the 12-month postintervention period" (cited in journal article). DBI scores for each drug were summed across all of the medications the patient was taking with sedative or anticholinergic properties to yield a single patient-level score. Table 1 lists medications included in the DBI.
	4.2 Could measurement or ascertainment of the outcome have differed between intervention groups?	PN	Mean Drug Burden Index score was based on information from Medicare Part D/NC Medicaid claims records. This is an observer-reported outcome not involving judgement, and ascertainment of the outcome was unlikely to differ between intervention groups.
	4.3a If N/PN/NI to 4.1 and 4.2: Were outcome assessors aware that a trial was taking place?	PY	Unable to find information about whether outcome assessors were aware that a trial was taking place or of the intervention received by study participants, which leads me to believe that outcome assessors were aware.
	4.3b If Y/PY/NI to 4.3a: Were outcome assessors aware of the intervention received by study participants?	PY	
	4.4 If Y/PY/NI to 4.3b: Could assessment of the outcome have been influenced by knowledge of intervention received?	PN	Mean Drug Burden Index score was based [illegible]

	4.5 If Y/PY/NI to 4.4: Is it likely that assessment of the outcome was influenced by knowledge of intervention received?	NA	on information from Medicare Part D/NC Medicaid claims records, which is an observer-reported outcome not involving judgement.
	**Risk of bias judgement**	**Low**	
**Bias in selection of the reported result**	5.1 Were the data that produced this result analysed in accordance with a pre-specified analysis plan that was finalized before unblinded outcome data were available for analysis?	NI	Unable to find information about this.
	5.2 ... multiple eligible outcome measurements (e.g. scales, definitions, time points) within the outcome domain?	PN	Based on the journal article alone, this result seems to correspond with the plan as stated in the methods section and is assumed to not have been selected from multiple eligible outcome measuresments or analyses of the data.
	5.3 ... multiple eligible analyses of the data?	PN	
	**Risk of bias judgement**		
**Overall bias**	**Risk of bias judgement**	**Some concerns**	I have chosen to give this overall result 'some concerns' for risk of bias despite there being some concerns for risk of bias from three individual domains. This is because my overall impression of this does not justify a high risk of bias, but rather some concerns for risk of bias.

**Unique ID**	Blalock(2020)_B	**Study ID**	1801	**Assessor**	RS
**Ref or Label**		**Aim**	assignment to intervention (the 'intention-to-treat' effect)		
**Experimental**	STEADI-Rx	**Comparator**	No-intervention comparison group	**Source**	Journal article(s)
**Outcome**	Risk of falling during 12-month post-intervention period, controlled for risk of falling during 12-month pre-intervention period, in intervention group compared with control group	**Results**	Odds Ratio 1.09 (95% CI 0.81, 1.47; p = 0.58)	**Weight**	1

Domain	Signalling question	Response	Comments
**Bias arising from the randomization process**	1a.1 Was the allocation sequence random?	NI	Unable to find a statement on how the allocation sequence was generated other than statements such as "... we report finding from a randomized control trial ...", " ... 34 were randomized to the no-treatment control and 31 to the intervention group".<br><br>Unable to find information on whether the allocation sequence was concealed until clusters were enrolled and assigned to interventoins.
	1a.2 Was the allocation sequence concealed until clusters were enrolled and assigned to interventions?	NI	
	1a.3 Did baseline differences between intervention groups suggest a problem with the randomization process?	PN	See Figure 1 in journal article.
	**Risk of bias judgement**	**Some concerns**	
**Bias arising from the timing of identification or recruitment of participants**	1b.1 Were all the individual participants identified and recruited (if appropriate) before randomization of clusters?	**N**	It is likely that not all individual participants were identified and recruited prior to randomisation of clusters, as some individual participants may have started using a pharmacy after randomisation of pharmacies to experimental intervention or control intervention groups. For instance, some individuals may have started visiting a certain pharmacy after recently having moved to the area.
	1b.2 If N/PN/NI to 1b.1: Is it likely that selection of individual participants was affected by knowledge of the intervention assigned to the cluster?	**It is considered very unlikely that selection of individual participants was affected by knowledge of the intervention assigned to the cluster, as individuals' decision on which pharmacy to visit was most likely not affected by knowledge of an ongoing study.**	
	1b.3 Were there baseline imbalances that suggest differential identification or recruitment of individual participants between intervention groups?	**PN**	See Figure 1 in journal article.
	**Risk of bias judgement**	**Low**	Low
	2.1a Were participants aware that they were in a trial?	PY	

**Bias due to deviations from intended interventions**	2.1b If Y/PY/NI to 2.1a: Were participants aware of their assigned intervention during the trial?	PY	It is possible that screening procedures increased the awareness of the role medications play in fall risk among participnats who screened positive but who did not receive a medication review, "leading them to speak with their healthcare provider about reducing the use of unneded medications or discontinuing these medications on their own" (cited in journal article).<br><br>Pharmacists delivering the intervention were aware of participants' assigned intervention during the trial as they directly interacted with the participant during the fall risk assessment and medication review.
	2.2 Were carers and people delivering the interventions aware of participants' assigned intervention during the trial?	Y	
	2.3 If Y/PY/NI to 2.1b or 2.2: Were there deviations from the intended intervention that arose because of the trial context?	NI	"Other factors that could have contributed to our null findings include contamination between the intervention and control group caused by providers who had patients in both groups and contemporaneous public health initiatives such as efforts to address the opioid crisis" (cited in journal article).<br><br>If intervention participants who screened positive for fall risk but who did not receive a medication review contacted their healthcare provider, who subsequently altered medications, this is considered a deviation from intervention that is part of usual practice, which does not cause bias. However, intervention contamination is caused by the trial context and may (if contamination took place) have caused bias in the estimated effect of intervention.
	2.4 If Y/PY to 2.3: Were these deviations likely to have affected the outcome?	NA	
	2.5 If Y/PY/NI to 2.4: Were these deviations from intended intervention balanced between groups?	NA	
	2.6 Was an appropriate analysis used to estimate the effect of assignment to intervention?	NI	
	2.7 If N/PN/NI to 2.6: Was there potential for a substantial impact (on the result) of the failure to analyse participants in the group to which they were randomized ?	PN	If there was contamination between intervention and control group, these deviations from intended interventions would probably not have a substantial impact on the estimated effects of interventions.
	**Risk of bias judgement**	**Some concerns**	
**Bias due to missing outcome data**	3.1a Were data for this outcome available for all clusters that recruited participants?	PY	"In the full sample, 10 participants had missing data for sex" (cited in journal article).
	3.1b Were data for this outcome available for all, or nearly all, participants within clusters?	PY	
	3.2 If N/PN/NI to 3.1a or 3.1b: Is there evidence that the result was not biased by missing data?	NA	
	3.3 If N/PN to 3.2 Could missingness in the outcome depend on its true value?	NA	
	3.4 If Y/PY/NI to 3.3: Is it likely that missingness in the outcome depended on its true value?	NA	
	**Risk of bias judgement**	**Low**	
**Bias in measurement of the outcome**	4.1 Was the method of measuring the outcome inappropriate?	PN	"Falls were assessed using claims records for emergency department visits and hospitalizations. For both the preintervention and postintervention period, participants were classified as having experienced a fall if they had any claims with an International Classification of Disease (ICD)-10 diagnostic code indicating (1) falls or repeated falls (W00-W19 and R296, respectively) or (2) an injury likely to be fall related (ie, head injury, lower limb fracture and other injury, upper limb fracture and other injury, neck/trunk fracture and other injury, and upper limb contusion and open wound)" (cited in journal article).
	4.2 Could measurement or ascertainment of the outcome have differed between intervention groups?	PN	Falls assessed using claims records for emergency department visits and hospitalizations is an outcome that reflects decisions made by the intervention provider, and the recording of this decision does not involve any judgement.
	4.3a If N/PN/NI to 4.1 and 4.2: Were outcome assessors aware that a trial was taking place?	PY	Unable to find information about whether outcome assessors were aware that a trial was taking place or of the intervention received by study participants, which leads me to believe that outcome assessors were aware.
	4.3b If Y/PY/NI to 4.3a: Were outcome assessors aware of the intervention received by study participants?	PY	
	4.4 If Y/PY/NI to 4.3b: Could assessment of the outcome have been influenced by knowledge of intervention received?	PN	It is considered unlikely that assessment of [illegible]

	4.5 If Y/PY/NI to 4.4: Is it likely that assessment of the outcome was influenced by knowledge of intervention received?	NA	the outcome was influenced by knowledge of the intervention received because the outcome reflects decisions made by the intervention provider.
	**Risk of bias judgement**	**Low**	
**Bias in selection of the reported result**	5.1 Were the data that produced this result analysed in accordance with a pre-specified analysis plan that was finalized before unblinded outcome data were available for analysis?	NI	Unable to find information about this.
	5.2 ... multiple eligible outcome measurements (e.g. scales, definitions, time points) within the outcome domain?	PN	Based on the journal article alone, this result seems to correspond with the plan as stated in the methods section and is assumed to not have been selected from multiple eligible outcome measuresments or analyses of the data.
	5.3 ... multiple eligible analyses of the data?	PN	
	**Risk of bias judgement**		
**Overall bias**	**Risk of bias judgement**	**Some concerns**	I have chosen to give this overall result 'some concerns' for risk of bias despite there being some concerns for risk of bias from three individual domains. An overall RoB judgement of some concerns was given because the reason for judging the three domains at some concerns was lack of information and not the presence of evidence of bias.

**Unique ID**	Blum (2021)_A	**Study ID**	3894	**Assessor**	RS
**Ref or Label**		**Aim**	assignment to intervention (the 'intention-to-treat' effect)		
**Experimental**	Structured pharmacotherapy optimisation intervention supported by a software-based clinical decision support tool	**Comparator**	Usual care including unstructured medication review unsupported by STOPP/START criteria or the systematic tool to reduce inappropriate prescribing (STRIP) tool	**Source**	Journal article(s); Trial protocol
**Outcome**	Rate of falls per person-year during 12 months follow-up in intervention group compared with control group	**Results**	Hazard ratio 0.96 (95% CI 0.79, 1.15; p = 0.64)	**Weight**	

Domain	Signalling question	Response	Comments
**Bias arising from the randomization process**	1a.1 Was the allocation sequence random?	Y	"Physicians are allocated 1:1 to either the intervention arm or the control arm, using a probabilistic minimisation method implemented by a web-based clinical trial management system (WebSpirit hosted by the Clinical Trials Unit (CTU) Bern). Minimisation is done according to country in order to ensure a balanced distribution of hospitals. The minimisation algorithm is implemented using randomisation lists generated by an independent statistician in Stata (StataCorp., Stata Statistical Software Version 14). Only system administrators who are otherwise not involved in the conduct of the trial have access to the randomisation lists, to ensure concealment of allocation." (cited in Adams et al. (2019)
	1a.2 Was the allocation sequence concealed until clusters were enrolled and assigned to interventions?	Y	
	1a.3 Did baseline differences between intervention groups suggest a problem with the randomization process?	N	Table 1 in the journal article suggests that cluter size, specialty type, and interval between first and final patient recruitment were similar, as well as patient characteristics were similar between the groups.
	**Risk of bias judgement**	**Low**	
**Bias arising from the timing of identification or recruitment of participants**	1b.1 Were all the individual participants identified and recruited (if appropriate) before randomization of clusters?	**N**	Individual participants were (doctors) were sequentially enrolled over 21 months and allocated in a 1:1 ratio to the intervention or control arms.<br><br>Individual participants (hospitalised patients) were included if they were admitted between 1 Dec 2016 and 31 Oct 2018.
	1b.2 If N/PN/NI to 1b.1: Is it likely that selection of individual participants was affected by knowledge of the intervention assigned to the cluster?	**It is considered unlikely that those responsible for recruiting participants or those admitting patients were affected by knowledge of cluster allocation.**	
	1b.3 Were there baseline imbalances that suggest differential identification or recruitment of individual participants between intervention groups?	**N**	See 1a.3
	**Risk of bias judgement**	**Low**	Low

**Bias due to deviations from intended interventions**	2.1a Were participants aware that they were in a trial?	Y	"The intervention team consisted of a doctor and a pharmacist; neither was blinded to enable direct interactions with both the attending hospital doctors and the participants" (cited in journal article).
	2.1b If Y/PY/NI to 2.1a: Were participants aware of their assigned intervention during the trial?	N	As the intervention was nurse led, blinding of ward nurses and patients was not possible. Blinding of the assessors collecting the fall and falls prevention practice data was also not possible.
	2.2 Were carers and people delivering the interventions aware of participants' assigned intervention during the trial?	Y	
	2.3 If Y/PY/NI to 2.1b or 2.2: Were there deviations from the intended intervention that arose because of the trial context?	NI	"We investigated potential contamination by examining changes in the use of 6-PACK programme components from the baseline to the randomised controlled trial period in the control wards."<br><br>The authors state that "no major protocol deviations or unexpected adverse events occured during the study period" and "no change [in the use of 6-PACK programme components] was observed on the control wards, suggesting that contamination was unlikely".
	2.4 If Y/PY to 2.3: Were these deviations likely to have affected the outcome?	NA	
	2.5 If Y/PY/NI to 2.4: Were these deviations from intended intervention balanced between groups?	NA	
	2.6 Was an appropriate analysis used to estimate the effect of assignment to intervention?	PY	It it stated that "all analyses used intention to treat principles". Table 2 in the journal article showed that the number of participants analysed in the intervention and control group were the same as the number of participants randomised.
	2.7 If N/PN/NI to 2.6: Was there potential for a substantial impact (on the result) of the failure to analyse participants in the group to which they were randomized ?	NA	
	**Risk of bias judgement**	**Some concerns**	
**Bias due to missing outcome data**	3.1a Were data for this outcome available for all clusters that recruited participants?	Y	"The primary analysis was performed according to intention to treat, including all clusters and participants in the allocated groups" (cited in journal article).
	3.1b Were data for this outcome available for all, or nearly all, participants within clusters?	N	During follow-up, 10 (0.5%) participants were lost to follow-up, 118 (5.9%) withdrew from the trial, and 385 (19.2%) died (375 within 365 days)" (cited in journal article).<br><br>I think that there is a theoretical chance that the outcome of death could have led to a bias in the outcome of falls, and when 19.2% of patients died, data from these patients are considered missing.
	3.2 If N/PN/NI to 3.1a or 3.1b: Is there evidence that the result was not biased by missing data?	PY	Figure 1 in the journal article shows that there were small differences between the the experimental and comparator intervention groups in the proportion of missing data due to deaths, withdrawals and loss to follow-up.
	3.3 If N/PN to 3.2 Could missingness in the outcome depend on its true value?	NA	
	3.4 If Y/PY/NI to 3.3: Is it likely that missingness in the outcome depended on its true value?	NA	
	**Risk of bias judgement**	**Low**	
**Bias in measurement of the outcome**	4.1 Was the method of measuring the outcome inappropriate?	PY	No diary was used and the data on falls were based on participants' recall during the follow-up period.<br><br>"Blinded team members collected follow-up and outcome data through telephone interviews with the participants or their proxies at 2, 6, and 12 months post-randomisation" (cited in journal article).
	4.2 Could measurement or ascertainment of the outcome have differed between intervention groups?	PN	As this is a large-scale multicentre cluster-randomised trial conducted in hospitals in several European countries, it is considered unlikely that the method of ascertaintment of falls differed between intervention groups.
	4.3a If N/PN/NI to 4.1 and 4.2: Were outcome assessors aware that a trial was taking place?	NA	
	4.3b If Y/PY/NI to 4.3a: Were outcome assessors aware of the intervention received by study participants?	NA	
	4.4 If Y/PY/NI to 4.3b: Could assessment of the outcome have been influenced by knowledge of intervention received?	NA	
	4.5 If Y/PY/NI to 4.4: Is it likely that assessment of the outcome was influenced by knowledge of intervention received?	NA	
	**Risk of bias judgement**	**High**	

**Bias in selection of the reported result**	5.1 Were the data that produced this result analysed in accordance with a pre-specified analysis plan that was finalized before unblinded outcome data were available for analysis?	Y	All statistical analyses including a description of all relevant derivations of variables will be described in a statistical analysis plan before the end of recruitment and without inspection of the data" (cited in the trial protocol Adam et al. 2019).<br><br>"Binary outcomes will be analysed using a mixed-effects logistic regression model using a fixed effect for the intervention group and random effects for centre and treating physician" (cited in the Adam et al. 2019).
	5.2 ... multiple eligible outcome measurements (e.g. scales, definitions, time points) within the outcome domain?	N	The rate of falls during 12 months follow-up was reported as described in the trial protocol by Adam et al. 2019, including the time point.
	5.3 ... multiple eligible analyses of the data?	N	
	**Risk of bias judgement**		
**Overall bias**	**Risk of bias judgement**	**High**	

**Unique ID**	Blum (2021)_B	**Study ID**	3894	**Assessor**	RS
**Ref or Label**		**Aim**	assignment to intervention (the 'intention-to-treat' effect)		
**Experimental**	Structured pharmacotherapy optimisation intervention supported by a software-based clinical decision support tool	**Comparator**	Usual care including unstructured medication review unsupported by STOPP/START criteria or the systematic tool to reduce inappropriate prescribing (STRIP) tool	**Source**	Journal article(s); Trial protocol
**Outcome**	Presence of drug overuse (based on STOPP criteria) during two months follow-up in intervention group compared with control group	**Results**	Odds ratio 0.99 (95% CI 0.82, 1.20; p = 0.91)	**Weight**	

Domain	Signalling question	Response	Comments
**Bias arising from the randomization process**	1a.1 Was the allocation sequence random?	Y	"Physicians are allocated 1:1 to either the intervention arm or the control arm, using a probabilistic minimisation method implemented by a web-based clinical trial management system (WebSpirit hosted by the Clinical Trials Unit (CTU) Bern). Minimisation is done according to country in order to ensure a balanced distribution of hospitals. The minimisation algorithm is implemented using randomisation lists generated by an independent statistician in Stata (StataCorp., Stata Statistical Software Version 14). Only system administrators who are otherwise not involved in the conduct of the trial have access to the randomisation lists, to ensure concealment of allocation." (cited in Adams et al. (2019)
	1a.2 Was the allocation sequence concealed until clusters were enrolled and assigned to interventions?	Y	
	1a.3 Did baseline differences between intervention groups suggest a problem with the randomization process?	N	Table 1 in the journal article suggests that cluter size, specialty type, and interval between first and final patient recruitment were similar, as well as patient characteristics were similar between the groups.
	**Risk of bias judgement**	**Low**	
**Bias arising from the timing of identification or recruitment of participants**	1b.1 Were all the individual participants identified and recruited (if appropriate) before randomization of clusters?	**N**	Individual participants were (doctors) were sequentially enrolled over 21 months and allocated in a 1:1 ratio to the intervention or control arms.<br><br>Individual participants (hospitalised patients) were included if they were admitted between 1 Dec 2016 and 31 Oct 2018.
	1b.2 If N/PN/NI to 1b.1: Is it likely that selection of individual participants was affected by knowledge of the intervention assigned to the cluster?	**It is considered unlikely that those responsible for recruiting participants or those admitting patients were affected by knowledge of cluster allocation.**	
	1b.3 Were there baseline imbalances that suggest differential identification or recruitment of individual participants between intervention groups?	**N**	See 1a.3
	**Risk of bias judgement**	**Low**	Low

**Bias due to deviations from intended interventions**	2.1a Were participants aware that they were in a trial?	Y	"The intervention team consisted of a doctor and a pharmacist; neither was blinded to enable direct interactions with both the attending hospital doctors and the participants" (cited in journal article).
	2.1b If Y/PY/NI to 2.1a: Were participants aware of their assigned intervention during the trial?	N	As the intervention was nurse led, blinding of ward nurses and patients was not possible. Blinding of the assessors collecting the fall and falls prevention practice data was also not possible.
	2.2 Were carers and people delivering the interventions aware of participants' assigned intervention during the trial?	Y	
	2.3 If Y/PY/NI to 2.1b or 2.2: Were there deviations from the intended intervention that arose because of the trial context?	NI	"We investigated potential contamination by examining changes in the use of 6-PACK programme components from the baseline to the randomised controlled trial period in the control wards."<br><br>The authors state that "no major protocol deviations or unexpected adverse events occured during the study period" and "no change [in the use of 6-PACK programme components] was observed on the control wards, suggesting that contamination was unlikely".
	2.4 If Y/PY to 2.3: Were these deviations likely to have affected the outcome?	NA	
	2.5 If Y/PY/NI to 2.4: Were these deviations from intended intervention balanced between groups?	NA	
	2.6 Was an appropriate analysis used to estimate the effect of assignment to intervention?	PY	It it stated that "all analyses used intention to treat principles". Table 2 in the journal article showed that the number of participants analysed in the intervention and control group were the same as the number of participants randomised.
	2.7 If N/PN/NI to 2.6: Was there potential for a substantial impact (on the result) of the failure to analyse participants in the group to which they were randomized ?	NA	
	**Risk of bias judgement**	**Some concerns**	
**Bias due to missing outcome data**	3.1a Were data for this outcome available for all clusters that recruited participants?	PY	"The primary analysis was performed according to intention to treat, including all clusters and participants in the allocated groups" (cited in journal article). This indicates that, even at 12 months follow-up, data were available from all clusters.
	3.1b Were data for this outcome available for all, or nearly all, participants within clusters?	N	Fig 1 in the journal article shows that, between baseline and two months follow-up, 134 participants in the control group and 120 participants in the intervention group discontinued the study.
	3.2 If N/PN/NI to 3.1a or 3.1b: Is there evidence that the result was not biased by missing data?	PY	Figure 1 in the journal article shows that there were small differences between the the experimental and comparator intervention groups in the proportion of missing data due to deaths, withdrawals and loss to follow-up.
	3.3 If N/PN to 3.2 Could missingness in the outcome depend on its true value?	NA	
	3.4 If Y/PY/NI to 3.3: Is it likely that missingness in the outcome depended on its true value?	NA	
	**Risk of bias judgement**	**Low**	
**Bias in measurement of the outcome**	4.1 Was the method of measuring the outcome inappropriate?	PN	"Drug compliance is measured using the ©MMAS-8.30–32 The numbers of drug–drug interactions, drug overuse and underuse, as well as potentially inappropriate medication are assessed for the intervention group at 2months, based on STRIPA information including the medical diagnoses from the index hospitalisation and the updated medication list from the 2-month telephone follow-up" (cited in trial protocol Adam et al. 2019).
	4.2 Could measurement or ascertainment of the outcome have differed between intervention groups?	PN	"Blinded team members collected follow-up and outcome data through telephone interviews with the participants or their proxies at 2, 6, and 12 months post-randomisation" (cited in journal article).
	4.3a If N/PN/NI to 4.1 and 4.2: Were outcome assessors aware that a trial was taking place?	Y	
	4.3b If Y/PY/NI to 4.3a: Were outcome assessors aware of the intervention received by study participants?	PN	"Blinded team members collected follow-up and outcome data through telephone interviews with the participants or their proxies at 2, 6, and 12 months post-randomisation" (cited in journal article).
	4.4 If Y/PY/NI to 4.3b: Could assessment of the outcome have been influenced by knowledge of intervention received?	NA	
	4.5 If Y/PY/NI to 4.4: Is it likely that assessment of the outcome was influenced by knowledge of intervention received?	NA	
	**Risk of bias judgement**	**Low**	

**Bias in selection of the reported result**	5.1 Were the data that produced this result analysed in accordance with a pre-specified analysis plan that was finalized before unblinded outcome data were available for analysis?	PY	All statistical analyses including a description of all relevant derivations of variables will be described in a statistical analysis plan before the end of recruitment and without inspection of the data" (cited in the trial protocol Adam et al. 2019).<br><br>"Continuous outcomes will be analysed using mixed-effects linear model with a fixed effect for the intervention group and random effects for centre and treating physician, adjusted for the baseline value as a covariate if available" (cited in journal article). This method of analysing the data on drug overuse is the same as what was actually done (as described in the journal article): "Between group differences for continuou outcomes were analysed using mixed effects linear regression models with fxed and random effects, and<br>adjustment for baseline values" (cited in journal article).
	5.2 ... multiple eligible outcome measurements (e.g. scales, definitions, time points) within the outcome domain?	PN	It is stated in the trial protocol that drug overuse will be assessed at two months follow-up, and also that information from STRIPA will be used in the assessment.
	5.3 ... multiple eligible analyses of the data?	NI	Unable to find information about this.
	**Risk of bias judgement**		
**Overall bias**	**Risk of bias judgement**	**Some concerns**	

**Unique ID**	Carroll(2012)_A	**Study ID**	1047	**Assessor**	RS
**Ref or Label**		**Aim**	assignment to intervention (the 'intention-to-treat' effect)		
**Experimental**	Fall risk assessment and recommended interventions based on decision support	**Comparator**	Usual care	**Source**	Journal article(s); Non-commercial trial registry record (e.g. ClinicalTrials.gov record); Personal communication with trialist
**Outcome**	Documentation of fall risk (percentage of patients who had their fall risk documented) during 6 months follow-up	**Results**	89% of patients in intervention group vs. 64% of patients in control group (p < .0001)	**Weight**	1

**Domain**	**Signalling question**	**Response**	**Comments**
**Bias arising from the randomization process**	1a.1 Was the allocation sequence random?	NI	In the journal article only a statement was made that the medical units were randomly assigned. Corresponding author Diane Carroll stated in an email that the allocation sequence was generated using a random numbers generator.
	1a.2 Was the allocation sequence concealed until clusters were enrolled and assigned to interventions?	NI	
	1a.3 Did baseline differences between intervention groups suggest a problem with the randomization process?	PN	"Patients in control and intervention units were similar, but patients in the control units were more likely to be younger and of white race and to have commercial insurance. Although none of these differences were significant, we adjusted for potential confounders (Table 2) that had P=.05 to P=.10" (cited in Dykes et al. 2010).
	**Risk of bias judgement**	**Some concerns**	
**Bias arising from the timing of identification or recruitment of participants**	1b.1 Were all the individual participants identified and recruited (if appropriate) before randomization of clusters?	**N**	Some patients were admitted to the hospital units after the clusters were randomised.
	1b.2 If N/PN/NI to 1b.1: Is it likely that selection of individual participants was affected by knowledge of the intervention assigned to the cluster?	**Patient records from all patients admitted to study hospital wards were eligible for participation in this study. It is unlikely that knowledge of the intervention assigned to the ward affected whether patients were admitted or not.**	
	1b.3 Were there baseline imbalances that suggest differential identification or recruitment of individual participants between intervention groups?	**PN**	See description for question 1a.3
	**Risk of bias judgement**	**Low**	Low
**Bias due to deviations from**	2.1a Were participants aware that they were in a trial?	PN	
	2.1b If Y/PY/NI to 2.1a: Were participants aware of their assigned intervention during the trial?	NA	As the intervention was nurse led, blinding of ward nurses and patients was not possible. Blinding of the assessors collecting the fall and falls prevention practice data was also not possible.
	2.2 Were carers and people delivering the interventions aware of participants' assigned intervention during the trial?	Y	
	2.3 If Y/PY/NI to 2.1b or 2.2: Were there deviations from the intended intervention that arose because of the trial context?	NI	Unable to find information about this.

intended interventions	2.4 If Y/PY to 2.3: Were these deviations likely to have affected the outcome?	NA	
	2.5 If Y/PY/NI to 2.4: Were these deviations from intended intervention balanced between groups?	NA	
	2.6 Was an appropriate analysis used to estimate the effect of assignment to intervention?	PY	Figure 2 in the journal article shows that all participants who were randomised were included in the analysis.
	2.7 If N/PN/NI to 2.6: Was there potential for a substantial impact (on the result) of the failure to analyse participants in the group to which they were randomized ?	NA	
	**Risk of bias judgement**	**Some concerns**	
**Bias due to missing outcome data**	3.1a Were data for this outcome available for all clusters that recruited participants?	PY	"The study involved 10 264 patients and 48 250 patient-days. No units withdrew from the study" (cited in Dykes et al. 2010).
	3.1b Were data for this outcome available for all, or nearly all, participants within clusters?	PY	Most likely, as Table 3 and Figure 2 in Dykes et al. 2010 shows that the total number of patients included in the analyses on falls were 5160 in intervention units and 5104 in control units, the same as the number of patients included in the study. These patients were presumably also eligible to be included in the analysis on documentation of fall risk
	3.2 If N/PN/NI to 3.1a or 3.1b: Is there evidence that the result was not biased by missing data?	NA	
	3.3 If N/PN to 3.2 Could missingness in the outcome depend on its true value?	NA	
	3.4 If Y/PY/NI to 3.3: Is it likely that missingness in the outcome depended on its true value?	NA	
	**Risk of bias judgement**	**Low**	
**Bias in measurement of the outcome**	4.1 Was the method of measuring the outcome inappropriate?	PN	"Data abstractors were trained by first reviewing the patient record with the principal investigator together and then independently, both doing same record, until there was at least a 90% agreement. Patient records were reviewed for documentation from the time of admission to the date of record review related to fall risk status and planned interventions. The patient records were searched again for documentation, including all nursing notes, flow sheets, and treatment records, for the implementation of fall prevention interventions."
	4.2 Could measurement or ascertainment of the outcome have differed between intervention groups?	PN	
	4.3a If N/PN/NI to 4.1 and 4.2: Were outcome assessors aware that a trial was taking place?	Y	
	4.3b If Y/PY/NI to 4.3a: Were outcome assessors aware of the intervention received by study participants?	Y	
	4.4 If Y/PY/NI to 4.3b: Could assessment of the outcome have been influenced by knowledge of intervention received?	PN	
	4.5 If Y/PY/NI to 4.4: Is it likely that assessment of the outcome was influenced by knowledge of intervention received?	NA	
	**Risk of bias judgement**	**Low**	
**Bias in selection of the reported result**	5.1 Were the data that produced this result analysed in accordance with a pre-specified analysis plan that was finalized before unblinded outcome data were available for analysis?	NI	The study was conducted for six months, from 01.01.2009 through 30.06.2009. The ClinicalTrials.Gov entry (documentation of planned and completed tailored interventions) was submitted June 18 2010.
	5.2 ... multiple eligible outcome measurements (e.g. scales, definitions, time points) within the outcome domain?	NI	Unable to find information about this.
	5.3 ... multiple eligible analyses of the data?	NI	Unable to find information about this.
	**Risk of bias judgement**		
**Overall bias**	**Risk of bias judgement**	**High**	A high overall Rob was given because of suspicion that there may have been bias in selection of the reported result, in addition to two other domains at some concerns for bias.

**Unique ID**	Clemson (2024)_A	**Study ID**	6277	**Assessor**	RS
**Ref or Label**		**Aim**	assignment to intervention (the 'intention-to-treat' effect)		
**Experimental**	iSOLVE intervention	**Comparator**	Usual general practice	**Source**	Journal article(s); Trial protocol; Non-commercial trial registry record (e.g. ClinicalTrials.gov record)
**Outcome**	Rate of falls, self-reported by patients using daily fall calendar mailed on a monthly basis, in intervention group compared with control group over 12 months follow-up	**Results**	Incidence rate ratio 0.96 (95% CI 0.77 - 1.20)	**Weight**	

Domain	Signalling question	Response	Comments
	1a.1 Was the allocation sequence random?	Y	"An independent statistician not involved in

Bias arising from the randomization process	1a.2 Was the allocation sequence concealed until clusters were enrolled and assigned to interventions?	Y	recruiting the GP practices will generate the randomisation sequence using a pre-determined block design." (cited in trial registry).<br><br>"computer-generated block randomization at a distant site by a researcher (JS) not involved with allocation or data collection." (cited in journal article).
	1a.3 Did baseline differences between intervention groups suggest a problem with the randomization process?	NI	The only baseline characteristics of participating GPs that are reported are the number of clusters and GPs randomised to each intervention, the number of years practicing as a GP, and GPs' estimated percentage of patients aged 65 years or older.
	**Risk of bias judgement**	**Low**	
Bias arising from the timing of identification or recruitment of participants	1b.1 Were all the individual participants identified and recruited (if appropriate) before randomization of clusters?	**Y**	"Practices were randomized individually after completing GP recruitment within each practice" (cited in journal article).<br><br>GP practices will be randomised to either the intervention or control arm once recruitment procedures are completed and informed consent is provided. This will be achieved using opaque concealed envelopes. (cited in trial registry).
	1b.2 If N/PN/NI to 1b.1: Is it likely that selection of individual participants was affected by knowledge of the intervention assigned to the cluster?		
	1b.3 Were there baseline imbalances that suggest differential identification or recruitment of individual participants between intervention groups?	**NI**	Please see the answer to signalling question 1a.3.
	**Risk of bias judgement**	**Low**	Low
Bias due to deviations from intended interventions	2.1a Were participants aware that they were in a trial?	Y	GPs were aware that they were in a trial. "The project manager will inform the GP practice if they are in the control or intervention group" (cited in trial registry).
	2.1b If Y/PY/NI to 2.1a: Were participants aware of their assigned intervention during the trial?	Y	GPs were aware of their assigned intervention, but they were not aware of what intervention their patients were assigned to. "The project manager will request for patients not to reveal their participation in the study to their GPs or staff at the GP practice to ensure GP practices are blinded from patient allocation. This is intended to minimise bias in health care provision by the GPs and GP practice staff as the GPs will be asked to administer the intervention to all their older patients aged 65 years and over as appropriate (note, this trial is a translational project and the intent is that they will adopt the process as usual care). However, this does not blind the GP to being in the intervention arm." (cited in trial registry).
	2.2 Were carers and people delivering the interventions aware of participants' assigned intervention during the trial?	N	
	2.3 If Y/PY/NI to 2.1b or 2.2: Were there deviations from the intended intervention that arose because of the trial context?	Y	"It is likely that, given the pragmatic nature of the project, multiple elements of introduced bias and potential contamination occurred that influenced the embedded trial results." (cited in journal article).
	2.4 If Y/PY to 2.3: Were these deviations likely to have affected the outcome?	Y	"In a subset of GP patients (n = 560), no significant effect was observed in reducing the rate of falls (IRR = 0.96). The pragmatic nature of the project and potential contamination across multiple elements likely influenced this outcome." (cited in journal article).<br><br>Also, there seems to have been an increase in fall prevention activities during the study period that was general to the area, which may have influenced the result: "However, an area-wide survey of GPs (n = 562) revealed an increase in fall prevention referrals to AHPs over 5 years, from 70 to 82% (p = 0.028)." (cited in journal article).
	2.5 If Y/PY/NI to 2.4: Were these deviations from intended intervention balanced between groups?	N	Clinics in the usual care group was likely more influenced than clinics in the iSOLVE intervention group, because at the time the study took place, there was ongoing work that may have increased awareness of fall prevention, potentially closing the gap that may have otherwise existed in fall prevention activities between intervention group. "For example, parallel work in upskilling AHPs, trial surverys, and area-wide surveys may have raised awareness of fall prevention options. The study focused on a specific geographic area, and the findings might not be generalizable to all GP populations without further research." (cited in journal article).

	2.6 Was an appropriate analysis used to estimate the effect of assignment to intervention?	Y	"All analyses will be pre-planned, conducted while masked to group allocation and will use an intention-to-treat approach" (cited in trial registry).
	2.7 If N/PN/NI to 2.6: Was there potential for a substantial impact (on the result) of the failure to analyse participants in the group to which they were randomized ?	NA	
	**Risk of bias judgement**	**High**	
**Bias due to missing outcome data**	3.1a Were data for this outcome available for all clusters that recruited participants?	N	No, one practice (3 GPs) withdrew post-randomisation.
	3.1b Were data for this outcome available for all, or nearly all, participants within clusters?	Y	A total of 267 (97%) of the 275 patient participants randomised to the iSOLVE intervention completed their 12-month fall calendars. A total of 273 (96%) of the 285 patient participants randomised to the control group completed their 12-month fall calendars.
	3.2 If N/PN/NI to 3.1a or 3.1b: Is there evidence that the result was not biased by missing data?	Y	There is little missing data, and there is a similar amount of missing data across the two study groups. With four percent missing data in the experimental intervention group and three percent missing data in the control group, it is very unlikely that result would have been substantially different, even if the number of falls in the missing 12-month fall calendars were systematically different than the number of falls reported in the fall calendars that were returned.
	3.3 If N/PN to 3.2 Could missingness in the outcome depend on its true value?	NA	
	3.4 If Y/PY/NI to 3.3: Is it likely that missingness in the outcome depended on its true value?	NA	
	**Risk of bias judgement**	**Low**	
**Bias in measurement of the outcome**	4.1 Was the method of measuring the outcome inappropriate?	N	"During the 12-month follow-up, patient participants were asked to self-report any falls in a monthly diary that was mailed to the blinded" research assistant (cited in journal article).<br><br>"Fall calendars also prompt patients to record the circumstances of a fall, whether any injuries occurred and whether any medical help was sought as a result of the fall. If a person does not return their fall calendar, they are telephoned to determine whether they have fallen." (cited in trial protocol).
	4.2 Could measurement or ascertainment of the outcome have differed between intervention groups?	N	Across the two intervention groups, the same method for ascertaining the number of falls was used at similar time points.
	4.3a If N/PN/NI to 4.1 and 4.2: Were outcome assessors aware that a trial was taking place?	Y	Patient participants were aware that a trial was taking place.
	4.3b If Y/PY/NI to 4.3a: Were outcome assessors aware of the intervention received by study participants?	Y	Patient participants, who were the outcome assessors, were aware of the intervention to which they were allocated and the intervention group to which their GP was allocated.
	4.4 If Y/PY/NI to 4.3b: Could assessment of the outcome have been influenced by knowledge of intervention received?	N	It is unlikely that patient participants' knowledge of which intervention their GP was allocated to would have influenced the nunmber of falls they reported in their monthly fall calendars.
	4.5 If Y/PY/NI to 4.4: Is it likely that assessment of the outcome was influenced by knowledge of intervention received?	NA	
	**Risk of bias judgement**	**Low**	

Bias in selection of the reported result	5.1 Were the data that produced this result analysed in accordance with a pre-specified analysis plan that was finalized before unblinded outcome data were available for analysis?	Y	The planned statistical analysis reported in the trial registry seems to correspond to the analysis as reported in the journal article. However, I (RS) am unaware of what date the planned statistical analysis was documented in the trial registry, and whether this was prior to unblinded outcome data were available for analysis. It says in the trial registry that people analysing the results/data are blinded.<br><br>Trial registry:<br>"The number of falls per person-year will be analysed using negative binomial regression models to estimate the difference in rates between the groups after one year (primary outcome). All analyses will be pre-planned, conducted while masked to group allocation and will use an intention-to-treat approach." (cited in trial registry).<br><br>Journal article.<br>"To analyze total falls per patient-participant, we used negative<br>binomial regression with days in the trial as the exposure, adjusting for<br>stratification by practice size (low/high) and allowing for clustering by GP<br>using the generalized estimating equations (GEE) approach with an
	5.2 ... multiple eligible outcome measurements (e.g. scales, definitions, time points) within the outcome domain?	N	
	5.3 ... multiple eligible analyses of the data?	N	
	**Risk of bias judgement**		
**Overall bias**	**Risk of bias judgement**	**High**	

**Unique ID**	Clemson (2024)_B	**Study ID**	6277	**Assessor**	RS
**Ref or Label**		**Aim**	assignment to intervention (the 'intention-to-treat' effect)		
**Experimental**	iSOLVE intervention	**Comparator**	Usual general practice	**Source**	
**Outcome**	Changes in GPs' engagement in fall prevention activities, including risk assessment, medication reviews, and providing advice, compared to the control group, at three months follow-up	**Results**	0.90 units (95% CI 0.33, 1.46)	**Weight**	

Domain	Signalling question	Response	Comments
Bias arising from the randomization process	1a.1 Was the allocation sequence random?	Y	"An independent statistician not involved in recruiting the GP practices will generate the randomisation sequence using a pre-determined block design." (cited in trial registry).<br><br>"computer-generated block randomization at a distant site by a researcher (JS) not involved with allocation or data collection." (cited in journal article).
	1a.2 Was the allocation sequence concealed until clusters were enrolled and assigned to interventions?	Y	
	1a.3 Did baseline differences between intervention groups suggest a problem with the randomization process?	NI	The only baseline characteristics of participating GPs that are reported are the number of clusters and GPs randomised to each intervention, the number of years practicing as a GP, and GPs' estimated percentage of patients aged 65 years or older.
	**Risk of bias judgement**	**Low**	
Bias arising from the timing of identification or recruitment of participants	1b.1 Were all the individual participants identified and recruited (if appropriate) before randomization of clusters?	**Y**	"Practices were randomized individually after completing GP recruitment within each practice" (cited in journal article).<br><br>GP practices will be randomised to either the intervention or control arm once recruitment procedures are completed and informed consent is provided. This will be achieved using opaque concealed envelopes. (cited in trial registry).
	1b.2 If N/PN/NI to 1b.1: Is it likely that selection of individual participants was affected by knowledge of the intervention assigned to the cluster?		
	1b.3 Were there baseline imbalances that suggest differential identification or recruitment of individual participants between intervention groups?	**NI**	Please see the answer to signalling question 1a.3.
	**Risk of bias judgement**	**Low**	Low
	2.1a Were participants aware that they were in a trial?	Y	GPs were aware that they were in a trial. "The project manager will inform the GP practice if they are in the control or intervention group" (cited in trial registry).

Bias due to deviations from intended interventions	2.1b If Y/PY/NI to 2.1a: Were participants aware of their assigned intervention during the trial?	Y	GPs were aware of their assigned intervention, but they were not aware of what intervention their patients were assigned to. "The project manager will request for patients not to reveal their participation in the study to their GPs or staff at the GP practice to ensure GP practices are blinded from patient allocation. This is intended to minimise bias in health care provision by the GPs and GP practice staff as the GPs will be asked to administer the intervention to all their older patients aged 65 years and over as appropriate (note, this trial is a translational project and the intent is that they will adopt the process as usual care). However, this does not blind the GP to being in the intervention arm." (cited in trial registry).
	2.2 Were carers and people delivering the interventions aware of participants' assigned intervention during the trial?	N	
	2.3 If Y/PY/NI to 2.1b or 2.2: Were there deviations from the intended intervention that arose because of the trial context?	Y	"It is likely that, given the pragmatic nature of the project, multiple elements of introduced bias and potential contamination occurred that influenced the embedded trial results." (cited in journal article).
	2.4 If Y/PY to 2.3: Were these deviations likely to have affected the outcome?	Y	"In a subset of GP patients (n = 560), no significant effect was observed in reducing the rate of falls (IRR = 0.96). The pragmatic nature of the project and potential contamination across multiple elements likely influenced this outcome." (cited in journal article).<br><br>Also, there seems to have been an increase in fall prevention activities during the study period that was general to the area, which may have influenced the result: "However, an area-wide survey of GPs (n = 562) revealed an increase in fall prevention referrals to AHPs over 5 years, from 70 to 82% (p = 0.028)." (cited in journal article).
	2.5 If Y/PY/NI to 2.4: Were these deviations from intended intervention balanced between groups?	N	Clinics in the usual care group was likely more influenced than clinics in the iSOLVE intervention group, because at the time the study took place, there was ongoing work that may have increased awareness of fall prevention, potentially closing the gap that may have otherwise existed in fall prevention activities between intervention group. "For example, parallel work in upskilling AHPs, trial surverys, and area-wide surveys may have raised awareness of fall prevention options. The study focused on a specific geographic area, and the findings might not be generalizable to all GP populations without further research." (cited in journal article).
	2.6 Was an appropriate analysis used to estimate the effect of assignment to intervention?	Y	"All analyses will be pre-planned, conducted while masked to group allocation and will use an intention-to-treat approach" (cited in trial registry).
	2.7 If N/PN/NI to 2.6: Was there potential for a substantial impact (on the result) of the failure to analyse participants in the group to which they were randomized ?	NA	
	**Risk of bias judgement**	**High**	
Bias due to missing outcome data	3.1a Were data for this outcome available for all clusters that recruited participants?	N	No, one practice (3 GPs) withdrew post-randomisation.
	3.1b Were data for this outcome available for all, or nearly all, participants within clusters?	N	A total of 68 of the 75 participating GPs responded to all three questions about assessing risk, reviewing medications, and providing advice at both baseline and 12 months. Of the GPs who responded, 31 of a total of 32 were in the experimental group and 37 of a total of 43 were in the control group.
	3.2 If N/PN/NI to 3.1a or 3.1b: Is there evidence that the result was not biased by missing data?	Y	The reasons for loss to follow-up were reported. In the experimental group, the 1 GP left the practice. In the control group, 3 GPs withdrew, 3 GPs left the practice, and 1 GP declined.
	3.3 If N/PN to 3.2 Could missingness in the outcome depend on its true value?	NA	
	3.4 If Y/PY/NI to 3.3: Is it likely that missingness in the outcome depended on its true value?	NA	
	**Risk of bias judgement**	**Low**	
	4.1 Was the method of measuring the outcome inappropriate?	PN	The method of ascertaining GPs' engagement in fall prevention practices were pre-specified, but there is no mention of the validity of this method: "The GPs complete a survey at baseline, 3 and 12 months, comprising a combination of open-ended and Likert scale response questions. The questions cover beliefs about fall prevention, knowledge of local fall prevention services, management of fall prevention and any changes in practice, and referral patterns." (cited in trial protocol).

**Bias in measurement of the outcome**	4.2 Could measurement or ascertainment of the outcome have differed between intervention groups?	N	To ascertain the outcome of GPs' engagement in fall prevention activities, the same method was used at the same time points across the two groups. In the trial registry it says that the survey has undergone pilot testing for readability and relevance, but the results of this pilot testing are not reported: "The survey has been designed specifically for this study and has undergone pilot testing for readability and relevance." (cited in trial registry).
	4.3a If N/PN/NI to 4.1 and 4.2: Were outcome assessors aware that a trial was taking place?	Y	GPs were aware that they were in a trial. "The project manager will inform the GP practice if they are in the control or intervention group" (cited in trial registry).
	4.3b If Y/PY/NI to 4.3a: Were outcome assessors aware of the intervention received by study participants?	N	GPs were aware of their assigned intervention, but they were not aware of what intervention their patients were assigned to. "The project manager will request for patients not to reveal their participation in the study to their GPs or staff at the GP practice to ensure GP practices are blinded from patient allocation. This is intended to minimise bias in health care provision by the GPs and GP practice staff as the GPs will be asked to administer the intervention to all their older patients aged 65 years and over as appropriate (note, this trial is a translational project and the intent is that they will adopt the process as usual care). However, this does not blind the GP to being in the intervention arm." (cited in trial registry)
	4.4 If Y/PY/NI to 4.3b: Could assessment of the outcome have been influenced by knowledge of intervention received?	NA	
	4.5 If Y/PY/NI to 4.4: Is it likely that assessment of the outcome was influenced by knowledge of intervention received?	NA	
	**Risk of bias judgement**	**Low**	
**Bias in selection of the reported result**	5.1 Were the data that produced this result analysed in accordance with a pre-specified analysis plan that was finalized before unblinded outcome data were available for analysis?	Y	The planned statistical analysis reported in the trial registry and in the trial protocol seems to correspond to the analysis as reported in the journal article. However, I (RS) am unaware of what date the planned statistical analysis was documented in the trial registry, and whether this was prior to unblinded outcome data were available for analysis. It says in the trial registry that people analysing the results/data are blinded.<br><br>Trial registry:<br>"Survey analysis will include frequency and descriptive statistics." (cited in trial registry).<br><br>Who is / are masked / blinded? The people analysing the results /data<br><br>Trial protocol:<br>"GP engagement in fall prevention management and GP referral practices will be assessed using questions 11 and 12, respectively, of the 12-month GP survey and corresponding questions on the baseline survey. For management, a score from 0 to 3 will be allocated to each of the first three items and summed to give a total out of 9. The total score for each GP at 12 months will be compared between groups using
	5.2 ... multiple eligible outcome measurements (e.g. scales, definitions, time points) within the outcome domain?	PN	Please see the answer to signalling question 5.1.
	5.3 ... multiple eligible analyses of the data?	PN	Please see the answer to signalling question 5.1.
	**Risk of bias judgement**		Unpredictable
**Overall bias**	**Risk of bias judgement**	**High**	High risk of bias due to deviations from indtended interventions.

**Unique ID**	Dykes(2010)_A	**Study ID**	1305	**Assessor**	RS
**Ref or Label**		**Aim**	assignment to intervention (the 'intention-to-treat' effect)		
**Experimental**	Fall Prevention Tool Kit (FPTK) using health information technology (HIT)	**Comparator**	Usual care related to fall prevention, i.e. no intervention	**Source**	Journal article(s); Non-commercial trial registry record (e.g. ClinicalTrials.gov record)
**Outcome**	Rate of patient falls per 1000 patient-days at six months follow-up in intervention group compared with control group (sensitivity analysis with patients aged 65 years or older)	**Results**	Mean Difference 2.08 (95% CI 0.61, 3.56; p = 0.003)	**Weight**	

**Domain**	**Signalling question**	**Response**	**Comments**
	1a.1 Was the allocation sequence random?	NI	"We used a stratified, cluster randomization

**Bias arising from the randomization process**	1a.2 Was the allocation sequence concealed until clusters were enrolled and assigned to interventions?	NI	design, with the randomized intervention at the unit (cluster) level within hospital (strata) and falls measured at the patient level" (cited in Dykes et al. 2010).
	1a.3 Did baseline differences between intervention groups suggest a problem with the randomization process?	PN	"Patients in control and intervention units were similar, but patients in the control units were more likely to be younger and of white race and to have commercial insurance. Although none of these differences were significant, we adjusted for potential confounders (Table 2) that had P=.05 to P=.10" (cited in Dykes et al. 2010).
	**Risk of bias judgement**	**Some concerns**	
**Bias arising from the timing of identification or recruitment of participants**	1b.1 Were all the individual participants identified and recruited (if appropriate) before randomization of clusters?	**N**	As the study was conducted in four short-stay hospitals, most individual participants were admitted after the clusters were allocated to intervention or control groups.
	1b.2 If N/PN/NI to 1b.1: Is it likely that selection of individual participants was affected by knowledge of the intervention assigned to the cluster?	**Given that patients were admitted to hospital units it is considered unlikely that knowledge of the intervention assigned to the cluster affected which unit the patient was admitted to.**	
	1b.3 Were there baseline imbalances that suggest differential identification or recruitment of individual participants between intervention groups?	**PN**	See description for question 1a.3.
	**Risk of bias judgement**	**Low**	Low
**Bias due to deviations from intended interventions**	2.1a Were participants aware that they were in a trial?	PN	
	2.1b If Y/PY/NI to 2.1a: Were participants aware of their assigned intervention during the trial?	NA	As the intervention was nurse led, blinding of ward nurses and patients was not possible. Blinding of the assessors collecting the fall and falls prevention practice data was also not possible.
	2.2 Were carers and people delivering the interventions aware of participants' assigned intervention during the trial?	Y	
	2.3 If Y/PY/NI to 2.1b or 2.2: Were there deviations from the intended intervention that arose because of the trial context?	NI	Unable to find information about this.
	2.4 If Y/PY to 2.3: Were these deviations likely to have affected the outcome?	NA	
	2.5 If Y/PY/NI to 2.4: Were these deviations from intended intervention balanced between groups?	NA	
	2.6 Was an appropriate analysis used to estimate the effect of assignment to intervention?	PY	Figure 2 in the journal article shows that all participants who were randomised were included in the analysis.
	2.7 If N/PN/NI to 2.6: Was there potential for a substantial impact (on the result) of the failure to analyse participants in the group to which they were randomized ?	NA	
	**Risk of bias judgement**	**Some concerns**	
**Bias due to missing outcome data**	3.1a Were data for this outcome available for all clusters that recruited participants?	PY	"The study involved 10 264 patients and 48 250 patient-days. No units withdrew from the study" (cited in Dykes et al. 2010).
	3.1b Were data for this outcome available for all, or nearly all, participants within clusters?	PY	Table 3 and Figure 2 in Dykes et al. 2010 shows that the total number of patients included in the analyses on falls were 5160 in intervention units and 5104 in control units, the same as the number of patients included in the study.
	3.2 If N/PN/NI to 3.1a or 3.1b: Is there evidence that the result was not biased by missing data?	NA	
	3.3 If N/PN to 3.2 Could missingness in the outcome depend on its true value?	NA	
	3.4 If Y/PY/NI to 3.3: Is it likely that missingness in the outcome depended on its true value?	NA	
	**Risk of bias judgement**	**Low**	
**Bias in measurement of the outcome**	4.1 Was the method of measuring the outcome inappropriate?	PN	
	4.2 Could measurement or ascertainment of the outcome have differed between intervention groups?	PN	"Reporting of patient falls and injurious falls is required at all hospitals and routinely recorded in an event reporting system in all units by the clinician caring for the patient at the time of a fall. Incidents are validated by unit managers and hospital quality personnel" (cited in Dykes et al. 2010).
	4.3a If N/PN/NI to 4.1 and 4.2: Were outcome assessors aware that a trial was taking place?	PY	
	4.3b If Y/PY/NI to 4.3a: Were outcome assessors aware of the intervention received by study participants?	PY	Given that patient falls were recorded by the clinician caring for the patient at the time of a fall, it is likely that the clinician was aware of the intervention received by study participants.
	4.4 If Y/PY/NI to 4.3b: Could assessment of the outcome have been influenced by knowledge of intervention received?	PN	A well established routine for recording patient falls was in place. This routine was unlikely to have been influenced by knowledge of the intervention received by study participants.
	4.5 If Y/PY/NI to 4.4: Is it likely that assessment of the outcome was influenced by knowledge of intervention received?	NA	

	**Risk of bias judgement**	**Low**	
**Bias in selection of the reported result**	5.1 Were the data that produced this result analysed in accordance with a pre-specified analysis plan that was finalized before unblinded outcome data were available for analysis?	N	The study was conducted for six months, from 01.01.2009 through 30.06.2009. The ClinicalTrials.Gov entry (rate of patient falls per 1000 patient days) was submitted June 18 2010.
	5.2 ... multiple eligible outcome measurements (e.g. scales, definitions, time points) within the outcome domain?	NI	Unable to find information about this as there we do not have a trial protocol or statistical analysis plan. What is written in the method section of the journal articles and in ClinicalTrials.gov could have been updated after selection of the reported result.
	5.3 ... multiple eligible analyses of the data?	NI	
	**Risk of bias judgement**		
**Overall bias**	**Risk of bias judgement**	**High**	Three individual domains at some concerns.

**Unique ID**	Dykes(2010)_B	**Study ID**	1305	**Assessor**	RS
**Ref or Label**		**Aim**	assignment to intervention (the 'intention-to-treat' effect)		
**Experimental**	Fall Prevention Tool Kit (FPTK) using health information technology (HIT)	**Comparator**	Usual care related to fall prevention, i.e. no intervention	**Source**	Journal article(s); Non-commercial trial registry record (e.g. ClinicalTrials.gov record)
**Outcome**	Adherence to intervention protocol (Morse Falls Scale completion)	**Results**	94% in IG, 81% in CG	**Weight**	

Domain	Signalling question	Response	Comments
**Bias arising from the randomization process**	1a.1 Was the allocation sequence random?	NI	"We used a stratified, cluster randomization design, with the randomized intervention at the unit (cluster) level within hospital (strata) and falls measured at the patient level" (cited in Dykes et al. 2010).
	1a.2 Was the allocation sequence concealed until clusters were enrolled and assigned to interventions?	NI	
	1a.3 Did baseline differences between intervention groups suggest a problem with the randomization process?	PN	"Patients in control and intervention units were similar, but patients in the control units were more likely to be younger and of white race and to have commercial insurance. Although none of these differences were significant, we adjusted for potential confounders (Table 2) that had P=.05 to P=.10" (cited in Dykes et al. 2010).
	**Risk of bias judgement**	**Some concerns**	
**Bias arising from the timing of identification or recruitment of participants**	1b.1 Were all the individual participants identified and recruited (if appropriate) before randomization of clusters?	**N**	As the study was conducted in four short-stay hospitals, most individual participants were admitted after the clusters were allocated to intervention or control groups.
	1b.2 If N/PN/NI to 1b.1: Is it likely that selection of individual participants was affected by knowledge of the intervention assigned to the cluster?	**Given that patients were admitted to hospital units it is considered unlikely that knowledge of the intervention assigned to the cluster affected which unit the patient was admitted to.**	
	1b.3 Were there baseline imbalances that suggest differential identification or recruitment of individual participants between intervention groups?	**PN**	See description for question 1a.3.
	**Risk of bias judgement**	**Low**	Low
**Bias due to deviations from intended interventions**	2.1a Were participants aware that they were in a trial?	PN	
	2.1b If Y/PY/NI to 2.1a: Were participants aware of their assigned intervention during the trial?	NA	As the intervention was nurse led, blinding of ward nurses and patients was not possible. Blinding of the assessors collecting the fall and falls prevention practice data was also not possible.
	2.2 Were carers and people delivering the interventions aware of participants' assigned intervention during the trial?	Y	
	2.3 If Y/PY/NI to 2.1b or 2.2: Were there deviations from the intended intervention that arose because of the trial context?	NI	Unable to find information about this.
	2.4 If Y/PY to 2.3: Were these deviations likely to have affected the outcome?	NA	
	2.5 If Y/PY/NI to 2.4: Were these deviations from intended intervention balanced between groups?	NA	
	2.6 Was an appropriate analysis used to estimate the effect of assignment to intervention?	PY	Figure 2 in the journal article shows that all participants who were randomised were included in the analysis.
	2.7 If N/PN/NI to 2.6: Was there potential for a substantial impact (on the result) of the failure to analyse participants in the group to which they were randomized ?	NA	
	**Risk of bias judgement**	**Some concerns**	
	3.1a Were data for this outcome available for all clusters that recruited participants?	PY	"The study involved 10 264 patients and 48 250 patient-days. No units withdrew from the study" (cited in Dykes et al. 2010).

Bias due to missing outcome data	3.1b Were data for this outcome available for all, or nearly all, participants within clusters?	PY	Table 3 and Figure 2 in Dykes et al. 2010 shows that the total number of patients included in the analyses on falls were 5160 in intervention units and 5104 in control units, the same as the number of patients included in the study.
	3.2 If N/PN/NI to 3.1a or 3.1b: Is there evidence that the result was not biased by missing data?	NA	
	3.3 If N/PN to 3.2 Could missingness in the outcome depend on its true value?	NA	
	3.4 If Y/PY/NI to 3.3: Is it likely that missingness in the outcome depended on its true value?	NA	
	**Risk of bias judgement**	**Low**	
Bias in measurement of the outcome	4.1 Was the method of measuring the outcome inappropriate?	PN	"Adherence to the protocol was measured through random assessment of MFS completion in control units and the use of FPTK components (including MFS completion) in intervention units" (cited in journal article).
	4.2 Could measurement or ascertainment of the outcome have differed between intervention groups?	NI	Unable to find information
	4.3a If N/PN/NI to 4.1 and 4.2: Were outcome assessors aware that a trial was taking place?	NI	Unable to find information
	4.3b If Y/PY/NI to 4.3a: Were outcome assessors aware of the intervention received by study participants?	NI	Unable to find information
	4.4 If Y/PY/NI to 4.3b: Could assessment of the outcome have been influenced by knowledge of intervention received?	PN	Adherence to the protocol was quantified by reviewing the a sample of MSF. The percentage of adherence to daily MFS completion is likely to have been quantified in the same way in both groups.
	4.5 If Y/PY/NI to 4.4: Is it likely that assessment of the outcome was influenced by knowledge of intervention received?	NA	
	**Risk of bias judgement**	**Some concerns**	
Bias in selection of the reported result	5.1 Were the data that produced this result analysed in accordance with a pre-specified analysis plan that was finalized before unblinded outcome data were available for analysis?	N	The study was conducted for six months, from 01.01.2009 through 30.06.2009. The ClinicalTrials.Gov entry (rate of patient falls per 1000 patient days) was submitted June 18 2010.
	5.2 ... multiple eligible outcome measurements (e.g. scales, definitions, time points) within the outcome domain?	NI	Unable to find information about this as there we do not have a trial protocol or statistical analysis plan. What is written in the method section of the journal articles and in ClinicalTrials.gov could have been updated after selection of the reported result.
	5.3 ... multiple eligible analyses of the data?	NI	
	**Risk of bias judgement**		
Overall bias	**Risk of bias judgement**	**High**	An overall RoB rating of high was given because of four domains at some concerns for RoB.

**Unique ID**	Ganz(2022)_A	**Study ID**	5477	**Assessor**	RS
**Ref or Label**		**Aim**	assignment to intervention (the 'intention-to-treat' effect)		
**Experimental**	Strategies to Reduce Injuries and Develop Confidence in Elders (STRIDE)Strategies to Reduce Injuries and Develop Confidence in Elders (STRIDE)	**Comparator**	Enhanced usual care	**Source**	Journal article(s); Trial protocol; Statistical analysis plan (SAP); Non-commercial trial registry record (e.g. ClinicalTrials.gov record)
**Outcome**	Rate of falls per 100 person-years during 27 months follow-up in intervention group compared with control group	**Results**	Rate ratio 0.97 (95% CI 0.93, 1.00; p = 0.048)	**Weight**	

Domain	Signalling question	Response	Comments
Bias arising from the randomization process	1a.1 Was the allocation sequence random?	Y	"A published SAS macro was used to conduct the covariate-constrained randomization (Chaudhary and Moulton, 2006; Greene, 2017). The macro generates a set of randomizations that satisfy balance on practice size, geography and race/ethnicity both within and across strata. From this set of valid assignments, one was selected at random. Only the trial biostatisticians participated in the generation of the randomization. No one else from the trial was involved in the process. To minimize the risk of selection bias, practice site names were masked during the process. The practice site randomization assignments to treatment groups were released to the clinical sites only after careful vetting of the entire randomization process by the trial biostatisticians." (SAP)
	1a.2 Was the allocation sequence concealed until clusters were enrolled and assigned to interventions?	Y	
	1a.3 Did baseline differences between intervention groups suggest a problem with the randomization process?	PN	Available baseline characteristics were well balanced between intervention and control groups.

	**Risk of bias judgement**	**Low**	
**Bias arising from the timing of identification or recruitment of participants**	1b.1 Were all the individual participants identified and recruited (if appropriate) before randomization of clusters?	**N**	Participants were recruited after randomisation was completed.
	1b.2 If N/PN/NI to 1b.1: Is it likely that selection of individual participants was affected by knowledge of the intervention assigned to the cluster?	**This is a large study conducted in 86 primary care practices in 10 health systems across the United States, so selection of individual participants was most likely not affected by knowledge of the intervention assigned to the cluster.**	
	1b.3 Were there baseline imbalances that suggest differential identification or recruitment of individual participants between intervention groups?	**PN**	See 1a.3.
	**Risk of bias judgement**	**Low**	Low
**Bias due to deviations from intended interventions**	2.1a Were participants aware that they were in a trial?	Y	Participants were aware that they were in a trial and could not be blinded to group assignment.
	2.1b If Y/PY/NI to 2.1a: Were participants aware of their assigned intervention during the trial?	Y	It was not possible to blind nurses who delivered this multifactorial fall prevention intervention.
	2.2 Were carers and people delivering the interventions aware of participants' assigned intervention during the trial?	Y	
	2.3 If Y/PY/NI to 2.1b or 2.2: Were there deviations from the intended intervention that arose because of the trial context?	NI	"Being aware of group assignment could have led intervention participants to take actions to reduce falls independent of actions agreed upon with the falls care manager, or led to reporting bias for the non-adjudicated outcomes evaluated in the current study; the direction of this latter bias is unclear" (journal article).
	2.4 If Y/PY to 2.3: Were these deviations likely to have affected the outcome?	NA	
	2.5 If Y/PY/NI to 2.4: Were these deviations from intended intervention balanced between groups?	NA	
	2.6 Was an appropriate analysis used to estimate the effect of assignment to intervention?	Y	"We conducted all analyses using an “intention to treat” approach" (journal article).<br><br>As to the proportion of participants who provided data on the outcome: "Completion of followup interviews was greater than 93% at each time point. Most participants completed all (71.8%) or all but one (9.2%) of the follow-up interviews." (journal article).
	2.7 If N/PN/NI to 2.6: Was there potential for a substantial impact (on the result) of the failure to analyse participants in the group to which they were randomized ?	NA	
	**Risk of bias judgement**	**Some concerns**	
**Bias due to missing outcome data**	3.1a Were data for this outcome available for all clusters that recruited participants?	Y	Figure 1 in the journal article shows that all study participants were included in the analysis.
	3.1b Were data for this outcome available for all, or nearly all, participants within clusters?	PY	"Completion of followup interviews was greater than 93% at each time point.<br>Most participants completed all (71.8%) or all but one (9.2%) of the follow-up interviews." (journal article
	3.2 If N/PN/NI to 3.1a or 3.1b: Is there evidence that the result was not biased by missing data?	NA	
	3.3 If N/PN to 3.2 Could missingness in the outcome depend on its true value?	NA	
	3.4 If Y/PY/NI to 3.3: Is it likely that missingness in the outcome depended on its true value?	NA	
	**Risk of bias judgement**	**Low**	
**Bias in measurement of the outcome**	4.1 Was the method of measuring the outcome inappropriate?	PN	"The primary source for fall-related outcome ascertainment was structured telephone interviews of participants (or their proxies), conducted by masked, trained interviewers at the Yale Recruitment and Assessment Center, beginning at 4 months post-enrollment and continuing every 4 months thereafter. Participants were mailed fall calendars to prospectively record their falls and to serve as a memory aid for follow-up interviews. If participants did not complete a particular follow-up interview, the next followup interview asked about falls over the time window since the participant was last interviewed" (journal article.)
	4.2 Could measurement or ascertainment of the outcome have differed between intervention groups?	N	Follow-up interviews were conducted by masked interviewers.
	4.3a If N/PN/NI to 4.1 and 4.2: Were outcome assessors aware that a trial was taking place?	PY	
	4.3b If Y/PY/NI to 4.3a: Were outcome assessors aware of the intervention received by study participants?	N	
	4.4 If Y/PY/NI to 4.3b: Could assessment of the outcome have been influenced by knowledge of intervention received?	NA	

	4.5 If Y/PY/NI to 4.4: Is it likely that assessment of the outcome was influenced by knowledge of intervention received?	NA	
	**Risk of bias judgement**	**Low**	
**Bias in selection of the reported result**	5.1 Were the data that produced this result analysed in accordance with a pre-specified analysis plan that was finalized before unblinded outcome data were available for analysis?	PY	The date the analysis plan for this outcome was finalized: 2.10.2020<br>The date unblinded outcome data were available for analysis: ?<br>Authors have provided a reason for the changes made to the SAP.<br><br>There were a few modifications to the SAP for the outcome of all falls:<br><br>On 8-1-2020: The dates of fall events were not recorded, and the exact counts of the number of events were not recorded. Thus, a proper analysis of time to first fall and recurrent falls could not be done.<br><br>On 2-10-2020: The dates of the falls were not recorded. Thus, the analytic plan for the first fall was modified from a time-to-event analysis to a practice-level Poisson model using time from baseline to the midpoint of the interview window in which the first fall was recorded as an offset in the model. Cumulative incidence rates were replaced by event rates per person-year of follow-up.
	5.2 ... multiple eligible outcome measurements (e.g. scales, definitions, time points) within the outcome domain?	PN	The reported result seems to have been reported in accordance with the statistical analysis plan except for the changes that were specified.
	5.3 ... multiple eligible analyses of the data?	PN	
	**Risk of bias judgement**		
**Overall bias**	**Risk of bias judgement**	**Some concerns**	

**Unique ID**	Healey(2004)_A	**Study ID**		**Assessor**	RS
**Ref or Label**		**Aim**	assignment to intervention (the 'intention-to-treat' effect)		
**Experimental**	Targeted risk factor factor reduction core care plan	**Comparator**	No change in practice or environment relevant to fall prevention	**Source**	Journal article(s)
**Outcome**	Risk of falling in intervention group compared with control group as a change from the six-month period before intervention implementation to the six-month period after intervention implementation	**Results**	Relative risk 0.71 (95% CI 0.55, 0.90; p = 0.006)	**Weight**	

Domain	Signalling question	Response	Comments
**Bias arising from the randomization process**	1a.1 Was the allocation sequence random?	Y	"The study wards were divided into matched pairs. In each pair, one ward was randomly allocated to control or intervention by lottery, witnessed by six health professionals. Group randomisation was used, as it would be unreasonable to expect staff to refrain from applying their knowledge of the intervention to individual patients within a particular ward" (cited in journal article).
	1a.2 Was the allocation sequence concealed until clusters were enrolled and assigned to interventions?	NI	
	1a.3 Did baseline differences between intervention groups suggest a problem with the randomization process?	PN	"In Table 2 the control and intervention wards are compared in terms of a number of key variables. It can be seen that intervention wards had a somewhat lower 'turnover' of patients, with fewer new admissions and a slightly longer mean length of stay. Where primary diagnoses could be grouped the groups were broadly similar in percentage terms. Mean age and gender ratios were similar across both groups." (journal article)
	**Risk of bias judgement**	**Some concerns**	
**Bias arising from the timing of identification or recruitment of participants**	1b.1 Were all the individual participants identified and recruited (if appropriate) before randomization of clusters?	**N**	It was not possible to recruite participants before randomisation of clusters. The study was conducted in hospital wards and enrollment must occur after practices have been randomised.
	1b.2 If N/PN/NI to 1b.1: Is it likely that selection of individual participants was affected by knowledge of the intervention assigned to the cluster?		
	1b.3 Were there baseline imbalances that suggest differential identification or recruitment of individual participants between intervention groups?	**PN**	See 1a.3
	**Risk of bias judgement**	**Low**	Low
	2.1a Were participants aware that they were in a trial?	PN	Unable to find information.

Bias due to deviations from intended interventions	2.1b If Y/PY/NI to 2.1a: Were participants aware of their assigned intervention during the trial?	NA	For experimental intervention: It was impossible to keep nursing staff who delivered the intervention unaware that they were delivering the intervention.<br><br>For control intervention: "Managers on control wards were made aware of the study, and the need not to introduce the care plan in their area. Control wards made no other changes to practice or environment relevant to falls prevention during the study" (journal article)
	2.2 Were carers and people delivering the interventions aware of participants' assigned intervention during the trial?	Y	
	2.3 If Y/PY/NI to 2.1b or 2.2: Were there deviations from the intended intervention that arose because of the trial context?	NI	
	2.4 If Y/PY to 2.3: Were these deviations likely to have affected the outcome?	NA	
	2.5 If Y/PY/NI to 2.4: Were these deviations from intended intervention balanced between groups?	NA	
	2.6 Was an appropriate analysis used to estimate the effect of assignment to intervention?	NI	
	2.7 If N/PN/NI to 2.6: Was there potential for a substantial impact (on the result) of the failure to analyse participants in the group to which they were randomized ?	PN	It is likely that data were available from all participants as data of falls were obtained from the hospital's health and safety department which routinely collates information from the accident and incident reporting system forms required to be used on all wards.
	**Risk of bias judgement**	**Some concerns**	
Bias due to missing outcome data	3.1a Were data for this outcome available for all clusters that recruited participants?	NI	It is likely that data were available from all participants as data of falls were obtained from the hospital's health and safety department which routinely collates information from the accident and incident reporting system forms required to be used on all wards.
	3.1b Were data for this outcome available for all, or nearly all, participants within clusters?	NI	See 3.1.a
	3.2 If N/PN/NI to 3.1a or 3.1b: Is there evidence that the result was not biased by missing data?	PN	
	3.3 If N/PN to 3.2 Could missingness in the outcome depend on its true value?	N	
	3.4 If Y/PY/NI to 3.3: Is it likely that missingness in the outcome depended on its true value?	NA	
	**Risk of bias judgement**	**Low**	
Bias in measurement of the outcome	4.1 Was the method of measuring the outcome inappropriate?	PN	"The falls and injury data for the study were obtained from the hospital's Health and Safety Department (H&S) which routinely collates information from the Accident and Incident Reporting System (AIRS) forms required to be used on all wards. Data collection was effectively 'blind' since H&S staff did not know which wards were control or intervention." (journal article)
	4.2 Could measurement or ascertainment of the outcome have differed between intervention groups?	PN	See 4.1
	4.3a If N/PN/NI to 4.1 and 4.2: Were outcome assessors aware that a trial was taking place?	NI	I am unable to find information about whether the participants were aware that they were in a trial and, if so, whether they knew which intervention they received.
	4.3b If Y/PY/NI to 4.3a: Were outcome assessors aware of the intervention received by study participants?	NI	See 4.3a
	4.4 If Y/PY/NI to 4.3b: Could assessment of the outcome have been influenced by knowledge of intervention received?	PN	Falls is an outcome that requires little to no judgement.
	4.5 If Y/PY/NI to 4.4: Is it likely that assessment of the outcome was influenced by knowledge of intervention received?	NA	
	**Risk of bias judgement**	**Low**	
Bias in selection of the reported result	5.1 Were the data that produced this result analysed in accordance with a pre-specified analysis plan that was finalized before unblinded outcome data were available for analysis?	NI	Unable to find information about this.
	5.2 ... multiple eligible outcome measurements (e.g. scales, definitions, time points) within the outcome domain?	NI	Unable to find information about this.
	5.3 ... multiple eligible analyses of the data?	NI	Unable to find information about this.
	**Risk of bias judgement**		
**Overall bias**	**Risk of bias judgement**	**High**	Three individual domains at some concerns for RoB.

**Unique ID**	Logan (2021)_A	**Study ID**	4872	**Assessor**	RS
**Ref or Label**		**Aim**	assignment to intervention (the 'intention-to-treat' effect)		

**Experimental**	The Guide to Action Care Homes (GtACH) Falls Prevention Programme.	**Comparator**	Usual care	**Source**	Journal article(s); Trial protocol; Non-commercial trial registry record (e.g. ClinicalTrials.gov record)
**Outcome**	Fall rate per 1000 resident-days between 91 and 180 days after randomisation in intervention group compared with control group	**Results**	Rate ratio 0.57 (95% CI 0.45, 0.71; p<0.001)	**Weight**	1

Domain	Signalling question	Response	Comments
**Bias arising from the randomization process**	1a.1 Was the allocation sequence random?	Y	"Site trial coordinators randomised care homes on a 1:1 basis to one of two parallel arms (the GtACH programme or usual care) using a bespoke computer generated pseudo random code of variable block randomisation within strata (site, care home type (nursing, residential, dual registration)) provided by the Norwich Clinical Trials Unit through a secure web based randomisation service" (cited in journal article).<br><br>"Randomisation will be based on a bespoke computer generated pseudo-random code using variable block randomisation within strata (site, care home type [nursing/residential/dual registration]) provided by the Norwich Clinical Trials Unit (NCTU) via a secure web-based randomisation service" (cited in trial protocol).
	1a.2 Was the allocation sequence concealed until clusters were enrolled and assigned to interventions?	Y	
	1a.3 Did baseline differences between intervention groups suggest a problem with the randomization process?	PN	Table 1 in the journal article shows that there were small baseline differences between intervention and control.
	**Risk of bias judgement**	**Low**	
**Bias arising from the timing of identification or recruitment of participants**	1b.1 Were all the individual participants identified and recruited (if appropriate) before randomization of clusters?	**NI**	Unable to find information about this.
	1b.2 If N/PN/NI to 1b.1: Is it likely that selection of individual participants was affected by knowledge of the intervention assigned to the cluster?	**The researchers, resident participants, and staff informants were blind to allocation at consent and to baseline data collection" (cited in journal article).**	
	1b.3 Were there baseline imbalances that suggest differential identification or recruitment of individual participants between intervention groups?	**PN**	See 1a.3.
	**Risk of bias judgement**	**Low**	Low
**Bias due to deviations from intended interventions**	2.1a Were participants aware that they were in a trial?	Y	"By the nature of the intervention, care home staff and resident participants could not be blind to allocation group." (journal article)
	2.1b If Y/PY/NI to 2.1a: Were participants aware of their assigned intervention during the trial?	Y	"By the nature of the intervention, care home staff and resident participants could not be blind to allocation group." (journal article)
	2.2 Were carers and people delivering the interventions aware of participants' assigned intervention during the trial?	Y	
	2.3 If Y/PY/NI to 2.1b or 2.2: Were there deviations from the intended intervention that arose because of the trial context?	PN	"Measures taken to guard against contamination between groups12 comprised: explaining the importance of usual care for the control group, training staff in trial design and confdentiality agreements, collating data on staff moving to other homes in the study, and not publishing or sharing the training manual publicly. To aid recruitment, retention, and adherence to the protocol, all control homes were offered the intervention after the 12 month data had been collected and checked" (journal article).
	2.4 If Y/PY to 2.3: Were these deviations likely to have affected the outcome?	NA	
	2.5 If Y/PY/NI to 2.4: Were these deviations from intended intervention balanced between groups?	NA	
	2.6 Was an appropriate analysis used to estimate the effect of assignment to intervention?	PY	Dette spørsmålet skal jeg egentlig ikke svare på da jeg har svart PN til spørsmål<br><br>"Analyses were undertaken on an intention-to-treat basis according to a prespecifed statistical analysis plan (available from author)" (journal article). Data for this analysis were available for 630 out of 775 (81%) participants randomised to the intervention group and for 712 out of 882 (81%) participants randomised to the control group.
	2.7 If N/PN/NI to 2.6: Was there potential for a substantial impact (on the result) of the failure to analyse participants in the group to which they were randomized ?	NA	
	**Risk of bias judgement**	**Low**	
**Bias due to**	3.1a Were data for this outcome available for all clusters that recruited participants?	NI	Unable to find information.
	3.1b Were data for this outcome available for all, or nearly all, participants within clusters?	PN	Data for this analysis were available for 630 out of 775 (81%) participants randomised to the intervention group and for 712 out of 882 (81%) participants randomised to the control group. (journal article).

**missing outcome data**	3.2 If N/PN/NI to 3.1a or 3.1b: Is there evidence that the result was not biased by missing data?	PY	There were small differences between groups in the proportion of missing data.
	3.3 If N/PN to 3.2 Could missingness in the outcome depend on its true value?	NA	
	3.4 If Y/PY/NI to 3.3: Is it likely that missingness in the outcome depended on its true value?	NA	
	**Risk of bias judgement**	**Low**	
**Bias in measurement of the outcome**	4.1 Was the method of measuring the outcome inappropriate?	PN	" Care home staff recorded falls in the resident's care plans and on incident forms, in keeping with usual standards of care. Every three months, researchers blinded to allocation read all the care plans and recorded the date, place, and impact of falls for all participants, including for those who had died. They cross checked the written care plans with other data held in the care home, such as incident forms, records of ambulance visits, and records of hospital admissions" (journal article)<br><br>"It is possible that the intervention could have influenced the reporting of falls differentially between intervention and control arms because of the nature of GtACH. Although it is difcult to be sure about the directionality of such a difference, the focus on reporting falls to trigger the GtACH intervention could well have increased falls reporting in intervention homes compared with control homes. Thus it is unlikely that such a difference would have contributed to an overestimation of treatment effect."
	4.2 Could measurement or ascertainment of the outcome have differed between intervention groups?	PN	See 4.1
	4.3a If N/PN/NI to 4.1 and 4.2: Were outcome assessors aware that a trial was taking place?	Y	"Researchers collecting data remained blind to allocation but documented if they became unblinded. By the nature of the intervention, care home staff and resident participants could not be blind to allocation group." (cited in journal article)
	4.3b If Y/PY/NI to 4.3a: Were outcome assessors aware of the intervention received by study participants?	Y	See previous answer.
	4.4 If Y/PY/NI to 4.3b: Could assessment of the outcome have been influenced by knowledge of intervention received?	PN	Even though outcome assessors were aware that a trial was taking place and of the intervention received by participants, falls requires little judgement.
	4.5 If Y/PY/NI to 4.4: Is it likely that assessment of the outcome was influenced by knowledge of intervention received?	NA	
	**Risk of bias judgement**	**Low**	
**Bias in selection of the reported result**	5.1 Were the data that produced this result analysed in accordance with a pre-specified analysis plan that was finalized before unblinded outcome data were available for analysis?	Y	"Data will be analysed according to a pre-specified statistical analysis plan which will be finalised prior to the start of the analysis" (trial protocol).<br><br>"The only deviation from the initial protocol was that to better standardise reporting we relabelled the intervals for falls from months (0-3, 6-9, and 9-12) to days (1-90, 181-270, and 271-360)" (journal article).<br><br>"Treatment allocations were concealed from the study statistician until the main analyses were complete." (cited in journal article).
	5.2 ... multiple eligible outcome measurements (e.g. scales, definitions, time points) within the outcome domain?	PN	"The primary outcome, rate of falling over the three month period prior to six months post-randomisation, will be expressed as the number of falls per 1,000 resident days for each group. This period was chosen to give time for the intervention to be implemented after training, while acknowledging that people in care homes have short life expectancies. The number of falls per resident will be compared between groups using a two-level Poisson or negative binomial model with resident at level one and care home at level two, with length of residence in care home as an offset. The choice of model to be used will be dependent on the dispersion of the data. The primary analysis will adjust for type of care home (residential, nursing, dual registration) and site" (trial protocol. The description of the analyses in the journal article corresponds to the plan detailed in the trial protocol.
	5.3 ... multiple eligible analyses of the data?	PN	
	**Risk of bias judgement**		
**Overall bias**	**Risk of bias judgement**	**Low**	

**Unique ID**	Phelan (2024)_A	**Study ID**	6686	**Assessor**	RS

**Ref or Label**		**Aim**	assignment to intervention (the 'intention-to-treat' effect)		
**Experimental**	STOP-FALLS	**Comparator**	Usual care	**Source**	Journal article(s); Trial protocol; Statistical analysis plan (SAP)
**Outcome**	Time to first medically treated fall in intervention group compared with control group, at 18 months follow-up	**Results**	Hazard ratio 1.11 (95% CI 0.94, 1.31; p=0.11)	**Weight**	

Domain	Signalling question	Response	Comments
**Bias arising from the randomization process**	1a.1 Was the allocation sequence random?	Y	"Randomization was computer generated and constrained randomization was used." (journal article).<br><br>"The randomization occurred after all clinics had agreed to participate, all clinics were randomized at one time, and clinics were unaware of other clinics' randomization assignment; therefore, randomization was concealed." (Trial protocol).
	1a.2 Was the allocation sequence concealed until clusters were enrolled and assigned to interventions?	Y	
	1a.3 Did baseline differences between intervention groups suggest a problem with the randomization process?	N	There were similar baseline characteristics between participants in the intervention group and participants in the control group.
	**Risk of bias judgement**	**Low**	
**Bias arising from the timing of identification or recruitment of participants**	1b.1 Were all the individual participants identified and recruited (if appropriate) before randomization of clusters?	**N**	"Primary care clinics are randomized, and then potential participants identifed as having their PCP within the clinic. Identifcation of potential participants, and the start of enrollment and intervention delivery, occur on a rolling basis, with an intervention launch date scheduled for each intervention and control clinic pair. All potential participants meeting eligibility criteria for a given clinic pair are identifed on the launch date, starting the 12-month intervention period for that clinic pair." (Trial protocol).
	1b.2 If N/PN/NI to 1b.1: Is it likely that selection of individual participants was affected by knowledge of the intervention assigned to the cluster?	**"Eligible participants are identified by the study programmer using KPWA automated data." However, based on the provided information in the journal article and in the trial protocol we are unable to determine whether the study programmer had knowledge of the intervention assigned to the cluster when recruiting participants.**	
	1b.3 Were there baseline imbalances that suggest differential identification or recruitment of individual participants between intervention groups?	**N**	There were similar baseline characteristics between participants in the intervention group and participants in the control group, and the number of participants recruited to each group were roughly similar (n = 1 106 in the intervention group and n = 1 261 in the control group).
	**Risk of bias judgement**	**Some concerns**	Some concerns
	2.1a Were participants aware that they were in a trial?	Y	"Participants within the intervention clinic are not blinded, but usual care participants are blinded." (Trial protocol).
	2.1b If Y/PY/NI to 2.1a: Were participants aware of their assigned intervention during the trial?	Y	Nursing staff in intervention wards were aware of the wards' assigned intervention.<br><br>Intervention clinic directors, providers, and staff are not blinded to randomization assignment. (Trial protocol).
	2.2 Were carers and people delivering the interventions aware of participants' assigned intervention during the trial?	Y	

**Bias due to deviations from intended interventions**	2.3 If Y/PY/NI to 2.1b or 2.2: Were there deviations from the intended intervention that arose because of the trial context?	Y	"Several deprescribing initiatives implemented by the delivery system contemporaneous with the STOP-FALLS trial may account for the high deprescribing rates in the usual care arm. These initiatives included a chronic pain clinic, where people prescribed high-dose opioids could be referred for intervention, an EHR "stop" that prohibited coprescribing of opioids and benzodiazepines, and an updated KPWA guideline on sedative-hypnotic prescribing." (Journal article).<br><br>"The trial was conducted during the COVID-19 pandemic, during which outpatient visits were frequently conducted remotely; this factor, along with the additional demands on health care professionals posed by COVID-19, may have limited deprescribing considerations." (Journal article). While COVID-19 is not considered part of the study context, the pandemic positioned the study in a extraordinary context which likely affected the outcome.
	2.4 If Y/PY to 2.3: Were these deviations likely to have affected the outcome?	Y	
	2.5 If Y/PY/NI to 2.4: Were these deviations from intended intervention balanced between groups?	PY	Since the deviations from the intervention were introduced by the deprescribing initiatives implemented by the health care delivery system, COVID-19 pandemic, it is likely that intervention clinics and control clinics were affected similarly.
	2.6 Was an appropriate analysis used to estimate the effect of assignment to intervention?	Y	The analysis was, according to the journal article, conducted by the intention-to-treat principle, whereby all clusters and participants were analysed in the groups they were initially assigned. However, the analysis excluded participants with missing outcome data (censored participants), but this analysis is considered appropriate with regard to this question.<br><br>"Once a participant is determined as having received care or having a PCP at a given clinic, their clinic assignment is fxed, and therefore their randomization assignment will be static throughout the study." (Trial protocol).
	2.7 If N/PN/NI to 2.6: Was there potential for a substantial impact (on the result) of the failure to analyse participants in the group to which they were randomized ?	NA	
	**Risk of bias judgement**	**Some concerns**	
**Bias due to missing outcome data**	3.1a Were data for this outcome available for all clusters that recruited participants?	Y	We assume that if one or more clusters had no analysable participants, it would have been reported.
	3.1b Were data for this outcome available for all, or nearly all, participants within clusters?	N	Even though the analysis was described as ITT, the analysis excluded participants with missing outcome data (censored participants):<br><br>"There were 159 of 1106 participants in the intervention group (14%) and 206 of 1261 participants in the usual care group (16%) who were censored due to disenrollment from the health plan prior to having a medically treated fall. A total of 72 of 1106 participants (7%) in the intervention group and 86 of 1261 (7%) in the usual care group died. Health plan disenrollment and death were the only reasons for loss to follow-up." (Journal article).
	3.2 If N/PN/NI to 3.1a or 3.1b: Is there evidence that the result was not biased by missing data?	Y	The proportions of participants missing are similar in across intervention groups.
	3.3 If N/PN to 3.2 Could missingness in the outcome depend on its true value?	NA	
	3.4 If Y/PY/NI to 3.3: Is it likely that missingness in the outcome depended on its true value?	NA	
	**Risk of bias judgement**	**Low**	
	4.1 Was the method of measuring the outcome inappropriate?	N	"The primary outcome was time (in days) to the first medically treated fall, ascertained from electronic utilization files of the health plan, which capture all health care encounters for patients in KPWA's integrated group practice. International Statistical Classification of Diseases and Related Health<br>Problems, Tenth Revision (ICD-10), injury codes (S or T), musculoskeletal disease codes (M), or fall related cause of injury codes (W) were used to identify medically treated falls." (Journal article).<br><br>This is considered appropriate since the outcome was medically treated falls, and not all falls.

**Bias in measurement of the outcome**	4.2 Could measurement or ascertainment of the outcome have differed between intervention groups?	N	Primary care practitioners in the intervention clinics were not blinded to intervention status, however primary care pratcitioners in the control clinics were. It may be possible that primary care practitioners in the intervention group payed more attention to falls than primary care practitioners in the control group, increasing intervention PCPs' inclination to ask about and and potentially register falls. However, given that falls is an event that is reported by the participant, ascertainment of falls is assumed not to have differed between intervention groups.
	4.3a If N/PN/NI to 4.1 and 4.2: Were outcome assessors aware that a trial was taking place?	Y	Yes, individual participants in the intervention clinics were aware that a trial was taking place.
	4.3b If Y/PY/NI to 4.3a: Were outcome assessors aware of the intervention received by study participants?	Y	Yes, individual participants in the intervention clinics were aware of their assigned intervention, whereas individual participants in the control clinics were not.
	4.4 If Y/PY/NI to 4.3b: Could assessment of the outcome have been influenced by knowledge of intervention received?	N	Falls is a participant-reported outcome that is assumed not to be influenced by knowledge of the intervention received.
	4.5 If Y/PY/NI to 4.4: Is it likely that assessment of the outcome was influenced by knowledge of intervention received?	NA	
	**Risk of bias judgement**	**Low**	
**Bias in selection of the reported result**	5.1 Were the data that produced this result analysed in accordance with a pre-specified analysis plan that was finalized before unblinded outcome data were available for analysis?	Y	"The statistical analysis plan was finalized prior to submission and no outcome data analysis will be conducted until after publication of the study's protocol." (Trial protocol). The analysis was conducted according to the SAP.
	5.2 ... multiple eligible outcome measurements (e.g. scales, definitions, time points) within the outcome domain?	N	The data analysis plan (SAP) specified that the outcome was going to be reduction in number of medically treated falls between intervention and usual care groups over 12 to 24 months. While stating "12 to 24 months" is vague, the choice of 18 months follow-up seems to be in line with the plan. Also, the article reports the number of medically treated falls, which is in line with the SAP.
	5.3 ... multiple eligible analyses of the data?	N	
	**Risk of bias judgement**		
**Overall bias**	**Risk of bias judgement**	**Some concerns**	

**Unique ID**	Phelan (2024)_B	**Study ID**	6686	**Assessor**	RS
**Ref or Label**		**Aim**	assignment to intervention (the 'intention-to-treat' effect)		
**Experimental**	STOP-FALLS	**Comparator**	Usual care	**Source**	
**Outcome**	Discontinuation of medications (defined as no prescription fill for 90 days), summarized across all target medication classes, referred to as "first target medication", at six months follow-up in intervention group compared with control group.	**Results**	Adjusted relative risk 1.24 (95% CI 0.90, 1.70)	**Weight**	

Domain	Signalling question	Response	Comments
**Bias arising from the randomization process**	1a.1 Was the allocation sequence random?	Y	"Randomization was computer generated and constrained randomization was used." (journal article).<br><br>"The randomization occurred after all clinics had agreed to participate, all clinics were randomized at one time, and clinics were unaware of other clinics' randomization assignment; therefore, randomization was concealed." (Trial protocol).
	1a.2 Was the allocation sequence concealed until clusters were enrolled and assigned to interventions?	Y	
	1a.3 Did baseline differences between intervention groups suggest a problem with the randomization process?	N	There were similar baseline characteristics between participants in the intervention group and participants in the control group.
	**Risk of bias judgement**	**Low**	
	1b.1 Were all the individual participants identified and recruited (if appropriate) before randomization of clusters?	**N**	"Primary care clinics are randomized, and then potential participants identifed as having their PCP within the clinic. Identifcation of potential participants, and the start of enrollment and intervention delivery, occur on a rolling basis, with an intervention launch date scheduled for each intervention and control clinic pair. All potential participants meeting eligibility criteria for a given clinic pair are identifed on the launch date, starting the 12-month intervention period for that clinic pair." (Trial protocol).

**Bias arising from the timing of identification or recruitment of participants**	1b.2 If N/PN/NI to 1b.1: Is it likely that selection of individual participants was affected by knowledge of the intervention assigned to the cluster?	**"Eligible participants are identified by the study programmer using KPWA automated data." However, based on the provided information in the journal article and in the trial protocol we are unable to determine whether the study programmer had knowledge of the intervention assigned to the cluster when recruiting participants.**	
	1b.3 Were there baseline imbalances that suggest differential identification or recruitment of individual participants between intervention groups?	**N**	There were similar baseline characteristics between participants in the intervention group and participants in the control group, and the number of participants recruited to each group were roughly similar (n = 1 106 in the intervention group and n = 1 261 in the control group).
	**Risk of bias judgement**	**Some concerns**	Some concerns
**Bias due to deviations from intended interventions**	2.1a Were participants aware that they were in a trial?	Y	"Participants within the intervention clinic are not blinded, but usual care participants are blinded." (Trial protocol).
	2.1b If Y/PY/NI to 2.1a: Were participants aware of their assigned intervention during the trial?	Y	Nursing staff in intervention wards were aware of the wards' assigned intervention.<br><br>Intervention clinic directors, providers, and staff are not blinded to randomization assignment. (Trial protocol).
	2.2 Were carers and people delivering the interventions aware of participants' assigned intervention during the trial?	Y	
	2.3 If Y/PY/NI to 2.1b or 2.2: Were there deviations from the intended intervention that arose because of the trial context?	Y	"Several deprescribing initiatives implemented by the delivery system contemporaneous with the STOP-FALLS trial may account for the high deprescribing rates in the usual care arm. These initiatives included a chronic pain clinic, where people prescribed high-dose opioids could be referred for intervention, an EHR "stop" that prohibited coprescribing of opioids and benzodiazepines, and an updated KPWA guideline on sedative-hypnotic prescribing." (Journal article).<br><br>"The trial was conducted during the COVID-19 pandemic, during which outpatient visits were frequently conducted remotely; this factor, along with the additional demands on health care professionals posed by COVID-19, may have limited deprescribing considerations." (Journal article). While COVID-19 is not considered part of the study context, the pandemic positioned the study in a extraordinary context which likely affected the outcome.
	2.4 If Y/PY to 2.3: Were these deviations likely to have affected the outcome?	Y	
	2.5 If Y/PY/NI to 2.4: Were these deviations from intended intervention balanced between groups?	PY	Since the deviations from the intervention were introduced by the deprescribing initiatives implemented by the health care delivery system, COVID-19 pandemic, it is likely that intervention clinics and control clinics were affected similarly.
	2.6 Was an appropriate analysis used to estimate the effect of assignment to intervention?	Y	The analysis was, according to the journal article, conducted by the intention-to-treat principle, whereby all clusters and participants were analysed in the groups they were initially assigned. However, the analysis excluded participants with missing outcome data (censored participants), but this analysis is considered appropriate with regard to this question.<br><br>"Once a participant is determined as having received care or having a PCP at a given clinic, their clinic assignment is fxed, and therefore their randomization assignment will be static throughout the study." (Trial protocol).
	2.7 If N/PN/NI to 2.6: Was there potential for a substantial impact (on the result) of the failure to analyse participants in the group to which they were randomized ?	NA	
	**Risk of bias judgement**	**Some concerns**	
	3.1a Were data for this outcome available for all clusters that recruited participants?	Y	We assume that if one or more clusters had no analysable participants, it would have been reported.

**Bias due to missing outcome data**	3.1b Were data for this outcome available for all, or nearly all, participants within clusters?	N	Even though the analysis was described as ITT, the analysis excluded participants with missing outcome data (censored participants):<br><br>"There were 159 of 1106 participants in the intervention group (14%) and 206 of 1261 participants in the usual care group (16%) who were censored due to disenrollment from the health plan prior to having a medically treated fall. A total of 72 of 1106 participants (7%) in the intervention group and 86 of 1261 (7%) in the usual care group died. Health plan disenrollment and death were the only reasons for loss to follow-up." (Journal article).
	3.2 If N/PN/NI to 3.1a or 3.1b: Is there evidence that the result was not biased by missing data?	Y	The proportions of participants missing are similar in across intervention groups.
	3.3 If N/PN to 3.2 Could missingness in the outcome depend on its true value?	NA	
	3.4 If Y/PY/NI to 3.3: Is it likely that missingness in the outcome depended on its true value?	NA	
	**Risk of bias judgement**	**Low**	
**Bias in measurement of the outcome**	4.1 Was the method of measuring the outcome inappropriate?	N	"Drug name, dose, frequency, route, and days' supply were obtained from automated [illegible]
	4.2 Could measurement or ascertainment of the outcome have differed between intervention groups?	N	While primary care practitioners who entered data into the electronic health records and study programmers who extracted data from the electronic health records were not blinded, medication data are objective and therefore not based on judgement. We therefore assume that the same methods for collecting and extracting data were used across intervention groups.
	4.3a If N/PN/NI to 4.1 and 4.2: Were outcome assessors aware that a trial was taking place?	Y	Yes, primary care practitioners in the intervention clinics were aware that a trial was taking place.
	4.3b If Y/PY/NI to 4.3a: Were outcome assessors aware of the intervention received by study participants?	Y	Yes, primary care practitioners in the intervention clinics were aware of intervention status of their clinic.
	4.4 If Y/PY/NI to 4.3b: Could assessment of the outcome have been influenced by knowledge of intervention received?	N	Even though primary care practitioners in the intervention group were not blinded to intervention status, we consider it unlikely that primary care practitioners entered medication data into the electroinc health records differently than they would have done if they were blinded to intervention status. Also, while the study programmers who extracted data from the electronic health records were not blinded, we consider it unlikely that data extraction was affected by knowledge of intervention status.
	4.5 If Y/PY/NI to 4.4: Is it likely that assessment of the outcome was influenced by knowledge of intervention received?	NA	
	**Risk of bias judgement**	**Low**	
**Bias in selection of the reported result**	5.1 Were the data that produced this result analysed in accordance with a pre-specified analysis plan that was finalized before unblinded outcome data were available for analysis?	Y	In the trial protocol, it says that the statistical analysis plan was finalised prior to submission and no outcome data analysis will be conducted until after publication of the study's protocol. This indicates that the analysis plan was finished before outcome data were available. It's also statet in the trial protocol that the biostatisticians are not blinded but will not have access to follow-up outcome data until after final analytic datasets are complete, and the statistical plan will be finalised before receiving any follow-up data.
	5.2 ... multiple eligible outcome measurements (e.g. scales, definitions, time points) within the outcome domain?	NI	While the information provided in the answer to signalling question 5.1 indicates that the result likely was not selected, on the basis of the result, from multiple eligible outcome measurements or from multiple eligible analyses of the data, we were unable to locate information about the plans for analysing medication data in the statistical analysis plan. Therefore, the details of the analysis plan were not reported in sufficient detail to make an assessment.
	5.3 ... multiple eligible analyses of the data?	NI	See the answer to signalling question 5.2.
	**Risk of bias judgement**		
**Overall bias**	**Risk of bias judgement**	**High**	

**Unique ID**	Tamblyn(2012)_A	**Study ID**	984	**Assessor**	RS
**Ref or Label**		**Aim**	assignment to intervention (the 'intention-to-treat' effect)		

**Experimental**	Information about patient-secific risk of injury based on non-modifiable risk factors and psychotropic drug doses presented on-screen in the form of graphics to physicians	**Comparator**	Commercial drug alerts presented to physicians	**Source**	Journal article(s); Non-commercial trial registry record (e.g. ClinicalTrials.gov record)
**Outcome**	Mean reduction in risk of injury per 1000 patients during 23 months follow-up in intervention group compared with control group	**Results**	Mean difference 1.7 (95% CI 0.2, 3.2; p = 0.02)	**Weight**	1

Domain	Signalling question	Response	Comments
**Bias arising from the randomization process**	1a.1 Was the allocation sequence random?	Y	Within each stratum, an equivalent number of physicians were randomized by the biostatistician to intervention and control groups using a random number table.
	1a.2 Was the allocation sequence concealed until clusters were enrolled and assigned to interventions?	Y	
	1a.3 Did baseline differences between intervention groups suggest a problem with the randomization process?	N	Observed differences in baseline characteristics between intervention groups are likely to be compatible with chance.
	**Risk of bias judgement**	**Low**	
**Bias arising from the timing of identification or recruitment of participants**	1b.1 Were all the individual participants identified and recruited (if appropriate) before randomization of clusters?	**PN**	Physicians were randomised to intervention or control, and individual participants were the physicians' patients during the study period. It is very likely that not all patients were identified and recruited before the physicians were randomised.
	1b.2 If N/PN/NI to 1b.1: Is it likely that selection of individual participants was affected by knowledge of the intervention assigned to the cluster?	**Given that the intervention consisted of computerised prescribing decision support with patient-specific risk estimates to the patients' physicians, is is not likely that selection of individual participants was affected by knowledge of the intervention assigned to the clusters.**	
	1b.3 Were there baseline imbalances that suggest differential identification or recruitment of individual participants between intervention groups?	**N**	Observed differences in baseline characteristics between intervention groups are likely to be compatible with chance.
	**Risk of bias judgement**	**Low**	Low
**Bias due to deviations from intended interventions**	2.1a Were participants aware that they were in a trial?	Y	
	2.1b If Y/PY/NI to 2.1a: Were participants aware of their assigned intervention during the trial?	Y	Physicians were not blinded to the intervention status, but were blinded to the specific study outcomes that were measured.
	2.2 Were carers and people delivering the interventions aware of participants' assigned intervention during the trial?	Y	
	2.3 If Y/PY/NI to 2.1b or 2.2: Were there deviations from the intended intervention that arose because of the trial context?	PN	
	2.4 If Y/PY to 2.3: Were these deviations likely to have affected the outcome?	NA	
	2.5 If Y/PY/NI to 2.4: Were these deviations from intended intervention balanced between groups?	NA	
	2.6 Was an appropriate analysis used to estimate the effect of assignment to intervention?	PY	Unless patients changed physician during follow-up, patients were analysed in the group to which they were assigned.
	2.7 If N/PN/NI to 2.6: Was there potential for a substantial impact (on the result) of the failure to analyse participants in the group to which they were randomized ?	NA	
	**Risk of bias judgement**	**Low**	
**Bias due to missing outcome data**	3.1a Were data for this outcome available for all clusters that recruited participants?	Y	
	3.1b Were data for this outcome available for all, or nearly all, participants within clusters?	Y	
	3.2 If N/PN/NI to 3.1a or 3.1b: Is there evidence that the result was not biased by missing data?	NA	
	3.3 If N/PN to 3.2 Could missingness in the outcome depend on its true value?	NA	
	3.4 If Y/PY/NI to 3.3: Is it likely that missingness in the outcome depended on its true value?	NA	
	**Risk of bias judgement**	**Low**	
**Bias in measurement of the outcome**	4.1 Was the method of measuring the outcome inappropriate?	N	Outcome was risk of injury atend of follow-up, which was submitted as the outcome to ClinicalTrials.gov in 2009. Authors provide a detailed explenation of how the risk of injury was calculated.
	4.2 Could measurement or ascertainment of the outcome have differed between intervention groups?	N	The same equation was used to calculate the risk of injury for both intervention groups.
	4.3a If N/PN/NI to 4.1 and 4.2: Were outcome assessors aware that a trial was taking place?	N	
	4.3b If Y/PY/NI to 4.3a: Were outcome assessors aware of the intervention received by study participants?	NA	
	4.4 If Y/PY/NI to 4.3b: Could assessment of the outcome have been influenced by knowledge of intervention received?	NA	
	4.5 If Y/PY/NI to 4.4: Is it likely that assessment of the outcome was influenced by knowledge of intervention received?	NA	

	**Risk of bias judgement**	**Low**	
**Bias in selection of the reported result**	5.1 Were the data that produced this result analysed in accordance with a pre-specified analysis plan that was finalized before unblinded outcome data were available for analysis?	PY	The authors published an analysis plan under Descriptive information / Detalied description at ClinicalTrials.gov before unblinded outcome data were available for analysis.
	5.2 ... multiple eligible outcome measurements (e.g. scales, definitions, time points) within the outcome domain?	PN	The journal article clearly specified the equation that was used to calculate patients' risk of injury, and the same equation was used to calculate risk of injury at both baseline and follow-up.
	5.3 ... multiple eligible analyses of the data?	PN	Same as above.
	**Risk of bias judgement**		
**Overall bias**	**Risk of bias judgement**	**Low**	

**Unique ID**	Snooks(2014)_A	**Study ID**	2484	**Assessor**	
**Ref or Label**		**Aim**	assignment to intervention (the 'intention-to-treat' effect)		
**Experimental**	Computerised Clinical Decision Support for paramedics attending older people who fall	**Comparator**	Usual care with paper-based protocols to assess patients and make decisions about their care instead of computerised clinical decision support	**Source**	Journal article(s); Trial protocol; Non-commercial trial registry record (e.g. ClinicalTrials.gov record)
**Outcome**	Odds of a patient being referred to a falls service during one month follow-up in intervention group compared with control group	**Results**	Odds Ratio 1.131 (95% CI 1.115, 3.717; p = 0.021)	**Weight**	1

**Domain**	**Signalling question**	**Response**	**Comments**
**Bias arising from the randomization process**	1a.1 Was the allocation sequence random?	PY	This is a cluster trial with paramedics as the unit of randomisation.<br><br>"The trial team consented volunteers and passed anonymous details to the West Wales Organisation for Rigorous Trials in Health (WWORTH) for randomisation stratified by current ambulance station" (cited in journal article).<br><br>"The West Wales Organisation for Rigorous Trials in Health (WWORTH) independently used random number tables to allocate paramedics, consented and stratified by current ambulance station, between intervention and control arms" (cited in journal article).
	1a.2 Was the allocation sequence concealed until clusters were enrolled and assigned to interventions?	PY	
	1a.3 Did baseline differences between intervention groups suggest a problem with the randomization process?	PN	Figure 2 in the journal article indicates that any baseline differences are compatible with chance.
	**Risk of bias judgement**	**Low**	
**Bias arising from the timing of identification or recruitment of participants**	1b.1 Were all the individual participants identified and recruited (if appropriate) before randomization of clusters?	**N**	Most or all individual participants were not identified and recruited before randomisation of clusters, as the individual participants were those attended by a study paramedic following their first emergency call.
	1b.2 If N/PN/NI to 1b.1: Is it likely that selection of individual participants was affected by knowledge of the intervention assigned to the cluster?	**Not likely because the older adults attended by paramedics were attended because a 999 emergency call was made due to a fall.**	
	1b.3 Were there baseline imbalances that suggest differential identification or recruitment of individual participants between intervention groups?	**PN**	Figure 2 in the journal article indicates that any baseline differences are compatible with chance.
	**Risk of bias judgement**	**Low**	Low
	2.1a Were participants aware that they were in a trial?	PN	
	2.1b If Y/PY/NI to 2.1a: Were participants aware of their assigned intervention during the trial?	NA	"As emergency callers are often in distress and in need of urgent aid, consent will not be attempted by phone or at the time when the patient is first attended. Patients will be identified from routinely available ambulance service information (control room and patient record forms) and will be contacted by post at their usual home and any temporary address (such as their hospital ward) 7 – 10 days after the index fall to inform them about the study" (cited in protocol).
	2.2 Were carers and people delivering the interventions aware of participants' assigned intervention during the trial?	Y	

**Bias due to deviations from intended interventions**	2.3 If Y/PY/NI to 2.1b or 2.2: Were there deviations from the intended intervention that arose because of the trial context?	NI	Under 'Deviations from protocol' in the journal article is says that the paramedic training was reduced in consultation with participating ambulance services from two days to half a day including assessment of competence. This is a change that arose because of the trial context, but we do not know whether the reduced training lead to deviations from intended intervention.
	2.4 If Y/PY to 2.3: Were these deviations likely to have affected the outcome?	NA	
	2.5 If Y/PY/NI to 2.4: Were these deviations from intended intervention balanced between groups?	NA	
	2.6 Was an appropriate analysis used to estimate the effect of assignment to intervention?	PY	"Measures of process, outcome and cost will be compared between intervention and control groups patients according to 'intention to treat'" (cited in Protocol S1).<br><br>"One intervention paramedic received training but no equipment; he remained in the intervention group for analysis by treatment allocated" (cited in journal article).
	2.7 If N/PN/NI to 2.6: Was there potential for a substantial impact (on the result) of the failure to analyse participants in the group to which they were randomized ?	NA	
	**Risk of bias judgement**	**Some concerns**	
**Bias due to missing outcome data**	3.1a Were data for this outcome available for all clusters that recruited participants?	N	17 out of 22 paramedics randomised to the experimental intervention were analysed and 18 out of 20 paramedics randomised to the control intervention were analysed.
	3.1b Were data for this outcome available for all, or nearly all, participants within clusters?	PN	200 patients in the experimental intervention and 144 patients in the control intervention group were lost to follow-up. The proportion of participants lost and the reasons for loss to follow-up were largely similar across groups.
	3.2 If N/PN/NI to 3.1a or 3.1b: Is there evidence that the result was not biased by missing data?	PY	Figure 1 in the journal article shows the reasons participants were lost to follow-up. The reasons for and the numbers of participants being lost to follow-up seem to be fairly similar across the two intervention groups.
	3.3 If N/PN to 3.2 Could missingness in the outcome depend on its true value?	NA	
	3.4 If Y/PY/NI to 3.3: Is it likely that missingness in the outcome depended on its true value?	NA	
	**Risk of bias judgement**	**Low**	
**Bias in measurement of the outcome**	4.1 Was the method of measuring the outcome inappropriate?	PN	"Operational indicators - ambulance service job cycle time, length of episode of emergency care and costs of care - were gathered from routine NHS sources" (cited in journal article).
	4.2 Could measurement or ascertainment of the outcome have differed between intervention groups?	PN	As routinely collected ambulance records were used ascertainment of this outcome did probably not differ between intervention groups.
	4.3a If N/PN/NI to 4.1 and 4.2: Were outcome assessors aware that a trial was taking place?	NI	Unable to find information about this.
	4.3b If Y/PY/NI to 4.3a: Were outcome assessors aware of the intervention received by study participants?	NI	Unable to find information about this.
	4.4 If Y/PY/NI to 4.3b: Could assessment of the outcome have been influenced by knowledge of intervention received?	PN	Because the outcome of whether a patient was left at scene without conveyance to an ED was collected from routine electronic record data, this outcome is not considered to be vulnerable to knowledge of intervention received.
	4.5 If Y/PY/NI to 4.4: Is it likely that assessment of the outcome was influenced by knowledge of intervention received?	NA	
	**Risk of bias judgement**	**Low**	
**Bias in selection of the reported result**	5.1 Were the data that produced this result analysed in accordance with a pre-specified analysis plan that was finalized before unblinded outcome data were available for analysis?	PN	It is stated in the trial protocol that intention to treat principles will be used to compare process, outcome and cost measures between intervention and control group. More specifically, it is stated that individuals will remain in the group to which they are first allocated. However, I was unable to find congruence between the reported result and an a priori analysis plan, i.e. a statement made prior to unblinded outcome data being available to investigators that their plan was to analyse and report the specific outcome domain, measurement and analysis that was reported.
	5.2 ... multiple eligible outcome measurements (e.g. scales, definitions, time points) within the outcome domain?	NI	Unable to find information about this.
	5.3 ... multiple eligible analyses of the data?	NI	Unable to find information about this.
	**Risk of bias judgement**		
**Overall bias**	**Risk of bias judgement**	**Some concerns**	An overall RoB judgement of some concerns was given because the reason for judging the two domains at some concerns was lack of information and not the presence of evidence of bias.

Unique ID	Snooks(2014)_B	Study ID	2484	Assessor	RS
Ref or Label		Aim	assignment to intervention (the 'intention-to-treat' effect)		
Experimental	Computerised Clinical Decision Support for paramedics attending older people who fall	Comparator	Usual care with paper-based protocols to assess patients and make decisions about their care instead of computerised clinical decision support	Source	Journal article(s); Trial protocol; Non-commercial trial registry record (e.g. ClinicalTrials.gov record)
Outcome	Odds of a patient reporting ≥1 falls (self-report) during one month follow-up in intervention group compared with control group	Results	Odds Ratio 0.752 (95% CI 0.503, 1.124; p = 0.165)	Weight	1

Domain	Signalling question	Response	Comments
**Bias arising from the randomization process**	1a.1 Was the allocation sequence random?	PY	This is a cluster trial with paramedics as the unit of randomisation.<br><br>"The trial team consented volunteers and passed anonymous details to the West Wales Organisation for Rigorous Trials in Health (WWORTH) for randomisation stratified by current ambulance station" (cited in journal article).
	1a.2 Was the allocation sequence concealed until clusters were enrolled and assigned to interventions?	PY	
	1a.3 Did baseline differences between intervention groups suggest a problem with the randomization process?	PN	Figure 2 in the journal article indicates that any baseline differences are compatible with chance.
	**Risk of bias judgement**	**Low**	
**Bias arising from the timing of identification or recruitment of participants**	1b.1 Were all the individual participants identified and recruited (if appropriate) before randomization of clusters?	**N**	Most or all individual participants were not identified and recruited before randomisation of clusters, as the individual participants were those attended by a study paramedic following their first emergency call.
	1b.2 If N/PN/NI to 1b.1: Is it likely that selection of individual participants was affected by knowledge of the intervention assigned to the cluster?	**Not likely because the older adults attended by paramedics were attended because a 999 emergency call was made due to a fall.**	
	1b.3 Were there baseline imbalances that suggest differential identification or recruitment of individual participants between intervention groups?	**PN**	Figure 2 in the journal article indicates that any baseline differences are compatible with chance.
	**Risk of bias judgement**	**Low**	Low
**Bias due to deviations from intended interventions**	2.1a Were participants aware that they were in a trial?	PN	
	2.1b If Y/PY/NI to 2.1a: Were participants aware of their assigned intervention during the trial?	NA	"As emergency callers are often in distress and in need of urgent aid, consent will not be attempted by phone or at the time when the patient is first attended. Patients will be identified from routinely available ambulance service information (control room and patient record forms) and will be contacted by post at their usual home and any temporary address (such as their hospital ward) 7 – 10 days after the index fall to inform them about the study" (cited in protocol).
	2.2 Were carers and people delivering the interventions aware of participants' assigned intervention during the trial?	Y	
	2.3 If Y/PY/NI to 2.1b or 2.2: Were there deviations from the intended intervention that arose because of the trial context?	NI	Under 'Deviations from protocol' in the journal article is says that the paramedic training was reduced in consultation with participating ambulance services from two days to half a day including assessment of competence. This is a change that arose because of the trial context, but we do not know whether the reduced training lead to deviations from intended intervention.
	2.4 If Y/PY to 2.3: Were these deviations likely to have affected the outcome?	NA	
	2.5 If Y/PY/NI to 2.4: Were these deviations from intended intervention balanced between groups?	NA	
	2.6 Was an appropriate analysis used to estimate the effect of assignment to intervention?	PY	"Measures of process, outcome and cost will be compared between intervention and control groups patients according to 'intention to treat'" (cited in Protocol S1).<br><br>"One intervention paramedic received training but no equipment; he remained in the intervention group for analysis by treatment allocated" (cited in journal article).
	2.7 If N/PN/NI to 2.6: Was there potential for a substantial impact (on the result) of the failure to analyse participants in the group to which they were randomized ?	NA	
	**Risk of bias judgement**	**Some concerns**	
	3.1a Were data for this outcome available for all clusters that recruited participants?	N	17 out of 22 paramedics randomised to the experimental intervention were analysed and 18 out of 20 paramedics randomised to the control intervention were analysed.

Bias due to missing outcome data	3.1b Were data for this outcome available for all, or nearly all, participants within clusters?	PN	200 patients in the experimental intervention and 144 patients in the control intervention group were lost to follow-up. The proportion of participants lost and the reasons for loss to follow-up were largely similar across groups.
	3.2 If N/PN/NI to 3.1a or 3.1b: Is there evidence that the result was not biased by missing data?	PY	Figure 1 in the journal article shows the reasons participants were lost to follow-up. The reasons for and the numbers of participants being lost to follow-up seem to be fairly similar across the two intervention groups.
	3.3 If N/PN to 3.2 Could missingness in the outcome depend on its true value?	NA	
	3.4 If Y/PY/NI to 3.3: Is it likely that missingness in the outcome depended on its true value?	NA	
	**Risk of bias judgement**	**Low**	
Bias in measurement of the outcome	4.1 Was the method of measuring the outcome inappropriate?	PN	"Quality of life of patients and carers, self-reported falls and independence will be followed up by questionnaire, to be administered by post (or interview, where necessary)" (cited in protocol).
	4.2 Could measurement or ascertainment of the outcome have differed between intervention groups?	PN	
	4.3a If N/PN/NI to 4.1 and 4.2: Were outcome assessors aware that a trial was taking place?	Y	It was not possible to blind patients.
	4.3b If Y/PY/NI to 4.3a: Were outcome assessors aware of the intervention received by study participants?	Y	It was not possible to blind patients.
	4.4 If Y/PY/NI to 4.3b: Could assessment of the outcome have been influenced by knowledge of intervention received?	PN	Even though falls are self-reported, they do not require much judgement on behalf of the patients.
	4.5 If Y/PY/NI to 4.4: Is it likely that assessment of the outcome was influenced by knowledge of intervention received?	NA	
	**Risk of bias judgement**	**Low**	
Bias in selection of the reported result	5.1 Were the data that produced this result analysed in accordance with a pre-specified analysis plan that was finalized before unblinded outcome data were available for analysis?	PN	It is stated in the trial protocol that intention to treat principles will be used to compare process, outcome and cost measures between intervention and control group. More specifically, it is stated that individuals will remain in the group to which they are first allocated. However, I was unable to find congruence between the reported result and an a priori analysis plan, i.e. a statement made prior to unblinded outcome data being available to investigators that their plan was to analyse and report the specific outcome domain, measurement and analysis that was reported.
	5.2 ... multiple eligible outcome measurements (e.g. scales, definitions, time points) within the outcome domain?	NI	Unable to find information about this.
	5.3 ... multiple eligible analyses of the data?	NI	Unable to find information about this.
	**Risk of bias judgement**		
**Overall bias**	**Risk of bias judgement**	**Some concerns**	An overall RoB judgement of some concerns was given because the reason for judging the two domains at some concerns was lack of information and not the presence of evidence of bias.

**Unique ID**	Weber, White & McIlvried(2008)_A	**Study ID**	1052	**Assessor**	RS
**Ref or Label**		**Aim**	assignment to intervention (the 'intention-to-treat' effect)		
**Experimental**	Standardized medication review with recommendations to primary care physician via the electronic medical record	**Comparator**	Usual care without medication review and recommendations sent to primary care physician	**Source**	Journal article(s)
**Outcome**	Odds of having had one or more fall-related medical encounters (as determined by EpicCare data only) during 15 months follow-up in intervention group compared with control group	**Results**	Odds Ratio 0.38 (p < 0.01)	**Weight**	1

**Domain**	**Signalling question**	**Response**	**Comments**
Bias arising from the randomization process	1a.1 Was the allocation sequence random?	NI	It says in the journal article that the clinic sites were randomized, but not how the allocation sequence was generated.<br><br>"We then randomized clinic sites to receive either the intervention or usual care. Fifteen clinic sites received the electronic intervention, whereas 3 clinic sites served as controls. Randomization occurred according to clinic site, not physician, to avoid the potential confounding effect of communication about the intervention among physicians and/or cross-covering of patients within the practice" (cited in Weber, White & McIlvried 2008).
	1a.2 Was the allocation sequence concealed until clusters were enrolled and assigned to interventions?	NI	

	1a.3 Did baseline differences between intervention groups suggest a problem with the randomization process?	PN	Table 1 in Weber, White & McIlvried 2008 indicates that any observed differences in prognostic factors between study groups are compatible with chance.
	**Risk of bias judgement**	**Some concerns**	
**Bias arising from the timing of identification or recruitment of participants**	1b.1 Were all the individual participants identified and recruited (if appropriate) before randomization of clusters?	**PN**	Clusters consisted of primary care practices, and patients have the opportunity change primary care physicians, e.g. if they want another physician or if they have moved. Therefore, although most patients were likely identified before randomisation of clusters, some patients may have been recruited after randomisation.
	1b.2 If N/PN/NI to 1b.1: Is it likely that selection of individual participants was affected by knowledge of the intervention assigned to the cluster?	**ow of the study before they chose primary care physician.**	
	1b.3 Were there baseline imbalances that suggest differential identification or recruitment of individual participants between intervention groups?	**PN**	See description for question 1a.3
	**Risk of bias judgement**	**Low**	Low
**Bias due to deviations from intended interventions**	2.1a Were participants aware that they were in a trial?	PY	Participants self-reported falls
	2.1b If Y/PY/NI to 2.1a: Were participants aware of their assigned intervention during the trial?	NI	Unable to find information
	2.2 Were carers and people delivering the interventions aware of participants' assigned intervention during the trial?	PY	
	2.3 If Y/PY/NI to 2.1b or 2.2: Were there deviations from the intended intervention that arose because of the trial context?	PN	Unlikely that intervention contamination caused deviations as the intervention was implemented at entire primary care practices.
	2.4 If Y/PY to 2.3: Were these deviations likely to have affected the outcome?	NA	
	2.5 If Y/PY/NI to 2.4: Were these deviations from intended intervention balanced between groups?	NA	
	2.6 Was an appropriate analysis used to estimate the effect of assignment to intervention?	NI	Unable to find information about whether or not all cluster and individuals were analysed according to the groups to which they were assigned.
	2.7 If N/PN/NI to 2.6: Was there potential for a substantial impact (on the result) of the failure to analyse participants in the group to which they were randomized ?	PN	
	**Risk of bias judgement**	**Some concerns**	
**Bias due to missing outcome data**	3.1a Were data for this outcome available for all clusters that recruited participants?	NI	"To identify falls, we obtained data on all medical encounters (inpatient hospitalizations, emergency department encounters, and outpatient visits). This data included dates of service, provider type, place of service, provider name, primary and secondary diagnosis, procedure code, and payment amount. We counted both encounters in which a fall was identified as a diagnosis and those with 1 or more diagnoses for a potential fall-related diagnosis, including head injury, fracture, hip injury, sprain, abrasion, or concussion" (cited in journal article).
	3.1b Were data for this outcome available for all, or nearly all, participants within clusters?	PN	"By the last patient survey at 15 months, 158 patients (76 %) in the comparison group and 337 (81.6%) in the intervention group were able to respond" (cited in journal article).
	3.2 If N/PN/NI to 3.1a or 3.1b: Is there evidence that the result was not biased by missing data?	PN	
	3.3 If N/PN to 3.2 Could missingness in the outcome depend on its true value?	PY	
	3.4 If Y/PY/NI to 3.3: Is it likely that missingness in the outcome depended on its true value?	PN	
	**Risk of bias judgement**	**Some concerns**	
**Bias in measurement of the outcome**	4.1 Was the method of measuring the outcome inappropriate?	PN	"To identify falls, we obtained data on all medical encounters (inpatient hospitalizations, emergency department encounters, and outpatient visits) ... To obtain direct information from patients regarding self-reported fall rates, patients were contacted by telephone by a study nurse at months 1, 3, 6, 9, 12 and 15, who collected data on self-reported falls. The nurse used a standardized definition of falls using a scripted template; no advice or other information was given. Thus, both self-reported fall rates and those requiring medical attention were captured." (cited in Weber, White & McIlvried 2008).
	4.2 Could measurement or ascertainment of the outcome have differed between intervention groups?	PN	The method of collecting data for falls and fall-related injuries was planned before the start of follow-up and was active (i.e. not passive such as for unplanned adverse events) and systematic.
	4.3a If N/PN/NI to 4.1 and 4.2: Were outcome assessors aware that a trial was taking place?	PY	I can not find information on blinding of of intervention providers, study participants, or outcome assessors. Persons collecting data on medical encounters were most likely aware that a trial was taking place.
	4.3b If Y/PY/NI to 4.3a: Were outcome assessors aware of the intervention received by study participants?	PY	I can not find information on blinding of of intervention providers, study participants, or outcome assessors

	4.4 If Y/PY/NI to 4.3b: Could assessment of the outcome have been influenced by knowledge of intervention received?	PY	Self-report on falls is a participant-reported outcome and may be sensitive to knowledge of the intervention received.
	4.5 If Y/PY/NI to 4.4: Is it likely that assessment of the outcome was influenced by knowledge of intervention received?	PN	
	**Risk of bias judgement**	**Some concerns**	
**Bias in selection of the reported result**	5.1 Were the data that produced this result analysed in accordance with a pre-specified analysis plan that was finalized before unblinded outcome data were available for analysis?	NI	No trial protocol, statistical analysis plan or trial registry record available.
	5.2 ... multiple eligible outcome measurements (e.g. scales, definitions, time points) within the outcome domain?	NI	Unable to find information
	5.3 ... multiple eligible analyses of the data?	NI	Unable to find information
	**Risk of bias judgement**		
**Overall bias**	**Risk of bias judgement**	**High**	This result is considered at high risk of bias because there are some concerns for a risk of bias in four domains.

**Unique ID**	Weber, White & McIlvried(2008)_B	**Study ID**	1052	**Assessor**	RS
**Ref or Label**		**Aim**	assignment to intervention (the 'intention-to-treat' effect)		
**Experimental**	Standardized medication review with recommendations to primary care physician via the electronic medical record	**Comparator**	Usual care without medication review and recommendations sent to primary care physician	**Source**	Journal article(s)
**Outcome**	Change in number of active medications during 12 months follow-up in intervention group compared with control group	**Results**	Mean difference -0.496 (p < 0.10)	**Weight**	1

Domain	Signalling question	Response	Comments
**Bias arising from the randomization process**	1a.1 Was the allocation sequence random?	NI	It says in the journal article that the clinic sites were randomized, but not how the allocation sequence was generated.<br><br>"We then randomized clinic sites to receive either the intervention or usual care. Fifteen clinic sites received the electronic intervention, whereas 3 clinic sites served as controls. Randomization occurred according to clinic site, not physician, to avoid the potential confounding effect of communication about the intervention among physicians and/or cross-covering of patients within the practice" (cited in Weber, White & McIlvried 2008).
	1a.2 Was the allocation sequence concealed until clusters were enrolled and assigned to interventions?	NI	
	1a.3 Did baseline differences between intervention groups suggest a problem with the randomization process?	PN	Table 1 in Weber, White & McIlvried 2008 indicates that any observed differences in prognostic factors between study groups are compatible with chance.
	**Risk of bias judgement**	**Some concerns**	
**Bias arising from the timing of identification or recruitment of participants**	1b.1 Were all the individual participants identified and recruited (if appropriate) before randomization of clusters?	**PN**	Clusters consisted of primary care practices, and patients have the opportunity change primary care physicians, e.g. if they want another physician or if they have moved. Therefore, although most patients were likely identified before randomisation of clusters, some patients may have been recruited after randomisation.
	1b.2 If N/PN/NI to 1b.1: Is it likely that selection of individual participants was affected by knowledge of the intervention assigned to the cluster?	**ow of the study before they chose primary care physician.**	
	1b.3 Were there baseline imbalances that suggest differential identification or recruitment of individual participants between intervention groups?	**PN**	See description for question 1a.4
	**Risk of bias judgement**	**Low**	Low
**Bias due to deviations from intended interventions**	2.1a Were participants aware that they were in a trial?	PY	Participants self-reported falls
	2.1b If Y/PY/NI to 2.1a: Were participants aware of their assigned intervention during the trial?	NI	Unable to find information
	2.2 Were carers and people delivering the interventions aware of participants' assigned intervention during the trial?	PY	
	2.3 If Y/PY/NI to 2.1b or 2.2: Were there deviations from the intended intervention that arose because of the trial context?	PN	Unlikely that intervention contamination caused deviations as the intervention was implemented at entire primary care practices.
	2.4 If Y/PY to 2.3: Were these deviations likely to have affected the outcome?	NA	
	2.5 If Y/PY/NI to 2.4: Were these deviations from intended intervention balanced between groups?	NA	
	2.6 Was an appropriate analysis used to estimate the effect of assignment to intervention?	NI	Unable to find information about whether or not all cluster and individuals were analysed according to the groups to which they were assigned.
	2.7 If N/PN/NI to 2.6: Was there potential for a substantial impact (on the result) of the failure to analyse participants in the group to which they were randomized ?	PN	
	**Risk of bias judgement**	**Some concerns**	

**Bias due to missing outcome data**	3.1a Were data for this outcome available for all clusters that recruited participants?	PY	The EPICcare database was queried to generate data files on medication usage, which contained information on order date, starting date, medication ID and description, quantity, number of refills, and daily dosage amounts. It is likely that the EPICcare database contained information on medication use for most or all participants.
	3.1b Were data for this outcome available for all, or nearly all, participants within clusters?	PY	
	3.2 If N/PN/NI to 3.1a or 3.1b: Is there evidence that the result was not biased by missing data?	NA	
	3.3 If N/PN to 3.2 Could missingness in the outcome depend on its true value?	NA	
	3.4 If Y/PY/NI to 3.3: Is it likely that missingness in the outcome depended on its true value?	NA	
	**Risk of bias judgement**	**Low**	
**Bias in measurement of the outcome**	4.1 Was the method of measuring the outcome inappropriate?	PN	"Medication patterns were evaluated to create measures of the total number of medications that were active in a given time period (with time periods defined relative to the intervention start date), the total number of medications that were started during a given time period, the total number of psychoactive medications that were active in a given time period, and the total number of psychoactive medications that were started during a given time period" (cited in journal article).
	4.2 Could measurement or ascertainment of the outcome have differed between intervention groups?	PN	The method of collecting data for falls and fall-related injuries was planned before the start of follow-up and was active (i.e. not passive such as for unplanned adverse events) and systematic.
	4.3a If N/PN/NI to 4.1 and 4.2: Were outcome assessors aware that a trial was taking place?	PY	I can not find information on blinding of of intervention providers, study participants, or outcome assessors.
	4.3b If Y/PY/NI to 4.3a: Were outcome assessors aware of the intervention received by study participants?	PY	I can not find information on blinding of of intervention providers, study participants, or outcome assessors
	4.4 If Y/PY/NI to 4.3b: Could assessment of the outcome have been influenced by knowledge of intervention received?	PY	The number of active medications is an outcome that reflects decisions made by the intervention provider. The process of making the decisions can be influenced by knowledge of the intervention received, but the recording of the decision does not involve any judgement.
	4.5 If Y/PY/NI to 4.4: Is it likely that assessment of the outcome was influenced by knowledge of intervention received?	PN	
	**Risk of bias judgement**	**Some concerns**	
**Bias in selection of the reported result**	5.1 Were the data that produced this result analysed in accordance with a pre-specified analysis plan that was finalized before unblinded outcome data were available for analysis?	NI	No trial protocol, statistical analysis plan or trial registry record available.
	5.2 ... multiple eligible outcome measurements (e.g. scales, definitions, time points) within the outcome domain?	NI	Unable to find information
	5.3 ... multiple eligible analyses of the data?	NI	Unable to find information
	**Risk of bias judgement**		
**Overall bias**	**Risk of bias judgement**	**High**	This result is considered at high risk of bias because there are some concerns for a risk of bias in four domains.

Risk of bias in results of cluster-randomised trials

Unique ID	Outcome	D1a	D1b	D2	D3	D4	D5	Overall
Aizen(2015)_A	Fall rate per 1000 bed-days in intervention group compared with control group (of participants admitted during the first phase of the study, three months of follow-up)	!	+	-	+	+	!	-
Barker(2016)_A	Rate of use of all 6-PACK programme components (fall risk tool and six interventions) per 1000 occupied bed days in intervention group compared with control group at 8 months follow-up	+	!	+	+	!	+	!
Barker(2016)_B	Rate of falls per 1000 occupied bed days during 12 months follow-up in intervention group compared with control group	+	!	+	+	+	+	!
Bhasin(2020)_A	Rate of times to first adjudicated serious fall injury per 100 person-years during 44 months follow-up in intervention group compared with control group	+	+	+	+	+	+	+
Blalock(2020)_A	Change in use of fall risk-increasing drugs (Drug Burden Index score) from 12-month pre-intervention period to 12-month post-intervention period (among participants who screened positive for fall risk) in intervention group compared with control group	!	+	!	+	+	!	!
Blalock(2020)_B	Risk of falling during 12-month post-intervention period, controlled for risk of falling during 12-month pre-intervention period, in intervention group compared with control group	!	+	!	+	+	!	!
Blum (2021)_A	Rate of falls per person-year during 12 months follow-up in intervention group compared with control group	+	+	!	+	-	+	-
Blum (2021)_B	Presence of drug overuse (based on STOPP criteria) during two months follow-up in intervention group compared with control group	+	+	!	+	+	!	!
Carroll(2012)_A	Documentation of fall risk (percentage of patients who had their fall risk documented) during 6 months follow-up	!	+	!	+	+	!	-
Clemson (2024)_A	Rate of falls, self-reported by patients using daily fall calendar mailed on a monthly basis, over 12 months follow-up in intervention group compared with control group	+	+	-	+	+	+	-
Clemson (2024)_B	Changes in GPs' engagement in fall prevention activities at three months follow-up, including risk assessment, medication reviews, and providing advice, compared to the control group	+	+	-	+	+	+	-
Dykes(2010)_A	Rate of patient falls per 1000 patient-days at six months follow-up in intervention group compared with control group (sensitivity analysis with patients aged 65 years or older)	!	+	!	+	+	!	-
Dykes(2010)_B	Adherence to intervention protocol (Morse Falls Scale completion)	!	+	!	+	!	!	-
Ganz(2022)_A	Rate of falls per 100 person-years during 27 months follow-up in intervention group compared with control group	+	+	!	+	+	+	!
Healey(2004)_A	Risk of falling in intervention group compared with control group as a change from the six-month period before intervention implementation to the six-month period after intervention implementation	!	+	!	+	+	!	-
Logan (2021)_A	Fall rate per 1000 resident-days between 91 and 180 days after randomisation in intervention group compared with control group	+	+	+	+	+	+	+
Phelan (2024)_A	Time to first medically treatd fall at 18 months follow-up	+	!	!	+	+	+	!
Phelan (2024)_B	Discontinuation of medications (defined as no prescription fill for 90 days), summarized across all target medication classes, referred to as "first target medication", at six months follow-up.	+	!	!	+	+	!	-
Tamblyn(2012)_A	Mean reduction in risk of injury per 1000 patients during 23 months follow-up in intervention group compared with control group	+	+	+	+	+	+	+
Snooks(2014)_A	Odds of a patient being referred to a falls service during one month follow-up in intervention group compared with control group	+	+	!	+	+	!	!
Snooks(2014)_B	Odds of a patient reporting ≥1 falls (self-report) during one month follow-up in intervention group compared with control group	+	+	!	+	+	!	!
Weber, White & M	Odds of having had one or more fall-related medical encounters (as determined by EpicCare data only) during 15 months follow-up in intervention group compared with control group	!	+	!	!	!	!	-
Weber, White & M	Change in number of active medications during 12 months follow-up in intervention group compared with control group	!	+	!	+	!	!	-

+ Low risk
! Some concerns
- High risk

D1a Randomisation process
D1b Timing of identification or recruitment of participants
D2 Deviations from the intended interventions
D3 Missing outcome data
D4 Measurement of the outcome
D5 Selection of the reported result

Risk of bias assessments for non-randomised studies of the effects of interventions

Study	Bias due to confounding	Bias in selection of participants into the study	Bias in classification of interventions	Bias due to deviations from intended interventions	Bias due to missing data	Bias in measurement of outcomes	Bias in selection of the reported result	Overall Bias
Dykes (2020)_A	Serious	Low	Serious	Low	Low	Low	Moderate	Serious
Ganz (2015)_A	Serious	Low	Low	Low	Moderate	Low	Low	Serious
Wenger (2010)_A	Serious	Low	Low	Low	Low	Moderate	NI	Serious
Groshaus (2012)_A	Serious	Low	NI	NI	Low	Low	NI	Serious
Groshaus (2012)_B	Serious	Low	NI	NI	Low	Low	NI	Serious
Wenger (2009)_A	Serious	Low	Low	Low	Low	Low	NI	Serious
Byrne (2005)_A	Serious	Low	Serious	NI	NI	NI	NI	Serious

Risk of bias assessments for individually randomised parallel group trials

**Unique ID**	Elley(2008)_A	**Study ID**	928	**Assessor**	RS
**Ref or Label**		**Aim**	assignment to intervention (the 'intention-to-treat' effect)		
**Experimental**	Falls-and-fracture nurse coordinator and multifactorial intervention	**Comparator**	Usual care plus an offer of two social visits	**Source**	Journal article(s); Non-commercial trial registry record (e.g. ClinicalTrials.gov record)
**Outcome**	Rate of falls per person-year at 12 months follow-up in intervention group compared with control group	**Results**	Rate ratio 0.96 (95% CI 0.70, 1.34)	**Weight**	1

Domain	Signalling question	Response	Comments
**Bias arising from the randomization process**	1.1 Was the allocation sequence random?	Y	"An independent researcher at a distant site carried out computer randomisation of participants, emailing allocation of randomisation of each individual after baseline assessment" (cited in trial protocol).
	1.2 Was the allocation sequence concealed until participants were enrolled and assigned to interventions?	Y	
	1.3 Did baseline differences between intervention groups suggest a problem with the randomization process?	N	"Baseline characteristics were balanced between the two groups (Table 1)" (cited in journal article).<br><br>Also, there were no baseline differences between the intervention groups in the outcomes such as the number of medications, potentially inappropriate prescriptions (PIPs), or potential prescription omissions (PPOs).
	**Risk of bias judgement**	**Low**	
**Bias due to deviations from intended interventions**	2.1.Were participants aware of their assigned intervention during the trial?	Y	"The research nurses who undertake outcome measures at each time point remain blind to allocation to minimise measurement bias, although blinding is difficult where the participant is aware of which group they are<br>in and home alterations may be evident at follow-up in the homes of some intervention participants" (cited in trial protocol).
	2.2.Were carers and people delivering the interventions aware of participants' assigned intervention during the trial?	Y	
	2.3. If Y/PY/NI to 2.1 or 2.2: Were there deviations from the intended intervention that arose because of the experimental context?	PN	"All control participants were offered social visits, and 75 of 157 (48%) accepted" (cited in journal article).<br><br>Any deviations from intended intervention are likely to be part of usual clinical practice.
	2.4 If Y/PY to 2.3: Were these deviations likely to have affected the outcome?	NA	
	2.5. If Y/PY/NI to 2.4: Were these deviations from intended intervention balanced between groups?	NA	
	2.6 Was an appropriate analysis used to estimate the effect of assignment to intervention?	PY	The trial protocol states that the analysis for the main outcome will be conducted according to ITT principles, and that negative binomial regression will be used to compare rate of falls.
	2.7 If N/PN/NI to 2.6: Was there potential for a substantial impact (on the result) of the failure to analyse participants in the group to which they were randomized?	NA	
	**Risk of bias judgement**	**Low**	
**Bias due to missing outcome data**	3.1 Were data for this outcome available for all, or nearly all, participants randomized?	Y	Table 2 in the journal article shows that all participants who were allocation to the study groups were also analysed for falls rate.
	3.2 If N/PN/NI to 3.1: Is there evidence that result was not biased by missing outcome data?	NA	
	3.3 If N/PN to 3.2: Could missingness in the outcome depend on its true value?	NA	
	3.4 If Y/PY/NI to 3.3: Is it likely that missingness in the outcome depended on its true value?	NA	
	**Risk of bias judgement**	**Low**	
**Bias in measurement of the outcome**	4.1 Was the method of measuring the outcome inappropriate?	PN	"Falls are recorded by participants using postcard calendars, completed daily and posted monthly to the research team. If a fall is indicated on the calendar, a follow-up telephone interview establishes the circumstances and consequences of the fall from the participant, including injury and hospital admission. In a few cases, reports were confirmed<br>from hospital records" (cited in trial protocol).
	4.2 Could measurement or ascertainment of the outcome have differed between intervention groups?	PN	The method of measuring falls seems thorough and was based on ProFaNE recommenations.
	4.3 Were outcome assessors aware of the intervention received by study participants?	N	"The research nurses who recorded the demographic, clinical, and outcome measures at baseline and after 1 year, remained blind to group allocation" (cited in journal article).

	4.4 If Y/PY/NI to 4.3: Could assessment of the outcome have been influenced by knowledge of intervention received?	NA	
	4.5 If Y/PY/NI to 4.4: Is it likely that assessment of the outcome was influenced by knowledge of intervention received?	NA	
	**Risk of bias judgement**	**Low**	
**Bias in selection of the reported result**	5.1 Were the data that produced this result analysed in accordance with a pre-specified analysis plan that was finalized before unblinded outcome data were available for analysis?	PY	The trial protocol was received 20.12.2006 and was published 29.07.2007. It is stated in the journal article that the trial was conducted between March 2005 and February 2007. Given that the protocol was received before the trial ended, unblinded outcome data was likely not available before the trial protocol was finished.
	5.2 ... multiple eligible outcome measurements (e.g. scales, definitions, time points) within the outcome domain?	PN	It is stated in the trial protocol that the study uses standardised definitions and outcome measures recommended by ProFaNE to allow pooling of results with other trials. The trial protocol states that the analysis for the main outcome will be conducted according to ITT principles, and that negative binomial regression will be used to compare rate of falls.
	5.3 ... multiple eligible analyses of the data?	PN	
	**Risk of bias judgement**	**Low**	
**Overall bias**	**Risk of bias judgement**	**Low**	

**Unique ID**	Ferrer(2014)_A	**Study ID**	2305	**Assessor**	RS
**Ref or Label**		**Aim**	assignment to intervention (the 'intention-to-treat' effect)		
**Experimental**	Multifactorial fall risk assessment and treatment recommendations made to patient and family physician based on algorithm	**Comparator**	Usual care	**Source**	Journal article(s); Trial protocol; Non-commercial trial registry record (e.g. ClinicalTrials.gov record)
**Outcome**	Risk of falling during 24 months follow-up in intervention group compared with control group	**Results**	Relative Risk 1.28 (95% CI 0.94, 1.75)	**Weight**	1

**Domain**	**Signalling question**	**Response**	**Comments**
**Bias arising from the randomization process**	1.1 Was the allocation sequence random?	Y	"... the subjects were randomized to an intervention or control group using a computer-generated randomization table" (cited in journal article).<br><br>Unable to find information on whether the allocation sequence was concealed to the person responsible for allocation participants to groups.
	1.2 Was the allocation sequence concealed until participants were enrolled and assigned to interventions?	NI	
	1.3 Did baseline differences between intervention groups suggest a problem with the randomization process?	N	"The baseline characteristics of the two study groups were similar (Table 1), although subjects in the control group reported more barriers (P=0.03) and had more anemia (P=0.03)" (cited in journal article).
	**Risk of bias judgement**	**Some concerns**	
**Bias due to deviations from intended interventions**	2.1.Were participants aware of their assigned intervention during the trial?	Y	This was a single-blind trial, and only independent assessors at each center who collected data via telephone were unaware of group allocation.
	2.2.Were carers and people delivering the interventions aware of participants' assigned intervention during the trial?	Y	
	2.3. If Y/PY/NI to 2.1 or 2.2: Were there deviations from the intended intervention that arose because of the experimental context?		
	2.4 If Y/PY to 2.3: Were these deviations likely to have affected the outcome?	NA	
	2.5. If Y/PY/NI to 2.4: Were these deviations from intended intervention balanced between groups?	NA	
	2.6 Was an appropriate analysis used to estimate the effect of assignment to intervention?	NA	
	2.7 If N/PN/NI to 2.6: Was there potential for a substantial impact (on the result) of the failure to analyse participants in the group to which they were randomized?	NA	
	**Risk of bias judgement**	**High**	
**Bias due to missing outcome data**	3.1 Were data for this outcome available for all, or nearly all, participants randomized?	Y	All 328 patients allocation to the two study groups were included in intention to treat falls analyses.
	3.2 If N/PN/NI to 3.1: Is there evidence that result was not biased by missing outcome data?	NA	
	3.3 If N/PN to 3.2: Could missingness in the outcome depend on its true value?	NA	
	3.4 If Y/PY/NI to 3.3: Is it likely that missingness in the outcome depended on its true value?	NA	
	**Risk of bias judgement**	**Low**	
	4.1 Was the method of measuring the outcome inappropriate?	N	"Falls were ascertained during the annual assessment by self-report on the monthly calendar and from medical records. The health care professional telephoned participants at 3-monthly intervals to collect information from the calendar. "

**Bias in measurement of the outcome**	4.2 Could measurement or ascertainment of the outcome have differed between intervention groups?	N	All participants were telephoned, and the healthcare professional who made the calls were unaware of participants' intervention status.
	4.3 Were outcome assessors aware of the intervention received by study participants?	Y	Falls were self-reported, so the study participants were the outcome assessors. However, the healthcare professional was unaware of the participants' intervention status: "To ensure blinding during data collection, measurements done by telephone were delegated to independent assessors at each center who were unaware of group allocation" (cited in journal article).
	4.4 If Y/PY/NI to 4.3: Could assessment of the outcome have been influenced by knowledge of intervention received?	PN	
	4.5 If Y/PY/NI to 4.4: Is it likely that assessment of the outcome was influenced by knowledge of intervention received?	NA	
	**Risk of bias judgement**	**Low**	
**Bias in selection of the reported result**	5.1 Were the data that produced this result analysed in accordance with a pre-specified analysis plan that was finalized before unblinded outcome data were available for analysis?	PY	The trial was conducted from January 2009 to December 2010. The last updated posted on ClinicalTrials.gov was 10.06.2010. The trial protocol was published March 2010.
	5.2 ... multiple eligible outcome measurements (e.g. scales, definitions, time points) within the outcome domain?	NI	Unable to find information about this. The trial protocol is only available in Spanish.
	5.3 ... multiple eligible analyses of the data?	NI	
	**Risk of bias judgement**	**Some concerns**	
**Overall bias**	**Risk of bias judgement**	**High**	

**Unique ID**	Frankenthal(2014)_A	**Study ID**	2012	**Assessor**	RS
**Ref or Label**		**Aim**	assignment to intervention (the 'intention-to-treat' effect)		
**Experimental**	Screening medications with STOPP/START criteria followed up with recommendations to the chief physician	**Comparator**	Usual pharmaceutical care	**Source**	Journal article(s); Non-commercial trial registry record (e.g. ClinicalTrials.gov record)
**Outcome**	Proportion of participants with potentially inappropriate prescriptions (PIPs) at 12-month follow-up	**Results**	22.5% in intervention group compared with 54.1% in control group (p < 0.001)	**Weight**	1

Domain	Signalling question	Response	Comments
**Bias arising from the randomization process**	1.1 Was the allocation sequence random?	Y	"A physician who was not part of the study randomized participants. Fixed stratified randomization was used to allocate residents to groups according to the three types of residents: ADL-dependent, ADL-independent, and primarily cognitively impaired. Subjects who were ADL-dependent with impaired cognition were assigned to the ADL-dependent group. Randomization for each level was according to simple list randomization" (cited in Frankenthal et al. 2014).
	1.2 Was the allocation sequence concealed until participants were enrolled and assigned to interventions?	PY	
	1.3 Did baseline differences between intervention groups suggest a problem with the randomization process?	PN	"There were no significant baseline differences between the two groups in terms of age, sex, Charlson Comorbidity Index (CCI), frequency of falls and hospitalizations in the preceding year, functional status, cognitive level, quality of life, prevalence of chronic illnesses and medications, average number of medications, and average monthly medication costs (Table 1)" (cited in Frankenthal et al. 2014).
	**Risk of bias judgement**	**Low**	
**Bias due to deviations from intended interventions**	2.1.Were participants aware of their assigned intervention during the trial?	N	According to the trial registration in ClinicalTrials.gov partcipants were blinded.<br><br>"The study pharmacist and the chief physician were not blinded to group assignment after randomization, but the other physicians and medical staff in the facility were blinded to the intervention assignment and the interventional recommendations" (cited in Frankenthal et al. 2014).
	2.2.Were carers and people delivering the interventions aware of participants' assigned intervention during the trial?	Y	
	2.3. If Y/PY/NI to 2.1 or 2.2: Were there deviations from the intended intervention that arose because of the experimental context?	PY	The study pharmacist provided both the experimental intervention and the control intervention.
	2.4 If Y/PY to 2.3: Were these deviations likely to have affected the outcome?		
	2.5. If Y/PY/NI to 2.4: Were these deviations from intended intervention balanced between groups?		
	2.6 Was an appropriate analysis used to estimate the effect of assignment to intervention?		
	2.7 If N/PN/NI to 2.6: Was there potential for a substantial impact (on the result) of the failure to analyse participants in the group to which they were randomized?		

Domain	Signalling question	Response	Comments
	**Risk of bias judgement**		
**Bias due to missing outcome data**	3.1 Were data for this outcome available for all, or nearly all, participants randomized?	N	According to table 3 in Frankenthal et al. 2014 outcome data at 12-month follow-up were available for 306 participants out of 359 participants who were included in the study.
	3.2 If N/PN/NI to 3.1: Is there evidence that result was not biased by missing outcome data?	PN	The statistical analyses did not correct for potential bias and no sensitivity analyses were conducted to show that the result was not sensitive to a range of plausible assumptions about the relationship between missingness in the outcome and its true value.
	3.3 If N/PN to 3.2: Could missingness in the outcome depend on its true value?	PY	The proportions of participants that did not provide outcome data were similar across the study groups (Figure 1). Figure 1 shows that in the intervention group 15 participants died and 8 participants left the facility in the intervention group, and 17 patients died and 13 left the facility in the control group.
	3.4 If Y/PY/NI to 3.3: Is it likely that missingness in the outcome depended on its true value?	PN	
	**Risk of bias judgement**	**Some concerns**	
**Bias in measurement of the outcome**	4.1 Was the method of measuring the outcome inappropriate?	PN	Har spurt korresponderende forfatter om hvordan data om PIPs ble målt
	4.2 Could measurement or ascertainment of the outcome have differed between intervention groups?		
	4.3 Were outcome assessors aware of the intervention received by study participants?		"Nurses who were unaware of participants' group assignments assessed the outcome measures in the study population" (cited in Frankenthal et al. 2014).
	4.4 If Y/PY/NI to 4.3: Could assessment of the outcome have been influenced by knowledge of intervention received?		
	4.5 If Y/PY/NI to 4.4: Is it likely that assessment of the outcome was influenced by knowledge of intervention received?		
	**Risk of bias judgement**		
**Bias in selection of the reported result**	5.1 Were the data that produced this result analysed in accordance with a pre-specified analysis plan that was finalized before unblinded outcome data were available for analysis?		Har etterspurt studieprotokoll eller SAP.
	5.2 ... multiple eligible outcome measurements (e.g. scales, definitions, time points) within the outcome domain?		
	5.3 ... multiple eligible analyses of the data?		
	**Risk of bias judgement**		
**Overall bias**	**Risk of bias judgement**		

**Unique ID**	Frankenthal(2014)_B	**Study ID**	2012	**Assessor**	RS
**Ref or Label**		**Aim**	assignment to intervention (the 'intention-to-treat' effect)		
**Experimental**	Screening medications with STOPP/START criteria followed up with recommendations to the chief physician	**Comparator**	Usual pharmaceutical care	**Source**	Journal article(s); Non-commercial trial registry record (e.g. ClinicalTrials.gov record)
**Outcome**	Average costs of medications per month (in Israeli shekels) at 12-month follow-up	**Results**	Mean (SD) of 279 (171.9) in intervention group compared with 402.3 (291.2) in control group (p < 0.001)	**Weight**	1

Domain	Signalling question	Response	Comments
**Bias arising from the randomization process**	1.1 Was the allocation sequence random?	Y	"A physician who was not part of the study randomized participants. Fixed stratified randomization was used to allocate residents to groups according to the three types of residents: ADL-dependent, ADL-independent, and primarily cognitively impaired. Subjects who were ADL-dependent with impaired cognition were assigned to the ADL-dependent group. Randomization for each level was according to simple list randomization" (cited in Frankenthal et al. 2014).
	1.2 Was the allocation sequence concealed until participants were enrolled and assigned to interventions?	PY	
	1.3 Did baseline differences between intervention groups suggest a problem with the randomization process?	PN	"There were no significant baseline differences between the two groups in terms of age, sex, Charlson Comorbidity Index (CCI), frequency of falls and hospitalizations in the preceding year, functional status, cognitive level, quality of life, prevalence of chronic illnesses and medications, average number of medications, and average monthly medication costs (Table 1)" (cited in Frankenthal et al. 2014).
	**Risk of bias judgement**	**Low**	
	2.1.Were participants aware of their assigned intervention during the trial?	N	According to the trial registration in ClinicalTrials.gov partcipants were blinded.

**Bias due to deviations from intended interventions**	2.2.Were carers and people delivering the interventions aware of participants' assigned intervention during the trial?	Y	[illegible] "The study pharmacist and the chief physician were not blinded to group assignment after randomization, but the other physicians and medical staff in the facility were blinded to the intervention assignment and the interventional recommendations" (cited in Frankenthal et al. 2014).
	2.3. If Y/PY/NI to 2.1 or 2.2: Were there deviations from the intended intervention that arose because of the experimental context?	PY	The study pharmacist provided both the experimental intervention and the control intervention.
	2.4 If Y/PY to 2.3: Were these deviations likely to have affected the outcome?		
	2.5. If Y/PY/NI to 2.4: Were these deviations from intended intervention balanced between groups?		
	2.6 Was an appropriate analysis used to estimate the effect of assignment to intervention?		
	2.7 If N/PN/NI to 2.6: Was there potential for a substantial impact (on the result) of the failure to analyse participants in the group to which they were randomized?		
	**Risk of bias judgement**		
**Bias due to missing outcome data**	3.1 Were data for this outcome available for all, or nearly all, participants randomized?	N	According to table 3 in Frankenthal et al. 2014 outcome data at 12-month follow-up were available for 306 participants out of 359 participants who were included in the study.
	3.2 If N/PN/NI to 3.1: Is there evidence that result was not biased by missing outcome data?	PN	The statistical analyses did not correct for potential bias and no sensitivity analyses were conducted to show that the result was not sensitive to a range of plausible assumptions about the relationship between missingness in the outcome and its true value.
	3.3 If N/PN to 3.2: Could missingness in the outcome depend on its true value?	PY	The proportions of participants that did not provide outcome data were similar across the study groups (Figure 1). Figure 1 shows that in the intervention group 15 participants died and 8 participants left the facility in the intervention group, and 17 patients died and 13 left the facility in the control group.
	3.4 If Y/PY/NI to 3.3: Is it likely that missingness in the outcome depended on its true value?	PN	
	**Risk of bias judgement**	**Some concerns**	
**Bias in measurement of the outcome**	4.1 Was the method of measuring the outcome inappropriate?		
	4.2 Could measurement or ascertainment of the outcome have differed between intervention groups?		
	4.3 Were outcome assessors aware of the intervention received by study participants?		
	4.4 If Y/PY/NI to 4.3: Could assessment of the outcome have been influenced by knowledge of intervention received?		
	4.5 If Y/PY/NI to 4.4: Is it likely that assessment of the outcome was influenced by knowledge of intervention received?		
	**Risk of bias judgement**		
**Bias in selection of the reported result**	5.1 Were the data that produced this result analysed in accordance with a pre-specified analysis plan that was finalized before unblinded outcome data were available for analysis?		
	5.2 ... multiple eligible outcome measurements (e.g. scales, definitions, time points) within the outcome domain?		
	5.3 ... multiple eligible analyses of the data?		
	**Risk of bias judgement**		
**Overall bias**	**Risk of bias judgement**		

**Unique ID**	Mahoney(2007)_A	**Study ID**	2066	**Assessor**	RS
**Ref or Label**		**Aim**	assignment to intervention (the 'intention-to-treat' effect)		
**Experimental**	Intermediate-intensity, community-based multifactorial falls intervention	**Comparator**	Home safety visits	**Source**	Journal article(s)
**Outcome**	Rate of falls per person-year (365.25 days) during 12 months follow-up in intervention group compared with control group	**Results**	Rate Ratio 0.81 (95% CI 0.57, 1.17; p = 0.27)	**Weight**	1

Domain	Signalling question	Response	Comments
**Bias arising from the randomization process**	1.1 Was the allocation sequence random?	Y	"Randomization to intervention or control groups was based on a computer-generated randomization table. After obtaining informed consent and before group assignment, the study researcher performed a baseline in-home assessment for function, health services use, and risk factors for falls. After baseline assessment, a research staff member opened a sealed envelope with study group assignment" (Mahoney et al. 2007).
	1.2 Was the allocation sequence concealed until participants were enrolled and assigned to interventions?	PY	

	1.3 Did baseline differences between intervention groups suggest a problem with the randomization process?	PN	Table 2 in Mahoney et al. 2007 shows a distribution of baseline characteristics between intervention groups that seems compatible with chance.
	**Risk of bias judgement**	**Low**	
**Bias due to deviations from intended interventions**	2.1.Were participants aware of their assigned intervention during the trial?	PY	"This study has a number of limitations. First, it was only single-blinded" (cited in Mahoney et al. 2007). In this study the study researcher who called subjects who did not return calendars was blinded.
	2.2.Were carers and people delivering the interventions aware of participants' assigned intervention during the trial?	Y	
	2.3. If Y/PY/NI to 2.1 or 2.2: Were there deviations from the intended intervention that arose because of the experimental context?	PY	"This study has a number of limitations ... Third, adherence to exercise relied on self-report, which may be subject to bias. Fourth, it is not known to what extent the effect in the group with low MMSE scores was due to starting medications for dementia. Fifth, contamination of the control group was possible, which may also have biased toward the null. Control subjects may have sought out other services for falls prevention as a result of the occupational therapist's visit, and physicians and physical therapists in Kenosha County may have increased their awareness of falls and appropriate therapies as a secondary effect of the intervention, thus potentially improving care for control patients" (cited in Mahoney et al. 2007).
	2.4 If Y/PY to 2.3: Were these deviations likely to have affected the outcome?	PY	
	2.5. If Y/PY/NI to 2.4: Were these deviations from intended intervention balanced between groups?	PN	
	2.6 Was an appropriate analysis used to estimate the effect of assignment to intervention?	PY	"All analyses were conducted based on intention-to-treat principle" (cited in Mahoney et al. 2007).
	2.7 If N/PN/NI to 2.6: Was there potential for a substantial impact (on the result) of the failure to analyse participants in the group to which they were randomized?	NA	
	**Risk of bias judgement**	**High**	
**Bias due to missing outcome data**	3.1 Were data for this outcome available for all, or nearly all, participants randomized?	PN	"Five subjects (3 in the control group, 2 in the intervention group) had no postrandomization follow-up. Of the remaining 344 subjects, 274 (80%) had at least 365 days of follow-up" (cited in Mahoney et al. 2007).<br><br>Figure 1 in Mahoney et al. 2007 shows that 139 out of 174 participants allocated to intervention and 143 out of 175 allocated to control completed the study
	3.2 If N/PN/NI to 3.1: Is there evidence that result was not biased by missing outcome data?	PY	In the intervention group, 9 patients died and 26 patients withdrew voluntarily before completing the study. In the control group, 8 patients died and 24 patients withdrew voluntarily before completing the study. In total, 20% of intervention group participants and 18% of control group participants did not complete the study. The rate of non-completion did not differ subsantially between intervention groups.
	3.3 If N/PN to 3.2: Could missingness in the outcome depend on its true value?	NA	
	3.4 If Y/PY/NI to 3.3: Is it likely that missingness in the outcome depended on its true value?	NA	
	**Risk of bias judgement**	**Low**	
**Bias in measurement of the outcome**	4.1 Was the method of measuring the outcome inappropriate?	PN	"At the baseline interview, before group assignment, the study researcher gave each participant 12 monthly falls diaries and calendars. Caregivers were asked to help with calendar reporting ... When a fall was reported, the researcher interviewed the subject or caregiver to verify the fall" (cited in Mahoney et al. 2007).
	4.2 Could measurement or ascertainment of the outcome have differed between intervention groups?	PN	The method of ascertaining the outcome seemed standardised and well planned before starting the study.
	4.3 Were outcome assessors aware of the intervention received by study participants?	Y	Yes, as the outcome is falls the outcome assessors were study participants, and study participants were aware of their assigned intervention.
	4.4 If Y/PY/NI to 4.3: Could assessment of the outcome have been influenced by knowledge of intervention received?	PN	Even though the outcome was patient-reported and the patients were aware of the intervention they were assigned to, determining whether or not a fall occured does not involve much judgement. Monthly calendars were provided to help participants document falls continuously.
	4.5 If Y/PY/NI to 4.4: Is it likely that assessment of the outcome was influenced by knowledge of intervention received?	NA	
	**Risk of bias judgement**	**Low**	
**Bias in selection of the reported**	5.1 Were the data that produced this result analysed in accordance with a pre-specified analysis plan that was finalized before unblinded outcome data were available for analysis?	NI	Did not find info
	5.2 ... multiple eligible outcome measurements (e.g. scales, definitions, time points) within the outcome domain?	NI	Did not find info

of the reported result	5.3 ... multiple eligible analyses of the data?	NI	Did not find info
	**Risk of bias judgement**	**Some concerns**	
**Overall bias**	**Risk of bias judgement**	**High**	

**Unique ID**	Peterson(2007)_A	**Study ID**	2667	**Assessor**	RS
**Ref or Label**		**Aim**	assignment to intervention (the 'intention-to-treat' effect)		
**Experimental**	Guided dosing within a computerized provider order entry (CPOE) presented to physicians	**Comparator**	Usual physician practice with no guided dosing presented to physicians	**Source**	Journal article(s)
**Outcome**	Ratio between prescribed medication dose and recommended medication dose during nine months follow-up	**Results**	Median (interquartile range) of 2.5 (1.0, 4.0) in intervention group compared with 3.0 (1.5, 5.0) in control group	**Weight**	1

**Domain**	**Signalling question**	**Response**	**Comments**
**Bias arising from the randomization process**	1.1 Was the allocation sequence random?	NI	
	1.2 Was the allocation sequence concealed until participants were enrolled and assigned to interventions?	NI	
	1.3 Did baseline differences between intervention groups suggest a problem with the randomization process?	NI	
	**Risk of bias judgement**	**Some concerns**	
**Bias due to deviations from intended interventions**	2.1.Were participants aware of their assigned intervention during the trial?	NI	The physicians receiving the guided dosing were aware that they were in a trial.
	2.2.Were carers and people delivering the interventions aware of participants' assigned intervention during the trial?	Y	
	2.3. If Y/PY/NI to 2.1 or 2.2: Were there deviations from the intended intervention that arose because of the experimental context?	PY	"To assess for a crossover effect, where dosing advice on intervention patients influences decisions on control patients, we compared "control-only" physicians and "intervention-only" physicians and a pre-trial period to the trial period" (cited in Peterson et al. 2007).<br><br>"Interventiononly physicians prescribed a significantly lower dose than control-only physicians (median 2.0 [1.0,4.0] vs median 4.0 [2.0,6.0], p<0001). The potential for crossover was also assessed by comparing a pre-trial period of 2 months (2315 medication orders) to the trial period. Pre-trial dosing was significantly higher with median 3.0 (2.0, 6.0) vs. median 3.0 (1.0, 5.0) for the trial period, p<0.001" (cited in Peterson et al. 2007).
	2.4 If Y/PY to 2.3: Were these deviations likely to have affected the outcome?	PY	Based on statistical analyses on prescribing dose among intervention-only physicians compared with control-only physicians it seems that the crossover impacted the outcome in terms of deviating the result towards the dull.
	2.5. If Y/PY/NI to 2.4: Were these deviations from intended intervention balanced between groups?	PN	These deviations were probably not balanced between groups as it is likely that dosing advice given to intervention patients influenced advice given to control patients, but not the other way around.
	2.6 Was an appropriate analysis used to estimate the effect of assignment to intervention?	NI	Unable to find information about this
	2.7 If N/PN/NI to 2.6: Was there potential for a substantial impact (on the result) of the failure to analyse participants in the group to which they were randomized?	NI	
	**Risk of bias judgement**	**High**	
**Bias due to missing outcome data**	3.1 Were data for this outcome available for all, or nearly all, participants randomized?	PY	"The CPOE system recorded all study orders including dosing parameters,<br>ordering provider, and location of patient. Additionally, the system logged whether the physicians viewed the advice for intervention patients. For the analysis, study medication orders entered via order sets or specialized sedation protocols were excluded" (cited in Peterson et al. 2007).
	3.2 If N/PN/NI to 3.1: Is there evidence that result was not biased by missing outcome data?	NA	
	3.3 If N/PN to 3.2: Could missingness in the outcome depend on its true value?	NA	
	3.4 If Y/PY/NI to 3.3: Is it likely that missingness in the outcome depended on its true value?	NA	
	**Risk of bias judgement**	**Low**	
**Bias in measurement of**	4.1 Was the method of measuring the outcome inappropriate?	PN	The hospital had a system in place for recording medication orders.
	4.2 Could measurement or ascertainment of the outcome have differed between intervention groups?	PN	The same CPOE system was used to record outcome data in both study groups.
	4.3 Were outcome assessors aware of the intervention received by study participants?	PY	
	4.4 If Y/PY/NI to 4.3: Could assessment of the outcome have been influenced by knowledge of intervention received?	PN	This is an outcome that reflects decisions made by the intervention provider

**the outcome**	4.5 If Y/PY/NI to 4.4: Is it likely that assessment of the outcome was influenced by knowledge of intervention received?	NA	[illegible] (physicians). There will have been judgement involved in the ordering decisions, but once the ordering decisions were made, there was no interpretation involved in the recording of this decision.
	**Risk of bias judgement**	**Low**	
**Bias in selection of the reported result**	5.1 Were the data that produced this result analysed in accordance with a pre-specified analysis plan that was finalized before unblinded outcome data were available for analysis?	NI	Unable to find an online trial registration, a trial protocol or a statistical analysis plan
	5.2 ... multiple eligible outcome measurements (e.g. scales, definitions, time points) within the outcome domain?	NI	
	5.3 ... multiple eligible analyses of the data?	NI	
	**Risk of bias judgement**	**Some concerns**	
**Overall bias**	**Risk of bias judgement**	**High**	

Risk of bias in results of individually-randomised parallel-group trials

Unique ID	Outcome	D1	D2	D3	D4	D5	Overall
Elley(2008)_A	Rate of falls per person-year at 12 months follow-up in intervention group compared with control group	+	+	+	+	+	+
Ferrer(2014)_A	Risk of falling during 24 months follow-up in intervention group compared with control group	!	+	+	+	!	!
Frankenthal(2014)_A	Number of medications prescribed at 12-month FUNumber of medications prescribed at 12-month FU	+	!	!	+	!	-
Frankenthal(2014)_B	Average number of falls per participant per year during 12 months follow-up	+	!	!	+	!	-
Gallagher, O'Connor, & O'Mahony (2011)_A	Proportion of patients with at least one fall during six months follow-up	+	-	+	+	!	-
Gallagher, O'Connor, & O'Mahony (2011)_B	Unnecessary polypharmacy, use of drugs at incorrect doses, and potential drug-drug and drug-disease interactions (ı	+	-	+	+	!	-
Lightbody (2002)_A	Number of fallers (data from postal questionnaires) during six months follow-up	-	!	+	+	!	-
Lightbody (2002)_B	Number of daily medications at six-month follow-upNumber of daily medications at six-month follow-up	-	!	+	-	!	-
Mahoney(2007)_A	Rate of falls per person-year (365.25 days) during 12 months follow-up in intervention group compared with control	+	-	+	+	!	-
Peterson(2007)_A	Ratio between prescribed medication dose and recommended medication dose during nine months follow-up	!	-	+	+	!	-

+ Low risk

! Some concerns

- High risk

D1 Randomisation process

D2 Deviations from the intended interventions

D3 Missing outcome data

D4 Measurement of the outcome

D5 Selection of the reported result
