# Supplementary material for: Effectiveness of clinical decision support in fall prevention among older adults: A systematic review and meta-analysis
Source: PLoS One. 2026 Jan 12;21(1):e0340025. doi: 10.1371/journal.pone.0340025 (PMC12795367; doi:10.1371/journal.pone.0340025)
Supplement: S8 Table — (DOCX) [file pone.0340025.s008.docx]

**S8 Table. Individual study results**

Aizen et al. (2015): Cluster-randomised trial

| Comparison | Healthcare practitioner performance | Patient outcomes | Other outcomes |
| --- | --- | --- | --- |
| Arm 1 (Intervention: Targeted multiple intervention falls prevention program based on patient's fall risk)  vs  Arm 2 (Control: Usual care) |  | Fall rate per 1000 bed-days in intervention group compared with control group (of participants admitted during the first phase of the study, three months of follow-up): Adjusted hazard ratio 1.36 (95% CI 0.87, 1.77; p = 0.08)  Fall rate per 1000 bed-days in intervention group compared with control group (of participants admitted during the second phase of the study, three months of follow-up): Adjusted hazard ratio 1.27 (95% CI 0.92, 1.67; p = 0.12) |  |

Barker et al. (2016): Cluster-randomised trial

| Comparison | Healthcare practitioner performance | Patient outcomes | Other outcomes |
| --- | --- | --- | --- |
| Arm 1 (Intervention: Nurse-led 6-PACK program for reducing fall-related injuries in acute hospitals)  vs  Arm 2 (Control: Usual fall prevention practices as part of existing hospital policy) | **Secondary outcome:** Rate of use of all 6-PACK programme components (fall risk tool and six interventions) per 1000 occupied bed days during eight months follow-up in intervention group compared with control group: Rate Ratio 3.05 (95 % CI 2.14, 4.34; p < 0.001) | **Primary outcomes:**  Rate of falls per 1000 occupied bed days during 12 months follow-up in intervention group compared with control group: Rate Ratio 1.04 (95% CI 0.78, 1.37; p = 0.796)  Rate of fall injuries per 1000 occupied bed days during 12 months follow-up in intervention group compared with control group: Rate Ratio 0.96 (95% CI 0.72, 1.27; p = 0.766) |  |

Bhasin et al. (2020): Cluster-randomised trial

| Comparison | Healthcare practitioner performance | Patient outcomes | Other outcomes |
| --- | --- | --- | --- |
| Arm 1 (Intervention: The Strategies to Reduce Injuries and Develop Confidence in Elders (STRIDE) intervention)  vs  Arm 2 (Control: Enhanced usual care) |  | **Primary outcome:** Rate of time to first adjudicated serious fall injury per 100 person-years of follow-up during 44 months follow-up in intervention group compared with control group: Hazard Ratio 0.92 (95% CI 0.80, 1.06; p = 0.25)  **Secondary outcome:** Rate of time to first participant-reported fall injury per 100 person-years of follow-up during 44 months follow-up in intervention group compared with control group: Hazard Ratio 0.90 (95% CI 0.83, 0.99, p = 0.004) | Serious adverse events: Rate of deaths from serious adverse events per 100 person-years of follow-up during 44 months follow-up in intervention group compared with control group: Hazard Ratio 1.01 (95% CI 0.84, 1.23; p = 0.88)  Serious adverse events: Rate of hospitalisations from serious adverse events per 100 person-years of follow-up during 44 months follow-up in intervention group compared with control group: Hazard Ratio 0.98 (95% CI 0.92, 1.04; p = 0.47) |

Blalock et al. (2020): Cluster-randomised trial

| Comparison | Healthcare practitioner performance | Patient outcomes | Other outcomes |
| --- | --- | --- | --- |
| Arm 1 (Intervention: STEADI-Rx)  vs  Arm 2 (Control: No-intervention control)  Note: No primary outcomes mentioned by authors. |  | Change in use of fall-risk increasing drugs (Drug Burden Index score) from 12-month pre-intervention period to 12-month post-intervention period (all participants) in intervention group compared with control group: No difference in mean DBI score (p = 0.66)  Change in use of fall risk-increasing drugs (Drug Burden Index score) from 12-month pre-intervention period to 12-month post-intervention period (among participants who screened positive for fall risk): Mean difference – 0.12 (Standard Error 0.02) in intervention group compared with – 0.08 (Standard Error – 0.01) in control group (p = 0.05)  Risk of falling (use of medications associated with an increased fall risk measured with the Drug Burden Index) during 12-month post-intervention period, controlled for risk of falling during 12-month pre-intervention period, in intervention group compared with control group: Odds Ratio 1.09 (95% CI 0.81, 1.47; p = 0.58) |  |

Blum et al. (2021): Cluster-randomised trial

| Comparison | Healthcare practitioner performance | Patient outcomes | Other outcomes |
| --- | --- | --- | --- |
| Arm 1 (Structured pharmacotherapy optimisation intervention supported by a software-based clinical decision support tool)  vs  Arm 2 (Usual care including unstructured medication review unsupported by STOPP/START criteria or the systematic tool to reduce inappropriate prescribing (STRIP) tool) | **Secondary outcomes:**  Presence of drug overuse (based on STOPP criteria) during two months follow-up in intervention group compared with control group: Odds ratio 0.99 (95% CI 0.82, 1.20; p = 0.91)  Presence of drug misuse (based on STOPP criteria) during two months follow-up in intervention group compared with control group: Odds ratio 0.99 (95% CI 0.81, 1.20; p = 0.92)  Presence of drug underuse (based on START criteria) during two months follow-up in intervention group compared with control group: Odds ratio 0.91 (95% CI 0.70, 1.17; p = 0.45)  Presence of clinically significant drug-drug interactions during two months follow-up in intervention group compared with control group: Odds ratio 0.87 (95% CI 0.67, 1.14, p = 0.31) | **Primary outcome:**  Rate of time to first confirmed drug-related hospital admission after discharge following the index hospital admission during 12 months follow-up in intervention group compared with control group: HR 0.95 (95% CI 0.77, 1.17; p = 0.62)  **Secondary outcomes:**  Rate of time to first fall during 12 months follow-up in intervention group compared with control group: Hazard ratio 0.96 (95% CI 0.79, 1.15; p = 0.64)  Number of long-term prescription drugs at 12-month follow-up in intervention group compared with control group: Adjusted mean difference -0.20 (95% CI -0.56, 0.17; p = 0.29)  Drug compliance (MMAS-8) at 12-month follow-up in intervention group compared with control group: Adjusted mean difference 0.03 (95% CI -0.05, 0.12; p = 0.46) | **Secondary outcomes:**  All-cause mortality during 12 months follow-up in intervention group compared with control group: Hazard Ratio 0.90 (95% CI 0.71, 1.13, p = 0.37)  Rate of time to first hospital admission during 12 months follow-up in intervention group compared with control group: Hazard Ratio 0.87 (95% CI 0.75, 1.01; p = 0.08) |

Byrne, M. (2005): Controlled before-after study

| Comparison | Healthcare practitioner performance | Patient outcomes | Other outcomes |
| --- | --- | --- | --- |
| Arm 1 (Intervention: Application and integration of computerized resident risk information decision tools into care processes)  vs  Arm 2 (Control: Usual care) |  | Rate of falls during nine months pre-intervention compared with nine months post-intervention: Pre-intervention fall rate of 0.312 in intervention group and 0.315 in control group compared with post-intervention fall rate of 0.318 in intervention group and 0.290 in control group. Percentage change in fall rate from pre-intervention to post-intervention: 0.6% in intervention group compared with -2.5 % in control group; risk difference 3.1%  Details of result:   - The intervention group consisted of nursing home group 1 (high access and high integration with care planning) and group 2 (moderate to high access and some integration with care planning) - The matched control group consisted of a matched sample of nursing homes that volunteered to participate in the project but were not selected (Group 5) or that did not volunteer (Group 6) since Group 5 did not yield high enough matches to use that group only. - Pre-intervention period: First three quarters (January – September) of 2002 - Post-intervention period: First three quarters (January – September) of 2004 |  |

Carroll, Dykes, & Hurley (2012): Cluster-randomised trial

| Comparison | Healthcare practitioner performance | Patient outcomes | Other outcomes |
| --- | --- | --- | --- |
| Arm 1 (Intervention: Fall Prevention Tool Kit (FPTK) using health information technology (HIT))  vs  Arm 2 (Control: Usual care related to fall prevention, i.e. no intervention)  Note: No primary outcome specified | Proportion of patient records reviewed, and with identified risks on the Morse Falls Scale, that had fall risk documented on the plan of care during six months follow-up: 89% in intervention group compared with 64% in control group (p < 0.0001) |  |  |

Clemson et al. (2024). Cluster-randomised trial

| Comparison | Healthcare practitioner performance | Patient outcomes | Other outcomes |
| --- | --- | --- | --- |
| Arm 1 (Intervention: The Integrated Solutions for Sustainable Fall Prevention (iSOLVE) intervention)  vs  Arm 2 (Control: Usual general practice care) | Changes in GPs' engagement in fall prevention activities, including risk assessment, medication reviews, and providing advice, compared to the control group, at three months follow-up: 0.90 units (95% CI 0.33, 1.46) | Rate of falls, self-reported by patients using daily fall calendar mailed on a monthly basis, in intervention group compared with control group over 12 months follow-up: Incidence rate ratio 0.96 (95% CI 0.77 - 1.20) |  |

Dykes et al. (2010): Cluster-randomised trial

| Comparison | Healthcare practitioner performance | Patient outcomes | Other outcomes |
| --- | --- | --- | --- |
| Arm 1 (Intervention: Fall Prevention Tool Kit (FPTK) using health information technology (HIT))  vs  Arm 2 (Control: Usual care related to fall prevention, i.e. no intervention) | Adherence to intervention protocol during six months follow-up (Morse Falls Scale completion): 94% in intervention units compared with 81% in control units | **Primary outcomes:**  Rate of patient falls per 1000 patient-days during six months follow-up in control group compared with intervention group (sensitivity analysis with patients aged 65 years or older): Rate difference 2.08 (95% CI 0.61, 3.56; p = 0.003)  Number of fall-related injuries per 1000 patient-days during 6 months follow-up (sensitivity analysis with patients aged 65 years or older): 7 in intervention group compared with 9 in the control group (p = 0.66) |  |

Dykes et al. (2020): Non-randomised controlled trial

| Comparison | Healthcare practitioner performance | Patient outcomes | Other outcomes |
| --- | --- | --- | --- |
| Arm 1 (Intervention: Patient-centered fal prevention toolkit (FPTK) that actively engages patients and family in the three-step fall prevention process)  vs  Arm 2 (Control: Usual care as it relates to fall prevention) |  | **Primary outcome:** Rate of patient falls per 1000 patient-days in 21-month post-intervention period compared with 21-month pre-intervention period (subgroup analysis of patients aged 65 years or older): Rate ratio 0.90 (95% CI 0.74, 1.09; p = 0.28)  **Secondary outcome:** Rate of patient falls with injury per 1000 patient-days in 21-month post-intervention period compared with 21-month pre-intervention period (subgroup analysis of patients aged 65 years or older): Rate ratio 0.52 (95% CI 0.34, 0.82; p = 0.004) |  |

Elley et al. (2008): Individually-randomised parallel-group trial

| Comparison | Healthcare practitioner performance | Patient outcomes | Other outcomes |
| --- | --- | --- | --- |
| Arm 1 (Intervention: Falls-and-fracture nurse (FFN) coordinator and multifactorial intervention)  vs  Arm 2 (Control: Usual care plus an offer of two social visits) |  | Rate of falls per person-year during 12 months follow-up in intervention group compared with control group: Rate Ratio 0.96 (95% CI 0.70, 1.34) |  |

Ferrer et al. (2014): Individually-randomised parallel-group trial

| Comparison | Healthcare practitioner performance | Patient outcomes | Other outcomes |
| --- | --- | --- | --- |
| Arm 1 (Intervention: Multifactorial fall risk assessment and treatment recommendations made to patient and family physician based on algorithm)  vs  Arm 2 (Control: Usual health care) |  | **Primary outcomes:**  Risk of falling during 24 months of follow-up in intervention group compared with control group: Relative Risk 1.28 (95% CI 0.94, 1.75)  Time until first fall during 24 months follow-up in intervention group compared with control group (Model 3): Hazard ratio 1.40 (95% CI 0.93, 2.10; p = 0.138)  Time until second fall during 24 months follow-up in intervention group compared with control group: No statistically significant differences between groups (p = 0.062)  Time until recurrent falls during 24 months follow-up in intervention group compared with control group (Model 3): Hazard Ratio 1.46 (95% CI 1.03, 2.09)  Fall rate during 12 months follow-up in intervention group compared with control group: Incidence rate 0.85 (95% CI 0.51, 1.40; p = 0.56)  Fall rate during 12 to 24 months follow-up in intervention group compared with control group: Incidence rate 2.06 (95% CI 1.22, 3.5; p = 0.08)  **Secondary outcome:** Mean number of fractures per participant at 24 months follow-up in control group compared with intervention group: Mean difference -0.03 (95% CI -0.09, – 0.02) | **Adverse events:** Mean number of hospitalisations per participant at 24 months follow-up in control group compared with intervention group: Mean difference -0.03 (95% CI -0.15, 0.10) |

Frankenthal et al. (2014): Individually-randomised parallel-group trial

| Comparison | Healthcare practitioner performance | Patient outcomes | Other outcomes |
| --- | --- | --- | --- |
| Arm 1 (Intervention: Screening medications with STOPP/START criteria followed up with recommendations to the chief physician)  vs  Arm 2 (Control: Usual pharmaceutical care) | **Potentially inappropriate prescriptions**  Proportion of participants with Potentially Inappropriate Prescriptions (PIPs) at six-month follow-up: 37.4% in intervention group compared with 56.0% in control group (p = 0.001)  Proportion of participants with PIPs at 12-month follow-up: 22.5% in intervention group compared with 54.1% in control group (p < 0.001)  **Potential prescription omissions**  Proportion of participants with Potential Prescription Omissions (PPOs) at six-month follow-up: 9.2% in intervention group compared with 25.2% of participants in control group (p < 0.001)  Proportion of participants with PPOs at 12-month follow-up: 6.3% in intervention group compared with 21.9% in control group (p < 0.001)  **Number of medications**  Number of medications prescribed at six-month follow-up: Mean (SD) of 8.1 (3.2) in intervention group compared with 9.0 (3.3) in control group (p = 0.2)  Number of medications prescribed at 12-month follow-up: Mean (SD) of 7.3 (2.7) in intervention group compared with 8.9 (3.2) in control group (p < 0.001) | Average number of falls per participant per year during 12 months follow-up: Mean (SD) of 0.8 (1.3) in intervention group compared with 1.3 (2.4) in control group (p = 0.28) | Average costs of medications per month (in Israeli shekels) at 12-month follow-up: Mean (SD) 279 (171.9) in intervention group compared with 402.3 (291.2) in control group (< 0.001)  Average number of hospitalisations per participant per year during 12 months follow-up: Mean (SD) of 0.5 (1.0) in intervention group compared with 0.5 (0.9) in control group (p = 0.10) |

Gallagher, O’Connor & O’Mahony (2011): Individually-randomised parallel-group trial

| Comparison | Healthcare practitioner performance | Patient outcomes | Other outcomes |
| --- | --- | --- | --- |
| Arm 1 (Intervention: Screening with STOPP/START criteria followed up with recommendations to patients’ attending physicians)  vs  Arm 2 (Control: Usual pharmaceutical care) | **Primary outcomes:**  Unnecessary polypharmacy, use of drugs at incorrect doses, and potential drug-drug and drug-disease interactions (patients with improvement in Medication Appropriateness Index (MAI) scores) at discharge: 135 (71.1%) in intervention group and 68 (35.4%) in control group, absolute risk reduction 35.7% (95% CI 26.3%, 44.9%); number needed to screen to yield improvement in MAI = 2.8 (95% CI 2.2, 3.8)  Number needed to screen using STOPP/START criteria to yield an improvement in the Medication Appropriateness Index at hospital discharge was 2.8 (95% CI 2.2, 3.8)  Frequency of unnecessary polypharmacy among patients (absence of indication, lack of efficacy, or therapeutic duplication) at discharge: 5.4% (n = 80) in the intervention group and 19.8% (n = 306) in the control group (X2 = 132.634, P < 0.001)  Underutilization of clinically indicated medications (patients with improvement in Assessment of Underutilization (AOU) index) at discharge: 60 (31.6%) in intervention group compared with 20 (10.4%) in control group, absolute risk reduction 21.2% (95% CI 13.3%, 29.1%); number needed to screen to yield reduction in AOU = 4.7 (95% CI 3.4, 7.5) | **Secondary outcome:** Proportion of patients with at least one fall during six months follow-up: 5.8% of participants in intervention group compared with 8.4% of patients in control group (p = 0.332) | **Secondary outcomes:** Frequency of hospital readmissions during six months follow-up: 67 readmission in the intervention group compared with 64 readmissions in the control group (p = 0.691)  Median length of hospital stay during six months follow-up: median (interquartile range) 8 (5 – 14) in the intervention group compared with 8.5 (5 – 15.75) in the control group (p = 0.471)  **Resource use:** Frequency of general practitioner visits during six months follow-up: There was a trend toward a lower frequency in the intervention group compared with the control group (p = 0.063) |

Ganz et al. (2015): Non-randomised controlled trial

| Comparison | Healthcare practitioner performance | Patient outcomes | Other outcomes |
| --- | --- | --- | --- |
| Arm 1 (Intervention: Assessing Care of Vulnerable Elders Practice Redesign for Improved Medical Care for Elders (ACOVEprime))  vs  Arm 2 (Control: Usual care) |  | **Primary outcome:**  Rate of episodes of care for injuries due to falls per 1000 person-years during 12 months follow-up in intervention group compared with control group: Rate Ratio 1.27 (95% CI 0.93, 1.73)  **Secondary outcome:**  Rate of episodes of care for injuries due to falls per 1000 person-years during 24 months follow-up in intervention group compared with control group: Rate Ratio 1.18 (95% CI 0.93, 1.49) |  |

Ganz et al. (2022): Cluster-randomised trial

| Comparison | Healthcare practitioner performance | Patient outcomes | Other outcomes |
| --- | --- | --- | --- |
| Arm 1 (Intervention: Strategies to Reduce Injuries and Develop Confidence in Elders (STRIDE))  vs  Arm 2 (Control: Enhanced usual care) |  | Risk of falling during 24 months follow-up: 65.4% in intervention group compared with 67.9% in control group (not statistically significant)  Rate of falls per 100 person-years during 24 months follow-up in intervention group compared with control group: Rate ratio 0.97 (95% CI 0.93, 1.00; p = 0.048)  Rate of falls leading to hospital admission per 100 person-years during 24 months follow-up in intervention group compared with control group: Rate ratio 0.91 (95% CI 0.77, 1.07; p = 0.263)  Rate of falls leading to medical attention per 100 person-years during 24 months follow-up in intervention group compared with control group: Rate ratio 0.97 (95 CI 0.89, 1.06; p = 0.477)  Rate of self-reported fractures per 100 person-years during 24 months follow-up in intervention group compared with control group: Rate ratio 0.93 (95% CI 0.80, 1.08; p = 0.337)  Rate of adjudicated fractures per 100 person-years during 27 months follow-up in intervention group compared with control group: Rate ratio 0.89 (95% CI 0.73, 1.07; p = 0.205) |  |

Groshaus et al. (2012); Non-randomised controlled trial with a stepped wedge design

| Comparison | Healthcare practitioner performance | Patient outcomes | Other outcomes |
| --- | --- | --- | --- |
| Before the intervention (control) was implemented  vs  After the intervention (multi-component knowledge translation intervention that incorporated a nurse-initiated decision support tool) was implemented | **Primary outcome:**  Mean rate of use of the 'order set' (the number of patients 65 years or older with the order set on their electronic medical record compared to the total number of patients 65 years or older on the units at the time of data collection) over a 2-week period after the intervention was implemented compared with before the intervention was implemented, over a three-month study duration: Mean difference 3.1 (95% CI 1.9, 5.3)  Number of consults ordered for each of orthopedics, geriatrics, psychiatry, and physiotherapy over a 2-week period: 278 total consults before the intervention was implemented compared with 262 total consults after the intervention was implemented (p < 0.2) | **Secondary outcome:** Odds of a fall happening on a unit over a 2-week period before the intervention was implemented compared with after the intervention was implemented, over a three-month study duration: Odds ratio 9.3 (95% CI 0.9, 100; p = 0.065) | **Secondary outcome:** Median number of days in hospital after the intervention was implemented compared with before the intervention was implemented, over a three-month study duration: Median of 45 days after the intervention was implemented compared with a median of 42 days before the intervention was implemented (p = 0.67) |

Healey et al. (2004): Cluster-randomised trial

| Comparison | Healthcare practitioner performance | Patient outcomes | Other outcomes |
| --- | --- | --- | --- |
| Arm 1 (Intervention: Targeted risk factor reduction core care plan)  vs  Arm 2 (Control: No change in practice or environment relevant to fall prevention)  Note: In this study, risk of falling refers to fall rate per 1000 occupied bed days: “The term ‘relative risk’ is more commonly used in describing the risk to an individual: it is important to clarify that in the context of this study the term was used in relation to the rate of falling between control and intervention wards. The same comparisons were also made for the secondary outcome of injuries from falls” (cited in journal article). |  | Risk of falling in intervention group compared with control group as a change from the six-month period before intervention implementation to the six-month period after intervention implementation: Relative risk 0.71 (95% CI 0.55, 0.90; p = 0.006)  Risk of having a fall-related injury in the intervention group compared with the control group as a change from the six-month period before intervention implementation to the six-month period after intervention implementation: Relative risk 1.35 (95% CI 0.80, 2.28; p = 0.26) |  |

Lightbody et al. (2002): Individually-randomised parallel-group trial

| Comparison | Healthcare practitioner performance | Patient outcomes | Other outcomes |
| --- | --- | --- | --- |
| Arm 1 (Intervention: Nurse-led fall prevention management plan and care pathway for older people)  vs  Arm 2 (Control: Usual care)  Note: All outcomes are defined by authors as primary | **Medications:**  Number of daily medications at six-month follow-up: 608 in intervention group compared with 684 in control group (p = 0.41)  Number of patients on more than three medications at six-month follow-up: 79 (49%) in intervention group compared with 94 (57%) in control group (p = 0.21)  Number of patients on target medications at six-month follow-up: 130 (81%) in intervention group compared with 132 (80%) in control group (p = 0.88) | Number of fallers (data from postal questionnaires) during six months follow-up: 36 (23%) in the intervention group compared with 39 (25%) in the control group (p = 0.89)  Number of falls (data from postal questionnaire) during six months follow-up: 89 in intervention group compared with 145 in the control group (p = 0.65) | **Resource use:**  Number of re-attendances in the Accident and Emergency Department (data from hospital database) during six months follow-up: 43 in the intervention group compared with 58 in the control group (p = 0.82)  ---  Number of fall-related general practitioner attendances (data from GP questionnaire) during six months follow-up: 91 in the intervention group compared with 67 in the control group (p = 0.14)  Number of fall-related hospital admissions (data from hospital database) during six months follow-up: 8 in intervention group compared with 10 in control group (p = 0.87)  Number of bed-days (data from hospital database) during six months follow-up: 470 in intervention group compared with 590 in control group (p = 0.91)  Number of bed-days with a fall or fall-related problem (data from hospital database) during six months follow-up: 69 in intervention group compared with 233 in control group (p = 0.56) |

Logan et al (2021): Cluster-randomised trial

| Comparison | Healthcare practitioner performance | Patient outcomes | Other outcomes |
| --- | --- | --- | --- |
| Arm 1 (Intervention: The Guide to Action Care Homes (GtACH) Falls Prevention Programme)  vs  Arm 2 (Control: Usual care defined as the absence of a systematic and coordinated falls prevention process) |  | **Primary outcome**: Fall rate per 1000 resident-days between 91 and 180 days after randomisation in intervention group compared with control group (minimally adjusted): Rate ratio 0.57 (95% CI 0.45, 0.71; p<0.001)  ---  Fall rate per 1000 resident-days between 1 and 90 days after randomisation in intervention group compared with control group (minimally adjusted): Rate ratio 0.60 (95% CI 0.49, 0.73; p<0.001)  Fall rate per 1000 resident-days between 181 and 270 days after randomisation in intervention group compared with control group (minimally adjusted): Rate ratio 0.85 (95% CI 0.69, 1.05; p = 0.13)  Fall rate per 1000 resident-days between 271 and 360 days after randomisation in intervention group compared with control group (minimally adjusted): Rate ratio 0.79 (95% CI 0.60, 1.03; p = 0.08)  Frequency of fractures between 0 and 180 days after randomisation in intervention group compared with control group (minimally adjusted): Odds ratio 1.19 (95% CI 0.70, 2.01; p = 0.53)  Frequency of fractures between 181 and 360 days after randomisation in intervention group compared with control group (minimally adjusted): Odds ratio 0.34 (95% CI 0.15, 0.75; p = 0.007) | **Economic evaluation:**  Incremental costs per quality-adjusted life-year (EQ-5D-5L-P-based QALY) at 12-month follow-up for the GtACH programme compared with control: £4544  Incremental cost per health-related quality of life (DEMQOL-P-U-based QALY) at 12-month follow-up for the GtACH programme compared with control: £20 889  Cost per fall averted at 12-month follow-up for the GtACH programme compared with control: £191  Deaths during 12 months follow-up in intervention group compared with control group (minimally adjusted): Odds ratio 0.93 (95% CI 0.73, 1.20; p = 0.58) |

Mahoney et al. (2007): Individually-randomised parallel-group trial

| Comparison | Healthcare practitioner performance | Patient outcomes | Other outcomes |
| --- | --- | --- | --- |
| Arm 1 (Intervention: Intermediate-intensity, community-based multifactorial falls risk assessment and recommendations to primary physician)  vs  Arm 2 (Control: Home safety visits) |  | Rate of falls per person-year (365.25 days) during 12 months follow-up in intervention group compared with control group: Rate Ratio 0.81 (95% CI 0.57, 1.17; p = 0.27) | Rate of all-cause hospitalisations per person-year (365.25 days) during 12 months of follow-up in intervention group compared with control group: Rate Ratio 1.05 (95% CI 0.71, 1.55; p = 0.82)  Rate of all-cause nursing home admissions per person-year (365.25 days) during 12 months of follow-up in intervention group compared with control group: Rate Ratio 0.72 (95% CI 0.38, 1.35; p = 0.30) |

Peterson et al. (2007): Individually-randomised parallel-group trial

| Comparison | Healthcare practitioner performance | Patient outcomes | Other outcomes |
| --- | --- | --- | --- |
| Arm 1 (Intervention: Guided dosing within a computerized provider order entry (CPOE) presented to physicians)  vs  Arm 2 (Control: Usual physician practice with no guided dosing presented to physicians) | **Primary outcome:** Ratio between prescribed medication dose and recommended medication dose during nine months follow-up: Median (interquartile range) of 2.5 (1.0, 4.0) in intervention group compared with 3.0 (1.5, 5.0) in control group (p < 0.001)  ---  Proportion of physicians who chose recommended doses during nine months follow-up: 28.6% in intervention group compared with 24.1% in control group (p < 0.001). |  |  |

Phelan et al. (2024). Cluster-randomised trial

| Comparison | Healthcare practitioner performance | Patient outcomes | Other outcomes |
| --- | --- | --- | --- |
| Arm 1 (Intervention: The STOPP-FALLS intervention: Patient education and clinician decision support)  vs  Arm 2 (Control: Usual care) | Discontinuation of medications (defined as no prescription fill for 90 days), summarized across all target medication classes, referred to as “first target medication”, at six months follow-up in intervention group compared with control group: Adjusted relative risk 1.24 (95% CI 0.90, 1.70) | Time to first medically treated fall in intervention group compared with control group, at 18 months follow-up: Hazard ratio 1.11 (95% CI 0.94, 1.31; p=0.11)  Note: This result was not included in the meta-analysis on the rate of fall injuries because the risk and not the rate was reported. |  |

Snooks et al. (2014): Cluster-randomised trial

| Comparison | Healthcare practitioner performance | Patient outcomes | Other outcomes |
| --- | --- | --- | --- |
| Arm 1 (Intervention: Computerised Clinical Decision Support for paramedics attending older people who fall)  vs  Arm 2 (Control: Usual care with paper-based protocols to assess patients and make decisions about their care instead of computerised clinical decision support) | **Primary outcomes:**  Odds of a patient being referred to a falls service during one month follow-up in intervention group compared with control group: Odds Ratio 2.036 (95% CI 1.115, 3.717; p = 0.021)  Odds of a patient being left at scene without conveyance to an emergency department during one month follow-up in intervention group compared with control group: Odds Ratio 1.131 (95% CI 0.839, 1.523; p = 0.419) | **Secondary outcome:**  Odds of a patient reporting ≥1 falls (self-report) during one month follow-up in intervention group compared with control group: Odds Ratio 0.752 (95% CI 0.503, 1.124; p = 0.165) | **Primary outcomes:**  Odds of a patient dying during one month follow-up in intervention group compared with control group: Odds Ratio 1.375 (95% CI 0.645, 2.930)  Patients with further emergency admission to hospital or death during one month follow-up: Odds Ratio 1.129 (95% CI 0.757, 1.685)  Patients with further emergency (ED attendance or emergency admission to hospital) or death: Odds Ratio 1.296 (95% CI 0.899, 1.870)  Odds of a patient experiencing an adverse event (either 999 call, emergency department attendance, emergency admission to hospital, or death) during one month follow-up in intervention group compared with control group: Odds Ratio 1.266 (95% CI 0.931, 1.720; p = 0.132)  Cost (in GBP) per patient of implementation and use of CCDS (intervention group) compared with cost of usual care with paper-based protocols (control group) during one month follow-up: Mean difference 247 (95% CI -247, 741) |

Tamblyn et al. (2012): Cluster-randomised trial

| Comparison | Healthcare practitioner performance | Patient outcomes | Other outcomes |
| --- | --- | --- | --- |
| Arm 1 (Intervention: Computerized prescribing decision support presenting patient-specific risk of psychotropic drug-related injury to family physicians)  vs  Arm 2 (Control: Access to standard features of the MOXXI clinical information system without patient-specific risk of psychotropic drug-related injury presented to physicians) |  | **Primary outcome:** Mean reduction in risk of injury (based on psychotropic medications and non-modifiable risk factors) per 1000 patients at 23-month follow-up in intervention group compared with control group: Mean difference 1.7 (95% CI 0.2, 3.2; p = 0.02) |  |

Weber, White & McIlvried (2008): Cluster-randomised trial

| Comparison | Healthcare practitioner performance | Patient outcomes | Other outcomes |
| --- | --- | --- | --- |
| Arm 1 (Intervention: Standardized medication review with recommendations to primary care physician via the electronic medical record)  vs  Arm 2 (Control: Usual care without medication review and recommendations sent to primary care physician)  Note: No primary outcome specified | Change in the number of active medications during 12 months follow-up in intervention group compared with control group (difference-in-differences): Mean difference -0.496 (p = 0.0875)  Change in the number of medications started during 12 months follow-up in intervention group compared with control group: Mean difference -0.199 (p < 0.01)  Change in the number of psychotropic medications during 12 months follow-up in intervention group compared with control group: Mean difference -0.204 (p < 0.05) | Odds of having had one or more fall-related medical encounters (as determined by EpicCare data only) during 15 months follow-up in intervention group compared with control group: Odds Ratio 0.38 (p < 0.01)  Odds of having experienced one or more falls or falls requiring medical attention (as determined by self-report and EpicCare data) during 15 months follow-up in intervention group compared with control group: Odds Ratio 0.86 (> 0.1) |  |

Wenger et al. (2009): Non-randomised controlled trial

| Comparison | Healthcare practitioner performance | Patient outcomes | Other outcomes |
| --- | --- | --- | --- |
| Arm 1 (Assessing Care of Vulnerable Elders 2 (ACOVE-2))  vs  Arm 2 (Control: Usual care) | Proportion of recommended care (quality indicators) for falls provided to patients during 13 months follow-up: 44% (95% CI 36%, 51%) in intervention group compared with 23% (95% CI 14% - 30%) in control group (p < 0.001) |  | Physicians’ knowledge about falls, incontinence, and cognitive impairment during 13 months follow-up: Mean change 1.9 in intervention group compared with 0.2 in control group (p = 0.05)  Physicians’ confidence concerning management of falls, incontinence, and cognitive impairment during 13 months follow-up: Mean change 0.64 in intervention group compared with 0.10 in control group (p < 0.05) |

Wenger et al. (2010): Non-randomised controlled trial

| Comparison | Healthcare practitioner performance | Patient outcomes | Other outcomes |
| --- | --- | --- | --- |
| Arm 1 (Intervention: Assessing Care of Vulnerable Elders Practice Redesign for Improved Medical Care for Elders (ACOVEprime))  vs  Arm 2 (Control: Usual care) | Proportion of recommended care (quality indicators) for falls provided to patients during 12 months follow-up: 60% of quality indicators provided in intervention group compared with 37.6% in control group (p < 0.001) |  | Mean change in healthcare practitioners' knowledge about falls and UI (0- to 100-point knowledge score change) during 12 months follow-up in intervention group compared with control group: Mean increase 14.8 points in intervention group compared with 3.2 points in control group (p = 0.007) |
