# Supplementary material for: Effectiveness of clinical decision support in fall prevention among older adults: A systematic review and meta-analysis
Source: PLoS One. 2026 Jan 12;21(1):e0340025. doi: 10.1371/journal.pone.0340025 (PMC12795367; doi:10.1371/journal.pone.0340025)
Supplement: S9 Table — (DOCX) [file pone.0340025.s009.docx]

**S9 Table. GRADE evidence profile**

**Author(s):** Rune Solli, Signe Flottorp
**Date:** 20.09.25
**Question:** Should clinical decision support targeted at health professionals be used to prevent falls among older adults?
**Settings:** Hospitals, residential care, primary care, the homes of older adults **Bibliography:** Solli et al: Effectiveness of clinical decision support in fall prevention among older adults: a systematic review and meta-analysis

| **Certainty assessment** | | | | | | | **Summary of findings** | | | | |
| --- | --- | --- | --- | --- | --- | --- | --- | --- | --- | --- | --- |
|  |  |  |  |  |  |  | **No of patients** | | **Effect** | | **Certainty** |
| **No of studies** | **Design** | **Limitations (risk of bias)** | **Inconsistency** | **Indirectness** | **Imprecision** | **Other considerations** | **CDS** | **Usual practice** | **Relative (95% CI)** | **Absolute effect (95% CI)*** |  |
| **Adherence to recommended practice: fall risk assessments and interventions** median (min–max) follow-up time was 7 (1–13) months. | | | | | | | | | | | |
| 5  3 | randomised trials  non- randomised trials | Very serious risk of bias due to confounding, deviations from intended interventions, and selection of reported results | No serious inconsistency; all results showed the same direction of effect. Effect size not considered in vote-counting. | No serious indirectness | No serious imprecision | None | Unavailable^(1)^ | Unavailable^(1)^ | NA | NA | ⊕⊕OO LOW  Due to very serious risk of bias |
| **Adherence to recommended medication review and prescribing** median (min–max) follow-up time was 9 (0–23) months | | | | | | | | | | | |
| 9 | randomised trials | Risk of bias due to deviations from intended interventions. | No serious inconsistency; all results showed the same direction of effect. Effect size not considered in vote-counting. | No serious indirectness | No serious imprecision | None | Unavailable^(1)^ | Unavailable^(1)^ | NA | NA | ⊕⊕⊕O MODERATE  Due to risk of bias |
| **Fall risk** median (min–max) follow-up time of 9 (1–24) months | | | | | | | | | | | |
| 9  1 | randomised trials  non-randomised trial | Risk of bias due to the randomisation process, deviations from intended interventions, and measurement of the outcome | No serious inconsistency | No serious indirectness | Imprecision due to the upper bound of the 95% confidence interval crossing the null effect | None | > 6 806^(2)^ | > 6 830^(2)^ | Odds ratio 0.93 (0.85, 1.01) | 14 fewer per 1 000 persons (32 fewer to 3 more) | ⊕⊕OO LOW  Due to risk of bias and imprecision |
| **Rate of falls in hospitals or residential care** median (min–max) follow-up time was 6 (3–21) months | | | | | | | | | | | |
| 7  1 | randomised trials  non-randomised trial | Risk of bias in the randomisation process and due to deviations from intended interventions | No serious inconsistency_(3)_ | No serious indirectness | No serious imprecision | None | > 25 092^(2)^ | > 24 962^(2)^ | Rate ratio 0.74 (0.63, 0.88) | 175 fewer per 1 000 person-years (249 fewer to 81 fewer) | ⊕⊕⊕O MODERATE  Due to risk of bias |
| **Rate of falls (in studies conducted among community-dwelling older adults)** median (min–max) follow-up time was 12 (12–24) months | | | | | | | | | | | |
| 5 | randomised trials | Risk of bias due to deviations from intended interventions | No serious inconsistency_(3)_ | No serious indirectness | Imprecision due to the upper bound of the 95% confidence interval including the null effect | None | 3 570 | 3 430 | Rate ratio 0.97 (0.93, 1.00) | 20 fewer per 1 000 person-years (47 fewer to 0 fewer) | ⊕⊕OO LOW  Due to risk of bias and imprecision |
| **Rate of falls (in studies conducted among patients with a mean age of ≥ 80 years)** median (min–max) follow-up time was 12 (3–12) months | | | | | | | | | | | |
| 7 | randomised trials | Risk of bias due to deviations from intended interventions and selection of reported results | No serious inconsistency_(3)_ | No serious indirectness | No serious imprecision | None | 200 + 155 + 164 + 160 + 1 525 + 502 + 174 = 2 880 | 308 + 157 + 164 + 146 + 1 861 + 573 + 175 = 3 384 | Rate ratio 0.72 (0.61, 0.86) | 188 fewer per 1 000 person-years (262 fewer to 94 fewer) | ⊕⊕⊕O MODERATE  Due to risk of bias |
| **Rate of falls (in studies conducted among patients with a mean age between 65 and 80 years)** median (min–max) follow-up time was 12 (6–24) months | | | | | | | | | | | |
| 5  1 | randomised trials  non-randomised trials | Risk of bias due to the randomisation process, confounding, and deviations from intended interventions | No serious inconsistency_(3)_ | No serious indirectness | Imprecision due to the upper bound of the 95% confidence interval crossing the null effect | None | 17698 + 275 + 2755 + 19 283 + 2 802 + 155 = 42 968 | 17566 + 285 + 2509 + 17 948 + 2 649 + 159 = 41 116 | Rate ratio 0.92 (0.84, 1.01) | 54 fewer per 1 000 person-years (108 fewer to 7 more) | ⊕⊕OO LOW  Due to risk of bias and imprecision |
| **Rate of fall injuries (in patients with mean age between 65 and 80 years old)** median (min-max) follow up 9 (6–12) months | | | | | | | | | | | |
| 3  1 | randomised trials  non-randomised trial | Risk of bias due to confounding deviations from intended interventions | No serious inconsistency_(3)_ | No serious indirectness | Imprecision due to the upper bound of the 95% confidence interval crossing the null effect | None | > 25 660^(2)^ | > 25 319^(2)^ | Rate ratio 0.80 (0.59, 1.09) | 33 fewer per 1 000 person-years (67 fewer to 15 more) | ⊕⊕OO LOW  Due to risk of bias and imprecision |
| **Rate of fall injuries (in patients with mean age 80 years and older)** median (min–max) follow-up time was 16.5 (6–24) months | | | | | | | | | | | |
| 1  1 | randomised trial  non-randomised trial | Very serious risk of bias due to the randomisation process and confounding | No serious inconsistency_(3)_ | No serious indirectness | Imprecision arises because the lower bound of the 95% confidence interval crosses the null effect | None | 2 092 | 1 353 | Rate ratio 1.29 (0.99, 1.69) | 48 more per 1 000 person-years (2 fewer to 113 more) | ⊕OOO VERY LOW  Due to very serious risk of bias and imprecision |
| * Assuming a control group fall risk of 28.7%, a fall rate of 672 falls per 1,000 person-years, and a fall injury rate of 164 fall injuries per 1,000 person-years, based on data from Bergen et al. [1]. The risk in the intervention group is based on the assumed risk in the comparison group and the relative effect of the intervention (and its 95% CI). | | | | | | | | | | | |
| **Explanations**  (1) The numbers of participating healthcare practitioners were not reported.  (2) One study did not report number of participants.  (3) Heterogeneity was party explained by the subgroup analysis. | | | | | | | | | | | |

Reference

1. Bergen G, Stevens MR, Burns ER. Falls and Fall Injuries Among Adults Aged ≥65 Years — United States, 2014. MMWR Morb Mortal Wkly Rep. 2016;65: 993–998. doi:10.15585/mmwr.mm6537a2
