# Supplementary figures and images for: Effectiveness of clinical decision support in fall prevention among older adults: A systematic review and meta-analysis
Source: PLoS One. 2026 Jan 12;21(1):e0340025. doi: 10.1371/journal.pone.0340025 (PMC12795367; doi:10.1371/journal.pone.0340025)

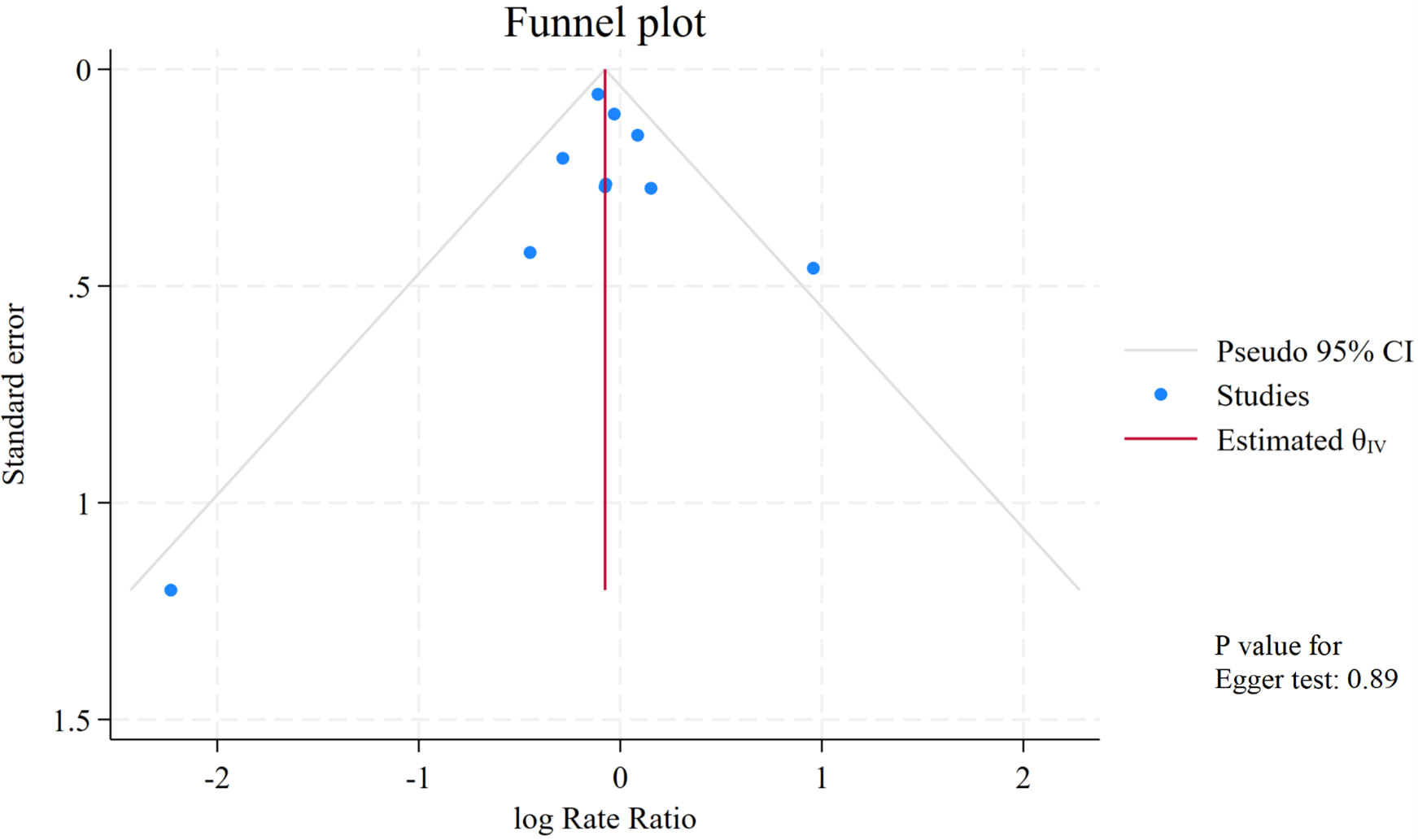

Supplement: S1 Fig — (PNG) [file pone.0340025.s013.png]

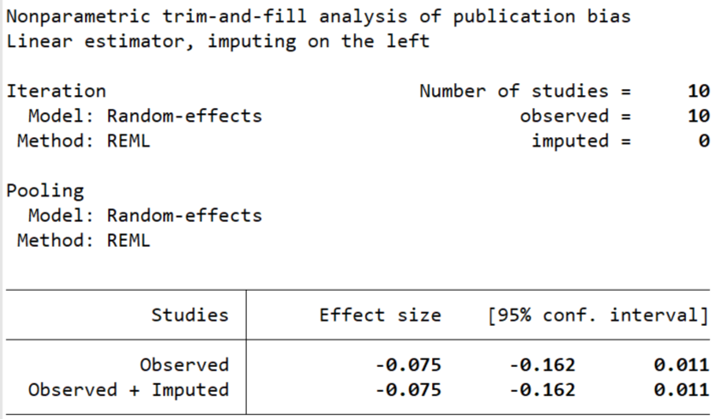

Supplement: S2 Fig — (PNG) [file pone.0340025.s014.png]

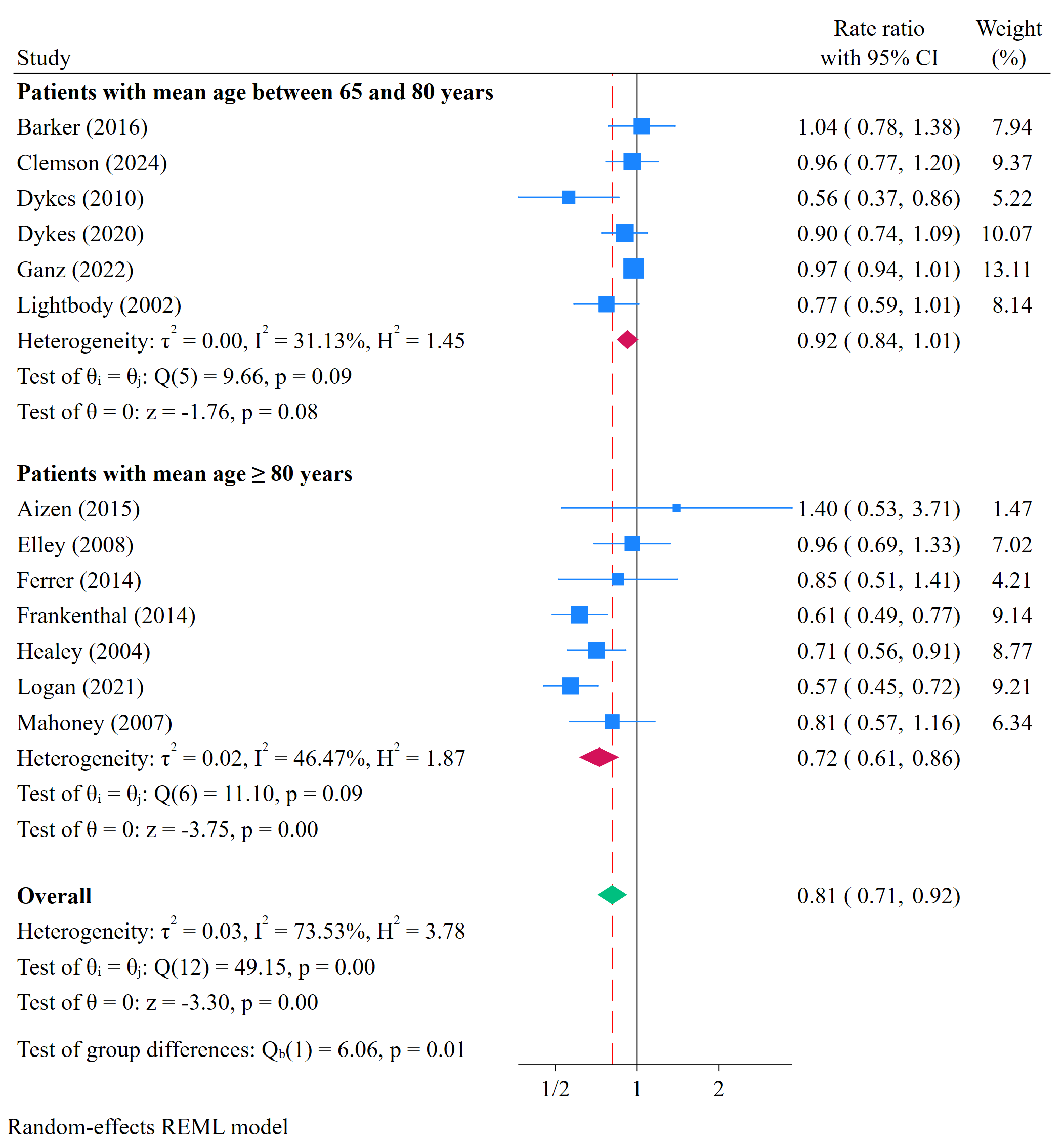

Supplement: S3 Fig — (PNG) [file pone.0340025.s015.png]

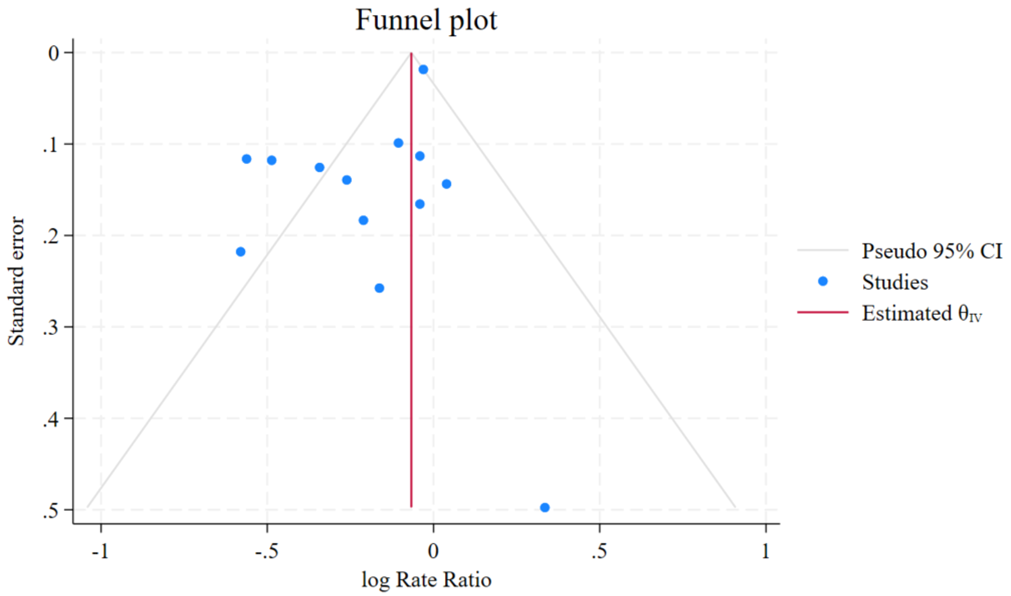

Supplement: S4 Fig — (PNG) [file pone.0340025.s016.png]

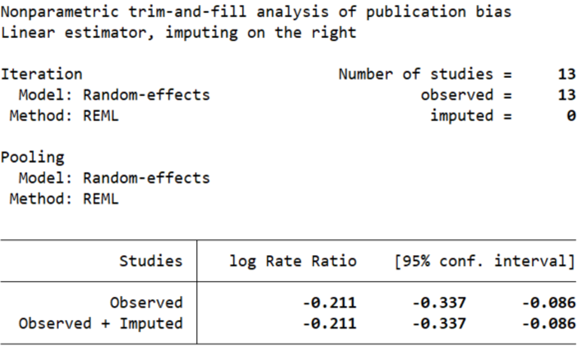

Supplement: S5 Fig — (PNG) [file pone.0340025.s017.png]

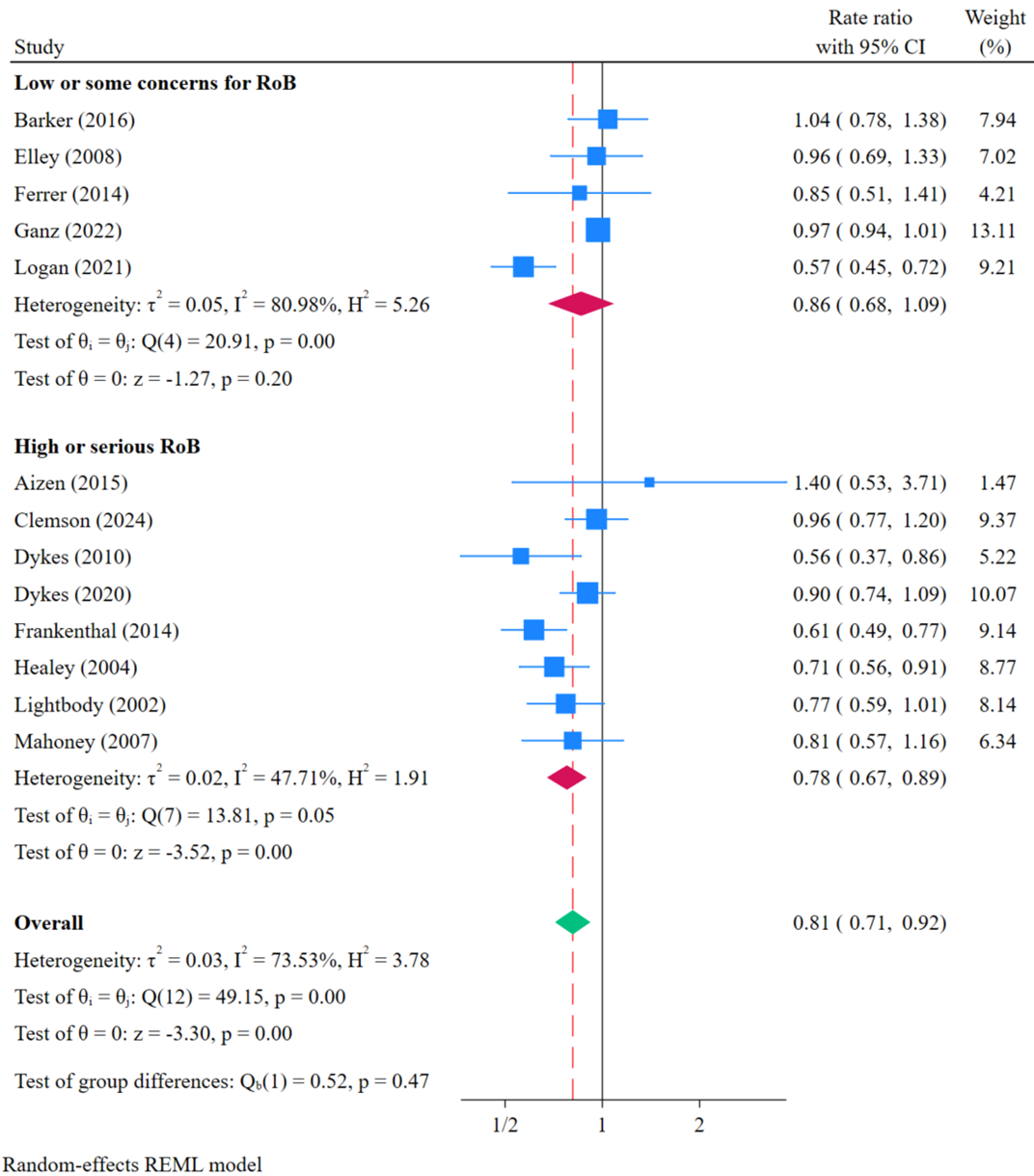

Supplement: S6 Fig — (PNG) [file pone.0340025.s018.png]

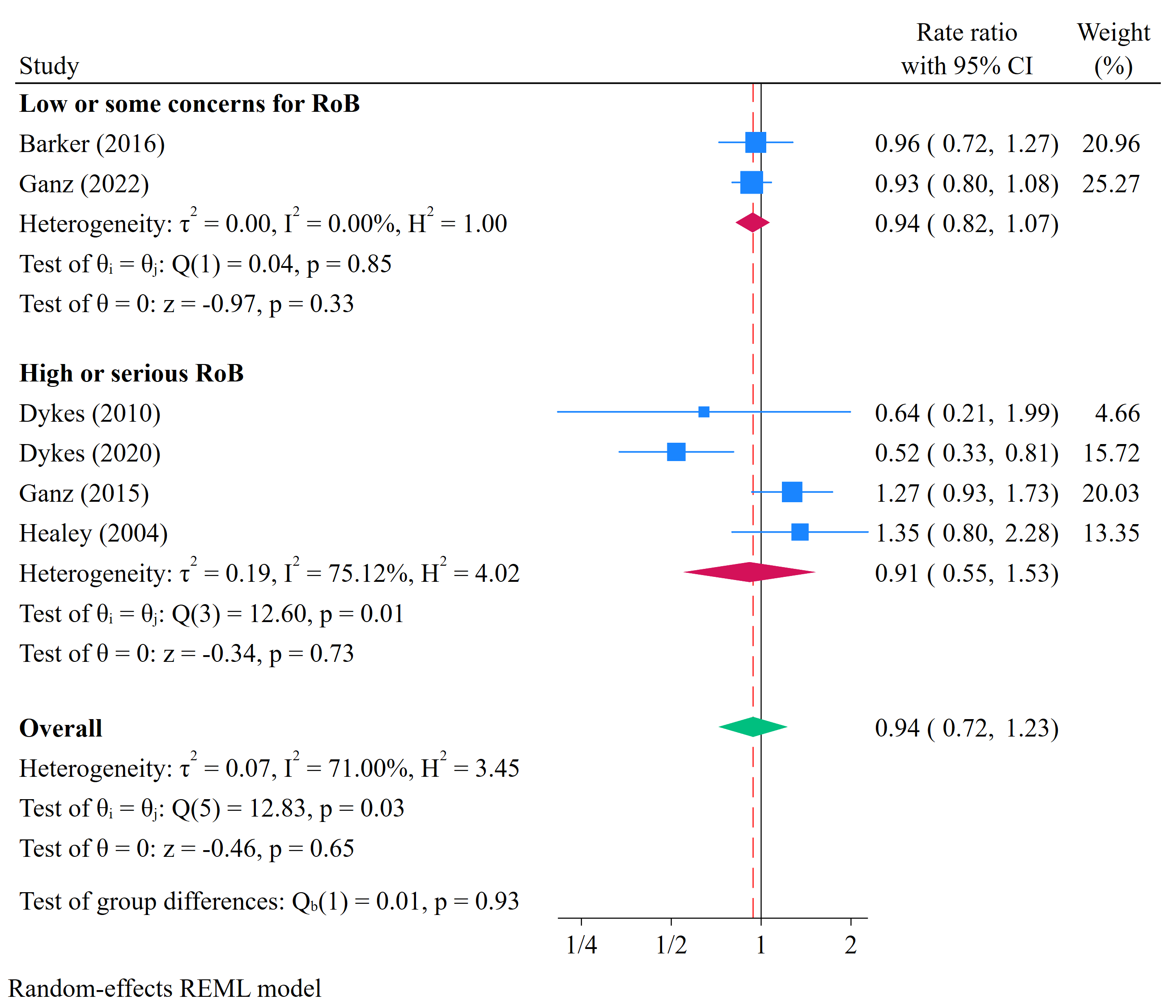

Supplement: S7 Fig — (PNG) [file pone.0340025.s019.png]

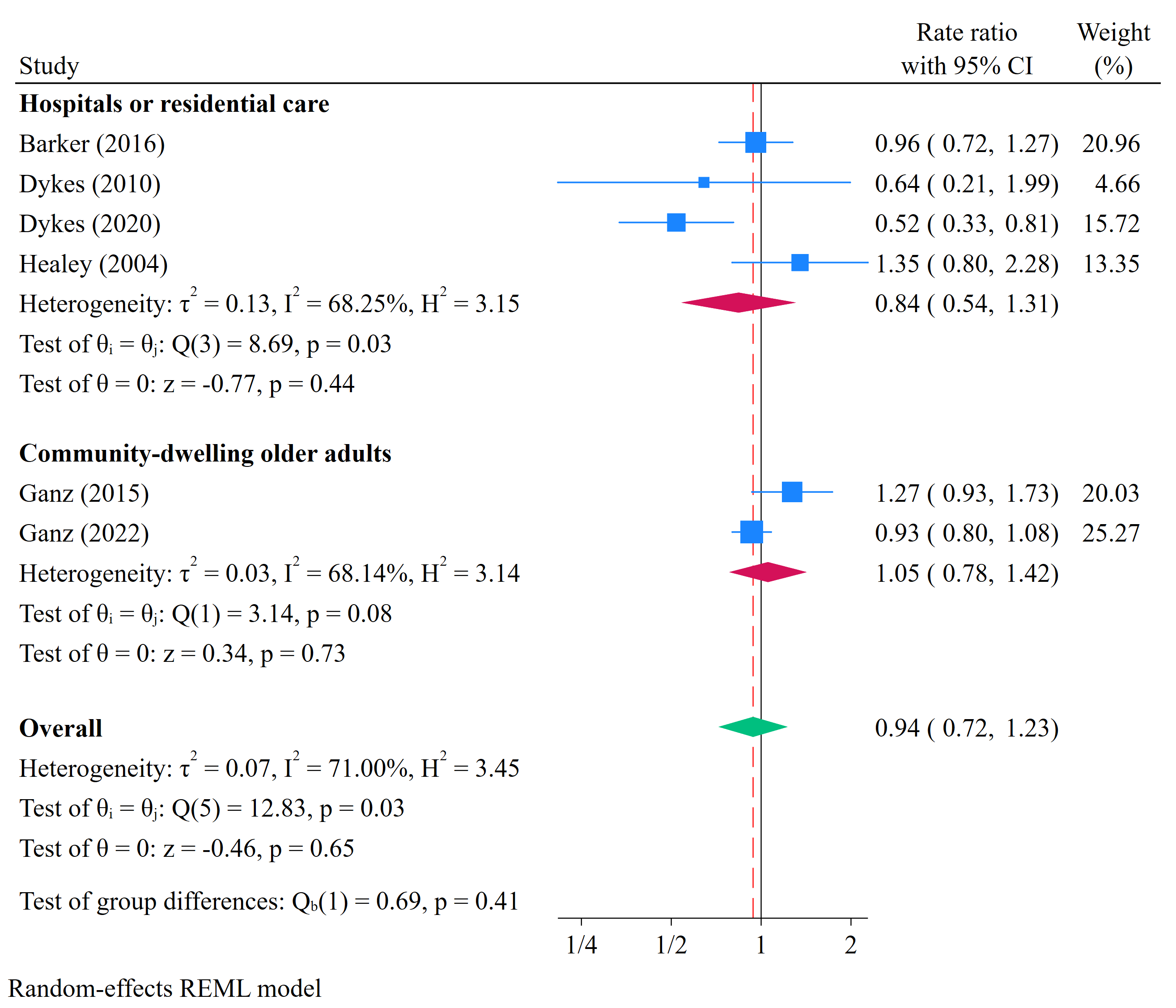

Supplement: S8 Fig — (PNG) [file pone.0340025.s020.png]
