# Supplementary material for: Effectiveness of clinical decision support in fall prevention among older adults: A systematic review and meta-analysis
Source: PLoS One. 2026 Jan 12;21(1):e0340025. doi: 10.1371/journal.pone.0340025 (PMC12795367; doi:10.1371/journal.pone.0340025)
Supplement: Attachment — Submitted filename: PONE-D-25-10237_R2_FC_OCT 6 2026.pdf [file pone.0340025.s023.pdf]

PLOS ONE

Effectiveness of clinical decision support in fall prevention among older adults: a systematic review and meta-analysis

--Manuscript Draft--

**Manuscript Number:**	PONE-D-25-10237R2
**Article Type:**	Research Article
**Full Title:**	Effectiveness of clinical decision support in fall prevention among older adults: a systematic review and meta-analysis
**Short Title:**	Effectiveness of clinical decision support in fall prevention: a systematic review and meta-analysis
**Corresponding Author:**	Rune Solli<br>Oslo Metropolitan University: OsloMet - storbyuniversitetet<br>Oslo, NORWAY
**Keywords:**	Falls; fall prevention; clinical decision support; implementation; Evidence-based Practice; Healthcare Practitioners
**Abstract:**	Background<br><br>Systematic use of Clinical Decision Support (CDS), which provides timely information to assist healthcare practitioners in decision-making, is recommended in the implementation of fall prevention among older adults. This systematic review aimed to evaluate the effects of CDS for fall prevention on healthcare practitioners' adherence to recommended practice, medication outcomes, and patient outcomes.<br><br>Methods<br><br>We searched Medline, EMBASE, CINAHL, Cochrane Library, Web of Science, AMED, PEDro, and Google Scholar from the earliest available dates through January 2025. We included randomised and non-randomised studies that directly compared interventions consisting of CDS presented on-screen or on paper to healthcare practitioners aiming to prevent falls in persons aged 65 years or older. We analysed healthcare practitioner performance, medication review and prescribing, fall risk, fall rate, and fall injury rate as primary outcomes. Two reviewers independently screened studies and assessed for risk of bias. We synthesised results using meta-analyses and vote-counting based on direction of effect, when possible, otherwise narratively, and we rated the certainty of the evidence using the GRADE approach.<br><br>Results<br><br>Of 25 included studies, 20 were randomised and five were non-randomised. Most CDS tools supported healthcare practitioners in performing multifactorial fall risk assessments and follow-up interventions based on identified risks (60%) and most were delivered electronically (60%). CDS may improve healthcare practitioners' adherence to recommended practice (all eight comparisons favouring CDS; 95% confidence interval [CI] 68% to 100%; low certainty) and likely improve medication review and prescribing (all nine comparisons favouring CDS; 95% CI 70% to 100%; moderate certainty), although the effect sizes are uncertain. CDS may reduce fall risk, but the effect may be small (odds ratio 0.93; 95% CI 0.81 to 1.01; low certainty). CDS likely reduces fall rates in hospitals or residential care (rate ratio [RaR] 0.74; 95% CI 0.63 to 0.88; moderate certainty) and in patients aged 80 years or older (RaR 0.72; 95% CI 0.61 to 0.86; moderate certainty). CDS may reduce fall rates in community-dwelling older adults (RaR 0.97; 95% CI 0.93 to 1.00; moderate certainty) and in patients aged between 65 and 80 years (RaR 0.92; 95% CI 0.84 to 1.01; low certainty), though the effects in both of these subgroups may be small. CDS may reduce fall injury rates in older adults aged between 65 and 80 years (RaR 0.80; 95% CI 0.59 to 1.09; low certainty). The evidence on fall injury rates in patients aged 80 years or older was very uncertain.<br><br>Conclusion

Powered by Editorial Manager® and ProduXion Manager® from Aries Systems Corporation

	CDS likely enhances healthcare practitioners' performance in fall prevention among older adults; however, the effect sizes remain unknown. Although CDS may improve patient outcomes in fall prevention, both the effect sizes and the certainty of evidence vary. Results from this study may inform the planning and implementation of CDS in fall prevention. Future studies should strive for clearer reporting of CDS design factors to allow for an evaluation of which factors may influence the success of CDS interventions in fall prevention.
**Order of Authors:**	Rune Solli
	Nina Rydland Olsen
	Linda Aimée Hartford Kvæl
	Stijn Van de Velde
	Are Hugo Pripp
	Signe Agnes Flottorp
	Therese Brovold
**Response to Reviewers:**	Response to Reviewers<br><br>Dear Dr. Nishant Premnath Jaiswal<br><br>Thank you for the opportunity to submit a revised draft of our manuscript titled "Effectiveness of clinical decision support in fall prevention among older adults: a systematic review and meta-analysis" to PLOS ONE. We greatly appreciate the time and effort you and the reviewers have dedicated to providing thoughtful and constructive feedback. We have carefully considered all comments and made revisions to address the suggestions provided.<br><br>All authors have reviewed and approved the submission of the revised manuscript. The manuscript has not been published and is not being considered for publication elsewhere, in whole or in part, in any language. We hope you will now be able to accept the article for publication in your journal.<br><br>Thank you for your consideration.<br><br>Yours sincerely<br>Rune Solli on behalf of the authors<br><br>Response to Reviewer 2<br>Comment 1:<br>This manuscript addresses an important and timely topic: the effectiveness of Clinical Decision Support (CDS) systems in fall prevention among older adults. The systematic review and meta-analysis are well designed, and the authors follow rigorous methods aligned with PRISMA and GRADE guidelines. However, several aspects require major attention before the manuscript can be considered for publication... Language and Grammar (Essential Revision):<br>The manuscript still contains numerous grammatical and syntactical issues that reduce clarity and weaken its impact. A professional English language editing is essential, especially in the Introduction and Discussion sections. For example:<br><br>Line 93: "Falls can cause significant health loss that leads to more complex care requirements" → awkward phrasing.<br><br>Line 92: "Falls are a major cause of morbidity and mortality…" → avoid redundancy by rephrasing the following lines more concisely.<br>Response 1:<br>Thank you for this comment. In response to your feedback, the manuscript has

Powered by Editorial Manager® and ProduXion Manager® from Aries Systems Corporation

undergone professional English language editing by a native English speaker. We have carefully revised the text, particularly the Introduction and Discussion sections, to improve clarity and address the issues you identified.

Comment 2:
GRADE Interpretation (Overstatement):
The authors frequently describe outcomes with low or very low certainty as "may improve" or "likely reduce." While this aligns with GRADE semantics, the consistent use of optimistic phrasing throughout the abstract and conclusion may mislead readers about the strength of evidence. I strongly recommend a more cautious tone, especially for:

Medication outcomes: classified as “low certainty” but still interpreted positively.

Fall injury in those 65–80 years: RaR 0.80 with CI crossing 1.00 should be clearly described as statistically non-significant.
Response 2:
Thank you for your comment on the interpretation of outcomes with low or very low certainty. We acknowledge that the repeated use of phrases such as “may improve” or “likely reduce” throughout the abstract and conclusion could potentially convey an overly optimistic impression. To address this, we have carefully reviewed the text and revised it ensure a more cautious and neutral tone, particularly for outcomes with low or very low certainty, to align with your suggestion and avoid the risk of misleading readers. Please see the updated text. We have explicitly described the result on fall injuries in those aged 65 to 80 years as statistically non-significant. Please see page 29, lines 556-557.

Comment 3:
Methodological Transparency:

The authors claim no language restriction, but earlier versions included a list of included languages. Ensure this inconsistency is corrected in both the Methods and Appendices.

Risk of bias assessments are now available in S7 Table, but summary RoB graphs in the main text are difficult to interpret (small font, unclear legends). Please improve readability and ensure consistency between main text and supplements.
Response 3:
Thank you for your comment regarding language restrictions. No language restrictions were imposed on the literature searches. In response to a previous Editor request, we removed the list of languages to avoid the implication that language restrictions had been applied. We have reviewed the methods section and Appendices to ensure consistency throughout. Additionally, we have revised the summary RoB graphs to improve readability, including increasing font sizes and clarifying legends, and to ensure consistency between the main text and supplements.

Comment 4:
Data Completeness (Minor):

The explanation for handling missing data is too brief. Please detail the imputation procedures used and specify how often author contact led to data retrieval.
Response 4
Thank you for your comment. We have expanded the Methods section to clarify that we contacted the corresponding authors of eight reports to obtain missing data, with up to three email attempts per author. Data retrieval was successful in four instances, and no data imputation was performed. Please see page 10, lines 208-213.

Comment 5:
Figures and Tables:

Figure readability is still suboptimal despite the revisions. Font sizes and labeling in forest plots and risk-of-bias visuals should be increased for clarity, especially for print

Powered by Editorial Manager® and ProduXion Manager® from Aries Systems Corporation

	versions.<br>Response 5:<br>Thank you for your comment. We have revised the figures to improve their readability in both the main text and supplementary materials, with particular attention to ensuring clarity in print versions. Font sizes and labelling in the forest plots and risk-of-bias visuals have been increased for enhanced clarity.<br><br>Comment 6:<br>Minor Points:<br><br>Use consistent terminology: sometimes "CDS systems" is used redundantly (the "S" already stands for "system").<br><br>Add a visual summary table of GRADE certainty per outcome (possibly alongside S9 Table) in the main manuscript for quick reference.<br><br>Discuss potential implementation barriers to CDS in LMIC settings – currently the review is biased toward high-income contexts.<br>Response 6:<br>Thank you for your observation regarding terminology. For clarity, in this manuscript, we have defined “CDS” as “clinical decision support” and have carefully reviewed the text to ensure consistent use and avoid any potential confusion.<br><br>We have added a GRADE summary of findings table in the manuscript for quick reference. Please see Table 4.<br><br>Thank you for highlighting the important point about implementation in LMIC. We acknowledge that the review predominantly focuses on high-income contexts. To address this, we have added a discussion of potential implementation barriers in LMIC, including limited digital infrastructure, restricted access to electronic health records, overburdened staff, and other challenges. Please see page 35, lines 713-724.<br><br>Response to Reviewer 3<br>Comment 1:<br>Pg. 5 ln 118 - Suggest changing to "Generally, the use of CDS by healthcare ...."<br>Pg 5 ln 121 - suggest changing to "patients [32], medication outcomes in older adults [33,34], and adults in general.<br>Pg 6 ln 134 - suggest changing to "This systematic review aimed to evaluate the effects of CDS for...."<br>Pg 8 ln 172 - since your inclusion criteria included restrictions on the type of studies for inclusion in the review, please clarify why you did not include restrictions on the study design within your search strategy?<br>Pg 10 ln 208 - Why did you include studies which did not specify one of your required outcomes?<br>Pg 14 ln 301 - what do you mean by the phrase "to a median (min-max) of 1 433 (312-46245) patient participants?<br>Pg 24 ln 460 - your interpretation of the impact of CDS on falls in hospital/residential care, although was significant, needs to include the considerable heterogenity present.<br>Pg 24 ln 466-478 - your interpretation of the sub group analysis I feel is inaccurate and likely do not reduce the rate of falls.<br>Pg 28 ln 572-574 - your results do not reflect that CDS does not reduce falls in pts >80<br>Pg 33 - Strengths and limitations section - please expand more on limitations of the review<br>Response 1:<br>Pg. 5 ln 118 - Thank you for the suggestion. We have updated the sentence to: "Generally, the use of CDS by healthcare ..." for improved clarity.<br>Pg 5 ln 121 - We have revised the text accordingly.<br>Pg 6 ln 134 - Thank you for your suggestion. We have revised the sentence to: “This systematic review aimed to evaluate the effects of CDS for fall prevention on …”<br>Pg 8 ln 172 – Thank you for this observation. Our inclusion criteria restricted study types during the screening and selection phases to ensure relevance and rigor. However, we did not restrict study design in the search strategy to maximise sensitivity, as publications may not always disclose the study design.<br>Pg 10 ln 208 - Thank you for your questions. Studies that did not specify one of our

Powered by Editorial Manager® and ProduXion Manager® from Aries Systems Corporation

	required outcomes were excluded. We have revised Table 1 in the Methods section to clarify this decision and enhance transparency.<br>Pg 14 ln 301 - This phrase indicates the median number of patient participants included across the studies, with the smallest study having 312 participants and the largest having 46,245. To improve clarity, have revised this sentence to: "… to a median number of 1,433 patient participants, ranging from 312 to 46,245."<br>Pg 24 ln 460 – Thank you for pointing this out. We have added a sentence in the Results section to acknowledge the substantial heterogeneity and revised the Discussion section to address the considerable heterogeneity and its implications for interpretating the effects of CDS on falls in hospital/residential care settings.<br>Pg 24 ln 466-478 – Thank you for pointing this out. Considering comments from all reviewers, we have updated the grading of certainty to explicitly reflect the certainty in the direction of effect. Minor adjustments were made in the certainty ratings, and the corresponding statements about the results have been revised for accuracy and clarity. For further details, please see our response to comment 5 from reviewer 4.<br>Pg 28 ln 572-574 - Thank you for your comment. Our results reflect that CDS interventions reduce the rate of falls among patients aged 80 years or older. In accordance with GRADE methodology, we have used the term "likely" to describe findings supported by moderate certainty evidence. We have retained this wording and clarified this choice in the methods section under "Assessment of certainty of the evidence". Please see page 14, lines 312-313 and Table 4.<br>Pg 33 - Strengths and limitations section - Thank you for the suggestion. We have expanded the Strengths and limitations section to include additional limitations, such as the uncertainty surrounding the specific effects of different types of CDS tools and the substantial risk of bias in most of the included studies.<br><br>Response to Reviewer 4<br>Comment 1<br>Thank you for the opportunity to review this revised manuscript. I did not review the original version but have examined the responses to the previous reviewer comments and have reviewed the revised version anew from my perspective.<br>I did not verify that all requests by the Academic Editor were addressed, but notice that the author did not add any information into the manuscript, as stated in their response, about any methods for author contact for missing outcome data. If they did perform author contact, the means (eg email first author) and frequency should be stated.<br>Response 1<br>Thank you for your comment. We have updated the Methods section to include details about author contact for missing outcome data. Specifically, we clarified that corresponding authors were contacted via email, with up to three follow-up attempts if necessary. Please see page 10, lines 208-211.<br><br>Comment 2<br>Abstract: i) please note the search date(s), ii) if space permits it would be excellent to provide a brief overview of the types of CDS interventions used most, iii) please see below for some suggestions and questions about the GRADE approach which may influence the reporting of results in the abstract<br>Introduction: stating that "interventions to prevent falls" are cost-effective and effective in falls and fall injuries could be more specific especially since not all interventions have been shown successful and the authors are arguing for a review of CDS interventions. Possibly "a variety of interventions aimed at older adults" or such would help.<br>Response 2<br>Thank you for these suggestions. We have added the search dates to the Methods section of the abstract, included a brief overview of the most used types of CDS, and reviewed the reporting of results in the abstract considering your suggestions regarding the GRADE approach. Please see the revised abstract. In the introduction, we have specified the types of fall prevention interventions that are cost-effective and effective in reducing falls and fall injuries. Please see pages 4-5, lines 103-107.<br><br>Comment 3:<br>Materials and methods:<br>1. Was the protocol only ever "drafted"? even if there were changes post hoc the protocol should have been in some form of final version before starting the review.<br>2. Spell out PRISMA at first use

Powered by Editorial Manager® and ProduXion Manager® from Aries Systems Corporation

	3. Selection process: please add details about who conducted full text review, and confirm whether consensus was required at title/abstract stage or just full text.<br>4. Data collection process: using verification for only 5 of 24 papers should be noted in the discussion as a limitation of the review (possibility for errors). Duplicate extraction or verification of all data is a standard for systematic reviews. This is particularly concerning if only one person chose which outcome data to use when studies reported on multiple outcomes.<br>5. Study risk of bias: please confirm if "the main outcome results" means the result data used for each outcome of interest to the review, or otherwise.<br>6. Synthesis methods: please add a section on unit of analysis issues, i.e. what was done to account for effects of clustering in cluster RCTs? Was the authors' result adjusted for clustering used for analysis and, if not reported/performed, what was done?<br>Response 3<br>1. Thank you for your comment. The protocol was drafted, finalised, and registered in PROSPERO before commencing the review. We have clarified this in the Methods section. Please see page 7, lines 152-156.<br>2. We have spelled out PRISMA at its first mention.<br>3. Thank you for your comment. We have added details about who conducted full-text review and clarified that consensus was required at both the title/abstract screening stage and the full-text screening stage. Please see page 9, lines 197-201.<br>4. Thank you for highlighting this issue. We have discussed the limited verification of collected data by a second reviewer as a limitation of the review in the Discussion section to acknowledge the possibility of errors. Additionally, we have clarified that the selection of each result was discussed and verified with the project statistician to ensure correctness and consistency. Please see page 38, lines 784-787.<br>5. We have specified which outcomes were assessed for risk of bias. Please see page 12, lines 253-254.<br>6. Thank you for your comment regarding unit of analysis issues. The cluster-randomised trials included in the review accounted for clustering in their original analyses. Additionally, in our syntheses, we ensured that only one outcome from each study was included, thus maintaining independence between studies in the analyses. We have included a sentence in the methods section to clarify this. Please see page 13, lines 291-292.<br><br>Comment 4<br>Results/conclusions:<br>1. it would be of interest to comment on how many studies had eligibility criteria related to increased risk for falls (e.g. previous fallers, 1+ risk factor etc), and to state whether the findings overall are most applicable to populations at increased risk (this may vary by outcome to some degree),<br>2. it is hard to know whether the findings for the fall risk outcome may be most applicable to certain settings or ages; can the authors comment on whether the 10 studies appeared to capture both settings and ages fairly well? For example, if the large majority of the participants in the analysis came from studies undertaken in residential/acute care there could be some concerns about directness to the entire population of interest (eg rating down for indirectness may apply) or some way to clarify this observation for the reader,<br>3. likewise with setting and age groups for fall rates is there any indication that the studies in residential/acute care also enrolled older people to help know whether findings could be most applicable to both setting and age combined?,<br>4. conversion of ORs to absolute risk difference (for falls risk) should first convert the OR to a RR (see appendix 3 in https://www.bmj.com/content/389/bmj-2024-081904),<br>5. the authors should speak to their reason for use of a separate study (ref 88) for control event rates (rather than the studies themselves) and describe this study in some detail,<br>6. in figure 2 there are some "FU" and "follow-up" so this could be made consistent; further the authors should be able to put something into the figure for the medication outcome findings for the Blalock study, even if just a narrative statement by the authors about the direction of effect,<br>7. for mortality and hospitalizations the reversion to relying on statistical significance goes against what was done for other outcomes; perhaps the authors comment about any directions of effect, speak to the small sample sizes for these rare outcomes and point again to where folks can find the results if they want.

Powered by Editorial Manager® and ProduXion Manager® from Aries Systems Corporation

Line 561-2: would suggest deleting this sentence since the authors did not assess the magnitude of effects in their review
Response 4:
1. Thank you for your comment. We have added details about how many studies included eligibility criteria related to increased fall risk in the results section. Please see pages 14-15, lines 337-339. Additionally, we have stated in the discussion section that the findings of this review are likely most applicable to populations at higher fall risk. Please see page 36, lines 743-745.
2. We have added details in the results section regarding the number of studies reporting fall risk that focused on different settings and age groups. These details suggest that the findings are largely applicable to the entire population of interest. Please see page 24, lines 446-449. Additionally, we have commented on the applicability of the findings from the meta-analysis on fall risk in the Discussion section. Please see page 33, lines 657-659.
3. Thank you for this comment. We have added details in the results section regarding the number of studies that included patients from different age groups within the hospitals or residential care subgroup. Please see pages 25-26, lines 488-492. Similarly, we have added details regarding the number of studies conducted in each setting within the subgroup by patients' age. Please see page 27 lines 508-512. Additionally, we have commented on the applicability of the findings from the meta-analyses on fall rate in the Discussion section. Please see page 34, lines 677-679.
4. Thank you for your comment. Before calculating the absolute fall risk in the intervention group and the absolute risk difference, we have converted the ORs (including the point estimate and lower and upper limits of the confidence interval) to RRs using the formula provided in appendix 3 of https://www.bmj.com/content/389/bmj-2024-081904. This process has been described in the methods section for clarity. Please see page 13, lines 295-297.
5. Thank you for your comment regarding the use of a separate study for control event rates. Due to the limited reporting of absolute numbers in the studies included in this review, we derived control intervention risks and rates from a report describing the epidemiology of falls among older adults. This approach aligns with Cochrane methodology [1]. We have clarified this in the methods section and provided a description of the study used for control event risks and rates. Please see page 13, lines 293-295. Additionally, we updated the control fall risk from 30% to 28.7%, as reported in the study. Please see Table 4.
6. In figure 2, we have replaced all instances of 'follow-up' with 'FU' for consistency and added a description of the medication outcome results for the Blalock study.
7. We acknowledge your comment about the reliance on statistical significance and have revised the text to place greater emphasis on the direction of effect, the small sample size, and the rarity of the outcomes. Additionally, we have informed the reader where individual study results can be found for further details.
Line 561-2: Thank you for your feedback regarding the sentence: "Had more CDS tools been designed for automatic decision support it could potentially have increased healthcare practitioners' adherence to recommendations." We have removed the sentence as suggested.

Comment 5
Major comment:
GRADE does not assess the certainty of the point estimate per se but it's relation to the "target" of certainty which should be defined in the methods section (see GRADE 34 guidance and J Clin Epidemiol. 2017 Jul:87:4-13. doi: 10.1016/j.jclinepi.2017.05.006 and BMJ. 2025 Apr 29:389:e081904. doi: 10.1136/bmj-2024-081904). For the provider outcomes, the target would need to be a direction of effect only since the analysis did not assess magnitude. For the patient outcomes, this could have been the null (direction of effect) or some form of a minimally important difference (even if approximate), especially to help determine whether findings are "little to no difference" or "an affect". It appears the authors just used direction of effect and if so this should be mentioned and the findings should be stated in this light. It is confusing what the authors have done for this since there is no statement in the methods and for falls risk (OR 0.93, 21 fewer fallers per 1000) they state little to no effect in the results (implying 21 fewer is below some threshold of an MID) but a slight reduction in the abstract and discussion.
The choice of the target can change the GRADE ratings for all domains but in particular inconsistency and imprecision. For the provider outcomes, rating a

conclusion of direction of effect where all studies showed the same direction would suggest against rating down for inconsistency; the size of effect doesn't matter for this target. With some of the effects not being statistically significant/precise for the direction there could be concern over imprecision but it may not be serious. For direction of effect for adherence, I would think low certainty overall seems appropriate with the text indicating rating down twice for ROB. The same applies for medication outcomes, unless the authors want to have a third category of No direction which a couple of the findings indicate (e.g. Blum & Lightbody) and where some inconsistency in direction could be noted. If the authors really think low certainty for the direction is appropriate (vs moderate with just serious ROB) they could consider rating down twice for ROB or possibly once for ROB and once for imprecision (as above) or inconsistency (noting really that a direction was not shown in a couple of cases). For the falls risk outcome, if the authors are rating certainty in direction of effect, then not rating down for imprecision makes sense (assuming they wouldn't be too strict on the upper limit of the 95% CI crossing the null slightly) and they would conclude there is an effect (possibly mentioning that it may not be important), but if they want to say there is "little to no" difference (implying use of an MID and that the point estimate is below this) then they likely should state what the MID was and make sure the entire 95% CI does not contain this value or else also rate down for imprecision. For the falls rate outcomes, if using a direction of effect the authors should note this in their conclusions whereas if they think the magnitude is at least as large as some small but important effect they may also rate down for either inconsistency (since ~30% of the weight in the analysis showed effects below what might be considered an MID) or for imprecision if the lower limit of the 95%CI (81 fewer falls) does not surpass their MID.
In summary, having a clear statement about what the authors were rating their certainty in (e.g. null/direction of effect) would be good as well as re-considering their assessments for the provider outcomes in light of this (not rating down for inconsistency). If using the direction of effect their falls risk findings should likely be "an effect", with a comment that the effects may be small. If MIDs were applied these should be stated with the above considerations added.
Response 5
Thank you for your detailed feedback regarding the use of GRADE to assess the certainty of evidence in this study. We appreciate your suggestions for clarifying our approach.

We graded the certainty of evidence based on the direction of effect rather than the MID. This decision was informed by the lack of established MID values for the outcomes of interest (fall risk, fall rate, and fall injury rate). Despite extensive literature searches conducted with the support of two librarians at OsloMet University, we were unable to identify absolute MID values for these outcomes, except for a single relative fall rate value specific to Parkinson's patients (25%). Given the lack of absolute MID values applicable to fall risk, fall rate, and fall injury rate, we determined that assessing the certainty of evidence for the direction of effect was the most appropriate approach.

In response to your feedback, we have revised the methods section to explicitly state that we assessed certainty in the direction of effect, using the null effect as the threshold. We also re-evaluated our GRADE ratings to reflect this approach. For provider outcomes, we did not rate down for inconsistency where all studies showed the same direction of effect, as suggested. For the fall risk outcome, we rated down for imprecision because of the slight crossing of the null by the upper limit of the CI.

To ensure consistency in reporting, we revised the results and discussion sections, as well as the abstract, to frame findings in terms of the direction of effect. For example, we now describe the findings for fall risk as indicating "an effect," while noting that the effects may be small. Additionally, we added a summary table of GRADE certainty for each outcome (Table 4) to facilitate quick reference. Finally, we added a note in the discussion to acknowledge the lack of established MID values and highlighted the need for future work to establish such thresholds for fall prevention outcomes. Please see page 36, lines 726-740.

References

Powered by Editorial Manager® and ProduXion Manager® from Aries Systems Corporation

	1. Schünemann HJ, Higgins JPT, Vist GE, Glasziou P, Akl EA, Skoetz N, et al. Chapter 14: Completing 'Summary of findings' tables and grading the certainty of the evidence. 6.4. Higgins JPT, Thomas J, Chandler J, Cumpston M, Li T, Page MJ, Welch VA (editors) Cochrane Handbook for Systematic Reviews of Interventions version 64 (updated August 2023) Cochrane, 2023 Available from www.training.cochrane.org/handbook. 6.4. Cochrane; 2023.
Additional Information:	
Question	Response
**Financial Disclosure**<br><br>Enter a financial disclosure statement that describes the sources of funding for the work included in this submission. Review the submission guidelines for detailed requirements. View published research articles from *PLOS ONE* for specific examples.<br><br>This statement is required for submission and **will appear in the published article** if the submission is accepted. Please make sure it is accurate.<br><br>**Funded studies**<br>Enter a statement with the following details:<br>• Initials of the authors who received each award<br>• Grant numbers awarded to each author<br>• The full name of each funder<br>• URL of each funder website<br>• Did the sponsors or funders play any role in the study design, data collection and analysis, decision to publish, or preparation of the manuscript?<br><br>Did you receive funding for this work?	Yes
Please add funding details.<br>as follow-up to "**Financial Disclosure**<br><br>Enter a financial disclosure statement that describes the sources of funding for the work included in this submission. Review the submission guidelines for detailed requirements. View published research articles from *PLOS ONE* for specific examples.<br><br>This statement is required for submission	This project received funding from the Research Council of Norway (project no. 301996). https://www.forskningsradet.no/en/<br>The funder did not play any role in the study design, data collection and and analysis, decision to publish, or preparation of the manuscript.

and **will appear in the published article** if the submission is accepted. Please make sure it is accurate.<br><br>**Funded studies**<br>Enter a statement with the following details:<br>• Initials of the authors who received each award<br>• Grant numbers awarded to each author<br>• The full name of each funder<br>• URL of each funder website<br>• Did the sponsors or funders play any role in the study design, data collection and analysis, decision to publish, or preparation of the manuscript?<br><br>Did you receive funding for this work?"	
Please select the country of your main research funder (please select carefully as in some cases this is used in fee calculation).<br>as follow-up to "**Financial Disclosure**<br><br>Enter a financial disclosure statement that describes the sources of funding for the work included in this submission. Review the submission guidelines for detailed requirements. View published research articles from *PLOS ONE* for specific examples.<br><br>This statement is required for submission and **will appear in the published article** if the submission is accepted. Please make sure it is accurate.<br><br>**Funded studies**<br>Enter a statement with the following details:<br>• Initials of the authors who received each award<br>• Grant numbers awarded to each author<br>• The full name of each funder<br>• URL of each funder website<br>• Did the sponsors or funders play any role in the study design, data collection and analysis, decision to publish, or preparation of the manuscript?	NORWAY - NO

Did you receive funding for this work?"	
**Competing Interests**<br><br>Use the instructions below to enter a competing interest statement for this submission. On behalf of all authors, disclose any competing interests that could be perceived to bias this work—acknowledging all financial support and any other relevant financial or non-financial competing interests.<br><br>This statement is required for submission and **will appear in the published article** if the submission is accepted. Please make sure it is accurate and that any funding sources listed in your Funding Information later in the submission form are also declared in your Financial Disclosure statement.<br><br>View published research articles from *PLOS ONE* for specific examples.<br><br>**NO authors have competing interests**<br><br>Enter: *The authors have declared that no competing interests exist.*<br><br>**Authors with competing interests**<br><br>Enter competing interest details beginning with this statement:<br><br>*I have read the journal's policy and the authors of this manuscript have the following competing interests: [insert competing interests here]*<br><br>* typeset	The authors have declared that no competing interests exist.
**Ethics Statement**<br><br>Enter an ethics statement for this submission. This statement is required if the study involved:	N/A

• Human participants<br>• Human specimens or tissue<br>• Vertebrate animals or cephalopods<br>• Vertebrate embryos or tissues<br>• Field research<br><br>Write "N/A" if the submission does not require an ethics statement.<br><br>General guidance is provided below. Consult the submission guidelines for detailed instructions. **Make sure that all information entered here is included in the Methods section of the manuscript.**	

Powered by Editorial Manager® and ProduXion Manager® from Aries Systems Corporation

**Format for specific study types**<br><br>**Human Subject Research (involving human participants and/or tissue)**<br>• Give the name of the institutional review board or ethics committee that approved the study<br>• Include the approval number and/or a statement indicating approval of this research<br>• Indicate the form of consent obtained (written/oral) or the reason that consent was not obtained (e.g. the data were analyzed anonymously)<br><br>**Animal Research (involving vertebrate animals, embryos or tissues)**<br>• Provide the name of the Institutional Animal Care and Use Committee (IACUC) or other relevant ethics board that reviewed the study protocol, and indicate whether they approved this research or granted a formal waiver of ethical approval<br>• Include an approval number if one was obtained<br>• If the study involved *non-human primates*, add *additional details* about animal welfare and steps taken to ameliorate suffering<br>• If anesthesia, euthanasia, or any kind of animal sacrifice is part of the study, include briefly which substances and/or methods were applied<br><br>**Field Research**<br><br>Include the following details if this study involves the collection of plant, animal, or other materials from a natural setting:<br>• Field permit number<br>• Name of the institution or relevant body that granted permission	
**Data Availability**<br><br>Authors are required to make all data underlying the findings described fully available, without restriction, and from the time of publication. PLOS allows rare exceptions to address legal and ethical concerns. See the PLOS Data Policy and FAQ for detailed information.	Yes - all data are fully available without restriction

Powered by Editorial Manager® and ProduXion Manager® from Aries Systems Corporation

A Data Availability Statement describing where the data can be found is required at submission. Your answers to this question constitute the Data Availability Statement and **will be published in the article**, if accepted.<br><br>**Important:** Stating 'data available on request from the author' is not sufficient. If your data are only available upon request, select 'No' for the first question and explain your exceptional situation in the text box.<br><br>Do the authors confirm that all data underlying the findings described in their manuscript are fully available without restriction?	
**Describe where the data may be found in full sentences. If you are copying our sample text, replace any instances of XXX with the appropriate details.**<br><br>• If the data are **held or will be held in a public repository**, include URLs, accession numbers or DOIs. If this information will only be available after acceptance, indicate this by ticking the box below. For example: *All XXX files are available from the XXX database (accession number(s) XXX, XXX.*).<br>• If the data are all contained **within the manuscript and/or Supporting Information files**, enter the following: *All relevant data are within the manuscript and its Supporting Information files.*<br>• If neither of these applies but you are able to provide **details of access elsewhere**, with or without limitations, please do so. For example:<br><br>*Data cannot be shared publicly because of [XXX]. Data are available from the XXX Institutional Data Access / Ethics Committee (contact via XXX) for researchers who meet the criteria for access to confidential data.*<br><br>*The data underlying the results presented in the study are available from (include the name of the third party*	All relevant data are within the manuscript and its Supporting Information files.

and contact information or URL).<br>• This text is appropriate if the data are owned by a third party and authors do not have permission to share the data.<br><br>* typeset	
Additional data availability information:	

Powered by Editorial Manager® and ProduXion Manager® from Aries Systems Corporation

Manuscript Click here to access/download;Manuscript;Manuscript.docx

Effectiveness of clinical decision support in fall prevention among older adults: a systematic review and meta-analysis

Rune Solli[1*], Nina Rydland Olsen[2], Linda Aimée Hartford Kvæl[1,3], Stijn Van de Velde[4], Are Hugo Pripp[1], Signe Agnes Flottorp[5], Therese Brovold[1]

[1] Department of Rehabilitation Science and Health Technology, Faculty of Health Sciences, OsloMet – Oslo Metropolitan University, Oslo, Norway

[2] Department of Health and Functioning, Western Norway University of Applied Sciences, Faculty of health and social sciences, Bergen, Norway

[3] Norwegian Social Research (NOVA), OsloMet – Oslo Metropolitan University, Oslo, Norway

[4] MAGIC Evidence Ecosystem Foundation; Research Department, Lovisenberg Diaconal Hospital, Oslo, Norway

[5] Division of Health Services, Norwegian Institute of Public Health, Oslo, Norway

*Corresponding author
Email: runesoll@oslomet.no (RS)

Abstract

Background: Systematic use of Clinical Decision Support (CDS), which provides timely information to assist healthcare practitioners in decision-making, is recommended in the implementation of fall prevention among older adults. This systematic review aimed to evaluate the effects of CDS for fall prevention on healthcare practitioners' adherence to recommended practice, medication outcomes, and patient outcomes.

Methods: We searched Medline, EMBASE, CINAHL, Cochrane Library, Web of Science, AMED, PEDro, and Google Scholar from the earliest available dates through January 2025. We included randomised and non-randomised studies that directly compared interventions consisting of CDS presented on-screen or on paper to healthcare practitioners aiming to prevent falls in persons aged 65 years or older. We analysed healthcare practitioner performance, medication review and prescribing, fall risk, fall rate, and fall injury rate as primary outcomes. Two reviewers independently screened studies and assessed for risk of bias. We synthesised results using meta-analyses and vote-counting based on direction of effect, when possible, otherwise narratively, and we rated the certainty of the evidence using the GRADE approach.

Results: Of 25 included studies, 20 were randomised and five were non-randomised. Most CDS tools supported healthcare practitioners in performing multifactorial fall risk assessments and follow-up interventions based on identified risks (60%) and most were delivered electronically (60%). CDS may improve healthcare practitioners' adherence to recommended practice (all eight comparisons favouring CDS; 95% confidence interval [CI] 68% to 100%; low certainty) and likely improve medication review and prescribing (all nine comparisons favouring CDS; 95% CI 70% to 100%; moderate certainty), although the effect

sizes are uncertain. CDS may reduce fall risk, but the effect may be small (odds ratio 0.93; 95% CI 0.81 to 1.01; low certainty). CDS likely reduces fall rates in hospitals or residential care (rate ratio [RaR] 0.74; 95% CI 0.63 to 0.88; moderate certainty) and in patients aged 80 years or older (RaR 0.72; 95% CI 0.61 to 0.86; moderate certainty). CDS may reduce fall rates in community-dwelling older adults (RaR 0.97; 95% CI 0.93 to 1.00; moderate certainty) and in patients aged between 65 and 80 years (RaR 0.92; 95% CI 0.84 to 1.01; low certainty), though the effects in both of these subgroups may be small. CDS may reduce fall injury rates in older adults aged between 65 and 80 years (RaR 0.80; 95% CI 0.59 to 1.09; low certainty). The evidence on fall injury rates in patients aged 80 years or older was very uncertain.

Conclusion: CDS likely enhances healthcare practitioners' performance in fall prevention among older adults; however, the effect sizes remain unknown. Although CDS may improve patient outcomes in fall prevention, both the effect sizes and the certainty of evidence vary. Results from this study may inform the planning and implementation of CDS in fall prevention. Future studies should strive for clearer reporting of CDS design factors to allow for an evaluation of which factors may influence the success of CDS interventions in fall prevention.

Registration: PROSPERO, CRD42021250500

Key words: Falls; Fall Prevention; Clinical Decision Support; Implementation; Evidence-based practice; Healthcare practitioners.

Introduction

Falls are a major cause of morbidity and mortality in community-dwelling older adults aged 65 years or older [1–3]. Annually, one in three older adults experiences a fall, which can lead to significant health loss and increased care needs [1–5]. Globally, fall-related injuries are one of the most expensive conditions in terms of economic expenditures [6–8]. A history of falls, higher age, female sex, and fear of falling are among the multitude of identified risk factors for falls [9,10]. There are a variety of interventions aimed at preventing falls in older adults that are cost-effective [11,12] and are associated with a reduction in the number of falls and fall-related injuries [11,13]. These interventions include muscle strength and balance training, home safety assessments and modifications, and medication adjustments [11,12]. Falls are often underreported, as older adults may not report falls unprompted [14,15]. The World Falls Guidelines 2022 (WFG2022) [16] emphasise the need to identify older adults at increased fall risk and recommend that all older adults receive advise on fall prevention and physical activity.

Despite scientific support for the implementation of fall-prevention recommendations for older adults, the systematic uptake of evidence-based fall prevention practices has been slow [16–19], and fall rates and fall-related mortality have not declined [3,20,21]. The WFG2022 recommend the systematic use of Clinical Decision Support (CDS) in fall prevention to identify older adults at increased risk of falling and to facilitate fall risk assessments and interventions. CDS is an implementation strategy found to improve healthcare practitioners' adherence to clinical guidelines [22]. CDS is defined as computerised or non-computerised tools that combine health-related and medical information with individual patient information to support clinical decision-making [23,24]. CDS may aid healthcare practitioners in identifying older adults at increased fall risk [25–27] as well as in individualising fall prevention interventions based on identified risk factors [26,28–30].

Several systematic reviews have evaluated the effects of CDS used by healthcare practitioners across a variety of settings, demonstrating small to moderate positive effects [22,31–39]. Generally, the use of CDS by healthcare practitioners may improve the implementation of clinical practice guidelines [22] and the performance of nurses and allied health professionals [31,36,37]. In addition, CDS may improve the hospital care of older patients [32], medication outcomes in older adults [33,34] and in adults in general [38,39]. The effect on patient outcomes seems less certain [37]; while the use of CDS by healthcare practitioners may not affect mortality, it may moderately improve morbidity outcomes [35] and a variety of other patient outcomes [38]. Although two systematic reviews found that CDS tools may prevent falls in nursing homes [31] and in hospitals [32], these reviews did not identify multiple known studies on the effects of CDS in fall prevention [40,41], and no statistical syntheses were conducted. Findings from systematic reviews indicate that the use of CDS by healthcare practitioners may provide important benefits in terms of healthcare

practitioners' performance and patient outcomes. However, no systematic review has investigated the effects of CDS on the performance of healthcare practitioners and patient outcomes in the context of fall prevention.

Objectives

This systematic review aimed to evaluate the effects of CDS for fall prevention on healthcare practitioners' adherence to recommended practice, medication outcomes, and on patient outcomes.

Materials and methods

Protocol and registration

The review protocol was drafted and finalised according to the Preferred Reporting Items for Systematic Reviews and Meta-Analyses (PRISMA) extension for study protocols (PRISMA-P) [42] and was registered in the PROSPERO international prospective register of systematic reviews (identification number CRD42021250500) prior to commencing the systematic review. We followed the recommendations in the Cochrane Handbook [43] and the guidance from the Cochrane Effective Practice and Organisation of Care (EPOC) [44]. The reporting of this systematic review was guided by the standards of the Preferred Reporting Items for Systematic Reviews and Meta-Analyses (PRISMA) [45]. See S1 Table for the populated PRISMA checklist. See S2 Table for differences between the protocol and the review.

Eligibility criteria

We included studies on how CDS interventions directed at healthcare practitioners, with a view to supporting their decisions aiming to prevent falls in older adults in any healthcare setting, affected healthcare practitioner performance and patient outcomes. We included randomised and non-randomised controlled trials, controlled before-after studies, and interrupted time-series studies. Detailed eligibility criteria are presented in Table 1.

Table 1. Study eligibility criteria.

	Inclusion criteria	Exclusion criteria
Participants	Healthcare practitioners (nurses, physiotherapists, general practitioners, occupational therapists, nursing assistants, pharmacists, physicians, primary care providers, paramedics)	Studies of CDS used by students.
Intervention	CDS used by healthcare practitioners in fall prevention interventions. We included both computerised and non-computerised CDS interventions.	Studies where CDS was not part of the intervention in at least one study group or arm.
Comparison	Usual care or no treatment.	
Outcomes	We included studies that reported at least one primary or secondary outcome.<br>Primary:<br>• Healthcare practitioner performance: Adherence to recommended practice and adherence to recommended medication prescribing and review.<br>• Patient outcomes: Rate of falls (i.e., number of falls per unit of follow-up time); risk of falling (i.e., the number of older adults who had one or more falls); and fall injuries.<br>Secondary:<br>• Deaths and hospitalisations.	Studies that did not report at least one primary or secondary outcome.
Design	RCTs: IRPGT and CRT; NRCTs; CBA studies; and ITS studies [44].	Uncontrolled before-after, cross-sectional, case-control, and cohort studies; as well as protocols, editorials, opinion papers, and conference abstracts.<br>Studies without a control group, except for ITS studies with multiple data points collected before and after the implementation of the intervention.
Setting	Any healthcare setting or in the homes of older adults.	Studies conducted in a setting other than a healthcare institution or in the homes of older adults.

CDS: Clinical Decision Support; RCT: Randomised controlled trial; IRPGT: Individually-randomised parallel-group trial; CRT: Cluster-randomised trial; NRCT: Non-randomised controlled trial; CBA: Controlled before-after study; ITS: Interrupted time-series

Information sources and search strategy

We searched the following databases from the earliest available date through January 2025: MEDLINE (Ovid), EMBASE (Ovid), CINAHL (EBSCOhost), The Cochrane Library, Web of Science, and AMED (Ovid). Supplemental searches were performed in Google Scholar (S3 Table). The literature search strategy was developed in cooperation with two librarians from the Literature Search Resource/Expert Group at Oslo Metropolitan University (OsloMet). The searches were developed to be highly sensitive and included terms synonymous with the fundamental concepts underlying CDS (e.g., decision rules, reminder systems, algorithms), falls (e.g., accidental falls, slip), and fall prevention (e.g., accident prevention, safety management). We did not impose any language or study design restrictions on the literature searches. To maximise the sensitivity of the search and capture a broader range of potentially relevant studies, study design was not restricted in the search strategy. One reviewer (RS) used the advanced search feature in PEDro to conduct manual searches for relevant clinical trials. Additionally, we searched the reference lists of the included articles and relevant systematic reviews. Cited reference searching for all included articles was carried out in Web of Science. See S3 Table for the complete search strategy.

Selection process

Search results were imported into EndNote, where duplicates were removed. Next, all unique abstracts and full text articles were uploaded to the Covidence systematic review software

[46]. Two reviewers (RS and either TB, NRO, or LAHK) independently screened titles, abstracts, and full texts against the eligibility criteria, with any disagreements resolved by consensus or by the decision of a third reviewer. Consensus between reviewers was required during both the title and abstract screening stage and the full-text screening stage.

Data collection process

We used an adapted version of the EPOC data collection form to extract relevant data from the included studies. The data extraction form was piloted on three reports by two reviewers. One reviewer (RS) independently extracted the data, and another reviewer (TB) checked the extracted data against five arbitrarily selected papers. We resolved any disagreements between reviewers by consensus, and if necessary, a third reviewer was consulted. To address missing outcome data, we contacted the corresponding authors of eight included reports via email. If no responses were received, follow-up emails were sent after two weeks, with a maximum of three email attempts per author. Our efforts to obtain missing data were successful in four instances. All data used in the analyses were obtained directly from primary sources or through successful author contact, with no data imputation performed.

Data items

Outcomes

For healthcare practitioner performance, we sought data on adherence to recommended practice, i.e., the extent to which healthcare practitioners gave specific advice, delivered specific interventions, or followed referral guidelines [44], as well as data on medication outcomes including medication review [47] and prescribing. For patient outcomes, we

included fall risk, fall rate, and fall injury rate. Fall risk refers to the proportion of individuals who experienced at least one fall over a given period – this outcome quantifies the likelihood of falling at the person level. Fall rate and fall injury rate refer to the total number of falls or fall injuries per unit of time, e.g., per 1,000 person-years, accounting for multiple falls per individual. Included in fall injuries were both minor injuries, such as bruises or abrasions, and fractures or other serious injuries requiring medical attention [48]. We also sought data for deaths and hospitalisations. If more than one result was available from a particular study, we used the primary outcome as specified by the authors, or the one that was used for sample size calculation. If study authors did not specify primary outcomes, we prioritised results deemed to be most relevant to the research question, those indicating drug overuse rather than misuse or underuse (due to the significant fall risk imposed by polypharmacy [49]), and results based on whole-group analyses rather than subgroup analyses.

Study characteristics

We extracted the following data on study characteristics: authors, publication year, funding, country, design, setting, duration, healthcare practitioners delivering the intervention (type and number), and patients receiving the intervention (number, sex, age).

Interventions and CDS

We extracted the characteristics of experimental and control interventions using the Template for Intervention Description and Replication (TIDieR) checklist [50], and using domains 1 and 3 from the intervention complexity assessment tool for systematic reviews (iCAT_SR) [51]. Interventions were categorised into 1) manual fall risk assessment and interventions based on CDS; 2) medication review and recommendations made to physician; 3)

automatically generated fall risk based on prediction models and recommended interventions; 4) guided medication dosing using computerised CDS; and 5) computerised CDS presented to paramedics on hand-held tablets [52,53]. Design factors of CDS tools were extracted using the GUideline Implementation with DEcision Support (GUIDES) framework [53] and elements from the two-stream model [54].

Study risk of bias assessment

Two review authors independently assessed risk of bias (RoB) for outcomes indicating healthcare practitioner performance, fall rates, fall risk, and fall injuries. We used the Revised Cochrane Risk of Bias tool for randomised trials (RoB 2.0) [55] for included RCTs, and the Risk Of Bias In Non-randomised Studies of Interventions (ROBINS-I) tool [56,57] for included non-randomised studies. Any disagreements between reviewers were resolved by consensus, and if necessary, a third reviewer was consulted.

Synthesis methods and effect measures

Healthcare practitioner performance

We performed two analyses related to healthcare practitioner performance, both of which used vote-counting based on direction of effect [43,58]. In the analysis of adherence to recommended practice, we included outcomes indicating the use of recommended intervention components or adherence to the intervention protocol or to the referral guideline [44]. In the analysis of medication outcomes, we included outcomes indicating changes in the use of fall-risk-increasing drugs (FRIDs), polypharmacy, drug underuse, or drug-related problems [33]. To decide which results were eligible for the analyses, we tabulated each

study result and compared it against the eligibility criteria. Each effect estimate was dichotomised into 'favouring CDS' or 'favouring control', based on the observed direction of effect alone. We used the sign test for differences in proportions and presented the proportion of results favouring CDS along with 95% confidence intervals (CIs) using the Wilson interval method [59]. The data used for analyses of healthcare practitioner performance outcomes are available in S1 Appendix.

Patient outcomes

Data on fall risk, fall rate, and fall injury rate were pooled into meta-analyses in line with previous work [11,60]. We assumed that underlying study effects followed a normal distribution and used random effects models and restricted maximum likelihood (REML) estimation methods [61]. Stata, version 18.0, StataCorp, College Station, Texas [62], was used for all analyses and pooled results were presented in forest plots. Results were presented as odds ratios (OR) for fall risk, and as rate ratios (RaR) for fall rate and fall injury rate, along with 95% CIs. Variation between study results (heterogeneity) was assessed by means of a visual inspection of forest plots and the I^2 statistic [61]. If I^2 values were 50% or higher, we sought potential explanations for the heterogeneity using post hoc subgroup analyses by RoB (low or some concerns for RoB versus high or serious RoB), study setting (community-dwelling versus hospital or residential care), and patient age (mean age of < 80 years versus ≥ 80 years). If point estimates and confidence intervals were missing but other information was available, e.g., number of events and the follow-up time in both comparison groups, we used established formulas to estimate the point estimates and confidence intervals [63]. Only one outcome per study was included in the analyses to ensure independence between studies. The data used for meta-analyses are available in S2 Appendix. Control intervention fall risks, fall rates, and fall injury rates were derived from a US report on the epidemiology of falls

among older adults [64], due to the limited reporting of absolute numbers in the studies included in this review. To report the absolute effects on fall risk, odds ratios were first converted to relative risks using the formula provided in appendix 3 of the Core GRADE 2 article [65].

Publication bias assessment

The possibility of publication bias was assessed through inspection of funnel plots of effect estimates against their standard errors for analyses that contained at least 10 effect estimates [43]. The Egger test for small-study effects and the trim-and-fill analysis were used for quantitative assessment of publication bias [66].

Assessment of certainty of the evidence

We judged the certainty of the evidence using the Grading of Recommendations Assessment, Development and Evaluation (GRADE) approach [65,67,68], applied to each outcome from vote-counting analyses and meta-analyses. Despite extensive literature searches conducted with the assistance of two librarians at OsloMet, no minimal important difference (MID) values were identified for fall risk, fall rate, or fall injury rate. Certainty was therefore rated based on whether the true effect lies on the observed side of null, with the null effect used as the threshold. Relative and absolute effects were reported alongside plain-language statements, following recommendations from GRADE guidance [69,70].

Results

Study selection

Fig 1 presents an overview of the study selection process. Our primary database searches identified 6,572 unique records, of which 6,527 were excluded after title and abstract screening. A total of 28 publications describing 25 unique studies were selected for inclusion [25–30,40,41,71–90]. S4 Table provides a summary of the excluded studies and the reason for exclusion.

Fig 1. PRISMA flow diagram.

Study characteristics

Study design, participants, and settings

Table 2 presents an overview of the characteristics of the included studies. The 25 studies comprised 13 cluster-randomised trials (CRTs), seven individually randomised parallel group trials (IRPGTs), four non-randomised controlled trials (NRCTs), and one controlled before-after (CBA) study. The study duration was median (min–max) 12 (1–60) months. The interventions were primarily delivered by nurses (16; 64%), physicians (15; 60%), pharmacists (6; 24%), and/or physiotherapists (4; 16%) to a median number of 1,433 patient participants, ranging from 312–46,245. Twelve (48%) studies had eligibility criteria related to increased risk for falls, such as a history of previous falls or the use of fall-risk-increasing

drugs. Most studies were conducted in hospitals (44%) or in primary care practices (36%) in North American (52%) and European (28%) countries.

Table 2. Characteristics of included studies.

Author & year	Country	Design	Setting	Study duration (months)	Healthcare practitioners	Patients
Aizen 2015	Israel	CRT	Rehabilitation geriatric hospital	6	Nurses<br>n = ?	Patients admitted to hospital<br>n = 508 (200 in CDS and 308 in CG), 52.2% female, mean 84.3 year
Barker 2016	Australia	CRT	Hospital	12	Nurses<br>n = ?	Patients admitted to hospital<br>n = 35,264 (17,698 in CDS and 17,566 in CG), 49.5% female, media 67.5 years
Bhasin 2020	USA	CRT	Primary care practices	44	Nurses<br>n = ?	Community-dwelling at increased risk for fall injuries<br>n = 5,451 (2,802 in CDS and 2,649 in CG), 62% female, mean 79.7 y
Blalock 2020	USA	CRT	Community pharmacies	24	Pharmacists<br>n = ?	Adults ≥65 years using either four or more chronic medications or ≥1 medication associated with increased fall risk, n = 3,213 (1,467 in CI and 1,745 in CG)
Blum 2021	Switzerland, Netherlands, Belgium, Republic of Ireland	CRT	Hospitals	12	Physicians and pharmacists<br>n = ?	Adults years with multimorbidity (≥3 chronic conditions) and polypharmacy (≥5 drugs used long term)<br>n = 2,008 (963 in CDS and 1,045 in CG), 44.7% female, median 79 y
Byrne 2005	USA	CBA	Nursing homes	33	Nurses<br>n = 153	n = ?, ? females, Mean age in IG: 82.5 (group 1) and 80.9 (group 2); CG: 79.2 (group 5) and 82.2 (group 6)
Carroll 2012	USA	CRT	Urban hospitals (academic medical centres & community hospitals)	6	Nurses<br>n = ?	Patients admitted during study period<br>n = 364, ? female, ? age
Dykes 2010						Patients admitted during study period<br>n = 10,264 (5,160 in CDS and 5,104 in CG), 54.5% female, mean 78 years (among patients 65 years or older)
Clemson 2024	Australia	CRT	Primary care practices	12	Physicians<br>n = 75<br>Allied health professionals n = 342	Community-dwelling older adults who had had a fall in the past year were concerned about falling<br>n = 560 (275 in CDS and 285 in CG), 67.9 % female, mean 78.6 year
Dykes 2020	USA	NRCT	Hospital (academic medical centres)	42	Nurses<br>n = ?	Patients admitted during study period<br>n = 37,231 (17,948 in pre-intervention group and 19,283 in post-intervention group), 53.8% female, mean 60.8 years
Elley 2008	New Zealand	IRPGT	Primary care practices	12	Nurses<br>n = 54	Adults who had fallen in the past 12 months<br>n = 312 (155 in CDS and 157 in CG), 68.9% female, mean 80.8 year
Ferrer 2014	Spain	IRPGT	Primary care practices	24	Physicians and nurses<br>n = ?	Community-dwelling older adults born in 1924 (85 years of age at st start)<br>n = 328 (164 in CDS and 164 in CG), 61.1% female
Frankenthal 2014	Israel	IRPGT	Hospital (Chronic care geriatric facility)	12	Pharmacist<br>n = ?	Residents prescribed at least one medication<br>n = 359 (183 in CDS and 176 in CG), 66.6% female, mean 82.7 year
Gallagher 2011	Ireland	IRPGT	University hospital	6	Physicians<br>n = ?	Patients admitted via the emergency department under care of a GP<br>n = 382 (190 in CDS and 192 in CG), 53.1% female, median 74.5 ye (IG) and 77 (CG)
Ganz 2015	USA	NRCT	Primary care practices	24	Physicians, nurse practitioner, physician assistant<br>n = 44	Patients who screened positive for fall risk<br>n = 1,791 (1,187 in CDS and 604 in control), 72% female, mean 82.9 years
Wenger 2010				12		Patients who screened positive for falls or fear of falling and UI:<br>n = 1,211 (586 in CDS and 625 in CG), 71.7% female, mean 83 year

Ganz 2022	USA	CRT	Primary care practices	60	Nurses<br>n = ?	Community-living persons at increased risk for serious fall injuries<br>n = 5,451 (2,802 in CDS and 2,649 in CG), 62% female, mean 79.7 y
Groshaus 2012	Canada	NRCT	Acute care hospitals	3	Nurses<br>n = ?	Patients residing on study units<br>n = ?, ? female, ? years
Healey 2004	UK	CRT	District general hospital	12	Nurses<br>n = ?	All older adults who received care in the wards during the study peri<br>n = 1,654 (905 in CDS and 749 in CG), 60% female, mean 81.3 year
Lightbody 2002	UK	IRPGT	University hospital	6	Nurses<br>n = ?	Older adults discharged from Accident and Emergency Department a a fall<br>n = 348 (171 in CDS and 177 in CG), 74.4% female, median 75 year
Logan 2021	UK	CRT	Long-term care homes	12	Care home staff<br>n = 3,609	Long-term care home residents in care homes for older adults<br>n = 1,657 (775 in CDS and 882 in CG), 67.9% female, mean 85 year
Mahoney 2007	USA	IRPGT	Home visits	12	Registered nurse, physical therapist, physician<br>n = ?	Community-dwelling adults with two falls in the past year or one fall the previous two years with injury or balance problems<br>n = 349 (174 in CDS and 175 in CG), 78.5% female, mean 80 years
Peterson 2007	USA	IRPGT	Tertiary care hospital	9	Physicians<br>n = 778	Inpatients ≥ receiving care on one of the order entry wards (i.e., emergency room, intensive care units, subacute units)<br>n = 2,981, ? female, = years
Phelan 2024	USA	CRT	Primary care practices	18	Physicians	Community-dwelling adults aged ≥ 60 years, prescribed at least 1 medication from any of 5 targeted medication classes (opioids, sedat hypnotics, skeletal muscle relaxants, tricyclic antidepressants, and fir generation antihistamines for at least 3 consecutive months<br>n = 2,367 (1,106 in CDS and 1,261 in CG), 63% female, mean 70.6
Snooks 2014	UK	CRT	Emergency ambulance services	1	Paramedics<br>n = 42	Community-dwelling older adults living in the catchment area of a participating falls service<br>n = 779 (436 in CDS and 343 in CG), 63.4% female, median 82.5 ye
Tamblyn 2012	Canada	CRT	Primary care practices	23	GPs<br>n = 81	Patients with a prescription for a psychotropic drug<br>n = 5,628 (2,887 in CDS and 2,741 in CG), 67.1% female, mean 75.2 years
Weber 2008	USA	CRT	Primary care practices	15	Pharmacists and GPs<br>n = ?	Community-dwelling patients at risk for falls<br>n = 620 (413 in CDS and 207 in CG), 79.2% female, mean 76.9 year
Wenger 2009	USA	NRCT	Primary care practices	32	Physicians<br>n = 40	Community-dwelling patients who had at least one of three geriatric conditions: falls and gait impairment, urinary incontinence, and cogn impairment<br>n = 644 (357 in CDS and 287 in CG), 66% female, mean 81 years

CBA: Controlled Before-After study; CCDS: Computerised Clinical Decision Support; CDS: Clinical Decision Support group; CG: Control group; CRT: Cluster-Randomised Trial; ED: Emergency Department; GP: General Practitioner; IG: Intervention Group; ITS: Interrupted Time-Series study; IRPGT: Individually-Randomised Parallel-Group Trial; NRCT: Non-Randomised Controlled Trial; UI: Urinary Incontinence

Interventions and CDS tools

Table 3 presents an overview of the characteristics of the interventions and CDS tools assessed in the included studies. Most (15; 60%) interventions delivered CDS to healthcare practitioners to aid with fall risk assessments, followed by an offer to the patient of specific fall prevention interventions. A total of 22 (88%) interventions consisted of more than one component delivered as a bundle, i.e., there was a defined order in the delivery of the components such as medication review prior to medication changes. Most interventions were directed at either one type of healthcare practitioner (12; 48%) or at two or more types of healthcare practitioners in the same healthcare setting (11; 44%). See S5 Table for a detailed description of the interventions. Most CDS tools were designed as algorithms presented as if-then-statements or as checklists with risk factors for falls, followed by recommendations for fall prevention interventions that were either generated automatically or chosen manually from a list (68%). Fifteen (60%) of the CDS tools were delivered electronically, three (12%) were delivered both electronically and on paper, two (8%) were paper-based, and for five (20%) studies, this remained unclear. Among the electronically delivered CDS interventions, nine (36%) included alerts, reminders, or prompts, whereas in the remaining studies, decision support was delivered on demand. The control interventions included usual care (76%), usual care plus a falls information pamphlet (4%), usual care plus an offer of two social visits (4%), home safety visits (4%), paper-based CDS (4%), and no treatment (4%). One study (4%) failed to mention control. See S6 Table for a detailed description of the CDS design factors.

Table 3. Characteristics of interventions and Clinical Decision Support.

Study	Type of intervention	Active components	n types of intervention deliverers	Organisational levels	CDS format	
Aizen 2015	1	High	1: Nurses	Low	Electronic	Risk assessment recommendatio
Barker 2016	1	High	3: Nurse, site clinical leader, champion	Intermediate	-	Checklist with r interventions; R intervention con

Bhasin 2020, Ganz 2022	1	High	3: Nurse, primary care physician, pharmacist	Intermediate	Electronic	Algorithm with recommendatio
Blalock 2020	2	High	2: Pharmacist, primary care physician	Intermediate	Electronic	Adapted STEAI and interventio
Blum 2021	2	High	3: Pharmacist, hospital physician, general practitioner	High	Electronic	Lists of FRIDs (
Byrne 2005	3	High	1: Nurse	Low	Electronic	Automatically g and interventio
Carroll 2012, Dykes 2010	1	High	1: Nurse	Low	Electronic and paper-based	Alerts printed o
Clemson 2024	1	High	> 7: Primary care physicians, physiotherapists, occupational therapists, nurses, podiatrists, pharmacists, exercise physiologists, and other professions	High	Electronic and paper-based	iSOLVE algorit checklist; GP fa Tailoring interv information aut studies which il tailoring option with patients ab
Dykes 2020	1	High	1: Nurse	Low	Electronic	Checklist with r interventions
Elley 2008	1	High	3: Nurse, trained practitioner, physiotherapist	Intermediate	-	Algorithm with recommendatio
Ferrer 2014	1	High	2: Nurse, primary care physician	Intermediate	-	Algorithm with recommendatio
Frankenthal 2014	2	High	2: Pharmacist, hospital physician	Intermediate	Electronic	Lists of FRIDs (
Gallagher 2011	2	High	1: Hospital physician	Low	-	Lists of FRIDs (
Ganz 2015, Wenger 2010	1	High	3: Primary care physician, nurse, physician assistant	Intermediate	Electronic	Medical record
Groshaus 2012	1	High	1: Nurse	Low	Electronic	Electronic orde
Healey 2004	1	High	1: Nurse	Low	Paper-based	Checklist with r recommendatio
Lightbody 2002	1	High	1: Nurse	Low	-	Checklist with r recommendatio
Logan 2021	1	High	3: Nurse, physiotherapist, occupational therapist	Intermediate	Paper-based	Checklist with r recommendatio
Mahoney 2007	1	High	3: Nurse, physiotherapist, primary care physician	Intermediate	Electronic	Algorithm with intervention rec
Peterson 2007	4	Low	1: Hospital physician	Low	Electronic	Guided medica on-screen
Phelan 2024	2	Intermediate	1: Primary care physician	Low	Electronic	Evidence-based Deprescribing p starters
Snooks 2014	5	High	1: Paramedic	Low	Electronic	Prompts to start automatically g
Tamblyn 2012	4	Low	1: Primary care physician	Low	Electronic	Predictive mode risk of injury; A prescribed a FR estimates; Guid
Weber 2008	2	High	3: Pharmacist, geriatrician, primary care physician	Intermediate	Electronic	Guided medica patient's fall ris
Wenger 2009	1	High	3: Nurse, primary care physician, medical assistant	Intermediate	Electronic and paper-based	Medical record appropriate acti

CDS: Clinical Decision Support; FRIDs: Fall-risk-increasing drugs; GP: General practitioner; STEADI: Stopping Elderly Accidents, DEaths, and Injuries; STOPP/START: Screening Tool of Older Person's Prescriptions and Screening Tool to Alert doctors to Right Treatment

Type of intervention:

1: Fall risk assessment and interventions based on CDS.

2: Medication review and recommendations made to physician.

3: Automatically generated fall risk based on prediction models, followed by recommended interventions.

4: Guided medication dosing using computerised CDS.

5: Computerised CDS presented to paramedics on hand-held tablets.

Active components (iCAT_SR domain 2):

High: More than one component and delivered as a bundle (clear order in the delivery of the components) (high level of complexity).

Intermediate: More than one component and delivered as a package (no specific order) (intermediate level of complexity).

Low: One component (low level of complexity).

Organisational levels and categories targeted by the intervention (iCAT_SR domain 3):

High: Intervention directed at two or more healthcare settings, e.g., primary care and hospitals (multi-level).

Intermediate: Intervention directed at two or more categories of healthcare practitioners within the same healthcare setting, e.g., nurse and physiotherapist in primary care (multi-category)

Low: Intervention directed at one category of healthcare practitioner, e.g., nurses (single category)

Risk of bias in included studies

A detailed description of RoB assessments is available in S7 Table. We assessed a total of 40 results across five outcomes. Out of 33 results from randomised trials, 19 results had high RoB, 10 results had some concerns, and four results had low RoB. All seven results from NRSI were judged to be at serious RoB, primarily owing to concerns for bias due to confounding.

Results of individual studies

Results from each individual study are available in S8 Table.

Healthcare practitioner performance

Adherence to recommended practice

Five CRTs [26,27,30,71,88] and three NRCTs [80,82,90] reported results on adherence to recommended practice (Fig 2). The common outcome across the various studies is the adherence to fall prevention interventions, including fall risk documentation, protocol adherence, and provision of recommended intervention components. The follow-up time was median (min–max) 7 (1–13) months. All eight results favoured the intervention (100% (95% CI 68% to 100%), $p < 0.01$). The certainty of evidence was low due to risk of bias (Table 4). CDS may improve healthcare practitioners' adherence to recommended practice in fall prevention.

Fig 2. Healthcare practitioner performance results.

FU: Follow-up; IQR: Interquartile range; DBI: Drug Burden Index; IG: Intervention group; CG: Control group; RoB: Overall risk of bias judgement.

† Based on subgroup analysis and therefore not randomised

* Non-randomised study. Serious risk of bias based on ROBINS-I

Sign test:

H0: n results favouring CDS = n results favouring control

Ha: n results favouring CDS ≠ n results favouring control

Table 4. Summary of findings.

CDS interventions compared with usual care for HCPs in fall prevention among older adults

Population: HCPs, including nurses, physiotherapists, general practitioners, occupational therapists, nursing assistants, pharmacists, physicians, primary care providers, and paramedics
Settings: Hospitals, residential care, primary care, and the homes of older adults
Intervention: CDS targeted at HCPs
Comparison: Usual care

**Outcomes**<br><br>**No of participants (studies)**	**Relative effects (95% CI)**	**Anticipated absolute effects* (95% CI)**			**Certainty of evidence**
		**In control**	**CDS interventions**	**Difference**	
**Adherence to recommended practice: fall risk assessments and interventions**<br><br>Follow-up: median 7 (1 to 13) months<br><br>[a] (8)	[b]	[b]	[b]	[b]	⊕⊕OO<br><br>Low[c]<br><br>Due to very serious risk of bias
**Adherence to recommended medication review and prescribing**<br><br>Follow-up: median 9 (0 to 23) months<br><br>[a] (9)	[b]	[b]	[b]	[b]	⊕⊕⊕O<br><br>Moderate[d]<br><br>Due to risk of bias
**Fall risk**<br><br>Follow-up: median 9 (1 to 24) months<br><br>> 13,636[e] (10)	OR 0.93 (0.85 to 1.01)	287 per 1,000	273 per 1,000 (255 to 290)	14 fewer fallers per 1,000 (32 fewer to 3 more)	⊕⊕OO<br><br>Low[f]<br><br>Due to risk of bias and imprecision
**Rate of falls**					
In hospitals or residential care<br><br>Follow-up: median 6 (3 to 21) months<br><br>> 50,054[e] (8)	RaR 0.74 (0.63 to 0.88)	672 per 1,000 person-years	497 per 1,000 person-years (423 to 591)	175 fewer falls per 1,000 person-years (249 fewer to 81 fewer)	⊕⊕⊕O<br><br>Moderate[g]<br><br>Due to risk of bias

In community-dwelling older adults<br><br>Follow-up: median 12 (12-24) months<br><br>7,000 (5)	RaR 0.97 (0.93 to 1.00)	672 per 1,000 person-years	652 per 1,000 person-years (625 to 672)	20 fewer falls per 1,000 person-years (47 fewer to 0 fewer)	⊕⊕OO<br><br>Low[h]<br><br>Due to risk of bias and imprecision
In patients with mean age ≥ 80 years<br><br>Follow-up: median 12 (3-12) months<br><br>6,264 (7)	RaR 0.72 (0.61 to 0.86)	672 per 1,000 person-years	484 per 1,000 person-years (410 to 578)	188 fewer falls per 1,000 person-years (262 fewer to 94 fewer)	⊕⊕⊕O<br><br>Moderate[i]<br><br>Due to risk of bias
In patients with mean age between 65 and 80 years<br><br>Follow-up: median 12 (6-24) months<br><br>84,084 (6)	RaR 0.92 (0.84 to 1.01)	672 per 1,000 person-years	618 per 1,000 person-years (564 to 679)	54 fewer falls per 1,000 person-years (108 fewer to 7 more)	⊕⊕OO<br><br>Low[j]<br><br>Due to risk of bias and imprecision
**Rate of fall injuries**					
In patients with mean age between 65 and 80 years<br><br>Follow-up: median 16.5 (6-24) months<br><br>> 50,979[e] (4)	RaR 0.80 (0.59 to 1.09)	164 per 1,000 person-years	131 per 1,000 person-years (97 to 179)	33 fewer fall injuries per 1,000 person-years (67 fewer to 15 more)	⊕⊕OO<br><br>Low[k]<br><br>Due to risk of bias and imprecision
In patients with mean age ≥ 80 years<br><br>Follow-up: median 9 (6-12) months<br><br>3,445 (2)	RaR 1.29 (0.99 to 1.69)	164 per 1,000 person-years	212 per 1,000 person-years (162 to 277)	48 more fall injuries per 1,000 person-years (2 fewer to 113 more)	⊕OOO<br><br>Very low[l]<br><br>Due to very serious risk of bias and imprecision

* Assuming a control group fall risk of 28.7%, a fall rate of 672 falls per 1,000 person-years, and a fall injury rate of 164 fall injuries per 1,000 person-years, based on data from Bergen et al. [64]. The risk in the intervention group is based on the assumed risk in the comparison group and the relative effect of the intervention (and its 95% CI).

CDS: clinical decision support; CI: confidence interval; HCPs: healthcare practitioners; OR: odds ratio; RR: rate ratio

Explanations

[a] The numbers of participating healthcare practitioners were not reported.

[b] Not estimable due to the use of different and incompletely reported effect measures across studies.

[c] Very serious risk of bias due to confounding, deviations from intended interventions, and selection of reported results.

[d] Risk of bias due to deviations from intended interventions.

[e] The number of participants were not reported in all studies.

[f] Risk of bias due to the randomisation process, deviations from intended interventions, and measurement of the outcome. Imprecision due to the upper bound of the 95% confidence interval crossing the null effect.

[g] Risk of bias in the randomisation process and due to deviations from intended interventions.

[h] Risk of bias due to deviations from intended interventions. Imprecision due to the upper bound of the 95% confidence interval including the null effect.

[i] Risk of bias due to deviations from intended interventions and selection of reported results.

[j] Risk of bias due to the randomisation process, confounding, and deviations from intended interventions. Imprecision due to the upper bound of the 95% confidence interval crossing the null effect.

[k] Risk of bias due to confounding deviations from intended interventions. Imprecision due to the upper bound of the 95% confidence interval crossing the null effect.

[l] Very serious risk of bias due to the randomisation process and confounding. Very serious imprecision arises because the 95% confidence interval includes both an effect favouring CDS and the possibility of appreciable harm.

Adherence to recommended medication review and prescribing

Five CRTs [25,41,72,74,89] and four IRPGTs [77,78,84,87] reported indicators of medication outcomes (Fig 2). The common outcome across the various studies was the reviewing and prescribing of drugs that may increase fall risk. The follow-up time was 9 (0–23) months. All nine results favoured the intervention (100% (95% CI 70% to 100%), $p < 0.01$). The certainty of evidence was moderate due to risk of bias. CDS likely improves medication reviewing and prescribing outcomes in fall prevention.

Patient outcomes

Fall risk

Nineteen of the included studies reported outcome data on fall risk (proportion of participants who fell) and/or fall rate (number of falls per x person-years), which made it possible to conduct meta-analyses on these outcomes. Ten studies were included in the meta-analysis on fall risk with a median (min–max) follow-up time of 9 (1–24) months [25,29,74,76,78,81,82,84,88,89] (Fig 3). Of these studies, six were conducted in patients aged 65 to 80 years, three in patients aged 80 years or older, and one in which the patients' ages were unknown. Furthermore, five studies were conducted in hospitals or residential care, while five focused on community-dwelling older adults. The overall estimate of the effect of CDS on fall risk was an odds ratio of 0.93 (95% CI 0.85 to 1.01). Assuming a baseline fall risk of 28.7% [64], a 7% reduction in the odds of falling results in 14 fewer fallers per 1,000 (95% CI 32 fewer to 3 more) in older adults receiving the intervention compared with control, during nine months of follow-up (Table 4). Heterogeneity was unimportant ($I^2 = 0\%$; $p = 0.19$). Visual inspection of the funnel plot showed no clear sign of small-study effects (S1 Fig). The Egger test suggested no evidence of small-study effects ($p = 0.89$). The trim-and-

fill analysis imputed no studies and therefore made no difference in the effect estimate (S2 Fig). The certainty of the evidence was low due to risk of bias and imprecision. CDS aimed at healthcare practitioners may reduce fall risk, but the effect may be small.

Fig 3. Meta-analysis comparing CDS interventions with control on fall risk.

Red dashed line shows the point estimate for the meta-analysis overall.

Risk of Bias legend

Circles: Green: Low risk of bias (RoB); Yellow: Some concerns for RoB; Red: High RoB; Orange: Serious RoB.

1a: Bias arising from the randomisation process.

1b: Bias arising from the timing of identification or recruitment of individual participants within clusters (for cluster-randomised only).

2: Bias due to deviations from intended interventions.

3: Bias due to missing outcome data.

4: Bias in measurement of the outcome.

5: Bias in selection of the reported result.

O: Overall RoB judgement.

* Non-randomised study. ROBINS-I was used to assess RoB.

Rate of falls

Thirteen studies were included in the meta-analysis on fall rate [26,28–30,40,71,76,77,81,83–86] (Fig 4). Substantial heterogeneity was present in the effect estimate ($I^2 = 74\%$; $p < 0.01$). Subgroup analyses revealed statistically significant differences in fall rates when grouped by

study setting ($p < 0.01$) and by patients' age ($p < 0.01$), and results are presented separately by study setting and by patients' age.

Regarding subgroup analysis by study setting, the median (min–max) follow-up time was 6 (3–21) months for studies conducted in hospitals or residential care, and 12 (12–24) months for studies in community-dwelling older adults. Of the eight studies conducted in hospitals or residential care, four included patients aged 80 years or older, and four included patients aged 65 to 80 years. Similarly, of the five studies focusing on community-dwelling older adults, three included patients aged 80 years or older, while two included patients aged 65 to 80 years. The overall estimate of the effect of CDS on fall rate in hospitals or residential care was a rate ratio of 0.74 (95% CI 0.63 to 0.88; $I^2 = 65\%$; $p < 0.01$). Assuming a baseline fall rate of 672 falls per 1,000 person-years [64], a 26% reduction in fall rate results in 175 fewer falls per 1,000 person-years (95% CI 249 fewer to 81 fewer) in hospitals or residential care settings (Table 4). The certainty of the evidence was moderate due to risk of bias. CDS aimed at healthcare practitioners likely reduces the rate of falls in hospitals and residential care. The substantial heterogeneity ($I^2 = 65\%$) suggests that the observed effects may vary across studies. In community-dwelling older adults, the overall estimate of the effect on fall rate was a rate ratio of 0.97 (95% CI 0.93 to 1.00; $I^2 = 0\%$; $p = 0.88$). The certainty of the evidence was low due to risk of bias and imprecision. CDS may reduce fall rate in community-dwelling older adults, though the effect may be small.

Regarding the subgroup analysis by patients' age, the median (min–max) follow-up time was 12 (3–12) months for studies involving patients with a mean age ≥ 80 years, and 12 (6–24) months for studies of patients with a mean age between 65 and 80 years (S3 Fig). Of the six studies including patients aged 65 to 80 years, four were conducted in hospitals or residential

care, while two focused on community-dwelling older adults. Of the seven studies including patients aged 80 years or older, four were conducted in hospitals or residential care, while three focused on community-dwelling older adults. The overall estimate of the effect of CDS on fall rate among patients with a mean age of ≥ 80 years was a rate ratio of 0.72 (95% CI 0.61 to 0.86; I^2 = 46%; p = 0.09). Assuming a baseline fall rate of 672 falls per 1,000 person-years [64], a 28% reduction in fall rate results in 188 fewer falls per 1,000 person-years (95% CI 262 fewer to 94 fewer). The certainty of the evidence was moderate due to risk of bias. CDS likely reduces the rate of falls among patients with a mean age of ≥ 80 years. In patients with a mean age between 65 and 80 years, the overall estimate of the effect on fall rate was a rate ratio of 0.92 (95% CI 0.84 to 1.01; I^2 = 31%; p = 0.09). The certainty of the evidence was low due to risk of bias and imprecision. CDS may reduce fall rate in patients with a mean age between 65 and 80 years, but the effect may be small. Visual inspection of the funnel plot showed no clear sign of small-study effects (S4 Fig). The Egger test suggested no evidence of small-study effects (p = 0.99). The trim-and-fill analysis imputed no studies and therefore made no difference in the effect estimate (S5 Fig). There was no statistically significant subgroup difference in fall rate when stratified by RoB (p = 0.47) (S6 Fig).

Fig 4. Meta-analysis comparing CDS interventions with control on fall rate, subgroup analysis by study setting.

Red dashed line shows the point estimate for the meta-analysis overall.

<u>Risk of Bias legend</u>

Circles: Green: Low risk of bias (RoB); Yellow: Some concerns for RoB; Red: High RoB; Orange: Serious RoB.

1a: Bias arising from the randomisation process.

1b: Bias arising from the timing of identification or recruitment of individual participants within clusters (for cluster-randomised only).

2: Bias due to deviations from intended interventions.

3: Bias due to missing outcome data.

4: Bias in measurement of the outcome.

5: Bias in selection of the reported result.

O: Overall RoB judgement.

* Non-randomised study. ROBINS-I was used to assess RoB.

Rate of fall injuries

Six studies were included in the meta-analysis on fall injury rate [26,28,30,79,81,83] (Fig 5). Substantial heterogeneity was present in the effect estimate (I^2 = 71%, p = 0.03). Subgroup analyses revealed a statistically significant difference in fall injury rate between patients with a mean age between 65 and 80 years and patients with a mean age of 80 years or older (p = 0.02), and results are presented separately by patients' age. The median (min–max) follow-up time was 16.5 (6–24) months for studies conducted in patients with a mean age between 65 and 80 years and 9 (6–12) months for studies in patients with a mean age of 80 years or older. The overall estimate of the effect of CDS in preventing fall injuries in patients with a mean age between 65 and 80 years was a rate ratio of 0.80 (95% CI 0.59 to 1.09; I^2 = 67%; p = 0.08), which indicates a statistically non-significant result as the confidence interval crosses the null effect. Assuming a baseline fall injury rate of 164 fall injuries per 1,000 person-years [64], a 20% reduction in fall injury rate results in 33 fewer fall injuries per 1,000 person-years (95% CI 67 fewer to 15 more) in older adults receiving the intervention compared with control (Table 4). The certainty of the evidence was low due to risk of bias and imprecision.

CDS interventions aimed at healthcare practitioners may reduce the fall injury rate in patients with a mean age between 65 and 80 years.

Fig 5. Meta-analysis comparing CDS interventions with control on fall injury rate, subgroup analysis by patients' age.

Red dashed line shows the point estimate for the meta-analysis overall.

Circles: Green: Low risk of bias (RoB); Yellow: Some concerns for RoB; Red: High RoB; Orange: Serious RoB.

1a: Bias arising from the randomisation process.

1b: Bias arising from the timing of identification or recruitment of individual participants within clusters (for cluster-randomised only).

2: Bias due to deviations from intended interventions.

3: Bias due to missing outcome data.

4: Bias in measurement of the outcome.

5: Bias in selection of the reported result.

O: Overall RoB judgement.

* Non-randomised study. ROBINS-I was used to assess RoB.

In patients with a mean age of 80 years or older, the overall estimate of the effect on fall injury rate was a rate ratio of 1.29 (95% CI 0.99 to 1.69; $I^2 = 0\%$; $p = 0.84$). The certainty of the effect estimate of CDS interventions on fall injury rate in patients with a mean age of 80 years or older is very low due to risk of bias and imprecision. We are very uncertain about the effect of CDS on fall injury rates in patients aged 80 years or older. There is no statistically

significant subgroup difference in fall injury rate when stratified by RoB (p = 0.93) or study setting (p = 0.41). See S7 and S8 Figs for subgroup analyses on fall injury rate.

Mortality and hospitalisations

Five CRTs [72–74,86,88] reported on mortality, and five IRPGTs [76–78,84,85] and three CRTs [73,74,88] reported hospital admissions. The sample sizes were small, the outcomes were rare, and no consistent patterns were observed in the direction of effect for mortality or hospital admissions. For further details on individual study results, see S8 Table.

Certainty of the evidence

Certainty of the evidence was assessed for nine outcomes, the results from which are available in a GRADE evidence profile in S9 Table and in Table 4.

Discussion

This systematic review summarised 25 studies (28 publications) of healthcare professionals' use of CDS in fall prevention and the effects on healthcare practitioner performance and patient outcomes. The interventions were delivered by nurses, physicians, physiotherapists, pharmacists, occupational therapists, and paramedics. Regarding the effects on healthcare practitioner performance, CDS likely improves fall risk assessments and the provision of recommended interventions and may improve medication review and prescribing. Regarding the effects on patient outcomes, CDS likely decreases the rate of falls in hospitals and residential care settings and in patients aged 80 years or older. Furthermore, CDS may reduce

fall rates in community-dwelling older adults and in patients aged 65 to 80 years, but the effects in these subgroups may be small. CDS interventions likely reduce fall risk slightly, and may reduce the rate of fall injuries in patients aged between 65 and 80 years. Finally, CDS may reduce fall injuries in adults aged 65 to 80 years, but the effect on fall injuries in adults aged 80 years or older remains very uncertain.

Interventions

A common feature of all experimental interventions included in this review is that they delivered decision support to healthcare practitioners aiming to prevent falls in older adults. The control interventions were generally poorly described as consisting of usual care, with different interventions depending on the setting or the type of healthcare practitioner. Most of the experimental interventions utilised CDS to aid healthcare practitioners in conducting risk assessments and implementing preventive measures across multiple domains such as gait and balance problems, environmental factors, and medications, as opposed to focusing on a single domain. Multifactorial fall prevention interventions have been shown effective in reducing falls [60], and using CDS when delivering such complex interventions may offer healthcare practitioners structure and guidance [91]. However, it is important to note that, even though most of the interventions provided CDS electronically, only 36% of the CDS tools automatically delivered recommendations to the healthcare practitioners. A systematic review found that providing CDS automatically versus on demand may lead to large improvements in adherence [23].

Healthcare practitioner performance

Our findings are consistent with previous systematic reviews reporting improvements in adherence to recommended practice [32,34] and medication outcomes [33]. Mebrahtu et al. [31] also found favourable effects in healthcare practitioner performance associated with the use of CDS, including improvements in nurses' adherence to hand disinfection guidance, insulin dosing, on-time blood sampling, and documentation of care. Kwan et al. [34] reported that CDS increased the proportion of patients receiving recommended care. Yourman et al. [33] found that, in most cases, CDS improved medication outcomes. Although improvements were mostly moderate, these results demonstrate the diversity of clinical areas in which CDS may be beneficial.

Patient outcomes

Our meta-analyses indicated that CDS interventions may prevent falls among 14 per 1,000 older adults during a follow-up period of nine months (with a baseline risk of 28.7% and a 7% risk reduction) and may prevent 175 falls per 1,000 person-years (with a baseline rate of 672 falls per 1,000 person-years and a 26% fall rate reduction). The studies included in the meta-analysis on fall risk represent a range of age groups and care settings, suggesting that the findings are broadly applicable to the population of older adults at risk of falls. There may be several explanations for the finding that CDS interventions have seemingly different effects on the risk versus the rate of falls. Dautzenberg et al. [13] hypothesised that fall rate may measure the risk of falls more accurately than the number of fallers. The outcome 'fall risk' counts the number of persons who fall, whether once or repeatedly, as a single outcome event. Therefore, repeated falls by the same person do not contribute additional events or data beyond the initial fall. In contrast, the outcome 'rate of falls' counts each individual fall as a

separate outcome event. For example, a person who falls five times during the follow-up period would contribute five events to the ‘rate of falls’ outcome but only one event to the ‘fall risk’ outcome. Consequently, ‘fall risk’ does not capture changes in the frequency of falls among older adults. It may be that the interventions included in our study were more effective at reducing the frequency of falls among those with the highest fall risk, i.e., recurrent fallers, than among one-time fallers. Such a reduction in the frequency of falls among recurrent fallers would result in a larger reduction in the rate of falls compared to fall risk. In agreement with our findings, earlier systematic reviews [13,92] reported that multifactorial interventions were associated with a reduction in fall rate but with a smaller reduction in fall risk.

The studies included in the meta-analyses on fall rate encompass a range of age groups and care settings, suggesting that the findings are broadly applicable to the entire study population. There are several potential explanations for the difference in fall rate reduction between settings, i.e., a 26% fall rate reduction in hospitals and residential care versus a 3% fall rate reduction in community-dwelling older adults. Older adults in hospitals and care homes are at high risk of falls [16], and interventions may be more effective in high-risk patients than in low-risk patients [93,94]. Therefore, interventions directed at high-risk patients will show larger absolute effects than interventions directed at low- to moderate-risk patients. Furthermore, CDS tools may be more readily implemented in hospital and residential care settings than in primary care due to the complexity of the setting [95], potentially increasing healthcare practitioners’ fidelity to the intervention protocol. Another possible explanation is differences in follow-up time in the included studies. The median follow-up time was six months for studies conducted in hospitals and residential care, and 12 months for studies in community-dwelling older adults. The effect of fall prevention

interventions may dwindle as time passes [96], presumably because of patients ceasing to participate in the fall prevention programme in which they participated during the study, such as physical exercise [16]. However, we opted not to conduct a subgroup analysis by follow-up time to minimise the risk of type I errors, which may have led to false positive results. Moreover, while our results indicate a 26% reduction in fall rate in hospitals and residential care, the substantial heterogeneity (I^2 = 65%) suggests that the observed effects likely vary across studies, potentially due to differences in study populations, settings, or intervention delivery. Despite this variability, CDS interventions targeting healthcare practitioners likely reduce fall rates in hospitals or residential care, though the effect size may depend on the context.

The certainty of the effects on fall injuries is low for patients aged 65 to 80 years and very low for patients aged 80 years or older, due to risk of bias and imprecision. Given that the outcome measure is a rate ratio and not a risk ratio, the width of the CIs surrounding the point estimate is calculated based on the number of events and not the number of participants. Therefore, the wide CIs around the point estimates for fall injury rate may be explained by the fact that fall injuries are somewhat rare when compared with falls [64]. Lastly, none of the studies included in this systematic review reported any negative effects attributable to the CDS interventions. Our findings are consistent with previous systematic reviews [31,33]. For instance, Yourman et al. found that, although CDS may cause errors in medication prescribing, in most cases, CDS helped to reduce side-effects and improve patient safety [33].

When implementing innovations into clinical practice, both high-income countries (HIC) and low- and middle-income countries (LMIC) face barriers related to political, social, and cultural factors, as well as resources and healthcare practitioner-related factors [97,98]. While

the studies included in this review were conducted in HIC, LMIC face additional barriers, such as physical challenges like unreliable power supplies and poor internet connectivity, limited access to electronic health records and computers, and human resources constraints, including overburdened staff and lack of formal training for healthcare workers [98]. This limited digital infrastructure could hinder the integration of CDS tools, which often rely on access to electronic health records and digital systems [91]. To address these challenges, CDS systems need to be adaptable to LMIC contexts, such as through offline functionality or paper-based versions. Additionally, CDS tools should be user-friendly to minimise the burden on already overworked staff.

A notable aspect of this study is the absence of established MID values for fall risk, fall rate, and fall injury rate. We assessed certainty based on whether the true effect lies on the observed side of the null, with the null effect used as the threshold. While some of the findings in this review suggest that the effects of CDS may be small, we did not determine what constitutes a clinically important effect, as this often depends on the context and setting.

What is considered an important effect (or MID), and therefore what is worth the effort of implementation, can vary significantly between settings. In HIC with robust healthcare infrastructure, even small reductions in fall risk or fall injuries may justify the use of CDS interventions. Conversely, in LMIC contexts, where resources such as computers, internet access, and trained personnel are limited, the threshold for what constitutes an important difference may be higher. In such settings, interventions with larger impacts may be prioritised to promote the best use of limited resources. Context-specific thresholds could enable a more nuanced evaluation of treatment effects and help guide decisions on whether CDS interventions are worth the effort required for implementation.

Implications

Given that many of the included studies targeted participants with increased fall risk, e.g., previous fallers or those with specific risk factors, the findings of this review are likely most applicable to populations at a higher risk of falls. Our estimates indicate that, on average, CDS interventions directed at healthcare practitioners may prevent 14 older adults per 1,000 from falling, with a 28.7% baseline fall risk. CDS interventions may also prevent 175 falls per 1,000 person-years in hospitals and residential care as well as prevent 188 falls per 1,000 person-years in adults aged 80 years and older, assuming a baseline fall rate of 672 falls per 1,000 person-years. However, further research is needed to better understand the significant differences in fall rate reduction between hospitals or residential care settings and community-dwelling older adults. Future studies should focus on identifying the factors that contribute to the greater effectiveness of interventions in hospitals and residential care and explore ways to adapt these strategies for community-dwelling older adults. This systematic review provides valuable insight that can be used when planning the use of CDS tools in fall prevention efforts among older adults. Directing interventions at high-risk patients may offer greater benefits than directing the interventions at low- to moderate-risk patients. However, CDS tools should be designed to automatically provide decision support on-screen instead of paper-based decision support provided on demand [23]. Only 36% of the CDS interventions in this review met these criteria and herein lies a potential to further improve patient outcomes, adherence to recommended practice, and medication outcomes.

Strengths and limitations

To our knowledge, this is the first systematic review to provide an overview of the effects of CDS used by healthcare practitioners in fall prevention among older adults. A major strength of this review is the thorough and sensitive literature search strategy. Potential non-reporting biases, poor indexing, and other factors make it impossible to know whether all relevant studies were in fact identified [43]. We therefore searched the reference lists of included articles and relevant systematic reviews. Also, the funnel plots along with the Egger tests and trim-and-fill analyses suggested no evidence of publication bias for fall risk or fall rate. This indicates that we have likely included most relevant studies. Furthermore, outcomes indicating healthcare practitioner performance were reported so varyingly among the studies that meta-analyses were not possible. We consider it a strength that we conducted vote-counting based on direction of effect instead of relying solely on textual descriptions of results [43]. Limitations of vote-counting include that the method provides no information on the magnitude of effects and does not account for differences in the relative sizes of the studies. Still, vote-counting enables a statistical analysis of whether there is any evidence of effect of the intervention. Also, this method may be superior to a narrative description, in which some results are privileged above others without appropriate justification [43]. Moreover, we performed meta-analyses on fall outcomes and used the GRADE approach with explicit judgements of the certainty of the evidence [99].

A limitation of this review is that verification of the collected data by a second reviewer was performed for only five of 25 included studies, raising concerns about potential errors during data collection. However, the selection of each result from each study was discussed and verified with the project statistician to ensure correctness and consistency. Another limitation of this review is the uncertainty surrounding the specific effects of different CDS tools. For

example, while some tools delivered decision support to healthcare practitioners conducting multifactorial risk assessments and interventions [40,86], other tools focused solely on guided medication dosing [41,87]. This variation in tool types and use cases makes it difficult to assess what effects can be expected from different tools or whether certain tools have any measurable impact at all. Furthermore, a limitation of the studies included in this review is that most results were judged to be at high or serious risk of bias. All the results from the included non-randomised studies were at risk of bias due to confounding. The lack of randomisation increases the risk of unequal distribution of confounding factors between intervention groups, as group assignment may be influenced by knowledge of prognostic factors. Moreover, none of the included non-randomised studies employed analysis methods that adequately controlled for all important confounders, such as age, a history of falls, sex, or gait and balance impairments. The direction and magnitude of this confounding, however, is not known. This makes it difficult to judge whether the effect estimates are greater or lower than the true effect. For the randomised studies, the main concerns for risk of bias were related to deviations from intended interventions, such as crossover effects in Peterson et al. [87] and intervention contamination in Clemson et al. [71].

Conclusions

CDS likely improves the performance of healthcare practitioners in fall prevention for older adults. CDS probably reduce falls and may reduce fall injury rates, but the effects can vary across different subgroups of older adults. This systematic review contributes valuable understanding of the effects of CDS directed at healthcare practitioners in the implementation of fall prevention among older adults. Prioritising resources and directing interventions at high-risk patients may offer the greatest effects in terms of preventing falls, but the

interventions should be long-lasting and CDS tools should be designed to improve adherence to recommended practice. Future research focusing on fall injuries should strive to improve precision by increasing the number of participants and the length of follow-up. In addition, meta-analyses of healthcare practitioner performance outcomes would become possible with more uniform and detailed reporting of results. Finally, the design of CDS tools may positively or negatively affect healthcare practitioners' adherence to recommended practice, however, additional research is needed to identify which specific design elements of CDS tools contribute to successful outcomes in fall prevention.

Acknowledgements

The authors are grateful to help provided by Elisabeth Karlsen and Kari Kalland (OsloMet) for electronic database searches.

References

1. Elias Filho J, Borel WP, Diz JBM, Barbosa AWC, Britto RR, Felício DC. Prevalence of falls and associated factors in community-dwelling older Brazilians: a systematic review and meta-analysis. Cad Saude Publica. 2019;35: 1–16. doi:10.1590/0102-311X00115718
2. James SL, Lucchesi LR, Bisignano C, Castle CD, Dingels Z V, Fox JT, et al. The global burden of falls: global, regional and national estimates of morbidity and mortality from the Global Burden of Disease Study 2017. Injury Prevention. 2020;26: i3–i11. doi:10.1136/injuryprev-2019-043286
3. Haagsma JA, Olij BF, Majdan M, van Beeck EF, Vos T, Castle CD, et al. Falls in older aged adults in 22 European countries: incidence, mortality and burden of disease from 1990 to 2017. Injury Prevention. 2020;26: i67–i74. doi:10.1136/injuryprev-2019-043347
4. Bergland A, Pettersen AM, Laake K. Falls reported among elderly Norwegians living at home. Physiotherapy Research International. 1998;3: 164–174. doi:10.1002/pri.138
5. Hektoen LF, Saltvedt I, Sletvold O, Helbostad JL, Lurås H, Halsteinli V. One-year health and care costs after hip fracture for home-dwelling elderly patients in Norway:

Results from the Trondheim Hip Fracture Trial. Scand J Public Health. 2016;44: 791–798. doi:10.1177/1403494816674162

6. Davis JC, Robertson MC, Ashe MC, Liu-Ambrose T, Khan KM, Marra CA. International comparison of cost of falls in older adults living in the community: a systematic review. Osteoporosis International. 2010;21: 1295–1306. doi:10.1007/s00198-009-1162-0
7. Florence CS, Bergen G, Atherly A, Burns E, Stevens J, Drake C. Medical Costs of Fatal and Nonfatal Falls in Older Adults. J Am Geriatr Soc. 2018;66: 693–698. doi:10.1111/jgs.15304
8. Heinrich S, Rapp K, Rissmann U, Becker C, König H-H. Cost of falls in old age: a systematic review. Osteoporosis International. 2010;21: 891–902. doi:10.1007/s00198-009-1100-1
9. Li Y, Hou L, Zhao H, Xie R, Yi Y, Ding X. Risk factors for falls among community-dwelling older adults: A systematic review and meta-analysis. Front Med (Lausanne). 2023;9: 1–13. doi:10.3389/fmed.2022.1019094
10. Shao L, Shi Y, Xie X-Y, Wang Z, Wang Z-A, Zhang J-E. Incidence and Risk Factors of Falls Among Older People in Nursing Homes: Systematic Review and Meta-Analysis. J Am Med Dir Assoc. 2023;24: 1708–1717. doi:10.1016/j.jamda.2023.06.002
11. Gillespie LD, Robertson MC, Gillespie WJ, Sherrington C, Gates S, Clemson LM, et al. Interventions for preventing falls in older people living in the community. Cochrane Database of Systematic Reviews. 2012;2012: 1–366. doi:10.1002/14651858.CD007146.pub3
12. Olij BF, Ophuis RH, Polinder S, van Beeck EF, Burdorf A, Panneman MJM, et al. Economic Evaluations of Falls Prevention Programs for Older Adults: A Systematic Review. J Am Geriatr Soc. 2018;66: 2197–2204. doi:10.1111/jgs.15578
13. Dautzenberg L, Beglinger S, Tsokani S, Zevgiti S, Raijmann RCMA, Rodondi N, et al. Interventions for preventing falls and fall-related fractures in community-dwelling older adults: A systematic review and network meta-analysis. J Am Geriatr Soc. 2021;69: 2973–2984. doi:10.1111/jgs.17375
14. Meekes WM, Korevaar JC, Leemrijse CJ, van de Goor IA. Practical and validated tool to assess falls risk in the primary care setting: a systematic review. BMJ Open. 2021;11: 1–10. doi:10.1136/bmjopen-2020-045431
15. Stevens JA, Ballesteros MF, Mack KA, Rudd RA, DeCaro E, Adler G. Gender Differences in Seeking Care for Falls in the Aged Medicare Population. Am J Prev Med. 2012;43: 59–62. doi:10.1016/j.amepre.2012.03.008
16. Montero-Odasso M, van der Velde N, Martin FC, Petrovic M, Tan MP, Ryg J, et al. World guidelines for falls prevention and management for older adults: a global initiative. Age Ageing. 2022;51: 1–36. doi:10.1093/ageing/afac205
17. Markle-Reid MF, Dykeman CS, Reimer HD, Boratto LJ, Goodall CE, McGugan JL. Engaging community organizations in falls prevention for older adults: Moving from research to action. Can J Public Health. 2015;106: e189–e196. doi:10.17269/cjph.106.4776
18. Olij BF, Erasmus V, Kuiper JI, van Zoest F, van Beeck EF, Polinder S. Falls prevention activities among community-dwelling elderly in the Netherlands: A Delphi study. Injury. 2017;48: 2017–2021. doi:10.1016/j.injury.2017.06.022
19. Leysens G, Vlaeyen E, Vanaken D, Janssens E, Dejaeger E, Cambier D, et al. Het toepassen van valpreventiemaatregelen bij thuiswonende ouderen: een survey onderzoek in Vlaanderen [The use of fall prevention strategies in home care: a survey

in Flanders]. Tijdschr Gerontol Geriatr. 2017;48: 121–133. doi:10.1007/s12439-017-0215-7

20. Moreland B, Kakara R, Henry A. Trends in Nonfatal Falls and Fall-Related Injuries Among Adults Aged ≥65 Years — United States, 2012–2018. MMWR Morb Mortal Wkly Rep. 2020;69: 875–881. doi:10.15585/mmwr.mm6927a5
21. Shaver AL, Clark CM, Hejna M, Feuerstein S, Wahler RG, Jacobs DM. Trends in fall-related mortality and fall risk increasing drugs among older individuals in the United States,1999–2017. Pharmacoepidemiol Drug Saf. 2021;30: 1049–1056. doi:10.1002/pds.5201
22. Fretheim A, Flottorp S, Oxman A. Effekt av tiltak for implementering av kliniske retningslinjer. Rapport fra Kunnskapssenteret nr. 10-2015. Oversikt over systematiske oversikter [Effect of interventions for implementing clinical practice guidelines]. Oslo; 2015.
23. Van de Velde S, Heselmans A, Delvaux N, Brandt L, Marco-Ruiz L, Spitaels D, et al. A systematic review of trials evaluating success factors of interventions with computerised clinical decision support. Implementation Science. 2018;13: 1–11. doi:10.1186/s13012-018-0790-1
24. Wasylewicz ATM, Scheepers-Hoeks AMJW. Clinical Decision Support Systems. Fundamentals of Clinical Data Science. Cham: Springer International Publishing; 2019. pp. 153–169. doi:10.1007/978-3-319-99713-1_11
25. Blalock SJ, Ferreri SP, Renfro CP, Robinson JM, Farley JF, Ray N, et al. Impact of STEADI-Rx : A Community Pharmacy-Based Fall Prevention Intervention. J Am Geriatr Soc. 2020;68: 1778–1786. doi:10.1111/jgs.16459
26. Dykes PC, Carroll DL, Hurley A, Lipsitz S, Benoit A, Chang F, et al. Fall prevention in acute care hospitals: a randomized trial. JAMA. 2010;304: 1912–1918. doi:10.1001/jama.2010.1567
27. Carroll DL, Dykes PC, Hurley AC. An Electronic Fall Prevention Toolkit: Effect on Documentation Quality. Nurs Res. 2012;61: 309–313. doi:10.1097/NNR.0b013e31825569de
28. Dykes PC, Burns Z, Adelman J, Benneyan J, Bogaisky M, Carter E, et al. Evaluation of a Patient-Centered Fall-Prevention Tool Kit to Reduce Falls and Injuries. JAMA Netw Open. 2020;3: e2025889. doi:10.1001/jamanetworkopen.2020.25889
29. Aizen E, Lutsyk G, Wainer L, Carmeli S. Effectiveness of individualized fall prevention program in geriatric rehabilitation hospital setting: a cluster randomized trial. Aging Clin Exp Res. 2015;27: 681–688. doi:10.1007/s40520-015-0330-7
30. Barker AL, Morello RT, Wolfe R, Brand CA, Haines TP, Hill KD, et al. 6-PACK programme to decrease fall injuries in acute hospitals: cluster randomised controlled trial. BMJ. 2016; h6781. doi:10.1136/bmj.h6781
31. Mebrahtu TF, Skyrme S, Randell R, Keenan A-M, Bloor K, Yang H, et al. Effects of computerised clinical decision support systems (CDSS) on nursing and allied health professional performance and patient outcomes: a systematic review of experimental and observational studies. BMJ Open. 2021;11: e053886. doi:10.1136/bmjopen-2021-053886
32. Damoiseaux-Volman BA, van der Velde N, Ruige SG, Romijn JA, Abu-Hanna A, Medlock S. Effect of Interventions With a Clinical Decision Support System for Hospitalized Older Patients: Systematic Review Mapping Implementation and Design Factors. JMIR Med Inform. 2021;9: 1–11. doi:10.2196/28023
33. Yourman L, Concato J, Agostini J V. Use of computer decision support interventions to improve medication prescribing in older adults: A systematic review. Am J Geriatr Pharmacother. 2008;6: 119–129. doi:10.1016/j.amjopharm.2008.06.001

34. Kwan JL, Lo L, Ferguson J, Goldberg H, Diaz-Martinez JP, Tomlinson G, et al. Computerised clinical decision support systems and absolute improvements in care: meta-analysis of controlled clinical trials. BMJ. 2020;370: 1–11. doi:10.1136/bmj.m3216
35. Moja L, Kwag KH, Lytras T, Bertizzolo L, Brandt L, Pecoraro V, et al. Effectiveness of Computerized Decision Support Systems Linked to Electronic Health Records: A Systematic Review and Meta-Analysis. Am J Public Health. 2014;104: e12–e22. doi:10.2105/AJPH.2014.302164
36. Garg AX, Adhikari NKJ, McDonald H, Rosas-Arellano MP, Devereaux PJ, Beyene J, et al. Effects of Computerized Clinical Decision Support Systems on Practitioner Performance and Patient Outcomes. JAMA. 2005;293: 1223–1238. doi:10.1001/jama.293.10.1223
37. Jaspers MWM, Smeulers M, Vermeulen H, Peute LW. Effects of clinical decision-support systems on practitioner performance and patient outcomes: a synthesis of high-quality systematic review findings. Journal of the American Medical Informatics Association. 2011;18: 327–334. doi:10.1136/amiajnl-2011-000094
38. Taheri Moghadam S, Sadoughi F, Velayati F, Ehsanzadeh SJ, Poursharif S. The effects of clinical decision support system for prescribing medication on patient outcomes and physician practice performance: a systematic review and meta-analysis. BMC Med Inform Decis Mak. 2021;21: 1–26. doi:10.1186/s12911-020-01376-8
39. Shahmoradi L, Safdari R, Ahmadi H, Zahmatkeshan M. Clinical decision support systems-based interventions to improve medication outcomes: A systematic literature review on features and effects. Med J Islam Repub Iran. 2021;35: 1–16. doi:10.47176/mjiri.35.27
40. Elley CR, Robertson MC, Garrett S, Kerse NM, McKinlay E, Lawton B, et al. Effectiveness of a Falls-and-Fracture Nurse Coordinator to Reduce Falls: A Randomized, Controlled Trial of At-Risk Older Adults. J Am Geriatr Soc. 2008;56: 1383–1389. doi:10.1111/j.1532-5415.2008.01802.x
41. Tamblyn R, Eguale T, Buckeridge DL, Huang A, Hanley J, Reidel K, et al. The effectiveness of a new generation of computerized drug alerts in reducing the risk of injury from drug side effects: a cluster randomized trial. Journal of the American Medical Informatics Association. 2012;19: 635–643. doi:10.1136/amiajnl-2011-000609
42. Shamseer L, Moher D, Clarke M, Ghersi D, Liberati A, Petticrew M, et al. Preferred reporting items for systematic review and meta-analysis protocols (PRISMA-P) 2015: elaboration and explanation. BMJ. 2015;349: g7647. doi:10.1136/bmj.g7647
43. Higgins J, Thomas J, Chandler J, Cumpston M, Li T, Page M, et al. Cochrane Handbook for Systematic Reviews of Interventions version 6.4 (updated August 2023). 6.4. Cochrane; 2023. Available: www.training.cochrane.org/handbook
44. EPOC resources for review authors. [cited 22 Nov 2020]. Available: https://epoc.cochrane.org/resources/epoc-resources-review-authors
45. Page MJ, McKenzie JE, Bossuyt PM, Boutron I, Hoffmann TC, Mulrow CD, et al. The PRISMA 2020 statement: an updated guideline for reporting systematic reviews. BMJ. 2021;372: n71. doi:10.1136/bmj.n71
46. Covidence Systematic Review Tool. In: Covidence [Internet]. [cited 17 May 2025]. Available: https://www.covidence.org/
47. Griese-Mammen N, Hersberger KE, Messerli M, Leikola S, Horvat N, van Mil JWF, et al. PCNE definition of medication review: reaching agreement. Int J Clin Pharm. 2018;40: 1199–1208. doi:10.1007/s11096-018-0696-7

48. El-Khoury F, Cassou B, Charles M-A, Dargent-Molina P. The effect of fall prevention exercise programmes on fall induced injuries in community dwelling older adults: systematic review and meta-analysis of randomised controlled trials. BMJ. 2013;347: f6234. doi:10.1136/bmj.f6234
49. Seppala LJ, van de Glind EMM, Daams JG, Ploegmakers KJ, de Vries M, Wermelink AMAT, et al. Fall-Risk-Increasing Drugs: A Systematic Review and Meta-analysis: III. Others. J Am Med Dir Assoc. 2018;19: 372.e1-372.e8. doi:10.1016/j.jamda.2017.12.099
50. Hoffmann TC, Glasziou PP, Boutron I, Milne R, Perera R, Moher D, et al. Better reporting of interventions: template for intervention description and replication (TIDieR) checklist and guide. BMJ. 2014;348: g1687–g1687. doi:10.1136/bmj.g1687
51. Lewin S, Hendry M, Chandler J, Oxman AD, Michie S, Shepperd S, et al. Assessing the complexity of interventions within systematic reviews: development, content and use of a new tool (iCAT_SR). BMC Med Res Methodol. 2017;17: 76. doi:10.1186/s12874-017-0349-x
52. Lamb SE, Becker C, Gillespie LD, Smith JL, Finnegan S, Potter R, et al. Reporting of complex interventions in clinical trials: development of a taxonomy to classify and describe fall-prevention interventions. Trials. 2011;12: 125. doi:10.1186/1745-6215-12-125
53. Van de Velde S, Kunnamo I, Roshanov P, Kortteisto T, Aertgeerts B, Vandvik PO, et al. The GUIDES checklist: development of a tool to improve the successful use of guideline-based computerised clinical decision support. Implementation Science. 2018;13: 86. doi:10.1186/s13012-018-0772-3
54. Medlock S, Wyatt JC, Patel VL, Shortliffe EH, Abu-Hanna A. Modeling information flows in clinical decision support: key insights for enhancing system effectiveness. Journal of the American Medical Informatics Association. 2016;23: 1001–1006. doi:10.1093/jamia/ocv177
55. Sterne JAC, Savović J, Page MJ, Elbers RG, Blencowe NS, Boutron I, et al. RoB 2: a revised tool for assessing risk of bias in randomised trials. BMJ. 2019;366: l4898. doi:10.1136/bmj.l4898
56. Sterne JA, Hernán MA, Reeves BC, Savović J, Berkman ND, Viswanathan M, et al. ROBINS-I: a tool for assessing risk of bias in non-randomised studies of interventions. BMJ. 2016;355: i4919. doi:10.1136/bmj.i4919
57. Sterne J, Hernán M, McAleenan A, Reeves B, Higgins J. Chapter 25: Assessing risk of bias in a non-randomized study. In: Higgins JPT, Thomas J, Chandler J, Cumpston M, Li T, Page MJ, Welch VA (editors). Cochrane Handbook for Systematic Reviews of Interventions version 6.4 (updated August 2023). Cochrane; 2023. Available: http://www.training.cochrane.org/handbook
58. Campbell M, McKenzie JE, Sowden A, Katikireddi SV, Brennan SE, Ellis S, et al. Synthesis without meta-analysis (SWiM) in systematic reviews: reporting guideline. BMJ. 2020;368: l6890. doi:10.1136/bmj.l6890
59. McKenzie J, Brennan S. Chapter 12: Synthesizing and presenting findings using other methods. In: Higgins JPT, Thomas J, Chandler J, Cumpston M, Li T, Page MJ, Welch VA (editors). Cochrane Handbook for Systematic Reviews of Interventions version 6.4 (updated August 2023). Cochrane; 2023. Available: http://www.training.cochrane.org/handbook
60. Tricco AC, Thomas SM, Veroniki AA, Hamid JS, Cogo E, Strifler L, et al. Comparisons of Interventions for Preventing Falls in Older Adults: A Systematic Review and Meta-analysis. JAMA. 2017;318: 1687–1699. doi:10.1001/jama.2017.15006

61. Deeks JJ, Higgins JP, Altman DG. Chapter 10: Analysing data and undertaking meta-analyses. In: Higgins JPT, Thomas J, Chandler J, Cumpston M, Li T, Page MJ, Welch VA (editors). Cochrane Handbook for Systematic Reviews of Interventions version 6.4 (updated August 2023). Cochrane; 2023. Available: www.training.cochrane.org/handbook

62. StataCorp. Stata Statistical Software: Release 18. College Station, TX. 2023.

63. Higgins J, Li T, Deeks J. Chapter 6: Choosing effect measures and computing estimates of effect. In: Higgins JPT, Thomas J, Chandler J, Cumpston M, Li T, Page MJ, Welch VA (editors). Cochrane Handbook for Systematic Reviews of Interventions version 6.4 (updated August 2023). Cochrane; 2023. Available: http://www.training.cochrane.org/handbook

64. Bergen G, Stevens MR, Burns ER. Falls and Fall Injuries Among Adults Aged ≥65 Years — United States, 2014. MMWR Morb Mortal Wkly Rep. 2016;65: 993–998. doi:10.15585/mmwr.mm6537a2

65. Guyatt G, Zeng L, Brignardello-Petersen R, Prasad M, De Beer H, Murad MH, et al. Core GRADE 2: choosing the target of certainty rating and assessing imprecision. BMJ. 2025;389: e081904. doi:10.1136/bmj-2024-081904

66. Egger M, Smith GD, Schneider M, Minder C. Bias in meta-analysis detected by a simple, graphical test. BMJ. 1997;315: 629–634. doi:10.1136/bmj.315.7109.629

67. Hultcrantz M, Rind D, Akl EA, Treweek S, Mustafa RA, Iorio A, et al. The GRADE Working Group clarifies the construct of certainty of evidence. J Clin Epidemiol. 2017;87: 4–13. doi:10.1016/j.jclinepi.2017.05.006

68. Zeng L, Brignardello-Petersen R, Hultcrantz M, Mustafa RA, Murad MH, Iorio A, et al. GRADE Guidance 34: update on rating imprecision using a minimally contextualized approach. J Clin Epidemiol. 2022;150: 216–224. doi:10.1016/j.jclinepi.2022.07.014

69. Santesso N, Glenton C, Dahm P, Garner P, Akl EA, Alper B, et al. GRADE guidelines 26: informative statements to communicate the findings of systematic reviews of interventions. J Clin Epidemiol. 2020;119: 126–135. doi:10.1016/j.jclinepi.2019.10.014

70. Guyatt G, Yao L, Murad MH, Hultcrantz M, Agoritsas T, De Beer H, et al. Core GRADE 6: presenting the evidence in summary of findings tables. BMJ. 2025;389: e083866. doi:10.1136/bmj-2024-083866

71. Clemson L, Mackenzie L, Lovarini M, Roberts C, Poulos R, Sherrington C, et al. Integrated solutions for sustainable fall prevention in primary care: a pragmatic hybrid-type 2 mixed methods implementation and effectiveness study. Front Public Health. 2024;12. doi:10.3389/fpubh.2024.1446525

72. Phelan EA, Williamson BD, Balderson BH, Cook AJ, Piccorelli A V., Fujii MM, et al. Reducing Central Nervous System–Active Medications to Prevent Falls and Injuries Among Older Adults. JAMA Netw Open. 2024;7: e2424234. doi:10.1001/jamanetworkopen.2024.24234

73. Bhasin S, Gill TM, Reuben DB, Latham NK, Ganz DA, Greene EJ, et al. A Randomized Trial of a Multifactorial Strategy to Prevent Serious Fall Injuries. New England Journal of Medicine. 2020;383: 129–140. doi:10.1056/NEJMoa2002183

74. Blum MR, Sallevelt BTGM, Spinewine A, O'Mahony D, Moutzouri E, Feller M, et al. Optimizing Therapy to Prevent Avoidable Hospital Admissions in Multimorbid Older Adults (OPERAM): cluster randomised controlled trial. BMJ. 2021;374: n1585. doi:10.1136/bmj.n1585

75. Byrne CM. Impact of prospective computerized clinical decision support information and targeted assistance on nursing home resident outcomes. University of Albany,

State University of New York. 2005. Available: https://www.proquest.com/openview/59a276fe7e8913563fcd2bb9b487d19d/1?pq-origsite=gscholar&cbl=18750&diss=y

76. Ferrer A, Formiga F, Sanz H, de Vries OJ, Pujol R, Badia T, et al. Multifactorial assessment and targeted intervention to reduce falls among the oldest-old: a randomized controlled trial. Clin Interv Aging. 2014;9: 383–393. doi:10.2147/CIA.S57580

77. Frankenthal D, Lerman Y, Kalendaryev E, Lerman Y. Intervention with the Screening Tool of Older Persons Potentially Inappropriate Prescriptions/Screening Tool to Alert Doctors to Right Treatment Criteria in Elderly Residents of a Chronic Geriatric Facility: A Randomized Clinical Trial. J Am Geriatr Soc. 2014;62: 1658–1665. doi:10.1111/jgs.12993

78. Gallagher PF, O’Connor MN, O’Mahony D. Prevention of Potentially Inappropriate Prescribing for Elderly Patients: A Randomized Controlled Trial Using STOPP/START Criteria. Clin Pharmacol Ther. 2011;89: 845–854. doi:10.1038/clpt.2011.44

79. Ganz DA, Kim S-B, Zingmond DS, Ramirez KD, Roth CP, Jennings LA, et al. Effect of a Falls Quality Improvement Program on Serious Fall-Related Injuries. J Am Geriatr Soc. 2015;63: 63–70. doi:10.1111/jgs.13154

80. Wenger NS, Roth CP, Hall WJ, Ganz DA, Snow V, Byrkit J, et al. Practice Redesign to Improve Care for Falls and Urinary Incontinence. Arch Intern Med. 2010;170: 1765–1772. doi:10.1001/archinternmed.2010.387

81. Ganz DA, Yuan AH, Greene EJ, Latham NK, Araujo K, Siu AL, et al. Effect of the STRIDE fall injury prevention intervention on falls, fall injuries, and health-related quality of life. J Am Geriatr Soc. 2022;70: 3221–3229. doi:10.1111/jgs.17964

82. Groshaus H, Boscan A, Khandwala F, Holroyd-Leduc J. Use of Clinical Decision Support to Improve the Quality of Care Provided to Older Hospitalized Patients. Appl Clin Inform. 2012;03: 94–102. doi:10.4338/ACI-2011-08-RA-0047

83. Healey F, Monro A, Cockram A, Adams V, Heseltine D. Using targeted risk factor reduction to prevent falls in older in-patients: a randomised controlled trial. Age Ageing. 2004;33: 390–395. doi:10.1093/ageing/afh130

84. Lightbody E. Evaluation of a nurse-led falls prevention programme versus usual care: a randomized controlled trial. Age Ageing. 2002;31: 203–210. doi:10.1093/ageing/31.3.203

85. Mahoney JE, Shea TA, Przybelski R, Jaros L, Gangnon R, Cech S, et al. Kenosha County Falls Prevention Study: A Randomized, Controlled Trial of an Intermediate-Intensity, Community-Based Multifactorial Falls Intervention. J Am Geriatr Soc. 2007;55: 489–498. doi:10.1111/j.1532-5415.2007.01144.x

86. Logan PA, Horne JC, Gladman JRF, Gordon AL, Sach T, Clark A, et al. Multifactorial falls prevention programme compared with usual care in UK care homes for older people: multicentre cluster randomised controlled trial with economic evaluation. BMJ. 2021;375: e066991. doi:10.1136/bmj-2021-066991

87. Peterson JF, Rosenbaum BP, Waitman LR, Habermann R, Powers J, Harrell D, et al. Physicians’ response to guided geriatric dosing: initial results from a randomized trial. Stud Health Technol Inform. 2007;129: 1037–1040. Available: http://www.ncbi.nlm.nih.gov/pubmed/17911873

88. Snooks HA, Carter B, Dale J, Foster T, Humphreys I, Logan PA, et al. Support and Assessment for Fall Emergency Referrals (SAFER 1): Cluster Randomised Trial of Computerised Clinical Decision Support for Paramedics. Quinn TJ, editor. PLoS One. 2014;9: e106436. doi:10.1371/journal.pone.0106436

89. Weber V, White A, McIlvried R. An Electronic Medical Record (EMR)-Based Intervention to Reduce Polypharmacy and Falls in an Ambulatory Rural Elderly Population. J Gen Intern Med. 2008;23: 399–404. doi:10.1007/s11606-007-0482-z
90. Wenger NS, Roth CP, Shekelle PG, Young RT, Solomon DH, Kamberg CJ, et al. A Practice-Based Intervention to Improve Primary Care for Falls, Urinary Incontinence, and Dementia. J Am Geriatr Soc. 2009;57: 547–555. doi:10.1111/j.1532-5415.2008.02128.x
91. Sutton RT, Pincock D, Baumgart DC, Sadowski DC, Fedorak RN, Kroeker KI. An overview of clinical decision support systems: benefits, risks, and strategies for success. NPJ Digit Med. 2020;3: 17. doi:10.1038/s41746-020-0221-y
92. Hopewell S, Adedire O, Copsey BJ, Boniface GJ, Sherrington C, Clemson L, et al. Multifactorial and multiple component interventions for preventing falls in older people living in the community. Cochrane Database of Systematic Reviews. 2018;7: CD012221. doi:10.1002/14651858.CD012221.pub2
93. Wang H, Boissel J-P, Nony P. Revisiting the relationship between baseline risk and risk under treatment. Emerg Themes Epidemiol. 2009;6: 1. doi:10.1186/1742-7622-6-1
94. Ho KM. Importance of the baseline risk in determining sample size and power of a randomized controlled trial. Journal of Emergency and Critical Care Medicine. 2018;2. doi:10.21037/jeccm.2018.08.06
95. Goodwin V, Jones-Hughes T, Thompson-Coon J, Boddy K, Stein K. Implementing the evidence for preventing falls among community-dwelling older people: A systematic review. J Safety Res. 2011;42: 443–451. doi:10.1016/j.jsr.2011.07.008
96. Finnegan S, Seers K, Bruce J. Long-term follow-up of exercise interventions aimed at preventing falls in older people living in the community: a systematic review and meta-analysis. Physiotherapy. 2019;105: 187–199. doi:10.1016/j.physio.2018.09.002
97. Wang T, Tan J-Y (Benjamin), Liu X-L, Zhao I. Barriers and enablers to implementing clinical practice guidelines in primary care: an overview of systematic reviews. BMJ Open. 2023;13: e062158. doi:10.1136/bmjopen-2022-062158
98. Leonard E, de Kock I, Bam W. Barriers and facilitators to implementing evidence-based health innovations in low- and middle-income countries: A systematic literature review. Eval Program Plann. 2020;82: 101832. doi:10.1016/j.evalprogplan.2020.101832
99. Schünemann H, Brożek J, Guyatt G, Oxman A. GRADE Handbook: Introduction to GRADE Handbook. 2013. Available: https://gdt.gradepro.org/app/handbook/handbook.html

Supporting information

S1 Table. PRISMA checklist.docx

S2 Table. Differences between protocol and review.docx

S3 Table. Electronic searches.docx

S4 Table. Excluded studies.xlsx

S5Table. Description of interventions and funding sources.docx

S6Table. Design factors of CDS.docx

S7 Table. Risk of bias.pdf

S8 Table. Individual study results.docx

S9 Table. GRADE evidence profile.docx

S1 Appendix. Data used for analyses of healthcare practitioner performance outcomes.xlsx

S2 Appendix. Data used for meta-analyses.xlsx

S3 Appendix. Extracted data.csv

S1 Fig. Funnell plot of comparison: CDS interventions vs control on fall risk.

S2 Fig. Trim-and-fill analysis of comparison: CDS interventions vs control on fall risk.

S3 Fig. Meta-analysis comparing CDS interventions with control on fall rate, subgroup. analysis by patients' age.

S4 Fig. Funnel plot of comparison: CDS interventions vs control on fall rate.

S5 Fig. Trim-and-fill analysis of comparison: CDS interventions vs control on fall rate.

S6 Fig. Meta-analysis comparing CDS interventions with control on fall rate, subgroup analysis by risk of bias.

S7 Fig. Meta-analysis comparing CDS interventions with control on fall injury rate, subgroup analysis by risk of bias.

S8 Fig. Meta-analysis comparing CDS interventions with control on fall injury rate, subgroup analysis by study setting.

Fig 1

Click here to access/download;Figure;Fig1.tif

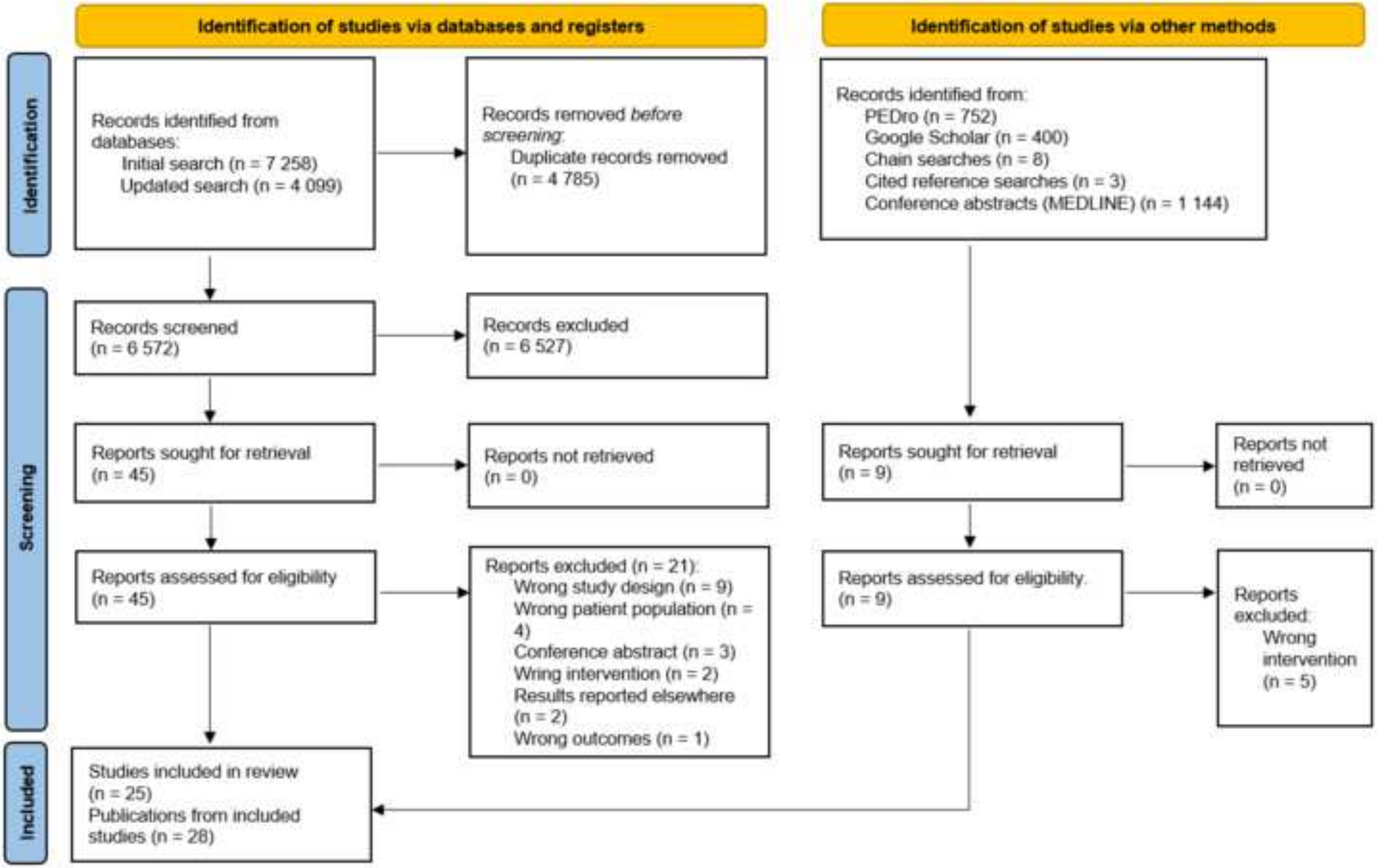

Fig2 Click here to access/download;Figure;Fig2.tif

Study	Direction of effect: Favours CDS	Direction of effect: Favours control	Results	Specific outcome	RoB
**Adherence to recommended practice**					
Barker (2016)	X		Rate ratio 3.05 (95% CI 2.14, 4.34)	Use of all 6-PACK programme components (fall risk tool and six interventions) during eight months FU	!
Carroll (2012)	X		89% in IG, 64% in CG (p < 0.0001)	Proportion of patient records reviewed that had fall risk documented on the plan of care during six months FU	-
Clemson (2024)	X		0.90 units (95% CI 0.33, 1.46)	Changes in GPs' engagement in fall prevention activities at 12 months FU, including risk assessments, medication reviews, and providing advice, compared to the control group. Composite score of how often they engaged with older patients in managing falls, max 9	-
Dykes (2010)	X		94% in IG, 81% in CG	Adherence to intervention protocol during six months FU (Morse Falls Scale completion)	-
Groshaus (2012)	X		Mean difference 3.1 (95% CI 1.9, 5.3)	Mean rate of use of the order set at the end of each two-week period for a total of three months	-*
Wenger (2009)	X		44% in IG, 23% in CG (p < 0.001)	Proportion of recommended care (quality indicators) for falls provided to patients during 13 months FU	-*
Wenger (2010)	X		60% in IG, 37.6% in CG (p < 0.001)	Proportion of recommended care (quality indicators) for falls provided to patients during 12 months FU	-*
Snooks (2014)	X		Odds ratio 2.04 (95% CI 1.12, 3.72) (p = 0.021)	Odds of a patient being referred to a falls service during one month FU in intervention group compared with control group	!
	Sign test p < 0.01				
**Medication outcomes**					
Blalock (2020)	X		Mean difference of – 0.12 in intervention group compared with – 0.08 in control group (p = 0.05)	Change in use of fall-risk-increasing drugs in individuals who screened positive for fall risk (DBI score) from 12-month pre-intervention period to 12-month post-intervention period*	!
Blum (2021)	X		Odds ratio 0.99 (95% CI 0.82, 1.20)	Presence of drug overuse based on STOPP criteria during two months FU in intervention group compared with control group	!
Frankenthal (2014)	X		Mean (SD) 7.3 (2.7) in IG, 8.9 (3.2) in CG (p < 0.001)	Number of medications prescribed at 12-month FU	!
Gallagher (2011)	X		Absolute risk reduction 35.7% (95% CI 26.3%, 44.9%)	Unnecessary polypharmacy, the use of drugs at incorrect doses, and potential drug-drug-diseases and drug-disease interactions at discharge	-
Lightbody (2002)	X		608 in IG, 684 in CG (p = 0.41)	Number of daily medications at six-month FU	-
Peterson (2007)	X		Median (IQR) 2.5 (1.0, 4.0) in IG, 3.0 (1.5, 5.0) in CG (p < 0.001)	Ratio between prescribed and recommended medication dose during nine months FU	-
Phelan (2024)	X		Adjusted relative risk 1.24 (95% CI 0.90, 1.70)	Discontinuation of medications (defined as no prescription fill for 90 days), summarized across all target medication classes, referred to as "first target medication", at six months FU	-
Tamblyn (2012)	X		Mean reduction 1.7 (95% CI 0.2, 3.2)	Reduction in risk of injury (based on psychotropic medications and non-modifiable risk factors) at 23-month FU	+
Weber (2008)	X		Mean reduction 0.496 (p = 0.088)	Change in number of active medications during 12 months FU	-
	Sign test p < 0.01				

Fig3
Click here to access/download;Figure;Fig3.tif

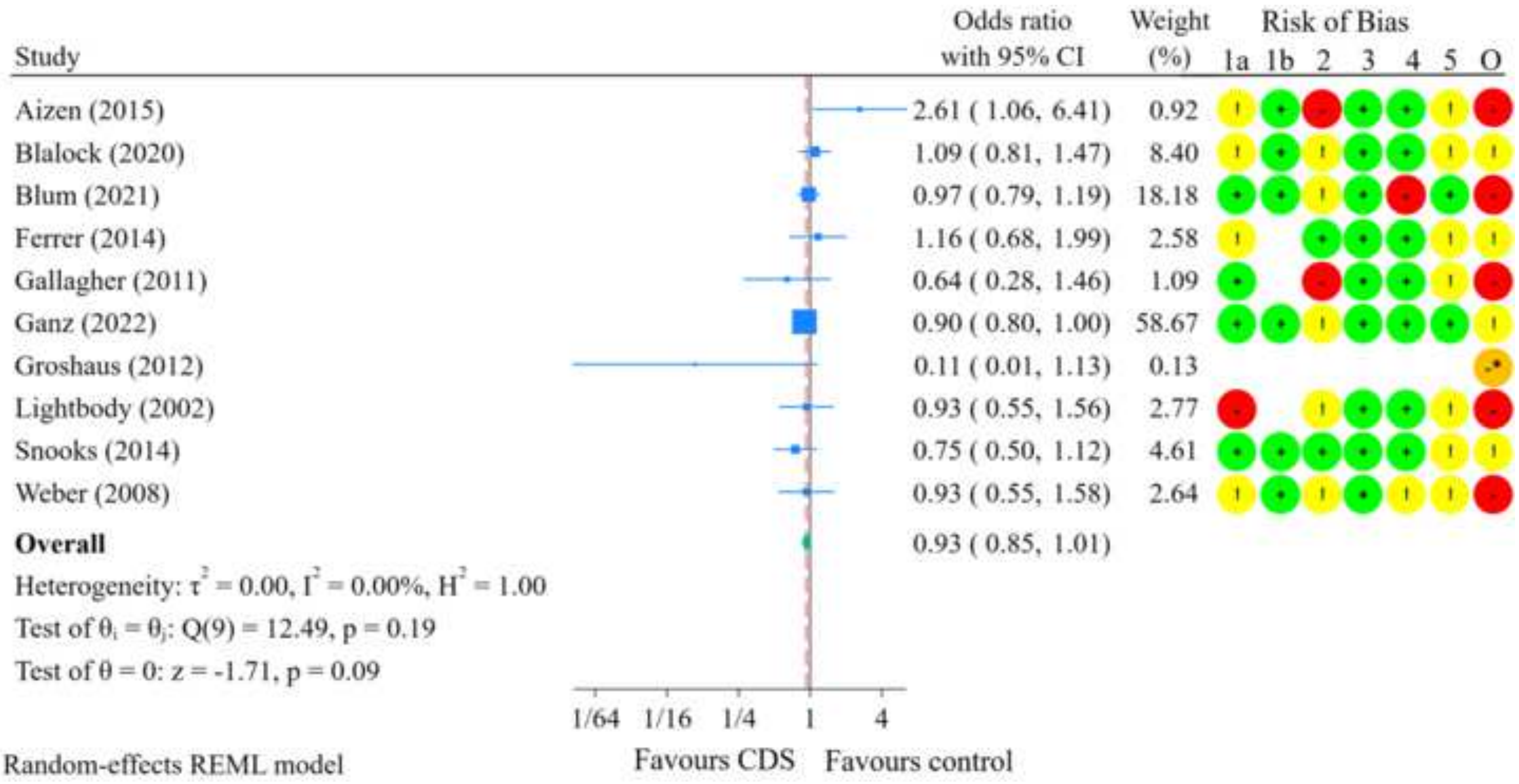

Fig4

Click here to access/download;Figure;Fig4.tif

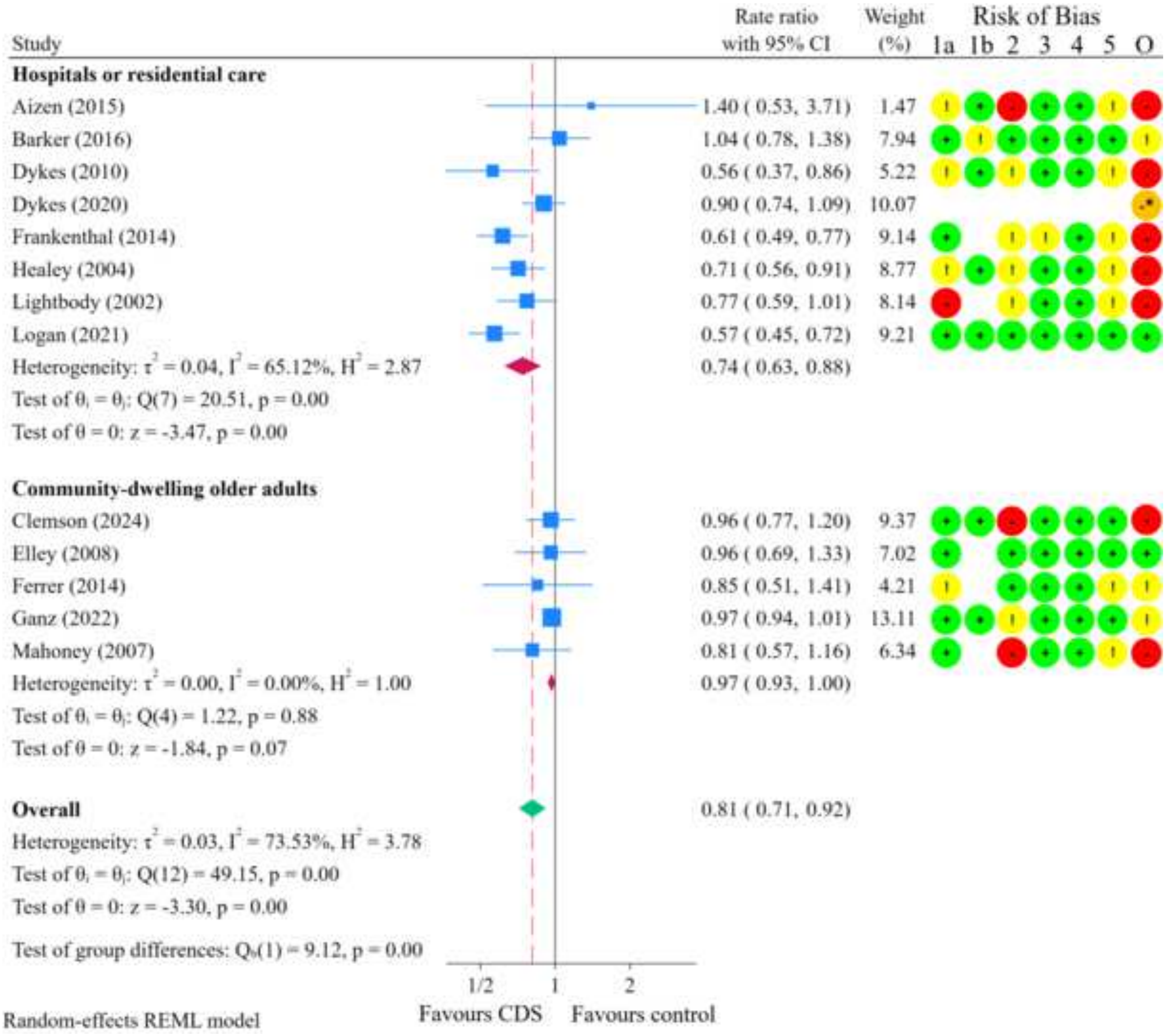

Fig5

Click here to access/download;Figure;Fig5.tif

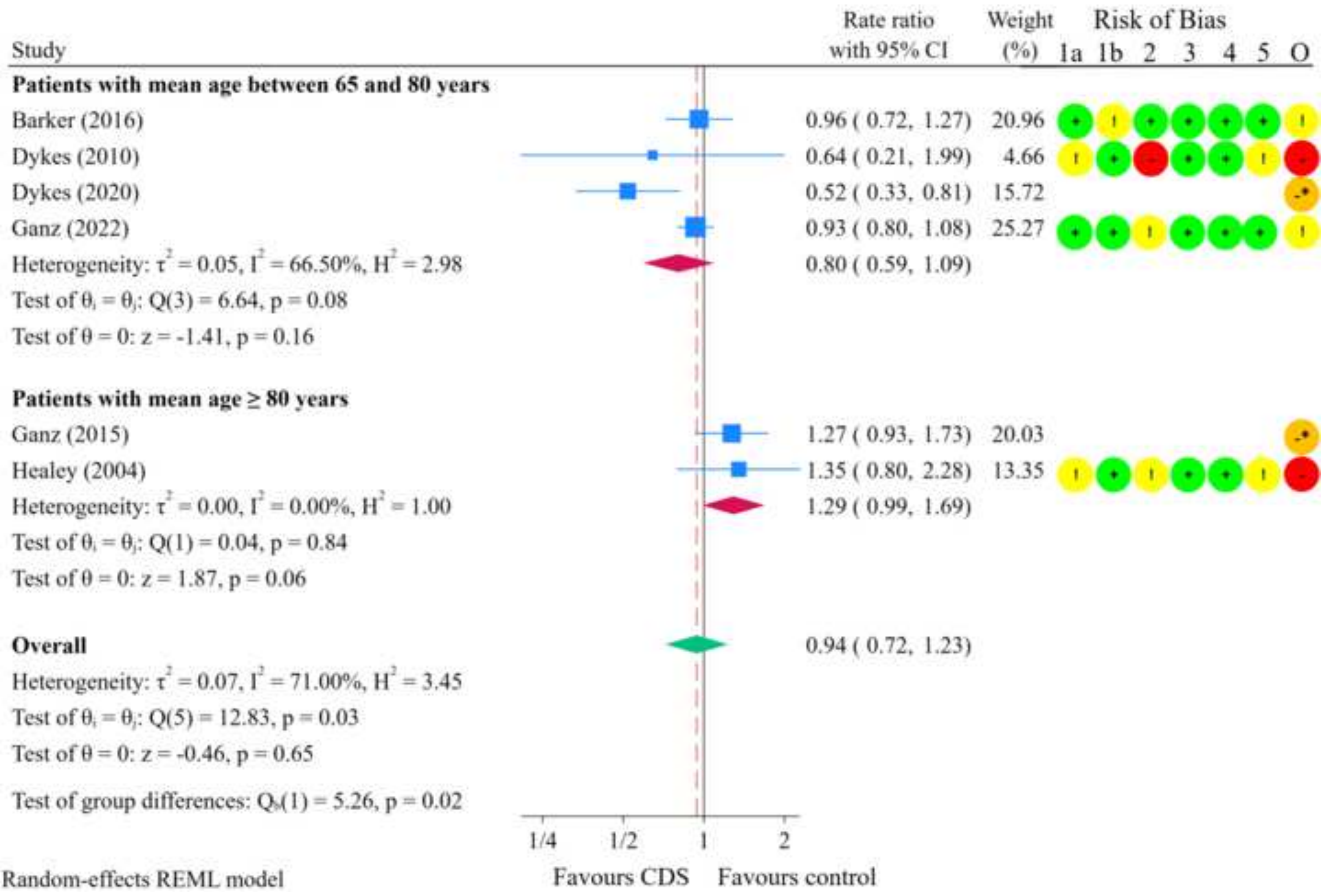

S1 Table

Click here to access/download
Supporting Information
S1_Table.docx

S2 Table

Click here to access/download
Supporting Information
S2_Table.docx

S3 Table

Click here to access/download
Supporting Information
S3_Table.docx

S4 Table

Click here to access/download
Supporting Information
S4_Table.xlsx

S5 Table

Click here to access/download
Supporting Information
S5_Table.docx

S6 Table

Click here to access/download
Supporting Information
S6_Table.docx

S7 Table

Click here to access/download
Supporting Information
S7_Table.pdf

S8 Table

Click here to access/download
Supporting Information
S8_Table.docx

S1 Appendix

Click here to access/download
Supporting Information
S1_Appendix.xlsx

S2 Appendix

Click here to access/download
Supporting Information
S2_Appendix.xlsx

S3 Appendix

Click here to access/download
Supporting Information
S3_Appendix.csv

S1 Fig

Click here to access/download
Supporting Information
S1_Fig.png

S2 Fig

Click here to access/download
Supporting Information
S2_Fig.png

S4 Fig

Click here to access/download
Supporting Information
S4_Fig.png

S5 Fig

Click here to access/download
Supporting Information
S5_Fig.png

S6 Fig

Click here to access/download
Supporting Information
S6_Fig.png

S9 Table

Click here to access/download
Supporting Information
S9_Table.docx

S3 Fig

Click here to access/download
Supporting Information
S3_Fig.png

S7 Fig

Click here to access/download
Supporting Information
S7_Fig.png

S8 Fig

Click here to access/download
Supporting Information
S8_Fig.png

Effectiveness of clinical decision support in fall prevention among older adults: a systematic review and meta-analysis

Rune Solli[1*], Nina Rydland Olsen[2], Linda Aimée Hartford Kvæl[1,3], Stijn Van de Velde[4], Are Hugo Pripp[1], Signe Agnes Flottorp[5], Therese Brovold[1]

[1] Department of Rehabilitation Science and Health Technology, Faculty of Health Sciences, OsloMet – Oslo Metropolitan University, Oslo, Norway

[2] Department of Health and Functioning, Western Norway University of Applied Sciences, Faculty of health and social sciences, Bergen, Norway

[3] Norwegian Social Research (NOVA), OsloMet – Oslo Metropolitan University, Oslo, Norway

[4] MAGIC Evidence Ecosystem Foundation; Research Department, Lovisenberg Diaconal Hospital, Oslo, Norway

[5] Division of Health Services, Norwegian Institute of Public Health, Oslo, Norway

*Corresponding author

E-mail: runesoll@oslomet.no (RS)

Abstract

Background: Systematic use of Clinical Decision Support (CDS), which provides timely information to assist healthcare practitioners in decision-making, is recommended in the implementation of fall prevention among older adults. This systematic review aimed to evaluate~~investigate~~ the effects of CDS for~~ in the implementation of~~ fall prevention on healthcare practitioners' adherence to recommended practice, medication outcomes, ~~performance~~ and patient outcomes.

Methods: We searched Medline, EMBASE, CINAHL~~inahl~~, Cochrane Library, Web of Science, AMED, PEDro, and Google Scholar from the earliest available dates through January 2025. We included randomised and non-randomised studies~~controlled trials, controlled before-after studies, and interrupted time-series~~ that directly compared interventions consisting of CDS presented on-screen or on paper to healthcare practitioners aiming to prevent falls in persons aged ~~≥~~65 years or older. We analysed healthcare practitioner performance, medication review and prescribing, fall risk, fall rate, and fall injury rate as primary outcomes. Two reviewers independently screened studies and assessed for risk of bias. We synthesi~~z~~sed results using meta-analyses and vote-counting based on direction of effect, when possible, otherwise narratively, and we rated the certainty of the evidence using the GRADE approach.

Results: Of 25 included studies, 20 were randomised and five were non-randomised. Most CDS tools supported healthcare practitioners in performing multifactorial fall risk assessments and follow-up interventions based on identified risks (60%) and most were delivered electronically (60%). CDS ~~interventions~~ may improve healthcare practitioners' adherence to recommended practice (all ~~8~~ eight comparisons favouring CDS; 95%

confidence interval [~~(~~CI~~)~~] 68%~~,~~ to 100%; ~~very~~ low certainty) and likely improve medication review and prescribing (all nine comparisons favouring CDS; 95% CI 70%~~,~~ to 100%; ~~low~~moderate certainty), although the effect sizes are uncertain. CDS ~~interventions likely~~may reduce fall risk, but the effect may be small ~~slightly~~ (odds ratio 0.93; 95% CI 0.81~~,~~ to 1.01; ~~moderate~~low certainty).~~,~~ CDS likely reduces fall rates in hospitals or residential care (rate ratio [~~(~~RaR~~)~~] 0.74; 95% CI 0.63~~,~~ to 0.88; moderate certainty) and in patients aged 80 years or older (RaR 0.72; 95% CI 0.61~~,~~ to 0.86; moderate certainty). CDS may reduce fall rates ~~, and likely have little to no impact on fall rate among~~in community-dwelling older adults (RaR 0.97; 95% CI 0.93~~,~~ to 1.00; moderate certainty) and in patients aged between 65 and 80 years (RaR 0.92; 95% CI 0.84 to 1.01; low certainty), though the effects in both of these subgroups may be small. CDS ~~interventions~~ may reduce fall injury rates in older adults aged between 65 and 80 years (RaR 0.80; 95% CI 0.59~~,~~ to 1.09; low certainty).~~, but t~~ The evidence on fall injury rates in patients aged 80 years or older was very uncertain.

Conclusion: CDS ~~interventions may~~ likely enhances healthcare ~~practitioners'~~ practitioners’ performance in fall prevention among older adults ~~and probably reduce falls in hospitals and residential care settings~~; however, the effect sizes remain unknown. Although CDS may improve patient outcomes in fall prevention, both the effect sizes and the certainty of evidence vary.~~However, they likely have little to no impact on fall rates among community-dwelling older adults. While CDS interventions may reduce fall injuries in adults aged 65 to 80 years, their effect on fall injuries in adults aged 80 years or older remains very uncertain.~~ Results from this study may inform the planning and implementation of CDS in fall prevention. Future studies should strive for clearer reporting of CDS design factors to allow for an evaluation of which factors may influence the success of CDS interventions used in fall prevention.

Registration: PROSPERO, CRD42021250500

Key words: Falls; Fall Prevention; Clinical Decision Support; Implementation; Evidence-based practice; Healthcare practitioners.

Introduction

Falls are a major cause of morbidity and mortality in community-dwelling older adults aged 65 years or older [1–3]. Annually, one in three older adults experiences a fall, which ~~can result in injuries, fractures, hospitalisations, disability, social isolation or death. Falls~~ can lead to~~cause~~ significant health loss ~~that leads to~~and increased~~more complex~~ care needs~~requirements~~ [1–5]. Globally, fall-related injuries are one of the most expensive conditions in terms of economic expenditures [6–8]. A history of falls, higher age, female sex, and fear of falling are among the multitude of identified risk factors for falls ~~that have been identified~~ [9,10]. There are ~~A~~a variety of ~~I~~interventions aimed at~~to~~ preventing falls in

older adults that are cost-effective [11,12] and are associated with a reduction~~s~~ in the number of falls and fall-related injuries [11,13]. These interventions include muscle strength and balance training, home safety assessments and modifications, and medication adjustments [11,12]. Falls are often ~~go~~ under~~ the radar~~reported, ~~and~~ as older adults ~~often will~~may not ~~spontaneously~~ report falls unprompted [14,15].~~–~~ The World Falls Guidelines 2022 (WFG2022) [16] emphasise the need to identify older adults at increased fall risk and recommend that all older adults ~~should be~~receive advise~~d~~ on fall prevention and physical activity.

Despite ~~the~~ scientific support ~~to~~ for the implementation of fall-prevention recommendations for ~~fall prevention among~~ older adults, the systematic uptake of evidence-based fall prevention practices has been slow [16–19], and fall rates and fall-related mortality~~deaths from falls have not been reduced~~ have not declined [3,20,21]. The WFG2022 recommend the systematic use of Clinical Decision Support (CDS) in fall prevention to identify ~~increased fall risk in~~ older adults at increased risk of falling and to facilitate~~offer~~ fall risk assessments and interventions. CDS is an implementation strategy found to improve healthcare practitioners' adherence to clinical guidelines ~~among healthcare practitioners~~ [22]. CDS is defined as computerised or non-computerised tools that combine health-related and medical information with individual patient information to support clinical decision-making [23,24]. CDS may aid healthcare practitioners in identifying older adults at increased fall risk [25–27] as well as in individualising~~tailoring~~ fall prevention interventions based on identified risk factors [26,28–30].

Several systematic reviews have evaluated the effects of CDS used by healthcare practitioners across a variety of settings, demonstrating ~~and have shown~~small to moderate

positive effects~~results~~ [22,31–39]. Generally, the use of CDS ~~used~~ by healthcare practitioners may improve the implementation of clinical practice guidelines [22] and the performance of nurses and allied health professionals [31,36,37]. In addition, CDS may improve the hospital care of older patients [32], ~~and~~ medication outcomes in older adults [33,34], and in adults in general [38,39]. The effect on patient outcomes seems less certain [37]~~,~~ ; ~~however~~ while the use of CDS by healthcare practitioners may not affect mortality, ~~but~~ it may moderately improve morbidity outcomes [35] and a variety of other patient outcomes [38]. ~~While~~ Although two systematic reviews found that CDS tools may prevent falls in nursing homes [31] and in hospitals [32], these reviews did not identify ~~several~~ multiple known studies on the effects of CDS in fall prevention [40,41], and no statistical syntheses were conducted. Findings from systematic reviews indicate that the use of CDS by healthcare practitioners may provide important benefits ~~on~~ in terms of healthcare practitioners' performance and ~~on~~ patient outcomes. However, no systematic review has investigated the effects of CDS on the performance of healthcare practitioners and ~~on~~ patient outcomes ~~exclusively~~ in the context of fall prevention.

Objectives

This systematic review aimed to evaluate the effects of CDS for~~used in the implementation of~~ fall prevention on healthcare practitioners' adherence to recommended practice, ~~and~~ medication outcomes, and on patient outcomes~~ such as fall rate and fall risk~~.

Materials and methods

Protocol and registration

The review protocol was drafted and finalised according to the Preferred Reporting Items for Systematic Reviews and Meta-Analyses (PRISMA) extension for study protocols (PRISMA-P) [42] and was registered in the PROSPERO ~~I~~international prospective register of systematic reviews (identification number CRD42021250500) prior to commencing the systematic review. We followed the recommendations in the Cochrane Handbook [43] and the guidance from the Cochrane Effective Practice and Organisation of Care (EPOC) [44]. The reporting of this systematic review was guided by the standards of the Preferred Reporting Items for Systematic Reviews and Meta-A~~a~~nalyses (PRISMA) [45]. See S1 Table for the populated PRISMA checklist. See S2 Table for differences between the protocol and the review.

Eligibility criteria

We included studies on ~~the effects of~~how CDS interventions directed ~~towards~~ at healthcare practitioners, with a view to supporting their decisions aiming to prevent falls in older adults in any healthcare setting, affected ~~on~~ healthcare practitioner performance and patient outcomes. We included randomised and non-randomised controlled trials, controlled before-after studies, and interrupted time-series studies. Detailed eligibility criteria are presented in Table 1.

Table 1. Study eligibility criteria.

	Inclusion criteria	Exclusion criteria
Participants	Healthcare practitioners (nurses, physiotherapists, general practitioners, occupational therapists,	Studies of CDS used by students.

	nursing assistants, pharmacists, physicians, primary care providers, paramedics)	
Intervention	CDS used by healthcare practitioners in fall prevention interventions. We included both computerised and non-computerised CDS interventions.	Studies where CDS was not part of the intervention in at least one study group or arm.
Comparison	Usual care or no treatment.	
Outcomes	We included studies that reported at least one primary or secondary outcome.<br>Primary:<br>• Healthcare practitioner performance: Adherence to recommended practice and ~~medication outcomes, i.e.,~~ adherence to recommended medication prescribing and review.<br>• Patient outcomes: Rate of falls (i.e., number of falls per unit of follow-up time); risk of falling (i.e., the number of older adults who had one or more falls); and fall injuries.<br>Secondary:<br>• Deaths and hospitalisations.	Studies that did not report at least one primary or secondary outcome.
Design	RCTs: IRPGT and CRT; NRCTs; CBA studies; and ITS studies [44].	Uncontrolled before-after, cross-sectional, case-control, and cohort studies; as well as protocols, editorials, opinion papers, and conference abstracts.<br>Studies without a control group, except for ITS studies with multiple data points collected before and after the implementation of the intervention.
Setting	Any healthcare setting or in the homes of older adults.	Studies conducted in a setting other than a healthcare institution or in the homes of older adults.

CDS: Clinical Decision Support; RCT: Randomised controlled trial; IRPGT: Individually-randomised parallel-group trial; CRT: Cluster-randomised trial; NRCT: Non-randomised controlled trial; CBA: Controlled before-after study; ITS: Interrupted time-series

Information sources and search strategy

We searched the following databases from the earliest available date through January 2025: MEDLINE (Ovid), EMBASE (Ovid), CINAHL~~inahl~~ (EBSCOhost), The Cochrane Library, Web of Science, and AMED (Ovid). Supplemental searches were performed in Google Scholar (S3 Table). The literature search strategy was developed in cooperation with two librarians from the Literature Search Resource/Expert Group at Oslo Metropolitan University (OsloMet). The searches were developed to be highly sensitive and included terms

synonymous with the fundamental concepts ~~of~~ underlying CDS (e.g., decision rules, reminder systems, algorithms), falls (e.g., accidental falls, slip), and fall prevention (e.g., accident prevention, safety management). We did not impose any language or study design restrictions on the literature searches. ~~Study design was not restricted in the search strategy t~~To maximi~~z~~se the sensitivity of the search and capture a broader range of potentially relevant studies, study design was not restricted in the search strategy. One reviewer (RS) used the advanced search feature ~~conducted manual searches~~ in PEDro to conduct manual searches~~using the advanced search feature~~ for relevant clinical trials. Additionally, we searched the reference lists of the included articles and relevant systematic reviews. Cited reference searching for all included articles was carried out in Web of Science. See S3 Table for the complete search strategy.

Selection process

Search results were imported into EndNote, ~~software~~ where duplicates were removed. Next, all unique abstracts and full text articles were uploaded to the Covidence systematic review software [46]. Two reviewers (RS and either TB, NRO, or LAHK) independently screened titles, ~~and~~ abstracts, and full texts against the eligibility criteria, with any disagreements resolved by consensus or by the decision of a third reviewer. Consensus between reviewers was required during both the title and abstract screening stage and the full-text screening stage.

Data collection process

We used an adapted version of the EPOC data collection form to extract relevant data from the included studies. The data extraction form was piloted on three reports by two reviewers.

One reviewer (RS) independently extracted the data, and another reviewer (TB) checked the extracted data against five arbitrarily selected papers. We resolved any disagreements between reviewers by consensus, and if necessary, a third reviewer was consulted. To address missing outcome data, we contacted the corresponding authors of eight included reports via email. If no responses were received, follow-up emails were sent after two weeks, with a maximum of three email attempts per author. Our efforts to obtain missing data were successful in four instances. All data used in the analyses were obtained directly from primary sources or through successful author contact, with no data imputation performed.

Data items

Outcomes

For healthcare practitioner performance, we sought data on adherence to recommended practice, i.e., the extent to which healthcare practitioners gave specific advice, delivered specific interventions, or followed referral guidelines [44]~~;~~, as well as data on ~~and~~ medication outcomes including medication review [47] and prescribing. For patient outcomes, we included fall risk, fall rate, and fall injury rate. Fall risk refers to the proportion of individuals who experienced at least one fall over a given period – this outcome quantifies the likelihood of falling at the person level. Fall rate and fall injury rate ~~measure~~refer to the total number of falls or fall injuries per unit of time, e.g., per 1,000 person-years, accounting for multiple falls per individual. Included in fall injuries were both minor injuries, such as bruises or abrasions, ~~as well as~~and fractures or other serious injuries requiring medical attention [48]. We also sought data for deaths and hospitalisations. If more than one result was available from a particular study, we used the primary outcome as specified by the authors, or the one that was used for sample size calculation. If study authors did not specify

primary outcomes, we prioritised results ~~judged~~deemed to be ~~more~~ most ~~over less~~ relevant to the research question, those~~results~~ indicating drug overuse ~~over~~ rather than misuse or underuse (~~because of~~due to the significant fall risk imposed by polypharmacy [49]), and results based on whole-group ~~over sub-group~~ analyses rather than subgroup analyses.

Study characteristics

We extracted the following data on study characteristics: authors, publication year, funding, country, design, setting, duration, healthcare practitioners delivering the intervention (type and number), and patients receiving the intervention (number, sex, age).

Interventions and CDS

We extracted the characteristics of experimental and control interventions using the Template for Intervention Description and Replication (TIDieR) checklist [50], and using domains 1 and 3 from the intervention complexity assessment tool for systematic reviews (iCAT_SR) [51]. Interventions were categorised into 1) manual fall risk assessment and interventions based on CDS; 2) medication review and recommendations made to physician; 3) automatically generated fall risk based on prediction models and recommended interventions; 4) guided medication dosing using computerised CDS; and 5) computerised CDS presented to paramedics on hand-held tablets [52,53]. Design factors of ~~the~~ CDS tools were extracted using the GUideline Implementation with DEcision Support (GUIDES) framework [53] and elements from the two-stream model [54].

Study risk of bias assessment

Two review authors independently assessed risk of bias (RoB) for ~~the main~~ outcomes indicating healthcare practitioner performance, fall rates, fall risk, and fall injuries ~~results~~. We used the Revised Cochrane Risk of Bias tool for randomised trials (RoB 2.0) [55] for included RCTs, and the Risk Of Bias In Non-randomised Studies of Interventions (ROBINS-I) tool [56,57] for included non-randomised studies. Any disagreements between reviewers were resolved by consensus, and if necessary, a third reviewer was consulted.

Synthesis methods and effect measures

Healthcare practitioner performance

We performed two analyses related to healthcare practitioner performance, both of which used vote-counting based on direction of effect [43,58]. In the analysis of adherence to recommended practice, we included outcomes indicating the use of recommended intervention components or adherence to the intervention protocol or to the referral guideline [44]. In the analysis of medication outcomes, we included outcomes indicating changes in the use of fall-risk-increasing drugs (FRIDs), polypharmacy, drug underuse, or drug-related problems [33]. To decide which results were eligible for the analyses, we tabulated each study result and compared it against the eligibility criteria. Each effect estimate was dichotomised into ‘favouring CDS’ or ‘favouring control’, based on the observed direction of effect alone. We used the sign test for differences in proportions and presented the proportion of results favouring CDS along with 95% confidence intervals (CIs) using the Wilson interval method [59]. The data used for analyses of healthcare practitioner performance outcomes are available in S1 Appendix.

Patient outcomes

Data on fall risk, fall rate, and fall injury rate were pooled into meta-analyses in line with previous work [11,60]. We assumed that underlying study effects followed a normal distribution and used random effects models and restricted maximum likelihood (REML) estimation methods [61]. Stata, version 18.0, StataCorp, College Station, Texas [62], was used for all analyses and pooled results were presented in forest plots. Results were presented as odds ratios (OR) for fall risk, and as rate ratios (RaR) for fall rate and fall injury rate, along with 95% CIs. Variation between study results (heterogeneity) was assessed ~~with~~ by means of a visual inspection of forest plots and the I^2 statistic [61]. If I^2 values were 50% or higher, we sought potential explanations for the heterogeneity using post hoc subgroup analyses by RoB (low or some concerns for RoB versus high or serious RoB), study setting (community-dwelling v~~s.~~ersus hospital or residential care), and patient age (mean age of < 80 years versus ≥ 80 years). If point estimates and confidence intervals were missing but other information was available, e.g., number of events and the follow-up time in both comparison groups, ~~was available,~~ we used established formulas to estimate the point estimates and confidence intervals [63]. Only one outcome per study was included in the analyses to ensure independence between studies. The data used for meta-analyses are available in S2 Appendix. Control intervention fall risks, fall rates, and fall injury rates were derived from a US report on the epidemiology of falls among older adults [64], due to the limited reporting of absolute numbers in the studies included in this review. To report the absolute effects on fall risk, odds ratios were first converted to relative risks using the formula provided in appendix 3 of the Core GRADE 2 article [65].

Publication bias assessment

The possibility of publication bias was assessed ~~by~~ through inspection of funnel plots of effect estimates against their standard errors for analyses that contained at least 10 effect estimates [43]. The Egger test for small-study effects and the trim-and-fill analysis were used for quantitative assessment of ~~to assess~~ publication bias ~~quantitatively~~ [66].

Assessment of certainty of the evidence

We judged the certainty of the evidence using the Grading of Recommendations Assessment, Development and Evaluation (GRADE) approach [65,67,68], applied to ~~for~~ each outcome ~~reported~~ from vote-counting ~~-~~analyses and meta-analyses. Despite extensive literature searches conducted with the assistance of two librarians at OsloMet, no minimal important difference (MID) values were identified for fall risk, fall rate, or fall injury rate. Certainty was therefore rated based on whether the true effect lies on the observed side of ~~the~~ null, with the null effect used as the threshold. Relative and absolute effects were reported alongside plain-language statements, following recommendations from GRADE guidance~~In line with GRADE guidance, we focused on the point estimate and certainty of the evidence when describing the results related to healthcare practitioner performance and patient outcomes~~ [69,70].

Results

Study selection

Fig 1 presents an overview of the study selection process. Our primary database searches identified 6,572 unique records, of which 6,527 were excluded after title and abstract screening. A total of 28 publications describing 25 unique studies were selected for inclusion [25–30,40,41,71–90]. S4 Table provides a summary of the excluded studies and the reason for exclusion.

Fig 1. PRISMA flow diagram.

Study characteristics

Study design, participants, and settings

Table 2 presents an overview of the characteristics of the included studies. The 25 studies comprised 13 cluster-randomised trials (CRTs), seven individually randomised parallel group trials (IRPGTs), four non-randomised controlled trials (NRCTs), and one controlled before-after (CBA) study. The study duration was median (min–max) 12 (1–60) months. The interventions were primarily delivered by nurses (16; 64%), physicians (15; 60%), pharmacists (6; 24%), and/or physiotherapists (4; 16%) to a median number ~~(min–max)~~ of 1,433 patient participants, ranging from ~~(~~312–46,245~~) patient participants~~. Twelve (48%) studies had eligibility criteria related to increased risk for falls, such as a history of previous

falls or the use of fall-risk-increasing drugs. Most studies were conducted in hospitals (44%) or in primary care practices (36%) in North American (52%) and European (28%) countries.

Table 2. Characteristics of included studies.

Author & year	Country	Design	Setting	Study duration (months)	Healthcare practitioners	Patients	Outcomes
Aizen 2015	Israel	CRT	Rehabilitation geriatric hospital	6	Nurses<br>n = ?	Patients admitted to hospital<br>n = 508 (200 in CDS and 308 in CG), 52.2% female, mean 84.3 years	-Rate of falls
Barker 2016	Australia	CRT	Hospital	12	Nurses<br>n = ?	Patients admitted to hospital<br>n = 35,264 (17,698 in CDS and 17,566 in CG), 49.5% female, median 67.5 years	-Adherence to recommended practice<br>-Rate of falls<br>-Rate of fall injuries
Bhasin 2020	USA	CRT	~~Primay~~Primary care practices	44	Nurses<br>n = ?	Community-dwelling at increased risk for fall injuries<br>n = 5,451 (2,802 in CDS and 2,649 in CG), 62% female, mean 79.7 years	-Rate of fall injuries
Blalock 2020	USA	CRT	Community pharmacies	24	Pharmacists<br>n = ?	Adults ≥65 years using either four or more chronic medications or ≥1 medication associated with increased fall risk, n = 3,213 (1,467 in CDS and 1,745 in CG)	-Medication outcomes<br>-Risk of falling
Blum 2021	Switzerland, Netherlands, Belgium, Republic of Ireland	CRT	Hospitals	12	Physicians and pharmacists<br>n = ?	Adults years with multimorbidity (≥3 chronic conditions) and polypharmacy (≥5 drugs used long term)<br>n = 2,008 (963 in CDS and 1,045 in CG), 44.7% female, median 79 years	-Rate of falls<br>-Medication outcomes<br>-Mortality<br>-Hospital admissions
Byrne 2005	USA	CBA	Nursing homes	33	Nurses<br>n = 153	n = ?, ? females, Mean age in IG: 82.5 (group 1) and 80.9 (group 2); in CG: 79.2 (group 5) and 82.2 (group 6)	- Rate of falls
Carroll 2012	USA	CRT	Urban hospitals (academic medical ~~centers~~centres & community hospitals)	6	Nurses<br>n = ?	Patients admitted during study period<br>n = 364, ? female, ? age	-Adherence to recommended practice
Dykes 2010						Patients admitted during study period<br>n = 10,264 (5,160 in CDS and 5,104 in CG), 54.5% female, mean 78.8 years (among patients 65 years or older)	-Rate of falls<br>-Rate of fall injuries<br>-Adherence to recommended practice
Clemson 2024	Australia	CRT	Primary care practices	12	Physicians<br>n = 75<br>Allied health professionals n = 342	Community-dwelling older adults who had had a fall in the past year or were concerned about falling<br>n = 560 (275 in CDS and 285 in CG), 67.9 % female, mean 78.6 years	-Rate of falls<br>-Adherence to recommended practice
Dykes 2020	USA	NRCT	Hospital (academic medical centres)	42	Nurses<br>n = ?	Patients admitted during study period<br>n = 37,231 (17,948 in pre-intervention group and 19,283 in post-intervention group), 53.8% female, mean 60.8 years	-Rate of falls<br>-Rate of fall injuries
Elley 2008	New Zealand	IRPGT	Primary care practices	12	Nurses<br>n = 54	Adults who had fallen in the past 12 months<br>n = 312 (155 in CDS and 157 in CG), 68.9% female, mean 80.8 years	-Rate of falls
Ferrer 2014	Spain	IRPGT	Primary care practices	24	Physicians and nurses<br>n = ?	Community-dwelling older adults born in 1924 (85 years of age at study start)<br>n = 328 (164 in CDS and 164 in CG), 61.1% female	-Risk of falling<br>-Time to first, second, and recurrent falls<br>-Hospital admissions
Frankenthal 2014	Israel	IRPGT	Hospital (Chronic care geriatric facility)	12	Pharmacist<br>n = ?	Residents prescribed at least one medication<br>n = 359 (183 in CDS and 176 in CG), 66.6% female, mean 82.7 years	-Rate of falls<br>-Hospital admissions
Gallagher 2011	Ireland	IRPGT	University hospital	6	Physicians<br>n = ?	Patients admitted via the emergency department under care of a GP<br>n = 382 (190 in CDS and 192 in CG), 53.1% female, median 74.5 years (IG) and 77 (CG)	-Medication outcomes<br>-Risk of falling<br>-Hospital admissions
Ganz 2015	USA	NRCT	Primary care practices	24	Physicians, nurse practitioner, physician assistant<br>n = 44	Patients who screened positive for fall risk<br>n = 1,791 (1,187 in CDS and 604 in control), 72% female, mean 82.9 years	-Rate of fall injuries
Wenger 2010				12		Patients who screened positive for falls or fear of falling and UI:<br>n = 1,211 (586 in CDS and 625 in CG), 71.7% female, mean 83 years	-Adherence to recommended practice

Ganz 2022	USA	CRT	Primary care practices	60	Nurses<br>n = ?	Community-living persons at increased risk for serious fall injuries<br>n = 5,451 (2,802 in CDS and 2,649 in CG), 62% female, mean 79.7 years	-Rate of falls<br>-Rate of falls leading to medical attention<br>-Hospital admissions
Groshaus 2012	Canada	NRCT	Acute care hospitals	3	Nurses<br>n = ?	Patients residing on study units<br>n = ?, ? female, ? years	-Adherence to recommended practice<br>-Risk of falling
Healey 2004	UK	CRT	District general hospital	12	Nurses<br>n = ?	All older adults who received care in the wards during the study period<br>n = 1,654 (905 in CDS and 749 in CG), 60% female, mean 81.3 years	-Rate of falls<br>-Rate of fall injuries
Lightbody 2002	UK	IRPGT	University hospital	6	Nurses<br>n = ?	Older adults discharged from Accident and Emergency Department after a fall<br>n = 348 (171 in CDS and 177 in CG), 74.4% female, median 75 years	-Medication outcomes<br>-Risk of falling<br>-Rate of falls<br>-Hospital admissions
Logan 2021	UK	CRT	Long-term care homes	12	Care home staff<br>n = 3,609	Long-term care home residents in care homes for older adults<br>n = 1,657 (775 in CDS and 882 in CG), 67.9% female, mean 85 years	-Rate of falls<br>-Mortality
Mahoney 2007	USA	IRPGT	Home visits	12	Registered nurse, physical therapist, physician<br>n = ?	Community-dwelling adults with two falls in the past year or one fall in the previous two years with injury or balance problems<br>n = 349 (174 in CDS and 175 in CG), 78.5% female, mean 80 years	-Rate of falls<br>-Hospital admissions
Peterson 2007	USA	IRPGT	Tertiary care hospital	9	Physicians<br>n = 778	Inpatients ≥ receiving care on one of the order entry wards (i.e., emergency room, intensive care units, subacute units)<br>n = 2,981, ? female, = years	-Adherence to recommended practice
Phelan 2024	USA	CRT	Primary care practices	18	Physicians	Community-dwelling adults aged ≥ 60 years, prescribed at least 1 medication from any of 5 targeted medication classes (opioids, sedative-hypnotics, skeletal muscle relaxants, tricyclic antidepressants, and first-generation antihistamines for at least 3 consecutive months<br>n = 2,367 (1,106 in CDS and 1,261 in CG), 63% female, mean 70.6	-Time to first medically treated fall (risk of fall injuries)<br>-Medication outcomes
Snooks 2014	UK	CRT	Emergency ambulance services	1	Paramedics<br>n = 42	Community-dwelling older adults living in the catchment area of a participating falls service<br>n = 779 (436 in CDS and 343 in CG), 63.4% female, median 82.5 years	-Adherence to recommended practice<br>-Risk of falling<br>-Mortality<br>-Hospital admissions
Tamblyn 2012	Canada	CRT	Primary care practices	23	GPs<br>n = 81	Patients with a prescription for a psychotropic drug<br>n = 5,628 (2,887 in CDS and 2,741 in CG), 67.1% female, mean 75.2 years	-Medication outcomes
Weber 2008	USA	CRT	Primary care practices	15	Pharmacists and GPs<br>n = ?	Community-dwelling patients at risk for falls<br>n = 620 (413 in CDS and 207 in CG), 79.2% female, mean 76.9 years	-Risk of falling<br>-Medication outcomes
Wenger 2009	USA	NRCT	Primary care practices	32	Physicians<br>n = 40	Community-dwelling patients who had at least one of three geriatric conditions: falls and gait impairment, urinary incontinence, and cognitive impairment<br>n = 644 (357 in CDS and 287 in CG), 66% female, mean 81 years	-Adherence to recommended practice

CBA: Controlled Before-After study; CCDS: Computerised Clinical Decision Support; CDS: Clinical Decision Support group; CG: Control group; CRT: Cluster-Randomised Trial; ED: Emergency Department; GP: General Practitioner; IG: Intervention Group; ITS: Interrupted Time-Series study; IRPGT: Individually-Randomised Parallel-Group Trial; NRCT: Non-Randomised Controlled Trial; UI: Urinary Incontinence

Interventions and CDS tools

Table 3 presents an overview of the characteristics of the interventions and CDS tools assessed in the included studies. Most (15; 60%) interventions delivered CDS to healthcare practitioners to aid with fall risk assessments, followed by an offer to the patient of specific fall prevention interventions. A total of 22 (88%) interventions consisted of more than one component ~~that were~~ delivered as a bundle, i.e., there was a defined order in the delivery of the components such as medication review prior to medication changes. Most interventions were directed at either one type of healthcare practitioner (12; 48%) or at two or more types of healthcare practitioners in the same healthcare setting (11; 44%). See S5 Table for a detailed description of the interventions. Most CDS tools were designed as algorithms presented as if-then-statements or as checklists with risk factors for falls, followed by recommendations for fall prevention interventions that were either generated automatically or chosen manually from a list (68%). Fifteen (60%) of the CDS tools were delivered electronically, three (12%) were delivered both electronically and on paper, two (8%) were paper-based, and for five (20%) studies, this remained unclear. Among the electronically delivered CDS interventions, nine (36%) included alerts, reminders, or prompts, whereas in the remaining studies, decision support was delivered on demand. The control interventions included usual care (76%), usual care plus a falls information pamphlet (4%), usual care plus an offer of two social visits (4%), home safety visits (4%), paper-based CDS (4%), and no treatment (4%). One study (4%) failed to mention control. See S6 Table for a detailed description of the CDS design factors.

Table 3. Characteristics of interventions and Clinical Decision Support.

Study	Type of intervention	Active components	n types of intervention deliverers	Organisational levels	CDS format	CDS features
Aizen 2015	1	High	1: Nurses	Low	Electronic	Risk assessment tool with intervention recommendations based on risk

Barker 2016	1	High	3: Nurse, site clinical leader, champion	Intermediate	-	Checklist with risk factors and recommended interventions; Reminders on the use of intervention components
Bhasin 2020, Ganz 2022	1	High	3: Nurse, primary care physician, pharmacist	Intermediate	Electronic	Algorithm with risk factors and intervention recommendations
Blalock 2020	2	High	2: Pharmacist, primary care physician	Intermediate	Electronic	Adapted STEADI algorithm with risk factors and intervention recommendations
Blum 2021	2	High	3: Pharmacist, hospital physician, general practitioner	High	Electronic	Lists of FRIDs (STOPP/START criteria)
Byrne 2005	3	High	1: Nurse	Low	Electronic	Automatically generated fall risk estimates and intervention recommendations
Carroll 2012, Dykes 2010	1	High	1: Nurse	Low	Electronic and paper-based	Alerts printed on paper to hang over bed
Clemson 2024	1	High	> 7: Primary care physicians, physiotherapists, occupational therapists, nurses, podiatrists, pharmacists, exercise physiologists, and other professions	High	Electronic and paper-based	iSOLVE algorithm; Fall risk assessment checklist; GP fall risk assessment chart; Tailoring interventions to fall risk chart; Risk information automatically sent to GP; Case studies which illustrate the algorithm and tailoring options; Examples of how to talk with patients about falls
Dykes 2020	1	High	1: Nurse	Low	Electronic	Checklist with risk factors and recommended interventions
Elley 2008	1	High	3: Nurse, trained practitioner, physiotherapist	Intermediate	-	Algorithm with risk factors and intervention recommendations
Ferrer 2014	1	High	2: Nurse, primary care physician	Intermediate	-	Algorithm with risk factors and intervention recommendations
Frankenthal 2014	2	High	2: Pharmacist, hospital physician	Intermediate	Electronic	Lists of FRIDs (STOPP/START criteria)
Gallagher 2011	2	High	1: Hospital physician	Low	-	Lists of FRIDs (STOPP/START criteria)
Ganz 2015, Wenger 2010	1	High	3: Primary care physician, nurse, physician assistant	Intermediate	Electronic	Medical record prompts
Groshaus 2012	1	High	1: Nurse	Low	Electronic	Electronic order set
Healey 2004	1	High	1: Nurse	Low	Paper-based	Checklist with risk factors and intervention recommendations
Lightbody 2002	1	High	1: Nurse	Low	-	Checklist with risk factors and intervention recommendations
Logan 2021	1	High	3: Nurse, physiotherapist, occupational therapist	Intermediate	Paper-based	Checklist with risk factors and intervention recommendations
Mahoney 2007	1	High	3: Nurse, physiotherapist, primary care physician	Intermediate	Electronic	Algorithm with automatically generated intervention recommendations
Peterson 2007	4	Low	1: Hospital physician	Low	Electronic	Guided medication dosing; Prompts presented on-screen
Phelan 2024	2	Intermediate	1: Primary care physician	Low	Electronic	Evidence-based pharmaceutical opinions; Deprescribing pearls with conversation starters
Snooks 2014	5	High	1: Paramedic	Low	Electronic	Prompts to start assessment; Algorithm with automatically generated care plan
Tamblyn 2012	4	Low	1: Primary care physician	Low	Electronic	Predictive model to automatically estimate risk of injury; Alerts when patient was prescribed a FRID; Graphics presenting risk estimates; Guided medication dosing
Weber 2008	2	High	3: Pharmacist, geriatrician, primary care physician	Intermediate	Electronic	Guided medication dosing; Alerts with patient's fall risk

Wenger 2009	1	High	3: Nurse, primary care physician, medical assistant	Intermediate	Electronic and paper-based	Medical record prompts with suggestions for appropriate action

CDS: Clinical Decision Support; FRIDs: Fall-risk-increasing drugs; GP: General practitioner; STEADI: Stopping Elderly Accidents, DEaths, and Injuries; STOPP/START: Screening Tool of Older Person’s Prescriptions and Screening Tool to Alert doctors to Right Treatment

Type of intervention:

1: Fall risk assessment and interventions based on CDS.

2: Medication review and recommendations made to physician.

3: Automatically generated fall risk based on prediction models, followed by recommended interventions.

4: Guided medication dosing using computerised CDS.

5: Computerised CDS presented to paramedics on hand-held tablets.

Active components (iCAT_SR domain 2):

High: More than one component and delivered as a bundle (clear order in the delivery of the components) (high level of complexity).

Intermediate: More than one component and delivered as a package (no specific order) (intermediate level of complexity).

Low: One component (low level of complexity).

Organisational levels and categories targeted by the intervention (iCAT_SR domain 3):

High: Intervention directed at two or more healthcare settings, e.g., primary care and hospitals (multi-level).

Intermediate: Intervention directed at two or more categories of healthcare practitioners within the same healthcare setting, e.g., nurse and physiotherapist in primary care (multi-category)

Low: Intervention directed at one category of healthcare practitioners, e.g., nurses (single category)

Risk of bias in included studies

A detailed description of RoB assessments is available in S7 Table. We assessed a total of 40 results across five outcomes. Out of 33 results from randomised trials, 19 results had high RoB~~;~~, 10 results had some concerns~~;~~, and four results had low RoB. All seven results from NRSI were judged to be at serious RoB, primarily owing to concerns for bias due to confounding.

Results of individual studies

Results from each individual study are available in S8 Table.

Healthcare practitioner performance

Adherence to recommended practice

Five CRTs [26,27,30,71,88] and three NRCTs [80,82,90] reported results on adherence to recommended practice (Fig 2). The common outcome across the various studies is the adherence to fall prevention interventions, including fall risk documentation, protocol adherence, and provision of recommended intervention components. The follow-up time was median (min–max) 7 (1–13) months. All eight results favoured the intervention (100% (95% CI 68%~~,~~ to 100%), $p < 0.01$). The certainty of evidence was ~~very~~ low due to risk of bias ~~and inconsistency~~ (~~S9~~ Table 4). CDS ~~interventions~~ may improve healthcare practitioners' adherence to recommended practice in fall prevention.

Fig 2. Healthcare practitioner performance results.

FU: Follow-up; IQR: Interquartile range; DBI: Drug Burden Index; IG: Intervention group; CG: Control group; RoB: Overall risk of bias judgement.

† Based on sub-group analysis and therefore not randomised

* Non-randomised study. Serious risk of bias based on ROBINS-I

Sign test:

H0: n results favouring CDS = n results favouring control

Ha: n results favouring CDS ≠ n results favouring control

Table 4. Summary of findings.

Formatted: Font: Bold

CDS interventions compared with usual care for HCPs in fall prevention among older adults

Population: HCPs, including nurses, physiotherapists, general practitioners, occupational therapists, nursing assistants, pharmacists, physicians, primary care providers, and paramedics
Settings: Hospitals, residential care, primary care, and the homes of older adults
Intervention: CDS targeted at HCPs
Comparison: Usual care

Outcomes<br><br>No of participants (studies)	Relative effects (95% CI)	Anticipated absolute effects* (95% CI)			Certainty of evidence
		In control	CDS interventions	Difference	
**Adherence to recommended practice: fall risk assessments and interventions**<br><br>Follow-up: median 7 (1 to 13) months<br><br>[a] (8)	[b]	[b]	[b]	[b]	⊕⊕OO<br><br>Low[c]<br><br>Due to very serious risk of bias
**Adherence to recommended medication review and prescribing**<br><br>Follow-up: median 9 (0 to 23) months<br><br>[a] (9)	[b]	[b]	[b]	[b]	⊕⊕⊕O<br><br>Moderate[d]<br><br>Due to risk of bias
**Fall risk**<br><br>Follow-up: median 9 (1 to 24) months<br><br>> 13,636[e] (10)	OR 0.93 (0.85 to 1.01)	287 per 1,000	273 per 1,000 (255 to 290)	14 fewer fallers per 1,000 (32 fewer to 3 more)	⊕⊕OO<br><br>Low[f]<br><br>Due to risk of bias and imprecision
**Rate of falls**					
In hospitals or residential care<br><br>Follow-up: median 6 (3 to 21) months	RaR 0.74 (0.63 to 0.88)	672 per 1,000 person-years	497 per 1,000 person-years (423 to 591)	175 fewer falls per 1,000 person-years (249 fewer to 81 fewer)	⊕⊕⊕O<br><br>Moderate[g]<br><br>Due to risk of bias

≥ 50,054[e] (8)					
In community-dwelling older adults<br><br>Follow-up: median 12 (12-24) months<br><br>7,000 (5)	RaR 0.97 (0.93 to 1.00)	672 per 1,000 person-years	652 per 1,000 person-years (625 to 672)	20 fewer falls per 1,000 person-years (47 fewer to 0 fewer)	⊕⊕OO<br><br>Low[h]<br><br>Due to risk of bias and imprecision
In patients with mean age ≥ 80 years<br><br>Follow-up: median 12 (3-12) months<br><br>6,264 (7)	RaR 0.72 (0.61 to 0.86)	672 per 1,000 person-years	484 per 1,000 person-years (410 to 578)	188 fewer falls per 1,000 person-years (262 fewer to 94 fewer)	⊕⊕⊕O<br><br>Moderate[i]<br><br>Due to risk of bias
In patients with mean age between 65 and 80 years<br><br>Follow-up: median 12 (6-24) months<br><br>84,084 (6)	RaR 0.92 (0.84 to 1.01)	672 per 1,000 person-years	618 per 1,000 person-years (564 to 679)	54 fewer falls per 1,000 person-years (108 fewer to 7 more)	⊕⊕OO<br><br>Low[j]<br><br>Due to risk of bias and imprecision
**Rate of fall injuries**					
In patients with mean age between 65 and 80 years<br><br>Follow-up: median 16.5 (6-24) months<br><br>≥ 50,979[e] (4)	RaR 0.80 (0.59 to 1.09)	164 per 1,000 person-years	131 per 1,000 person-years (97 to 179)	33 fewer fall injuries per 1,000 person-years (67 fewer to 15 more)	⊕⊕OO<br><br>Low[k]<br><br>Due to risk of bias and imprecision
In patients with mean age ≥ 80 years<br><br>Follow-up: median 9 (6-12) months<br><br>3,445 (2)	RaR 1.29 (0.99 to 1.69)	164 per 1,000 person-years	212 per 1,000 person-years (162 to 277)	48 more fall injuries per 1,000 person-years (2 fewer to 113 more)	⊕OOO<br><br>Very low[l]<br><br>Due to very serious risk of bias and imprecision

* Assuming a control group fall risk of 28.7%, a fall rate of 672 falls per 1,000 person-years, and a fall injury rate of 164 fall injuries per 1,000 person-years, based on data from Bergen et al. [64]. The risk in the intervention group is based on the assumed risk in the comparison group and the relative effect of the intervention (and its 95% CI).

CDS: clinical decision support; CI: confidence interval; HCPs: healthcare practitioners; OR: odds ratio; RR: rate ratio

Explanations

[a] The numbers of participating healthcare practitioners were not reported.

[b] Not estimable due to the use of different and incompletely reported effect measures across studies.

[c] Very serious risk of bias due to confounding, deviations from intended interventions, and selection of reported results.

[d] Risk of bias due to deviations from intended interventions.

[e] The number of participants were not reported in all studies.

[f] Risk of bias due to the randomisation process, deviations from intended interventions, and measurement of the outcome. Imprecision due to the upper bound of the 95% confidence interval crossing the null effect.

[g] Risk of bias in the randomisation process and due to deviations from intended interventions.

[h] Risk of bias due to deviations from intended interventions. Imprecision due to the upper bound of the 95% confidence interval including the null effect.

[i] Risk of bias due to deviations from intended interventions and selection of reported results.

[j] Risk of bias due to the randomisation process, confounding, and deviations from intended interventions. Imprecision due to the upper bound of the 95% confidence interval crossing the null effect.

[k] Risk of bias due to confounding deviations from intended interventions. Imprecision due to the upper bound of the 95% confidence interval crossing the null effect.

[l] Very serious risk of bias due to the randomisation process and confounding. Very serious imprecision arises because the 95% confidence interval includes both an effect favouring CDS and the possibility of appreciable harm.

Adherence to recommended ~~M~~medication review and prescribing~~outcomes~~

Five CRTs [25,41,72,74,89] and four IRPGTs [77,78,84,87] reported indicators of medication outcomes (Fig 2). The common outcome across the various studies was the reviewing and prescribing of drugs that may increase fall risk. The follow-up time was 9 (0–23) months. All nine results favoured the intervention (100% (95% CI 70%~~,~~ to 100%), p ~~-~~ < ~~-~~ 0.01). The certainty of evidence was ~~low~~moderate due to risk of bias~~ and inconsistency~~. CDS ~~interventions may~~likely improves medication reviewing and prescribing outcomes in fall prevention.

Patient outcomes

Fall risk

Nineteen of the included studies reported outcome data on fall risk (proportion of participants who fell) and/or fall rate (number of falls per x person-years), which made it possible to conduct meta-analyses on these outcomes. Ten studies were included in the meta-analysis on fall risk with a median (min–max) follow-up time of 9 (1–24) months [25,29,74,76,78,81,82,84,88,89] (Fig 3). Of these studies, six were conducted in patients aged 65 to 80 years, three in patients aged 80 years or older, and one in which the patients' ages were unknown. Furthermore, five studies were conducted in hospitals or residential care, while five focused on community-dwelling older adults. The overall estimate of the effect of CDS on fall risk was an odds ratio of 0.93 (95% CI 0.85~~,~~ to 1.01). Assuming a baseline fall risk of ~~30~~28.7% [64], a 7% reduction in the odds of falling~~number of older adults who fall~~ results in ~~21~~14 fewer fallers per 1,000 (95% CI ~~45~~32 fewer to 3 more) in older adults

receiving the intervention compared with control, during nine months of follow-up (~~S9~~ Table 4). Heterogeneity was unimportant (I^2 = 0%; p = 0.19). Visual inspection of the funnel plot showed no clear sign of small-study effects (S1 Fig). The Egger test suggested no evidence of small-study effects (p = 0.89). The trim-and-fill analysis imputed no studies and therefore made no difference in the effect estimate (S2 Fig). The certainty of the evidence was ~~moderate~~low due to risk of bias and imprecision. CDS ~~interventions~~ aimed at healthcare practitioners ~~likely~~ may reduce fall risk, but the effect may be small ~~result in little to no difference in fall risk~~.

Fig 3. Meta-analysis comparing CDS interventions with control on fall risk.

Red dashed line shows the point estimate for the meta-analysis overall.

Risk of Bias legend

Circles: Green~~ +~~: Low risk of bias (RoB); ~~y~~Yellow~~ !~~: Some concerns for RoB; ~~r~~Red~~ –~~: High RoB; ~~o~~Orange~~ –~~: Serious RoB.

1a: Bias arising from the randomisation process.

1b: Bias arising from the timing of identification or recruitment of individual participants within clusters (for cluster-randomised only).

2: Bias due to deviations from intended interventions.

3: Bias due to missing outcome data.

4: Bias in measurement of the outcome.

5: Bias in selection of the reported result.

O: Overall RoB judgement.

* Non-randomised study. ROBINS-I was used to assess RoB.

Rate of falls

Thirteen studies were included in the meta-analysis on fall rate [26,28–30,40,71,76,77,81,83–86] (Fig 4). Substantial heterogeneity was present in the effect estimate ($I^2 = 74\%$; $p < 0.01$). Subgroup analyses revealed statistically significant differences in fall rates when grouped by study setting ($p < 0.01$) and by patients' age ($p < 0.01$), and results are presented separately by study setting and by patients' age.

Regarding subgroup analysis by study setting, the median (min–max) follow-up time was 6 (3–21) months for studies conducted in hospitals or residential care, and 12 (12–24) months for studies in community-dwelling older adults. Of the eight studies conducted in hospitals or residential care, four included ~~paients~~patients aged 80 years or older, and four included patients aged 65 to 80 years. Similarly, of the five studies focusing on community-dwelling older adults, three included patients aged 80 years or older, while two included patients aged 65 to 80 years. The overall estimate of the effect of CDS on fall rate in hospitals or residential care was a rate ratio of 0.74 (95% CI 0.63~~,~~ to 0.88; $I^2 = 65\%$; $p < 0.01$). Assuming a baseline fall rate of 672 falls per 1,000 person-years [64], a 26% reduction in fall rate results in 175 fewer falls per 1,000 person-years (95% CI 249 fewer to 81 fewer) in hospitals or residential care settings~~older adults receiving the intervention compared with control~~ (~~S9~~ Table 4). The certainty of the evidence was moderate due to risk of bias. CDS ~~interventions~~ aimed at healthcare practitioners likely reduces the rate of falls in hospitals ~~or~~ and residential care. The substantial heterogeneity ($I^2 = 65\%$) suggests that the observed effects may vary across studies. In community-dwelling older adults, the overall estimate of the effect on fall rate was a rate ratio of 0.97 (95% CI 0.93~~,~~ to 1.00; $I^2 = 0\%$; $p = 0.88$). The certainty of the evidence was ~~moderate~~low due to risk of bias and imprecision. CDS ~~interventions likely~~may reduce

~~result in little to no difference in~~ fall rate in community-dwelling older adults, though the effect may be small.

Regarding the subgroup analysis by patients' age, the median (min–max) follow-up time was 12 (3–12) months for studies ~~of~~ involving patients with a mean age ≥ 80 years, and 12 (6–24) months for studies of patients with a mean age between 65 and 80 years (S3 Fig). Of the six studies including patients aged 65 to 80 years, four were conducted in hospitals or residential care, while two focused on community-dwelling older adults. Of the seven studies including patients aged 80 years or older, four were conducted in hospitals or residential care, while three ~~foucsed~~focused on community-dwelling older adults. The overall estimate of the effect of CDS on fall rate among patients with a mean age of ≥ 80 years was a rate ratio of 0.72 (95% CI 0.61~~,~~ to 0.86; I^2 = 46%; p = 0.09). Assuming a baseline fall rate of 672 falls per 1,000 person-years [64], a 28% reduction in fall rate results in 188 fewer falls per 1,000 person-years (95% CI 262 fewer to 94 fewer)~~ with a mean age of ≥ 80 years~~. The certainty of the evidence was moderate due to risk of bias. CDS ~~interventions aimed at healthcare practitioners~~ likely reduces the rate of falls among patients with a mean age of ≥ 80 years. In patients with a mean age between 65 and 80 years, the overall estimate of the effect on fall rate was a rate ratio of 0.92 (95% CI 0.84~~,~~ to 1.01; I^2 = 31%; p = 0.09). The certainty of the evidence was ~~very~~ low due to risk of bias and imprecision. CDS may reduce fall rate in patients with a mean age between 65 and 80 years, but the effect may be small. Visual inspection of the funnel plot showed no clear sign of small-study effects (S4 Fig). The Egger test suggested no evidence of small-study effects (p = 0.99). The trim-and-fill analysis imputed no studies and therefore made no difference in the effect estimate (S5 Fig). There was no statistically significant subgroup ~~-~~difference in fall rate when stratified by RoB (p = 0.47) (S6 Fig).

Fig 4. Meta-analysis comparing CDS interventions with control on fall rate, subgroup analysis by study setting.

Red dashed line shows the point estimate for the meta-analysis overall.

<u>Risk of Bias legend</u>

Circles: Green~~ +~~: Low risk of bias (RoB); ~~y~~Yellow~~ !~~: Some concerns for RoB; ~~r~~Red~~ –~~: High RoB; O~~o~~range~~ –~~: Serious RoB.

1a: Bias arising from the randomisation process.

1b: Bias arising from the timing of identification or recruitment of individual participants within clusters (for cluster-randomised only).

2: Bias due to deviations from intended interventions.

3: Bias due to missing outcome data.

4: Bias in measurement of the outcome.

5: Bias in selection of the reported result.

O: Overall RoB judgement.

* Non-randomised study. ROBINS-I was used to assess RoB.

Rate of fall injuries

Six studies were included in the meta-analysis on fall injury rate [26,28,30,79,81,83] (Fig 5). Substantial heterogeneity was present in the effect estimate (I^2 = 71%, p = 0.03). Subgroup analyses revealed a statistically significant difference in fall injury rate between patients with a mean age between 65 and 80 years and patients with a mean age of 80 years or older (p = 0.02), and results are presented separately by patients’ age. The median (min–max) follow-up time was 16.5 (6–24) months for studies conducted in patients with a mean age between 65

and 80 years and 9 (6–12) months for studies in patients with a mean age of 80 years or older. The overall estimate of the effect of CDS ~~to~~ in preventing fall injuries in patients with a mean age between 65 and 80 years was a rate ratio of 0.80 (95% CI 0.59~~,~~ to 1.09; $I^2 = 67\%$; p = 0.08), which indicates a statistically non-significant result as the confidence interval crosses the null effect. Assuming a baseline fall injury rate of 164 fall injuries per 1,000 person-years [64], a 20% reduction in fall injury rate results in 33 fewer fall injuries per 1,000 person-years (95% CI 67 fewer to 15 more) in older adults receiving the intervention compared with control (~~S9~~ Table 4). The certainty of the evidence was low due to risk of bias and imprecision. CDS interventions aimed at healthcare practitioners may reduce the fall injury rate in patients with a mean age between 65 and 80 years.

Fig 5. Meta-analysis comparing CDS interventions with control on fall injury rate, subgroup analysis by patients' age.

Red dashed line shows the point estimate for the meta-analysis overall.

Circles: Green~~ +~~: Low risk of bias (RoB); ~~y~~Yellow~~ !~~: Some concerns for RoB; ~~r~~Red~~ –~~: High RoB; ~~o~~Orange~~ –~~: Serious RoB.

1a: Bias arising from the randomisation process.

1b: Bias arising from the timing of identification or recruitment of individual participants within clusters (for cluster-randomised only).

2: Bias due to deviations from intended interventions.

3: Bias due to missing outcome data.

4: Bias in measurement of the outcome.

5: Bias in selection of the reported result.

O: Overall RoB judgement.

*   Non-randomised study. ROBINS-I was used to assess RoB.

In patients with a mean age of 80 years or older, the overall estimate of the effect on fall injury rate was a rate ratio of 1.29 (95% CI 0.99~~,~~ to 1.69; I^2 = 0%; p = 0.84). ~~Certainty was rated down twice for risk of bias and once for imprecision.~~ The certainty of the effect estimate of CDS interventions on fall injury rate in patients with a mean age of 80 years or older is very low due to risk of bias and imprecision. We are very uncertain about the effect of CDS on fall injury rates in patients aged 80 years or older. There is no statistically significant subgroup ~~-~~difference in fall injury rate when stratified by RoB (p = 0.93) or study setting (p = 0.41). See S7 and S8 Figs for subgroup analyses on fall injury rate.

Mortality and hospitalisations

Five CRTs [72–74,86,88] reported on mortality~~;~~, and five IRPGTs [76–78,84,85] and three CRTs [73,74,88] reported hospital admissions. ~~No studies found statistically significant differences between groups in mortality or hospital admissions.~~ The sample sizes were small, the outcomes were rare, and no consistent patterns were observed in the direction of effect for mortality or hospital admissions. For further details on individual study results, see S8 Table.

Certainty of the evidence

Certainty of the evidence was assessed for nine outcomes, the results from which are available in a GRADE evidence profile in S9 Table and in Table 4.

Discussion

This systematic review summari~~z~~sed ~~the effects from~~ 25 studies (28 publications) of healthcare professionals’ use of CDS ~~used by healthcare practitioners~~ in fall prevention and the effects on healthcare practitioner performance and patient outcomes. The interventions were delivered by nurses, physicians, physiotherapists, pharmacists, occupational therapists, and paramedics. Regarding the effects on healthcare practitioner performance, CDS ~~interventions~~ likely~~may~~ improves ~~healthcare practitioners’ adherence to recommended practice regarding~~ fall risk assessments and the provision of recommended interventions ~~to prevent falls, but the evidence is very uncertain. CDS interventions~~ and may improve ~~healthcare practitioners’ adherence to recommended~~ medication review and prescribing. Regarding the effects on patient outcomes,~~Interventions using~~ CDS ~~to prevent falls among older adults~~ likely decreases the rate of falls in hospitals ~~or~~ and residential care settings and in patients aged 80 years or older.~~,~~ Furthermore, CDS may reduce fall rates in community-dwelling older adults and in ~~pateints~~patients aged 65 to 80 years, but the effects in these subgroups may be small~~, but they likely make little to no difference in fall rate among community-dwelling older adults. CDS interventions likely reduce the rate of falls among patients aged 80 years or older, but the certainty of the evidence on the effect on fall rate among adults aged between 65 and 80 years was very low~~. CDS interventions likely reduce fall risk slightly, and may reduce the rate of fall injuries in patients aged between 65 and 80 years. Finally, CDS may reduce fall injuries in adults aged 65 to 80 years, but the~~ir~~ effect on fall injuries in adults aged 80 years or older remains very uncertain.

Interventions

A common feature of all experimental interventions included in this review is that they delivered decision support to healthcare practitioners aiming to prevent falls in older adults. The control interventions were generally poorly described as consisting of usual care, with different interventions depending on the setting or the type of healthcare practitioners. Most of the experimental interventions utilised CDS to aid healthcare practitioners in conducting risk assessments and implementing preventive measures across multiple domains such as gait and balance problems, environmental factors, and medications, as opposed to focusing on a single domain. Multifactorial fall prevention interventions have been shown effective in reducing falls [60], and using CDS when delivering such complex interventions may offer healthcare practitioners structure and guidance [91]. NextHowever, it is important to note that, even though most of the interventions provided CDS electronically, only 36% of the CDS tools automatically delivered recommendations to the healthcare practitioners. A systematic review found that providing CDS automatically versus on demand may lead to large improvements in adherence [23]. Had more CDS tools been designed for automatic decision support it could potentially have increased healthcare practitioners' adherence to recommendations.

Healthcare practitioner performance

Our findings are consistent with previous systematic reviews reporting improvements in adherence to recommended practice [32,34] and medication outcomes [33]. Mebrahtu et al. [31] also found favourable effects in healthcare practitioner performance associated with the use of CDS, including improvements in nurses' adherence to hand disinfection guidance, insulin dosing, on-time blood sampling, and documentation of care. Kwan et al. 2020 [34]

reported that CDS ~~systems~~ increased the proportion of patients receiving recommended care. Yourman et al. ~~2008~~ [33] found that, in most cases, CDS improved medication outcomes. Although improvements were mostly moderate, these results demonstrate the diversity of clinical areas in which CDS ~~tools~~ may be beneficial.

Patient outcomes

Our meta-analyses indicated that CDS interventions may prevent falls among ~~21~~14 per 1,000 older adults during a follow-up period of nine months (with a baseline risk of ~~30~~28.7% and a 7% risk reduction) and may prevent 175 falls per 1,000 person-years (with a baseline rate of 672 falls per 1,000 person-years and a 26% fall rate reduction). The studies included in the meta-analysis on fall risk represent a range of age groups and care settings, suggesting that the findings are broadly applicable to the population of older adults at risk of falls. There may be several explanations for the finding that CDS interventions have seemingly different effects on the risk versus the rate of falls. Dautzenberg et al. ~~2021~~ [13] hypothesi~~z~~sed that fall rate may measure the risk of falls more accurately than the number of fallers. The outcome ‘fall risk’ counts the number of persons who fall, whether once or repeatedly, as a single outcome event. Therefore, repeated falls by the same person do not contribute additional events or data beyond the initial fall. In contrast, the outcome ‘rate of falls’ counts each individual fall as a separate outcome event. For example, a person who falls five times during the follow-up period would contribute five events to the ‘rate of falls’ outcome but only one event to the ‘fall risk’ outcome. Consequently, ‘fall risk’ does not capture changes in the frequency of falls among older adults. It may be that the interventions included in our study were more effective at reducing the frequency of falls among those with the highest fall risk, i.e., recurrent fallers, than among one-time fallers. Such a reduction in the frequency of falls

among recurrent fallers would result in a larger reduction in the rate of falls compared to fall risk. In agreement with our findings, earlier systematic reviews [13,92] reported that multifactorial interventions were associated with a reduction in fall rate but with a smaller reduction in fall risk.

Next, tThe studies included in the meta-analyses on fall rate encompass a range of age groups and care settings, suggesting that the findings are broadly applicable to the entire study population. tThere are several potential explanations for the difference in fall rate reduction between settings, i.e., a 26% fall rate reduction in hospitals and residential care vs.ersus a 3% fall rate reduction in community-dwelling older adults. Older adults in hospitals and care homes are at high risk of falls [16], and interventions may be more effective in high-risk patients than in low-risk patients [93,94]. Therefore, interventions directed towards at high-risk patients will show larger absolute effects than interventions directed towards at low- to moderate-risk patients. Furthermore, CDS tools may be more readily implemented in hospital and residential care settings than in primary care due to the complexity of the setting [95], potentially increasing healthcare practitioners' fidelity to the intervention protocol. Another possible explanation is differences in follow-up time in the included studies. The median follow-up time was 6 six months for studies conducted in hospitals and residential care, and 12 months for studies in community-dwelling older adults. The effect of fall prevention interventions may dwindle as time passes [96]. , This dwindling is presumably because of cessation by patients ceasing to participate in of the fall prevention programme in which they participated in during the study, such as physical exercise [16]. However, we opted not to conduct a subgroup analysis by follow-up time to minimise the risk of type I errors, which may have led to false positive results. Moreover, while our results indicate a 26% reduction in fall rate in hospitals orand residential care, the substantial heterogeneity (I^2 = 65%)

suggests that the observed effects likely vary across studies, potentially due to differences in study populations, settings, or intervention delivery. Despite this variability, CDS interventions targeting healthcare practitioners likely reduce fall rates in hospitals or residential care, though the effect size may depend on the context.

The certainty of the ~~results~~ effects on fall injuries is low for patients aged 65 to 80 years and very low for patients aged 80 years or older, due to risk of bias and imprecision. Given that the outcome measure is a rate ratio and not a risk ratio, the width of the CIs surrounding the point estimate is calculated based on the number of events and not the number of participants. Therefore, the wide CIs around the point estimates ~~on~~ for fall injury rate may be explained by the fact that fall injuries are somewhat rare~~,~~ when compared with falls [64]. Lastly, none of the studies included in this systematic review reported any negative effects attributable to the CDS interventions. Our findings are consistent with previous systematic reviews [31,33]. For instance, Yourman et al. ~~(2008)~~ found that, although CDS may cause errors in medication prescribing, in most cases, CDS helped to reduce side-effects and improve patient safety [33].

When implementing innovations into clinical practice, both high-income countries (HIC) and low- and middle-income countries (LMIC) face barriers related to political, social, and cultural factors, as well as resources~~,~~ and healthcare practitioner-related factors [97,98]. While the studies included in this review were conducted in HIC, LMIC face additional barriers, such as physical challenges like unreliable power supplies and poor internet connectivity, limited access to electronic health records and computers, and human resources constraints, including overburdened staff and lack of formal training for healthcare workers [98]. This limited digital infrastructure could hinder the integration of CDS tools, which often rely on access to electronic health records and digital systems [91]. To address these

challenges, CDS systems need to be adaptable to LMIC contexts, such as through offline functionality or paper-based versions. Additionally, CDS tools should be user-friendly to minimise the burden on already overworked staff.

A notable aspect of this study is the absence of established MID values for fall risk, fall rate, and fall injury rate. We assessed certainty based on whether the true effect lies on the observed side of the null, with the null effect used as the threshold. While some of the findings in this review suggest that the effects of CDS may be small, we did not determine what constitutes a clinically important effect, as this often depends on the context and setting.

What is considered an important effect (or MID), and therefore what is worth the effort of implementation, can vary significantly between settings. In HIC with robust healthcare infrastructure, even small reductions in fall risk or fall injuries may justify the use of CDS interventions. Conversely, in LMIC contexts, where resources such as computers, internet access, and trained personnel are limited, the threshold for what constitutes an important difference may be higher. In such settings, interventions with larger impacts may be prioriti~~z~~sed to promote the best use of limited resources. Context-specific thresholds could enable a more nuanced evaluation of treatment effects and help guide decisions on whether CDS interventions are worth the effort required for implementation.

Implications

Given that many of the included studies targeted participants with increased fall risk, e.g., previous fallers or those with specific risk factors, the findings of this review are likely most applicable to populations at a higher risk of falls. Our estimates indicate that, on average,

CDS interventions directed ~~towards~~ at healthcare practitioners may prevent ~~21~~14 older adults per 1,000 from falling, with a ~~30~~28.7% baseline fall risk. CDS interventions may also prevent 175 falls per 1,000 person-years in hospitals and residential care as well as prevent 188 falls per 1,000 person-years in adults aged 80 years and older, assuming a baseline fall rate of 672 falls per 1,000 person-years. However, further research is needed to better understand the significant differences in fall rate reduction between hospitals or residential care settings and community-dwelling older adults. Future studies should focus on identifying the factors that contribute to the ~~higher~~ greater effectiveness of interventions in hospitals and residential care and explore ways to adapt these strategies for community-dwelling older adults. This systematic review provides valuable insight that can be used when planning the use of CDS tools in fall prevention efforts among older adults. Directing interventions ~~towards~~ at high-risk patients may offer greater benefits than directing the interventions ~~towards~~ at low- to moderate-risk patients. However, ~~the~~ CDS tools should be ~~appropriately~~ designed to automatically provide decision support on-screen instead of paper-based decision support provided on demand [23]. Only 36% of the CDS interventions in this review met these criteria and herein lies a potential to further improve patient outcomes, adherence to recommended practice, and medication outcomes.

Strengths and limitations

To our knowledge, this is the first systematic review to provide an overview of the effects of CDS used by healthcare practitioners in fall prevention among older adults. A major strength ~~in~~ of this review is the thorough and sensitive literature search strategy. Potential non-reporting biases, poor indexing, and other factors make it impossible to know whether all relevant studies were in fact identified [43]. We therefore searched the reference lists of

included articles and relevant systematic reviews. Also, the funnel plots along with the Egger tests and trim-and-fill analyses suggested no evidence of publication bias for fall risk or fall rate. This indicates that we ~~may~~ have likely included most relevant studies. Furthermore, outcomes indicating healthcare practitioner performance were reported so varyingly among the studies that meta-analyses were not possible. We consider it a strength that we conducted vote-counting based on direction of effect instead of relying solely on textual descriptions of results [43]. Limitations of vote-counting include that the method provides no information on the magnitude of effects and does not account for differences in the relative sizes of the studies. Still, vote-counting enables a statistical analysis of whether there is any evidence of effect of the intervention. Also, this method may be superior to a narrative description, in which some results are privileged above others without appropriate justification [43]. ~~Lastly~~Moreover, we performed meta-analyses on fall outcomes and used the GRADE approach with explicit judgements of the certainty of the evidence [99].

A limitation of this review is that verification of the collected data by a second reviewer was performed for only five of 25 included studies, raising concerns about potential errors during data collection. However, the selection of each result from each study was discussed and verified with the project statistician to ensure correctness and consistency. Another limitation of this review is the uncertainty surrounding the specific effects of different CDS tools. For example, while some tools delivered decision support to healthcare practitioners ~~while~~ conducting multifactorial risk assessments and interventions [40,86], other tools focused solely on guided medication dosing [41,87]. This variation in tool types and use cases makes it difficult to assess what effects can be expected from different tools or whether certain tools have any measurable impact at all. Furthermore, a limitation of the studies included in this review is that most results were judged to be at high or serious risk of bias. All the results

from the included non-randomised studies were at risk of bias due to confounding. The lack of randomisation increases the risk of unequal distribution of confounding factors between intervention groups, as group assignment may be influenced by knowledge of prognostic factors. Moreover, none of the included non-randomised studies employed analysis methods that adequately controlled for all important confounders, such as age, a history of falls, sex, or gait and balance impairments. The direction and magnitude of this confounding, however, is not known. This makes it difficult to judge whether the effect estimates ~~is~~are greater or lower than the true effect. For the randomised studies, the main concerns for risk of bias were related to deviations from intended interventions, such as crossover effects in Peterson et al. [87] and intervention contamination in Clemson et al. [71].

Conclusions

CDS ~~tools~~ likely~~may~~ improves the performance of healthcare practitioners in fall prevention for older adults. CDS ~~interventions~~ probably reduce falls and may reduce fall injury rates, but the effects can vary across different subgroups of older adults. This systematic review contributes valuable understanding of the effects of CDS directed ~~towards~~ at healthcare practitioners in the implementation of fall prevention among older adults. Prioritising resources and directing interventions ~~towards~~ at high-risk patients may offer the greatest effects in terms of preventing falls, but the interventions should be long-lasting and ~~the~~ CDS tools should be designed ~~appropriately~~ to improve adherence to recommended practice. Future research focusing on fall injuries should strive to improve precision by increasing the number of participants and the length of follow-up. In addition, meta-analyses of healthcare practitioner performance outcomes would become possible with more uniform and detailed reporting of results. Finally, the design of ~~the~~ CDS tools may positively or negatively affect

healthcare practitioners' adherence to recommended practice, however, additional research is needed to identify which specific design elements of CDS tools contribute to successful outcomes in fall prevention.

Acknowledgements

The authors are grateful to help provided by Elisabeth Karlsen and Kari Kalland (OsloMet) for electronic database searches.

References

1. Elias Filho J, Borel WP, Diz JBM, Barbosa AWC, Britto RR, Felício DC. Prevalence of falls and associated factors in community-dwelling older Brazilians: a systematic review and meta-analysis. Cad Saude Publica. 2019;35: 1–16. doi:10.1590/0102-311X00115718
2. James SL, Lucchesi LR, Bisignano C, Castle CD, Dingels Z V, Fox JT, et al. The global burden of falls: global, regional and national estimates of morbidity and mortality from the Global Burden of Disease Study 2017. Injury Prevention. 2020;26: i3–i11. doi:10.1136/injuryprev-2019-043286
3. Haagsma JA, Olij BF, Majdan M, van Beeck EF, Vos T, Castle CD, et al. Falls in older aged adults in 22 European countries: incidence, mortality and burden of disease from 1990 to 2017. Injury Prevention. 2020;26: i67–i74. doi:10.1136/injuryprev-2019-043347
4. Bergland A, Pettersen AM, Laake K. Falls reported among elderly Norwegians living at home. Physiotherapy Research International. 1998;3: 164–174. doi:10.1002/pri.138
5. Hektoen LF, Saltvedt I, Sletvold O, Helbostad JL, Lurås H, Halsteinli V. One-year health and care costs after hip fracture for home-dwelling elderly patients in Norway: Results from the Trondheim Hip Fracture Trial. Scand J Public Health. 2016;44: 791–798. doi:10.1177/1403494816674162
6. Davis JC, Robertson MC, Ashe MC, Liu-Ambrose T, Khan KM, Marra CA. International comparison of cost of falls in older adults living in the community: a systematic review. Osteoporosis International. 2010;21: 1295–1306. doi:10.1007/s00198-009-1162-0
7. Florence CS, Bergen G, Atherly A, Burns E, Stevens J, Drake C. Medical Costs of Fatal and Nonfatal Falls in Older Adults. J Am Geriatr Soc. 2018;66: 693–698. doi:10.1111/jgs.15304

8. Heinrich S, Rapp K, Rissmann U, Becker C, König H-H. Cost of falls in old age: a systematic review. Osteoporosis International. 2010;21: 891–902. doi:10.1007/s00198-009-1100-1
9. Li Y, Hou L, Zhao H, Xie R, Yi Y, Ding X. Risk factors for falls among community-dwelling older adults: A systematic review and meta-analysis. Front Med (Lausanne). 2023;9: 1–13. doi:10.3389/fmed.2022.1019094
10. Shao L, Shi Y, Xie X-Y, Wang Z, Wang Z-A, Zhang J-E. Incidence and Risk Factors of Falls Among Older People in Nursing Homes: Systematic Review and Meta-Analysis. J Am Med Dir Assoc. 2023;24: 1708–1717. doi:10.1016/j.jamda.2023.06.002
11. Gillespie LD, Robertson MC, Gillespie WJ, Sherrington C, Gates S, Clemson LM, et al. Interventions for preventing falls in older people living in the community. Cochrane Database of Systematic Reviews. 2012;2012: 1–366. doi:10.1002/14651858.CD007146.pub3
12. Olij BF, Ophuis RH, Polinder S, van Beeck EF, Burdorf A, Panneman MJM, et al. Economic Evaluations of Falls Prevention Programs for Older Adults: A Systematic Review. J Am Geriatr Soc. 2018;66: 2197–2204. doi:10.1111/jgs.15578
13. Dautzenberg L, Beglinger S, Tsokani S, Zevgiti S, Raijmann RCMA, Rodondi N, et al. Interventions for preventing falls and fall-related fractures in community-dwelling older adults: A systematic review and network meta-analysis. J Am Geriatr Soc. 2021;69: 2973–2984. doi:10.1111/jgs.17375
14. Meekes WM, Korevaar JC, Leemrijse CJ, van de Goor IA. Practical and validated tool to assess falls risk in the primary care setting: a systematic review. BMJ Open. 2021;11: 1–10. doi:10.1136/bmjopen-2020-045431
15. Stevens JA, Ballesteros MF, Mack KA, Rudd RA, DeCaro E, Adler G. Gender Differences in Seeking Care for Falls in the Aged Medicare Population. Am J Prev Med. 2012;43: 59–62. doi:10.1016/j.amepre.2012.03.008
16. Montero-Odasso M, van der Velde N, Martin FC, Petrovic M, Tan MP, Ryg J, et al. World guidelines for falls prevention and management for older adults: a global initiative. Age Ageing. 2022;51: 1–36. doi:10.1093/ageing/afac205
17. Markle-Reid MF, Dykeman CS, Reimer HD, Boratto LJ, Goodall CE, McGugan JL. Engaging community organizations in falls prevention for older adults: Moving from research to action. Can J Public Health. 2015;106: e189–e196. doi:10.17269/cjph.106.4776
18. Olij BF, Erasmus V, Kuiper JI, van Zoest F, van Beeck EF, Polinder S. Falls prevention activities among community-dwelling elderly in the Netherlands: A Delphi study. Injury. 2017;48: 2017–2021. doi:10.1016/j.injury.2017.06.022
19. Leysens G, Vlaeyen E, Vanaken D, Janssens E, Dejaeger E, Cambier D, et al. Het toepassen van valpreventiemaatregelen bij thuiswonende ouderen: een survey onderzoek in Vlaanderen [The use of fall prevention strategies in home care: a survey in Flanders]. Tijdschr Gerontol Geriatr. 2017;48: 121–133. doi:10.1007/s12439-017-0215-7
20. Moreland B, Kakara R, Henry A. Trends in Nonfatal Falls and Fall-Related Injuries Among Adults Aged ≥65 Years — United States, 2012–2018. MMWR Morb Mortal Wkly Rep. 2020;69: 875–881. doi:10.15585/mmwr.mm6927a5
21. Shaver AL, Clark CM, Hejna M, Feuerstein S, Wahler RG, Jacobs DM. Trends in fall-related mortality and fall risk increasing drugs among older individuals in the United States,1999–2017. Pharmacoepidemiol Drug Saf. 2021;30: 1049–1056. doi:10.1002/pds.5201

22. Fretheim A, Flottorp S, Oxman A. Effekt av tiltak for implementering av kliniske retningslinjer. Rapport fra Kunnskapssenteret nr. 10-2015. Oversikt over systematiske oversikter [Effect of interventions for implementing clinical practice guidelines]. Oslo; 2015.

23. Van de Velde S, Heselmans A, Delvaux N, Brandt L, Marco-Ruiz L, Spitaels D, et al. A systematic review of trials evaluating success factors of interventions with computerised clinical decision support. Implementation Science. 2018;13: 1–11. doi:10.1186/s13012-018-0790-1

24. Wasylewicz ATM, Scheepers-Hoeks AMJW. Clinical Decision Support Systems. Fundamentals of Clinical Data Science. Cham: Springer International Publishing; 2019. pp. 153–169. doi:10.1007/978-3-319-99713-1_11

25. Blalock SJ, Ferreri SP, Renfro CP, Robinson JM, Farley JF, Ray N, et al. Impact of STEADI-Rx : A Community Pharmacy-Based Fall Prevention Intervention. J Am Geriatr Soc. 2020;68: 1778–1786. doi:10.1111/jgs.16459

26. Dykes PC, Carroll DL, Hurley A, Lipsitz S, Benoit A, Chang F, et al. Fall prevention in acute care hospitals: a randomized trial. JAMA. 2010;304: 1912–1918. doi:10.1001/jama.2010.1567

27. Carroll DL, Dykes PC, Hurley AC. An Electronic Fall Prevention Toolkit: Effect on Documentation Quality. Nurs Res. 2012;61: 309–313. doi:10.1097/NNR.0b013e31825569de

28. Dykes PC, Burns Z, Adelman J, Benneyan J, Bogaisky M, Carter E, et al. Evaluation of a Patient-Centered Fall-Prevention Tool Kit to Reduce Falls and Injuries. JAMA Netw Open. 2020;3: e2025889. doi:10.1001/jamanetworkopen.2020.25889

29. Aizen E, Lutsyk G, Wainer L, Carmeli S. Effectiveness of individualized fall prevention program in geriatric rehabilitation hospital setting: a cluster randomized trial. Aging Clin Exp Res. 2015;27: 681–688. doi:10.1007/s40520-015-0330-7

30. Barker AL, Morello RT, Wolfe R, Brand CA, Haines TP, Hill KD, et al. 6-PACK programme to decrease fall injuries in acute hospitals: cluster randomised controlled trial. BMJ. 2016; h6781. doi:10.1136/bmj.h6781

31. Mebrahtu TF, Skyrme S, Randell R, Keenan A-M, Bloor K, Yang H, et al. Effects of computerised clinical decision support systems (CDSS) on nursing and allied health professional performance and patient outcomes: a systematic review of experimental and observational studies. BMJ Open. 2021;11: e053886. doi:10.1136/bmjopen-2021-053886

32. Damoiseaux-Volman BA, van der Velde N, Ruige SG, Romijn JA, Abu-Hanna A, Medlock S. Effect of Interventions With a Clinical Decision Support System for Hospitalized Older Patients: Systematic Review Mapping Implementation and Design Factors. JMIR Med Inform. 2021;9: 1–11. doi:10.2196/28023

33. Yourman L, Concato J, Agostini J V. Use of computer decision support interventions to improve medication prescribing in older adults: A systematic review. Am J Geriatr Pharmacother. 2008;6: 119–129. doi:10.1016/j.amjopharm.2008.06.001

34. Kwan JL, Lo L, Ferguson J, Goldberg H, Diaz-Martinez JP, Tomlinson G, et al. Computerised clinical decision support systems and absolute improvements in care: meta-analysis of controlled clinical trials. BMJ. 2020;370: 1–11. doi:10.1136/bmj.m3216

35. Moja L, Kwag KH, Lytras T, Bertizzolo L, Brandt L, Pecoraro V, et al. Effectiveness of Computerized Decision Support Systems Linked to Electronic Health Records: A Systematic Review and Meta-Analysis. Am J Public Health. 2014;104: e12–e22. doi:10.2105/AJPH.2014.302164

36. Garg AX, Adhikari NKJ, McDonald H, Rosas-Arellano MP, Devereaux PJ, Beyene J, et al. Effects of Computerized Clinical Decision Support Systems on Practitioner Performance and Patient Outcomes. JAMA. 2005;293: 1223–1238. doi:10.1001/jama.293.10.1223
37. Jaspers MWM, Smeulers M, Vermeulen H, Peute LW. Effects of clinical decision-support systems on practitioner performance and patient outcomes: a synthesis of high-quality systematic review findings. Journal of the American Medical Informatics Association. 2011;18: 327–334. doi:10.1136/amiajnl-2011-000094
38. Taheri Moghadam S, Sadoughi F, Velayati F, Ehsanzadeh SJ, Poursharif S. The effects of clinical decision support system for prescribing medication on patient outcomes and physician practice performance: a systematic review and meta-analysis. BMC Med Inform Decis Mak. 2021;21: 1–26. doi:10.1186/s12911-020-01376-8
39. Shahmoradi L, Safdari R, Ahmadi H, Zahmatkeshan M. Clinical decision support systems-based interventions to improve medication outcomes: A systematic literature review on features and effects. Med J Islam Repub Iran. 2021;35: 1–16. doi:10.47176/mjiri.35.27
40. Elley CR, Robertson MC, Garrett S, Kerse NM, McKinlay E, Lawton B, et al. Effectiveness of a Falls-and-Fracture Nurse Coordinator to Reduce Falls: A Randomized, Controlled Trial of At-Risk Older Adults. J Am Geriatr Soc. 2008;56: 1383–1389. doi:10.1111/j.1532-5415.2008.01802.x
41. Tamblyn R, Eguale T, Buckeridge DL, Huang A, Hanley J, Reidel K, et al. The effectiveness of a new generation of computerized drug alerts in reducing the risk of injury from drug side effects: a cluster randomized trial. Journal of the American Medical Informatics Association. 2012;19: 635–643. doi:10.1136/amiajnl-2011-000609
42. Shamseer L, Moher D, Clarke M, Ghersi D, Liberati A, Petticrew M, et al. Preferred reporting items for systematic review and meta-analysis protocols (PRISMA-P) 2015: elaboration and explanation. BMJ. 2015;349: g7647. doi:10.1136/bmj.g7647
43. Higgins J, Thomas J, Chandler J, Cumpston M, Li T, Page M, et al. Cochrane Handbook for Systematic Reviews of Interventions version 6.4 (updated August 2023). 6.4. Cochrane; 2023. Available: www.training.cochrane.org/handbook
44. EPOC resources for review authors. [cited 22 Nov 2020]. Available: https://epoc.cochrane.org/resources/epoc-resources-review-authors
45. Page MJ, McKenzie JE, Bossuyt PM, Boutron I, Hoffmann TC, Mulrow CD, et al. The PRISMA 2020 statement: an updated guideline for reporting systematic reviews. BMJ. 2021;372: n71. doi:10.1136/bmj.n71
46. Covidence Systematic Review Tool. In: Covidence [Internet]. [cited 17 May 2025]. Available: https://www.covidence.org/
47. Griese-Mammen N, Hersberger KE, Messerli M, Leikola S, Horvat N, van Mil JWF, et al. PCNE definition of medication review: reaching agreement. Int J Clin Pharm. 2018;40: 1199–1208. doi:10.1007/s11096-018-0696-7
48. El-Khoury F, Cassou B, Charles M-A, Dargent-Molina P. The effect of fall prevention exercise programmes on fall induced injuries in community dwelling older adults: systematic review and meta-analysis of randomised controlled trials. BMJ. 2013;347: f6234. doi:10.1136/bmj.f6234
49. Seppala LJ, van de Glind EMM, Daams JG, Ploegmakers KJ, de Vries M, Wermelink AMAT, et al. Fall-Risk-Increasing Drugs: A Systematic Review and Meta-analysis: III. Others. J Am Med Dir Assoc. 2018;19: 372.e1-372.e8. doi:10.1016/j.jamda.2017.12.099

50. Hoffmann TC, Glasziou PP, Boutron I, Milne R, Perera R, Moher D, et al. Better reporting of interventions: template for intervention description and replication (TIDieR) checklist and guide. BMJ. 2014;348: g1687–g1687. doi:10.1136/bmj.g1687
51. Lewin S, Hendry M, Chandler J, Oxman AD, Michie S, Shepperd S, et al. Assessing the complexity of interventions within systematic reviews: development, content and use of a new tool (iCAT_SR). BMC Med Res Methodol. 2017;17: 76. doi:10.1186/s12874-017-0349-x
52. Lamb SE, Becker C, Gillespie LD, Smith JL, Finnegan S, Potter R, et al. Reporting of complex interventions in clinical trials: development of a taxonomy to classify and describe fall-prevention interventions. Trials. 2011;12: 125. doi:10.1186/1745-6215-12-125
53. Van de Velde S, Kunnamo I, Roshanov P, Kortteisto T, Aertgeerts B, Vandvik PO, et al. The GUIDES checklist: development of a tool to improve the successful use of guideline-based computerised clinical decision support. Implementation Science. 2018;13: 86. doi:10.1186/s13012-018-0772-3
54. Medlock S, Wyatt JC, Patel VL, Shortliffe EH, Abu-Hanna A. Modeling information flows in clinical decision support: key insights for enhancing system effectiveness. Journal of the American Medical Informatics Association. 2016;23: 1001–1006. doi:10.1093/jamia/ocv177
55. Sterne JAC, Savović J, Page MJ, Elbers RG, Blencowe NS, Boutron I, et al. RoB 2: a revised tool for assessing risk of bias in randomised trials. BMJ. 2019;366: l4898. doi:10.1136/bmj.l4898
56. Sterne JA, Hernán MA, Reeves BC, Savović J, Berkman ND, Viswanathan M, et al. ROBINS-I: a tool for assessing risk of bias in non-randomised studies of interventions. BMJ. 2016;355: i4919. doi:10.1136/bmj.i4919
57. Sterne J, Hernán M, McAleenan A, Reeves B, Higgins J. Chapter 25: Assessing risk of bias in a non-randomized study. In: Higgins JPT, Thomas J, Chandler J, Cumpston M, Li T, Page MJ, Welch VA (editors). Cochrane Handbook for Systematic Reviews of Interventions version 6.4 (updated August 2023). Cochrane; 2023. Available: http://www.training.cochrane.org/handbook
58. Campbell M, McKenzie JE, Sowden A, Katikireddi SV, Brennan SE, Ellis S, et al. Synthesis without meta-analysis (SWiM) in systematic reviews: reporting guideline. BMJ. 2020;368: l6890. doi:10.1136/bmj.l6890
59. McKenzie J, Brennan S. Chapter 12: Synthesizing and presenting findings using other methods. In: Higgins JPT, Thomas J, Chandler J, Cumpston M, Li T, Page MJ, Welch VA (editors). Cochrane Handbook for Systematic Reviews of Interventions version 6.4 (updated August 2023). Cochrane; 2023. Available: http://www.training.cochrane.org/handbook
60. Tricco AC, Thomas SM, Veroniki AA, Hamid JS, Cogo E, Strifler L, et al. Comparisons of Interventions for Preventing Falls in Older Adults: A Systematic Review and Meta-analysis. JAMA. 2017;318: 1687–1699. doi:10.1001/jama.2017.15006
61. Deeks JJ, Higgins JP, Altman DG. Chapter 10: Analysing data and undertaking meta-analyses. In: Higgins JPT, Thomas J, Chandler J, Cumpston M, Li T, Page MJ, Welch VA (editors). Cochrane Handbook for Systematic Reviews of Interventions version 6.4 (updated August 2023). Cochrane; 2023. Available: www.training.cochrane.org/handbook
62. StataCorp. Stata Statistical Software: Release 18. College Station, TX. 2023.
63. Higgins J, Li T, Deeks J. Chapter 6: Choosing effect measures and computing estimates of effect. In: Higgins JPT, Thomas J, Chandler J, Cumpston M, Li T, Page

MJ, Welch VA (editors). Cochrane Handbook for Systematic Reviews of Interventions version 6.4 (updated August 2023). Cochrane; 2023. Available: http://www.training.cochrane.org/handbook

64. Bergen G, Stevens MR, Burns ER. Falls and Fall Injuries Among Adults Aged ≥65 Years — United States, 2014. MMWR Morb Mortal Wkly Rep. 2016;65: 993–998. doi:10.15585/mmwr.mm6537a2
65. Guyatt G, Zeng L, Brignardello-Petersen R, Prasad M, De Beer H, Murad MH, et al. Core GRADE 2: choosing the target of certainty rating and assessing imprecision. BMJ. 2025;389: e081904. doi:10.1136/bmj-2024-081904
66. Egger M, Smith GD, Schneider M, Minder C. Bias in meta-analysis detected by a simple, graphical test. BMJ. 1997;315: 629–634. doi:10.1136/bmj.315.7109.629
67. Hultcrantz M, Rind D, Akl EA, Treweek S, Mustafa RA, Iorio A, et al. The GRADE Working Group clarifies the construct of certainty of evidence. J Clin Epidemiol. 2017;87: 4–13. doi:10.1016/j.jclinepi.2017.05.006
68. Zeng L, Brignardello-Petersen R, Hultcrantz M, Mustafa RA, Murad MH, Iorio A, et al. GRADE Guidance 34: update on rating imprecision using a minimally contextualized approach. J Clin Epidemiol. 2022;150: 216–224. doi:10.1016/j.jclinepi.2022.07.014
69. Santesso N, Glenton C, Dahm P, Garner P, Akl EA, Alper B, et al. GRADE guidelines 26: informative statements to communicate the findings of systematic reviews of interventions. J Clin Epidemiol. 2020;119: 126–135. doi:10.1016/j.jclinepi.2019.10.014
70. Guyatt G, Yao L, Murad MH, Hultcrantz M, Agoritsas T, De Beer H, et al. Core GRADE 6: presenting the evidence in summary of findings tables. BMJ. 2025;389: e083866. doi:10.1136/bmj-2024-083866
71. Clemson L, Mackenzie L, Lovarini M, Roberts C, Poulos R, Sherrington C, et al. Integrated solutions for sustainable fall prevention in primary care: a pragmatic hybrid-type 2 mixed methods implementation and effectiveness study. Front Public Health. 2024;12. doi:10.3389/fpubh.2024.1446525
72. Phelan EA, Williamson BD, Balderson BH, Cook AJ, Piccorelli A V., Fujii MM, et al. Reducing Central Nervous System–Active Medications to Prevent Falls and Injuries Among Older Adults. JAMA Netw Open. 2024;7: e2424234. doi:10.1001/jamanetworkopen.2024.24234
73. Bhasin S, Gill TM, Reuben DB, Latham NK, Ganz DA, Greene EJ, et al. A Randomized Trial of a Multifactorial Strategy to Prevent Serious Fall Injuries. New England Journal of Medicine. 2020;383: 129–140. doi:10.1056/NEJMoa2002183
74. Blum MR, Sallevelt BTGM, Spinewine A, O'Mahony D, Moutzouri E, Feller M, et al. Optimizing Therapy to Prevent Avoidable Hospital Admissions in Multimorbid Older Adults (OPERAM): cluster randomised controlled trial. BMJ. 2021;374: n1585. doi:10.1136/bmj.n1585
75. Byrne CM. Impact of prospective computerized clinical decision support information and targeted assistance on nursing home resident outcomes. University of Albany, State University of New York. 2005. Available: https://www.proquest.com/openview/59a276fe7e8913563fcd2bb9b487d19d/1?pq-origsite=gscholar&cbl=18750&diss=y
76. Ferrer A, Formiga F, Sanz H, de Vries OJ, Pujol R, Badia T, et al. Multifactorial assessment and targeted intervention to reduce falls among the oldest-old: a randomized controlled trial. Clin Interv Aging. 2014;9: 383–393. doi:10.2147/CIA.S57580

77. Frankenthal D, Lerman Y, Kalendaryev E, Lerman Y. Intervention with the Screening Tool of Older Persons Potentially Inappropriate Prescriptions/Screening Tool to Alert Doctors to Right Treatment Criteria in Elderly Residents of a Chronic Geriatric Facility: A Randomized Clinical Trial. J Am Geriatr Soc. 2014;62: 1658–1665. doi:10.1111/jgs.12993
78. Gallagher PF, O'Connor MN, O'Mahony D. Prevention of Potentially Inappropriate Prescribing for Elderly Patients: A Randomized Controlled Trial Using STOPP/START Criteria. Clin Pharmacol Ther. 2011;89: 845–854. doi:10.1038/clpt.2011.44
79. Ganz DA, Kim S-B, Zingmond DS, Ramirez KD, Roth CP, Jennings LA, et al. Effect of a Falls Quality Improvement Program on Serious Fall-Related Injuries. J Am Geriatr Soc. 2015;63: 63–70. doi:10.1111/jgs.13154
80. Wenger NS, Roth CP, Hall WJ, Ganz DA, Snow V, Byrkit J, et al. Practice Redesign to Improve Care for Falls and Urinary Incontinence. Arch Intern Med. 2010;170: 1765–1772. doi:10.1001/archinternmed.2010.387
81. Ganz DA, Yuan AH, Greene EJ, Latham NK, Araujo K, Siu AL, et al. Effect of the STRIDE fall injury prevention intervention on falls, fall injuries, and health-related quality of life. J Am Geriatr Soc. 2022;70: 3221–3229. doi:10.1111/jgs.17964
82. Groshaus H, Boscan A, Khandwala F, Holroyd-Leduc J. Use of Clinical Decision Support to Improve the Quality of Care Provided to Older Hospitalized Patients. Appl Clin Inform. 2012;03: 94–102. doi:10.4338/ACI-2011-08-RA-0047
83. Healey F, Monro A, Cockram A, Adams V, Heseltine D. Using targeted risk factor reduction to prevent falls in older in-patients: a randomised controlled trial. Age Ageing. 2004;33: 390–395. doi:10.1093/ageing/afh130
84. Lightbody E. Evaluation of a nurse-led falls prevention programme versus usual care: a randomized controlled trial. Age Ageing. 2002;31: 203–210. doi:10.1093/ageing/31.3.203
85. Mahoney JE, Shea TA, Przybelski R, Jaros L, Gangnon R, Cech S, et al. Kenosha County Falls Prevention Study: A Randomized, Controlled Trial of an Intermediate-Intensity, Community-Based Multifactorial Falls Intervention. J Am Geriatr Soc. 2007;55: 489–498. doi:10.1111/j.1532-5415.2007.01144.x
86. Logan PA, Horne JC, Gladman JRF, Gordon AL, Sach T, Clark A, et al. Multifactorial falls prevention programme compared with usual care in UK care homes for older people: multicentre cluster randomised controlled trial with economic evaluation. BMJ. 2021;375: e066991. doi:10.1136/bmj-2021-066991
87. Peterson JF, Rosenbaum BP, Waitman LR, Habermann R, Powers J, Harrell D, et al. Physicians' response to guided geriatric dosing: initial results from a randomized trial. Stud Health Technol Inform. 2007;129: 1037–1040. Available: http://www.ncbi.nlm.nih.gov/pubmed/17911873
88. Snooks HA, Carter B, Dale J, Foster T, Humphreys I, Logan PA, et al. Support and Assessment for Fall Emergency Referrals (SAFER 1): Cluster Randomised Trial of Computerised Clinical Decision Support for Paramedics. Quinn TJ, editor. PLoS One. 2014;9: e106436. doi:10.1371/journal.pone.0106436
89. Weber V, White A, McIlvried R. An Electronic Medical Record (EMR)-Based Intervention to Reduce Polypharmacy and Falls in an Ambulatory Rural Elderly Population. J Gen Intern Med. 2008;23: 399–404. doi:10.1007/s11606-007-0482-z
90. Wenger NS, Roth CP, Shekelle PG, Young RT, Solomon DH, Kamberg CJ, et al. A Practice-Based Intervention to Improve Primary Care for Falls, Urinary Incontinence, and Dementia. J Am Geriatr Soc. 2009;57: 547–555. doi:10.1111/j.1532-5415.2008.02128.x

91. Sutton RT, Pincock D, Baumgart DC, Sadowski DC, Fedorak RN, Kroeker KI. An overview of clinical decision support systems: benefits, risks, and strategies for success. NPJ Digit Med. 2020;3: 17. doi:10.1038/s41746-020-0221-y
92. Hopewell S, Adedire O, Copsey BJ, Boniface GJ, Sherrington C, Clemson L, et al. Multifactorial and multiple component interventions for preventing falls in older people living in the community. Cochrane Database of Systematic Reviews. 2018;7: CD012221. doi:10.1002/14651858.CD012221.pub2
93. Wang H, Boissel J-P, Nony P. Revisiting the relationship between baseline risk and risk under treatment. Emerg Themes Epidemiol. 2009;6: 1. doi:10.1186/1742-7622-6-1
94. Ho KM. Importance of the baseline risk in determining sample size and power of a randomized controlled trial. Journal of Emergency and Critical Care Medicine. 2018;2. doi:10.21037/jeccm.2018.08.06
95. Goodwin V, Jones-Hughes T, Thompson-Coon J, Boddy K, Stein K. Implementing the evidence for preventing falls among community-dwelling older people: A systematic review. J Safety Res. 2011;42: 443–451. doi:10.1016/j.jsr.2011.07.008
96. Finnegan S, Seers K, Bruce J. Long-term follow-up of exercise interventions aimed at preventing falls in older people living in the community: a systematic review and meta-analysis. Physiotherapy. 2019;105: 187–199. doi:10.1016/j.physio.2018.09.002
97. Wang T, Tan J-Y (Benjamin), Liu X-L, Zhao I. Barriers and enablers to implementing clinical practice guidelines in primary care: an overview of systematic reviews. BMJ Open. 2023;13: e062158. doi:10.1136/bmjopen-2022-062158
98. Leonard E, de Kock I, Bam W. Barriers and facilitators to implementing evidence-based health innovations in low- and middle-income countries: A systematic literature review. Eval Program Plann. 2020;82: 101832. doi:10.1016/j.evalprogplan.2020.101832
99. Schünemann H, Brożek J, Guyatt G, Oxman A. GRADE Handbook: Introduction to GRADE Handbook. 2013. Available: https://gdt.gradepro.org/app/handbook/handbook.html

Supporting information

S1 Table. PRISMA checklist.docx

S2 Table. Differences between protocol and review.docx

S3 Table. Electronic searches.docx

S4 Table. Excluded studies.xlsx

S5Table. Description of interventions and funding sources.docx

S6Table. Design factors of CDS.docx

S7 Table. Risk of bias.pdf

S8 Table. Individual study results.docx

S9 Table. GRADE evidence profile.docx

S1 Appendix. Data used for analyses of healthcare practitioner performance outcomes.xlsx

S2 Appendix. Data used for meta-analyses.xlsx

S3 Appendix. Extracted data.csv

S1 Fig. Funnell plot of comparison: CDS interventions vs control on fall risk.

S2 Fig. Trim-and-fill analysis of comparison: CDS interventions vs control on fall risk.

S3 Fig. Meta-analysis comparing CDS interventions with control on fall rate, subgroup. analysis by patients’ age.

S4 Fig. Funnel plot of comparison: CDS interventions vs control on fall rate.

S5 Fig. Trim-and-fill analysis of comparison: CDS interventions vs control on fall rate.

S6 Fig. Meta-analysis comparing CDS interventions with control on fall rate, subgroup analysis by risk of bias.

S7 Fig. Meta-analysis comparing CDS interventions with control on fall injury rate, subgroup analysis by risk of bias.

S8 Fig. Meta-analysis comparing CDS interventions with control on fall injury rate, subgroup analysis by study setting.

Response to Reviewers

Dear Dr. Nishant Premnath Jaiswal

Thank you for the opportunity to submit a revised draft of our manuscript titled "Effectiveness of clinical decision support in fall prevention among older adults: a systematic review and meta-analysis" to PLOS ONE. We greatly appreciate the time and effort you and the reviewers have dedicated to providing thoughtful and constructive feedback. We have carefully considered all comments and made revisions to address the suggestions provided.

All authors have reviewed and approved the submission of the revised manuscript. The manuscript has not been published and is not being considered for publication elsewhere, in whole or in part, in any language. We hope you will now be able to accept the article for publication in your journal.

Thank you for your consideration.

Yours sincerely
Rune Solli on behalf of the authors

Response to Reviewer 2
Comment 1:
This manuscript addresses an important and timely topic: the effectiveness of Clinical Decision Support (CDS) systems in fall prevention among older adults. The systematic review and meta-analysis are well designed, and the authors follow rigorous methods aligned with PRISMA and GRADE guidelines. However, several aspects require major attention before the manuscript can be considered for publication... Language and Grammar (Essential Revision): The manuscript still contains numerous grammatical and syntactical issues that reduce clarity and weaken its impact. A professional English language editing is essential, especially in the Introduction and Discussion sections. For example:

Line 93: "Falls can cause significant health loss that leads to more complex care requirements" → awkward phrasing.

Line 92: "Falls are a major cause of morbidity and mortality…" → avoid redundancy by rephrasing the following lines more concisely.
Response 1:
Thank you for this comment. In response to your feedback, the manuscript has undergone professional English language editing by a native English speaker. We have carefully revised

the text, particularly the Introduction and Discussion sections, to improve clarity and address the issues you identified.

Comment 2:
GRADE Interpretation (Overstatement):
The authors frequently describe outcomes with low or very low certainty as "may improve" or "likely reduce." While this aligns with GRADE semantics, the consistent use of optimistic phrasing throughout the abstract and conclusion may mislead readers about the strength of evidence. I strongly recommend a more cautious tone, especially for:

Medication outcomes: classified as "low certainty" but still interpreted positively.

Fall injury in those 65–80 years: RaR 0.80 with CI crossing 1.00 should be clearly described as statistically non-significant.
<u>Response 2:</u>
Thank you for your comment on the interpretation of outcomes with low or very low certainty. We acknowledge that the repeated use of phrases such as "may improve" or "likely reduce" throughout the abstract and conclusion could potentially convey an overly optimistic impression. To address this, we have carefully reviewed the text and revised it ensure a more cautious and neutral tone, particularly for outcomes with low or very low certainty, to align with your suggestion and avoid the risk of misleading readers. Please see the updated text. We have explicitly described the result on fall injuries in those aged 65 to 80 years as statistically non-significant. Please see page 29, lines 556-557.

Comment 3:
Methodological Transparency:

The authors claim no language restriction, but earlier versions included a list of included languages. Ensure this inconsistency is corrected in both the Methods and Appendices.

Risk of bias assessments are now available in S7 Table, but summary RoB graphs in the main text are difficult to interpret (small font, unclear legends). Please improve readability and ensure consistency between main text and supplements.
<u>Response 3:</u>
Thank you for your comment regarding language restrictions. No language restrictions were imposed on the literature searches. In response to a previous Editor request, we removed the list of languages to avoid the implication that language restrictions had been applied. We have reviewed the methods section and Appendices to ensure consistency throughout. Additionally, we have revised the summary RoB graphs to improve readability, including increasing font sizes and clarifying legends, and to ensure consistency between the main text and supplements.

Comment 4:
Data Completeness (Minor):

The explanation for handling missing data is too brief. Please detail the imputation procedures used and specify how often author contact led to data retrieval.
Response 4
Thank you for your comment. We have expanded the Methods section to clarify that we contacted the corresponding authors of eight reports to obtain missing data, with up to three email attempts per author. Data retrieval was successful in four instances, and no data imputation was performed. Please see page 10, lines 208-213.

Comment 5:
Figures and Tables:

Figure readability is still suboptimal despite the revisions. Font sizes and labeling in forest plots and risk-of-bias visuals should be increased for clarity, especially for print versions.
Response 5:
Thank you for your comment. We have revised the figures to improve their readability in both the main text and supplementary materials, with particular attention to ensuring clarity in print versions. Font sizes and labelling in the forest plots and risk-of-bias visuals have been increased for enhanced clarity.

Comment 6:
Minor Points:

Use consistent terminology: sometimes "CDS systems" is used redundantly (the "S" already stands for "system").

Add a visual summary table of GRADE certainty per outcome (possibly alongside S9 Table) in the main manuscript for quick reference.

Discuss potential implementation barriers to CDS in LMIC settings – currently the review is biased toward high-income contexts.
Response 6:
Thank you for your observation regarding terminology. For clarity, in this manuscript, we have defined “CDS” as “clinical decision support” and have carefully reviewed the text to ensure consistent use and avoid any potential confusion.

We have added a GRADE summary of findings table in the manuscript for quick reference. Please see Table 4.

Thank you for highlighting the important point about implementation in LMIC. We acknowledge that the review predominantly focuses on high-income contexts. To address this, we have added a discussion of potential implementation barriers in LMIC, including limited

digital infrastructure, restricted access to electronic health records, overburdened staff, and other challenges. Please see page 35, lines 713-724.

Response to Reviewer 3

Comment 1:

Pg. 5 ln 118 - Suggest changing to "Generally, the use of CDS by healthcare ...."
Pg 5 ln 121 - suggest changing to "patients [32], medication outcomes in older adults [33,34], and adults in general.
Pg 6 ln 134 - suggest changing to "This systematic review aimed to evaluate the effects of CDS for...."
Pg 8 ln 172 - since your inclusion criteria included restrictions on the type of studies for inclusion in the review, please clarify why you did not include restrictions on the study design within your search strategy?
Pg 10 ln 208 - Why did you include studies which did not specify one of your required outcomes?
Pg 14 ln 301 - what do you mean by the phrase "to a median (min-max) of 1 433 (312-46245) patient participants?
Pg 24 ln 460 - your interpretation of the impact of CDS on falls in hospital/residential care, although was significant, needs to include the considerable heterogenity present.
Pg 24 ln 466-478 - your interpretation of the sub group analysis I feel is inaccurate and likely do not reduce the rate of falls.
Pg 28 ln 572-574 - your results do not reflect that CDS does not reduce falls in pts >80
Pg 33 - Strengths and limitations section - please expand more on limitations of the review

<u>Response 1:</u>

Pg. 5 ln 118 - Thank you for the suggestion. We have updated the sentence to: "Generally, the use of CDS by healthcare ..." for improved clarity.
Pg 5 ln 121 - We have revised the text accordingly.
Pg 6 ln 134 - Thank you for your suggestion. We have revised the sentence to: “This systematic review aimed to evaluate the effects of CDS for fall prevention on …”
Pg 8 ln 172 – Thank you for this observation. Our inclusion criteria restricted study types during the screening and selection phases to ensure relevance and rigor. However, we did not restrict study design in the search strategy to maximise sensitivity, as publications may not always disclose the study design.
Pg 10 ln 208 - Thank you for your questions. Studies that did not specify one of our required outcomes were excluded. We have revised Table 1 in the Methods section to clarify this decision and enhance transparency.
Pg 14 ln 301 - This phrase indicates the median number of patient participants included across the studies, with the smallest study having 312 participants and the largest having 46,245. To improve clarity, have revised this sentence to: "… to a median number of 1,433 patient participants, ranging from 312 to 46,245."
Pg 24 ln 460 – Thank you for pointing this out. We have added a sentence in the Results section to acknowledge the substantial heterogeneity and revised the Discussion section to address the considerable heterogeneity and its implications for interpretating the effects of CDS on falls in hospital/residential care settings.

Pg 24 ln 466-478 – Thank you for pointing this out. Considering comments from all reviewers, we have updated the grading of certainty to explicitly reflect the certainty in the direction of effect. Minor adjustments were made in the certainty ratings, and the corresponding statements about the results have been revised for accuracy and clarity. For further details, please see our response to comment 5 from reviewer 4.
Pg 28 ln 572-574 - Thank you for your comment. Our results reflect that CDS interventions reduce the rate of falls among patients aged 80 years or older. In accordance with GRADE methodology, we have used the term "likely" to describe findings supported by moderate certainty evidence. We have retained this wording and clarified this choice in the methods section under "Assessment of certainty of the evidence". Please see page 14, lines 312-313 and Table 4.
Pg 33 - Strengths and limitations section - Thank you for the suggestion. We have expanded the Strengths and limitations section to include additional limitations, such as the uncertainty surrounding the specific effects of different types of CDS tools and the substantial risk of bias in most of the included studies.

Response to Reviewer 4

Comment 1

Thank you for the opportunity to review this revised manuscript. I did not review the original version but have examined the responses to the previous reviewer comments and have reviewed the revised version anew from my perspective.
I did not verify that all requests by the Academic Editor were addressed, but notice that the author did not add any information into the manuscript, as stated in their response, about any methods for author contact for missing outcome data. If they did perform author contact, the means (eg email first author) and frequency should be stated.

Response 1

Thank you for your comment. We have updated the Methods section to include details about author contact for missing outcome data. Specifically, we clarified that corresponding authors were contacted via email, with up to three follow-up attempts if necessary. Please see page 10, lines 208-211.

Comment 2

Abstract: i) please note the search date(s), ii) if space permits it would be excellent to provide a brief overview of the types of CDS interventions used most, iii) please see below for some suggestions and questions about the GRADE approach which may influence the reporting of results in the abstract
Introduction: stating that "interventions to prevent falls" are cost-effective and effective in falls and fall injuries could be more specific especially since not all interventions have been shown successful and the authors are arguing for a review of CDS interventions. Possibly "a variety of interventions aimed at older adults" or such would help.

Response 2

Thank you for these suggestions. We have added the search dates to the Methods section of the abstract, included a brief overview of the most used types of CDS, and reviewed the reporting of results in the abstract considering your suggestions regarding the GRADE

approach. Please see the revised abstract. In the introduction, we have specified the types of fall prevention interventions that are cost-effective and effective in reducing falls and fall injuries. Please see pages 4-5, lines 103-107.

Comment 3:

Materials and methods:

1. Was the protocol only ever "drafted"? even if there were changes post hoc the protocol should have been in some form of final version before starting the review.

2. Spell out PRISMA at first use

3. Selection process: please add details about who conducted full text review, and confirm whether consensus was required at title/abstract stage or just full text.

4. Data collection process: using verification for only 5 of 24 papers should be noted in the discussion as a limitation of the review (possibility for errors). Duplicate extraction or verification of all data is a standard for systematic reviews. This is particularly concerning if only one person chose which outcome data to use when studies reported on multiple outcomes.

5. Study risk of bias: please confirm if "the main outcome results" means the result data used for each outcome of interest to the review, or otherwise.

6. Synthesis methods: please add a section on unit of analysis issues, i.e. what was done to account for effects of clustering in cluster RCTs? Was the authors' result adjusted for clustering used for analysis and, if not reported/performed, what was done?

Response 3

1. Thank you for your comment. The protocol was drafted, finalised, and registered in PROSPERO before commencing the review. We have clarified this in the Methods section. Please see page 7, lines 152-156.

2. We have spelled out PRISMA at its first mention.

3. Thank you for your comment. We have added details about who conducted full-text review and clarified that consensus was required at both the title/abstract screening stage and the full-text screening stage. Please see page 9, lines 197-201.

4. Thank you for highlighting this issue. We have discussed the limited verification of collected data by a second reviewer as a limitation of the review in the Discussion section to acknowledge the possibility of errors. Additionally, we have clarified that the selection of each result was discussed and verified with the project statistician to ensure correctness and consistency. Please see page 38, lines 784-787.

5. We have specified which outcomes were assessed for risk of bias. Please see page 12, lines 253-254.

6. Thank you for your comment regarding unit of analysis issues. The cluster-randomised trials included in the review accounted for clustering in their original analyses. Additionally, in our syntheses, we ensured that only one outcome from each study was included, thus maintaining independence between studies in the analyses. We have included a sentence in the methods section to clarify this. Please see page 13, lines 291-292.

Comment 4

Results/conclusions:
1. it would be of interest to comment on how many studies had eligibility criteria related to increased risk for falls (e.g. previous fallers, 1+ risk factor etc), and to state whether the findings overall are most applicable to populations at increased risk (this may vary by outcome to some degree),
2. it is hard to know whether the findings for the fall risk outcome may be most applicable to certain settings or ages; can the authors comment on whether the 10 studies appeared to capture both settings and ages fairly well? For example, if the large majority of the participants in the analysis came from studies undertaken in residential/acute care there could be some concerns about directness to the entire population of interest (eg rating down for indirectness may apply) or some way to clarify this observation for the reader,
3. likewise with setting and age groups for fall rates is there any indication that the studies in residential/acute care also enrolled older people to help know whether findings could be most applicable to both setting and age combined?,
4. conversion of ORs to absolute risk difference (for falls risk) should first convert the OR to a RR (see appendix 3 in https://www.bmj.com/content/389/bmj-2024-081904),
5. the authors should speak to their reason for use of a separate study (ref 88) for control event rates (rather than the studies themselves) and describe this study in some detail,
6. in figure 2 there are some "FU" and "follow-up" so this could be made consistent; further the authors should be able to put something into the figure for the medication outcome findings for the Blalock study, even if just a narrative statement by the authors about the direction of effect,
7. for mortality and hospitalizations the reversion to relying on statistical significance goes against what was done for other outcomes; perhaps the authors comment about any directions of effect, speak to the small sample sizes for these rare outcomes and point again to where folks can find the results if they want.
Line 561-2: would suggest deleting this sentence since the authors did not assess the magnitude of effects in their review
<u>Response 4:</u>
1. Thank you for your comment. We have added details about how many studies included eligibility criteria related to increased fall risk in the results section. Please see pages 14-15, lines 337-339. Additionally, we have stated in the discussion section that the findings of this review are likely most applicable to populations at higher fall risk. Please see page 36, lines 743-745.
2. We have added details in the results section regarding the number of studies reporting fall risk that focused on different settings and age groups. These details suggest that the findings are largely applicable to the entire population of interest. Please see page 24, lines 446-449. Additionally, we have commented on the applicability of the findings from the meta-analysis on fall risk in the Discussion section. Please see page 33, lines 657-659.
3. Thank you for this comment. We have added details in the results section regarding the number of studies that included patients from different age groups within the hospitals or residential care subgroup. Please see pages 25-26, lines 488-492. Similarly, we have added details regarding the number of studies conducted in each setting within the subgroup by patients' age. Please see page 27 lines 508-512. Additionally, we have commented on the

applicability of the findings from the meta-analyses on fall rate in the Discussion section. Please see page 34, lines 677-679.
4. Thank you for your comment. Before calculating the absolute fall risk in the intervention group and the absolute risk difference, we have converted the ORs (including the point estimate and lower and upper limits of the confidence interval) to RRs using the formula provided in appendix 3 of https://www.bmj.com/content/389/bmj-2024-081904. This process has been described in the methods section for clarity. Please see page 13, lines 295-297.
5. Thank you for your comment regarding the use of a separate study for control event rates. Due to the limited reporting of absolute numbers in the studies included in this review, we derived control intervention risks and rates from a report describing the epidemiology of falls among older adults. This approach aligns with Cochrane methodology [1]. We have clarified this in the methods section and provided a description of the study used for control event risks and rates. Please see page 13, lines 293-295. Additionally, we updated the control fall risk from 30% to 28.7%, as reported in the study. Please see Table 4.
6. In figure 2, we have replaced all instances of 'follow-up' with 'FU' for consistency and added a description of the medication outcome results for the Blalock study.
7. We acknowledge your comment about the reliance on statistical significance and have revised the text to place greater emphasis on the direction of effect, the small sample size, and the rarity of the outcomes. Additionally, we have informed the reader where individual study results can be found for further details.
Line 561-2: Thank you for your feedback regarding the sentence: "Had more CDS tools been designed for automatic decision support it could potentially have increased healthcare practitioners' adherence to recommendations." We have removed the sentence as suggested.

Comment 5

Major comment:

GRADE does not assess the certainty of the point estimate per se but it's relation to the "target" of certainty which should be defined in the methods section (see GRADE 34 guidance and J Clin Epidemiol. 2017 Jul:87:4-13. doi: 10.1016/j.jclinepi.2017.05.006 and BMJ. 2025 Apr 29:389:e081904. doi: 10.1136/bmj-2024-081904). For the provider outcomes, the target would need to be a direction of effect only since the analysis did not assess magnitude. For the patient outcomes, this could have been the null (direction of effect) or some form of a minimally important difference (even if approximate), especially to help determine whether findings are "little to no difference" or "an affect". It appears the authors just used direction of effect and if so this should be mentioned and the findings should be stated in this light. It is confusing what the authors have done for this since there is no statement in the methods and for falls risk (OR 0.93, 21 fewer fallers per 1000) they state little to no effect in the results (implying 21 fewer is below some threshold of an MID) but a slight reduction in the abstract and discussion.

The choice of the target can change the GRADE ratings for all domains but in particular inconsistency and imprecision. For the provider outcomes, rating a conclusion of direction of effect where all studies showed the same direction would suggest against rating down for inconsistency; the size of effect doesn't matter for this target. With some of the effects not being statistically significant/precise for the direction there could be concern over imprecision

but it may not be serious. For direction of effect for adherence, I would think low certainty overall seems appropriate with the text indicating rating down twice for ROB. The same applies for medication outcomes, unless the authors want to have a third category of No direction which a couple of the findings indicate (e.g. Blum & Lightbody) and where some inconsistency in direction could be noted. If the authors really think low certainty for the direction is appropriate (vs moderate with just serious ROB) they could consider rating down twice for ROB or possibly once for ROB and once for imprecision (as above) or inconsistency (noting really that a direction was not shown in a couple of cases). For the falls risk outcome, if the authors are rating certainty in direction of effect, then not rating down for imprecision makes sense (assuming they wouldn't be too strict on the upper limit of the 95% CI crossing the null slightly) and they would conclude there is an effect (possibly mentioning that it may not be important), but if they want to say there is "little to no" difference (implying use of an MID and that the point estimate is below this) then they likely should state what the MID was and make sure the entire 95% CI does not contain this value or else also rate down for imprecision. For the falls rate outcomes, if using a direction of effect the authors should note this in their conclusions whereas if they think the magnitude is at least as large as some small but important effect they may also rate down for either inconsistency (since ~30% of the weight in the analysis showed effects below what might be considered an MID) or for imprecision if the lower limit of the 95%CI (81 fewer falls) does not surpass their MID.
In summary, having a clear statement about what the authors were rating their certainty in (e.g. null/direction of effect) would be good as well as re-considering their assessments for the provider outcomes in light of this (not rating down for inconsistency). If using the direction of effect their falls risk findings should likely be "an effect", with a comment that the effects may be small. If MIDs were applied these should be stated with the above considerations added.
Response 5
Thank you for your detailed feedback regarding the use of GRADE to assess the certainty of evidence in this study. We appreciate your suggestions for clarifying our approach.

We graded the certainty of evidence based on the direction of effect rather than the MID. This decision was informed by the lack of established MID values for the outcomes of interest (fall risk, fall rate, and fall injury rate). Despite extensive literature searches conducted with the support of two librarians at OsloMet University, we were unable to identify absolute MID values for these outcomes, except for a single relative fall rate value specific to Parkinson's patients (25%). Given the lack of absolute MID values applicable to fall risk, fall rate, and fall injury rate, we determined that assessing the certainty of evidence for the direction of effect was the most appropriate approach.

In response to your feedback, we have revised the methods section to explicitly state that we assessed certainty in the direction of effect, using the null effect as the threshold. We also re-evaluated our GRADE ratings to reflect this approach. For provider outcomes, we did not rate down for inconsistency where all studies showed the same direction of effect, as suggested. For the fall risk outcome, we rated down for imprecision because of the slight crossing of the null by the upper limit of the CI.

To ensure consistency in reporting, we revised the results and discussion sections, as well as the abstract, to frame findings in terms of the direction of effect. For example, we now describe the findings for fall risk as indicating “an effect,” while noting that the effects may be small. Additionally, we added a summary table of GRADE certainty for each outcome (Table 4) to facilitate quick reference. Finally, we added a note in the discussion to acknowledge the lack of established MID values and highlighted the need for future work to establish such thresholds for fall prevention outcomes. Please see page 36, lines 726-740.

References

1. Schünemann HJ, Higgins JPT, Vist GE, Glasziou P, Akl EA, Skoetz N, et al. Chapter 14: Completing ‘Summary of findings’ tables and grading the certainty of the evidence. 6.4. Higgins JPT, Thomas J, Chandler J, Cumpston M, Li T, Page MJ, Welch VA (editors) Cochrane Handbook for Systematic Reviews of Interventions version 64 (updated August 2023) Cochrane, 2023 Available from www.training.cochrane.org/handbook. 6.4. Cochrane; 2023.
