# Supplementary material for: Effectiveness of clinical decision support in fall prevention among older adults: A systematic review and meta-analysis
Source: PLoS One. 2026 Jan 12;21(1):e0340025. doi: 10.1371/journal.pone.0340025 (PMC12795367; doi:10.1371/journal.pone.0340025)
Supplement: Attachment — Submitted filename: Response to Reviewers.docx [file pone.0340025.s021.docx]

**Response to Reviewers**

Dear Dr. Nishant Premnath Jaiswal

Thank you for giving us the opportunity to submit a revised draft of our manuscript titled "Effectiveness of clinical decision support in fall prevention among older adults: a systematic review and meta-analysis" to PLOS ONE. We appreciate the time and effort you and the reviewers have invested in providing valuable feedback on our manuscript. We have carefully considered the comments and have revised the manuscript to address the suggestions provided.

All authors have reviewed and approved the submission of the revised manuscript. The manuscript has not been published and is not being considered for publication elsewhere, in whole or in part, in any language. We hope you will now be able to accept the article for publication in your journal.

Thank you for your consideration.

Yours sincerely

Rune Solli on behalf of the authors

**Response to the Academic Editor**

**Comment 1:**

*Please ensure that your manuscript meets PLOS ONE's style requirements, including those for file naming.*

Response 1:

Thank you for your feedback. We have made changes to the manuscript to meet PLOS ONE’s style requirements, including the following:

- We corrected an error on the title page; please refer to page 1, line 10 for details. We have also updated affiliation 5 and removed affiliation 6 for a co-author. Affiliation 6 was removed because this co-author is no longer associated with that institution. Please see page 1, lines 5, 15, and 16.
- Headings:
  - We used level 1 heading for all major sections of the manuscript, level 2 heading for sub-sections of major sections, and level 3 heading for sub-sections within level 2 headings.
  - We used bold type, sentence case, and the correct font size for the different heading levels.
- Figures and tables:
  - We made corrections to the table and figure titles and legends.
  - We reformatted Table 4 to a figure, more specifically to Figure 2, because it contains graphics.
  - We renamed Fig 2 as Fig 3, Fig 3 as Fig 4, and Fig 4 as Fig 5.
  - We reformatted the manuscript’s figure files as .tif files.
  - We renamed Figure file names to Fig1, Fig2, Fig3, etc.
  - We changed the resolution of the manuscript Figs to 300 dpi.
  - We moved the tables and figures to appear directly after the paragraph where they are first cited.
- Supporting information:
  - Please see our response to Comment 3 for details on the changes made to the supporting information.
- References:
  - We changed reference style to comply with PLOS ONE’s guidelines. For more information on the changes made to the reference list, please refer to our response to Comment 4.

**Comment 2:**

*We note that you have contradictory statements about the literature search in your manuscript. Please can you remove the list of languages the studies reported are in as this suggests that a language restriction was imposed.*

Response 2:

Thank you for pointing this out. We have removed the list of languages the studies are reported in. Please see page 7, lines 154-155.

**Comment 3:**

*As required by our policy on Data Availability, please ensure your manuscript or supplementary information includes the following:*

*A numbered table of all studies identified in the literature search, including those that were excluded from the analyses.*

*For every excluded study, the table should list the reason(s) for exclusion.*

*If any of the included studies are unpublished, include a link (URL) to the primary source or detailed information about how the content can be accessed.*

*A table of all data extracted from the primary research sources for the systematic review and/or meta-analysis. The table must include the following information for each study:*

*Name of data extractors and date of data extraction*

*Confirmation that the study was eligible to be included in the review.*

*All data extracted from each study for the reported systematic review and/or meta-analysis that would be needed to replicate your analyses.*

*If data or supporting information were obtained from another source (e.g. correspondence with the author of the original research article), please provide the source of data and dates on which the data/information were obtained by your research group.*

*If applicable for your analysis, a table showing the completed risk of bias and quality/certainty assessments for each study or outcome. Please ensure this is provided for each domain or parameter assessed. For example, if you used the Cochrane risk-of-bias tool for randomized trials, provide answers to each of the signalling questions for each study. If you used GRADE to assess certainty of evidence, provide judgements about each of the quality of evidence factor. This should be provided for each outcome.*

*An explanation of how missing data were handled.*

*This information can be included in the main text, supplementary information, or relevant data repository. Please note that providing these underlying data is a requirement for publication in this journal, and if these data are not provided your manuscript might be rejected.*

Response 3:

Thank you for the reminder. We have updated the manuscript to include the required information regarding *PLOS ONE's* policy on Data Availability. We have now included a numbered table listing all studies identified in the literature search, including those excluded from the analysis and the reason for their exclusion. Please see S4 table. One of the included studies, Byrne 2005, is a PhD thesis, and we have now provided a URL link to this source. We have now also included an appendix (S3 Appendix), showing data extracted from the primary research sources, including the name of the data extractors and the date of data extraction, and confirmation that the study was eligible to be included in the review. All the data needed to replicate our analyses were obtained from publicly available primary sources, including journal articles and clinical trials registries. These data are available in S1 and S2 appendices. We have now included the completed risk of bias assessments for each study, along with the answers to the signalling questions, available in S7 Table. We have included a table showing the certainty assessments for each of each outcome, available in S9 table. We explained how missing data were handled, including seeking information from original study authors and imputing missing data. For further details, please see page 12, lines 264-267.

**Comment 4:**

*Please review your reference list to ensure that it is complete and correct. If you have cited papers that have been retracted, please include the rationale for doing so in the manuscript text, or remove these references and replace them with relevant current references. Any changes to the reference list should be mentioned in the rebuttal letter that accompanies your revised manuscript. If you need to cite a retracted article, indicate the article’s retracted status in the References list and also include a citation and full reference for the retraction notice.*

Response 4:

Thank you for pointing this out. We have updated the references in line with PLOS ONE's guidelines. We have reviewed the references with regards to volume number, page numbers, and article identifiers. We removed Montero-Odasso et al. 2021, titled ‘New horizons in falls prevention and management for older adults: a global initiative,’ from the list of included studies, as it was mistakenly included and did not meet our inclusion criteria. When reviewing the references we identified two duplicate references and these were removed (Tamblyn et al. 2012 and Elley et al. 2008). Additionally, we identified that we had referenced multiple versions of the Cochrane handbook. Therefore, we removed references to Cochrane Handbook version 6.3 from 2022 and earlier versions, and we now cite the correct version that we used, version 6.4 from 2023. We did not cite any retracted articles.

**Response to Reviewer 1**

**Comment 1:**

*Abstract:*

*Please clarify the statement that the effect on medications was uncertain. Methods did not describe a search for effects on any medication. Page 10 of the manuscript describes monitoring use of "fall-risk increasing drugs”. It may help to mention this in the abstract so readers know what type of medications were measured/reported.*

*The statement, “CDS interventions may reduce fall injury rate in older adults aged between 65 and 80 years (RaR 0.80; 95% CI 0.59, 1.09)” does not seem accurate because the confidence interval crosses the 1.0 value. Please consider restating this*

*If I understand the data correctly, it may be more accurate to change the last sentence of the Abstract to: “but the evidence on fall injury rate in community-dwelling patients aged 80 years or older was very uncertain.” In other words, CDS use worked well in hospitals and residential care, but the effect was not statistically significant in community dwelling patients. This is such an important observation that it needs to be clear here and in the Conclusion of the Abstract on Page 3.*

Response 1:

Thank you for pointing this out. We have now added details of the primary outcomes of the systematic review in the methods section of the abstract, including medication outcomes, which consisted of medication review and prescribing. Please see page 2, lines 42-44. In the results section of the abstract, we replaced the statement, “however the effect on medication outcomes was very uncertain,” with “…and medication review and prescribing (all nine comparisons favouring CDS; 95% CI 70%, 100%; low certainty).” Please see page 2, lines 51-53.

Regarding the statement, “*CDS interventions may reduce fall injury rate in older adults aged between 65 and 80 years (RaR 0.80; 95% CI 0.59, 1.09),*” GRADE guidance suggests that findings should be communicated based on the point estimate and the certainty of evidence [1]. Our best estimate indicates a 20% reduction in fall injury rate, which, together with the low certainty of evidence for this outcome, supports our decision to keep the original phrasing. We hope this explains our choice. If the reviewer still feels this interpretation is incorrect, we are open to reconsider and making necessary changes. We have now clarified in the methods section that results are described in line with GRADE guidance, including a brief explanation of the approach, along with the Santesso reference [1]. Please see page 13, lines 279-281. We have now also included the certainty of evidence rating when reporting the results in the results section of the abstract. Please see pages 2-3, lines 49-61.

Thank you for highlighting the results of CDS interventions among community-dwelling older adults and the uncertainty regarding to fall injuries. We appreciate your insight and have revised the conclusion section of the abstract to emphasize the finding that CDS interventions may reduce falls in hospitals and residential care settings, but likely not in community-dwelling older adults. We have also emphasized the uncertainty of CDS interventions’ effects on fall injury rates in adults aged 80 years or older. Please see page 3, lines 63-70.

**Comment 2:**

*Manuscript (MS)*

*Please check grammar throughout the MS. For example, Page 4, Introduction line 2 “leading” should be “leads”. This is an important, well-done study, potentially affecting important clinical and health economic outcomes. Grammatical errors distract from its credibility and potential clinical use.*

*I do not have time to address every grammatical error. This needs review by a good editor.*

Response 2:

Thank you for your feedback. We have corrected the sentence in the second line of the introduction; please see page 4, lines 92-93. Additionally, we have reviewed the manuscript and addressed the grammatical errors we identified.

**Response to Reviewer 2**

**Comment 1:**

*This systematic review and meta-analysis is an important contribution to the literature on fall prevention in older adults using CDS interventions.*

*The methodology is rigorous, and the conclusions are generally well-supported by the evidence.*

*Strengths include the comprehensive search, transparent methods, proper use of meta-analytic techniques, subgroup analyses, and the application of GRADE.*

*Minor points for improvement:*

*Discussion clarity: Some explanations regarding the difference between fall rate and fall risk outcomes could be streamlined to enhance reader understanding.*

*Graphical presentation: Figures could be slightly improved to enhance readability (e.g., legends and risk of bias charts could be made larger for clarity).*

*No ethical concerns were identified.*

*No concerns regarding plagiarism, redundant publication, or data fabrication were noted.*

*Recommendation:*

*Minor revision (language polishing in Discussion and minor graphical improvements).*

Response 1:

Thank you for your positive feedback on our systematic review and meta-analysis. We appreciate your recognition of our rigorous methodology. We have revised the manuscript to better explain the differences between fall risk and fall rate outcomes. Specifically, we have clarified the difference between fall risk and fall rate in the methods section; please see page 9, lines 197-203. We have also clarified how interventions might have a different impact on the rate of falls versus fall risk; please see pages 30-31, lines 612-626. We have enlarged the legends and the risk of bias charts to enhance readability. Please see Figs 2-5.

**References**

1.	Santesso N, Glenton C, Dahm P, Garner P, Akl EA, Alper B, et al. GRADE guidelines 26: informative statements to communicate the findings of systematic reviews of interventions. J Clin Epidemiol. 2020;119: 126–135. doi:10.1016/j.jclinepi.2019.10.014
