# Supplementary material for: Effectiveness of clinical decision support in fall prevention among older adults: A systematic review and meta-analysis
Source: PLoS One. 2026 Jan 12;21(1):e0340025. doi: 10.1371/journal.pone.0340025 (PMC12795367; doi:10.1371/journal.pone.0340025)
Supplement: Attachment — Submitted filename: Response_to_Reviewers_auresp_2.docx [file pone.0340025.s022.docx]

**Response to Reviewers**

Dear Dr. Nishant Premnath Jaiswal

Thank you for the opportunity to submit a revised draft of our manuscript titled "Effectiveness of clinical decision support in fall prevention among older adults: a systematic review and meta-analysis" to PLOS ONE. We greatly appreciate the time and effort you and the reviewers have dedicated to providing thoughtful and constructive feedback. We have carefully considered all comments and made revisions to address the suggestions provided.

All authors have reviewed and approved the submission of the revised manuscript. The manuscript has not been published and is not being considered for publication elsewhere, in whole or in part, in any language. We hope you will now be able to accept the article for publication in your journal.

Thank you for your consideration.

Yours sincerely

Rune Solli on behalf of the authors

**Response to Reviewer 2**

**Comment 1:**

*This manuscript addresses an important and timely topic: the effectiveness of Clinical Decision Support (CDS) systems in fall prevention among older adults. The systematic review and meta-analysis are well designed, and the authors follow rigorous methods aligned with PRISMA and GRADE guidelines. However, several aspects require major attention before the manuscript can be considered for publication... Language and Grammar (Essential Revision):
The manuscript still contains numerous grammatical and syntactical issues that reduce clarity and weaken its impact. A professional English language editing is essential, especially in the Introduction and Discussion sections. For example:

Line 93: “Falls can cause significant health loss that leads to more complex care requirements” → awkward phrasing.

Line 92: “Falls are a major cause of morbidity and mortality…” → avoid redundancy by rephrasing the following lines more concisely.*

Response 1:

Thank you for this comment. In response to your feedback, the manuscript has undergone professional English language editing by a native English speaker. We have carefully revised the text, particularly the Introduction and Discussion sections, to improve clarity and address the issues you identified.

**Comment 2:**

*GRADE Interpretation (Overstatement):
The authors frequently describe outcomes with low or very low certainty as "may improve" or "likely reduce." While this aligns with GRADE semantics, the consistent use of optimistic phrasing throughout the abstract and conclusion may mislead readers about the strength of evidence. I strongly recommend a more cautious tone, especially for:

Medication outcomes: classified as “low certainty” but still interpreted positively.

Fall injury in those 65–80 years: RaR 0.80 with CI crossing 1.00 should be clearly described as statistically non-significant.*

Response 2:

Thank you for your comment on the interpretation of outcomes with low or very low certainty. We acknowledge that the repeated use of phrases such as “may improve” or “likely reduce” throughout the abstract and conclusion could potentially convey an overly optimistic impression. To address this, we have carefully reviewed the text and revised it ensure a more cautious and neutral tone, particularly for outcomes with low or very low certainty, to align with your suggestion and avoid the risk of misleading readers. Please see the updated text. We have explicitly described the result on fall injuries in those aged 65 to 80 years as statistically non-significant. Please see page 29, lines 556-557.

**Comment 3:**

*Methodological Transparency:

The authors claim no language restriction, but earlier versions included a list of included languages. Ensure this inconsistency is corrected in both the Methods and Appendices.

Risk of bias assessments are now available in S7 Table, but summary RoB graphs in the main text are difficult to interpret (small font, unclear legends). Please improve readability and ensure consistency between main text and supplements.*

Response 3:

Thank you for your comment regarding language restrictions. No language restrictions were imposed on the literature searches. In response to a previous Editor request, we removed the list of languages to avoid the implication that language restrictions had been applied. We have reviewed the methods section and Appendices to ensure consistency throughout. Additionally, we have revised the summary RoB graphs to improve readability, including increasing font sizes and clarifying legends, and to ensure consistency between the main text and supplements.

**Comment 4:**

*Data Completeness (Minor):

The explanation for handling missing data is too brief. Please detail the imputation procedures used and specify how often author contact led to data retrieval.*

Response 4

Thank you for your comment. We have expanded the Methods section to clarify that we contacted the corresponding authors of eight reports to obtain missing data, with up to three email attempts per author. Data retrieval was successful in four instances, and no data imputation was performed. Please see page 10, lines 208-213.

**Comment 5:**

*Figures and Tables:

Figure readability is still suboptimal despite the revisions. Font sizes and labeling in forest plots and risk-of-bias visuals should be increased for clarity, especially for print versions.*

Response 5:

Thank you for your comment. We have revised the figures to improve their readability in both the main text and supplementary materials, with particular attention to ensuring clarity in print versions. Font sizes and labelling in the forest plots and risk-of-bias visuals have been increased for enhanced clarity.

**Comment 6:**

*Minor Points:

Use consistent terminology: sometimes "CDS systems" is used redundantly (the "S" already stands for "system").

Add a visual summary table of GRADE certainty per outcome (possibly alongside S9 Table) in the main manuscript for quick reference.

Discuss potential implementation barriers to CDS in LMIC settings – currently the review is biased toward high-income contexts.*

Response 6:

Thank you for your observation regarding terminology. For clarity, in this manuscript, we have defined “CDS” as “clinical decision support” and have carefully reviewed the text to ensure consistent use and avoid any potential confusion.

We have added a GRADE summary of findings table in the manuscript for quick reference. Please see Table 4.

Thank you for highlighting the important point about implementation in LMIC. We acknowledge that the review predominantly focuses on high-income contexts. To address this, we have added a discussion of potential implementation barriers in LMIC, including limited digital infrastructure, restricted access to electronic health records, overburdened staff, and other challenges. Please see page 35, lines 713-724.

**Response to Reviewer 3**

**Comment 1:**

*Pg. 5 ln 118 - Suggest changing to "Generally, the use of CDS by healthcare ...."
Pg 5 ln 121 - suggest changing to "patients [32], medication outcomes in older adults [33,34], and adults in general.
Pg 6 ln 134 - suggest changing to "This systematic review aimed to evaluate the effects of CDS for...."
Pg 8 ln 172 - since your inclusion criteria included restrictions on the type of studies for inclusion in the review, please clarify why you did not include restrictions on the study design within your search strategy?
Pg 10 ln 208 - Why did you include studies which did not specify one of your required outcomes?
Pg 14 ln 301 - what do you mean by the phrase "to a median (min-max) of 1 433 (312-46245) patient participants?
Pg 24 ln 460 - your interpretation of the impact of CDS on falls in hospital/residential care, although was significant, needs to include the considerable heterogenity present.
Pg 24 ln 466-478 - your interpretation of the sub group analysis I feel is inaccurate and likely do not reduce the rate of falls.
Pg 28 ln 572-574 - your results do not reflect that CDS does not reduce falls in pts >80
Pg 33 - Strengths and limitations section - please expand more on limitations of the review*

Response 1:

Pg. 5 ln 118 - Thank you for the suggestion. We have updated the sentence to: "Generally, the use of CDS by healthcare ..." for improved clarity.

Pg 5 ln 121 - We have revised the text accordingly.

Pg 6 ln 134 - Thank you for your suggestion. We have revised the sentence to: “This systematic review aimed to evaluate the effects of CDS for fall prevention on …”

Pg 8 ln 172 – Thank you for this observation. Our inclusion criteria restricted study types during the screening and selection phases to ensure relevance and rigor. However, we did not restrict study design in the search strategy to maximise sensitivity, as publications may not always disclose the study design.

Pg 10 ln 208 - Thank you for your questions. Studies that did not specify one of our required outcomes were excluded. We have revised Table 1 in the Methods section to clarify this decision and enhance transparency.

Pg 14 ln 301 - This phrase indicates the median number of patient participants included across the studies, with the smallest study having 312 participants and the largest having 46,245. To improve clarity, have revised this sentence to: "… to a median number of 1,433 patient participants, ranging from 312 to 46,245."

Pg 24 ln 460 – Thank you for pointing this out. We have added a sentence in the Results section to acknowledge the substantial heterogeneity and revised the Discussion section to address the considerable heterogeneity and its implications for interpretating the effects of CDS on falls in hospital/residential care settings.

Pg 24 ln 466-478 – Thank you for pointing this out. Considering comments from all reviewers, we have updated the grading of certainty to explicitly reflect the certainty in the direction of effect. Minor adjustments were made in the certainty ratings, and the corresponding statements about the results have been revised for accuracy and clarity. For further details, please see our response to comment 5 from reviewer 4.

Pg 28 ln 572-574 - Thank you for your comment. Our results reflect that CDS interventions reduce the rate of falls among patients aged 80 years or older. In accordance with GRADE methodology, we have used the term “likely” to describe findings supported by moderate certainty evidence. We have retained this wording and clarified this choice in the methods section under “Assessment of certainty of the evidence”. Please see page 14, lines 312-313 and Table 4.

Pg 33 - Strengths and limitations section - Thank you for the suggestion. We have expanded the Strengths and limitations section to include additional limitations, such as the uncertainty surrounding the specific effects of different types of CDS tools and the substantial risk of bias in most of the included studies.

**Response to Reviewer 4**

**Comment 1**

*Thank you for the opportunity to review this revised manuscript. I did not review the original version but have examined the responses to the previous reviewer comments and have reviewed the revised version anew from my perspective.
I did not verify that all requests by the Academic Editor were addressed, but notice that the author did not add any information into the manuscript, as stated in their response, about any methods for author contact for missing outcome data. If they did perform author contact, the means (eg email first author) and frequency should be stated.*

Response 1

Thank you for your comment. We have updated the Methods section to include details about author contact for missing outcome data. Specifically, we clarified that corresponding authors were contacted via email, with up to three follow-up attempts if necessary. Please see page 10, lines 208-211.

**Comment 2**

*Abstract: i) please note the search date(s), ii) if space permits it would be excellent to provide a brief overview of the types of CDS interventions used most, iii) please see below for some suggestions and questions about the GRADE approach which may influence the reporting of results in the abstract
Introduction: stating that “interventions to prevent falls” are cost-effective and effective in falls and fall injuries could be more specific especially since not all interventions have been shown successful and the authors are arguing for a review of CDS interventions. Possibly “a variety of interventions aimed at older adults” or such would help.*

Response 2

Thank you for these suggestions. We have added the search dates to the Methods section of the abstract, included a brief overview of the most used types of CDS, and reviewed the reporting of results in the abstract considering your suggestions regarding the GRADE approach. Please see the revised abstract. In the introduction, we have specified the types of fall prevention interventions that are cost-effective and effective in reducing falls and fall injuries. Please see pages 4-5, lines 103-107.

**Comment 3:**

*Materials and methods:
1. Was the protocol only ever “drafted”? even if there were changes post hoc the protocol should have been in some form of final version before starting the review.
2. Spell out PRISMA at first use
3. Selection process: please add details about who conducted full text review, and confirm whether consensus was required at title/abstract stage or just full text.
4. Data collection process: using verification for only 5 of 24 papers should be noted in the discussion as a limitation of the review (possibility for errors). Duplicate extraction or verification of all data is a standard for systematic reviews. This is particularly concerning if only one person chose which outcome data to use when studies reported on multiple outcomes.
5. Study risk of bias: please confirm if “the main outcome results” means the result data used for each outcome of interest to the review, or otherwise.
6. Synthesis methods: please add a section on unit of analysis issues, i.e. what was done to account for effects of clustering in cluster RCTs? Was the authors’ result adjusted for clustering used for analysis and, if not reported/performed, what was done?*

Response 3

1. Thank you for your comment. The protocol was drafted, finalised, and registered in PROSPERO before commencing the review. We have clarified this in the Methods section. Please see page 7, lines 152-156.

2. We have spelled out PRISMA at its first mention.

3. Thank you for your comment. We have added details about who conducted full-text review and clarified that consensus was required at both the title/abstract screening stage and the full-text screening stage. Please see page 9, lines 197-201.

4. Thank you for highlighting this issue. We have discussed the limited verification of collected data by a second reviewer as a limitation of the review in the Discussion section to acknowledge the possibility of errors. Additionally, we have clarified that the selection of each result was discussed and verified with the project statistician to ensure correctness and consistency. Please see page 38, lines 784-787.

5. We have specified which outcomes were assessed for risk of bias. Please see page 12, lines 253-254.

6. Thank you for your comment regarding unit of analysis issues. The cluster-randomised trials included in the review accounted for clustering in their original analyses. Additionally, in our syntheses, we ensured that only one outcome from each study was included, thus maintaining independence between studies in the analyses. We have included a sentence in the methods section to clarify this. Please see page 13, lines 291-292.

**Comment 4**

*Results/conclusions:
1. it would be of interest to comment on how many studies had eligibility criteria related to increased risk for falls (e.g. previous fallers, 1+ risk factor etc), and to state whether the findings overall are most applicable to populations at increased risk (this may vary by outcome to some degree),
2. it is hard to know whether the findings for the fall risk outcome may be most applicable to certain settings or ages; can the authors comment on whether the 10 studies appeared to capture both settings and ages fairly well? For example, if the large majority of the participants in the analysis came from studies undertaken in residential/acute care there could be some concerns about directness to the entire population of interest (eg rating down for indirectness may apply) or some way to clarify this observation for the reader,
3. likewise with setting and age groups for fall rates is there any indication that the studies in residential/acute care also enrolled older people to help know whether findings could be most applicable to both setting and age combined?,
4. conversion of ORs to absolute risk difference (for falls risk) should first convert the OR to a RR (see appendix 3 in https://www.bmj.com/content/389/bmj-2024-081904),
5. the authors should speak to their reason for use of a separate study (ref 88) for control event rates (rather than the studies themselves) and describe this study in some detail,
6. in figure 2 there are some “FU” and “follow-up” so this could be made consistent; further the authors should be able to put something into the figure for the medication outcome findings for the Blalock study, even if just a narrative statement by the authors about the direction of effect,
7. for mortality and hospitalizations the reversion to relying on statistical significance goes against what was done for other outcomes; perhaps the authors comment about any directions of effect, speak to the small sample sizes for these rare outcomes and point again to where folks can find the results if they want.
Line 561-2: would suggest deleting this sentence since the authors did not assess the magnitude of effects in their review*

Response 4:

1. Thank you for your comment. We have added details about how many studies included eligibility criteria related to increased fall risk in the results section. Please see pages 14-15, lines 337-339. Additionally, we have stated in the discussion section that the findings of this review are likely most applicable to populations at higher fall risk. Please see page 36, lines 743-745.

2. We have added details in the results section regarding the number of studies reporting fall risk that focused on different settings and age groups. These details suggest that the findings are largely applicable to the entire population of interest. Please see page 24, lines 446-449. Additionally, we have commented on the applicability of the findings from the meta-analysis on fall risk in the Discussion section. Please see page 33, lines 657-659.

3. Thank you for this comment. We have added details in the results section regarding the number of studies that included patients from different age groups within the hospitals or residential care subgroup. Please see pages 25-26, lines 488-492. Similarly, we have added details regarding the number of studies conducted in each setting within the subgroup by patients’ age. Please see page 27 lines 508-512. Additionally, we have commented on the applicability of the findings from the meta-analyses on fall rate in the Discussion section. Please see page 34, lines 677-679.

4. Thank you for your comment. Before calculating the absolute fall risk in the intervention group and the absolute risk difference, we have converted the ORs (including the point estimate and lower and upper limits of the confidence interval) to RRs using the formula provided in appendix 3 of https://www.bmj.com/content/389/bmj-2024-081904. This process has been described in the methods section for clarity. Please see page 13, lines 295-297.

5. Thank you for your comment regarding the use of a separate study for control event rates. Due to the limited reporting of absolute numbers in the studies included in this review, we derived control intervention risks and rates from a report describing the epidemiology of falls among older adults. This approach aligns with Cochrane methodology [1]. We have clarified this in the methods section and provided a description of the study used for control event risks and rates. Please see page 13, lines 293-295. Additionally, we updated the control fall risk from 30% to 28.7%, as reported in the study. Please see Table 4.

6. In figure 2, we have replaced all instances of ‘follow-up’ with ‘FU’ for consistency and added a description of the medication outcome results for the Blalock study.

7. We acknowledge your comment about the reliance on statistical significance and have revised the text to place greater emphasis on the direction of effect, the small sample size, and the rarity of the outcomes. Additionally, we have informed the reader where individual study results can be found for further details.

Line 561-2: Thank you for your feedback regarding the sentence: "Had more CDS tools been designed for automatic decision support it could potentially have increased healthcare practitioners’ adherence to recommendations." We have removed the sentence as suggested.

**Comment 5**

*Major comment:
GRADE does not assess the certainty of the point estimate per se but it’s relation to the “target” of certainty which should be defined in the methods section (see GRADE 34 guidance and J Clin Epidemiol. 2017 Jul:87:4-13. doi: 10.1016/j.jclinepi.2017.05.006 and BMJ. 2025 Apr 29:389:e081904. doi: 10.1136/bmj-2024-081904). For the provider outcomes, the target would need to be a direction of effect only since the analysis did not assess magnitude. For the patient outcomes, this could have been the null (direction of effect) or some form of a minimally important difference (even if approximate), especially to help determine whether findings are “little to no difference” or “an affect”. It appears the authors just used direction of effect and if so this should be mentioned and the findings should be stated in this light. It is confusing what the authors have done for this since there is no statement in the methods and for falls risk (OR 0.93, 21 fewer fallers per 1000) they state little to no effect in the results (implying 21 fewer is below some threshold of an MID) but a slight reduction in the abstract and discussion.
The choice of the target can change the GRADE ratings for all domains but in particular inconsistency and imprecision. For the provider outcomes, rating a conclusion of direction of effect where all studies showed the same direction would suggest against rating down for inconsistency; the size of effect doesn’t matter for this target. With some of the effects not being statistically significant/precise for the direction there could be concern over imprecision but it may not be serious. For direction of effect for adherence, I would think low certainty overall seems appropriate with the text indicating rating down twice for ROB. The same applies for medication outcomes, unless the authors want to have a third category of No direction which a couple of the findings indicate (e.g. Blum & Lightbody) and where some inconsistency in direction could be noted. If the authors really think low certainty for the direction is appropriate (vs moderate with just serious ROB) they could consider rating down twice for ROB or possibly once for ROB and once for imprecision (as above) or inconsistency (noting really that a direction was not shown in a couple of cases). For the falls risk outcome, if the authors are rating certainty in direction of effect, then not rating down for imprecision makes sense (assuming they wouldn’t be too strict on the upper limit of the 95% CI crossing the null slightly) and they would conclude there is an effect (possibly mentioning that it may not be important), but if they want to say there is “little to no” difference (implying use of an MID and that the point estimate is below this) then they likely should state what the MID was and make sure the entire 95% CI does not contain this value or else also rate down for imprecision. For the falls rate outcomes, if using a direction of effect the authors should note this in their conclusions whereas if they think the magnitude is at least as large as some small but important effect they may also rate down for either inconsistency (since ~30% of the weight in the analysis showed effects below what might be considered an MID) or for imprecision if the lower limit of the 95%CI (81 fewer falls) does not surpass their MID.
In summary, having a clear statement about what the authors were rating their certainty in (e.g. null/direction of effect) would be good as well as re-considering their assessments for the provider outcomes in light of this (not rating down for inconsistency). If using the direction of effect their falls risk findings should likely be “an effect”, with a comment that the effects may be small. If MIDs were applied these should be stated with the above considerations added.*

Response 5

Thank you for your detailed feedback regarding the use of GRADE to assess the certainty of evidence in this study. We appreciate your suggestions for clarifying our approach.

We graded the certainty of evidence based on the direction of effect rather than the MID. This decision was informed by the lack of established MID values for the outcomes of interest (fall risk, fall rate, and fall injury rate). Despite extensive literature searches conducted with the support of two librarians at OsloMet University, we were unable to identify absolute MID values for these outcomes, except for a single relative fall rate value specific to Parkinson’s patients (25%). Given the lack of absolute MID values applicable to fall risk, fall rate, and fall injury rate, we determined that assessing the certainty of evidence for the direction of effect was the most appropriate approach.

In response to your feedback, we have revised the methods section to explicitly state that we assessed certainty in the direction of effect, using the null effect as the threshold. We also re-evaluated our GRADE ratings to reflect this approach. For provider outcomes, we did not rate down for inconsistency where all studies showed the same direction of effect, as suggested. For the fall risk outcome, we rated down for imprecision because of the slight crossing of the null by the upper limit of the CI.

To ensure consistency in reporting, we revised the results and discussion sections, as well as the abstract, to frame findings in terms of the direction of effect. For example, we now describe the findings for fall risk as indicating “an effect,” while noting that the effects may be small. Additionally, we added a summary table of GRADE certainty for each outcome (Table 4) to facilitate quick reference. Finally, we added a note in the discussion to acknowledge the lack of established MID values and highlighted the need for future work to establish such thresholds for fall prevention outcomes. Please see page 36, lines 726-740.

**References**

1.	Schünemann HJ, Higgins JPT, Vist GE, Glasziou P, Akl EA, Skoetz N, et al. Chapter 14: Completing ‘Summary of findings’ tables and grading the certainty of the evidence. 6.4. Higgins JPT, Thomas J, Chandler J, Cumpston M, Li T, Page MJ, Welch VA (editors) Cochrane Handbook for Systematic Reviews of Interventions version 64 (updated August 2023) Cochrane, 2023 Available from www.training.cochrane.org/handbook. 6.4. Cochrane; 2023.
