# Supplementary material for: Effectiveness of clinical decision support in fall prevention among older adults: A systematic review and meta-analysis
Source: PLoS One. 2026 Jan 12;21(1):e0340025. doi: 10.1371/journal.pone.0340025 (PMC12795367; doi:10.1371/journal.pone.0340025)
Supplement: Attachment — Submitted filename: Response_to_Reviewers_auresp_3.docx [file pone.0340025.s024.docx]

**Response to Reviewers**

Dear Dr. Sascha Köpke

Thank you for the opportunity to submit a revised draft of our manuscript titled "Effectiveness of clinical decision support in fall prevention among older adults: a systematic review and meta-analysis" to PLOS ONE. We sincerely appreciate the time and effort you and the reviewers have invested in providing thoughtful and constructive feedback. We have carefully considered all comments and made revisions to address the suggestions provided.

All authors have reviewed and approved the submission of this revised manuscript. We confirm that the manuscript has not been published and is not under consideration for publication elsewhere, in whole or in part, in any language.

We hope that the revisions meet your expectations, and we look forward to the possibility of having our article accepted for publication.

Thank you for your time and consideration.

Yours sincerely

Rune Solli on behalf of the authors

**Response to Reviewer 2**

**Comment 1:**

*This manuscript addresses a highly relevant topic, considering the growing prevalence of falls among older adults and the urgent need for effective interventions. The systematic review and meta-analysis presented demonstrate methodological rigor, including a comprehensive search strategy across multiple databases, application of the GRADE approach, and appropriate use of meta-analytic techniques.

Strengths

Comprehensive and transparent search strategy.

Clearly defined inclusion criteria, encompassing different study designs.

Robust synthesis of the data, including both meta-analyses and risk of bias assessment.

Discussion aligned with the presented evidence, highlighting differences between settings (hospital/residential care versus community).

Suggestions for Improvement

Clarity on outcomes: The explanation of the difference between “fall rate” and “fall risk” could be made more concise and accompanied by practical examples to improve readability for a broader audience.

English language polishing: Although the manuscript has been revised, some sentences could be more fluent and precise. A professional language editing service is recommended.

Graphical presentation: Figures and tables have been improved, but further adjustments could increase readability, such as standardizing font size and enlarging legends, especially for risk-of-bias charts.

Practical conclusions: It would strengthen the paper to explicitly emphasize in the discussion the clinical and implementation implications across different settings (primary care versus hospital/residential), to enhance the translation of findings into practice.

Overall, this is a technically sound study with data that support its conclusions. It represents a valuable contribution to the literature on fall prevention in older adults. I recommend acceptance after minor revisions, mainly focusing on clarity of writing, conceptual distinction between outcomes, and minor graphical improvements.*

Response 1:

Thank you for your feedback. We have clarified the explanation of the difference between “fall rate” and “fall risk” in the Methods, Results, and Discussion sections. Please see page 10, lines 214-221; page 24, lines 438-439; and pages 33-34, lines 663-683. Additionally, we have included a practical example to enhance clarity, which can be found on page 33, lines 673-677.

Thank you for your comment regarding the language of the manuscript. As a reminder, the manuscript underwent professional language editing during the previous revision round. However, we have carefully reviewed the text again in this round to further improve fluency and precision. We hope that the revised manuscript meets the required expectations.

Graphical presentation: Thank you for this suggestion. We appreciate your feedback on improving the figures and tables. In the previous two rounds, we increased the font size and enlarged the legends for the risk-of-bias charts. We believe these figures are now clear, suitable for publication, and compliant with the journal’s submission guidelines regarding figures. However, please let us know if there are any specific aspects that still require further adjustments.

Practical conclusions: Thank you for this valuable suggestion. We have revised the discussion section to explicitly emphasize the clinical and implementation implications of our findings across different settings. Please see the updated Discussion section on page 38, lines 782-795.

**Response to Reviewer 3**

**Comment 1:**

*Thank you for resubmitting your revised manuscript, and you have taken into consideration the reviewers feedback. I do have some ongoing feedback /questions which is included in the document which is around:
1. The final number of included studies reported in the manuscript is different to that reported in the PRISMA diagram - please clarify the actual number.
2. Your eligibility criteria is confusing - your report only including studies based in healthcare settings, yet you included community dwelling studies?
3. I do not agree with all your interpretations about the results obtained - you indicate that CDS may reduce fall rate across all age groups and settings, yet this is not supported by your results. Please re-evaluate this.*

Response 1:

1. Thank you for your comment. To clarify, the PRISMA diagram shows that, from the primary literature searches, 45 reports were assessed for eligibility, of which 21 were excluded. Additionally, as described on the right side of the PRISMA diagram, nine reports were assessed for eligibility via other methods, including the Physiotherapy Evidence Database (PEDro), Google Scholar, chain searches, cited reference searches, and conference abstracts, of which five were excluded. As a result, a total of 28 publications from 25 unique studies were included in the review. We confirm that the inclusion of 28 publications from 25 unique studies is accurately reported in both the PRISMA diagram and the manuscript. We kindly ask for further clarification if there is a specific point we have misunderstood. Additionally, we have clarified the meaning of PEDro in the manuscript by writing it out in full upon its first mention.

2. Thank you for highlighting this discrepancy between the eligibility criteria in the main text and Table 1. We have revised the main text to clarify that studies conducted in any healthcare setting, as well as those conducted in the homes of older adults, were eligible for inclusion.

3. Thank you for your feedback. We have re-evaluated our interpretation of the results and revised the manuscript to ensure it does not suggest that CDS reduces fall rates across all age groups and settings. The updated Results section and revised conclusion now reflect this clarification.

**Response to Reviewer 4**

**Comment 1**

*Good revision. Only 2 minor comments for revision. 1. for the GRADE rating of injurious falls in the 80+ age category, there should only be one level rated down for imprecision since the effect shows harm (magnitude not relevant) with slight imprecision (0.99 lower CI limit). This won't change the overall GRADE but will add accuracy.*

Response 1

Thank you for this valuable point. We agree that only one level should be rated down for imprecision, as the confidence interval slightly crosses the null effect. We have made the necessary changes to the manuscript. Please see Table 4 and S9 Table.

**Comment 2**

*2. in the discussion, the beginning sentence of the patient outcomes section uses one of the 4 estimates for falls rates without a reason. Perhaps just state that the intervention may reduce fall risk (14 fewer) and fall rates (range 20-188 fewer), since you later discuss the differences across subgroups.*

Response 2

Thank you for pointing this out. We have revised the beginning sentence of the patient outcomes section of the Discussion as suggested, and it now states that our meta-analyses suggested a possible reduction in fall risk (ranging from 32 fewer to three more fallers) and fall rates (ranging from 20 to 188 fewer falls).
